# Intracellular iron accumulation facilitates mycobacterial infection in old mouse macrophages

**DOI:** 10.1007/s11357-023-01048-1

**Published:** 2023-12-30

**Authors:** Stephen K. Kotey, Xuejuan Tan, Owen Fleming, Ramakrishnama Raju Kasiraju, Audrey L. Dagnell, Kyle N. Van Pelt, Janet Rogers, Steven D. Hartson, Nidheesh Thadathil, Ramasamy Selvarani, Rojina Ranjit, Sreemathi Logan, Sathyaseelan S. Deepa, Arlan Richardson, Yong Cheng

**Affiliations:** 1 Department of Biochemistry and Molecular Biology, Oklahoma State University, 246 Noble Research Center, Stillwater, OK 74078 USA; 2 Oklahoma Center for Respiratory and Infectious Diseases, Oklahoma State University, Stillwater, OK USA; 3 Center for Genomics and Proteomics, Oklahoma State University, Stillwater, OK USA; 4 Department of Biochemistry & Molecular Biology, University of Oklahoma Health Sciences Center, Oklahoma City, OK USA; 5 Okalahoma City Veteran Affairs Medical Center, Oklahoma City, OK USA

**Keywords:** Macrophages, Aging, Mice, Non-tuberculous Mycobacteria, Intracellular iron, Phagolysosome maturation

## Abstract

Aging has a significant impact on the immune system, leading to a gradual decline in immune function and changes in the body’s ability to respond to bacterial infections. Non-tuberculous mycobacteria (NTM), also known as atypical mycobacteria or environmental mycobacteria, are commonly found in soil, water, and various environmental sources. While many NTM species are considered opportunistic pathogens, some can cause significant infections, particularly in individuals with compromised immune systems, such as older individuals. When mycobacteria enter the body, macrophages are among the first immune cells to encounter them and attempt to engulf mycobacteria through a process called phagocytosis. Some NTM species, including *Mycobacterium avium* (*M. avium*) can survive and replicate within macrophages. However, little is known about the interaction between NTM and macrophages in older individuals. In this study, we investigated the response of bone marrow–derived macrophage (BMMs) isolated from young (5 months) and old (25 months) mice to *M. avium* serotype 4, one of the main NTM species in patients with pulmonary NTM diseases. Our results demonstrated that BMMs from old mice have an increased level of intracellular iron and are more susceptible to *M. avium* serotype 4 infection compared to BMMs from young mice. The whole-cell proteomic analysis indicated a dysregulated expression of iron homeostasis–associated proteins in old BMMs regardless of mycobacterial infection. Deferoxamine, an iron chelator, significantly rescued mycobacterial killing and phagolysosome maturation in BMMs from old mice. Therefore, our data for the first time indicate that an intracellular iron accumulation improves NTM survival within macrophages from old mice and suggest a potential application of iron-chelating drugs as a host-directed therapy for pulmonary NTM infection in older individuals.

**Supplementary Information:**

The online version contains supplementary material available at 10.1007/s11357-023-01048-1.

## Introduction

Macrophages are key players in the immune response, including inflammation. With aging, there is often a phenomenon known as “inflammaging” which refers to chronic, low-level inflammation that occurs as a result of the aging process [1]. Macrophages are implicated in this process, as they can shift from their normal protective functions to a state of chronic activation, leading to the release of pro-inflammatory molecules. This chronic inflammation is believed to contribute to various age-related diseases, such as cardiovascular disease, neurodegenerative disorders, and even cancers [2]. Aging can impair the phagocytic capacity of macrophages, affecting their ability to clear cellular debris and pathogens. This decline in phagocytic function can lead to a compromised immune response and increased susceptibility to infections [3]. Aging leads to immune system changes, including a decline in immune function known as immunosenescence [4]. Macrophages, as part of the immune system, play a critical role in age-related immunosenescence [5, 6]. However, the mechanism by which macrophages contribute to aging progression in humans remains to be defined.

Older individuals are generally more susceptible to bacterial lung infections due to immunosenescence. Immunosenescence can lead to a reduced ability to recognize and respond to pathogens, making older individuals more vulnerable to infections [7]. Non-tuberculous mycobacteria (NTM) are a group of bacteria related to *Mycobacterium tuberculosis*, the causative agent of tuberculosis (TB), but do not cause TB [8]. Instead, NTM can cause a range of infections, particularly in the older population or people with compromised immune systems or underlying lung conditions [9]. The most common manifestation of NTM infection is lung disease (NTM-PD), and the global burden of NTM-PD infection is increasing [10, 11]. As we mentioned above, macrophages are the primary cells responsible for recognizing and engulfing invading pathogens, including NTM. Upon encountering NTM, macrophages attempt to phagocytose (engulf) the bacteria. However, NTM evolved strategies to manipulate macrophage responses and evade immune detection [12, 13]. Some NTM species, such as *Mycobacterium avium* (*M. avium*), can alter the maturation of the phagosome, hindering lysosomal fusion and thus avoiding exposure to destructive enzymes [12]. This enables the bacteria to persist within macrophage and potentially disseminate throughout the body. *M. avium* is one of the main NTM species that were identified in the old population with NTM-PD [9, 14, 15]. However, little is known about the mechanism by which macrophages fail to control *M. avium* intracellular infection in older individuals and aging animal models.

Iron is an essential element for the growth and metabolism of most bacteria, including intracellular pathogens. Intracellular bacteria have evolved various strategies to acquire iron from hostile environment within host cells [16, 17]. They may produce specialized iron-chelating molecules called siderophores to scavenge iron from host proteins. Some bacteria can also exploit host iron-transport systems to import iron. By acquiring sufficient iron, intracellular bacteria can support their replication and survival within host cells. Iron acquisition can also enhance the virulence of intracellular pathogens, allowing them to evade host immune responses and establish persistent infections [16]. To control intracellular bacterial infection, host cells employ several strategies to limit bacterial access to iron. This includes the production of iron-binding proteins, such as ferritin and lactoferrin, which sequester iron and prevent its availability to bacteria. In contrast, intracellular iron accumulation in host cells can inadvertently promote bacterial growth, and excess iron can provide a favorable environment for bacterial pathogens to flourish [18]. Understanding the interactions between intracellular bacteria and iron metabolism within host cells can provide insights into potential therapeutic targets for treating bacterial infections. Developing strategies to disrupt bacterial iron acquisition or utilization may help control infections.

In the current study, we investigated *M. avium* survival in bone marrow–derived macrophages (BMMs) isolated from young (5 months) and old (25 months) mice and found that BMMs from old mice are more susceptible to *M. avium* infection in cell culture. Whole-cell proteomic analysis further identified a dysregulated cellular response such as intracellular iron homeostasis in old mouse BMMs compared to young mouse BMMs regardless of *M. avium* infection. The defect of old mouse BMMs in controlling intracellular *M. avium* can be rescued by iron chelator, deferoxamine (DFO). Taken together, our data indicate that an increased accumulation of intracellular iron in old mouse macrophages make them more susceptible to *M. avium* infection.

## Methods

### Mice

Wild-type young (5-month old, female) and old (25-month old, female) C57BL/6 mice were used for all experiments. All mice were generated and housed under specific pathogen-free and barrier conditions in the institutional animal facility at Oklahoma City VA Medical Center which is accredited through the Animal Welfare Assurance (#A3361-01). All animal experiments were approved by the Institutional Animal Care and Use Committees (IACUC #1676288 and # 22068) of the Oklahoma City VA Medical Center and the University of Oklahoma Health Sciences Center.

### Macrophage culture

Mouse BMMs were prepared from femur bones of young (*n* = 9) and old (*n* = 9) mice as we did previously [19]. Briefly, BMMs were subjected to differentiation by culturing in high glucose DMEM (Gibco, Cat. no. SH30243.01) supplemented with 10% fetal bovine serum (Gibco, Cat. no. 10438–026), 20% L929 conditional medium, and 1X pen-strep (Fisher Scientific, Cat. no. SV30010) for 7 days at 37 °C with 5% CO_2_.

### Bacterial strains

*M. avium* serotype 4 and tdTomato-expressing *M. avium* were grown in Middlebrock 7H9 broth (Difco™, Cat. no. 271310) media supplemented with 10% Middlebrook OADC and 0.5% glycerol (Fisher Chemical, Cat. no. G33-500) until mid-exponential phase before use as we did previously [20, 21]. For the *M. avium* infection assay, the *M. avium* cells were washed with complete BMM medium for 3 times before infection.

### *M. avium* survival assay in BMMs

BMMs from young and old mice were seeded at the density of 2 × 10^5^ cells per well in 96-well TC-treated plates (Corning Costar, Cat. no. 07–200-90) at 37 °C and 5% CO_2_. Pre-seeded cells were infected with *M. avium* serotype 4 at an MOI of 10 for 1 h and then washed thrice with complete BMM medium. Washed cells were incubated in complete BMM medium for an additional 1, 24, and 72 h at 37 °C and 5% CO_2_. At each time point, infected cells were washed three times with ice-cold PBS and lysed with 0.05% SDS. The cell lysates were then serially diluted and spread onto 7H10 agar plates (HiMedia, Cat. no. M199-500G) supplemented with 10% OADC and 0.5% glycerol. The plates were incubated at 37 °C for 7 days before counting. For the iron chelator assay, the experiment was performed in the presence of deferoxamine mesylate salt (Sigma Aldrich, Cat. no. D9533-1G) with a final concentration of 200 µM.

### Mouse BMM growth assay

Mouse bone marrow was prepared as described above, and cell growth was determined after a 7-day incubation in the complete BMM medium in vitro at 37 °C and 5% CO_2_. Bright-field images of random fields of the cells were taken using EVOS M5000 Fluorescence Microscope. Cell numbers per field were counted and quantified.

### RNA isolation and cDNA synthesis

Total RNA was isolated from the uninfected or *M. avium*–infected mouse BMMs using Monarch® Total RNA Miniprep Kit (NEB, Cat. No. T2010S) following the manufacturer’s protocol. RNA concentration and purity were measured using a Nanodrop ND-1000 Spectrophotometer (Marshall Scientific, Cat. no. ND-1000). cDNA was synthesized using NEB AMV Reverse Transcriptase (NEB, Cat. no. M0277) according to the manufacturer’s protocol. The cDNA samples were immediately stored at − 20 °C until use.

### Quantitative RT-PCR

Prepared cDNAs were used in the quantitative RT-PCR with Luna® Universal qPCR Master Mix (NEB, Cat. No. M3003) following the manufacturer’s instruction. The RT-PCR was run in Roche’s Lightcycler® 96 machine. GAPDH (glyceraldehyde-3-phosphate dehydrogenase) was used as a load control. The relative expression level of the genes of interest was calculated using the uninfected young BMMs as a reference. Primers used in the qRT-PCR are listed below: GAPDH_FW, 5′-TCGTCCCGTAGACAAAATGG-3′; GAPDH_RE, 5′-TTGAGGT CAATGAAGGGGTC-3′; TNF-α_FW, 5′-ACGGCATGGATCTCAAAGACA-3′; TNF-α_RE, 5′-CTG ACGGTGTGGGTGAGGA-3′; IL-1β_FW, 5′-TACAGGCTCCGAGATGAACAA-3′; IL-1β_RE, 5′-CT TGTACAAAGCTCATGGAGAA-3′; IL-6_FW, 5′-AACTCTAATTCATATCTTCAACCA-3′; IL-6_RE, 5′-GGTCCTTAGCCACTCCTTCT-3′; IL-10_FW, 5′-AAGACAATAACTGCACCCACTT-3′; IL-10_RE, 5′-TCCTGCATTAAGGAGTCGGTTA-3′; Arg-1_FW, 5′-CTAATGACAGCTCCTTTCAAAT T-3′; Arg-1_RE, 5′-GATGCTTCCAACTGCCAGACT-3′.

### Intracellular iron concentration measurement

Mouse BMMs were seeded in triplicates at a density of 2 × 10^5^ cells/well in 96-well plate at 37 °C and 5% CO_2_. The cells were then uninfected or infected with *M. avium* at an MOI of 10 for 24 h. An iron assay kit (Sigma-Aldrich, Cat. No. MAK025) was used to determine the iron abundance in BMMs following the manufacturer’s protocol. The absorbance at 593 nm (*A*_593_) was measured. A standard curve was obtained from the absorbance values of the standards against their concentrations (ng/µl). The concentration of intracellular iron was determined using the equation of the standard curve.

### Sample preparation for whole-cell proteomics

BMMs from young and old mice were pre-seeded at a cell density of 8 × 10^5^ cells per well in 12-well TC-treated plates (Fisherbrand, Cat. no. FB012928) at 37 °C and 5% CO_2_. Pre-seeded cells were then infected with *M. avium* serotype 4 at an MOI 10 and incubated at 37 °C and 5% CO_2_ for 24 h as we did previously [20]. After infection, the cells were washed with pre-cold HyClone Dulbecco’s phosphate buffer saline (Cytiva, Cat. no. SH30013.02) thrice and then detached by treating the cells with trypsin (Corning, Cat. no. 25–053-Cl). The whole-cell proteome was analyzed in the Proteomics Core Facility at Oklahoma State University in Stillwater.

### Liquid chromatography mass spectrometry

Uninfected or *M. avium*–infected BMM cell pellets were dissolved in the solution with 0.1 M Tris–HCl (pH 8.5), 8 M urea, and 10 mM Tris (2-carboxyethyl) phosphine hydrochloride. After incubation at RT (room temperature) for 60 min, the cell lysates were treated with 10 mM iodoacetamide for 30 min in the dark at RT. Alkylation reactions were then diluted fivefold with 100 mM Tris–HCl (pH 8.5), and samples were digested overnight with 2 µg of trypsin/LysC (Promega, Cat. No. V5072) at 37 °C. After digestion, an additional 1°g of trypsin/LysC was added, and the samples were treated for another 4 h. Peptide digests were then acidified in 1% trifluoroacetic acid (Sigma Aldrich, Cat. No. 299537) and desalted on C18 spin columns (HMMS18R, Nest Group) following the manufacturer’s handbook.

Peptides were re-dissolved in mobile phase A (0.1% aqueous formic acid) and separated using a 75-micron × 50-cm capillary column packed with 2-micron PepMap C18 beads (Thermo, Cat. No. PN164942) configured for trap-column (Thermo, Cat. No. PN164705) sample injections. Peptide separations utilized acetonitrile/water/formic acid (80:20:0.1) as mobile phase B, applied as a 4–33% gradient during the 60-min chromatography separation. Eluting peptides were ionized using a stainless-steel emitter in a Nanospray Flex ion source (Thermo Fisher). Peptide ions were analyzed in an Orbitrap Fusion mass spectrometer (Thermo Fisher) using a 5 Hz “high/low” data-dependent MS/MS strategy, in which an MS1 survey scan was performed in the Orbitrap sector at 120,000 nominal resolution, followed by quadrupole selection of + 2 to + 6 peptide ions with a dynamic exclusion repetition count of one, HCD fragmentation at 32% energy, and rapid-rate MS/MS fragment ion scans in the ion trap sector.

### Proteomic analysis and label-free quantitation

The raw MS data were searched using the analysis software MaxQuant (version 2.2.0.0) [22] against the database comprised of 55,311 Mus musculus protein sequences (Uniprot Version UP000000589) that were downloaded from Uniprot. Searches utilized the default MaxQuant settings, supplemented with deamidation of N/Q, and pyroglutamate cyclization of Q. The MaxQuant “match between runs” feature was enabled to propagate peptide identifications between LC–MS/MS runs [22]. LFQ intensity value of each sample was extracted with MaxQuant’s Perseus application. Potential contaminants and reverse proteins were filtered out. To create a normal distribution from the datasets, a log_2_ transformation was made. Statistically significant variations were acquired by applying a log_2_–fold-change (≥ 1.0 or ≤  − 1.0) and a *P* value equal or less than 0.05. Also, proteins that were unique to each of the samples were sorted by filtering out proteins that appeared in at least two out of the three replicates of one sample but absent or only detected in one replicate in the other sample. A false discovery rate (FDR) of 0.05 was used in the generation of volcano plots while sample *t* test analyses for pairwise analyses. Pathway enrichment was performed in Metascape (https://www.metascape.org) [23]. The custom analysis setting using only GO Biological Processes was selected.

### Confocal fluorescent microscopy analysis

Young and old mouse BMMs (3 × 10^5^) were pre-seeded onto glass coverslips at 37 °C and 5% CO_2_. Pre-seeded cells were infected by tdTomato-expressing *M. avium* strain at an MOI of 10 and incubated at 37 °C, 5% CO_2_ for 4 h, followed by three washes with incomplete DMEM medium. Washed cells were then incubated in complete BMM medium at 37 °C and 5% CO_2_ for another 24 h. After infection, the cells were fixed in 4% paraformaldehyde (PFA) for 2 h and subsequently permeabilized with 0.2% Triton X-100 (FisherBiotech, CAS 9002–93-1) in PBS for 20 min at RT. Acidified comparts of the cells were stained with 75 µM LysoTracker™ Green DND-26 (Thermo Fisher Scientific, Cat. No. L7526) for 30 min. The colocalization of tdTomato-expressing *M. avium* and LysoTracker was visualized and quantified using ZEISS LSM 980 confocal microscope as did previously [24] in the microscopy core facility at Oklahoma State University in Stillwater.

### Statistical analysis

The data obtained were analyzed by one-way ANOVA. A value of *P* ≤ 0.05 was considered significant. The computer program GraphPad PRISM 9.5.0 was used for the analysis.

### Data availability

The mass spectrometry proteomics data have been deposited to the ProteomeXchange Consortium via the PRIDE [25] partner repository with the dataset identifier PXD045821″. 

## Results

### BMMs from old mice have a defective protection against *M. avium* infection

**Fig. 1:**
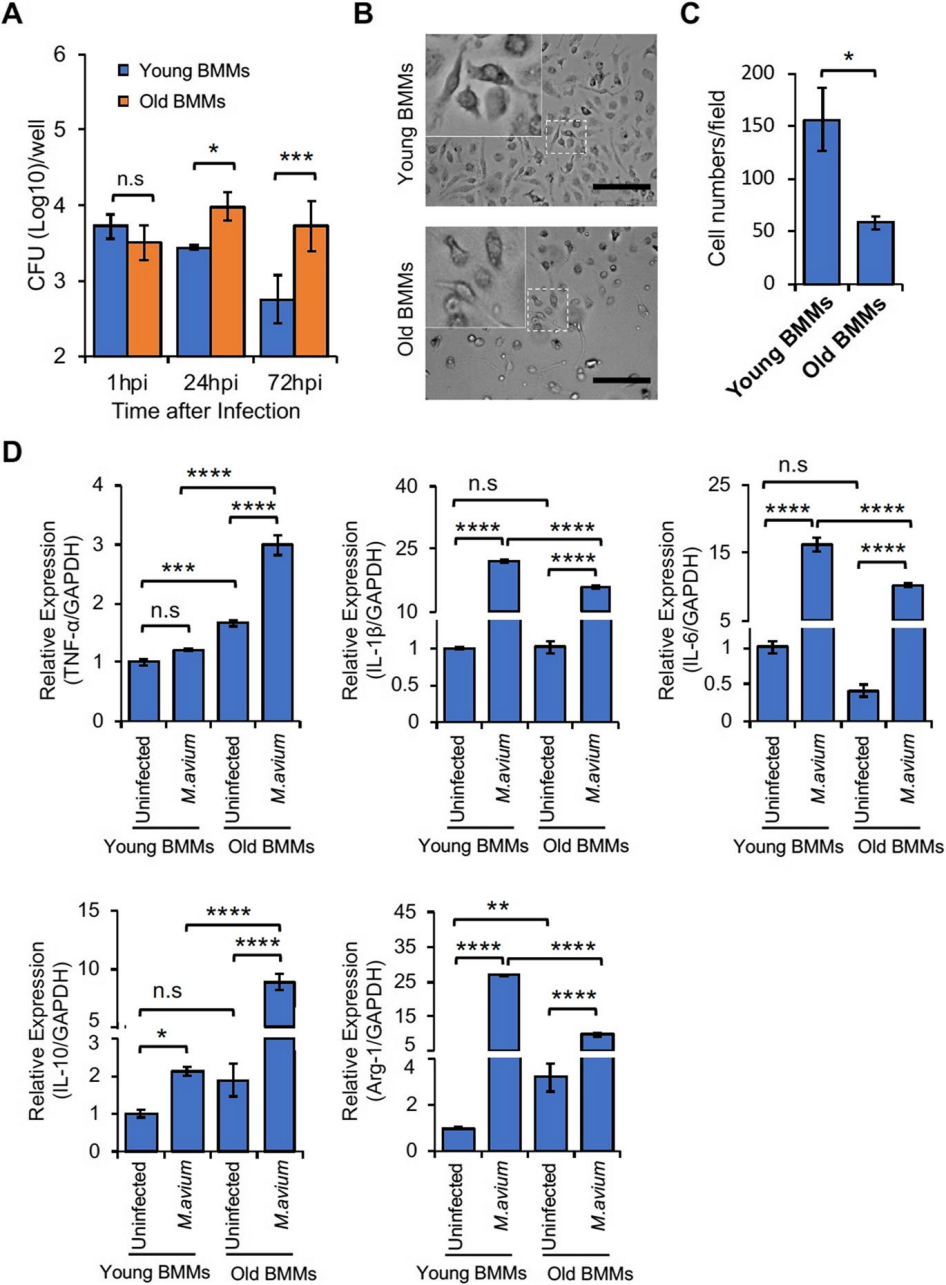
Mouse BMMs response to *M. avium* infection in vitro. **A**) *M. avium* survival assay. BMMs from young and old mice were infected with *M. avium* in cell culture at a MOI = 5 and then mycobacterial number within BMMs was determined at 1, 24, and 72 h post-infection. **B**) Microscopy images for mouse BMMs in cell culture. **C**) Quantitative analysis for microscopy images in (**B**). **D**) Similar to (**A**), but qRT-PCR analysis for TNF-α, IL-1β, IL-6, IL-10, and Arg-1 in BMMs uninfected or infected with *M. avium* for 24 h. Each transcript level was normalized to *Gapdh* and was expressed as fold change relative to uninfected young BMMs. In (**A**) and (**D**), data are presented as the mean ± SD (*n* = 3). All the results are representative of 3 independent experiments. n.s., not statistically significant; *p < 0.05, **p < 0.01, ***p < 0.001, and ****p < 0.0001 by one-way ANOVA, followed by Tukey’s post hoc test

To investigate if aging affects the antimycobacterial activity of macrophages, we infected BMMs from young and old mice using *M. avium* in vitro in cell culture system. As seen in Fig. 1A, a higher number of *M. avium* was detected in BMMs from old mouse at 24 and 72 h post-infection when compared to BMMs from young mouse. Interestingly, there was no significant difference in *M. avium* number within BMMs from old and young mice at 1 h post-infection, indicating that aging does not change the phagocytic activity of mouse BMMs to uptake *M. avium* in cell culture. To better understand the in vitro function of BMMs from old mice, we also measured BMM proliferation in cell culture. As shown in Fig. 1B and 1C**,** relative to BMMs from young mice, an attenuated cell proliferation was observed in BMMs from old mice after 1 week of incubation in cell culture.

As we described above, macrophages are professional phagocytes that can uptake and destroy invading bacteria. Under infection conditions, macrophages can exhibit different activation states or phenotypes based on the signals they receive. These activation states are often classified into two broad categories: the classically activated (M1) and the alternatively activated (M2) macrophages, which also play a critical role in aging process [26–28]. M1 and M2 macrophages have distinct antimycobacterial activity in tissue. To determine if *M. avium* infection induces a differential M1 and M2 macrophage activation in BMMs from old mice, we measured the expression of M1 (TNF-α, IL-1β, and IL-6) and M2 (IL-10 and Arg-1) marker genes in BMMs from young and old mice that were uninfected or infected with *M. avium* for 24 h. As seen in Fig. 1D, *M. avium* infection significantly induced the expression of all five genes in BMMs from old mice. The expression of TNF-α and IL-10 was much higher in *M. avium*–infected BMMs from old mice compared to infected BMMs from young mice. In contrast, the expression of IL-1β, IL-6, and Arg-1 was lower in *M. avium*–infected BMMs from old mice compared to infected BMMs from young mice. Therefore, the quantitative RT-PCR results indicate a dysregulated macrophage activation in BMMs from old mice during *M. avium* infection.

### Differential cellular activity in naïve BMMs from old mice relative to naïve BMMs from young mice

**Fig. 2:**
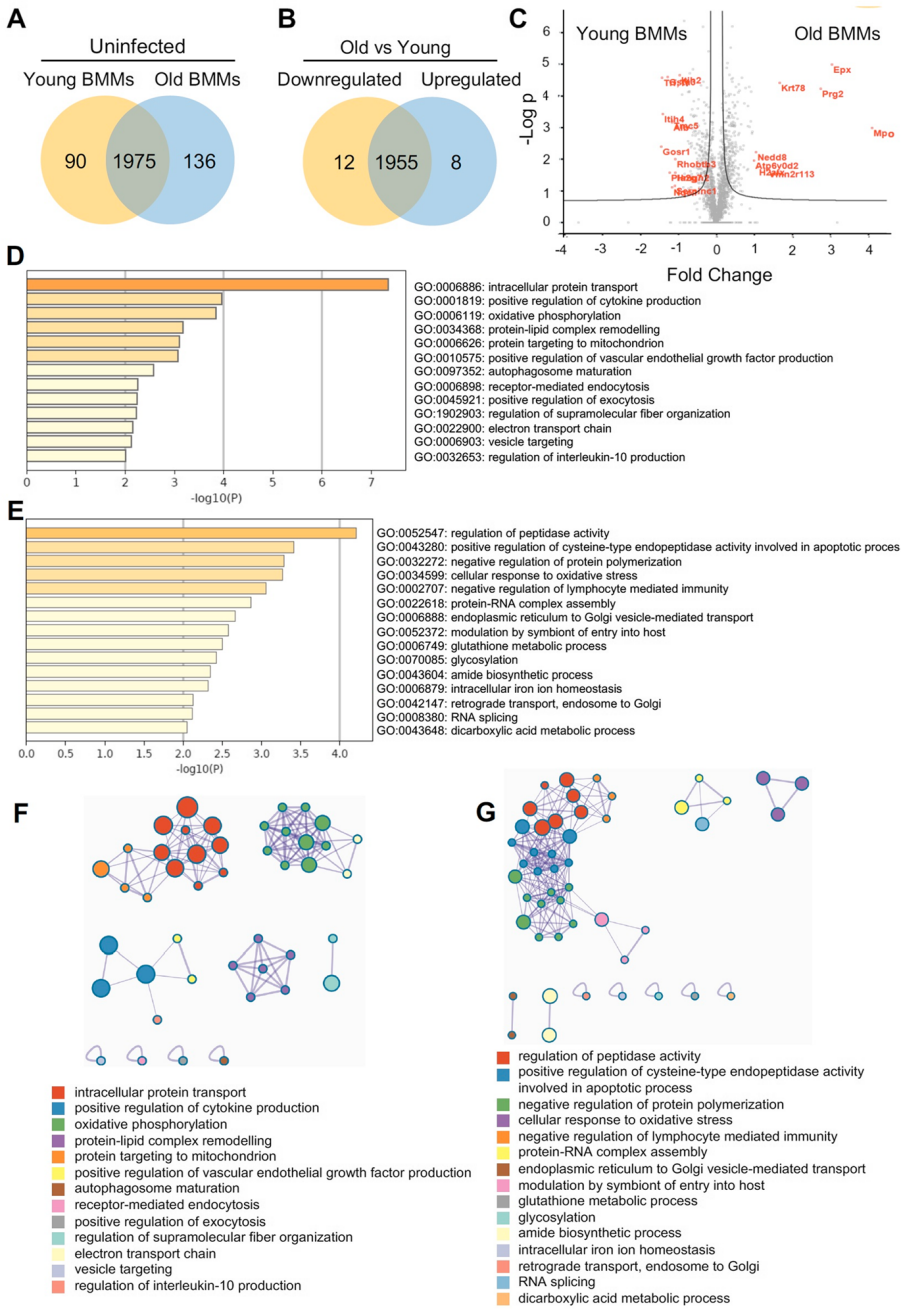
Proteomic and Go enrichment analysis for uninfected BMMs from young and old mice. **A**) Venn diagram for unique and overlapping proteins in young or old BMMs (*n* = 3/group). **B**) Similar to (**A**), but Venn diagram for differentially regulated proteins between young and old BMMs in overlapping proteins (threshold: twofold change and *P* value ≤ 0.05). **C)** Volcano plot for proteins identified in young or old BMMs. **D**) The Go pathway analysis using differentially upregulated proteins in old BMMs vs. young BMMs. **E**) Similar to (**D**), but the downregulated proteins in old BMMs. **F**) Metascape network for enriched ontology clusters based on upregulated proteins in old BMMs vs. young BMMs. **G**) Similar to (**F**), but for downregulated proteins in old BMMs

To further understand the cellular response to *M. avium* infection in BMMs from young and old mice, we performed a whole-cell proteomic analysis and compared the protein profiles in young and old BMMs that were uninfected or infected with *M. avium* in cell culture. As shown in Fig. 2A, we identified 2111 and 2065 proteins in uninfected BMMs from old and young mice, respectively. Among those, 1975 proteins were identified in BMMs from uninfected old and young mice, 136 unique proteins in uninfected old BMMs (Supplementary Table 1A), and 90 unique proteins in uninfected young BMMs (Supplementary Table 1B). Among 1975 overlapping proteins, the abundance of 8 proteins was upregulated and 12 proteins downregulated in old BMMs (threshold: twofold change; *P* value ≤ 0.05) when compared to young BMMs (Fig. 2B and Supplementary Table 1C). A comparable protein level was found for 1955 proteins (Supplementary Table 1D) in uninfected BMMs from old and young mice. The volcano plot in Fig. 2C illustrates differentially abundant proteins analyzed in Fig. 2B that are significantly different in uninfected BMMs from old and young mice. The full list of these proteins is available in Supplementary Table 1A–1D. To determine the engagement of these differentially expressed proteins in age-associated changes of cellular pathways in macrophages, we performed Go Enrichment Pathway Analysis using the upregulated protein list in old BMMs (136 in Fig. 2A and 8 in Fig. 2B; Supplementary Table 1A and 1C) via the online pathway analysis tool, Metascape. As shown in Fig. 2D, 13 pathways were enriched for biological process, such as protein targeting to mitochondria and autophagosome maturation. We also found 15 enriched pathways for biological process (Fig. 2E) when we analyzed downregulated protein list in old BMMs (90 in Fig. 2A and 12 in Fig. 2B; Supplementary Table 1B and 1C), including intracellular iron homeostasis pathway, which is involved in mycobacterial intracellular survival and replication within macrophages [17, 29]. We further performed Metascape network analysis for enriched ontology clusters as described previously [23]. As shown in Fig. 2F and G, the enriched pathways are identified in various ontology databases. The top 4 enriched pathways show clear intra-cluster similarity in the upregulated proteins in uninfected BMMs from old mice (Fig. 2F). In the downregulated proteins in uninfected old BMMs, the top 4 enriched pathways show both intra-cluster and inter-cluster similarity (Fig. 2G).

### *M. avium* infection induces differential cellular responses in BMMs from old mice compared to infected BMMs from young mice

**Fig. 3:**
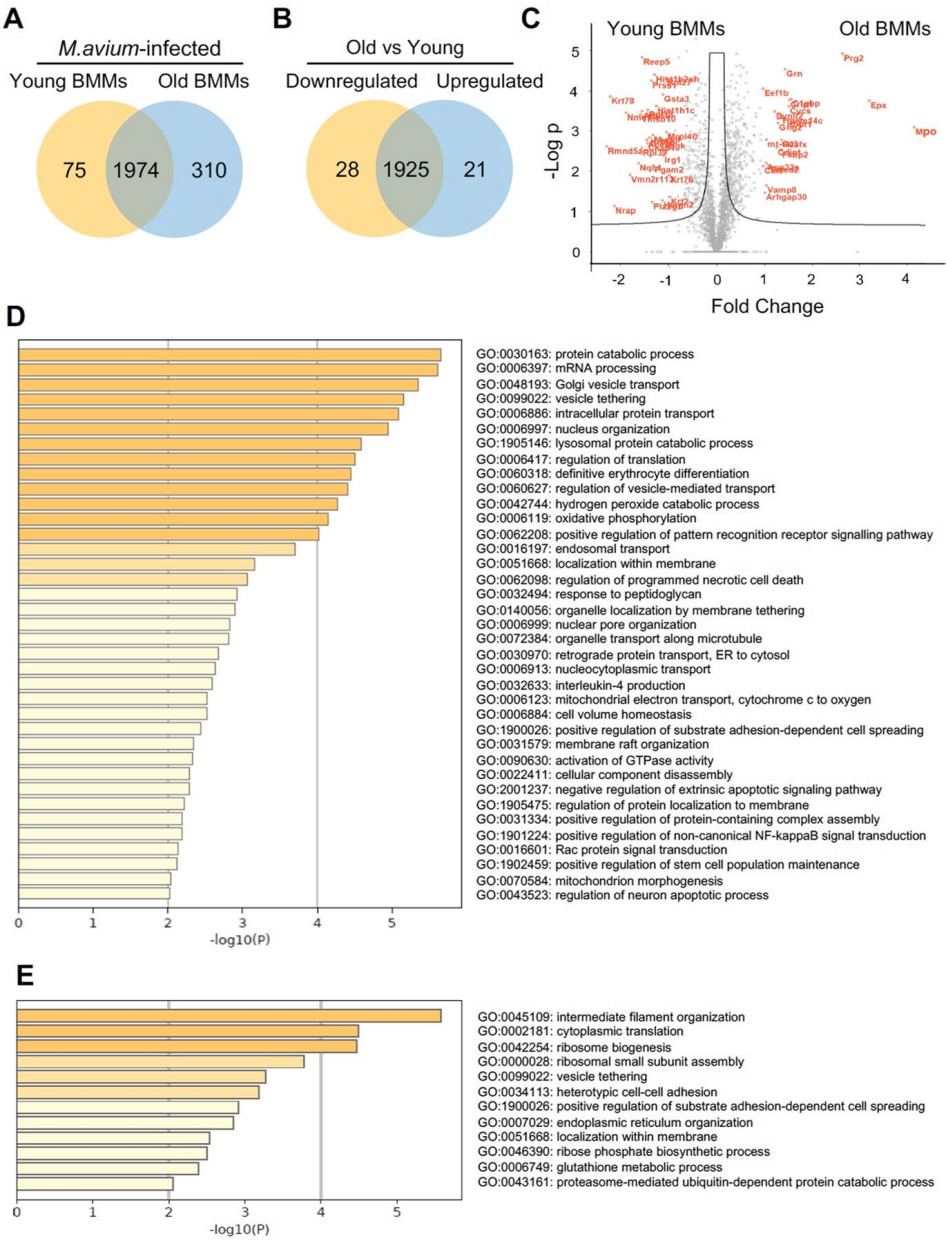
Proteomic and Go enrichment analysis for BMMs from young and old mice after *M. avium* infection. **A**) Venn diagram for unique proteins in *M. avium*–infected young or old BMMs (*n* = 3/group) at 24 h post-infection. **B**) Similar to (**A**), but Venn diagram for differentially regulated proteins between *M. avium*–infected young and old BMMs (threshold: twofold change and *P* value ≤ 0.05). **C**) Volcano plot for proteins identified in *M. avium*–infected young or old BMMs. **D**) The Go pathway analysis for the upregulated proteins in *M. avium*–infected old BMMs vs. *M. avium*–infected young BMMs. **E**) Similar to (**D**), but the downregulated proteins in *M. avium*–infected old BMMs

**Fig. 4:**
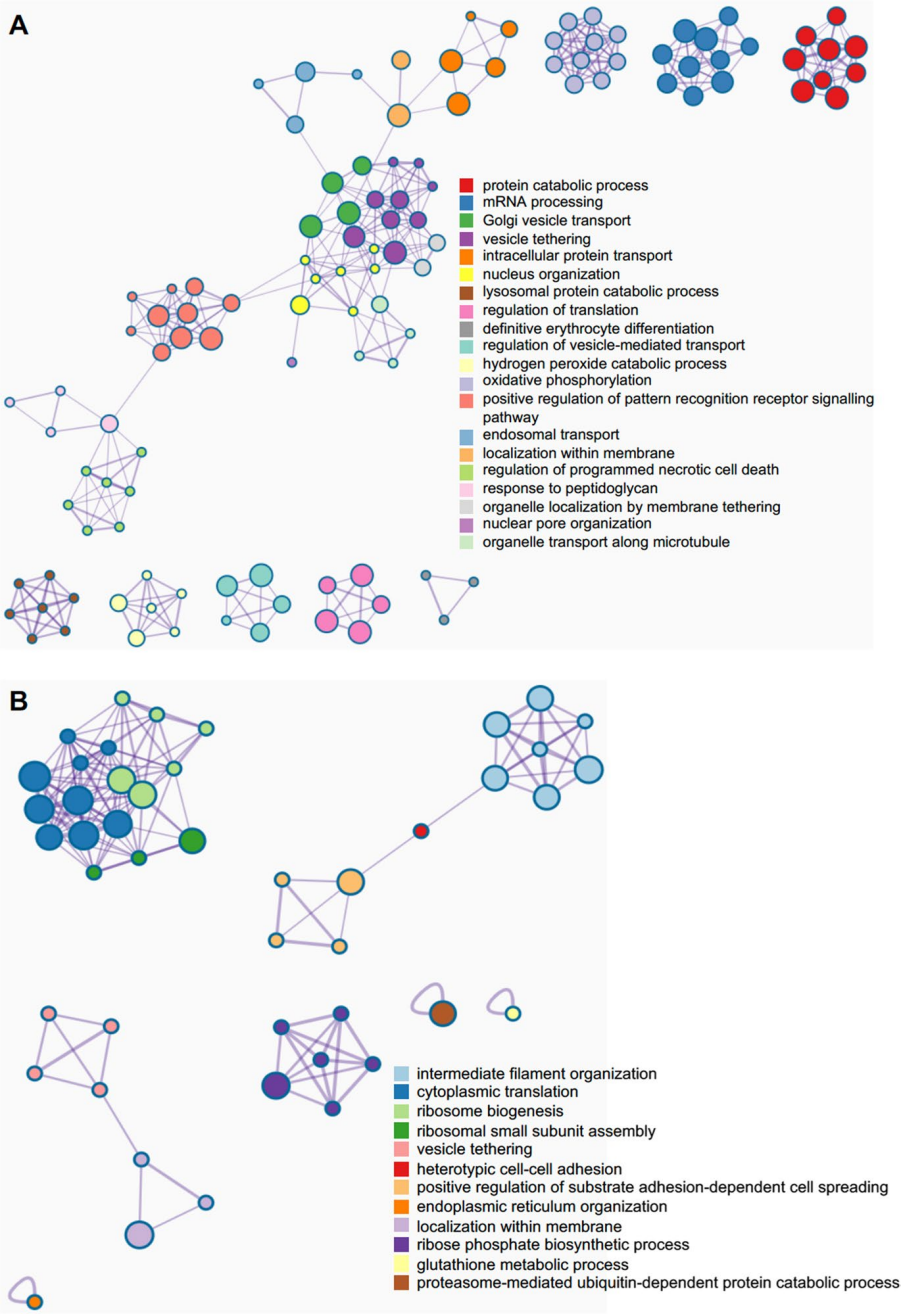
Metascape network for enriched ontology clusters. The analysis was performed using proteins that were differentially expressed in *M. avium*–infected old BMMs vs. infected young BMMs at 24 h post-infection. **A**) Metascape network for enriched ontology clusters based on the upregulated proteins in *M. avium*–infected old BMMs vs. *M. avium*–infected young BMMs. **B**) Similar to (**A**), but using the downregulated proteins in *M. avium*–infected old BMMs. Each term is indicated by a circular node. The number of input proteins falling into that term is represented by the circle size, and the cluster identities are represented by colors

We also analyzed the differentially expressed proteins in *M. avium*–infected BMMs from old and young mice (Fig. 3). As shown in Fig. 3A, we identified 2284 and 2049 proteins in *M. avium*–infected old and young BMMs, respectively. Among those, 310 proteins are unique in *M. avium*–infected old BMMs (Supplementary Table 2A), 75 proteins are unique in *M. avium*–infected young BMMs (Supplementary Table 2B), and 1974 proteins are identified in both infected old and young BMMs. Among 1974 overlapping proteins, the abundance of 21 proteins were upregulated, and 28 proteins were downregulated (threshold: twofold change; *P* value ≤ 0.05) in *M. avium*–infected old BMMs compared to infected young BMMs (Fig. 3B and Supplementary Table 2C). There was no significant difference in 1925 protein abundance (Supplementary Table 2D) between *M. avium*–infected old and young BMMs. The volcano plot in Fig. 3C illustrates differentially expressed proteins that are significantly different in *M. avium*–infected old and young BMMs that were included in Fig. 3B. The full list of these proteins is available in Supplementary Table 2A–2D. Similar to Fig. 2D, we also analyzed the Go pathway enrichment using Metascape. As seen in Fig. 3D, we identified 37 enriched Go pathways based on the upregulated protein list in *M. avium*–infected old BMMs (310 in Fig. 3A and 21 in Fig. 3B; Supplementary Table 2A and 2C), and 12 enriched Go pathways (Fig. 3E) based on downregulated protein list (75 in Fig. 3A and 28 in Fig. 3B and (Supplementary Table 2B and 2C). Among 37 upregulated pathways in *M. avium*–infected old BMMs, there are two mitochondria-related pathways: mitochondrial electron transport (GO: 0006123) and mitochondrion morphogenesis (GO: 0070584) that dysregulate cellular functions in aging cells [30–32]. A number of pathways involved in host response to microbial infections [33–35] were also enriched in *M. avium*–infected BMMs from old mice, including lysosomal protein catabolic process (GO: 1905146), hydrogen peroxide catabolic process (GO: 0042744), pattern recognition receptor signaling (GO: 0062208), programmed necrotic cell death (GO: 0062098), apoptotic signaling (GO: 2001237), and non-canonical NF-KappaB signaling (GO: 1901224). Metascape network analysis for enriched ontology clusters shows that the top 20 upregulated present a high inter-cluster and intra-cluster similarity (Fig. 4A). A similar result was observed for 12 downregulated pathways in *M. avium*–infected old BMMs relative to infected young BMMs (Fig. 4B).

### Intracellular iron homeostasis is dysregulated in BMMs from old mice regardless of *M. avium* infection

**Fig. 5:**
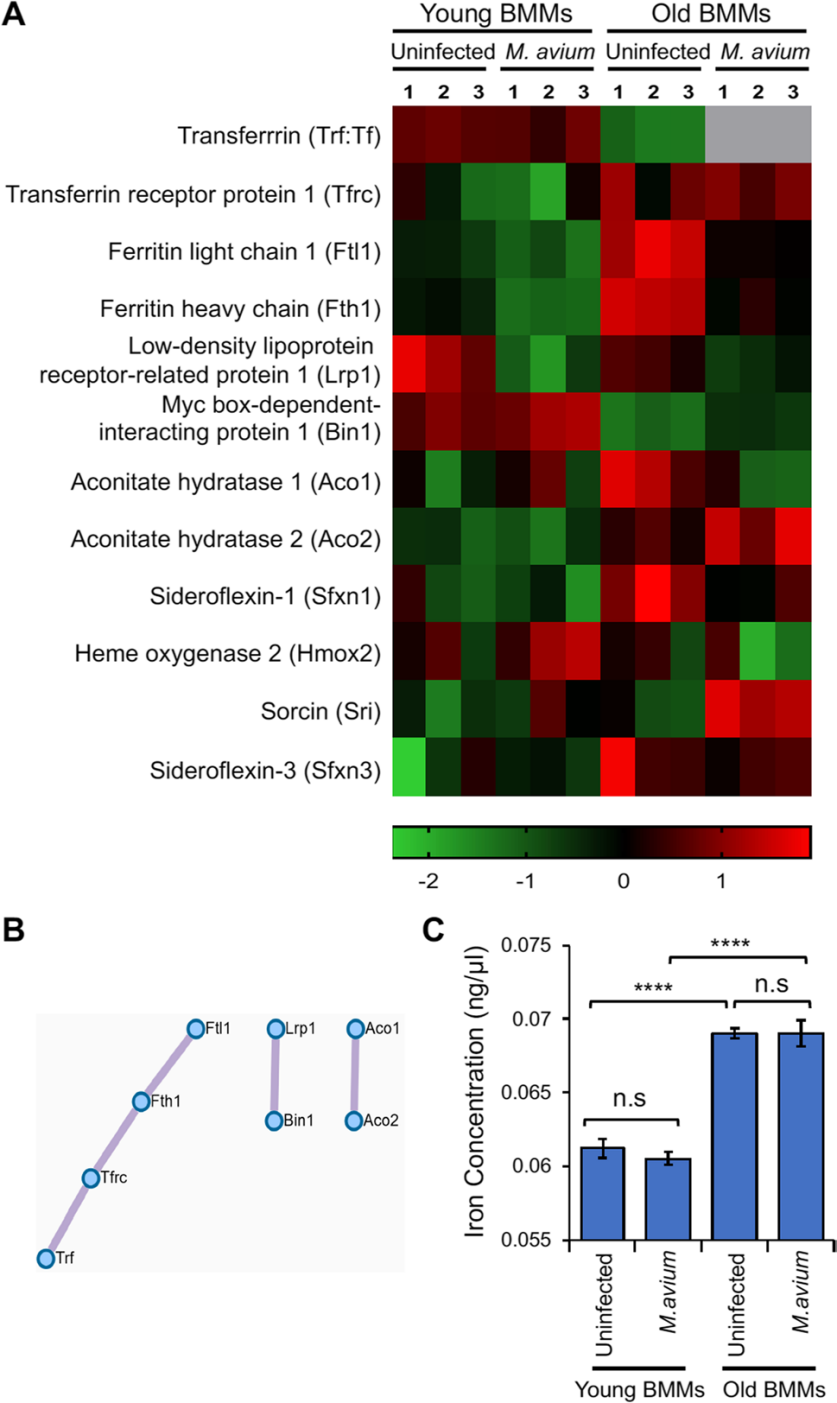
Iron homeostasis in BMMs from young and old mice with/without *M. avium* infection. **A**) Heatmap for iron homeostasis–associated proteins identified in the proteomic analysis. **B**) Metascape protein–protein Interaction network for iron homeostasis–associated proteins shown in (**A**). **C**) Intracellular iron concentration in young and old BMMs that were uninfected or infected with *M. avium* (MOI = 5) for 24 h. In (**C**), data are presented as the mean ± SD (*n* = 3) and representative of 3 independent experiments. n.s., not statistically significant; ****p < 0.0001 by one-way ANOVA, followed by Tukey’s post hoc test

Our pathway enrichment analysis shows that the intracellular iron homeostasis pathway was downregulated in uninfected BMMs isolated from old mice compared to uninfected BMMs from young mice (Fig. 2E). As shown in Fig. 3A, 75 proteins were detected in *M. avium*–infected young BMMs but not in infected old BMMs. Surprisingly, transferrin (Trf) was not detected in *M. avium*–infected old BMMs most likely due to a low protein abundance that was under our detection limit. Therefore, we hypothesized that aging likely dysregulates iron homeostasis in macrophages. As shown in Fig. 5A and Supplementary Table 3, a list of intracellular iron homeostasis–associated proteins were dysregulated in old BMMs compared to young BMMs regardless of *M. avium* infection. Interestingly, *M. avium* infection decreased the transferrin abundance in old BMMs compared to uninfected old BMMs, but not in young BMMs. Within macrophages, transferrin acts together with transferrin receptors (Tfrc) to facilitate the uptake, transport, and utilization of extracellular iron. When transferrin binds to extracellular iron, it forms a transferrin-iron complex. This complex binds to transferrin receptors on the cell surface. The cell then engulfs the transferrin-iron complex through receptor-mediated endocytosis, forming vesicles called endosomes [16, 17]. Once inside macrophages, iron can be stored within ferritin, a protein complex consisting of heavy and light chains that acts as an iron storage depot. When the body needs iron, such as during periods of increased demand, macrophages release iron by breaking down ferritin and releasing iron back into the cytoplasm, where it becomes available for various cellular processes. Different from transferrin, an increased abundance of the transferrin receptor was detected in BMMs from old mice after *M. avium* infection in comparison to BMMs from young mice (Fig. 5A). The protein abundance of the ferritin light (Ftl1) and heavy (Fth1) chain was higher in uninfected old BMMs compared to young BMMs. Similar to transferrin, *M. avium* infection decreased the abundance of ferritin light and heavy chain in old BMMs but had no effect in young BMMs. Lrp1 (low-density lipoprotein receptor–related protein 1) is a multifunctional cell surface receptor that plays a crucial role in various cellular processes, including the regulation of iron homeostasis. Lrp1 on the surface of macrophages can recognize and capture the haptoglobin-hemoglobin complex, facilitating its endocytosis and uptake by macrophages [36]. As shown in Fig. 5A and Supplementary Table 3, the level of Lrp1 in uninfected old BMMs was slightly lower than that observed in uninfected young BMMs. *M. avium* infection inhibited the expression of Lrp1 in BMMs from both old and young mice. IRP1 and IRP2 (iron regulatory proteins 1 and 2, also called Aco1 and Aco2, respectively) are two cytosolic proteins that maintain cellular iron homeostasis by regulating the expression of genes involved in iron homeostasis [37]. As seen in Fig. 5A and Supplementary Table 3, IRP2 (Aco2) expression was upregulated in old BMMs relative to young BMMs. The Metascape protein–protein network analysis shows a clear interaction between several proteins involved in iron homeostasis, e.g., Trf, Tfrc, Fth1, and Ftl1 (Fig. 5B).

We further measured the intracellular iron concentration in BMMs isolated from old and young mice that were uninfected or infected with *M. avium* in vitro in cell culture. As shown in Fig. 5C, we detected a higher level of intracellular iron concentration in old BMMs regardless of *M. avium* infection when compared to young BMMs. Unexpectedly, *M. avium* infection had no effect on intracellular iron concentration in BMMs from either old or young mice at 24 h post-infection in cell culture.

## Intracellular iron accumulation increases *M. avium* survival in BMMs from old mice

**Fig. 6:**
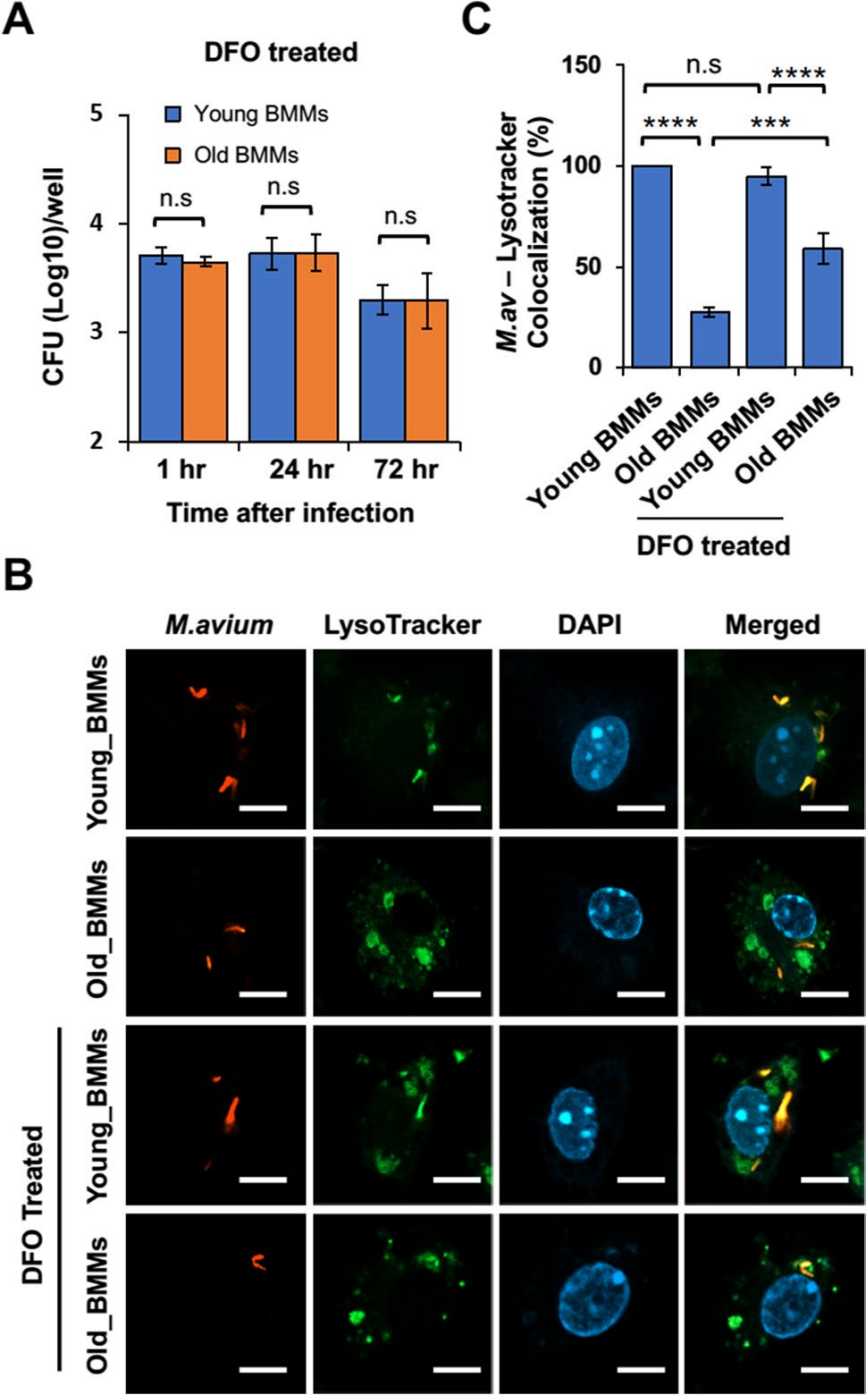
Effect of intracellular iron on *M. avium* survival in BMMs from young and old mice. **A**) *M. avium* survival assay. Young and old BMMs were infected with *M. avium* in cell culture at an MOI = 5 in the presence of DFO (deferoxamine), and then mycobacterial number within BMMs was determined at 1, 24, and 72 h post-infection. **B**) Confocal microscopy analysis for the colocalization of tdTomato-expressing *M. avium* with LysoTracker Green DND-26 at 24 h post-infection with or without DFO. **C**) Quantitation of the colocalization of tdTomato-expressing *M. avium* with LysoTracker Green DND-26 in confocal microscopy analysis. In (**A**) and (**C**), data are presented as the mean ± SD (*n* = 3). All the results are representative of 3 independent experiments. n.s., not statistically significant; ***p < 0.001 and ****p < 0.0001 by one-way ANOVA, followed by Tukey’s post hoc test

As we described above, iron is an essential nutrient for bacterial survival and replication within macrophages [16, 17]. To determine if an increased *M. avium* replication is due to upregulated iron accumulation in old mouse BMMs, we analyzed *M. avium* survival in BMMs from old and young mice in the presence of the iron chelator, deferoxamine. As seen in Fig. 6A, deferoxamine significantly rescued *M. avium* killing within old BMMs to a similar level as seen in young BMMs at 24 and 72 h post-infection. *M. avium* is an intracellular bacteria pathogen that survives within macrophages by blocking phagolysosome maturation, a process that generally destroys invading bacteria [12, 13]. This process can be measured by analyzing the colocalization of fluorescent *M. avium* and LysoTracker dye. A colocalization indicates mycobacterial degradation within macrophages. As shown in Fig. 6B and C, the phagolysosome maturation was impaired in *M. avium*–infected old BMMs when compared to infected young BMMs. Consistent with Fig. 6A, deferoxamine improved the colocalization of RFP-expressing *M. avium* with LysoTracker dye, indicating a successful phagolysosome maturation and *M. avium* degradation within old BMMs.

## Discussion

Bacterial lung infections can have severe consequences in older individuals due to decreased efficiency of immune cells, such as macrophages and neutrophils, along with impaired clearance mechanisms [7]. However, there is still little information about the mechanism(s) that causes an increased susceptibility to bacterial lung infections in older individuals. Aging alters macrophage function in old mice and older individuals, including reduced phagocytic activity (the ability to engulf and destroy pathogens), decreased production of inflammatory cytokines, and changes in their tissue-specific functions [3]. These changes can contribute to increased susceptibility to infections and impaired wound healing in older individuals. Similarly, in this study, we found that macrophage activation was dysregulated in BMMs isolated from old (25 months) mice in cell culture after *M. avium* infection by analyzing M1 and M2 macrophage markers (Fig. 1D). The whole-cell proteomic analysis further showed that a number of antibacterial pathways were upregulated in *M. avium*–infected old mouse BMMs when compared to infected young mouse BMMs **(**Fig. 3D**)**. In contrast, we found that BMMs from old mice have an attenuated antimycobacterial activity when compared to BMMs from young mice (Fig. 1A). For the first time, we found that the accumulation of intracellular iron facilitates *M. avium* intracellular survival in old BMMs as shown by the following: (1) BMMs from old mice had a much higher level of intracellular iron when compared to BMMs from young mice (Fig. 5C); (2) The iron chelator, deferoxamine, significantly rescued *M. avium* killing in old mouse BMMs to a similar level as seen in young mouse BMMS (Fig. 6A); and (3) phagolysosome maturation, a process critical for clearing invading bacteria within macrophages, was rescued by deferoxamine in *M. avium*–infected old mouse BMMs (Fig. 6B and C). Taken together, our results suggest that intracellular iron accumulation is one of the main host factors contributing to *M. avium* intracellular survival and increased host susceptibility to bacterial infection in macrophages from old mice.

It is well established that iron accumulates in various tissues in aging due to factors such as increased dietary intake over time, decreased iron excretion, and cumulative iron deposits from chronic low-grade inflammation [38–40]. It remains unknown about the physiological consequences of iron accumulation in aging tissues. However, excess iron in tissues can lead to the generation of reactive oxygen species (ROS) through the Fenton reaction. The ROS generated can in turn damage cellular components and contribute to age-related diseases such as neurodegenerative disorders, cardiovascular diseases, and cancers [38–40]. For example, excess iron in the brain has been implicated in neurodegenerative disorders like Alzheimer’s and Parkinson’s disease [41, 42]. Macrophages play a key role in iron recycling and homeostasis in tissues [36]. Therefore, age-related changes in macrophage function potentially affect iron recycling and intracellular iron homeostasis within macrophages. The latter would further influence their cellular response to invading microbial pathogens. In our current study, we show for the first time an age-related accumulation of iron in uninfected BMMs from old mice compared to uninfected BMMs from young mice (Fig. 5C). The accumulation of intracellular iron in uninfected old BMMs correlates with a dysregulated expression of a panel of iron homeostasis–associated proteins (Fig. 5A). Interestingly, *M. avium*, an intracellular bacterial pathogen that primarily infects alveolar macrophages, alters the expression profile of iron homeostasis–associated proteins in old BMMs (Fig. 5A) but does not change the intracellular iron concentration (Fig. 5C). It is unclear if *M. avium* infection induces a distinct iron homeostasis pathway for intracellular iron accumulation in BMMs from old mice, or if uninfected and *M. avium*–infected BMMs from old mice share the same mechanism for intracellular iron accumulation in cell culture. We will continue investigating these mechanisms in future studies.

Currently, there are two iron-chelating drugs, deferoxamine and deferasirox, that have been approved by FDA to treat patients with iron overload such as hereditary hemochromatosis, a genetic disorder that leads to excessive iron absorption from the diet, resulting in iron overload in various organs [43]. In the context of aging, iron chelation therapy is a topic of interest due to the potential role of iron accumulation in age-related diseases and conditions. Iron chelating drugs are being explored as a potential therapeutic strategy to manage neurodegenerative diseases and age-related diseases, such as Alzheimer’s disease [44]. Our results show for the first time that deferoxamine improved *M. avium* killing in BMMs from old mice in cell culture (Fig. 6). Considering the accumulation of iron in various tissues in older individuals and the role of macrophages in iron recycling, our study points to a potential application of iron chelator–based therapy as a novel host-directed therapy against mycobacterial lung infection in older individuals. In the future, we intend to explore the potential of combining iron chelators with antimycobacterial antibiotics, such as clarithromycin and rifampicin, as an innovative approach to combat *M. avium* lung infections in old mice.

This study intends to understand the mechanism by which aged mouse macrophages are more susceptible to *M. avium* infection in cell culture. While our results indicate the engagement of intracellular iron accumulation in aged mouse macrophages, we also realized the limitation of this study: (1) We only used BMMs from female young and old mice based on the previous findings that middle-aged to elderly women are more susceptible to *M. avium* infection [14]. In the future study, we will also use BMMs from male mice and determine if there is any sex-biased effect on *M. avium* infection in aged mouse macrophages; (2) It has been found that iron chelator, deferoxamine, also chelates zinc and copper [45]. Therefore, it is possible that deferoxamine also chelates zinc and copper in aged mouse macrophages in our study (Fig. 6). However, it is well-known that intracellular iron is required for mycobacterial survival and replication within macrophages [16–18]. In contrast, intracellular zinc and copper facilitates mycobacterial killing within macrophages [46]. Therefore, intracellular iron and zinc/copper play opposite roles in macrophages in response to mycobacterial infection. Our results in Fig. 6 show that deferoxamine improved *M. avium* killing in aged mouse macrophages, indicating the likelihood that intracellular iron accumulation plays a major role in increasing host susceptibility to *M. avium* infection in vitro. In the future study, we will further investigate the cellular pathways responsible for intracellular iron accumulation using genetical tools to determine the role of intracellular iron in *M. avium* infection in aged mouse macrophages.

In conclusion, our study demonstrates for the first time that intracellular iron accumulation within host cells facilitates non-tuberculous mycobacterial survival within macrophages from old mice, potentially responsible for the disease progression. Our research has reaffirmed the importance of iron accumulation in age-associated diseases and has for the first time provided valuable insights into the interaction of intracellular iron homeostasis and macrophage function in aging. These findings have implications not only for non-tuberculous mycobacterial lung infection in older individuals but also for the age-related decline in host immunity in response to other microbial lung infections. As we move forward, it is crucial to understand the mechanisms that are responsible for iron accumulation in aging macrophages and how dysregulated intracellular iron homeostasis affects macrophage function in aging. Overall, this study contributes to our understanding of age-associated immunosenescence and highlights the need for further investigation in this area.

### Supplementary Information

Below is the link to the electronic supplementary material.

Supplementary file1 (DOCX 43 KB)

Supplementary file2 (DOCX 34 KB)

Supplementary file3 (DOCX 23 KB)

Supplementary file4 (DOCX 628 KB)

Supplementary file5 (DOCX 75 KB)

Supplementary file6 (DOCX 30 KB)

Supplementary file7 (DOCX 30 KB)

Supplementary file8 (DOCX 616 KB)

Supplementary file9 (DOCX 36 KB)
