# Supplementary material for: Intracellular iron accumulation facilitates mycobacterial infection in old mouse macrophages
Source: GeroScience. 2023 Dec 30;46(2):2739–54. doi: 10.1007/s11357-023-01048-1 (PMC10828278; doi:10.1007/s11357-023-01048-1)
Supplement: Supplementary file 1 — Supplementary file1 (DOCX 43 KB) [file 11357_2023_1048_MOESM1_ESM.docx]

**Supplementary Table 1A: Proteins Unique in Uninfected Old BMMs *vs* Uninfected Young BMMs**

| **Protein IDs** | **Protein names** | **Gene names** | **Uninfected Old BMMs** | | | **Uninfected Young BMMs** | | |
| --- | --- | --- | --- | --- | --- | --- | --- | --- |
|  |  |  | **LFQ intensity 1967_br1_tr1** | **LFQ intensity 1967_br1_tr2** | **LFQ intensity 1967_br1_tr3** | **LFQ intensity 1967_br3_tr1** | **LFQ intensity 1967_br3_tr2** | **LFQ intensity 1967_br3_tr3** |
| Q9QZE7;A0A1D5RLR0 | Translin-associated protein X | Tsnax | 270620000 | 312230000 | 0 | 0 | 0 | 0 |
| E9PVZ8;E9QAH1;Q9CTU9 | Golgi autoantigen, golgin subfamily b, macrogolgin 1 | Golgb1 | 211960000 | 254140000 | 0 | 0 | 0 | 0 |
| Q91VR8 | Protein BRICK1 | Brk1 | 68083000 | 0 | 64621000 | 56552000 | 0 | 0 |
| P56382 | ATP synthase subunit epsilon, mitochondrial | Atp5e | 35263000 | 33008000 | 0 | 0 | 16717000 | 0 |
| P97825 | Hematological and neurological expressed 1 protein;Hematological and neurological expressed 1 protein, N-terminally processed | Hn1 | 31390000 | 30177000 | 30943000 | 0 | 27858000 | 0 |
| Q91WK5 | Glycine cleavage system H protein, mitochondrial | Gcsh | 28729000 | 33422000 | 32088000 | 0 | 0 | 0 |
| Q61017 | Guanine nucleotide-binding protein G(I)/G(S)/G(O) subunit gamma-T2 | Gngt2 | 28625000 | 20112000 | 27536000 | 0 | 0 | 0 |
| Q923L7 | Ear6 protein | Ear6 | 25001000 | 25879000 | 32841000 | 0 | 0 | 0 |
| Q9D358;A0A1W2P7X3;Q561M1 | Low molecular weight phosphotyrosine protein phosphatase | Acp1 | 20437000 | 18397000 | 21391000 | 18724000 | 0 | 0 |
| Q8BGH7 | CDC42 small effector protein 2 | Cdc42se2 | 16989000 | 12177000 | 15265000 | 0 | 0 | 0 |
| Q8K273 | Membrane magnesium transporter 1 | Mmgt1 | 15269000 | 15415000 | 0 | 0 | 0 | 0 |
| G5E895;D3Z494;S4R2G9 | Aldo-keto reductase family 1, member B10 (aldose reductase) | Akr1b10 | 14630000 | 15572000 | 14113000 | 9503000 | 0 | 0 |
| A2A5R2;F6YCJ0;D3YYK9;G3X9K3 | Brefeldin A-inhibited guanine nucleotide-exchange protein 2 | Arfgef2 | 14153000 | 10208000 | 13441000 | 0 | 8195000 | 0 |
| Q921L6;Q60598 | Src substrate cortactin | Cttn | 13962000 | 9698600 | 0 | 7909800 | 0 | 0 |
| Q5SV41;P53808 | Phosphatidylcholine transfer protein | Pctp | 13960000 | 0 | 10183000 | 15046000 | 0 | 0 |
| Q3U2S8;D3Z019;D3YZ46 | Voltage-gated hydrogen channel 1 | Hvcn1 | 13906000 | 10034000 | 14040000 | 0 | 20306000 | 0 |
| P56387 | Dynein light chain Tctex-type 3 | Dynlt3 | 13160000 | 10366000 | 11076000 | 0 | 0 | 0 |
| O35942;F6TQF4;F6THK7;B1AUW8;B1AUW9;Q8BR10;B1AUW6;Q62406 | Serine/threonine-protein kinase Nek2;Interleukin-1 receptor-associated kinase 1 | Nek2;Irak1 | 12755000 | 12348000 | 0 | 0 | 0 | 0 |
| O09047 | C3a anaphylatoxin chemotactic receptor | C3ar1 | 12511000 | 0 | 11544000 | 11923000 | 0 | 0 |
| Q9CXT8;D3Z1T9 | Mitochondrial-processing peptidase subunit beta | Pmpcb | 11972000 | 15304000 | 12211000 | 0 | 10924000 | 0 |
| E9QNQ2;B2RXR6;J3QK13;J3QK37;J3QNJ4 | Serine/threonine-protein phosphatase 6 regulatory ankyrin repeat subunit B | Ankrd44 | 11614000 | 13231000 | 0 | 0 | 0 | 6177200 |
| Q99K23;A0A1B0GSM1;A0A1B0GSK1 | Ufm1-specific protease 2 | Ufsp2 | 11513000 | 0 | 11922000 | 0 | 12654000 | 0 |
| Q99J56 | Derlin-1 | Derl1 | 11481000 | 11635000 | 11593000 | 0 | 0 | 10170000 |
| Q9CR68 | Cytochrome b-c1 complex subunit Rieske, mitochondrial;Cytochrome b-c1 complex subunit 11 | Uqcrfs1 | 11047000 | 11255000 | 13291000 | 0 | 10188000 | 0 |
| Q8CGA0;A0A338P7F2 | Protein phosphatase 1F | Ppm1f | 10583000 | 0 | 6566400 | 0 | 0 | 0 |
| Q3UMA3;Q99LI8;B1ATY9;F6VV02 | Hepatocyte growth factor-regulated tyrosine kinase substrate | Hgs | 9781400 | 8901300 | 9788800 | 0 | 0 | 9634900 |
| Q8JZN5;A0A0G2JDY4;A0A0G2JF25 | Acyl-CoA dehydrogenase family member 9, mitochondrial | Acad9 | 9640600 | 9755400 | 10132000 | 0 | 7702800 | 0 |
| Q63810 | Calcineurin subunit B type 1 | Ppp3r1 | 9551800 | 9894200 | 9413800 | 0 | 10318000 | 0 |
| Q91VK1 | Basic leucine zipper and W2 domain-containing protein 2 | Bzw2 | 9520600 | 9655000 | 8573000 | 0 | 12027000 | 0 |
| O89051;A0A2I3BQ30;A0A2I3BPW7 | Integral membrane protein 2B;BRI2, membrane form;BRI2 intracellular domain;BRI2C, soluble form;Bri23 peptide | Itm2b | 9390500 | 8737700 | 0 | 0 | 0 | 0 |
| Q91ZA3;D3YWM4;H3BL62 | Propionyl-CoA carboxylase alpha chain, mitochondrial | Pcca | 9320200 | 8849300 | 0 | 0 | 9303700 | 0 |
| Q9ERL7;D3Z2F6 | Glia maturation factor gamma | Gmfg | 9136300 | 8803400 | 6946900 | 0 | 0 | 0 |
| Q9JHS9;A0A1L1SVD8 | Spliceosome-associated protein CWC15 homolog | Cwc15 | 9111900 | 0 | 8782900 | 0 | 7595800 | 0 |
| Q8R1G6;A0A087WPL1;F7C957;E9Q996 | PDZ and LIM domain protein 2 | Pdlim2 | 8900100 | 7815900 | 9662400 | 0 | 0 | 0 |
| P70122;F6TN03 | Ribosome maturation protein SBDS | Sbds | 8869100 | 8640700 | 0 | 0 | 6177500 | 0 |
| A0A1D5RL86;P61514 | 60S ribosomal protein L37a | Rpl37a | 8684100 | 9671100 | 10210000 | 0 | 0 | 0 |
| Q9D8N2;Q3TH34;D6RIQ6;D3YUG1 | Protein FAM45A | Fam45a | 8638700 | 9162400 | 11111000 | 0 | 0 | 8195500 |
| D3YZ09;Q9CY66 | H/ACA ribonucleoprotein complex subunit 1 | Gar1 | 8470000 | 6436300 | 6145800 | 0 | 0 | 6488300 |
| Q80UU9 | Membrane-associated progesterone receptor component 2 | Pgrmc2 | 8322500 | 8862700 | 8425100 | 0 | 0 | 0 |
| Q9DBL1;E9Q5L3;A0A140LHL4 | Short/branched chain specific acyl-CoA dehydrogenase, mitochondrial | Acadsb | 8292500 | 9610400 | 0 | 9443300 | 0 | 0 |
| Q99KU0;Q5SXR2 | Vacuole membrane protein 1 | Vmp1 | 8198800 | 7479900 | 6812600 | 6731200 | 0 | 0 |
| A0A1Y7VLJ5;A0A0R4J022;Q8BNQ3 | Integral membrane protein GPR137B | Gpr137b | 7920300 | 0 | 5973300 | 8366400 | 0 | 0 |
| O35682;A0A0N4SW94 | Myeloid-associated differentiation marker | Myadm | 7880900 | 6707900 | 7747500 | 0 | 0 | 9272500 |
| P30993 | C5a anaphylatoxin chemotactic receptor 1 | C5ar1 | 7738400 | 7554200 | 2862000 | 6479600 | 0 | 0 |
| P45591 | Cofilin-2 | Cfl2 | 7688200 | 7888500 | 7512200 | 0 | 6556100 | 0 |
| Q6NSR8;F6T2H5 | Probable aminopeptidase NPEPL1 | Npepl1 | 7585300 | 8994200 | 8914500 | 0 | 0 | 7970200 |
| Q91V04 | Translocating chain-associated membrane protein 1 | Tram1 | 7551300 | 0 | 13922000 | 0 | 0 | 0 |
| B1ASZ3;Q64516;Q8C635;Q9WU65 | Glycerol kinase | Gyk;Gk | 7515900 | 9447700 | 7611700 | 0 | 12174000 | 0 |
| Q6NZB0;A2ALF0;A2ALF3;F6TQL3;F7CXJ2;F6QIL6;D3Z4I5 | DnaJ homolog subfamily C member 8 | Dnajc8 | 7426800 | 7100200 | 8233300 | 7312200 | 0 | 0 |
| Q571E4;Q8CC47 | N-acetylgalactosamine-6-sulfatase | Galns | 7389100 | 5814000 | 6425900 | 0 | 0 | 3529800 |
| Q8R164 | Valacyclovir hydrolase | Bphl | 7275400 | 8038800 | 8299500 | 0 | 0 | 0 |
| F6TCV0;D3YX27;Q9JIY5;D3YX28;F6XUR8;S4R1B3;S4R1A8;A2RT60 | Serine protease HTRA2, mitochondrial | Htra2 | 7142000 | 8433700 | 8662100 | 0 | 8419000 | 0 |
| O35459;F7B227 | Delta(3,5)-Delta(2,4)-dienoyl-CoA isomerase, mitochondrial | Ech1 | 7107700 | 8597600 | 6365900 | 6846000 | 0 | 0 |
| A0A1Y7VN19;Q9CXR1 | Dehydrogenase/reductase SDR family member 7 | Dhrs7 | 7072800 | 5310700 | 0 | 5070100 | 0 | 0 |
| J3QJX3;Q9Z2G6 | Protein sel-1 homolog 1 | Sel1l | 7043100 | 0 | 7841300 | 6210600 | 0 | 0 |
| Q8BH69 | Selenide, water dikinase 1 | Sephs1 | 6681700 | 0 | 6253200 | 0 | 0 | 0 |
| Q9R0M6;A2AFP4;A2AFP5 | Ras-related protein Rab-9A | Rab9a | 6556500 | 6031100 | 5682800 | 0 | 0 | 0 |
| E9PVP3;Q5F2B1;F6XX36;Q8R0J2;Q9R0Q9;F6ZGG4 | Mannose-P-dolichol utilization defect 1 protein | Mpdu1 | 6555900 | 6411800 | 6474000 | 0 | 0 | 5501500 |
| Q80VA0 | N-acetylgalactosaminyltransferase 7 | Galnt7 | 6535000 | 7491900 | 6915800 | 0 | 0 | 0 |
| Q80TM9;F6ZL69;F6YR29 | Nischarin | Nisch | 6363600 | 0 | 7259800 | 5679600 | 0 | 0 |
| Q8BJ71;A0A1D5RLQ0;A0A1D5RM86 | Nuclear pore complex protein Nup93 | Nup93 | 6300300 | 0 | 7626500 | 0 | 0 | 0 |
| P51885 | Lumican | Lum | 6079200 | 6490600 | 0 | 0 | 0 | 7624500 |
| A0A2R8VKS6;Q9D1C8 | Vacuolar protein sorting-associated protein 28 homolog | Vps28 | 6039400 | 0 | 5693500 | 0 | 0 | 0 |
| Q9ERS2 | NADH dehydrogenase [ubiquinone] 1 alpha subcomplex subunit 13 | Ndufa13 | 6034000 | 0 | 5915300 | 0 | 0 | 5107900 |
| P14576;E9PXC0;A0A1W2P809;A0A1Y7VJJ0 | Signal recognition particle 54 kDa protein | Srp54;Srp54c | 6012100 | 6230200 | 4866800 | 0 | 4244600 | 0 |
| Q99LE1 | RILP-like protein 2 | Rilpl2 | 5884500 | 0 | 5880200 | 0 | 0 | 0 |
| Q91YJ2 | Sorting nexin-4 | Snx4 | 5848700 | 6822500 | 6355400 | 7523300 | 0 | 0 |
| Q3TIR6;P61759 | Prefoldin subunit 3 | Vbp1 | 5838400 | 6149500 | 7335300 | 0 | 0 | 6722400 |
| Q8K1R7 | Serine/threonine-protein kinase Nek9 | Nek9 | 5649900 | 6232600 | 6259400 | 7443900 | 0 | 0 |
| Q5SUR0 | Phosphoribosylformylglycinamidine synthase | Pfas | 5505300 | 0 | 4966800 | 0 | 0 | 0 |
| Q8BPX9 | Solute carrier family 15 member 3 | Slc15a3 | 5308800 | 3874600 | 6486900 | 4173500 | 0 | 0 |
| Q8BLN5;F7BJL0 | Lanosterol synthase | Lss | 5270600 | 2931200 | 0 | 0 | 3480300 | 0 |
| P11152 | Lipoprotein lipase | Lpl | 5071100 | 0 | 5794600 | 0 | 0 | 0 |
| Q91ZN5;A0A286YE16;Q9D1L5 | Adenosine 3-phospho 5-phosphosulfate transporter 1 | Slc35b2 | 5048200 | 4448400 | 4515600 | 0 | 0 | 0 |
| Q8R3V5;A2AWI7;A2AWI9 | Endophilin-B2 | Sh3glb2 | 5025100 | 4618000 | 4773200 | 0 | 0 | 0 |
| Q5FWX7;E9Q6C1;F6UHR6;E9PXZ2;A0A0A6YXH3;A2A8Z1 | Oxysterol-binding protein;Oxysterol-binding protein-related protein 9 | Osbpl9 | 4978600 | 4843600 | 0 | 0 | 0 | 0 |
| F6ZFT1;F8WJ64;Q9CR21 | Acyl carrier protein;Acyl carrier protein, mitochondrial | Ndufab1 | 4896900 | 0 | 4692800 | 0 | 0 | 0 |
| Q6PF96;Q921G7 | Electron transfer flavoprotein-ubiquinone oxidoreductase, mitochondrial | Etfdh | 4815500 | 5123600 | 4533700 | 0 | 3648200 | 0 |
| Q9ESW4 | Acylglycerol kinase, mitochondrial | Agk | 4718600 | 3987300 | 5036300 | 0 | 0 | 0 |
| Q99LD9;D3Z487;D3Z7J6 | Translation initiation factor eIF-2B subunit beta | Eif2b2 | 4645100 | 4815300 | 5403500 | 0 | 0 | 0 |
| E0CYJ0;E0CXA9;Q6PEB6;G8JL35 | MOB-like protein phocein | Mob4 | 4562500 | 4604300 | 5756400 | 0 | 0 | 0 |
| Q9D1K7;F8WIU1;H7BXB9 | UPF0687 protein C20orf27 homolog | 1700037H04Rik | 4560700 | 0 | 4081400 | 0 | 4665000 | 0 |
| Q9CPN8;A6X8Z3;Q5SF07 | Insulin-like growth factor 2 mRNA-binding protein 3 | Igf2bp3 | 4558900 | 3539700 | 3062400 | 0 | 0 | 0 |
| A0A0U1RQ06;P62743 | AP-2 complex subunit sigma | Ap2s1 | 4393500 | 3663600 | 3684000 | 0 | 0 | 4566100 |
| Q8K0D5 | Elongation factor G, mitochondrial | Gfm1 | 4389500 | 5252400 | 5492200 | 0 | 4941100 | 0 |
| A2BGI8;Q9D868;A2BGI9 | Peptidyl-prolyl cis-trans isomerase;Peptidyl-prolyl cis-trans isomerase H | Ppih | 4359200 | 0 | 4052800 | 4202800 | 0 | 0 |
| Q9Z1K5;H7BWY7;H9KV21 | E3 ubiquitin-protein ligase ARIH1 | Arih1 | 4309400 | 5671100 | 5032400 | 0 | 0 | 0 |
| A0A494BAQ2;A0A494B9Y5;Q9WTK5 | Nuclear factor NF-kappa-B p100 subunit;Nuclear factor NF-kappa-B p52 subunit | Nfkb2 | 4088700 | 4190400 | 3782200 | 0 | 3639300 | 0 |
| Q3UVG3 | Protein FAM91A1 | Fam91a1 | 3988700 | 5011200 | 4716000 | 0 | 0 | 0 |
| A0A1L1SVC7;Q9CQ02;A0A1L1SRX4;A0A1L1SRM6;A0A1L1ST08;A0A1L1SRJ9 | COMM domain-containing protein 4 | Commd4 | 3978200 | 0 | 5851500 | 0 | 0 | 5402100 |
| E9PYN1;Q8R5M8;A0A668KLV1;E0CY16 | Cell adhesion molecule 1 | Cadm1 | 3922500 | 4624700 | 0 | 0 | 0 | 0 |
| H3BIX0;H3BL37;O08784;F6R3V4;H3BK88 | Treacle protein | Tcof1 | 3901800 | 4065600 | 0 | 0 | 0 | 3967700 |
| A2A5K2;P55065;A2A5K3;A2A5K4 | Phospholipid transfer protein | Pltp | 3769900 | 0 | 3933000 | 0 | 0 | 4614100 |
| Q64735;A0A0A6YVT1;A0A0A6YXN9;A0A0A6YY75;A0A0A6YXR6 | Complement component receptor 1-like protein | Cr1l | 3651500 | 3382200 | 3407500 | 0 | 0 | 3040300 |
| Q8C0L6;A0A1B0GRJ2;Q3TXR6 | Peroxisomal N(1)-acetyl-spermine/spermidine oxidase | Paox | 3612000 | 4804300 | 0 | 0 | 0 | 0 |
| Q6IFX2 | Keratin, type I cytoskeletal 42 | Krt42 | 3587700 | 0 | 3869200 | 0 | 0 | 0 |
| Q3UUQ7 | GPI inositol-deacylase | Pgap1 | 3439700 | 0 | 3503300 | 4398400 | 0 | 0 |
| Q91WT8;D3YXZ5;D3Z113 | RNA-binding protein 47 | Rbm47 | 3422400 | 0 | 3566100 | 0 | 0 | 0 |
| Q9D1H8 | 39S ribosomal protein L53, mitochondrial | Mrpl53 | 3400000 | 3792800 | 3524000 | 0 | 0 | 0 |
| D3Z3C3;Q6YGZ1 | Heparanase;Heparanase 8 kDa subunit;Heparanase 50 kDa subunit | Hpse | 3366100 | 0 | 5477900 | 0 | 0 | 3797400 |
| D3Z3M7;D3Z2Z1;F8WIA1;Q922J3;F6RCU2;Q8C0S5;F6RAY2;F7CB97 | CAP-Gly domain-containing linker protein 1 | Clip1 | 3073700 | 3018900 | 0 | 0 | 0 | 0 |
| A2A9I0;O35166 | Golgi SNAP receptor complex member 2 | Gosr2 | 3005400 | 2725900 | 2683900 | 0 | 2622800 | 0 |
| Q80YR5;F6WIZ2 | Scaffold attachment factor B2 | Safb2 | 2875000 | 2632000 | 2625100 | 0 | 0 | 0 |
| Q8CBE3;H3BK66;Q05BF4;H3BJR7;H3BJS1 | WD repeat-containing protein 37 | Wdr37 | 2702400 | 2741900 | 1955200 | 0 | 0 | 0 |
| B9EKI5;Q3TTA7 | E3 ubiquitin-protein ligase CBL-B | Cblb | 2482000 | 1664700 | 1940300 | 0 | 1091800 | 0 |
| Q80VJ3 | 2-deoxynucleoside 5-phosphate N-hydrolase 1 | Dnph1 | 2464700 | 0 | 3505400 | 0 | 0 | 2729300 |
| Q9CQA1 | Trafficking protein particle complex subunit 5 | Trappc5 | 2406800 | 2829900 | 2937000 | 0 | 2263800 | 0 |
| Q80WT5;H3BIZ9;H3BKM5;H3BJH7 | Aftiphilin | Aftph | 2232500 | 2184900 | 2693200 | 0 | 0 | 0 |
| Q3UMT1;F6XWD4 | Protein phosphatase 1 regulatory subunit 12C | Ppp1r12c | 2154800 | 1990800 | 2287700 | 0 | 0 | 2170100 |
| Q8BVU5 | ADP-ribose pyrophosphatase, mitochondrial | Nudt9 | 1987900 | 1920300 | 1754000 | 0 | 0 | 0 |
| Q3T9A5;Q8BZM1;D6RGR3;D3Z0L3 | Glomulin | Glmn | 1213700 | 1060600 | 0 | 0 | 0 | 0 |
| A0A0R4J0Q6;Q80VI1 | E3 ubiquitin-protein ligase TRIM56 | Trim56 | 1034800 | 974380 | 1102400 | 0 | 0 | 995430 |
| A0A1W2P855;A0A1W2P7Z8;A0A1W2P7S8;A0A1W2P7H4;A0A1W2P6Q8;Q6A068 | Cell division cycle 5-like protein | Cdc5l | 911970 | 649600 | 0 | 0 | 0 | 0 |
| Q8C4Q6 | Axin interactor, dorsalization-associated protein | Aida | 788080 | 1035200 | 1889300 | 0 | 2697100 | 0 |
| A0A087WRH2;D3Z3C1;Q9WUD8;J3QPY3 | Fas apoptotic inhibitory molecule 1 | Faim;Gm6432 | 0 | 4348100 | 4162500 | 0 | 0 | 0 |
| A0A2R8VHH1;Q99P88 | Nuclear pore complex protein Nup155 | Nup155 | 0 | 9680200 | 8784800 | 11463000 | 0 | 0 |
| A0A2R8VHP3 | Predicted pseudogene 5478 | Gm5478 | 0 | 10092000 | 9476500 | 0 | 0 | 6791000 |
| D3Z079;Q8K400;D3Z2Q2;F6WXQ4 | Syntaxin-binding protein 5 | Stxbp5 | 0 | 5099800 | 6328100 | 0 | 4801400 | 0 |
| F7CTF8;E0CYI3;E0CYB9;Q6PCP5;F7B1A4;F6VAL0 | Mitochondrial fission factor | Mff | 0 | 6727200 | 5850300 | 0 | 0 | 0 |
| E9PV12;Q9D5V5;G3X914;E9Q6Z0 | Cullin-5 | Cul5 | 0 | 11366000 | 10773000 | 0 | 5253600 | 0 |
| E9Q9Q2;A0A087WP65;A0A087WPF0;Q80ZH9;A0A087WQI5 | R3H domain containing 1 | R3hdm1 | 0 | 4558100 | 5432800 | 0 | 0 | 0 |
| F8WJG3;P62996 | Transformer-2 protein homolog beta | Tra2b | 0 | 9788100 | 6077400 | 5040300 | 0 | 0 |
| P17665 | Cytochrome c oxidase subunit 7C, mitochondrial | Cox7c | 0 | 12575000 | 15648000 | 0 | 0 | 0 |
| P18654;B1AXN9 | Ribosomal protein S6 kinase alpha-3 | Rps6ka3 | 0 | 6855000 | 6591600 | 0 | 0 | 8072000 |
| Q3UHD6;A0A0G2JF85;A0A0G2JG07;A0A0G2JGD5 | Sorting nexin-27 | Snx27 | 0 | 6758900 | 9663700 | 6957700 | 0 | 0 |
| Q6A0A2;A0A087WPU9 | La-related protein 4B | Larp4b | 0 | 4684600 | 4550100 | 0 | 0 | 0 |
| Q8BTV2;A0A494B9T0 | Cleavage and polyadenylation specificity factor subunit 7 | Cpsf7 | 0 | 2222200 | 2259700 | 0 | 0 | 0 |
| Q8CFI7 | DNA-directed RNA polymerase II subunit RPB2 | Polr2b | 0 | 222770000 | 166210000 | 0 | 0 | 0 |
| Q923D4 | Splicing factor 3B subunit 5 | Sf3b5 | 0 | 9722700 | 7558400 | 0 | 9360700 | 0 |
| Q99K28 | ADP-ribosylation factor GTPase-activating protein 2 | Arfgap2 | 0 | 5022000 | 4320400 | 0 | 0 | 0 |
| Q99PG2 | Opioid growth factor receptor | Ogfr | 0 | 2107200 | 2947100 | 1960900 | 0 | 0 |
| Q9CQA3 | Succinate dehydrogenase [ubiquinone] iron-sulfur subunit, mitochondrial | Sdhb | 0 | 3640700 | 3873700 | 0 | 0 | 4256400 |
| Q9CQV6;M0QWC2;Q91VR7 | Microtubule-associated proteins 1A/1B light chain 3B;Microtubule-associated proteins 1A/1B light chain 3A | Map1lc3b;Map1lc3a | 0 | 29141000 | 26083000 | 0 | 0 | 0 |
| Q9DAR7;Q3TBW9;D6RFQ0 | m7GpppX diphosphatase | Dcps | 0 | 4950200 | 3408800 | 0 | 0 | 4310300 |
| Q9EPK2 | Protein XRP2 | Rp2 | 0 | 3595700 | 3992700 | 3987700 | 0 | 0 |
| Q9JHK4;A0A2K6EDL0 | Geranylgeranyl transferase type-2 subunit alpha | Rabggta | 0 | 4162800 | 4435800 | 0 | 0 | 0 |
