# Supplementary material for: Intracellular iron accumulation facilitates mycobacterial infection in old mouse macrophages
Source: GeroScience. 2023 Dec 30;46(2):2739–54. doi: 10.1007/s11357-023-01048-1 (PMC10828278; doi:10.1007/s11357-023-01048-1)
Supplement: Supplementary file 2 — Supplementary file2 (DOCX 34 KB) [file 11357_2023_1048_MOESM2_ESM.docx]

**Supplementary Table 1B: Proteins Unique in Uninfected Young BMMs *vs* Uninfected Old BMMs**

| **Protein IDs** | **Protein names** | **Gene names** | **Uninfected Old BMMs** | | | **Uninfected Young BMMs** | | |
| --- | --- | --- | --- | --- | --- | --- | --- | --- |
|  |  |  | **LFQ intensity 1967_br1_tr1** | **LFQ intensity 1967_br1_tr2** | **LFQ intensity 1967_br1_tr3** | **LFQ intensity 1967_br3_tr1** | **LFQ intensity 1967_br3_tr2** | **LFQ intensity 1967_br3_tr3** |
| P62892 | 60S ribosomal protein L39 | Rpl39 | 5555100 | 0 | 0 | 71363000 | 69990000 | 66721000 |
| A0A0R4J1K4;A0A0R4J1K9;A0A0R4J101;P13808 | Anion exchange protein 2 | Slc4a2 | 57379000 | 0 | 0 | 66374000 | 59055000 | 0 |
| D3YU60;E9QJW0;Q91VS7 | Microsomal glutathione S-transferase 1 | Mgst1 | 0 | 18042000 | 0 | 34432000 | 31451000 | 31382000 |
| A0A3B2W7N5;F6U1P7;B2RWC4 | Leucine rich repeat containing 73 | Lrrc73 | 0 | 0 | 20791000 | 34034000 | 22291000 | 14942000 |
| P62141;A0A0J9YUU8;A0A0J9YUG2 | Serine/threonine-protein phosphatase PP1-beta catalytic subunit | Ppp1cb | 0 | 0 | 0 | 15235000 | 15746000 | 16049000 |
| Q6PGC1;Q8BN72 | ATP-dependent RNA helicase Dhx29 | Dhx29 | 12914000 | 0 | 0 | 14566000 | 14310000 | 0 |
| E9Q9H2;P54103;E9Q9H3;Q3TRX6 | DnaJ homolog subfamily C member 2;DnaJ homolog subfamily C member 2, N-terminally processed | Dnajc2 | 0 | 0 | 0 | 14389000 | 15793000 | 13671000 |
| O55042 | Alpha-synuclein | Snca | 0 | 0 | 0 | 12446000 | 0 | 10940000 |
| A0A3Q4EH04;A0A3Q4L335;A0A3Q4L393;Q8BL97 | Serine/arginine-rich splicing factor 7 | Srsf7 | 0 | 6829100 | 0 | 11961000 | 11235000 | 10720000 |
| Q8R550;B0R0Y8 | SH3 domain-containing kinase-binding protein 1 | Sh3kbp1 | 0 | 0 | 11317000 | 11887000 | 11255000 | 10925000 |
| Q6ZWY3;D6RH49;D3YYB0 | 40S ribosomal protein S27-like;40S ribosomal protein S27 | Rps27l | 0 | 0 | 0 | 11054000 | 11223000 | 11746000 |
| A0A1B0GT75;A0A1B0GR63;A0A1B0GSH4 | Charged multivesicular body protein 2A | Chmp2a | 0 | 0 | 2281400 | 10401000 | 12610000 | 0 |
| P21995 | Embigin | Emb | 0 | 0 | 0 | 9942300 | 10967000 | 11626000 |
| Q8BUM3 | Tyrosine-protein phosphatase non-receptor type 7 | Ptpn7 | 0 | 0 | 7652000 | 9122000 | 0 | 10241000 |
| A0A2R8W6X5;Q9R060 | Cytosolic Fe-S cluster assembly factor NUBP1 | Nubp1 | 0 | 0 | 0 | 8935800 | 0 | 4593500 |
| Q80VQ0;J3QMK6;E9Q3E1;F6QPV9 | Aldehyde dehydrogenase family 3 member B1 | Aldh3b1 | 8638500 | 0 | 0 | 8931400 | 9009500 | 8690300 |
| Q3V3R1 | Monofunctional C1-tetrahydrofolate synthase, mitochondrial | Mthfd1l | 0 | 0 | 8115400 | 8717100 | 7796700 | 7457500 |
| Q91ZR1 | Ras-related protein Rab-4B | Rab4b | 0 | 0 | 8129400 | 8681500 | 8654400 | 8248600 |
| E9PW20;Q9CY57;D3YZA1;D3Z7T7 | Chromatin target of PRMT1 protein | Chtop | 0 | 0 | 4764600 | 8463100 | 10109000 | 0 |
| A8Y5E9;B2KGA7;Q505B7 | Protein archease | Zbtb8os | 0 | 0 | 0 | 8377000 | 0 | 6761100 |
| Q3UDF0;A2AR27 | Solute carrier family 2, facilitated glucose transporter member 6 | Slc2a6 | 0 | 0 | 0 | 7958200 | 6105900 | 7429500 |
| P31324;H3BK84 | cAMP-dependent protein kinase type II-beta regulatory subunit | Prkar2b | 0 | 0 | 0 | 7727100 | 0 | 9734200 |
| O88845 | A-kinase anchor protein 10, mitochondrial | Akap10 | 0 | 0 | 0 | 7606100 | 9473600 | 0 |
| A0A0B4J1G1;E9Q415;A0A0B4J1E6;P08101 | Low affinity immunoglobulin gamma Fc region receptor II | Fcgr2b;Fcgr2 | 0 | 0 | 0 | 7465700 | 6739600 | 7881100 |
| Q8BYA0;F6UI15;B1ATU0;Q9D2H8 | Tubulin-specific chaperone D | Tbcd | 0 | 7206600 | 0 | 7359300 | 6907400 | 6950100 |
| O35900;O35901 | U6 snRNA-associated Sm-like protein LSm2 | Lsm2 | 0 | 0 | 0 | 7328400 | 7791800 | 8313700 |
| Q62086 | Serum paraoxonase/arylesterase 2 | Pon2 | 0 | 5894100 | 0 | 7239400 | 7051900 | 0 |
| O08797 | SPI6 | Serpinb9 | 0 | 0 | 0 | 7210100 | 6222200 | 0 |
| P61967;D3Z268;D3Z0D6;D3YXN0;B1B0F6;Q8BW87 | AP-1 complex subunit sigma-1A | Ap1s1 | 0 | 5777400 | 0 | 7073800 | 0 | 7929400 |
| Q9CWW6 | Peptidyl-prolyl cis-trans isomerase NIMA-interacting 4 | Pin4 | 0 | 0 | 7559900 | 7071600 | 7131700 | 0 |
| D3YVK1;P15105 | Glutamine synthetase | Glul | 0 | 0 | 0 | 7040900 | 9682000 | 8794600 |
| Q8CCH2 | NHL repeat-containing protein 3 | Nhlrc3 | 0 | 5649000 | 0 | 6957900 | 5093500 | 5945300 |
| O70152;A2BDX2;F8WII3 | Dolichol-phosphate mannosyltransferase subunit 1 | Dpm1 | 6365000 | 0 | 0 | 6872500 | 6450400 | 9239100 |
| Q8BQ47 | Protein canopy homolog 4 | Cnpy4 | 0 | 0 | 0 | 6803200 | 4914600 | 0 |
| Q3ULW8;A0A5H1ZRP0;A0A5H1ZRL8;A0A5H1ZRL5 | | Parp3 | 0 | 0 | 0 | 6782800 | 0 | 7695000 |
| Q9EP72 | ER membrane protein complex subunit 7 | Emc7 | 0 | 0 | 0 | 6643800 | 0 | 6384900 |
| H3BJ30;H3BJW3;Q6NVF9;H3BKW0 | Cleavage and polyadenylation specificity factor subunit 6 | Cpsf6 | 0 | 0 | 0 | 6546500 | 0 | 5123500 |
| Q9D7M1 | Glucose-induced degradation protein 8 homolog | Gid8 | 0 | 0 | 0 | 6165000 | 4117200 | 0 |
| Q9CXF4 | TBC1 domain family member 15 | Tbc1d15 | 0 | 0 | 4980000 | 6074400 | 6042800 | 0 |
| Q8BVF2;A0A0A6YXV1 | Phosducin-like protein 3 | Pdcl3 | 0 | 0 | 0 | 5870500 | 3591300 | 4678700 |
| E9QPD7;G5E8R3;Q05920;A0A286YCC5;A0A494B912 | Pyruvate carboxylase;Pyruvate carboxylase, mitochondrial | Pcx;Pc | 0 | 0 | 4585000 | 5870000 | 5114100 | 5155100 |
| Q01965;E9PV93;E9PX73 | T-lymphocyte surface antigen Ly-9 | Ly9 | 0 | 4466700 | 0 | 5869600 | 5847200 | 0 |
| Q8QZS1;E0CX19 | 3-hydroxyisobutyryl-CoA hydrolase, mitochondrial | Hibch | 0 | 0 | 5106600 | 5373300 | 5868900 | 6422200 |
| O55098 | Serine/threonine-protein kinase 10 | Stk10 | 0 | 0 | 0 | 5288000 | 5814700 | 1933500 |
| Q99KR3 | Beta-lactamase-like protein 2 | Lactb2 | 0 | 0 | 0 | 4929000 | 4772400 | 4307900 |
| O35864;A0A087WQA8;A0A087WQ60;A0A087WRH6 | COP9 signalosome complex subunit 5 | Cops5 | 0 | 0 | 4719400 | 4863400 | 5500900 | 5215400 |
| A0A452J8C7;Q8K4Q8 | Collectin-12 | Colec12 | 0 | 0 | 0 | 4668300 | 3523900 | 3685800 |
| P51855;A2AQN9;Q3UEE2 | Glutathione synthetase | Gss | 0 | 0 | 0 | 4656600 | 4244800 | 5227100 |
| Q9CYN9 | Renin receptor | Atp6ap2 | 5102500 | 0 | 0 | 4496300 | 4036000 | 4930900 |
| Q9D6U8 | Protein FAM162A | Fam162a | 0 | 0 | 6430500 | 4468200 | 5466000 | 5195700 |
| A0A494BAJ6;Q9WUL7 | ADP-ribosylation factor-like protein 3 | Arl3 | 0 | 0 | 5089500 | 4424400 | 5249500 | 0 |
| F7D5L2;Q99J09 | Methylosome protein 50 | Wdr77 | 0 | 4560400 | 0 | 4423000 | 6155200 | 5673700 |
| Q6PB93 | Polypeptide N-acetylgalactosaminyltransferase 2;Polypeptide N-acetylgalactosaminyltransferase 2 soluble form | Galnt2 | 4644000 | 0 | 0 | 4384300 | 0 | 4590700 |
| E9PYK3 | Protein mono-ADP-ribosyltransferase PARP4 | Parp4 | 0 | 0 | 0 | 4312100 | 3423300 | 0 |
| Q8CAA7;E0CX81 | Glucose 1,6-bisphosphate synthase | Pgm2l1 | 0 | 0 | 0 | 4158200 | 4755300 | 4879400 |
| Q9R0A0 | Peroxisomal membrane protein PEX14 | Pex14 | 0 | 3340900 | 0 | 4050000 | 4371800 | 0 |
| H3BJ71;H3BKL6;H3BL19;H3BJI6;H3BK44;E9PY90;H3BK48;E9Q3S2;H3BKH2;H3BJS0;A0A1W2P711;Q91ZV0;H3BJ35 | Melanoma inhibitory activity protein 2 | Ctage5;Mia2 | 0 | 0 | 3469900 | 3941400 | 0 | 3408200 |
| A2AAN0;O35250 | Exocyst complex component 7 | Exoc7 | 0 | 0 | 0 | 3725700 | 3080900 | 0 |
| Q922V4;D3Z4V1;F8WI31 | Pleiotropic regulator 1 | Plrg1 | 0 | 0 | 0 | 3572500 | 0 | 4909800 |
| Q9QYF9;Q8VCV2;Q8CBD0 | Protein NDRG3 | Ndrg3 | 4143100 | 0 | 0 | 3500700 | 3392000 | 0 |
| F8VQE9;Q8VHH5;A0A0G2JER6;A0A1D5RMG4;A0A0G2JDW1;A0A087WRF2;Q8BXK8 | Arf-GAP with GTPase, ANK repeat and PH domain-containing protein 3 | Agap3 | 3294500 | 0 | 0 | 3377800 | 3465300 | 3474200 |
| Q6PAM1;A8Y5J8;A2ADZ3;A2ADZ4;A2ADZ2 | Alpha-taxilin | Txlna | 0 | 3510300 | 0 | 3306200 | 4230000 | 3259200 |
| Q925I1;V9GWS5;H3BKI6;H3BK90 | ATPase family AAA domain-containing protein 3 | Atad3 | 0 | 4038600 | 0 | 3302000 | 3575700 | 3071800 |
| Q9DAV6;Q9D6A7;I7HJI5 | R86 | Serpinb9b | 0 | 0 | 0 | 3199900 | 3727800 | 5439900 |
| F8VPX1;Q6A4J8;E9PXY8;G3UWR8;E0CY04 | Ubiquitin carboxyl-terminal hydrolase;Ubiquitin carboxyl-terminal hydrolase 7 | Usp7 | 0 | 0 | 3181900 | 3129300 | 3207800 | 0 |
| Q61102 | ATP-binding cassette sub-family B member 7, mitochondrial | Abcb7 | 0 | 3339600 | 0 | 2834600 | 3720200 | 3630200 |
| Q8QZZ7;D3YXX8;A0A0J9YVB8;D3Z5E8;G3X9K8 | EKC/KEOPS complex subunit Tprkb | Tprkb | 0 | 0 | 0 | 2823500 | 0 | 2934300 |
| Q9JIP4;B1PL19 | Pannexin-1 | Panx1 | 0 | 0 | 0 | 2771700 | 2645000 | 2898400 |
| A0A0J9YUD5;B9EJ54;E9Q880 | Nucleoporin 205 | Nup205 | 0 | 0 | 2378400 | 2678200 | 2711300 | 0 |
| Q8C4V1;G3X9N1;D3Z5T4 | Rho GTPase-activating protein 24 | Arhgap24 | 0 | 0 | 0 | 2559700 | 3128500 | 0 |
| Q8VE97;Q542V3;A0A0A6YWA5 | Serine/arginine-rich splicing factor 4 | Srsf4 | 0 | 0 | 0 | 2543500 | 0 | 2404200 |
| Q99M71 | Mammalian ependymin-related protein 1 | Epdr1 | 2301800 | 0 | 0 | 2254600 | 2461700 | 0 |
| E9QPI5;Q6A026 | Sister chromatid cohesion protein PDS5 homolog A | Pds5a | 0 | 0 | 2606300 | 2141300 | 0 | 1844500 |
| E9QL65;Q8CI04 | Conserved oligomeric Golgi complex subunit 3 | Cog3 | 0 | 0 | 0 | 2072200 | 0 | 1892800 |
| Q91WG2 | Rab GTPase-binding effector protein 2 | Rabep2 | 0 | 0 | 0 | 2014300 | 2140500 | 0 |
| Q9CRB0 | Sorting nexin-24 | Snx24 | 0 | 0 | 0 | 1624900 | 1500700 | 0 |
| A0A0R4J015;B2RX12 | Canalicular multispecific organic anion transporter 2 | Abcc3 | 0 | 0 | 3311400 | 0 | 4726700 | 4941900 |
| A0A2I3BRS9;Q8BH93 | MAPK-interacting and spindle-stabilizing protein-like | Mapk1ip1l | 16901000 | 0 | 0 | 0 | 10264000 | 12671000 |
| B0V2V6;A0A494BAK1;Q3UH70;Q9ESU6 | Bromodomain-containing protein 4 | Brd4 | 0 | 1223700 | 0 | 0 | 1320300 | 1309000 |
| A6PWS5 | Gelsolin | Gsn | 0 | 0 | 0 | 0 | 48026000 | 40720000 |
| E9Q6W2;Q9D2R6 | Cytochrome c oxidase assembly factor 3 homolog, mitochondrial | Coa3 | 0 | 0 | 0 | 0 | 10969000 | 9333200 |
| P32233 | Developmentally-regulated GTP-binding protein 1 | Drg1 | 0 | 0 | 0 | 0 | 4376100 | 4557900 |
| Q09200;A0A1W2P6F0 | Beta-1,4 N-acetylgalactosaminyltransferase 1 | B4galnt1 | 4006300 | 0 | 0 | 0 | 4159400 | 3589500 |
| Q3TL72;Q8C878 | NEDD8-activating enzyme E1 catalytic subunit | Uba3 | 0 | 0 | 0 | 0 | 5819300 | 5302400 |
| Q9CQW9 | Interferon-induced transmembrane protein 3 | Ifitm3 | 30336000 | 0 | 0 | 0 | 37172000 | 40846000 |
| Q9CZR8;Q9CX33;D3Z4M7 | Elongation factor Ts, mitochondrial | Tsfm | 0 | 0 | 12511000 | 0 | 11212000 | 14232000 |
| Q9D0F3;D3Z5B9 | Protein ERGIC-53 | Lman1 | 0 | 0 | 15992000 | 0 | 16393000 | 16766000 |
| Q9DBE8;F6RBY3 | Alpha-1,3/1,6-mannosyltransferase ALG2 | Alg2 | 0 | 0 | 0 | 0 | 5885800 | 5992800 |
| Q9JJV2;D3YWS3 | Profilin-2;Profilin | Pfn2 | 0 | 0 | 0 | 0 | 7183700 | 6738900 |
| Q9JLJ5;Q4V9V3 | Elongation of very long chain fatty acids protein 1;Elongation of very long chain fatty acids protein | Elovl1 | 0 | 0 | 4395600 | 0 | 4061800 | 4024800 |
