# Supplementary material for: Intracellular iron accumulation facilitates mycobacterial infection in old mouse macrophages
Source: GeroScience. 2023 Dec 30;46(2):2739–54. doi: 10.1007/s11357-023-01048-1 (PMC10828278; doi:10.1007/s11357-023-01048-1)
Supplement: Supplementary file 3 — Supplementary file3 (DOCX 23 KB) [file 11357_2023_1048_MOESM3_ESM.docx]

**Supplementary Table 1C: Proteins with Differential Abundance (Uninfected Old BMMs *vs* Uninfected Young BMMs)**

| **Significant** | **-LOG(P-value)** | **Fold Change (Log2)** | **Protein IDs** | **Protein names** | **Gene names** | **Uninfected Old BMMs** | | | **Uninfected Young BMMs** | | |
| --- | --- | --- | --- | --- | --- | --- | --- | --- | --- | --- | --- |
|  |  |  |  |  |  | **LFQ intensity 1967_br1_tr1** | **LFQ intensity 1967_br1_tr2** | **LFQ intensity 1967_br1_tr3** | **LFQ intensity 1967_br3_tr1** | **LFQ intensity 1967_br3_tr2** | **LFQ intensity 1967_br3_tr3** |
| + | 2.993070892 | 4.104220708 | P11247;F7DC05 | Myeloperoxidase;Myeloperoxidase light chain;Myeloperoxidase heavy chain | Mpo | 24.11854362 | 24.46076393 | 24.46176338 | 19.56902122 | 20.0249939 | 21.13439369 |
| + | 4.994609724 | 3.036848704 | P49290 | Eosinophil peroxidase;Eosinophil peroxidase light chain;Eosinophil peroxidase heavy chain | Epx | 25.31295586 | 25.21922493 | 25.4247036 | 22.46148872 | 22.15523911 | 22.22961044 |
| + | 4.226845195 | 2.748422623 | Q61878 | Bone marrow proteoglycan;Eosinophil granule major basic protein | Prg2 | 28.64182472 | 28.66081619 | 28.60434723 | 26.01173592 | 26.06903076 | 25.5809536 |
| + | 4.405215755 | 1.669260025 | E9Q0F0 | Keratin 78 | Krt78 | 25.19764328 | 25.20985031 | 25.10017014 | 23.62756157 | 23.36024666 | 23.51207542 |
| + | 1.696668771 | 1.349707921 | A0A3Q4EIB3;A0A3Q4EG45;E9PZA6 | vomeronasal 2, receptor 113 | Vmn2r113 | 26.60385323 | 25.98665619 | 26.73389053 | NaN | 25.10908699 | 25.07443047 |
| + | 1.75607938 | 1.085156123 | P27661 | Histone H2AX | H2afx | 24.98450661 | 24.86764145 | 25.11981201 | 23.40911674 | 23.9762249 | 24.33115005 |
| + | 2.220119987 | 1.044712702 | P29595;A0A2I3BRG0 | NEDD8 | Nedd8 | 23.950737 | 23.48689461 | 23.3269062 | 22.52078056 | 22.45226669 | 22.65735245 |
| + | 1.958187634 | 0.993357658 | Q80SY3 | V-type proton ATPase subunit d 2 | Atp6v0d2 | 23.43058586 | 23.75399208 | 23.31451225 | 22.44387054 | 22.5688076 | NaN |
| + | 4.654073941 | -0.96593984 | Q61703;G3X977;F6SGM1 | Inter-alpha-trypsin inhibitor heavy chain H2 | Itih2 | 24.84507942 | 24.83166313 | 24.73112297 | 25.79367256 | 25.78862953 | 25.72338295 |
| + | 1.579824405 | -1.083986282 | A3KGL9;F6W687;P09602;Q5XK38;B7ZCQ3 | Non-histone chromosomal protein HMG-17 | Hmgn2 | 21.80597305 | 21.98728561 | NaN | 23.35501289 | 22.68071747 | 22.90611649 |
| + | 2.000540867 | -1.090227127 | Q9CTN4 | Rho-related BTB domain-containing protein 3 | Rhobtb3 | NaN | 24.34448624 | 24.56360435 | NaN | 25.55121613 | 25.53732872 |
| + | 1.166053248 | -1.098037084 | P32261;A0A0A6YXS8;A0A0A6YX49;A0A0A6YWH7 | Antithrombin-III | Serpinc1 | 24.12597084 | 23.77223396 | 22.73894501 | 24.50294113 | 24.94628143 | 24.4820385 |
| + | 1.120501103 | -1.171578407 | Q64669 | NAD(P)H dehydrogenase [quinone] 1 | Nqo1 | NaN | 22.51998901 | 21.62996483 | 22.82063293 | 23.57265282 | 23.34638023 |
| + | 3.160318016 | -1.172434489 | P07724 | Serum albumin | Alb | 30.90131569 | 31.28366661 | 31.14576721 | 32.32514572 | 32.17744827 | 32.34545898 |
| + | 3.221133934 | -1.176109314 | Q32NZ6 | Transmembrane channel-like protein 5 | Tmc5 | 28.56383705 | 28.93024445 | 28.66715813 | 29.82382393 | 29.9903717 | 29.87537193 |
| + | 1.578008032 | -1.231017113 | Q60963;E9Q330;E9Q6J0 | Platelet-activating factor acetylhydrolase | Pla2g7 | 22.13592911 | NaN | 22.24519157 | 23.20825958 | 23.17331696 | 23.88315582 |
| + | 4.590976275 | -1.292211533 | P30115;A0A087WQI6;Q6P8Q0;P13745;D3Z6A6;D3YZV3;P24472;P10648 | Glutathione S-transferase A3 | Gsta3 | 24.95588875 | 24.77339172 | 24.84689903 | 26.12078094 | 26.20228386 | 26.1297493 |
| + | 3.422520599 | -1.418572108 | A0A2K6EDJ7;E9Q5L2;E9PVD2;A6X935 | Inter alpha-trypsin inhibitor, heavy chain 4 | Itih4 | 23.59775734 | 23.42149734 | 23.46063995 | 25.02766228 | 25.02888489 | 24.6790638 |
| + | 4.568384001 | -1.435453415 | F7CJN9;Q921I1;F7BAE9 | Serotransferrin | Trf;Tf | 26.05895424 | 25.87420082 | 25.88313293 | 27.3698616 | 27.42478371 | 27.32800293 |
| + | 2.398562376 | -1.461322149 | O88630 | Golgi SNAP receptor complex member 1 | Gosr1 | NaN | 20.28048134 | 20.2998333 | 22.02860641 | 21.65197754 | 21.57385445 |
