# Supplementary material for: Intracellular iron accumulation facilitates mycobacterial infection in old mouse macrophages
Source: GeroScience. 2023 Dec 30;46(2):2739–54. doi: 10.1007/s11357-023-01048-1 (PMC10828278; doi:10.1007/s11357-023-01048-1)
Supplement: Supplementary file 4 — Supplementary file4 (DOCX 628 KB) [file 11357_2023_1048_MOESM4_ESM.docx]

| **Significant** | **-LOG(P-value)** | **Fold Change (Log2)** | **Protein IDs** | **Protein names** | **Gene names** | **Uninfected Old BMMs** | | | **Uninfected Young BMMs** | | |
| --- | --- | --- | --- | --- | --- | --- | --- | --- | --- | --- | --- |
|  |  |  |  |  |  | **LFQ intensity 1967_br1_tr1** | **LFQ intensity 1967_br1_tr2** | **LFQ intensity 1967_br1_tr3** | **LFQ intensity 1967_br3_tr1** | **LFQ intensity 1967_br3_tr2** | **LFQ intensity 1967_br3_tr3** |
| **+** | 3.230542331 | -0.946339289 | F6W4D3;D3YY36;Q9DBD0 | Inhibitor of carbonic anhydrase | 1300017J02Rik;Ica | 24.63250732 | 24.51821327 | 24.50754547 | 25.60050964 | 25.57082939 | 25.3259449 |
| **+** | 4.043357359 | -0.904710134 | O70250 | Phosphoglycerate mutase 2 | Pgam2 | 24.84258842 | 24.84919167 | 24.91588402 | 25.73476982 | 25.87638092 | 25.71064377 |
| **+** | 3.205805775 | -0.902074178 | Q9D379;E9PWK1;F6YTS6;D3Z4M3 | Epoxide hydrolase 1 | Ephx1 | 23.67087746 | 23.71156311 | 23.42726326 | 24.52677345 | 24.4526825 | 24.53647041 |
| **+** | 1.109583752 | -0.86984698 | P10833;A0A1B0GRG1;A0A1B0GRT5;P62071 | Ras-related protein R-Ras | Rras | 22.36810303 | 21.62462044 | 22.53188515 | 22.96712303 | 23.49509239 | 22.67193413 |
| **+** | 6.370500444 | -0.856557846 | Q91VB8;P01942;A7M7S6;P06467 | Hemoglobin subunit alpha | haemaglobin alpha 2;Hba | 27.94737434 | 27.94273949 | 27.96251106 | 28.82025146 | 28.7821312 | 28.81991577 |
| **+** | 1.548975252 | -0.839506149 | P55258;Q3TYH2;Q8K386 | Ras-related protein Rab-8A | Rab8a | 23.63621902 | 24.18646431 | 24.29403877 | 24.9077034 | 25.11255264 | 24.61498451 |
| **+** | 4.33020245 | -0.775742213 | P61358;A2A4Q0 | 60S ribosomal protein L27 | Rpl27 | 25.61714363 | 25.55356216 | 25.54654312 | 26.29691124 | 26.41383553 | 26.33372879 |
| **+** | 4.407532333 | -0.756583532 | Q9D1R9;A0A0G2JGY8;A0A0G2JEY6 | 60S ribosomal protein L34 | Rpl34 | 25.43622017 | 25.40727043 | 25.36299133 | 26.16198349 | 26.10185432 | 26.21239471 |
| **+** | 2.573046883 | -0.74835968 | P17897 | Lysozyme C-1 | Lyz1 | 25.15135574 | 25.32563591 | 25.11941528 | 25.97105789 | 26.09452438 | 25.7759037 |
| **+** | 4.118285769 | -0.745095571 | A0A0N4SVU1;P28665;P28666 | Murinoglobulin-1;Murinoglobulin-2 | Mug1;Mug2 | 29.61338615 | 29.60823059 | 29.52576256 | 30.27475357 | 30.31527328 | 30.39263916 |
| **+** | 1.083883546 | -0.738979022 | Q08879 | Fibulin-1 | Fbln1 | NaN | 23.33660698 | 23.0372963 | 24.30667686 | 23.6028614 | 23.86825371 |
| **+** | 3.010860725 | -0.73570315 | V9GX81 | Maestro heat-like repeat family member 6 | Mroh6 | 32.06284714 | 31.9621315 | 32.11042023 | 32.78237915 | NaN | 32.77929306 |
| **+** | 4.149971102 | -0.718830744 | O09172;H3BJA3;A0A0G2JDI4;F6VNW5 | Glutamate--cysteine ligase regulatory subunit | Gclm | 25.55496788 | 25.50706291 | 25.41921616 | 26.23925209 | 26.18861771 | 26.20986938 |
| **+** | 4.122716933 | -0.715838114 | Q6ZWY8;A0A0N4SVF0 | Thymosin beta-10 | Tmsb10 | 27.24181747 | 27.26758385 | 27.32817268 | 28.01265335 | 28.04380035 | 27.92863464 |
| **+** | 2.496008863 | -0.715514183 | Q00623 | Apolipoprotein A-I;Proapolipoprotein A-I;Truncated apolipoprotein A-I | Apoa1 | 25.60851288 | NaN | 25.64501762 | 26.37845421 | 26.30610466 | NaN |
| **+** | 4.713405464 | -0.700379054 | P24270;A2AL20 | Catalase | Cat | 26.34389305 | 26.27776337 | 26.32808685 | 27.04593468 | 27.03138924 | 26.97355652 |
| **+** | 1.482334676 | -0.696221034 | A0A1L1SRX2;O08739;F6XQD0;D3Z4N3;D3YU73 | AMP deaminase 3 | Ampd3 | 21.93470383 | 22.41449356 | 22.52291298 | 23.22128487 | 22.81567764 | 22.92381096 |
| **+** | 4.469617387 | -0.688365936 | A0A494BB95;Q60872;Q3UTA4;Q3TQZ4;J3QP87;A0A1Y7VK80;A0A1Y7VJE9;J3QQ02;J3QPI8;A0A1Y7VLT7;Q8BX20;Q3UT53;J3QMW5;A0A1Y7VNG9;F6YNI8 | Eukaryotic translation initiation factor 1A | Eif1a;Gm8300;Gm2016;Gm2035;Gm2056;Gm6803;Gm5662 | 22.98340988 | 22.87273216 | 22.89727974 | 23.60229492 | 23.60399437 | 23.6122303 |
| **+** | 1.623106138 | -0.680320422 | Q3TET1;Q3UI47;B1AQZ2;P28741;B1AQZ5;Q61771 | Kinesin-like protein;Kinesin-like protein KIF3A | Kif3a | 20.31118965 | 20.63759041 | 20.62301254 | 21.09448814 | NaN | 21.31401443 |
| **+** | 3.40668638 | -0.676535924 | P19973;A2A6J7;A2A6J4;A0A1B0GRF5 | Lymphocyte-specific protein 1 | Lsp1 | 25.50297165 | 25.36111832 | 25.37438011 | 26.02058029 | 26.16520691 | 26.08229065 |
| **+** | 1.114332644 | -0.670197805 | Q80X95;Q6NTA4;G3UYP1 | Ras-related GTP-binding protein A;Ras-related GTP-binding protein B | Rraga;Rragb | 23.10134888 | 22.53067207 | 22.49896812 | NaN | 23.38521767 | 23.37583733 |
| **+** | 2.375933235 | -0.668813705 | A0A1L1SQA8;P62852 | 40S ribosomal protein S25 | Rps25 | 25.81992149 | 25.53130341 | 25.75954056 | 26.37049294 | 26.4989624 | 26.24775124 |
| **+** | 2.515896974 | -0.667628606 | A0A0G2JDW7;Q6ZWU9;A0A0G2JG29;A0A0G2JEX7 | 40S ribosomal protein S27 | Rps27 | 25.25140381 | 25.48566628 | 25.20828819 | 26.09968948 | 25.91802406 | 25.93053055 |
| **+** | 3.059210257 | -0.660463333 | P01027;H3BL60 | Complement C3;Complement C3 beta chain;C3-beta-c;Complement C3 alpha chain;C3a anaphylatoxin;Acylation stimulating protein;Complement C3b alpha chain;Complement C3c alpha chain fragment 1;Complement C3dg fragment;Complement C3g fragment;Complement C3d fragment;Complement C3f fragment;Complement C3c alpha chain fragment 2 | C3 | 25.45233727 | 25.44755173 | 25.49759293 | 26.17375183 | 26.22083092 | 25.98428917 |
| **+** | 1.533015416 | -0.654617945 | P97298;Q5ND37;B7ZC25;F6S1M4;E9PWS2 | Pigment epithelium-derived factor | Serpinf1 | 23.6132431 | 23.16054153 | 22.99645233 | 23.77334023 | 23.96965408 | 23.9910965 |
| **+** | 2.437902597 | -0.65278244 | P62320;A0A1W2P7K5 | Small nuclear ribonucleoprotein Sm D3 | Snrpd3 | 24.34888077 | 24.48578835 | 24.37059402 | 25.25086212 | 24.95415878 | 24.95858955 |
| **+** | 2.965356591 | -0.641450246 | P01029 | Complement C4-B;Complement C4 beta chain;Complement C4 alpha chain;C4a anaphylatoxin;Complement C4 gamma chain | C4b | 25.11699867 | 25.22468185 | 25.0116291 | 25.71250916 | 25.84871292 | 25.71643829 |
| **+** | 3.572729858 | -0.640293757 | Q9D2R0 | Acetoacetyl-CoA synthetase | Aacs | 22.06151009 | 22.09439087 | 21.94831467 | 22.68045998 | 22.62227631 | 22.72236061 |
| **+** | 1.452293902 | -0.635531108 | P50543 | Protein S100-A11 | S100a11 | 26.40662193 | 26.1822834 | 26.10579491 | 26.50470161 | 27.01680183 | 27.07979012 |
| **+** | 2.7619811 | -0.635184606 | Q61549;A0A3B2WB94 | EGF-like module-containing mucin-like hormone receptor-like 1 | Emr1 | 22.9924469 | 23.13819695 | 23.09489059 | 23.57738495 | 23.72286224 | 23.83084106 |
| **+** | 4.840419751 | -0.617631912 | P67984 | 60S ribosomal protein L22 | Rpl22 | 26.05897522 | 26.09820366 | 26.05235672 | 26.70569611 | 26.64801025 | 26.70872498 |
| **+** | 3.356108466 | -0.61412557 | A0A0R4J2B2;Q6WVG3 | BTB/POZ domain-containing protein KCTD12 | Kctd12 | 24.40262604 | 24.4820385 | 24.31858635 | 24.96115303 | 25.0089817 | 25.07549286 |
| **+** | 2.85227127 | -0.613696416 | P62267 | 40S ribosomal protein S23 | Rps23 | 25.08440018 | 25.28911972 | 25.32433319 | 25.80328941 | 25.86135864 | 25.87429428 |
| **+** | 3.346423876 | -0.602908452 | Q8QZY6 | Tetraspanin-14 | Tspan14 | 25.29498482 | 25.44651222 | 25.31648254 | 25.98439789 | 25.98997116 | 25.89233589 |
| **+** | 3.838160344 | -0.595802307 | A0A3Q4EBK4;Q6P1B9;O08539;A0A3Q4EBR8 | Myc box-dependent-interacting protein 1 | Bin1 | 23.64339447 | 23.72004509 | 23.67163277 | 24.22036934 | 24.34170532 | 24.26040459 |
| **+** | 3.39100173 | -0.590287526 | P62631 | Elongation factor 1-alpha 2 | Eef1a2 | 23.99550247 | 23.90582275 | 24.08845139 | 24.56104088 | 24.59741592 | 24.60218239 |
| **+** | 1.749805569 | -0.58531189 | P16460 | Argininosuccinate synthase | Ass1 | 22.70023918 | 23.196661 | 23.02776337 | 23.5148468 | 23.63677216 | 23.52898026 |
| **+** | 2.89468101 | -0.579360326 | P21460;A2APX3 | Cystatin-C | Cst3 | 25.52892113 | 25.33514214 | 25.3734169 | 25.93851662 | 25.96602631 | 26.07101822 |
| **+** | 2.337137133 | -0.578378042 | A0A0N4SVQ1;Q62425 | Cytochrome c oxidase subunit NDUFA4 | Ndufa4 | 24.44714165 | 24.68779182 | 24.75572586 | 25.27330589 | 25.21033478 | 25.14215279 |
| **+** | 2.741278758 | -0.554919561 | Q64523;Q6GSS7 | Histone H2A type 2-C;Histone H2A type 2-A | Hist2h2ac;Hist2h2aa1 | 25.52510071 | 25.75498772 | 25.69101715 | 26.26974297 | 26.20463753 | 26.16148376 |
| **+** | 2.791977505 | -0.544267654 | A2AQU8;Q9D975 | Sulfiredoxin-1 | Srxn1 | 23.78596306 | 23.63134193 | 23.6848526 | 24.26270103 | 24.33108139 | 24.14117813 |
| **+** | 2.883041299 | -0.542371114 | Q9CR57;A0A1L1SUF6 | 60S ribosomal protein L14 | Rpl14 | 26.67855263 | 26.65731049 | 26.69882584 | 27.3512764 | 27.13359451 | 27.17693138 |
| **+** | 3.367430247 | -0.533702215 | Q6ZWV7 | 60S ribosomal protein L35 | Rpl35 | 25.86062622 | 25.98226547 | 26.02318382 | 26.48313141 | 26.50815201 | 26.47589874 |
| **+** | 2.202295027 | -0.533069611 | Q9Z130;D3YTQ3;F6VQH5 | Heterogeneous nuclear ribonucleoprotein D-like | Hnrnpdl | 22.17404938 | 21.93261337 | 21.94288445 | 22.52106857 | 22.67165375 | 22.45603371 |
| **+** | 3.089302736 | -0.519785563 | Q64337;D3YZJ1;F6VD69 | Sequestosome-1 | Sqstm1 | 24.87605286 | 24.73773766 | 24.92705345 | 25.38386726 | 25.35568428 | 25.36064911 |
| **+** | 2.469730217 | -0.51938947 | Q6ZQ06 | Centrosomal protein of 162 kDa | Cep162 | 31.31386757 | NaN | 31.16296959 | 31.74663162 | 31.79331398 | 31.73347855 |
| **+** | 2.485951124 | -0.517604192 | P46062;E9Q0Y4;Q3V403 | Signal-induced proliferation-associated protein 1 | Sipa1 | 21.67560005 | 21.78200722 | 21.58233261 | 22.08102226 | 22.243536 | 22.2681942 |
| **+** | 1.724695253 | -0.516638438 | Q9WTM5;A0A1B0GRW3;A0A1B0GSR4;A0A1B0GT54;A0A1B0GR89 | RuvB-like 2 | Ruvbl2 | 22.979002 | 22.67682648 | 23.08864403 | 23.32512283 | 23.45249367 | 23.51677132 |
| **+** | 3.008693448 | -0.505365372 | G3X8R0;A0A494BBE3;Q60870 | Receptor expression-enhancing protein;Receptor expression-enhancing protein 5 | Reep5 | 24.87825203 | 24.99049187 | 24.96587181 | 25.4014225 | 25.54507065 | 25.40421867 |
| **+** | 2.015544044 | -0.502096176 | P62900;A0A0A6YX26;A0A0A6YXL3 | 60S ribosomal protein L31 | Rpl31 | 26.75493622 | 26.43504524 | 26.66220856 | 27.02136421 | 27.14326286 | 27.19385147 |
| **+** | 1.553127112 | -0.500899633 | H3BJL1;Q8K0Q5 | Rho GTPase-activating protein 18 | Arhgap18 | 19.96479797 | 19.98050308 | 20.06052208 | 20.58719444 | 20.70193291 | 20.21939468 |
| **+** | 3.606345697 | -0.493783315 | Q8BG07 | Phospholipase D4 | Pld4 | 25.39875031 | 25.42300415 | 25.43558502 | 25.95462418 | 25.94810867 | 25.83595657 |
| **+** | 2.815122034 | -0.484585444 | B1ARA3;P61255;B1ARA5 | 60S ribosomal protein L26 | Rpl26 | 26.04264069 | 26.09452438 | 26.09305382 | 26.46804047 | 26.67492104 | 26.54101372 |
| **+** | 1.265446493 | -0.483261108 | A0A140LHP7;O54885 | TYRO protein tyrosine kinase-binding protein | Tyrobp | 23.50385284 | 23.74724579 | 23.64405441 | 24.18895721 | 24.35601997 | 23.79995918 |
| **+** | 1.692107897 | -0.481088003 | H7BX99;P19221 | Prothrombin;Prothrombin;Activation peptide fragment 1;Activation peptide fragment 2;Thrombin light chain;Thrombin heavy chain | F2 | 22.47919083 | 22.8848896 | 22.68566513 | 23.07107162 | 23.25853729 | 23.16340065 |
| **+** | 1.910710417 | -0.479977926 | P11680 | Properdin | Cfp | 23.18916702 | 23.34638023 | 23.48775291 | 23.71983719 | 23.78954887 | 23.95384789 |
| **+** | 1.939450185 | -0.478192012 | Q9EPN1;A0A5F8MPY6 | Neurobeachin | Nbea | 25.16767883 | 25.31544495 | 25.191782 | 25.87923431 | 25.68963242 | 25.54061508 |
| **+** | 3.889532387 | -0.471475601 | Q8K354;A0A338P797;A0A1B0GRG8 | Carbonyl reductase [NADPH] 3 | Cbr3 | 24.50008774 | 24.54020119 | 24.59269142 | 25.01482391 | 25.04764175 | 24.98494148 |
| **+** | 3.053236092 | -0.470552444 | P10639 | Thioredoxin | Txn | 27.191782 | 27.01935005 | 27.11941528 | 27.54752922 | 27.60632515 | 27.5883503 |
| **+** | 1.243362728 | -0.464813868 | Q6PHN9 | Ras-related protein Rab-35 | Rab35 | 22.77828217 | 22.6583786 | 22.58123016 | 23.45763779 | 23.05273247 | 22.90196228 |
| **+** | 2.445933827 | -0.463074366 | A0A0R4J0F6;Q99KY4;D6RHK5;A0A0G2JGP6;A0A0G2JEW6 | Cyclin-G-associated kinase | Gak | 25.94093895 | 26.03747749 | 25.85550117 | 26.51361275 | 26.33473396 | 26.37479401 |
| **+** | 2.634919487 | -0.462656021 | P47915;W4VSN7;A0A1L1SS27;A0A1L1STJ3;A0A1L1SUN1 | 60S ribosomal protein L29 | Rpl29;Gm3550 | 26.04481125 | 26.17239761 | 26.17186356 | 26.53687096 | 26.69617462 | 26.5439949 |
| **+** | 2.446339837 | -0.461406708 | P84104 | Serine/arginine-rich splicing factor 3 | Srsf3 | 25.05771637 | 24.87398911 | 24.84196472 | 25.4461956 | 25.38218689 | 25.32950783 |
| **+** | 2.132027428 | -0.460964203 | Q8CE50 | Sorting nexin-30 | Snx30 | 22.47938919 | 22.28418541 | 22.57588577 | 22.96199226 | 22.91128349 | 22.84907722 |
| **+** | 1.515350978 | -0.45572567 | Q8C522;A0A1L1SSA0 | Endonuclease domain-containing 1 protein | Endod1 | 22.37594414 | NaN | 22.51434135 | NaN | 22.85781479 | 22.94392204 |
| **+** | 1.112530199 | -0.455117861 | P28667 | MARCKS-related protein | Marcksl1 | 22.58189583 | 22.90361786 | NaN | 23.00728989 | 23.28404427 | 23.30228996 |
| **+** | 2.884812473 | -0.448331197 | Q6GQT1;A0A0N4SUX6 | Alpha-2-macroglobulin-P | A2mp | 29.58496857 | 29.70182037 | 29.76296997 | 30.10186958 | 30.12398529 | 30.16889763 |
| **+** | 1.702838965 | -0.448274612 | Q9Z1R9 | Protease, serine 1 (trypsin 1) | Prss1 | 31.44440842 | 31.63287544 | 31.53087616 | 32.19560242 | 31.8967762 | 31.86060524 |
| **+** | 2.300828075 | -0.447901408 | Q8CIB9 | N-acetyltransferase ESCO2 | Esco2 | 29.04637718 | 28.9890728 | 28.9942894 | 29.3033886 | 29.55301666 | 29.51703835 |
| **+** | 2.103696768 | -0.442796071 | P63073;A0A0G2JGT5;A0A0G2JH04;A0A0G2JFB4;A0A0G2JG98 | Eukaryotic translation initiation factor 4E | Eif4e | 24.25248718 | 23.98494148 | 24.08553505 | 24.55303383 | 24.62622643 | 24.47209167 |
| **+** | 1.41692508 | -0.441736221 | Q9DB25;D6RCG2 | Dolichyl-phosphate beta-glucosyltransferase | Alg5 | 23.1061821 | 23.13847733 | 23.11413383 | 23.80084801 | 23.58267784 | 23.30047607 |
| **+** | 1.848782326 | -0.437204997 | Q9JJF9 | Signal peptide peptidase-like 2A | Sppl2a | 22.64074898 | 22.79817963 | 22.91579247 | 23.2060833 | 23.34813881 | 23.11211395 |
| **+** | 2.43926666 | -0.430165609 | P15864;I7HFT9;Q07133 | Histone H1.2 | Hist1h1c | 28.3746357 | 28.56550789 | 28.45328712 | 28.85718155 | 28.98139 | 28.84535599 |
| **+** | 1.264883743 | -0.423856099 | P30416;F6S2D5;F7CAT1 | Peptidyl-prolyl cis-trans isomerase FKBP4;Peptidyl-prolyl cis-trans isomerase FKBP4, N-terminally processed | Fkbp4 | 23.16035652 | 22.93833351 | 22.64904213 | 23.26126671 | 23.44075775 | 23.317276 |
| **+** | 1.720482285 | -0.423743248 | E9QLA5;Q0GNC1;A0A1Y7VM80 | Inverted formin-2 | Inf2 | 22.97599792 | 23.13174248 | 22.92359352 | 23.37597084 | 23.49240494 | NaN |
| **+** | 2.392010818 | -0.419934591 | Q6PGB6 | N-alpha-acetyltransferase 50 | Naa50 | 23.58978081 | 23.46898842 | 23.58428383 | 24.05870628 | 23.98667717 | 23.85747337 |
| **+** | 2.280456296 | -0.419464747 | Q61753;F6ZSB7 | D-3-phosphoglycerate dehydrogenase | Phgdh | 23.34028053 | 23.54910278 | 23.43007469 | 23.76749229 | 23.90646553 | 23.90389442 |
| **+** | 2.008032988 | -0.417889913 | E0CY91;E0CYW1;E0CY38;A0A1Y7VKT9;E0CZB3;Q9EPV8 | Ubiquitin-like protein 5 | Ubl5 | 23.38060188 | 23.44543839 | 23.55461693 | NaN | 23.91497231 | 23.84124565 |
| **+** | 4.332344512 | -0.412071228 | A0A1B0GR11;Q93092 | Transaldolase | Taldo1 | 27.35413742 | 27.30539131 | 27.29482651 | 27.74573517 | 27.73821449 | 27.70661926 |
| **+** | 1.554580972 | -0.410545985 | P54227;D3Z1Z8;D3Z5N2 | Stathmin | Stmn1 | 23.68688393 | 23.35796738 | 23.30396271 | 23.86419678 | 23.89550591 | 23.82074928 |
| **+** | 2.370015277 | -0.404209137 | Q8CBB6;Q8CGP2;Q8CGP1;Q6ZWY9;Q64525;Q64478;Q64475;P10854;P10853;Q9D2U9;Q8CGP0;Q64524;P70696 | Histone H2B;Histone H2B type 1-P;Histone H2B type 1-K;Histone H2B type 1-C/E/G;Histone H2B type 2-B;Histone H2B type 1-H;Histone H2B type 1-B;Histone H2B type 1-M;Histone H2B type 1-F/J/L;Histone H2B type 3-A;Histone H2B type 3-B;Histone H2B type 2-E;Histone H2B type 1-A | Hist1h2br;Hist1h2bp;Hist1h2bk;Hist1h2bc;Hist2h2bb;Hist1h2bh;Hist1h2bb;Hist1h2bm;Hist1h2bf;Hist3h2ba;Hist3h2bb;Hist2h2be;Hist1h2ba | 30.35877609 | 30.42612457 | 30.4841423 | 30.80770111 | 30.93754387 | 30.7364254 |
| **+** | 1.541227689 | -0.397247314 | Q61152 | Tyrosine-protein phosphatase non-receptor type 18 | Ptpn18 | 23.25258827 | 23.33183289 | 23.09294128 | 23.6132431 | 23.79372215 | 23.46213913 |
| **+** | 1.577490194 | -0.397162755 | Q99K94;Q8C3V4;A0A087WSP5;P42225;A0A087WRI1;Q8CFQ1;A0A087WSQ5 | Signal transducer and activator of transcription;Signal transducer and activator of transcription 1 | Stat1 | 22.35124207 | 22.39325333 | 22.06338882 | 22.73851204 | 22.69376945 | 22.56709099 |
| **+** | 1.361070394 | -0.396087646 | P25911;P16277 | Tyrosine-protein kinase Lyn | Lyn | 23.26198387 | 23.56569672 | 23.36238861 | 23.99774361 | 23.68688393 | 23.69370461 |
| **+** | 1.933883532 | -0.393330256 | A0A0B4J1M6;E9PUV2;E9PVR4 |  | Fcgr3 | 23.1024704 | 23.05864143 | 22.83109283 | 23.42700768 | 23.33128738 | 23.41390038 |
| **+** | 1.86514495 | -0.391995748 | Q8CGP6;C0HKE7;C0HKE1;C0HKE2;C0HKE3;C0HKE4;C0HKE5;C0HKE6;C0HKE9;A0A0N4SV66;Q8CGP4;C0HKE8;Q8BFU2;Q8CGP5;Q8CGP7;Q8R1M2;G3UWL7 | Histone H2A type 1-H;Histone H2A;Histone H2A type 3;Histone H2A type 1-F;Histone H2A type 1-K;Histone H2A.J | Hist1h2ah;Hist1h2aa;Hist3h2a;Hist1h2af;Hist1h2ak;H2afj | 29.79076195 | 29.64204788 | 29.95670891 | 30.21512032 | 30.14758682 | 30.20279884 |
| **+** | 2.825809157 | -0.390940984 | Q3TXV4;Q921E2;Q3TM43;D6RFT6 | Ras-related protein Rab-31 | Rab31 | 23.47357941 | 23.50554848 | 23.5135231 | 23.79094124 | 23.93021584 | 23.94431686 |
| **+** | 1.686104658 | -0.389880498 | Q921W0 | Charged multivesicular body protein 1a | Chmp1a | 22.94628143 | 22.74825096 | 23.07581139 | 23.36305809 | 23.35178185 | 23.22514534 |
| **+** | 2.404206553 | -0.385889053 | Q91X52;A2AC16 | L-xylulose reductase | Dcxr | 22.67232323 | 22.52649879 | 22.60984421 | 23.051157 | 23.02272606 | 22.89245033 |
| **+** | 2.265059227 | -0.384876251 | Q8BL80;D3Z1Y5;A0A2I3BRZ0 | Rho GTPase-activating protein 22 | Arhgap22 | 21.56699753 | 21.76838112 | 21.56843758 | 22.06015968 | 21.98554802 | 22.01273727 |
| **+** | 2.288125602 | -0.381995519 | P21956 | Lactadherin | Mfge8 | 25.42751884 | 25.61520958 | 25.46404076 | 25.83561897 | 25.95883369 | 25.85830307 |
| **+** | 2.556374619 | -0.380467097 | B1AQR8;G3X9T7;O08573 | Galectin;Galectin-9 | Lgals9 | 24.59792709 | 24.52097321 | 24.50718307 | 25.01767349 | 24.84531975 | 24.90449142 |
| **+** | 2.220288026 | -0.376431147 | Q8BMS9;A2APB1;A2AVI0 | Ras association domain-containing protein 2 | Rassf2 | 23.56232262 | 23.32292366 | 23.44468117 | 23.80774117 | 23.85033607 | 23.80114365 |
| **+** | 1.985233594 | -0.372491201 | S4R2J9;A0A0A0MQ79;Q3TLH4;S4R294;S4R2L9;S4R209;S4R2E2 | Protein PRRC2C | Prrc2c | 22.25747299 | 22.2617836 | 22.11518288 | NaN | 22.60722733 | 22.56071472 |
| **+** | 1.371573715 | -0.37228775 | Q6A0D4;D3Z731 | Raftlin | Rftn1 | 22.84563637 | 23.01280594 | 23.25255775 | 23.48897934 | 23.40937614 | 23.32950783 |
| **+** | 1.381367444 | -0.372176488 | P14069 | Protein S100-A6 | S100a6 | 28.67266464 | 28.57675934 | 28.48608017 | 28.81796646 | 29.17676544 | 28.85730171 |
| **+** | 2.80076425 | -0.372080485 | P26040 | Ezrin | Ezr | 25.94543266 | 25.98685074 | 26.08202744 | 26.32321548 | 26.39556694 | 26.41176987 |
| **+** | 1.843444967 | -0.368232727 | P43274 | Histone H1.4 | Hist1h1e | 26.79600143 | 26.94748497 | 27.08294106 | 27.30469513 | 27.36861229 | 27.25781822 |
| **+** | 2.037737 | -0.367841085 | O70274;Q63739 | Protein tyrosine phosphatase type IVA 2;Protein tyrosine phosphatase type IVA 1 | Ptp4a2;Ptp4a1 | 23.17395782 | 23.10915184 | 23.10749245 | 23.53587723 | 23.60444641 | 23.35380173 |
| **+** | 1.240407864 | -0.366720835 | Q8K4Z5 | Splicing factor 3A subunit 1 | Sf3a1 | 22.71208954 | 22.33736992 | 22.73849106 | 22.86748123 | 23.03650856 | 22.98412323 |
| **+** | 2.71329867 | -0.365947088 | P07141;D3Z090;D3YTW1;F6RNW8 | Macrophage colony-stimulating factor 1;Processed macrophage colony-stimulating factor 1 | Csf1 | 25.43799973 | 25.37085915 | 25.50218391 | 25.75279427 | 25.86641502 | 25.78967476 |
| **+** | 1.876532003 | -0.363375346 | A0A0U1RNK7;E9PX48;A2A9M5;A2A9M4;Q8R1A4;A0A0U1RNY4;A2AW79;F6ZJ55 | Dedicator of cytokinesis protein 7 | Dock7 | 31.83774567 | 32.08806229 | 32.01587677 | 32.39693069 | 32.37533951 | 32.25954056 |
| **+** | 1.253298842 | -0.359767596 | Q9D024;F7C265;B1AR93 | Coiled-coil domain-containing protein 47 | Ccdc47 | 23.3742466 | NaN | 23.23806381 | 23.72265244 | 23.502882 | 23.77223396 |
| **+** | 1.77109114 | -0.359216054 | Q9DCG9;Q8VCR4 | Multifunctional methyltransferase subunit TRM112-like protein | Trmt112 | 23.99912071 | 23.77575302 | 23.73913002 | 24.27575874 | 24.13586617 | 24.18002701 |
| **+** | 6.179095889 | -0.356132507 | P14131 | 40S ribosomal protein S16 | Rps16 | 25.78389168 | 25.77989197 | 25.77582932 | 26.12626648 | 26.13461304 | 26.14713097 |
| **+** | 2.128806818 | -0.354484558 | E9Q7Q3;D3Z6I8 | Tropomyosin 3, gamma | Tpm3 | 26.30246544 | 26.44189835 | 26.5036087 | 26.78406715 | 26.8289299 | 26.69842911 |
| **+** | 4.451091592 | -0.350561778 | Q8BP67 | 60S ribosomal protein L24 | Rpl24 | 25.99856186 | 25.98320198 | 26.00489616 | 26.37787437 | 26.32556725 | 26.33490372 |
| **+** | 2.807885297 | -0.350432714 | E9QN37;A1L314;Q5RKV8 | Macrophage-expressed gene 1 protein | Mpeg1 | 26.72129822 | 26.76167107 | 26.78231812 | 27.1848011 | 27.0415554 | 27.09022903 |
| **+** | 2.105734487 | -0.342969259 | P43276 | Histone H1.5 | Hist1h1b | 27.18555832 | 27.11812782 | 26.97967339 | 27.38648415 | 27.42390251 | 27.50188065 |
| **+** | 3.467084835 | -0.337812424 | E9Q604;G5E8F1;E9Q5K8;Q3U1U4;P05555;A0A0R4J1B4;D6RJ73 | Integrin alpha-M | Itgam | 25.94407082 | 25.9405117 | 25.91957092 | 26.29551125 | 26.3066082 | 26.21547127 |
| **+** | 1.608815542 | -0.335325241 | D3YU17;Q8VCM8 | Nicalin | Ncln | 22.9516449 | 22.64530754 | 22.8720932 | 23.14314079 | 23.20952415 | 23.12235641 |
| **+** | 3.227116748 | -0.334529877 | Q542I8;P11835;M0QWA7;D3YYP8;D3Z1S4;M0QWJ6 | Integrin beta;Integrin beta-2 | Itgb2 | 25.92861176 | 25.93433952 | 25.95890045 | 26.32795143 | 26.2819252 | 26.21556473 |
| **+** | 1.576364528 | -0.330800374 | E9QKA4;A2A8V8;A2A8V9;E9PUK6;Q52KI8;F6T4M4;A2A983;F6UK16 | Serine/arginine repetitive matrix protein 1 | Srrm1 | 22.34597397 | 22.53055382 | 22.52353477 | 22.94685173 | 22.73772812 | 22.70788383 |
| **+** | 3.801029829 | -0.327503204 | P35700;B1AXW5;B1AXW6;B1AXW4 | Peroxiredoxin-1 | Prdx1 | 31.40486908 | 31.42787361 | 31.42196846 | 31.79059792 | 31.72866249 | 31.71796036 |
| **+** | 1.584850594 | -0.327075322 | Q6IRU5;F7BHJ0 | Clathrin light chain B | Cltb | 24.3347683 | 24.46432114 | 24.27994347 | 24.81401634 | 24.69943428 | 24.54680824 |
| **+** | 1.60763659 | -0.326054891 | Q9D6Y9;F6ZHD8;G3UW30 | 1,4-alpha-glucan-branching enzyme | Gbe1 | 22.98815155 | 22.76350594 | 22.73405075 | 23.1049366 | 23.24820709 | 23.11072922 |
| **+** | 1.778229779 | -0.32414945 | P49615;A0A0G2JDL3 | Cyclin-dependent-like kinase 5 | Cdk5 | 22.0237751 | 21.98137283 | 21.93768692 | 22.18422699 | 22.45085907 | 22.28019714 |
| **+** | 2.160078228 | -0.321843465 | A0A1W2P7G2;Q9D1J3;A0A1W2P6N2 | SAP domain-containing ribonucleoprotein | Sarnp | 23.23890877 | 23.24318886 | 23.21319199 | 23.55086327 | 23.66275215 | 23.44720459 |
| **+** | 1.905393624 | -0.319378535 | Q05144;A0A2R8VHH0 | Ras-related C3 botulinum toxin substrate 2 | Rac2 | 26.9770546 | 27.16365433 | 27.20704079 | 27.4186039 | 27.40835571 | 27.4789257 |
| **+** | 1.316205867 | -0.318446477 | O09106 | Histone deacetylase 1 | Hdac1 | 23.11205101 | 22.93234444 | 22.8188591 | 23.18292427 | 23.42072678 | 23.21494293 |
| **+** | 2.35552564 | -0.316671371 | P47791 | Glutathione reductase, mitochondrial | Gsr | 24.24408913 | 24.27824211 | 24.25486565 | 24.46855354 | 24.63561058 | 24.62304688 |
| **+** | 2.719255629 | -0.316144943 | A0A1W2P768;P84228;F8WI35;A0A8I4SYN6 | Histone H3.2;Histone H3 | Hist1h3b;H3f3a | 29.45561981 | 29.53713989 | 29.42991829 | 29.75437927 | 29.84810448 | 29.76862907 |
| **+** | 1.517968282 | -0.309821447 | J3QNU6;Q8BWG8;E0CY53;E0CYB1 | Beta-arrestin-1 | Arrb1 | 24.2777462 | 24.3773613 | 24.29663086 | 24.74760437 | 24.68072701 | 24.45287132 |
| **+** | 2.338790615 | -0.309744517 | D3YYM6;D3Z1S8;Q91V55;P97461 | 40S ribosomal protein S5;40S ribosomal protein S5, N-terminally processed | Rps5 | 25.30162811 | 25.42710304 | 25.40944099 | 25.61554527 | 25.71690941 | 25.73495102 |
| **+** | 1.489460775 | -0.309712728 | Q9JIW9;F6QC68 | Ras-related protein Ral-B | Ralb | 23.11130333 | 23.31506538 | 23.36105156 | 23.61918831 | 23.6408596 | 23.45651054 |
| **+** | 2.109860783 | -0.309050242 | A0A1W2P6X3;Q9JL26;A2AB60;G3UWI1 | Formin-like protein 1 | Fmnl1 | 23.58210373 | 23.50772858 | 23.45638466 | 23.90242195 | 23.72941208 | 23.84153366 |
| **+** | 1.933847779 | -0.30832545 | Q9CX56;Q9CPS5;Q3TG45 | 26S proteasome non-ATPase regulatory subunit 8 | Psmd8 | 23.65086174 | 23.69774055 | 23.57945824 | 24.018013 | 23.8292923 | 24.00573158 |
| **+** | 1.44944505 | -0.308190028 | P63213 | Guanine nucleotide-binding protein G(I)/G(S)/G(O) subunit gamma-2 | Gng2 | 24.70688248 | 24.86740494 | 24.60523796 | 25.15201378 | 24.93759727 | 25.01448441 |
| **+** | 2.246320455 | -0.306035995 | Q9R0P9 | Ubiquitin carboxyl-terminal hydrolase isozyme L1 | Uchl1 | 27.10329628 | 27.14345741 | 26.99312973 | 27.31855202 | 27.43181229 | 27.40762711 |
| **+** | 2.22306072 | -0.305091222 | Q3TLP8;P63001;P60764;A2AC13 | Ras-related C3 botulinum toxin substrate 1;Ras-related C3 botulinum toxin substrate 3 | Rac1;Rac3 | 26.38322449 | 26.30716324 | 26.26459885 | 26.7137146 | 26.56733704 | 26.5892086 |
| **+** | 2.030031991 | -0.302041372 | E9Q2A6;Q9QVP9;Q3UDE9;F7CCX1 | Protein-tyrosine kinase 2-beta | Ptk2b | 23.5641861 | 23.41351318 | 23.6041069 | 23.87427139 | 23.77876282 | 23.83489609 |
| **+** | 1.621237879 | -0.300844828 | A2AFJ1;Q60973;A2AFI9;F6ZLC6;F6U539 | Histone-binding protein RBBP7 | Rbbp7 | 22.66296959 | 22.73307991 | 22.80286026 | 23.17113495 | 22.91436958 | 23.01593971 |
| **+** | 3.122216159 | -0.298580805 | P19096;A0A0U1RNJ1;A0A0U1RPP5 | Fatty acid synthase;[Acyl-carrier-protein] S-acetyltransferase;[Acyl-carrier-protein] S-malonyltransferase;3-oxoacyl-[acyl-carrier-protein] synthase;3-oxoacyl-[acyl-carrier-protein] reductase;3-hydroxyacyl-[acyl-carrier-protein] dehydratase;Enoyl-[acyl-carrier-protein] reductase;Oleoyl-[acyl-carrier-protein] hydrolase | Fasn | 24.95619774 | 24.95189285 | 24.98997116 | 25.27430153 | 25.31084251 | 25.20866013 |
| **+** | 1.547431306 | -0.29639562 | Q80UE5;O70318;Q80UE4;Q8C928;A0A1W2P6I5;A0A1W2P7I2;A0A1W2P7I4;A0A1W2P896;A0A1W2P7H7;A0A1W2P6H2;A0A1W2P8C0 | Band 4.1-like protein 2 | Epb4.1l2;Epb41l2 | 23.16034126 | 23.31146622 | 23.31782722 | 23.70007133 | 23.46438408 | 23.51436615 |
| **+** | 1.859357495 | -0.293810527 | Q9D2V7;G3X9L5;E9PYU1 | Coronin-7 | Coro7 | 24.05779839 | 24.21889305 | 24.12265778 | 24.48787498 | 24.46998215 | 24.32292366 |
| **+** | 2.520325931 | -0.2915802 | P25444;D3YVC1;D3YWJ3 | 40S ribosomal protein S2 | Rps2 | 26.10323524 | 25.96840096 | 26.08068657 | 26.36310768 | 26.30667686 | 26.35727882 |
| **+** | 2.439606687 | -0.291528702 | P14148;F6XI62 | 60S ribosomal protein L7 | Rpl7 | 26.08415794 | 26.12750626 | 26.17983818 | 26.36969566 | 26.49801826 | 26.39837456 |
| **+** | 2.698152669 | -0.287104289 | Q07113 | Cation-independent mannose-6-phosphate receptor | Igf2r | 24.46201324 | 24.4078846 | 24.38416481 | 24.64030838 | 24.72930717 | 24.74575996 |
| **+** | 1.749008272 | -0.28706042 | P62830;A2A6F8 | 60S ribosomal protein L23 | Rpl23 | 26.1313591 | 26.2655468 | 26.36205482 | 26.6031456 | 26.50718307 | 26.50981331 |
| **+** | 1.879949675 | -0.285822233 | A0A087WRY3;Q80XU3 | Nuclear ubiquitous casein and cyclin-dependent kinase substrate 1 | Nucks1 | 24.48744583 | 24.5545578 | 24.4457531 | NaN | 24.82909966 | 24.73438263 |
| **+** | 2.022875286 | -0.285514832 | G3X9Q3;Q3TBD2 | Minor histocompatibility protein HA-1 | Hmha1 | 23.99722672 | 23.83643723 | 23.86296844 | 24.23095894 | 24.20732117 | 24.11489677 |
| **+** | 3.69316146 | -0.283385595 | O88844;A0A087WPT4;A0A087WRS9;D3YVY3;A0A087WRM4 | Isocitrate dehydrogenase [NADP] cytoplasmic | Idh1 | 26.21517563 | 26.25369453 | 26.24963188 | 26.55699921 | 26.51618576 | 26.49547386 |
| **+** | 2.189566721 | -0.28329277 | O70475;D3YXP9 | UDP-glucose 6-dehydrogenase | Ugdh | 22.60485268 | 22.57597923 | 22.58540916 | 22.80149841 | 22.97741318 | 22.83720779 |
| **+** | 2.224956102 | -0.281568527 | A2A547;P84099 | Ribosomal protein L19;60S ribosomal protein L19 | Rpl19 | 26.26375771 | 26.19432068 | 26.13838768 | 26.41459465 | 26.54755783 | 26.47901917 |
| **+** | 1.555261514 | -0.281085332 | Q3TUQ7;Q5EG47;Q8BRK8 | 5-AMP-activated protein kinase catalytic subunit alpha-1 | Prkaa1 | 23.21032715 | 23.01411819 | 23.28559685 | 23.44316292 | 23.48799706 | 23.42213821 |
| **+** | 1.606920305 | -0.280618032 | F6YVP7;P62270;S4R1N6;A0A1Y7VKY1;A0A3Q4EGP3 | 40S ribosomal protein S18 | Gm10260;Rps18 | 26.44977379 | 26.36334229 | 26.25095177 | 26.74599075 | 26.58849335 | 26.57143784 |
| **+** | 1.489625574 | -0.278013865 | Q3UHJ0;A0A571BDM4 | AP2-associated protein kinase 1 | Aak1 | 22.28833199 | 22.46383476 | 22.30220795 | 22.54672623 | 22.75892067 | 22.58276939 |
| **+** | 2.653069802 | -0.277805964 | P09581 | Macrophage colony-stimulating factor 1 receptor | Csf1r | 23.56616211 | 23.50857353 | 23.45437813 | 23.76870537 | 23.75989532 | 23.83393097 |
| **+** | 2.186704629 | -0.274120967 | Q923D2;E9PZC3;E9PZC4;A0A0U1RPU7 | Flavin reductase (NADPH) | Blvrb | 27.14413834 | 27.13339806 | 27.08800507 | 27.3036499 | 27.40973091 | 27.47452354 |
| **+** | 2.761320689 | -0.271571477 | Q8BL66;A0A1W2P7A6 | Early endosome antigen 1 | Eea1 | 23.70693588 | 23.76951218 | 23.82143211 | 24.02529716 | 24.06784248 | 24.01945496 |
| **+** | 1.529270739 | -0.265165329 | Q80SZ7 | Guanine nucleotide-binding protein G(I)/G(S)/G(O) subunit gamma-5 | Gng5 | 23.43872833 | 23.57669449 | 23.43224335 | NaN | 23.70788383 | 23.7875576 |
| **+** | 1.565596469 | -0.263864517 | Q3TWW8;A0A0A6YXX6 | Serine/arginine-rich splicing factor 6 | Srsf6 | 24.26585007 | 24.39309692 | 24.2382679 | 24.68511963 | 24.4980793 | 24.50560951 |
| **+** | 1.969247031 | -0.262559255 | Q91V12;E9PYH2;A0A0E2W844 | Cytosolic acyl coenzyme A thioester hydrolase | Acot7 | 24.62304688 | 24.64289856 | 24.63295174 | 24.88171005 | 24.80282021 | 25.00204468 |
| **+** | 2.919637048 | -0.257811864 | A0A087WR50;A0A087WS56;Q3UHL6;P11276;A0A087WSN6;B7ZNJ1;B9EHT6;Q4KL80;A0A087WS99;A0A087WSU6;A0A087WQE0;A0A087WQW8 | Fibronectin;Anastellin | Fn1 | 25.23764801 | 25.2088089 | 25.24256325 | 25.42918015 | 25.52563858 | 25.50763702 |
| **+** | 3.732522201 | -0.257564545 | Q3U7R1;A0A1W2P784 | Extended synaptotagmin-1 | Esyt1 | 25.78386688 | 25.76948738 | 25.77339172 | 26.02995872 | 26.06735039 | 26.00213051 |
| **+** | 1.805086999 | -0.252229055 | O55222;D3YZA5;A0A1B0GRF6;A0A1B0GR42 | Integrin-linked protein kinase | Ilk | 23.57899857 | 23.68024445 | 23.59730148 | 23.96613693 | 23.77846336 | 23.86863136 |
| **+** | 2.176169343 | -0.252170563 | P68404;Q4VA93;P20444;Q3TQ39;Q2NKI4;P63318 | Protein kinase C beta type | Prkcb | 22.59559631 | 22.57223511 | 22.72525597 | 22.87274933 | 22.90479469 | 22.87205505 |
| **+** | 2.125438341 | -0.250703812 | Q62093 | Serine/arginine-rich splicing factor 2 | Srsf2 | 25.08362961 | 25.15969467 | 25.18997383 | 25.45732498 | 25.32354164 | 25.40454292 |
| **+** | 1.713263174 | -0.249677658 | P46978;D3YZN5 | Dolichyl-diphosphooligosaccharide--protein glycosyltransferase subunit STT3A | Stt3a | 24.53011322 | 24.63561058 | 24.73840904 | 24.83137321 | 24.90094948 | 24.92084312 |
| **+** | 2.701980218 | -0.248502731 | Q8VDW0;D6RHT5 | ATP-dependent RNA helicase DDX39A | Ddx39a | 24.51430511 | 24.55367851 | 24.62839699 | 24.80577469 | 24.80469322 | 24.8314209 |
| **+** | 4.255697806 | -0.246805827 | Q8K1B8;A0A494B9W3;A0A494BBJ8;A0A494B9F7 | Fermitin family homolog 3 | Fermt3 | 25.68431664 | 25.64921761 | 25.65899849 | 25.8959446 | 25.92648697 | 25.91051865 |
| **+** | 2.834543867 | -0.246157964 | P12970 | 60S ribosomal protein L7a | Rpl7a | 26.36327553 | 26.40431595 | 26.35004616 | 26.64595222 | 26.56462097 | 26.64553833 |
| **+** | 2.805111133 | -0.2457002 | A0A1C7CYV0;Q99K51;B1AX58 | Plastin-3 | Pls3 | 24.23366737 | 24.28573799 | 24.21785927 | 24.44215012 | 24.51340103 | 24.51881409 |
| **+** | 2.770883164 | -0.241140366 | O35405 | Phospholipase D3 | Pld3 | 26.53418541 | 26.58075333 | 26.61848831 | 26.80230331 | 26.79389572 | 26.86064911 |
| **+** | 2.159664302 | -0.237661362 | D3Z0B9;A0A1B0GSU0;Q571I9 | Aldehyde dehydrogenase family 16 member A1 | Aldh16a1 | 23.55964279 | 23.60523796 | 23.51026535 | 23.86844254 | 23.77705956 | 23.7426281 |
| **+** | 1.809470191 | -0.234443665 | A0A0J9YUZ4;P63158;A0A0J9YUD8;D3YZ18;D3YVC6 | High mobility group protein B1 | Hmgb1 | 25.98187447 | 25.95426941 | 25.80919075 | 26.10775185 | 26.18268204 | 26.15823174 |
| **+** | 2.011080657 | -0.232522329 | A0A5F8MPP7;A2AAY5 | SH3 and PX domain-containing protein 2B | Sh3pxd2b | 23.94467354 | 23.90288162 | 23.8094101 | 24.13014221 | 24.16353798 | 24.06085205 |
| **+** | 2.751427952 | -0.226931254 | P19253;A0A1B0GSQ6;A0A1B0GS68;A0A1B0GSB2;A0A1B0GTA1;A0A1B0GQW6;A0A1B0GSC2;A0A1B0GSF0;A0A1B0GSL5;A0A1B0GRH1;A0A1B0GT00;A0A1B0GSJ2 | 60S ribosomal protein L13a | Rpl13a | 25.87485695 | 25.88632393 | 25.8702774 | 26.04603958 | 26.11824799 | 26.14796448 |
| **+** | 2.426474219 | -0.221498489 | Q8VDN2 | Sodium/potassium-transporting ATPase subunit alpha-1 | Atp1a1 | 25.81406403 | 25.89654541 | 25.85250473 | 26.06993294 | 26.0310955 | 26.12658119 |
| **+** | 2.293533066 | -0.218938828 | O70145;A0A087WPH0 | Neutrophil cytosol factor 2 | Ncf2 | 24.98997116 | 24.90985489 | 24.87895393 | 25.18623734 | 25.13402557 | 25.11533356 |
| **+** | 2.515500597 | -0.211110433 | P62281;A0A1B0GRR3;A0A1B0GSE8 | 40S ribosomal protein S11 | Rps11 | 26.1838932 | 26.2872715 | 26.27737236 | 26.45974922 | 26.46312141 | 26.45899773 |
| **+** | 3.647789901 | -0.205100377 | E9PUA7;D3Z637;F8WHQ1;Q62393;D3Z125;D3Z7X7;D3Z2U2 | Tumor protein D52 | Tpd52 | 25.14199638 | 25.17066193 | 25.15826988 | 25.38010597 | 25.37145805 | 25.3346653 |
| **+** | 5.406795347 | -0.20501709 | Q9JMH6;A0A1W2P6U1;Q9D8I4;A0A0U1RPS1;A0A0U1RPC6;A0A0M3HEP9 | Thioredoxin reductase 1, cytoplasmic | Txnrd1 | 24.90219307 | 24.88943672 | 24.88874054 | 25.09579086 | 25.10609436 | 25.09353638 |
| **+** | 3.468692188 | -0.188719432 | Q9D0E1;B8JK33;B8JK32;F6W322;A0A3Q4EH91;F7C9U3;A0A3Q4L2X3 | Heterogeneous nuclear ribonucleoprotein M | Hnrnpm | 25.16649055 | 25.16188622 | 25.17702675 | 25.38304329 | 25.36048126 | 25.32803726 |
| **+** | 3.542435819 | 0.176305771 | Q9Z1G3;A0A2I3BPD0;A0A2I3BQD9 | V-type proton ATPase subunit C 1 | Atp6v1c1 | 25.32199478 | 25.34658432 | 25.37129211 | 25.17866135 | 25.165802 | 25.16649055 |
| **+** | 4.160718472 | 0.190630595 | Q99LB4;D3YTL5;P24452;D3YZN3;D3YU77;D3Z4K5;D3Z014 | Macrophage-capping protein | Capg | 29.21613884 | 29.21440125 | 29.18432808 | 29.01299858 | 29.00771523 | 29.02226257 |
| **+** | 4.073394985 | 0.200817108 | Q8C253;P16110 | Galectin;Galectin-3 | Lgals3 | 31.47271347 | 31.48687744 | 31.47566414 | 31.28928566 | 31.28911972 | 31.25439835 |
| **+** | 2.695062397 | 0.202342351 | Q9Z2U0;A0A338P7D7;Q9CWH6;B7ZMS4;A0A3Q4L361;A0A3Q4EHS3;A0A3Q4EG42 | Proteasome subunit alpha type-7 | Psma7 | 25.51068878 | 25.53812981 | 25.52525139 | 25.35518074 | 25.34336853 | 25.26849365 |
| **+** | 2.270439104 | 0.21125857 | P21981;G3UXE8;Q9D7I9 | Protein-glutamine gamma-glutamyltransferase 2 | Tgm2 | 27.81292725 | 27.79327583 | 27.73872948 | 27.60681915 | 27.59654999 | 27.5077877 |
| **+** | 2.037356297 | 0.217917124 | P61164;A0A494BB86;A0A494BAH0 | Alpha-centractin | Actr1a | 25.38136101 | 25.28019142 | 25.34820557 | 25.18336296 | 25.11076164 | 25.06188202 |
| **+** | 2.349146613 | 0.21806399 | Q6NZD2;Q9WV80;D3YWH1 | Sorting nexin-1 | Snx1 | 25.10896873 | 25.13703918 | 25.16633797 | 24.85148048 | 24.95184898 | 24.95482445 |
| **+** | 3.168253205 | 0.21814092 | P09103;E9Q8G8 | Protein disulfide-isomerase | P4hb | 27.58706284 | 27.60448837 | 27.58827972 | 27.34612656 | 27.41876602 | 27.36051559 |
| **+** | 2.780787078 | 0.21870931 | Q91YQ5;A0A0N4SUJ8 | Dolichyl-diphosphooligosaccharide--protein glycosyltransferase subunit 1 | Rpn1 | 25.72801018 | 25.75849915 | 25.82388687 | 25.55036545 | 25.54085159 | 25.56305122 |
| **+** | 2.470230551 | 0.21939532 | O35855;O88374;A0A1B0GX27;A0A1B0GST1;A0A1B0GQY4 | Branched-chain-amino-acid aminotransferase, mitochondrial;Branched-chain-amino-acid aminotransferase | Bcat2 | 24.89661407 | 24.95402527 | 24.91729736 | 24.72228813 | 24.64267731 | 24.74478531 |
| **+** | 2.919245471 | 0.226636887 | Q9Z0J0 | Epididymal secretory protein E1 | Npc2 | 27.72657013 | 27.769701 | 27.68779182 | 27.47336197 | 27.52020836 | 27.51058197 |
| **+** | 2.372968481 | 0.228221893 | P10605 | Cathepsin B;Cathepsin B light chain;Cathepsin B heavy chain | Ctsb | 29.43290901 | 29.43123436 | 29.43874931 | 29.13268852 | 29.26508141 | 29.22045708 |
| **+** | 1.927496684 | 0.228635152 | P48962;Q3V132 | ADP/ATP translocase 1 | Slc25a4 | 25.66052246 | 25.65717316 | 25.58098221 | 25.44426918 | 25.45444107 | 25.31406212 |
| **+** | 3.162827908 | 0.229353587 | Q91VA7;A0A668KL51;V9GXV0 | Isocitrate dehydrogenase [NAD] subunit, mitochondrial | Idh3b | 24.04693413 | 24.08934021 | 24.06529617 | 23.81850815 | 23.87942123 | 23.81558037 |
| **+** | 2.898598617 | 0.231671651 | P84084 | ADP-ribosylation factor 5 | Arf5 | 24.86107635 | 24.91305542 | 24.95828056 | 24.68992424 | 24.67271233 | 24.67476082 |
| **+** | 3.786892274 | 0.231862386 | Q7TPR4;A1BN54 | Alpha-actinin-1 | Actn1 | 26.73853683 | 26.72741318 | 26.72338295 | 26.47826195 | 26.53021622 | 26.48526764 |
| **+** | 2.157521521 | 0.232067108 | Q9JHF5;F6XRE6;F6ZFB8;A0A494B9E3 | V-type proton ATPase subunit a | Tcirg1 | 25.60181427 | 25.57539558 | 25.65058708 | 25.45082664 | 25.36643028 | 25.31433868 |
| **+** | 2.387167685 | 0.233665466 | Q91VR5;A0A1Y7VM48 | ATP-dependent RNA helicase DDX1 | Ddx1 | 23.55403137 | 23.53255272 | 23.5480442 | 23.35393524 | 23.34638023 | 23.23331642 |
| **+** | 1.88332955 | 0.235040029 | P70349;B0R1E3 | Histidine triad nucleotide-binding protein 1 | Hint1 | 27.36444473 | 27.51080894 | 27.51803398 | 27.27568626 | 27.21131897 | 27.20116234 |
| **+** | 2.293688928 | 0.235190074 | Q60766 | Immunity-related GTPase family M protein 1 | Irgm1 | 23.39662933 | 23.33456421 | 23.26785278 | 23.10455132 | 23.12858391 | 23.06034088 |
| **+** | 2.021611762 | 0.236233393 | Q8BK64;A0A1Y7VM19 | Activator of 90 kDa heat shock protein ATPase homolog 1 | Ahsa1 | 24.66063118 | 24.6798687 | 24.62683868 | 24.41680145 | 24.33756065 | 24.50427628 |
| **+** | 1.799653275 | 0.23770841 | A0A0A0MQ90;P97352 | Protein S100-A13 | S100a13 | 24.5836544 | 24.66101265 | 24.75404358 | 24.36573029 | 24.47580719 | 24.44404793 |
| **+** | 2.26026572 | 0.237973531 | Q91XH6;O88384;F6UHS3;E0CYE5 | Vesicle transport through interaction with t-SNAREs homolog 1B | Vti1b | 23.81626511 | 23.84594154 | 23.74139404 | 23.51050758 | 23.61649895 | 23.56267357 |
| **+** | 2.75171016 | 0.238159815 | Q9CZ13;A0A0A6YW82;A0A0A6YWX6;A0A0A6YVZ0 | Cytochrome b-c1 complex subunit 1, mitochondrial | Uqcrc1 | 25.14183998 | 25.20717239 | 25.15313721 | 24.92456436 | 24.97473717 | 24.88836861 |
| **+** | 1.895680532 | 0.238933563 | Q8R2Y8 | Peptidyl-tRNA hydrolase 2, mitochondrial | Ptrh2 | 22.7080307 | 22.77553177 | 22.67217255 | 22.39869118 | 22.4798317 | 22.56041145 |
| **+** | 2.642784195 | 0.239014943 | Q9DBP5;A0A0R4J093 | UMP-CMP kinase | Cmpk1 | 25.91104507 | 25.92938042 | 25.89702988 | 25.63666153 | 25.64402771 | 25.7397213 |
| **+** | 2.881226337 | 0.239639282 | Q99JY9;A0A087WRA1;A0A087WQ14;A0A087WP86;A0A087WS98;Q641P0;A0A087WQ83;A0A087WPR6 | Actin-related protein 3 | Actr3 | 27.30452156 | 27.35892296 | 27.31337166 | 27.12159157 | 27.09778214 | 27.03852463 |
| **+** | 1.91174383 | 0.241170247 | Q8JZQ2;Q920A7 | AFG3-like protein 2 | Afg3l2 | 23.18227196 | 23.31547928 | 23.28785324 | 23.04899788 | 22.94554901 | 23.06754684 |
| **+** | 2.888314268 | 0.243556341 | Q9CVB6;D3YXG6;A0A087WRT2 | Actin-related protein 2/3 complex subunit 2 | Arpc2 | 27.00260162 | 26.91627121 | 26.962677 | 26.72858238 | 26.73905182 | 26.68324661 |
| **+** | 3.984845943 | 0.244422913 | O09159 | Lysosomal alpha-mannosidase | Man2b1 | 26.41878128 | 26.39024544 | 26.42438316 | 26.14330101 | 26.1813736 | 26.17546654 |
| **+** | 2.361601431 | 0.246905645 | Q08857;A0A0G2JFB7 | Platelet glycoprotein 4 | Cd36 | 27.15141487 | 27.24090767 | 27.25421715 | 26.92036629 | 26.96961021 | 27.01584625 |
| **+** | 2.152952145 | 0.247463226 | P26443;F7CFA5 | Glutamate dehydrogenase 1, mitochondrial | Glud1 | 26.89700699 | 26.94960213 | 26.96102142 | 26.6666851 | 26.77391815 | 26.6246376 |
| **+** | 3.185632401 | 0.247861862 | P97449;A0A0U1RNS3 | Aminopeptidase N | Anpep | 28.27905655 | 28.23817635 | 28.3064785 | 28.02411461 | 27.99964905 | 28.05636215 |
| **+** | 2.269171478 | 0.251800537 | Q9CRD0;A0A0J9YUB6;A0A0J9YUK7;A0A0J9YTV6;A0A0J9YTV7 | OCIA domain-containing protein 1 | Ociad1 | 23.5447464 | 23.53278923 | 23.54933739 | 23.33606339 | 23.19920921 | 23.33619881 |
| **+** | 2.124282628 | 0.251911163 | Q9DB27;Q9CQ21 | Malignant T-cell-amplified sequence 1 | Mcts1 | 22.98032761 | 23.09056854 | 23.05722046 | 22.7218399 | 22.79586411 | 22.85467911 |
| **+** | 2.331372288 | 0.252082189 | Q9DCX2;B1ASE2 | ATP synthase subunit d, mitochondrial | Atp5h | 25.94920158 | 25.85519409 | 25.86180878 | 25.62878609 | 25.5853157 | 25.69585609 |
| **+** | 1.628688108 | 0.253243128 | Q99PV0;B7ZC27 | Pre-mRNA-processing-splicing factor 8 | Prpf8 | 23.05639267 | 23.22351074 | 23.05337906 | 22.94028854 | 22.84059334 | 22.7926712 |
| **+** | 2.761899516 | 0.254151662 | Q505F5;E9PV22;F6Z4L9;F6YT33 | Leucine-rich repeat-containing protein 47 | Lrrc47 | 23.29656029 | 23.36212158 | 23.3899498 | 23.07316399 | 23.13509941 | 23.07791328 |
| **+** | 3.175192675 | 0.256336212 | P97807;H3BKG7 | Fumarate hydratase, mitochondrial | Fh | 24.60438919 | 24.66752434 | 24.60772324 | 24.40093422 | 24.36846352 | 24.34123039 |
| **+** | 2.767840855 | 0.256616592 | Q99KQ4 | Nicotinamide phosphoribosyltransferase | Nampt | 24.44120026 | 24.45982742 | 24.3760376 | 24.12384224 | 24.19940376 | 24.1839695 |
| **+** | 2.65254105 | 0.25803566 | Q99LC5 | Electron transfer flavoprotein subunit alpha, mitochondrial | Etfa | 25.44654274 | 25.48741531 | 25.44944191 | 25.26395416 | 25.14417648 | 25.20116234 |
| **+** | 2.683878796 | 0.259909312 | Q80WQ2;A0A1D5RLY2;A0A1D5RLN5 | Protein VAC14 homolog | Vac14 | 23.51833344 | 23.46288681 | 23.44783592 | 23.1799221 | 23.27511787 | 23.19428825 |
| **+** | 2.910728946 | 0.259983063 | Q8K411 | Presequence protease, mitochondrial | Pitrm1 | 23.43326187 | 23.50917816 | 23.51917267 | 23.19385147 | 23.24205399 | 23.24575806 |
| **+** | 2.24273303 | 0.260985692 | Q60930;A0A286YCR8;D3YZT5;D3YUN8 | Voltage-dependent anion-selective channel protein 2 | Vdac2 | 27.89394379 | 27.88075256 | 27.98527718 | 27.59063721 | 27.70990944 | 27.6764698 |
| **+** | 1.655918314 | 0.26373291 | O54734 | Dolichyl-diphosphooligosaccharide--protein glycosyltransferase 48 kDa subunit | Ddost | 25.32006836 | 25.40308189 | 25.45710564 | 25.0122261 | 25.16215515 | 25.2146759 |
| **+** | 4.8814696 | 0.266957601 | Q6ZWZ4;P47964 | 60S ribosomal protein L36 | Rpl36 | 26.49832344 | 26.48589706 | 26.5206871 | 26.23306465 | 26.23775864 | 26.23321152 |
| **+** | 3.53754035 | 0.267937342 | Q8BFR4;A0A1W2P8D3;A0A1W2P6W9 | N-acetylglucosamine-6-sulfatase | Gns | 26.01994324 | 26.04660225 | 26.02997971 | 25.74121475 | 25.80663681 | 25.7448616 |
| **+** | 1.617845645 | 0.270103455 | Q8BP48;A0A0G2JF71;A0A0G2JFL1 | Methionine aminopeptidase 1 | Metap1 | 22.66925621 | 22.75056267 | 22.79649734 | 22.45244408 | 22.59149361 | 22.36206818 |
| **+** | 1.767006354 | 0.270202637 | Q8R0H9;A0A2R8VI72 | ADP-ribosylation factor-binding protein GGA1 | Gga1 | 23.14836502 | 22.94742203 | 23.08673477 | 22.7487011 | 22.85918045 | 22.76403236 |
| **+** | 1.51329121 | 0.273089727 | Z4YJU8;A0A6I8MWY5;E9PUQ5;A0A6I8MX07;Q921M4;A2AN45;A2AN48;A2AN46 | Golgin subfamily A member 2 | Golga2 | 22.5737381 | 22.35622215 | 22.40418625 | 22.2189827 | 22.06966209 | 22.22623253 |
| **+** | 1.658644704 | 0.275047938 | P56542;D3Z512;D3Z5U3 | Deoxyribonuclease-2-alpha | Dnase2;Dnase2a | 24.39473343 | 24.48388481 | 24.34975815 | 24.23805046 | 24.01622772 | 24.14895439 |
| **+** | 2.373160257 | 0.276802063 | Q8BMS1 | Trifunctional enzyme subunit alpha, mitochondrial;Long-chain enoyl-CoA hydratase;Long chain 3-hydroxyacyl-CoA dehydrogenase | Hadha | 25.32230568 | 25.33063698 | 25.29551125 | 25.13033867 | 25.00607491 | 24.98163414 |
| **+** | 3.299911396 | 0.277208964 | Q9QZE5;Q7TNQ1 | Coatomer subunit gamma-1 | Copg1 | 24.30201149 | 24.23513031 | 24.27994347 | 23.97753525 | 23.97604942 | 24.0318737 |
| **+** | 1.568823129 | 0.278811773 | Q8BYW1 | Rho GTPase-activating protein 25 | Arhgap25 | 23.42329407 | 23.59411621 | 23.52420425 | 23.18452835 | 23.36399269 | 23.15665817 |
| **+** | 2.338642436 | 0.279612223 | A0A0R4J138;P50429 | Arylsulfatase B | Arsb | 24.24176407 | 24.13922691 | 24.11545181 | 23.93992996 | 23.87979507 | 23.83788109 |
| **+** | 1.523062171 | 0.279789607 | A0A0R3P9C8;Q9DC69 | NADH dehydrogenase [ubiquinone] 1 alpha subcomplex subunit 9, mitochondrial | Ndufa9 | 23.10828972 | 23.22561646 | 23.20357704 | 23.00961494 | 22.75158501 | 22.93691444 |
| **+** | 1.786764184 | 0.282129288 | P61211;F8WIB1;E9Q006 | ADP-ribosylation factor-like protein 1 | Arl1 | 24.13335991 | 23.98146057 | 24.15452766 | 23.76819992 | 23.8978138 | 23.75694656 |
| **+** | 1.522874819 | 0.284274419 | Q78IK4;B1AV14 | MICOS complex subunit Mic27 | Apool | 22.96126938 | 23.08846664 | 22.84450531 | 22.69466209 | 22.58820152 | 22.75855446 |
| **+** | 1.432769163 | 0.285439809 | Q99KF1;A0A286YDS5 | Transmembrane emp24 domain-containing protein 9 | Tmed9 | 25.56656837 | 25.61453438 | 25.51842308 | 25.39365387 | 25.34285927 | 25.10669327 |
| **+** | 1.789556002 | 0.286911647 | Q5SQX6;F6QD74 | Cytoplasmic FMR1-interacting protein 2 | Cyfip2 | 23.20495033 | 23.35997963 | 23.19182014 | 22.88544846 | 22.96100426 | 23.04956245 |
| **+** | 2.172143557 | 0.287986755 | Q9CZU6;Q80X68 | Citrate synthase, mitochondrial;Citrate synthase | Cs;Csl | 26.64938164 | 26.60837173 | 26.62756157 | 26.39568138 | 26.2312336 | 26.3944397 |
| **+** | 5.033754756 | 0.289269765 | P50516;D3Z1B9;D3YWH3;D3YZ23 | V-type proton ATPase catalytic subunit A | Atp6v1a | 27.41053963 | 27.39843941 | 27.42878151 | 27.12996483 | 27.1268177 | 27.11316872 |
| **+** | 1.614396584 | 0.289849599 | Q99L45;E0CXJ3 | Eukaryotic translation initiation factor 2 subunit 2 | Eif2s2 | 25.73440742 | 25.79704285 | 25.82213593 | 25.51246834 | 25.35168076 | 25.61988831 |
| **+** | 1.730161525 | 0.291100184 | P57716 | Nicastrin | Ncstn | 24.33149147 | 24.48142242 | 24.24901772 | 24.03263092 | 24.02546692 | 24.13053322 |
| **+** | 1.653483055 | 0.291662852 | Q07417 | Short-chain specific acyl-CoA dehydrogenase, mitochondrial | Acads | 24.34529877 | 24.37158966 | 24.46973419 | 24.17835617 | 24.17119598 | 23.96208191 |
| **+** | 1.570610304 | 0.296069463 | P62855 | 40S ribosomal protein S26 | Rps26 | 27.16240501 | 27.10269547 | 26.88320351 | 26.76572418 | 26.77555275 | 26.71881866 |
| **+** | 2.023862373 | 0.298074722 | Q91VH6;A0A3B2W7C9;A0A3F2YNL8;A0A3B2W4I2;A0A3B2WCC1;A0A3B2W4G7 | Protein MEMO1 | Memo1 | 23.66253471 | 23.85908508 | 23.81714249 | 23.46513176 | 23.45412636 | 23.52528 |
| **+** | 1.801586905 | 0.299818039 | Q9CPW4;Q3UA72 | Actin-related protein 2/3 complex subunit 5 | Arpc5 | 27.24508667 | 27.40859795 | 27.35640717 | 26.9234314 | 27.09717941 | 27.09002686 |
| **+** | 1.582147709 | 0.302082698 | Q9CQ65 | S-methyl-5-thioadenosine phosphorylase | Mtap | 24.21852303 | 24.27880859 | 24.42930794 | 24.03178978 | 23.89031792 | 24.09828377 |
| **+** | 1.771551184 | 0.302667618 | Q8BGH2 | Sorting and assembly machinery component 50 homolog | Samm50 | 23.31603241 | 23.38350487 | 23.46737289 | 23.20638275 | 23.05968094 | 22.99284363 |
| **+** | 1.355922583 | 0.30413119 | B7ZNL2;Q78ZA7;A0A140LJB5;A0A140LJ37;A0A140LI78 | Nucleosome assembly protein 1-like 4 | Nap1l4 | 24.21252441 | 24.27326965 | 24.39800072 | 23.85633278 | 23.95491219 | 24.16015625 |
| **+** | 2.558065677 | 0.304318746 | Q8BIG7;H3BJ37 | Catechol O-methyltransferase domain-containing protein 1 | Comtd1 | 24.5434494 | 24.58812141 | 24.59115028 | 24.32443619 | 24.18366623 | 24.30166245 |
| **+** | 1.674394402 | 0.305323919 | Q3TCN2 | Putative phospholipase B-like 2;Putative phospholipase B-like 2 28 kDa form;Putative phospholipase B-like 2 40 kDa form;Putative phospholipase B-like 2 15 kDa form | Plbd2 | 24.10325623 | 24.15707397 | 24.23651695 | 23.8111763 | 23.76516724 | 24.00453186 |
| **+** | 2.634220187 | 0.306891123 | P70296;D3Z1V4;D6RHS6;E9QLE5;Q8VIN1 | Phosphatidylethanolamine-binding protein 1;Hippocampal cholinergic neurostimulating peptide | Pebp1 | 27.19966698 | 27.06845665 | 27.16557121 | 26.81298828 | 26.87923431 | 26.82079887 |
| **+** | 1.521766027 | 0.308048884 | A0A0U1RPM2;P16879;A0A0U1RNL3;A0A0U1RPB9;A0A0U1RQ80 | Tyrosine-protein kinase Fes/Fps | Fes | 22.64671516 | 22.50149727 | 22.55356216 | 22.3318882 | 22.09306908 | 22.35267067 |
| **+** | 2.37309841 | 0.308120728 | Q9DBG5;A0A3B2WCW2 | Perilipin-3 | Plin3 | 25.69500732 | 25.56313896 | 25.5421505 | 25.31163979 | 25.31592941 | 25.2483654 |
| **+** | 3.356826159 | 0.30930837 | P08249;A0A0G2JF23;A0A0G2JGY4 | Malate dehydrogenase, mitochondrial | Mdh2 | 27.37567329 | 27.43364525 | 27.43412209 | 27.14131355 | 27.06661034 | 27.10759163 |
| **+** | 1.447372164 | 0.310280482 | Q9CQM5 | Thioredoxin domain-containing protein 17 | Txndc17 | 25.74558067 | 25.57951546 | 25.91542816 | 25.43647575 | 25.39718437 | 25.47602272 |
| **+** | 1.741173273 | 0.311139425 | Q9JKV1;A0A0A6YVU8;D3YUD8 | Proteasomal ubiquitin receptor ADRM1 | Adrm1;Gm9774 | 24.02047348 | 24.00092697 | 23.866745 | 23.52898026 | 23.67798805 | 23.74775887 |
| **+** | 2.388816321 | 0.311745326 | Q99JX4;A2A702;A2A701 | Eukaryotic translation initiation factor 3 subunit M | Eif3m | 24.33312988 | 24.32092857 | 24.22464561 | 23.93490028 | 23.94779778 | 24.06077003 |
| **+** | 2.751477496 | 0.312760035 | Q91XV3 | Brain acid soluble protein 1 | Basp1 | 27.56128883 | 27.53430367 | 27.5042305 | 27.23608017 | 27.27870178 | 27.14676094 |
| **+** | 1.975591703 | 0.313280741 | P62331 | ADP-ribosylation factor 6 | Arf6 | 24.84143829 | 24.64267731 | 24.66155624 | 24.34732819 | 24.43790436 | 24.42059708 |
| **+** | 1.810427105 | 0.313536644 | Q9D338 | 39S ribosomal protein L19, mitochondrial | Mrpl19 | 21.84987831 | 21.76076889 | 21.87975883 | 21.45938873 | 21.57380867 | NaN |
| **+** | 1.384170064 | 0.314996084 | P05132 | cAMP-dependent protein kinase catalytic subunit alpha | Prkaca | 22.79014587 | 22.62415123 | 22.56746292 | 22.50678444 | 22.29574966 | 22.23423767 |
| **+** | 1.333110252 | 0.315378825 | P51410;A0A0G2JES3;A0A140T8T4;D3Z629;D3YZT0;A0A0G2JFQ3 | 60S ribosomal protein L9 | Rpl9 | 26.25970459 | 26.40944099 | 26.40989304 | 25.86924171 | 26.05258369 | 26.21107674 |
| **+** | 3.366974632 | 0.316626867 | P10107;A0A494BBD8 | Annexin A1 | Anxa1 | 28.70437813 | 28.70305824 | 28.7461834 | 28.35497856 | 28.444664 | 28.4040966 |
| **+** | 1.591608175 | 0.316663106 | Q9EQU5;A2BE93;A2BE92 | Protein SET | Set | 25.76283836 | 25.55607986 | 25.77258492 | 25.27976608 | 25.38152695 | 25.48022079 |
| **+** | 2.12664503 | 0.319455465 | A0A0J9YUI1;P61087;A0A0J9YU07;Q3V3R8;A0A0J9YUR9;D3Z4U3;F8WIC2 | Ubiquitin-conjugating enzyme E2 K | Ube2k | 24.26226997 | 24.16300011 | 24.12786102 | 23.87201691 | 23.77575302 | 23.94699478 |
| **+** | 1.447211741 | 0.319527944 | Q6P4T2 | U5 small nuclear ribonucleoprotein 200 kDa helicase | Snrnp200 | 23.16430664 | 23.26656532 | 23.29740143 | 22.78777695 | 22.87712097 | 23.10479164 |
| **+** | 3.3358141 | 0.320185343 | A2ACG7;Q9DBG6 | Dolichyl-diphosphooligosaccharide--protein glycosyltransferase subunit 2 | Rpn2 | 25.97694588 | 25.9589653 | 25.9510479 | 25.58651924 | 25.65313339 | 25.68675041 |
| **+** | 3.990285722 | 0.32384491 | Q8BFR5;A0A0U1RNQ6;A0A0U1RPC4 | Elongation factor Tu, mitochondrial | Tufm | 25.82951164 | 25.8617382 | 25.81836319 | 25.54064369 | 25.51367378 | 25.48376083 |
| **+** | 1.898648758 | 0.324420293 | Q9D880 | Mitochondrial import inner membrane translocase subunit TIM50 | Timm50 | 23.67734337 | 23.5976429 | 23.58371162 | 23.43237114 | 23.24622154 | 23.20684433 |
| **+** | 2.405691724 | 0.324546814 | Q9R0X4;Q32MW3 | Acyl-coenzyme A thioesterase 9, mitochondrial;Acyl-coenzyme A thioesterase 10, mitochondrial | Acot9;Acot10 | 23.55765724 | 23.69529915 | 23.614254 | 23.26026154 | 23.37132454 | 23.26198387 |
| **+** | 2.506016144 | 0.327085495 | A0A338P786;P68037;A0A338P7E5;A0A338P702 | Ubiquitin-conjugating enzyme E2 L3 | Ube2l3 | 26.10167313 | 26.19366264 | 26.21525002 | 25.77786064 | 25.84280396 | 25.9086647 |
| **+** | 3.585914216 | 0.327863057 | Q9WUU7 | Cathepsin Z | Ctsz | 27.04249573 | 26.96873093 | 27.05382729 | 26.69630814 | 26.69789886 | 26.68725777 |
| **+** | 4.048985709 | 0.328272502 | Q61171;D3Z4A4 | Peroxiredoxin-2 | Prdx2 | 27.58541679 | 27.63623238 | 27.60787773 | 27.25709915 | 27.30669403 | 27.28091621 |
| **+** | 1.480695805 | 0.33080705 | P53395 | Lipoamide acyltransferase component of branched-chain alpha-keto acid dehydrogenase complex, mitochondrial | Dbt | 21.92019844 | 22.1180687 | NaN | 21.66869354 | 21.63559914 | 21.76068687 |
| **+** | 2.885135112 | 0.331135432 | Q920A5 | Retinoid-inducible serine carboxypeptidase | Scpep1 | 26.10027122 | 25.97108078 | 26.06995392 | 25.70601273 | 25.74234581 | 25.69954109 |
| **+** | 1.87720831 | 0.33119901 | Q9D0K2;Q3UJQ9 | Succinyl-CoA:3-ketoacid coenzyme A transferase 1, mitochondrial;Succinyl-CoA:3-ketoacid-coenzyme A transferase | Oxct1 | 25.31710243 | 25.37947845 | 25.53836632 | 25.16180992 | 25.0578804 | 25.02165985 |
| **+** | 3.069639258 | 0.331883748 | Q60932 | Voltage-dependent anion-selective channel protein 1 | Vdac1 | 26.1825676 | 26.30127907 | 26.25889587 | 25.89896774 | 25.9405117 | 25.90761185 |
| **+** | 2.474418544 | 0.332342148 | P29416 | Beta-hexosaminidase subunit alpha | Hexa | 26.46032715 | 26.4792347 | 26.55449867 | 26.0802784 | 26.18495369 | 26.23180199 |
| **+** | 1.288116391 | 0.33253034 | P62869;A0A3B2WBM3 | Transcription elongation factor B polypeptide 2 | Tceb2 | 25.00710106 | 25.05395126 | 24.92610359 | 24.81313515 | 24.73954201 | 24.43688774 |
| **+** | 1.889152344 | 0.332807541 | Q9WU81 | Sugar phosphate exchanger 2 | Slc37a2 | 24.69805717 | 24.50488281 | 24.50802994 | 24.17378998 | 24.21549034 | 24.32326698 |
| **+** | 3.470189736 | 0.334012349 | Q99P91;Q8BVA0 | Transmembrane glycoprotein NMB | Gpnmb | 30.87019348 | 30.89417458 | 30.96023369 | 30.59227753 | 30.57776642 | 30.55252075 |
| **+** | 2.077657597 | 0.335863749 | Q9D2G2 | Dihydrolipoyllysine-residue succinyltransferase component of 2-oxoglutarate dehydrogenase complex, mitochondrial | Dlst | 24.60636902 | 24.64861488 | 24.51038742 | 24.35440636 | 24.24190903 | 24.16146469 |
| **+** | 3.302226765 | 0.337742488 | Q9CR62;Q5SX46;Q5SX48 | Mitochondrial 2-oxoglutarate/malate carrier protein | Slc25a11 | 25.48206902 | 25.58178711 | 25.49905396 | 25.19358826 | 25.16100311 | 25.19509125 |
| **+** | 1.351963717 | 0.33778286 | P0DOV2;P0DOV1;Q8CGE8 | Interferon-activable protein 204 | Ifi204 | 22.83564758 | 22.78466606 | 22.75015259 | NaN | 22.3235836 | 22.5811615 |
| **+** | 4.668119957 | 0.338176727 | Q03265;D3Z6F5;D6RJ16;A0A0E2WDP1 | ATP synthase subunit alpha, mitochondrial;ATP synthase subunit alpha | Atp5a1 | 27.81573868 | 27.81726456 | 27.85311127 | 27.48416138 | 27.5069561 | 27.48046684 |
| **+** | 3.469570611 | 0.339065552 | Q8CAQ8;E9Q800;E9QAY6;A0A1B0GX08;A0A0U1RQ14;E9PVS5 | MICOS complex subunit Mic60 | Immt | 24.88892555 | 24.90306664 | 24.830019 | 24.55385399 | 24.55531883 | 24.49564171 |
| **+** | 2.04127592 | 0.339410782 | Q9CPQ3;A0A2R8VHM4;A0A2R8VHJ4 | Mitochondrial import receptor subunit TOM22 homolog | Tomm22 | 24.20075226 | 24.11314774 | 24.31713676 | 23.79817963 | 23.87577057 | 23.93885422 |
| **+** | 1.340018819 | 0.342074712 | Q9WTL7 | Acyl-protein thioesterase 2 | Lypla2 | 24.5088768 | 24.39512634 | 24.29459953 | 24.12786102 | 23.85623932 | 24.1882782 |
| **+** | 1.862716385 | 0.343529383 | Q8C0E2 | Vacuolar protein sorting-associated protein 26B | Vps26b | 23.53290939 | 23.45022964 | 23.41854095 | 22.97557831 | 23.18558884 | 23.2099247 |
| **+** | 2.245091453 | 0.344204585 | P62307 | Small nuclear ribonucleoprotein F | Snrpf | 24.66367531 | 24.68062019 | 24.63344955 | 24.39027786 | 24.19159508 | 24.36325836 |
| **+** | 1.30564336 | 0.345054626 | Q9EQ80;D3Z0G0;E9PWJ6 | NIF3-like protein 1 | Nif3l1 | 23.02025223 | 23.15375519 | NaN | NaN | 22.69833374 | 22.78556442 |
| **+** | 1.647622066 | 0.345434189 | G3UZY2;P97493;G3UX99;A2A439 | Thioredoxin, mitochondrial | Txn2 | 24.04751778 | 24.16154099 | 24.22309875 | NaN | 23.73995399 | 23.85728264 |
| **+** | 1.440189737 | 0.345846812 | Q58A65 | C-Jun-amino-terminal kinase-interacting protein 4 | Spag9 | 22.73766518 | 23.00653648 | 22.70668221 | 22.58598137 | 22.43094254 | 22.39641953 |
| **+** | 1.368088217 | 0.346340179 | O54965;Q8C4F9;Q8CB78;A0A0G2JEM4;A0A0G2JGT7 | E3 ubiquitin-protein ligase RNF13 | Rnf13 | 25.93510246 | 25.65299606 | 25.83742523 | 25.322855 | 25.61464691 | 25.44900131 |
| **+** | 1.338709695 | 0.34702301 | E9PVG7;P97820;B7ZNR9;B2RUE8;A0A0A6YWR8;F8VPL5;A0A0A6YWM8;A0A0A6YW53;P83510;B9EKN8;E9PUL9;E0CY98;E0CXD6;E0CZF8;E0CZD7;B2RQ80;A0A0A6YWJ2;A0A0A6YVZ8;A0A0A6YXE5;A0A0A6YVR7 | Mitogen-activated protein kinase kinase kinase kinase 4 | Map4k4 | 21.51325798 | 21.8639698 | 21.57865334 | 21.39210129 | 21.32163048 | 21.20108032 |
| **+** | 2.007023732 | 0.347587585 | A0A0R4J0P1;Q9D7B6;D3YTT4;D6RDD5 | Isobutyryl-CoA dehydrogenase, mitochondrial | Acad8 | 23.00686073 | 22.86315727 | NaN | 22.59213257 | 22.55156898 | 22.6185627 |
| **+** | 2.608536978 | 0.349945068 | Q8VEB4;A0A1D5RLZ5 | Group XV phospholipase A2 | Pla2g15 | 24.00247383 | 23.91606712 | 23.888834 | 23.65916252 | 23.52850342 | 23.56987381 |
| **+** | 2.614295931 | 0.351811727 | P50518;A0A0N4SW07;A0A0N4SW34;A0A0N4SWA3;Q9D593 | V-type proton ATPase subunit E 1 | Atp6v1e1 | 26.38937569 | 26.36999512 | 26.37290192 | 26.12799835 | 25.98045921 | 25.96837997 |
| **+** | 2.029559014 | 0.353532791 | P13597 | Intercellular adhesion molecule 1 | Icam1 | 23.13053322 | 23.08589172 | 22.97564697 | 22.82908058 | 22.64092445 | 22.66146851 |
| **+** | 1.700127165 | 0.354735057 | A0A1W2P7X0;E9QMV2;Q4KML4 | Costars family protein ABRACL | Abracl | 25.05511093 | 25.04992867 | 25.04401779 | 24.50597382 | 24.79491234 | 24.78396606 |
| **+** | 1.750628995 | 0.355845133 | F7CUQ1;Q91WX5;O08734 | Bcl-2 homologous antagonist/killer | Bak1 | 23.57346153 | 23.73427963 | 23.6790638 | 23.29417801 | 23.17700386 | 23.44808769 |
| **+** | 3.458198339 | 0.356355667 | E9Q1W0;E9Q1T1;A0A0G2JGS4;Q6PHZ2;E9Q1V9;Q8CCM0;E9PXV3;F6RWZ9;F8WIS9;P11798;E9QAJ4;D6RDQ8;A0A286YCB8;A0A286YDL6;A0A286YDK9;F6WHR9 | Calcium/calmodulin-dependent protein kinase type II subunit delta | Camk2d | 24.68864441 | 24.59531212 | 24.64438438 | 24.29200363 | 24.31146622 | 24.25580406 |
| **+** | 2.261986934 | 0.356390635 | Q9QYJ0 | DnaJ homolog subfamily A member 2 | Dnaja2 | 24.15684319 | 24.21193123 | 24.10037041 | 23.70472145 | 23.90113258 | 23.79411888 |
| **+** | 1.803500135 | 0.360811869 | Q9DB73;G3UZG6;G3UXV8;G3UZ57 | NADH-cytochrome b5 reductase 1 | Cyb5r1 | 23.78974915 | 23.8752079 | 23.84009361 | 23.33128738 | 23.46276283 | 23.62856483 |
| **+** | 2.23220726 | 0.361666361 | P17047 | Lysosome-associated membrane glycoprotein 2 | Lamp2 | 27.02210426 | 26.93820381 | 26.8872757 | 26.69696999 | 26.52531052 | 26.54030418 |
| **+** | 1.393893028 | 0.362225215 | Q9CQE8 | UPF0568 protein C14orf166 homolog |  | 23.5641861 | 23.58943748 | 23.53100586 | 23.23842812 | 22.97470284 | 23.38482285 |
| **+** | 1.941637854 | 0.364320755 | Q8BIJ6;E9PWN2;E9PWN3 | Isoleucine--tRNA ligase, mitochondrial | Iars2 | 23.35850334 | 23.29921913 | 23.17951202 | 22.99832916 | 22.95516014 | 22.79078293 |
| **+** | 1.636273755 | 0.367023468 | Q922Q4;A2ABZ3;A2ABZ0;A2ABZ2;Q922W5 | Pyrroline-5-carboxylate reductase 2 | Pycr2 | 23.56523132 | 23.33510971 | 23.65250397 | 23.17276764 | 23.20450211 | 23.07450485 |
| **+** | 1.266138712 | 0.367324829 | A0A0R4J0V5;P08775 | DNA-directed RNA polymerase II subunit RPB1 | Polr2a | 21.10686874 | NaN | 21.27344131 | NaN | 20.79098129 | 20.85467911 |
| **+** | 1.631884757 | 0.367775599 | P63260;B1ATY1;G3UYG0;E9Q606 | Actin, cytoplasmic 2;Actin, cytoplasmic 2, N-terminally processed | Actg1 | 28.70001793 | 28.91581535 | 28.73843956 | 28.31786156 | 28.57231903 | 28.36076546 |
| **+** | 2.437633351 | 0.368015925 | Q5ND34;K4DI77;F6XD87 | WD repeat-containing protein 81 | Wdr81 | 22.79344368 | 22.70699883 | 22.82535744 | 22.47590637 | 22.43316078 | 22.31268501 |
| **+** | 1.993343396 | 0.370260874 | P59999;Q3TX55;E9PWA7 | Actin-related protein 2/3 complex subunit 4 | Arpc4 | 27.46762085 | 27.62999535 | 27.50960159 | 27.22195625 | 27.03684807 | 27.23763084 |
| **+** | 3.057314095 | 0.371733348 | Q9D051 | Pyruvate dehydrogenase E1 component subunit beta, mitochondrial | Pdhb | 25.07471657 | 25.02837944 | 25.11862373 | 24.75913429 | 24.70107651 | 24.6463089 |
| **+** | 5.521079698 | 0.373072306 | O35129;F6QPR1;F6Q8V7 | Prohibitin-2 | Phb2 | 25.77218246 | 25.79044533 | 25.77097321 | 25.41728592 | 25.39076996 | 25.4063282 |
| **+** | 4.056125999 | 0.373214086 | P62814;Q91YH6;A0A0U1RNU9 | V-type proton ATPase subunit B, brain isoform | Atp6v1b2 | 27.34460449 | 27.30791092 | 27.31440926 | 26.96002769 | 26.97749138 | 26.90976334 |
| **+** | 1.26055392 | 0.374265671 | Q6P9Q4 | FH1/FH2 domain-containing protein 1 | Fhod1 | 22.46617889 | 22.26238441 | 22.50045204 | 21.91155815 | 21.92361259 | 22.27104759 |
| **+** | 2.355189051 | 0.376686096 | Q60597;Z4YJV4;B2RXT3;E9Q7L0;Q5SVY0;Q5SVY1 | 2-oxoglutarate dehydrogenase, mitochondrial | Ogdh | 24.61161232 | 24.5086956 | 24.55062866 | 24.28157043 | 24.08156013 | 24.17774773 |
| **+** | 1.511361991 | 0.377658208 | Q6ZWQ5;Q3TGS7;Q3V2H3;O70493 | Sorting nexin-12 | Snx12 | 25.48821259 | 25.30830956 | 25.56502914 | 24.91218758 | 25.10824966 | 25.20813942 |
| **+** | 2.728044898 | 0.38290596 | D3Z5U5;Q9Z257;Q9JJX6;D3YYR5;Q9Z256 | P2X purinoceptor;P2X purinoceptor 4 | P2rx4 | 23.76698685 | 23.70956612 | 23.68795204 | 23.36265755 | 23.24816322 | 23.40496635 |
| **+** | 2.498829626 | 0.383809408 | D3YWT0;D3Z569;D3YTS1;Q9R0P6 | Signal peptidase complex catalytic subunit SEC11;Signal peptidase complex catalytic subunit SEC11A | Sec11a | 24.33360863 | 24.19038963 | 24.17881203 | 23.8179245 | 23.81411362 | 23.91934395 |
| **+** | 3.111473914 | 0.385516485 | Q9CPU4 | Microsomal glutathione S-transferase 3 | Mgst3 | 25.75411987 | 25.75964165 | 25.80407715 | 25.39933777 | 25.44729996 | 25.31465149 |
| **+** | 1.56782069 | 0.387613297 | P36423;F6SG79;Q3TRY5 | Thromboxane-A synthase | Tbxas1 | 23.80950928 | 23.58428383 | 23.75796509 | 23.39689064 | 23.14949799 | 23.44252968 |
| **+** | 2.086828157 | 0.390042623 | O35295 | Transcriptional activator protein Pur-beta | Purb | 24.44177055 | 24.3998909 | 24.22221375 | 23.98137283 | 23.88269043 | 24.02968407 |
| **+** | 1.136233373 | 0.391536077 | Q80ZJ1 | Ras-related protein Rap-2a | Rap2a | 23.08341026 | 22.96608353 | 23.06152725 | 22.56052971 | 22.42434502 | 22.95153809 |
| **+** | 1.480710765 | 0.394700368 | D3Z7P3;D3Z7P4;F7B327;F6U529;F6RDM4 | Glutaminase kidney isoform, mitochondrial | Gls | 25.21892929 | 24.96679688 | 25.0558548 | 24.54610252 | 24.87731743 | 24.63405991 |
| **+** | 1.407222417 | 0.395223618 | F6UND7;P08103 | Non-specific protein-tyrosine kinase;Tyrosine-protein kinase HCK | Hck | 23.07540321 | 23.25342369 | 23.08553505 | 22.88233566 | 22.50949287 | 22.83686256 |
| **+** | 2.4361525 | 0.395820618 | Q9WVA2;Q4FZG7 | Mitochondrial import inner membrane translocase subunit Tim8 A | Timm8a1 | 23.00971794 | 23.17424774 | 23.191082 | 22.72369576 | 22.78278732 | 22.68110275 |
| **+** | 4.526108856 | 0.397460302 | Q9DCW4;A0A0U1RNP5;A0A0N4SVE0;A0A0U1RNR3;A0A0U1RNK9;A0A0N4SWE9;A0A0U1RQB4 | Electron transfer flavoprotein subunit beta | Etfb | 25.11187553 | 25.09796333 | 25.12178802 | 24.7470932 | 24.68880463 | 24.70334816 |
| **+** | 3.148524306 | 0.397684733 | P51569;Q8BGZ6;A2BDV6 | Alpha-galactosidase A | Gla | 25.74632454 | 25.81315994 | 25.75003624 | 25.33650589 | 25.33527946 | 25.44468117 |
| **+** | 1.770653922 | 0.40110906 | Q61425 | Hydroxyacyl-coenzyme A dehydrogenase, mitochondrial | Hadh | 24.68554688 | 24.55637169 | 24.49753189 | 24.05713654 | 24.13578796 | 24.34319878 |
| **+** | 3.397374266 | 0.401750565 | P63038;D3Z2F2;D3Z7J9 | 60 kDa heat shock protein, mitochondrial | Hspd1 | 27.04916 | 27.11515427 | 27.16930389 | 26.68685722 | 26.71279716 | 26.72871208 |
| **+** | 1.832896225 | 0.402598699 | P84078;P61205 | ADP-ribosylation factor 1;ADP-ribosylation factor 3 | Arf1;Arf3 | 26.85055161 | 26.9304409 | 27.0487442 | 26.4616394 | 26.46134186 | 26.69895935 |
| **+** | 2.126168356 | 0.404184977 | P10922 | Histone H1.0;Histone H1.0, N-terminally processed | H1f0 | 24.50875473 | 24.52945709 | 24.30827522 | 24.06628227 | 24.09948921 | 23.96816063 |
| **+** | 3.041089255 | 0.407262166 | P17439;A0A0G2JDK2 | Glucosylceramidase | Gba | 25.61865807 | 25.49365807 | 25.57201576 | 25.16503525 | 25.1968174 | 25.10069275 |
| **+** | 1.537181229 | 0.407560349 | A0A494B9R1;Q62084;A0A494B933;A0A494BB30 | Protein phosphatase 1 regulatory subunit 14B | Ppp1r14b | 22.08063316 | 21.84310722 | 22.19781494 | 21.51026535 | 21.66414261 | 21.72446632 |
| **+** | 1.673511579 | 0.412850062 | Q91W86;A0A1L1SRH3;A0A1L1SQ62;A0A1L1SV27 | Vacuolar protein sorting-associated protein 11 homolog | Vps11 | 23.79114151 | 23.83595657 | 23.82522202 | 23.55987549 | 23.18863869 | 23.46525574 |
| **+** | 1.793217179 | 0.413687388 | A0A0N4SVK3;P62322 | U6 snRNA-associated Sm-like protein LSm5 | Lsm5 | 22.92134285 | 22.60921288 | 22.82361031 | 22.31561852 | 22.33349991 | 22.46398544 |
| **+** | 2.481580399 | 0.414756139 | Q9WTP6;F7BP55 | Adenylate kinase 2, mitochondrial;Adenylate kinase 2, mitochondrial, N-terminally processed | Ak2 | 24.67696762 | 24.56389427 | 24.57184219 | 24.08237267 | 24.26613617 | 24.21992683 |
| **+** | 3.32731542 | 0.414827983 | Q6NSP9;P52927 | High mobility group protein HMGI-C | Hmga2 | 25.32974815 | 25.24822044 | 25.20482254 | 24.81597137 | 24.86386681 | 24.85846901 |
| **+** | 2.532590145 | 0.417467117 | Q8BWT1;A0A494B9J2 | 3-ketoacyl-CoA thiolase, mitochondrial | Acaa2 | 26.02533913 | 25.9237709 | 26.09766197 | 25.67847252 | 25.55952644 | 25.55637169 |
| **+** | 6.189350849 | 0.417861303 | P56480 | ATP synthase subunit beta, mitochondrial | Atp5b | 28.61810493 | 28.62508965 | 28.61988831 | 28.19441605 | 28.19746399 | 28.21761894 |
| **+** | 4.450575319 | 0.419750214 | P28650;J3QN31 | Adenylosuccinate synthetase isozyme 1 | Adssl1 | 24.37961006 | 24.34407997 | 24.40736771 | 23.94628143 | 23.97683716 | 23.94868851 |
| **+** | 1.882665354 | 0.421117783 | G3UZ34;A2AH85;O08810;G3UXK8 | 116 kDa U5 small nuclear ribonucleoprotein component | Eftud2 | 22.92817497 | 23.16679001 | 23.0442028 | 22.49526405 | 22.64061737 | 22.73993301 |
| **+** | 2.324618891 | 0.421337128 | O88653;A0A0G2JGQ3 | Ragulator complex protein LAMTOR3 | Lamtor3 | 24.90641975 | 24.91031265 | 24.94377899 | 24.64267731 | 24.45048141 | 24.40334129 |
| **+** | 3.793913531 | 0.423377355 | Q99N15;A2AFQ2;O08756 | 3-hydroxyacyl-CoA dehydrogenase type-2 | Hsd17b10 | 25.22523308 | 25.18321228 | 25.19110489 | 24.800354 | 24.72088051 | 24.80818367 |
| **+** | 2.239754467 | 0.424462636 | Q9QXB9 | Developmentally-regulated GTP-binding protein 2 | Drg2 | 23.22840691 | 22.99762344 | 23.05592918 | 22.59871101 | 22.68275452 | 22.72710609 |
| **+** | 2.349395416 | 0.426305771 | Q9Z2I0 | LETM1 and EF-hand domain-containing protein 1, mitochondrial | Letm1 | 24.35137749 | 24.34827232 | 24.35655785 | 23.96393394 | 23.78316689 | 24.03018951 |
| **+** | 2.254652089 | 0.427047094 | Q99JB2;A2AG39;F6WI02;A2AG41 | Stomatin-like protein 2, mitochondrial | Stoml2 | 23.32471085 | 23.30215073 | 23.31174469 | 22.89287758 | 23.01779175 | 22.74679565 |
| **+** | 1.907819983 | 0.42862765 | Q9D819 | Inorganic pyrophosphatase | Ppa1 | 23.77635574 | 23.78436661 | 23.87577057 | 23.20184326 | 23.51448631 | 23.4342804 |
| **+** | 1.651256657 | 0.429581324 | Q91YP2;A0A286YD12;A0A286YD77 | Neurolysin, mitochondrial | Nln | 22.8133316 | 22.56725311 | 22.69737816 | 22.07535362 | 22.33199692 | 22.38186836 |
| **+** | 3.083205947 | 0.430774689 | F6WR04;O70370 | Cathepsin S | Ctss | 29.14219093 | 29.14557266 | 29.12713814 | 28.64602089 | 28.67613411 | 28.80042267 |
| **+** | 4.556904005 | 0.431845983 | Q921F2;Q6VYI4;Q6VYI5;Q8BLD4;Q8R0B4;A0A087WRZ5;A0A087WR97;A0A087WQA5;A0A087WSH7;A0A087WSE4;H3BJV1;A0A087WRP4;A0A087WS74;A0A087WNY6;A0A087WQX8;A0A087WS17;A0A087WP57;A0A087WSC6 | TAR DNA-binding protein 43 | Tardbp | 24.5731144 | 24.51677132 | 24.56337166 | 24.13547516 | 24.12147141 | 24.10077286 |
| **+** | 2.218833895 | 0.434704463 | G3X8X7;Q920Q4;A2BI90 | Vacuolar protein sorting-associated protein 16 homolog | Vps16 | 22.6065712 | 22.62573624 | 22.3924675 | 22.10098076 | 22.16729546 | 22.05238533 |
| **+** | 1.136800543 | 0.437065125 | A0A286YDB7;A0A286YCT4;A0A286YCG8;Q9CY50 | Translocon-associated protein subunit alpha | Ssr1 | 25.11036301 | 24.83161354 | 24.82293892 | 24.23622513 | 24.45035553 | 24.76713943 |
| **+** | 3.18589946 | 0.437252045 | Q9Z2Z6 | Mitochondrial carnitine/acylcarnitine carrier protein | Slc25a20 | 23.18999672 | 23.26842308 | 23.31906891 | 22.87088966 | 22.81023598 | 22.78460693 |
| **+** | 1.257749264 | 0.438699722 | A0A0U1RP13;Q8VDP6;A0A0U1RNI6;A0A0U1RPV3;A0A0U1RQ57;A0A0U1RP60;Q05BY1 | CDP-diacylglycerol--inositol 3-phosphatidyltransferase | Cdipt | 24.70128822 | 24.38890076 | 24.75806618 | 23.96137428 | 24.20866013 | 24.36212158 |
| **+** | 3.046452066 | 0.439132055 | P00405 | Cytochrome c oxidase subunit 2 | Mtco2 | 26.76724052 | 26.79538345 | 26.69670486 | 26.38692856 | 26.2491436 | 26.30586052 |
| **+** | 1.527221744 | 0.440636953 | E9QMX7;Q640N3 | Rho GTPase-activating protein 30 | Arhgap30 | 23.26198387 | 23.18436241 | 22.91130066 | 22.71931458 | 22.79255104 | 22.52387047 |
| **+** | 3.121278808 | 0.441761017 | Q9JIG7 | Coiled-coil domain-containing protein 22 | Ccdc22 | 23.40898705 | 23.47320747 | 23.54921913 | 23.03707886 | 23.07809258 | 22.99095917 |
| **+** | 2.832094524 | 0.443009059 | Q62426 | Cystatin-B | Cstb | 29.59106445 | 29.5298996 | 29.53973007 | 29.15192223 | 29.17569542 | 29.0040493 |
| **+** | 1.655140253 | 0.446243286 | Q8R4R6;A2ATJ2 | Nucleoporin NUP53 | Nup35 | NaN | 21.88136482 | 21.95939445 | 21.41905403 | 21.52921867 | NaN |
| **+** | 2.234014279 | 0.447583516 | Q9WTP7;F6RP11 | GTP:AMP phosphotransferase AK3, mitochondrial | Ak3 | 23.62466431 | 23.42495918 | 23.54450989 | 23.10402489 | 22.9712677 | 23.17609024 |
| **+** | 1.884865261 | 0.448080063 | Q8BU33;A0A1W2P8E1;A0A1W2P727;A0A1W2P8D6;A0A1W2P6L8 | Acetolactate synthase-like protein | Ilvbl | 23.15117073 | 23.16567039 | 23.15506744 | 22.50035477 | 22.83335304 | 22.79396057 |
| **+** | 1.733458742 | 0.44938151 | O70172;F6RJE8;A2ASS3 | Phosphatidylinositol 5-phosphate 4-kinase type-2 alpha | Pip4k2a | 23.82609558 | 23.93544006 | 23.64328384 | 23.22834969 | 23.32580757 | 23.5025177 |
| **+** | 2.400293707 | 0.450203578 | Q9Z110;D3Z0B4;H3BKJ8;H3BLE8 | Delta-1-pyrroline-5-carboxylate synthase;Glutamate 5-kinase;Gamma-glutamyl phosphate reductase | Aldh18a1 | 24.24590302 | 24.12036514 | 24.0205574 | 23.74036598 | 23.68688393 | 23.60896492 |
| **+** | 1.495111381 | 0.456505458 | Q91VD9;A0A087WQ77;A0A087WSU3 | NADH-ubiquinone oxidoreductase 75 kDa subunit, mitochondrial | Ndufs1 | 22.68045998 | 22.6298542 | 22.91002083 | 22.06259918 | 22.36238861 | 22.42583084 |
| **+** | 3.058669125 | 0.457050959 | P55772;Q8CDV7;F7B9M9;D6RHQ2;D6RFA9 | Ectonucleoside triphosphate diphosphohydrolase 1 | Entpd1 | 25.09418106 | 24.93093681 | 24.96194839 | 24.524086 | 24.56016731 | 24.53166008 |
| **+** | 1.616141941 | 0.458432515 | P35282;A0A1W2P6Z4 | Ras-related protein Rab-21 | Rab21 | 24.65992355 | 24.60087967 | 24.9789753 | 24.30577278 | 24.37398148 | 24.18472672 |
| **+** | 3.311948619 | 0.460828145 | P20108 | Thioredoxin-dependent peroxide reductase, mitochondrial | Prdx3 | 25.66006088 | 25.54816246 | 25.60354042 | 25.0871563 | 25.15000153 | 25.19212151 |
| **+** | 3.633452494 | 0.463787715 | Q6PGH1 | Protein BUD31 homolog | Bud31 | 21.83047295 | 21.88285828 | 21.8900032 | 21.35670471 | 21.46463394 | 21.39063263 |
| **+** | 1.255572635 | 0.465795517 | Q9D0S9 | Histidine triad nucleotide-binding protein 2, mitochondrial | Hint2 | 24.68120956 | 24.2663517 | 24.23921585 | 23.93759727 | 23.75399208 | 24.09780121 |
| **+** | 2.120317364 | 0.469035467 | Q8VEH3;F6QKK2 | ADP-ribosylation factor-like protein 8A | Arl8a | 25.93210983 | 26.16394234 | 26.10974503 | 25.67227936 | 25.65212059 | 25.47429085 |
| **+** | 4.035167199 | 0.470053355 | O08749 | Dihydrolipoyl dehydrogenase, mitochondrial | Dld | 24.95006943 | 24.97140884 | 24.91592979 | 24.46880341 | 24.43663406 | 24.52181053 |
| **+** | 1.145609643 | 0.47289594 | Q9QWL7;Q99PS0 | Keratin, type I cytoskeletal 17 | Krt17 | 23.63289642 | 23.79709244 | 23.739748 | 23.44189835 | 22.87423325 | 23.43491745 |
| **+** | 2.399174158 | 0.473909378 | P67778;Q5SQG5 | Prohibitin | Phb | 25.77499962 | 25.82140732 | 25.73781586 | 25.20724678 | 25.25165558 | 25.4535923 |
| **+** | 3.037254717 | 0.480353038 | Q922U2;P07744 | Keratin, type II cytoskeletal 5 | Krt5 | 26.01692963 | 25.92361259 | 26.04553986 | 25.59457207 | 25.48594284 | 25.46450806 |
| **+** | 3.143249229 | 0.480567932 | Q6NXH9 | Keratin, type II cytoskeletal 73 | Krt73 | 27.2624855 | 27.21085358 | 27.33866692 | 26.83725548 | 26.81200981 | 26.72103691 |
| **+** | 1.314725535 | 0.48184522 | E9QMK9;Q8BH86 | UPF0317 protein C14orf159 homolog, mitochondrial | 9030617O03Rik | 22.56064606 | 22.33867645 | 22.68946648 | 21.85052681 | 21.97987366 | 22.31285286 |
| **+** | 3.712185334 | 0.482935588 | Q8BMF4 | Dihydrolipoyllysine-residue acetyltransferase component of pyruvate dehydrogenase complex, mitochondrial | Dlat | 24.87960815 | 24.80715179 | 24.77730942 | 24.34577179 | 24.29942894 | 24.37006187 |
| **+** | 1.083273472 | 0.483420054 | Q9CQK7 | RWD domain-containing protein 1 | Rwdd1 | 22.41913223 | 22.93115425 | 22.46480751 | 21.96426964 | 22.01811409 | 22.3824501 |
| **+** | 2.87158428 | 0.484710693 | Q62048;D3Z375 | Astrocytic phosphoprotein PEA-15 | Pea15;Pea15a | 25.536026 | 25.60178566 | 25.54615974 | 24.98901939 | 25.1843853 | 25.05643463 |
| **+** | 2.743901603 | 0.484782537 | P28474;A0A0G2JGS3;Q64437 | Alcohol dehydrogenase class-3 | Adh5 | 25.43872833 | 25.42040443 | 25.52948761 | 24.86523628 | 25.0322094 | 25.03682709 |
| **+** | 3.069068445 | 0.486164093 | Q9D3D9;D3Z7S4 | ATP synthase subunit delta, mitochondrial | Atp5d | 26.58792114 | 26.44837189 | 26.53568459 | 26.10751152 | 26.01678085 | 25.98919296 |
| **+** | 3.681772413 | 0.486885071 | P20152;A0A0A6YWC8;A2AKJ2;P31001;G5E846;A0A2R8W6R6;G3X981;P15331;D3YZ35;A0A0R4J036;P46660;P08553;P08551 | Vimentin | Vim | 30.97739601 | 31.01025391 | 30.99658012 | 30.52654648 | 30.55964851 | 30.43737984 |
| **+** | 1.959203169 | 0.488927205 | Q9CQB4;Q9D855 | Cytochrome b-c1 complex subunit 7 | Uqcrb | 23.99533081 | 24.16230965 | 24.06587219 | 23.72691917 | 23.39780426 | 23.6320076 |
| **+** | 2.195458255 | 0.489959717 | Q8BWQ6;D3YW20;D3YW19;I1E4X5;F6RR19;H3BJE8 | UPF0505 protein C16orf62 homolog | 9030624J02Rik | 22.75460434 | 22.89463615 | 22.92685318 | 22.47607803 | 22.41142082 | 22.21871567 |
| **+** | 1.783551892 | 0.491665522 | Q8R1V4;Q5SVW9 | Transmembrane emp24 domain-containing protein 4 | Tmed4 | 23.50239563 | 23.34759712 | 23.54049683 | 22.89211655 | 22.8365345 | 23.18684196 |
| **+** | 1.742001551 | 0.497216543 | Q3TCW6;P34960;Q8BJC0;D3Z705 | Macrophage metalloelastase | Mmp12 | 23.22110748 | 23.45538139 | 23.19908905 | 22.76338577 | 22.9800148 | 22.64052773 |
| **+** | 3.453578076 | 0.498121897 | Q9D0M3;A0A2R8VHK1 | Cytochrome c1, heme protein, mitochondrial | Cyc1 | 24.51304054 | 24.62950897 | 24.51231766 | 24.07528877 | 24.07332802 | 24.01188469 |
| **+** | 2.541910655 | 0.49943161 | A0A087WPT7;Q6P549;A0A1B0GR64;A0A1B0GST7 | Phosphatidylinositol 3,4,5-trisphosphate 5-phosphatase 2 | Inppl1 | 24.14779091 | 24.0134201 | 23.93912315 | 23.62622643 | 23.47864723 | 23.49716568 |
| **+** | 2.493114604 | 0.500761032 | Q80UM7 | Mannosyl-oligosaccharide glucosidase | Mogs | 23.27241516 | 23.27284241 | 23.17723083 | 22.72665024 | 22.62153816 | 22.87201691 |
| **+** | 2.16341461 | 0.503257116 | Q8CC88 | von Willebrand factor A domain-containing protein 8 | Vwa8 | 22.55173302 | 22.72048378 | 22.41818047 | 22.10983658 | 21.97109222 | 22.09969711 |
| **+** | 4.288465856 | 0.508467992 | A2A513;P02535;B1ATJ5;Q61897;Q62168;Q61765;Q6IFX3;Q8VCW2;Q9Z320;Q497I4 | Keratin, type I cytoskeletal 10 | Krt10 | 25.34955597 | 25.32385254 | 25.31409836 | 24.78656197 | 24.80484009 | 24.87070084 |
| **+** | 1.317426208 | 0.509261449 | Q8CGB9;Q9JHR7 | Insulin-degrading enzyme | Ide | 26.2614994 | 26.2743721 | 26.11777115 | 25.78760719 | 25.37577248 | 25.96247864 |
| **+** | 2.447300897 | 0.511599859 | A0A0A0MQA5;P68368;A0A087WQS4;A0A087WRB4;A0A087WSB0;A0A087WSL5;Q3UX10;A0A087WS35 | Tubulin alpha-4A chain | Tuba4a | 25.42831802 | 25.55332756 | 25.45041847 | 24.82298851 | 25.00397491 | 25.07030106 |
| **+** | 2.374782329 | 0.519194285 | D3YUM1;Q91YT0;D3Z1U9;D6RG60;A0A494BAS8;D3Z0K1;D3YXX5 | NADH dehydrogenase [ubiquinone] flavoprotein 1, mitochondrial | Ndufv1 | 22.58982658 | 22.68889046 | 22.55849838 | 22.16189384 | 21.93502617 | 22.18271255 |
| **+** | 2.272212471 | 0.521921158 | E9Q634;A0A1L1STM1 | Unconventional myosin-Ie | Myo1e | 23.68153191 | 23.63511276 | 23.60104942 | 23.29544067 | 23.06858063 | 22.98790932 |
| **+** | 1.148254994 | 0.530194283 | P61971 | Nuclear transport factor 2 | Nutf2 | 25.15614891 | NaN | 25.14343834 | 24.76890564 | 24.47029305 | NaN |
| **+** | 1.550205313 | 0.536035538 | Q8BGB7 | Enolase-phosphatase E1 | Enoph1 | 22.68861389 | 23.09881401 | 22.91743279 | 22.57722473 | 22.27631187 | 22.24321747 |
| **+** | 1.37829231 | 0.545717239 | P0DN34 | NADH dehydrogenase [ubiquinone] 1 beta subcomplex subunit 1 | Ndufb1 | 22.6881218 | 23.26927948 | 23.03071213 | 22.57537842 | 22.46126366 | 22.31431961 |
| **+** | 3.281009804 | 0.555465062 | Q9CQW2;A0A0N4SVB8 | ADP-ribosylation factor-like protein 8B | Arl8b | 27.16901779 | 27.20284462 | 27.129179 | 26.65594673 | 26.51133728 | 26.66736221 |
| **+** | 3.814413767 | 0.557291031 | P35486 | Pyruvate dehydrogenase E1 component subunit alpha, somatic form, mitochondrial | Pdha1 | 24.22317123 | 24.25486565 | 24.22927284 | 23.6482296 | 23.63178635 | 23.75542068 |
| **+** | 2.247645237 | 0.560139338 | A0A087WR20;P49935 | Pro-cathepsin H;Cathepsin H mini chain;Cathepsin H;Cathepsin H heavy chain;Cathepsin H light chain | Ctsh | NaN | 23.62967682 | 23.64118958 | 23.18027115 | 23.07550049 | 22.97010994 |
| **+** | 4.115489625 | 0.57433637 | A0A7N9VR94 | AHNAK nucleoprotein 2 | Ahnak2 | 27.50930023 | 27.54502678 | 27.47645569 | 26.92411041 | 26.89388657 | 26.98977661 |
| **+** | 1.700240299 | 0.574562391 | Q7TMF3;A0A0R4J275;F6RBR6 | NADH dehydrogenase [ubiquinone] 1 alpha subcomplex subunit 12 | Ndufa12 | 21.94695854 | 22.28551292 | 22.1288681 | NaN | 21.55295181 | 21.53881645 |
| **+** | 1.731490586 | 0.575889587 | O70251;A0A087WS46;G3UX43;M0QWK5;M0QWH8;G3UZ47 | Elongation factor 1-beta | Eef1b;Eef1b2 | 25.69649315 | 25.80923843 | 25.96485901 | 25.11632538 | 25.12198639 | 25.50461006 |
| **+** | 2.767522111 | 0.57671992 | Q8BIW1 | Protein prune homolog | Prune | 22.60324669 | 22.45841408 | 22.47837639 | 21.8147583 | 22.0206604 | 21.97445869 |
| **+** | 1.25507758 | 0.581383705 | Q922K7;E9QN31;A0A0N4SW16 | Probable 28S rRNA (cytosine-C(5))-methyltransferase | Nop2 | 22.92159843 | NaN | 22.69408798 | 22.49125671 | 22.0891304 | 22.09899139 |
| **+** | 1.494328174 | 0.581809998 | G5E8A0;A0A338P6F2;Q8CI95 | Oxysterol-binding protein;Oxysterol-binding protein-related protein 11 | Osbpl11 | 21.49887085 | 21.51470184 | 21.9780941 | 20.9057312 | 21.17590714 | 21.16459846 |
| **+** | 4.360776247 | 0.5842158 | Q3U9N4;P28798;H3BJE0;H3BJ90;H3BLC9 | Granulins;Acrogranin;Granulin-1;Granulin-2;Granulin-3;Granulin-4;Granulin-5;Granulin-6;Granulin-7 | Grn | 27.8696785 | 27.83171082 | 27.91598701 | 27.27488708 | 27.32440186 | 27.26543999 |
| **+** | 1.394092201 | 0.585386912 | Q91V77;P56565;D3YUT6 | Protein S100;Protein S100-A1 | S100a1 | 24.49167252 | 24.20523453 | 24.23702812 | 23.42316437 | 23.73024178 | 24.02436829 |
| **+** | 1.534626443 | 0.58794721 | Q9CQX8;Q9D6T9 | 28S ribosomal protein S36, mitochondrial | Mrps36 | 24.46724892 | 24.41183281 | 24.64284325 | 23.73489952 | 24.24321747 | 23.77996635 |
| **+** | 2.82334076 | 0.59680589 | Q9EQ32 | Phosphoinositide 3-kinase adapter protein 1 | Pik3ap1 | 22.72949409 | 22.60970879 | 22.68547249 | NaN | 22.04086304 | 22.11530876 |
| **+** | 4.167596461 | 0.60362943 | Q99L43;A2AMQ5;Q6PBC0;F6S4G2 | Phosphatidate cytidylyltransferase 2;Phosphatidate cytidylyltransferase | Cds2 | 23.28149986 | 23.20934486 | 23.25824928 | 22.59281731 | 22.65726662 | 22.6881218 |
| **+** | 2.317440964 | 0.626481374 | Q9DCT2 | NADH dehydrogenase [ubiquinone] iron-sulfur protein 3, mitochondrial | Ndufs3 | 23.1687336 | 22.88520622 | 23.19971848 | 22.55175591 | 22.39796066 | 22.4244976 |
| **+** | 1.002950098 | 0.626626333 | Q9CQN6 | Transmembrane protein 14C | Tmem14c | 23.51099014 | 23.10683823 | 23.98858643 | 22.68553734 | 23.18022537 | 22.86077309 |
| **+** | 1.184782684 | 0.629487991 | O54890 | Integrin beta-3 | Itgb3 | 23.30619049 | 22.62102509 | 23.33551788 | 22.28404427 | 22.51373863 | 22.57648659 |
| **+** | 2.391570592 | 0.629891078 | P04104 | Keratin, type II cytoskeletal 1 | Krt1 | 26.0355072 | 26.16474724 | 25.95291519 | 25.44319534 | 25.55885506 | 25.261446 |
| **+** | 1.830636827 | 0.631207784 | Q9CPU0;A0A494BAF7;A0A494BBE7;A0A494B970 | Lactoylglutathione lyase | Glo1 | 24.04576874 | 23.90306664 | 23.89568901 | 23.25968742 | 23.09812355 | 23.59309006 |
| **+** | 3.105080324 | 0.653716405 | Q99LC3;A0A087WR38 | NADH dehydrogenase [ubiquinone] 1 alpha subcomplex subunit 10, mitochondrial | Ndufa10 | 22.6602726 | 22.83095741 | 22.68587875 | 21.97777939 | 22.12984276 | 22.1083374 |
| **+** | 1.397013244 | 0.657197952 | A0A087WRC6;A0A087WR57;Q91XC8 | Death-associated protein 1 | Dap | 23.69805717 | 23.52504158 | 23.33142281 | 22.57357597 | 22.78392792 | 23.22542381 |
| **+** | 4.571636333 | 0.675069173 | A0A0R4J083;P51174 | Long-chain specific acyl-CoA dehydrogenase, mitochondrial | Acadl | 25.69938278 | 25.68099594 | 25.77535057 | 25.04380989 | 25.06357002 | 25.02314186 |
| **+** | 0.990256598 | 0.685296059 | Q924Z4;D3Z4M2;D3YTM0;D3Z0Z2 | Ceramide synthase 2 | Cers2 | 24.48615837 | NaN | 24.01069069 | 23.5685997 | 23.55765724 | NaN |
| **+** | 2.305334722 | 0.687053045 | P97821 | Dipeptidyl peptidase 1;Dipeptidyl peptidase 1 exclusion domain chain;Dipeptidyl peptidase 1 heavy chain;Dipeptidyl peptidase 1 light chain | Ctsc | 23.16074181 | 22.95162582 | 23.36866379 | 22.42930794 | 22.49098778 | 22.49957657 |
| **+** | 2.436350404 | 0.71650823 | Q9CQR4 | Acyl-coenzyme A thioesterase 13;Acyl-coenzyme A thioesterase 13, N-terminally processed | Acot13 | 25.11723709 | 25.41322327 | 25.10425568 | 24.59423065 | 24.38613892 | 24.50482178 |
| **+** | 1.756580376 | 0.724371592 | Q810Q5;A2AK37 | Normal mucosa of esophagus-specific gene 1 protein | Nmes1;AA467197 | 24.50766754 | 24.71187782 | 24.38311005 | 23.82522202 | 23.52707291 | 24.07724571 |
| **+** | 3.762146573 | 0.731874466 | Q61838 | Alpha-2-macroglobulin;Alpha-2-macroglobulin 165 kDa subunit;Alpha-2-macroglobulin 35 kDa subunit | A2m | 27.94268417 | 28.0730629 | 27.97322845 | 27.33432579 | 27.20778656 | 27.25123978 |
| **+** | 4.24653539 | 0.765289307 | P09528;A0A494BA92;A0A494B9D4;A0A494BAP3 | Ferritin heavy chain;Ferritin heavy chain, N-terminally processed | Fth1 | 26.74650383 | 26.6875248 | 26.64333916 | 25.93429375 | 25.9759407 | 25.87126541 |
| **+** | 1.661956858 | 0.807320913 | Q9CZU3 | Superkiller viralicidic activity 2-like 2 | Skiv2l2 | NaN | 26.15332985 | 26.24172783 | 25.6672802 | 25.21848679 | 25.2848568 |
| **+** | 1.696253489 | 0.824757576 | A0A498WFS2;Q922Y1 | UBX domain-containing protein 1 | Ubxn1 | 24.13969612 | 24.16530418 | 23.73737717 | 23.23470688 | NaN | 23.14402962 |
| **+** | 3.682490742 | 0.825727463 | Q3TTY5 | Keratin, type II cytoskeletal 2 epidermal | Krt2 | 24.93571091 | 24.99252319 | 24.86154938 | 24.12289429 | 24.18260574 | 24.00710106 |
| **+** | 3.041672111 | 0.827517192 | P12265;Q99KJ6;D3YY48 | Beta-glucuronidase | Gusb | 23.88706779 | 23.76779556 | 23.78286743 | 23.13146019 | 22.8339901 | 22.98972893 |
| **+** | 4.284375534 | 0.863489151 | Q60648 | Ganglioside GM2 activator | Gm2a | 25.97070694 | 25.94223595 | 25.97024536 | 25.03527641 | 25.06980896 | 25.18763542 |
| **+** | 3.02018697 | 0.873009364 | Q8R5L1;O35658 | Complement component 1 Q subcomponent-binding protein, mitochondrial | C1qbp | 24.8994751 | 24.98415756 | 24.94190025 | 23.90104103 | 24.0679245 | 24.23753929 |
| **+** | 3.197253231 | 0.905281703 | A0A1B0GR60;Q9CPX4;P29391;P49945;A0A1Y7VNT9;A0A1B0GRH4 | Ferritin;Ferritin light chain 1 | Ftl1 | 26.43906212 | 26.71292877 | 26.57773209 | 25.72340775 | 25.71871376 | 25.57175636 |
| **+** | 4.130373416 | 0.914655685 | E9Q9C5;P63082 | V-type proton ATPase 16 kDa proteolipid subunit | Atp6v0c | 27.68458557 | 27.66817284 | 27.69670486 | 26.86714554 | 26.6815052 | 26.75684547 |
| **+** | 2.811187803 | 0.935563405 | A0A0R4J069;Q9JLI6;F7D651;F6ZFP8;D3Z7G6;F7BB44 | Selenocysteine lyase | Scly | 23.79124069 | 24.16200256 | 23.91259956 | 23.12759399 | 22.97214508 | 22.95941353 |
| **+** | 2.87493622 | 0.944239934 | Q00519;A0A3B2WBF8;A0A3B2W454;A0A3B2WDA8 | Xanthine dehydrogenase/oxidase;Xanthine dehydrogenase;Xanthine oxidase | Xdh | 22.96017265 | 22.79284859 | 23.04433632 | 21.98714638 | 22.14864349 | 21.82884789 |
|  | 0.595743991 | -0.581852913 | Q3UMB9 | WASH complex subunit 7 | Kiaa1033 | NaN | 22.85224342 | 23.20457649 | 23.93210983 | 23.28841591 | NaN |
|  | 0.417611709 | -0.518039703 | P56394 | Cytochrome c oxidase copper chaperone | Cox17 | 23.71764183 | 22.85644722 | 21.97200584 | 23.35743141 | 23.09707832 | 23.64570427 |
|  | 0.921941526 | -0.516258558 | Q9QXD8 | LIM domain-containing protein 1 | Limd1 | 21.75759888 | 22.32495689 | 21.88840675 | 22.62765121 | NaN | 22.38550758 |
|  | 0.633380207 | -0.512536049 | A0A338P6P6;D4AFX6;Q6ZQK5 | Arf-GAP with coiled-coil, ANK repeat and PH domain-containing protein 2 | Acap2 | NaN | 22.33440018 | 22.60995674 | NaN | 23.25436211 | 22.71506691 |
|  | 0.760916029 | -0.441057841 | G3UVU2;D3YW09;Q62203;D3Z5A6 | Splicing factor 3A subunit 2 | Sf3a2 | 22.96736908 | 22.99139023 | 23.32621956 | 23.27056313 | 23.80153847 | NaN |
|  | 0.163150268 | -0.435995738 | E9PZF4;Q80XB4;E9Q641;F7CXG1 | Nebulin-related-anchoring protein | Nrap | 30.27364349 | 30.31559753 | 32.50585556 | 32.83392334 | 30.81191826 | 30.7572422 |
|  | 0.911559133 | -0.388968786 | Q3ULG5;P97311 | DNA helicase;DNA replication licensing factor MCM6 | Mcm6 | 22.63260651 | 22.85680771 | 22.72192383 | 23.49326134 | 22.8704567 | 23.01452637 |
|  | 0.659745929 | -0.348312378 | O88811 | Signal transducing adapter molecule 2 | Stam2 | 26.8967762 | 27.17797661 | NaN | 27.69325256 | 27.24508667 | 27.21872711 |
|  | 0.733843227 | -0.3424263 | Q9R190 | Metastasis-associated protein MTA2 | Mta2 | 22.61735153 | 22.5328846 | NaN | 23.08415794 | 22.75093079 | NaN |
|  | 0.797151025 | -0.332016309 | A0A087WQM0;A0A087WRU0;A0A087WQ94;A0A6I8MWZ2;A0A087WQS0;A0A1D5RM59;E9Q0S6;Q9DBT6 | Tensin 1 | Tns1 | 22.2281723 | 22.18652534 | NaN | 22.38044357 | 22.81340981 | 22.42424202 |
|  | 0.729705631 | -0.33106486 | Q9R1J0 | Sterol-4-alpha-carboxylate 3-dehydrogenase, decarboxylating | Nsdhl | 21.62046432 | 21.90437126 | 22.21543121 | 22.21794701 | 22.05662537 | 22.45888901 |
|  | 0.436630641 | -0.329910278 | Q9Z1D1 | Eukaryotic translation initiation factor 3 subunit G | Eif3g | 23.58267784 | 22.90974617 | 23.98737144 | 23.88175774 | 23.91916275 | 23.6686058 |
|  | 1.228293384 | -0.325540543 | Q9R1R8;Q9QYF1 | Retinol dehydrogenase 11 | Rdh11 | 22.96232796 | 22.8454628 | 22.54608917 | 23.13078499 | 23.09042168 | 23.10929489 |
|  | 0.659569002 | -0.324537595 | Q8JZZ5;P53811 | Phosphatidylinositol transfer protein beta isoform | Pitpnb | NaN | 23.08345985 | 23.5985527 | 23.61369324 | 23.58852196 | 23.79441643 |
|  | 0.536365512 | -0.324478785 | Q9JIA7;A0A1B0GS43 | Sphingosine kinase 2 | Sphk2 | 23.15519142 | 22.46243858 | 22.73438263 | 23.38929367 | 22.78741837 | 23.14873695 |
|  | 0.880677531 | -0.320005099 | Q3UE37 | Ubiquitin-conjugating enzyme E2 Z | Ube2z | NaN | 22.40789795 | 22.33382607 | 22.70588112 | 22.88814545 | 22.47857475 |
|  | 1.133723257 | -0.307177862 | Q04899 | Cyclin-dependent kinase 18 | Cdk18 | 23.87389565 | 24.28376198 | 24.11433983 | 24.48382187 | 24.38218689 | 24.32752228 |
|  | 1.29351331 | -0.305787404 | A0A0G2JGD2;P07091 | Protein S100-A4 | S100a4 | 29.30875778 | 29.29184532 | 29.63082886 | 29.69634056 | 29.71904755 | 29.73340607 |
|  | 0.956482893 | -0.299775441 | Q8K1T1 | Leucine-rich repeat-containing protein 25 | Lrrc25 | 23.24474144 | 23.34516335 | 23.51929283 | 23.85262489 | 23.71983719 | 23.43606186 |
|  | 0.684728703 | -0.299455007 | Q3TC93;A0A1W2P6R8;A0A1W2P6H5 | HCLS1-binding protein 3 | Hs1bp3 | 23.92406464 | 23.48345375 | 23.60828781 | 24.18139267 | 23.68185425 | 24.0509243 |
|  | 0.438179763 | -0.298951149 | Q9Z1J3;F7CZD1;F6TXD3 | Cysteine desulfurase, mitochondrial | Nfs1 | 23.19587898 | 22.98858643 | NaN | 23.73799515 | 23.41931152 | 23.01624489 |
|  | 1.270812686 | -0.295476278 | Q8BMK4 | Cytoskeleton-associated protein 4 | Ckap4 | 22.74110603 | 22.47588158 | 22.38152695 | 22.85872459 | 22.79748726 | 22.82873154 |
|  | 1.119626274 | -0.293525696 | Q9DCC4 | Pyrroline-5-carboxylate reductase 3 | Pycrl | 22.44904518 | 22.80238724 | 22.61495018 | 22.77733994 | 22.98582649 | 22.98379326 |
|  | 0.830901771 | -0.285975774 | E9PYK0;Q8VCW4;A0A494BBK7;E9Q8P6 | Protein unc-93 homolog B1 | Unc93b1 | 22.98712921 | 22.90987396 | 23.40665436 | 23.45010376 | 23.31132889 | 23.40015221 |
|  | 0.5917154 | -0.284521103 | Q99P31;A0A0U1RPF2;A0A0U1RPE7;A0A0U1RQ49 | Hsp70-binding protein 1 | Hspbp1 | 22.53604317 | 22.47030449 | 22.96552086 | 22.9867115 | 22.89690971 | NaN |
|  | 0.881286846 | -0.282225927 | P97450;E9QAD6 | ATP synthase-coupling factor 6, mitochondrial | Atp5j | 25.31568718 | 25.44502831 | 25.3924427 | 25.73874283 | 25.87265205 | 25.38844109 |
|  | 1.17465517 | -0.279029846 | F6WMJ3;Q8K4I3;A2AFJ8;H3BKQ0;H3BJ53 | Rho guanine nucleotide exchange factor 6 | Arhgef6 | 22.546278 | 22.39801216 | 22.44101143 | 22.69618988 | 22.58957481 | 22.93662643 |
|  | 0.837823518 | -0.27833271 | Q3TBU7;Q3U2K8;Q80WC7 | Arf-GAP domain and FG repeat-containing protein 2 | Agfg2 | NaN | 22.37052727 | 22.36594391 | 22.86502838 | 22.56202126 | 22.51265526 |
|  | 0 | -0.277668953 | Q62086 | Serum paraoxonase/arylesterase 2 | Pon2 | NaN | 22.49084091 | NaN | 22.78743935 | 22.74958038 | NaN |
|  | 0.533298114 | -0.276746114 | P30935 | Somatostatin receptor type 3 | Sstr3 | 23.84498405 | NaN | 23.60964012 | 24.0582943 | 23.71408272 | 24.23979759 |
|  | 0.605212364 | -0.274636269 | A0A1B0GQY8;D6RHM6;D3Z2F3;A0A1B0GSM3;A0A1B0GS63;Q8K221 | Arfaptin-2 | Arfip2 | 20.49335861 | 20.70210075 | NaN | NaN | 21.00696373 | 20.73776817 |
|  | 1.457190403 | -0.273359299 | Q60855;F7D1J2 | Receptor-interacting serine/threonine-protein kinase 1 | Ripk1 | 22.20553207 | 22.33440018 | 22.49250412 | 22.63995361 | 22.56479073 | 22.64776993 |
|  | 0.943817681 | -0.269634247 | I1E4X0;F6YMR0;E9PUF2;B7ZNS2;B1AZP2;H3BJW9;H3BKJ0;H3BL90;H3BJD4 | Disks large-associated protein 4 | Dlgap4 | 22.55060577 | 22.38255501 | 22.33128738 | 22.59696198 | 22.92196083 | 22.5544281 |
|  | 1.324389727 | -0.268925985 | A0A2I3BRQ3;Q61704 | Inter-alpha-trypsin inhibitor heavy chain H3 | Itih3 | 26.28302002 | 26.53790665 | 26.50382233 | 26.63339424 | 26.80820847 | 26.68992424 |
|  | 0.861616633 | -0.267786662 | P62748;A0A1Y7VMK3;P84075;A2A7R5;E9PV73;D3Z2Z8;D3YVA2;Q91X97 | Hippocalcin-like protein 1;Neuron-specific calcium-binding protein hippocalcin | Hpcal1;Hpca | 23.63854027 | 23.83826637 | 23.50263786 | 24.08942032 | 23.72619247 | 23.9671917 |
|  | 1.213718427 | -0.265981674 | A8DUK4;P02089;P02088;E9Q223;P02104 | Hemoglobin subunit beta-2;Hemoglobin subunit beta-1;Hemoglobin subunit epsilon-Y2 | Hbbt1;Hbb-b2;Hbb-b1;Hbb-bs;Hbb-y | 26.46380806 | 26.40390968 | 26.68525314 | 26.74534988 | 26.7092514 | 26.89631462 |
|  | 1.397862286 | -0.265837987 | Q8CFQ9;P56959;G3UXT7;G3UZD2;Q91VQ2 | RNA-binding protein FUS | Fus | 23.97762299 | 24.06833458 | 24.05581474 | 24.4651947 | 24.24234581 | 24.19174576 |
|  | 0.506890924 | -0.265446345 | Q9CPT4 | Myeloid-derived growth factor | Mydgf | 24.06931877 | 24.47914124 | 23.76658249 | 24.48142242 | 24.45807648 | 24.17188263 |
|  | 1.458327638 | -0.262742678 | A0A0R4J007;P70261 | Paladin | Pald1 | NaN | 22.38874245 | 22.42195892 | 22.73421669 | 22.71007156 | 22.55999184 |
|  | 1.028426474 | -0.262640635 | Q9JIK5 | Nucleolar RNA helicase 2 | Ddx21 | 22.37028694 | 22.28144264 | 22.18649483 | 22.69565964 | 22.59559631 | 22.33489037 |
|  | 1.360891539 | -0.259466171 | Q61655;Q9QY15 | ATP-dependent RNA helicase DDX19A | Ddx19a | 23.4582634 | 23.30952454 | 23.38165855 | 23.48836517 | 23.74046898 | 23.69901085 |
|  | 0.499572828 | -0.257436117 | P11031 | Activated RNA polymerase II transcriptional coactivator p15 | Sub1 | 22.4342556 | NaN | 21.88375282 | 22.51400375 | 22.36035347 | 22.37496376 |
|  | 0.635924195 | -0.257411321 | O35955 | Proteasome subunit beta type-10 | Psmb10 | 22.50210381 | 22.00672531 | 22.18643379 | 22.41578484 | 22.70752525 | 22.34418678 |
|  | 1.385618949 | -0.257222493 | P41241;A0A1L1SR46;A0A1L1STA1;A0A1L1SQQ5;D3YVQ8;D6RGA0;A0A0R4J1P8;D3Z4T5;A0A0R4J1N6;P41242 | Tyrosine-protein kinase CSK | Csk | 23.33429146 | 23.16346169 | 23.05540085 | 23.48443794 | 23.45701027 | 23.38337326 |
|  | 1.296671738 | -0.256535848 | E9Q0G1;Q9CQJ6 | Density-regulated protein | Denr | 23.23167801 | 23.07339287 | 23.19290352 | 23.35191536 | 23.49313927 | NaN |
|  | 0.743509259 | -0.253213247 | P26350;A0A087WP98;A0A087WPN6;A0A087WQN2 | Prothymosin alpha;Prothymosin alpha, N-terminally processed;Thymosin alpha | Ptma | 26.93977356 | 26.5553627 | 26.57252121 | 27.05486298 | 27.01520729 | 26.75722694 |
|  | 0.578023823 | -0.253079732 | Q3TFQ1;Q3UBW1 | SPRY domain-containing protein 7 | Spryd7 | 21.43415451 | 21.49057007 | 21.40660095 | 21.59905243 | 22.06999016 | 21.42152214 |
|  | 0.8345081 | -0.249040604 | Q4VBE8 | WD repeat-containing protein 18 | Wdr18 | 21.6132431 | 21.1487999 | 21.32207108 | 21.63458061 | 21.64257813 | 21.55407715 |
|  | 0.872866787 | -0.24779892 | Q64521;A2AQR0 | Glycerol-3-phosphate dehydrogenase, mitochondrial;Glycerol-3-phosphate dehydrogenase | Gpd2 | 21.61593819 | 21.64763832 | 21.86370468 | 22.01338577 | 21.75029564 | 22.10699654 |
|  | 0.639480901 | -0.246870995 | Q80U93;A0A0A6YW83 | Nuclear pore complex protein Nup214 | Nup214 | NaN | 21.33941078 | 21.39519119 | 21.47253799 | NaN | 21.75580597 |
|  | 0.991666879 | -0.246218999 | P47713;Q9DBX5;A0A087WPN8 | Cytosolic phospholipase A2;Phospholipase A2;Lysophospholipase | Pla2g4a | 22.47894478 | 22.37597084 | 22.46405983 | 22.72404861 | 22.47580719 | 22.85777664 |
|  | 0.489431513 | -0.245510101 | Q9Z1A1;F6QJV5;B8JJG7;B8JJG8;B8JJG9 |  | Tfg | 23.49472618 | 23.30257034 | 23.90765762 | 23.91250801 | 23.96525574 | 23.5637207 |
|  | 0.594725025 | -0.243889491 | A0A140LHA2;Q9WVA3;A0A140LJ21;A0A140LI47;A0A140LIM5 | Mitotic checkpoint protein BUB3 | Bub3 | 22.30710793 | 22.58364105 | 22.91312981 | 22.84724236 | 22.75027657 | 22.93802834 |
|  | 0.705950068 | -0.243641535 | Q61074;A0A0J9YVG0;A0A0J9YV96;A0A0J9YUB0 | Protein phosphatase 1G | Ppm1g | 22.99481201 | 22.88924217 | 22.96634865 | 22.94508362 | 23.15981865 | 23.47642517 |
|  | 1.629062436 | -0.241892497 | Q9Z0G0;A0A1D5RML2 | PDZ domain-containing protein GIPC1 | Gipc1 | 22.45332336 | 22.38592911 | 22.4986763 | 22.59675598 | 22.79954338 | 22.6673069 |
|  | 1.123963203 | -0.239780426 | Q9Z0L8 | Gamma-glutamyl hydrolase | Ggh | 24.91195869 | 24.63295174 | 24.83253288 | 24.93548584 | 25.13072968 | 25.03056908 |
|  | 0.788934024 | -0.238329569 | Q9ES74;Q3TN15 | Serine/threonine-protein kinase Nek7 | Nek7 | 22.34025383 | 22.36434174 | 22.37148476 | 22.77177048 | 22.32215309 | 22.69714546 |
|  | 0.613267661 | -0.238135338 | P35991;A2BDW0 | Tyrosine-protein kinase BTK | Btk | 23.2353344 | 23.51267815 | 23.07696915 | 23.50348854 | 23.52276993 | NaN |
|  | 0.858859664 | -0.236787796 | Q9WVE8;A0A2R8W6S4;A0A2R8W750;Q3UP40;A0A338P6P7 | Protein kinase C and casein kinase substrate in neurons protein 2 | Pacsin2 | 23.70260811 | 23.31920624 | 23.54427528 | 23.83132362 | 23.81323242 | 23.63189697 |
|  | 1.231468722 | -0.234312693 | Q64133 | Amine oxidase [flavin-containing] A | Maoa | 23.74663162 | 23.89439583 | 23.78516579 | 23.93048668 | 24.00667381 | 24.19197083 |
|  | 1.06378908 | -0.231079102 | P70404 | Isocitrate dehydrogenase [NAD] subunit gamma 1, mitochondrial | Idh3g | 24.08707428 | 24.04501915 | 24.00127029 | 24.10413551 | 24.27526093 | 24.44720459 |
|  | 1.194837066 | -0.229915619 | E9Q7G0;F6ZQA3 | Nuclear mitotic apparatus protein 1 | Numa1 | 22.4126091 | 22.63920403 | 22.37488365 | 22.7285614 | 22.63307381 | 22.75480843 |
|  | 1.187377234 | -0.229403496 | E9PV14;A2AUK7;A2AUK8;A2AUK5;Q9Z2H5;A0A2R8VHB6 | Band 4.1-like protein 1 | Epb4.1l1;Epb41l1 | 22.88069344 | 23.06569099 | 23.02086258 | 23.17026329 | NaN | 23.26670837 |
|  | 0.561574716 | -0.229330699 | Q8BH02;A0A0A6YWQ0 | Torsin-4A | Tor4a | 22.24982834 | 21.87867355 | NaN | 22.34201813 | 22.13899231 | 22.3997345 |
|  | 0.584942876 | -0.229212443 | Q9QUJ7 | Long-chain-fatty-acid--CoA ligase 4 | Acsl4 | 22.8892231 | 22.71510887 | 22.38316154 | 22.90811539 | 23.04288292 | 22.72413254 |
|  | 1.422433141 | -0.227718353 | Q9DBS1 | Transmembrane protein 43 | Tmem43 | 24.52432442 | 24.69083023 | 24.50706291 | 24.79352379 | 24.72650337 | 24.88534546 |
|  | 1.346231483 | -0.227515539 | O35551;J3QJV7;Q3U983 | Rab GTPase-binding effector protein 1 | Rabep1 | 22.4498024 | 22.29277611 | 22.44268227 | 22.56634712 | 22.55854416 | 22.74291611 |
|  | 0.688849856 | -0.227005641 | O35379;D3YZY1;E9Q1I5 | Multidrug resistance-associated protein 1 | Abcc1 | 22.61741829 | 22.40657616 | 22.14426231 | 22.73842812 | 22.56713676 | 22.5437088 |
|  | 1.565736354 | -0.225606283 | P47963 | 60S ribosomal protein L13 | Rpl13 | 26.95049286 | 26.87572479 | 26.91479111 | 27.0305481 | 27.24762344 | 27.13965607 |
|  | 1.655056801 | -0.224538167 | P61222 | ATP-binding cassette sub-family E member 1 | Abce1 | 23.74232101 | 23.78046608 | 23.71156311 | 23.85595322 | 24.00075531 | 24.05125618 |
|  | 1.322778073 | -0.222253164 | Q3TQP6;P06801 | Malic enzyme;NADP-dependent malic enzyme | Me1 | 22.68189621 | 22.73152542 | 22.76555252 | 22.98039818 | 22.80605125 | 23.05928421 |
|  | 1.11603798 | -0.222115199 | P58021;E9PZ69 | Transmembrane 9 superfamily member 2 | Tm9sf2 | 22.90699768 | 22.99698639 | 23.21639442 | 23.24302864 | 23.29782104 | 23.2458744 |
|  | 0.780177757 | -0.22079277 | P70677;A0A1B0GRX1 | Caspase-3;Caspase-3 subunit p17;Caspase-3 subunit p12 | Casp3 | NaN | 22.70047188 | 22.48816872 | 22.67327309 | 22.8783741 | 22.89369202 |
|  | 0.586475692 | -0.219220161 | Q9QZM0;Q99NB8 | Ubiquilin-2 | Ubqln2 | NaN | 22.99765778 | 22.72238159 | NaN | 23.05087471 | 23.10760498 |
|  | 0.389650179 | -0.218600591 | Q8R1I1 | Cytochrome b-c1 complex subunit 9 | Uqcr10 | 25.06574821 | 24.85224342 | 24.48578835 | 24.90540886 | 24.80877304 | 25.34539986 |
|  | 1.762675169 | -0.217006048 | Q9Z1Z2 | Serine-threonine kinase receptor-associated protein | Strap | 23.71040726 | 23.82948685 | 23.67798805 | 23.99955177 | 23.97254944 | 23.89679909 |
|  | 0.969737533 | -0.215169271 | Q3UHX2 | 28 kDa heat- and acid-stable phosphoprotein | Pdap1 | 23.0582943 | 23.26198387 | 23.03650856 | 23.27682304 | 23.48308372 | 23.24238777 |
|  | 1.625127747 | -0.214028041 | Q9D8S5;O35326 | Serine/arginine-rich splicing factor 5 | Srsf5 | 23.76819992 | 23.81431007 | 23.6365509 | 23.90407753 | 24.00135612 | 23.95571136 |
|  | 0.795305787 | -0.21265475 | Q9D8T2;A0A2R8VKQ7 | Gasdermin-D | Gsdmdc1 | 23.02580452 | 23.31409836 | 22.9185257 | 23.33388138 | 23.33592606 | 23.22658539 |
|  | 1.490650586 | -0.212161382 | P62889;A0A2I3BQF4 | 60S ribosomal protein L30 | Rpl30 | 26.22409248 | 26.05655861 | 26.14452744 | 26.38035393 | 26.26660156 | 26.41470718 |
|  | 1.746596376 | -0.211695989 | Q7TSI3;A0A0U1RQ86;A0A0U1RNI2 | Serine/threonine-protein phosphatase 6 regulatory subunit 1 | Ppp6r1 | 22.58701134 | 22.5402832 | 22.45289612 | 22.80524445 | 22.73620033 | 22.67383385 |
|  | 0.581701035 | -0.210552851 | Q8CHP8 | Phosphoglycolate phosphatase | Pgp | 23.16622353 | 22.82675552 | 22.83389282 | 23.38311005 | 23.06496811 | 23.01045227 |
|  | 1.685026372 | -0.209977468 | Q8K297 | Procollagen galactosyltransferase 1 | Colgalt1 | 23.05110741 | 22.96982956 | 22.96310425 | 23.28855705 | 23.11876678 | 23.20664978 |
|  | 0.984992319 | -0.208286921 | D3Z191;Q80X71;D3Z6E0;D3Z0M2 | Transmembrane protein 106B | Tmem106b | 22.65602303 | 22.66120911 | 22.52245712 | 22.82310486 | 22.66810799 | 22.97333717 |
|  | 0.700546907 | -0.205207825 | P47758;A0A1W2P830 | Signal recognition particle receptor subunit beta | Srprb | 22.89471054 | 22.91221619 | 22.78871346 | 22.89596748 | 23.31962013 | 22.99567604 |
|  | 0.99862327 | -0.204848607 | Q9CZE3;A0A1Y7VIZ0;A0A140LHK2;E9QLQ7;Q91YQ1;Q8QZZ8 | Ras-related protein Rab-32 | Rab32 | 24.17850876 | 24.01826668 | 24.24154472 | 24.28369141 | 24.49008179 | 24.27909279 |
|  | 0.756928499 | -0.204150518 | Q60710;F6TVP2;E9PYG9 | Deoxynucleoside triphosphate triphosphohydrolase SAMHD1 | Samhd1 | 23.00025749 | 23.11327553 | 23.00627899 | 23.01428795 | 23.40872765 | 23.30924797 |
|  | 1.61816919 | -0.204142253 | Q9D662;A2ANA0;A2AN97;A2AN98;A2AN99 | Protein transport protein Sec23B | Sec23b | 23.5148468 | 23.5666256 | 23.40106392 | 23.75715065 | 23.65381622 | 23.6839962 |
|  | 1.104021429 | -0.203617732 | Q501J6;Q3U741;Q3TU25 | Probable ATP-dependent RNA helicase DDX17 | Ddx17 | 23.74796295 | 23.98667717 | 23.72088051 | 23.98450661 | 24.0513401 | 24.03052711 |
|  | 0.789501038 | -0.202615102 | E9QA74;E9Q405;A0A1C7ZN10;K3W4L0;B2RRE2;E9QAX2;Q9JMH9 | Unconventional myosin-XVIIIa | Myo18a | 22.71686745 | 22.44745827 | 22.7698555 | 22.83840179 | 22.74157906 | 22.96204567 |
|  | 0.764401761 | -0.202119827 | Q9JKX6;A2ATT5;A0A0A6YVU1 | ADP-sugar pyrophosphatase | Nudt5 | 22.80689621 | 22.78282738 | 22.71826935 | 23.11381531 | 23.06493568 | 22.73560143 |
|  | 1.717325159 | -0.201915741 | Q64281;A0A1W2P6F1;A0A1W2P7X1 | Leukocyte immunoglobulin-like receptor subfamily B member 4 | Lilrb4 | 25.28781891 | 25.30302238 | 25.41705894 | 25.48099136 | 25.53359222 | 25.59906387 |
|  | 1.247119326 | -0.200087865 | P63321;A0A1Y7VL93 | Ras-related protein Ral-A | Rala | 24.58623314 | 24.61969376 | 24.81601906 | 24.83532906 | 24.91547394 | 24.87140656 |
|  | 0.755352355 | -0.199367523 | Q3UPV6;P62482;E0CXZ9;A0A571BGH0;A0A571BF54;A0A571BEM7;E0CXI4;A0A5F8MPA4;A0A5F8MPK0;P63143;E0CYS2;A0A571BFX6;A0A571BE49 | Voltage-gated potassium channel subunit beta-2 | Kcnab2 | 24.4738884 | 24.78056717 | 24.72499657 | 24.86679268 | 24.72343445 | 24.98732758 |
|  | 2.085031357 | -0.199117661 | P62242 | 40S ribosomal protein S8 | Rps8 | 26.30627823 | 26.21495247 | 26.18818283 | 26.44542313 | 26.46397972 | 26.39736366 |
|  | 1.027200003 | -0.199063619 | P62301;Q921R2;A0A0U1RQ71 | 40S ribosomal protein S13 | Rps13 | 25.64737892 | 25.73277855 | 25.71444893 | 26.06379509 | 25.85891914 | 25.76908302 |
|  | 2.009521422 | -0.198209763 | O35604 | Niemann-Pick C1 protein | Npc1 | 24.98836899 | 24.97609329 | 25.00380325 | 25.22468185 | 25.23443604 | 25.10377693 |
|  | 0.6018664 | -0.197881063 | E9QN99;A0A087WPF8;A0A087WSR2;A0A087WRJ2;Q8VCR7;A0A1L1SUX1;A0A087WP24;A0A1L1SRH5 | Alpha/beta hydrolase domain-containing protein 14B | Abhd14b | 24.23534966 | 24.04909706 | 24.37464523 | 24.52043343 | 24.19174576 | 24.54055595 |
|  | 0.790907126 | -0.19633166 | A0A1B0GT56;P51829 | Adenylate cyclase type 7 | Adcy7 | 21.3658638 | 21.28765678 | 21.53748894 | 21.67844009 | 21.68358994 | 21.41797447 |
|  | 0.711685631 | -0.194953918 | A0A0R4J079;Q8BMP6 | Golgi resident protein GCP60 | Acbd3 | 23.39401436 | 23.42059708 | 23.25551414 | 23.32443619 | 23.64548492 | 23.68506622 |
|  | 1.959965779 | -0.194624583 | Q09014;F8WH69;S4R293 | Neutrophil cytosol factor 1 | Ncf1 | 25.01256752 | 24.97701073 | 25.05179596 | 25.19005013 | 25.15433502 | 25.28086281 |
|  | 2.196582252 | -0.194258372 | P62849;A0A286YEB7 | 40S ribosomal protein S24 | Rps24 | 25.64498901 | 25.63870621 | 25.61464691 | 25.77057076 | 25.81677628 | 25.89377022 |
|  | 2.420695335 | -0.191214879 | P05063 | Fructose-bisphosphate aldolase C | Aldoc | 24.45813751 | 24.45801353 | 24.49185562 | 24.60896492 | 24.71151161 | 24.66117477 |
|  | 1.010332806 | -0.190897624 | Q6ZWQ7 | Signal peptidase complex subunit 3 | Spcs3 | 24.192873 | 24.03992653 | 24.04984474 | 24.28806496 | 24.15614891 | 24.41112328 |
|  | 0.548196807 | -0.188034058 | Q76LS9 | Protein FAM63A | Fam63a | 23.67949295 | 23.34380722 | 23.85395622 | 23.76880455 | 23.81821632 | 23.85433769 |
|  | 0.49859952 | -0.187161128 | Q8VBV7;A0A087WPM5 | COP9 signalosome complex subunit 8 | Cops8 | 22.8521862 | 22.66392517 | 22.32942581 | 22.91734314 | 22.76906776 | 22.72060966 |
|  | 0.813170396 | -0.186315854 | Q8JZR2;Q5ND50;Q64010;Q3TQV3;F7D232 | Adapter molecule crk | Crk | 23.28729057 | 23.1817112 | 23.416996 | NaN | 23.54026031 | 23.42303658 |
|  | 1.059749413 | -0.18320783 | E9Q1G8;E9Q9F5;A0A0R4J233;A0A1W2P6J7;Q8C650 | Septin-10 | Septin10 | 24.63848495 | 24.81063652 | 24.78606224 | 25.01699257 | 24.8116169 | 24.95619774 |
|  | 0.568013351 | -0.183164597 | Q91YP3;A0A0N4SV34 | Deoxyribose-phosphate aldolase | Dera | 22.7382431 | 22.62882996 | NaN | 22.97507095 | 22.7583313 | NaN |
|  | 1.428743702 | -0.182628632 | Q921Y0;Q3UDM0;Q8BPB0 | MOB kinase activator 1A;MOB kinase activator 1B | Mob1a;Mob1b | 23.49594688 | 23.66665649 | 23.56825066 | 23.8179245 | 23.70366478 | 23.75715065 |
|  | 1.031626757 | -0.182048798 | Q6PGH2 | Hematological and neurological expressed 1-like protein | Hn1l | 23.60648155 | 23.52312851 | 23.43783951 | 23.57149506 | 23.75633621 | 23.78576469 |
|  | 1.247133841 | -0.182021459 | P60867 | 40S ribosomal protein S20 | Rps20 | 26.07777596 | 26.22771454 | 26.09804344 | 26.29430199 | 26.41189766 | 26.24339867 |
|  | 0.578330985 | -0.181706111 | P63328;E9Q6P2;G3X8U7;E0CZ78;P48453 | Serine/threonine-protein phosphatase 2B catalytic subunit alpha isoform | Ppp3ca | 23.55039406 | 23.81860542 | 23.77685738 | 23.84900093 | 24.11131859 | 23.73065567 |
|  | 1.408187438 | -0.180880864 | E9QL31;A0A0R4J104;P98078;E9PX84;Q3TRE6;F6TQN9;Q9DCE6;E0CXT5;E0CZ53;E0CYJ2 | Disabled homolog 2 | Dab2 | 24.54521751 | 24.66649437 | 24.74565697 | 24.81293869 | 24.82803345 | 24.85903931 |
|  | 0.889044181 | -0.180719376 | Q8R146;A0A0R4J107;A0A0A6YXV0 | Acylamino-acid-releasing enzyme | Apeh | 23.72213173 | 23.74693871 | 23.71512985 | 23.83113098 | 23.79887199 | 24.09635544 |
|  | 0.268370916 | -0.180705388 | O88696 | ATP-dependent Clp protease proteolytic subunit, mitochondrial | Clpp | 23.75276947 | 23.58703423 | 23.22203636 | NaN | 23.92324829 | 23.47938919 |
|  | 1.712676908 | -0.18052419 | D3YWR7;Q8BVI4;A0A0G2JGY0;A0A0G2JGJ1;D3Z1A1;D3Z099 | Dihydropteridine reductase | Qdpr | 25.18461227 | 25.16495895 | 25.04693413 | 25.35269165 | 25.28347969 | 25.30190659 |
|  | 0.785926183 | -0.179804484 | Q9ESP1 | Stromal cell-derived factor 2-like protein 1 | Sdf2l1 | 24.24858284 | 24.19775581 | 24.30598259 | 24.47097588 | 24.23928833 | 24.58147049 |
|  | 0.750425062 | -0.178898493 | P61290;A2A4J1;A2A4J3 | Proteasome activator complex subunit 3 | Psme3 | 22.75712967 | 22.60079956 | 22.94415474 | 23.02079582 | 22.86151123 | 22.9564724 |
|  | 1.173026903 | -0.178795497 | O09005 | Sphingolipid delta(4)-desaturase DES1 | Degs1 | 23.76222992 | 23.85176659 | 23.65468979 | 24.01563263 | 23.92297745 | 23.86646271 |
|  | 0.911232699 | -0.178578059 | P61750;E9Q798;F6UFB9;D3YV25;E9Q2C2 | ADP-ribosylation factor 4 | Arf4 | 24.80577469 | 25.02453613 | 25.05187988 | 25.11414146 | 25.23213196 | 25.07165146 |
|  | 0.733669985 | -0.178459803 | Q06138;Q9DB16 | Calcium-binding protein 39 | Cab39 | 23.47901917 | 23.47914124 | 23.17125702 | 23.53824806 | 23.48922348 | 23.63732529 |
|  | 0.484420253 | -0.17804273 | G3UYQ2;A0A1B0GX81;G3UZT6;Q3UF95;Q9Z1R2;G3UXT8;G3UYZ0;G3V013;S4R183;S4R224 | Large proline-rich protein BAG6 | Bag6 | 23.04261589 | 22.6923008 | 23.15813828 | 23.08457947 | 23.04758453 | 23.29501915 |
|  | 1.789744442 | -0.176629384 | O09167 | 60S ribosomal protein L21 | Rpl21 | 26.65853691 | 26.58118439 | 26.63851357 | 26.87783241 | 26.77026749 | 26.76002312 |
|  | 0.112723543 | -0.176172256 | A0A0G2JG95;Q8BX10 | Serine/threonine-protein phosphatase PGAM5, mitochondrial | Pgam5 | 22.63072014 | 21.60476303 | NaN | 22.42910385 | 22.15872383 | NaN |
|  | 0.464939645 | -0.175317128 | A0A0R4J0D3;Q3TDQ1 | Dolichyl-diphosphooligosaccharide--protein glycosyltransferase subunit STT3B | Stt3b | 22.99291229 | 23.33073997 | 23.44556427 | 23.53954887 | 23.25261688 | 23.50300217 |
|  | 2.434513667 | -0.173693975 | P97351 | 40S ribosomal protein S3a | Rps3a | 26.50554848 | 26.53687096 | 26.52243996 | 26.69458199 | 26.7425251 | 26.64883423 |
|  | 1.112329334 | -0.17317009 | Q9JI75;A0A1Y7VMA0;A0A1Y7VL95 | Ribosyldihydronicotinamide dehydrogenase [quinone] | Nqo2 | 23.10886383 | 23.1479454 | 23.21225739 | 23.21102524 | 23.33565331 | 23.44189835 |
|  | 0.566548786 | -0.173123042 | Q8BTI8 | Serine/arginine repetitive matrix protein 2 | Srrm2 | 23.17004967 | 23.14219093 | 23.30535507 | 23.46051407 | 23.54486465 | 23.13158607 |
|  | 0.696544534 | -0.172892253 | Q9ERB0 | Synaptosomal-associated protein 29 | Snap29 | 22.65473366 | 22.64587975 | 22.67398453 | 22.60688782 | 22.96504402 | 22.92134285 |
|  | 0.339470037 | -0.172631582 | P97315 | Cysteine and glycine-rich protein 1 | Csrp1 | 24.9471283 | 25.26674461 | 24.87314415 | 25.52954674 | 24.94730759 | 25.12805748 |
|  | 0.559449218 | -0.172606786 | P62274 | 40S ribosomal protein S29 | Rps29 | 25.0527916 | 25.11012459 | 25.09026909 | 25.38633728 | 24.98546219 | 25.39920616 |
|  | 1.68430177 | -0.171789805 | A0A1Y7VJ48;P48428 | Tubulin-specific chaperone A | Tbca | 24.49856567 | 24.38495445 | 24.42930794 | 24.6118927 | 24.66432571 | 24.55197906 |
|  | 2.474808879 | -0.169961929 | P62908;D3YV43;A0A140LI77 | 40S ribosomal protein S3 | Rps3 | 26.42934036 | 26.43542671 | 26.45809174 | 26.57571411 | 26.66112137 | 26.59590912 |
|  | 0.471145363 | -0.169216474 | O35465 | Peptidyl-prolyl cis-trans isomerase FKBP8 | Fkbp8 | 23.48000526 | 23.26298714 | 23.08321571 | 23.46351051 | NaN | 23.42572784 |
|  | 0.4457506 | -0.168838819 | A0A1B0GRG3;A0A1B0GSK8;O35566 | CD151 antigen | Cd151 | NaN | 21.25971603 | 21.28765678 | 21.29451561 | 21.35169983 | 21.68136024 |
|  | 0.472770401 | -0.168750763 | Q9JJI8 | 60S ribosomal protein L38 | Rpl38 | 25.63549995 | 25.69508553 | 25.96088982 | 26.07204056 | 25.69789886 | 26.02778816 |
|  | 0.697797582 | -0.168003082 | Q64674 | Spermidine synthase | Srm | 23.42085457 | 23.53599548 | 23.65589142 | 23.59900665 | 23.87633324 | 23.64141083 |
|  | 1.506037755 | -0.167792002 | P54923 | [Protein ADP-ribosylarginine] hydrolase | Adprh | 23.76405334 | 23.72536087 | 23.88278389 | 23.94859886 | 23.99757195 | 23.92940331 |
|  | 0.182937898 | -0.167349815 | P97333 | Neuropilin-1 | Nrp1 | NaN | 21.96652412 | 22.30089569 | 21.94563866 | 22.1867981 | 22.77074242 |
|  | 0.705929613 | -0.167270025 | A0A494BAN1;A0A494BA56;A0A0G2JE32;A0A0G2JGL0;P62838;P61079;P61080;Q6ZWY6 | Ubiquitin-conjugating enzyme E2 D2;Ubiquitin-conjugating enzyme E2 D3;Ubiquitin-conjugating enzyme E2 D1;Ubiquitin-conjugating enzyme E2 D2B | Ube2d2;Ube2d3;Ube2d1;Ube2d2b | 25.51029587 | 25.28390312 | 25.3530941 | 25.57850838 | 25.39037704 | 25.68021774 |
|  | 0.706019564 | -0.166367849 | E9Q421;Q8BXX3;Q8BL63;Q9CXY9 | GPI-anchor transamidase | Pigk | 22.85477448 | 22.59454918 | 22.93842316 | 22.9629631 | 23.01231194 | 22.91157532 |
|  | 1.490880882 | -0.165608724 | Q62419;A0A3B2W7K0;A2ALV1;Q8BXU5;A2ALV3;Q62420 | Endophilin-A2 | Sh3gl1 | 23.56267357 | 23.45148849 | 23.50603294 | 23.65294075 | 23.61447906 | 23.74960136 |
|  | 0.226364551 | -0.164881706 | A0A0U1RQA5;A0A0U1RP20;A0A0U1RQA0;O35316 | Sodium- and chloride-dependent taurine transporter | Slc6a6 | 23.56534767 | NaN | 24.06874466 | 23.90820694 | 24.0556488 | NaN |
|  | 1.624306851 | -0.164745331 | P62911 | 60S ribosomal protein L32 | Rpl32 | 26.05519295 | 26.0434761 | 26.09313393 | 26.16802216 | 26.20405769 | 26.31395912 |
|  | 0.967274788 | -0.164621353 | A0A571BEI2;Q6PIU9 | Uncharacterized protein FLJ45252 homolog |  | 22.71795654 | 22.48377419 | 22.48505211 | 22.75849342 | 22.69954109 | 22.72261238 |
|  | 0.340907652 | -0.16461436 | A2AF47;A2AF67;A2AF65;A0A1D5RLE0;A0A5F8MPL9;E9QMR2;A0A1D5RMM1;F8VPN7;Q8BIK4 | Dedicator of cytokinesis protein 11 | Dock11 | 22.38763809 | 22.4793644 | 22.26616478 | 22.49711609 | 22.89159775 | 22.23829651 |
|  | 0.888802178 | -0.164040883 | E9Q4Q2;D3YZC9;D3YZD0;Q64213;D3YVH4;F8WHF9;D6RDB7 | Splicing factor 1 | Sf1 | 23.18871498 | 23.13354683 | 23.30702591 | 23.51050758 | 23.28742981 | 23.32347298 |
|  | 1.431831603 | -0.163915634 | H7BX95;Q6PDM2;F7AI47;F6QXN3 | Serine/arginine-rich splicing factor 1 | Srsf1 | 24.01128769 | 23.98919296 | 24.05870628 | 24.27788734 | 24.11235237 | 24.16069412 |
|  | 0.992461207 | -0.163277308 | Q9JK81;F8WGG3;F7A3N3 | UPF0160 protein MYG1, mitochondrial | Myg1 | 23.10536766 | 23.03331947 | 22.85570717 | 23.1310215 | 23.20212746 | 23.15107727 |
|  | 1.714667599 | -0.162660599 | Q8K2B3;A0A1Y7VJ55 | Succinate dehydrogenase [ubiquinone] flavoprotein subunit, mitochondrial | Sdha | 23.77414513 | 23.81967926 | 23.70956612 | 23.97980499 | 23.93111801 | 23.8804493 |
|  | 0.65590724 | -0.162522634 | P54823 | Probable ATP-dependent RNA helicase DDX6 | Ddx6 | 23.15675163 | 23.16871834 | 23.41015244 | 23.33005524 | 23.33360863 | 23.55952644 |
|  | 0.66526967 | -0.161612829 | F6SLP4;A0A494BAW5;A0A494BAK4;Q9DC16;A0A494BAX4 | Endoplasmic reticulum-Golgi intermediate compartment protein 1 | Ergic1 | 23.08872604 | 22.79536819 | 22.79899025 | 23.03111649 | 22.98132133 | 23.15548515 |
|  | 1.01197261 | -0.161567688 | Q8C052;A0A1D5RLY6;A0A1D5RMG7 | Microtubule-associated protein 1S;MAP1S heavy chain;MAP1S light chain | Map1s | 22.78733826 | 22.73516846 | 22.56264877 | 22.82065201 | 22.82940865 | 22.9197979 |
|  | 1.058491753 | -0.161445618 | Q91YS8;D3Z368 | Calcium/calmodulin-dependent protein kinase type 1 | Camk1 | 24.2908783 | 24.11735535 | 24.32182312 | 24.45870209 | 24.3448925 | 24.41079903 |
|  | 0.668871099 | -0.160994848 | A0A1B0GRV0;Q9Z0S1;D3Z0E6;D3Z5X0 | 3(2),5-bisphosphate nucleotidase 1 | Bpnt1 | 22.86944008 | 22.92902374 | 22.84613228 | 23.20386124 | 23.08191681 | 22.8418026 |
|  | 0.738804335 | -0.160158157 | P68134;P68033;P63268;P62737;A0A1D5RM20;D3Z2K3;A0A494B9T3;D3YZY0 | Actin, alpha skeletal muscle;Actin, alpha cardiac muscle 1;Actin, gamma-enteric smooth muscle;Actin, aortic smooth muscle | Acta1;Actc1;Actg2;Acta2 | 31.01710701 | 30.70742607 | 30.77541161 | 31.01045227 | 31.0392437 | 30.93072319 |
|  | 0.516707424 | -0.158777237 | Q9ERU9 | E3 SUMO-protein ligase RanBP2 | Ranbp2 | 22.56994438 | 22.66765404 | 22.67766571 | 23.03207588 | 22.77792168 | 22.58159828 |
|  | 1.232513315 | -0.15852801 | P35980;A0A1B0GSS8;A0A1B0GQU8;A0A1B0GSF7;A0A1B0GSA8;A0A1B0GS28;A0A1B0GRZ3 | 60S ribosomal protein L18 | Rpl18 | 26.49952507 | 26.5036087 | 26.54666138 | 26.78930092 | 26.64016914 | 26.59590912 |
|  | 0.429404608 | -0.158456802 | P30355;A0A0J9YUC8 | Arachidonate 5-lipoxygenase-activating protein | Alox5ap | NaN | 27.32526016 | 27.42894173 | NaN | 27.66438103 | 27.40673447 |
|  | 0.967682935 | -0.15838178 | A0A3Q4EI12;P35293;A0A452J8C1;A0A3Q4EIF7 | Ras-related protein Rab-18 | Rab18 | 25.17352295 | 25.02732468 | 25.18120384 | 25.39136124 | 25.27469254 | 25.19114304 |
|  | 1.355700648 | -0.158312798 | Q9CR26;F8WJC2;F6W5Q8 | Vacuolar protein sorting-associated protein VTA1 homolog | Vta1 | 22.93091965 | 23.02492523 | 22.96432114 | 23.0898571 | 23.17354584 | NaN |
|  | 0.705093372 | -0.157978058 | Q8CGA3 | Large neutral amino acids transporter small subunit 4 | Slc43a2 | 22.68468094 | 22.68754578 | 22.41147232 | 22.78813553 | 22.8073101 | 22.66218758 |
|  | 1.063188575 | -0.157773972 | Q9D0F9;A2CEK3 | Phosphoglucomutase-1 | Pgm1;Pgm2 | 23.54745483 | 23.72858238 | 23.52528 | 23.80390549 | 23.75959015 | 23.71114349 |
|  | 0.98141079 | -0.157312393 | Q9EP89;A0A1L1SVF9 | Serine beta-lactamase-like protein LACTB, mitochondrial | Lactb | 22.92596817 | 22.81638145 | NaN | 23.03553581 | 23.0214386 | NaN |
|  | 1.283348216 | -0.157272339 | P70372 | ELAV-like protein 1 | Elavl1 | 24.22949409 | 24.11822701 | 24.11155701 | 24.39538765 | 24.27767372 | 24.25803375 |
|  | 2.526914236 | -0.156103134 | Q9EQP2 | EH domain-containing protein 4 | Ehd4 | 24.86830139 | 24.79009628 | 24.81499481 | 24.99489975 | 24.97088242 | 24.97591972 |
|  | 0.544447022 | -0.155989329 | Q9CQ22;A0A0A6YX02 | Ragulator complex protein LAMTOR1 | Lamtor1 | 24.57166862 | 24.1882782 | 24.26756668 | 24.58617592 | 24.41751099 | 24.49179459 |
|  | 0.899591727 | -0.154876709 | P97372;G3X9V0;E0CZ90 | Proteasome activator complex subunit 2 | Psme2 | 24.73789215 | 24.7683506 | 24.67411423 | 24.91993523 | 24.73629379 | 24.98875809 |
|  | 2.30141292 | -0.154844284 | P62702 | 40S ribosomal protein S4, X isoform | Rps4x | 26.26640511 | 26.31620598 | 26.26819038 | 26.42271614 | 26.48251534 | 26.41010284 |
|  | 1.582827985 | -0.154055913 | O89017 | Legumain | Lgmn | 25.26359558 | 25.22276688 | 25.33279037 | 25.37026024 | 25.47735214 | 25.43370819 |
|  | 0.496024181 | -0.153869629 | A2AQ43;A2AQ44;A2AQ42;A2AQ45;A2AQ41;Q80TY0;A0A0A6YWT1;F6VVN1;A2AQ39;A2AQ47 | Formin-binding protein 1 | Fnbp1 | 23.44758415 | 23.51255798 | 23.40444565 | 23.82114029 | 23.63732529 | 23.36773109 |
|  | 1.923635883 | -0.153443654 | F8WJK8;Q99L47;E9Q1V0;E9Q1X9 | Hsc70-interacting protein | St13 | 25.04839134 | 25.09900665 | 25.0771637 | 25.18173409 | 25.21389771 | 25.28926086 |
|  | 0.519875961 | -0.153137207 | A0A6I8MX27;P16125;D3Z7F0;A0A0N4SVV8 | L-lactate dehydrogenase B chain;L-lactate dehydrogenase | Ldhb | 22.45033073 | 22.64449501 | 22.2515049 | 22.48519897 | 22.62305832 | 22.69748497 |
|  | 1.527391976 | -0.152693431 | Q8K2C7 | Protein OS-9 | Os9 | 22.27545929 | 22.28895187 | 22.22796631 | 22.43249893 | 22.33701706 | 22.48094177 |
|  | 0.547279126 | -0.151587804 | A0A0A6YXE3;Q9JKP5;G3X9Q0;A0A0A6YVV8;A0A0A6YWB0;A0A0A6YXP3;A0A2K6EDM5;Q8C181;Q3U581;A0A0A6YWG1;Q3U570;A0A0A6YXQ4;A0A0A6YXL7;S4R267;A0A2I3BRX8;A0A0A6YWJ5;Q8R003 | Muscleblind-like protein 1;Muscleblind-like protein 2 | Mbnl1;Mbnl2 | 24.56592941 | 24.86693382 | 24.60082245 | 24.81357574 | 24.97057533 | 24.70429802 |
|  | 0.692758195 | -0.151506424 | Q91WK2 | Eukaryotic translation initiation factor 3 subunit H | Eif3h | 24.8511467 | 24.61503983 | 24.53658867 | 24.84985924 | 24.75633621 | 24.85109901 |
|  | 0.565751863 | -0.151395798 | Q61249;A0A0B4J1F7;Q9QZ29 | Immunoglobulin-binding protein 1 | Igbp1 | 22.81520844 | 22.46281242 | 22.47276115 | 22.6919384 | 22.78384781 | 22.7291832 |
|  | 0.763712635 | -0.151278178 | P61027;Q9DD03;D3YUS4 | Ras-related protein Rab-10 | Rab10 | 25.28132248 | 25.11779213 | 25.07549286 | 25.21682358 | 25.27415848 | 25.43745995 |
|  | 0.445773774 | -0.150595983 | P35831;F6Z0X5;D6RGT2 | Tyrosine-protein phosphatase non-receptor type 12 | Ptpn12 | 22.5104351 | 22.25721359 | 22.24907494 | 22.26984978 | 22.66999054 | 22.52867126 |
|  | 1.653024132 | -0.150583903 | P62754 | 40S ribosomal protein S6 | Rps6 | 26.33154297 | 26.44797707 | 26.37949562 | 26.55153847 | 26.56946945 | 26.48975945 |
|  | 1.433543732 | -0.15046374 | D3Z3A0;Q9DCL8 | Protein phosphatase inhibitor 2 | Ppp1r2 | 23.71994019 | 23.6135807 | 23.77916527 | 23.84794998 | 23.8493824 | 23.866745 |
|  | 1.176277117 | -0.149803162 | Q3TWV4;P84091;A0A338P798;A0A338P6T4 | AP-2 complex subunit mu | Ap2m1 | 24.88543892 | 24.83137321 | 24.96349335 | 25.1312809 | 25.02225304 | 24.97618103 |
|  | 0.370156078 | -0.14959844 | Q7TNG5;D6RGM3;A0A0U1RQ42 | Echinoderm microtubule-associated protein-like 2 | Eml2 | 23.16448975 | 22.96837044 | 23.03424263 | 23.01070786 | 23.3998909 | NaN |
|  | 1.679699037 | -0.149368922 | P63005;Q5SW16 | Platelet-activating factor acetylhydrolase IB subunit alpha | Pafah1b1 | 24.92243195 | 25.03267288 | 24.91287231 | 25.10637474 | 25.0832653 | 25.12644386 |
|  | 1.319287024 | -0.149183909 | Q8BQ30;A0A087WRI9 | Phostensin | Ppp1r18 | 23.32416153 | 23.32333565 | 23.37079239 | 23.55039406 | 23.52707291 | 23.38837433 |
|  | 2.005221059 | -0.14867719 | P32020 | Non-specific lipid-transfer protein | Scp2 | 25.09393883 | 25.10569572 | 25.16061783 | 25.280756 | 25.30438232 | 25.22114563 |
|  | 2.768540886 | -0.148663203 | Q9D3L3;O09044;B0R030;B0R029;E9Q8A1;P60879 | Synaptosomal-associated protein;Synaptosomal-associated protein 23 | Snap23 | 24.0539093 | 24.04685211 | 24.02005005 | 24.15499115 | 24.20530891 | 24.20650101 |
|  | 0.754246359 | -0.148438136 | Q3V4D5;B1AUY9;B1AUZ1;B1AUY8;B1AUY7;Q3UX61;Q9QY36 | N-alpha-acetyltransferase 11;N-alpha-acetyltransferase 10 | Naa10;Naa11 | 23.36879539 | 23.420084 | 23.36746407 | 23.61031723 | 23.6344471 | 23.35689354 |
|  | 0.156916616 | -0.148346583 | Q9ERG0 | LIM domain and actin-binding protein 1 | Lima1 | 22.29689789 | NaN | 22.09452057 | 22.86448097 | 22.12512016 | 22.0425663 |
|  | 2.268595027 | -0.147699992 | Q9DCD0 | 6-phosphogluconate dehydrogenase, decarboxylating | Pgd | 27.79525948 | 27.80728722 | 27.84565544 | 27.92461967 | 27.96504593 | 28.00163651 |
|  | 1.026472444 | -0.147081375 | Q61029 | Lamina-associated polypeptide 2, isoforms beta/delta/epsilon/gamma | Tmpo | 23.78815651 | 23.94342232 | 23.73221016 | 23.93634033 | 24.01324844 | 23.95544434 |
|  | 1.62571397 | -0.146588008 | A0A1D5RLW5;A0A1D5RM85;P62717;A0A1D5RM79;A0A1D5RME4;A0A1D5RMC7 | 60S ribosomal protein L18a | Rpl18a | 25.16257858 | 25.084198 | 25.22158813 | 25.28668976 | 25.29824066 | 25.32319832 |
|  | 0.404018344 | -0.145192464 | Q99P65;D6RI95;D3YWS9 | Equilibrative nucleoside transporter 3 | Slc29a3 | 22.61616325 | 22.56832123 | 23.01901436 | 22.80226898 | 22.85661888 | 22.98018837 |
|  | 1.451758527 | -0.144870122 | B7ZCL8;P70290;A2AN84;B7ZCL9;B7ZCM0;B7ZCM1;D6RFD5 | 55 kDa erythrocyte membrane protein | Mpp1 | 25.26828003 | 25.14616013 | 25.21892929 | 25.41386795 | 25.31444359 | 25.33966827 |
|  | 2.608658785 | -0.144769669 | P08752;A0A0A6YWA9;B2RSH2;A0A0A6YXN0;A0A0A6YXC2 | Guanine nucleotide-binding protein G(i) subunit alpha-2 | Gnai2 | 26.62435913 | 26.58677673 | 26.60526657 | 26.75709915 | 26.77806282 | 26.71554947 |
|  | 1.212989172 | -0.14461263 | A0A3B2WCL5;Q9CT10 | Ran-binding protein 3 | Ranbp3 | 22.55285835 | 22.49472618 | 22.63999939 | 22.6571579 | 22.77898407 | 22.68527985 |
|  | 1.043777706 | -0.14413325 | Q7TMM9 | Tubulin beta-2A chain | Tubb2a | 24.359375 | 24.48480606 | 24.4759922 | 24.66448975 | 24.49032593 | 24.59775734 |
|  | 0.24334491 | -0.142244339 | P56812;D3Z7Q5 | Programmed cell death protein 5 | Pdcd5 | 23.21170807 | 23.64383507 | 23.82133484 | 23.8907814 | 23.79223251 | 23.42059708 |
|  | 0.262464471 | -0.141598066 | Q31125 | Zinc transporter SLC39A7 | Slc39a7 | 23.16934586 | NaN | 23.42534447 | 23.15556335 | 23.53682518 | 23.62444115 |
|  | 1.072287737 | -0.141569773 | B7ZNP3;Q3V4B5 | COMM domain-containing protein 6 | Commd6 | 23.59217834 | 23.45236778 | 23.55578613 | 23.63344955 | 23.7664814 | 23.62511063 |
|  | 2.064027055 | -0.14152209 | P08030;A0A1D5RLR6 | Adenine phosphoribosyltransferase | Aprt | 24.3087616 | 24.24103546 | 24.33844566 | 24.42579269 | 24.4461956 | 24.44082069 |
|  | 0.844152136 | -0.141066233 | Q3TCU5;Q9R233;G3UZZ2;A0A3Q4EGL0 | Tapasin | Tapbp | 23.57888222 | 23.58817863 | 23.78596306 | 23.75235939 | 23.86844254 | 23.75542068 |
|  | 0.913263585 | -0.140784582 | O09110;A0A0R4J1Q6;A2AGS2;P70236 | Dual specificity mitogen-activated protein kinase kinase 3 | Map2k3 | 22.80142021 | 22.69466209 | 22.67661285 | 22.74404716 | 22.92156219 | 22.92943954 |
|  | 1.377757227 | -0.140616735 | A0A0R4J0Z1;P08003 | Protein disulfide-isomerase A4 | Pdia4 | 25.23950768 | 25.10888863 | 25.2242775 | 25.36879539 | 25.33864975 | 25.28707886 |
|  | 1.350813399 | -0.139691035 | Q78ZM0;O70492;D3Z789;D3Z6Z0 | Sorting nexin-3 | Snx3 | 26.27682304 | 26.39819527 | 26.42337418 | 26.53382874 | 26.47500229 | 26.50863457 |
|  | 1.292698502 | -0.138734818 | Q8VBT6;A0A0U1RNM0;A0A0U1RNE8;A0A0U1RPR7 | Apolipoprotein B receptor | Apobr | 23.8338356 | 23.89993668 | 23.93786621 | 23.95162582 | 24.08537483 | 24.05084229 |
|  | 0.454962281 | -0.138069789 | G3UZ60;G3UWI9;G3UWX9;Q9Z172;G3UZA7 | Small ubiquitin-related modifier 3;Small ubiquitin-related modifier | Sumo3 | 25.574646 | 25.60430527 | 25.48225403 | 25.82347298 | 25.8111515 | 25.44079018 |
|  | 0.637454849 | -0.137619019 | P68254 | 14-3-3 protein theta | Ywhaq | 25.8771286 | 25.75692177 | 25.55092239 | 25.8289299 | 25.87032509 | 25.89857483 |
|  | 1.609320998 | -0.137524923 | A0A3B2WDD2;A0A3B2WBL1;Q5XJF6;P53026;A0A3B2W824;A0A3B2W820;A0A3B2WCA5;D6RE43 | Ribosomal protein;60S ribosomal protein L10a | Rpl10a | 26.48643303 | 26.42927742 | 26.4942379 | 26.64016914 | 26.64154816 | 26.54080582 |
|  | 0.492969556 | -0.136873881 | Q8R3D1;D6RI34;D6RJI8 | TBC1 domain family member 13 | Tbc1d13 | 22.58419228 | NaN | 22.3999691 | 22.73413467 | 22.49128151 | 22.66144753 |
|  | 0.243701598 | -0.136727333 | Q5JC28;H3BK65;P42567;F6W2Q5;H3BJB8;A0A0R4J0A0;H3BLE4 | Epidermal growth factor receptor substrate 15 | Eps15 | 21.97088242 | 22.25917053 | NaN | 22.10836983 | NaN | 22.39513779 |
|  | 1.763177568 | -0.13661321 | Q8C483;P26638;A2AFS0;A2AFS1 | Serine--tRNA ligase, cytoplasmic | Sars | 24.82779121 | 24.83860397 | 24.74586296 | 24.96181679 | 24.95730591 | 24.90297508 |
|  | 0.620427395 | -0.13639005 | Q91W90;E9PXX7;A0A0R4J1Y7 | Thioredoxin domain-containing protein 5 | Txndc5 | 23.94966888 | 24.00195885 | 23.99524498 | 24.0921669 | 24.29914856 | 23.9647274 |
|  | 0.44967668 | -0.136287689 | Q80Y14;A0A1Y7VN70 | Glutaredoxin-related protein 5, mitochondrial | Glrx5 | 23.21815491 | 23.13832092 | 23.40652466 | 23.29949951 | 23.59696198 | 23.27540207 |
|  | 0.770347963 | -0.136246363 | Q61024 | Asparagine synthetase [glutamine-hydrolyzing] | Asns | 22.38595581 | 22.20401001 | 22.20158958 | 22.29328156 | 22.46620369 | 22.44080925 |
|  | 0.874671561 | -0.136084239 | P62264;D3YVF4;D3Z7I1;A0A494BAS4 | 40S ribosomal protein S14 | Rps14 | 26.5387516 | 26.56321144 | 26.39517403 | 26.61203384 | 26.73091507 | 26.56244087 |
|  | 1.05263088 | -0.136025111 | P14733 | Lamin-B1 | Lmnb1 | 24.1589241 | 24.01239586 | 23.95606613 | 24.17004967 | 24.1890316 | 24.17638016 |
|  | 1.448627513 | -0.135953903 | Q9CWK8 | Sorting nexin-2 | Snx2 | 25.5693531 | 25.47691917 | 25.59043694 | 25.70340157 | 25.71179962 | 25.62936974 |
|  | 1.222031333 | -0.135413488 | P51660 | Peroxisomal multifunctional enzyme type 2;(3R)-hydroxyacyl-CoA dehydrogenase;Enoyl-CoA hydratase 2 | Hsd17b4 | 25.14949799 | 25.17039299 | 25.27447891 | 25.32436752 | 25.39800072 | 25.27824211 |
|  | 0.995704447 | -0.134633382 | S4R1B8;S4R270;A0A5F8MQ05;D3Z6Q9;S4R2J8;S4R171;S4R1Q1 | Bridging integrator 2 | Bin2 | 23.19389725 | 23.19239235 | 23.04706764 | 23.2041893 | 23.28926086 | 23.34380722 |
|  | 1.013125196 | -0.134508769 | Q61510;Q5SU71;Q5SU72 | E3 ubiquitin/ISG15 ligase TRIM25 | Trim25 | 22.82880974 | 22.69548988 | 22.890522 | 22.91333008 | 22.98712921 | 22.91788864 |
|  | 0.372801868 | -0.134488424 | P61965;F6Q3W0 | WD repeat-containing protein 5 | Wdr5 | 22.6711998 | 22.50741386 | 22.75325775 | 22.52626038 | 22.97640038 | 22.83267593 |
|  | 1.591145764 | -0.133710861 | Q8C845;Q9D8Y0;Q9D4J1 | EF-hand domain-containing protein D2 | Efhd2 | 26.67006683 | 26.69005775 | 26.59903526 | 26.83701515 | 26.77831268 | 26.7449646 |
|  | 0.487188005 | -0.133674622 | F7CDT0;F6WMC0;P61082;G5E919 | NEDD8-conjugating enzyme Ubc12 | Ube2m | 24.00401688 | 24.26849365 | 23.89087486 | 24.10461617 | 24.23118019 | 24.2286129 |
|  | 0.712156996 | -0.133521398 | P41105 | 60S ribosomal protein L28 | Rpl28 | 26.28730774 | 26.30413818 | 26.33104706 | 26.41369057 | 26.30990601 | 26.5994606 |
|  | 1.783953874 | -0.132950465 | G5E829;A0A1W2P7C7;F7AAP4;E9Q828;Q3UHH0;Q6Q477;F8WHB1;Q9R0K7;A0A1W2P867;A0A1W2P7R3 | Plasma membrane calcium-transporting ATPase 1 | Atp2b1 | 23.5525074 | 23.6105423 | 23.63223076 | 23.71952248 | 23.7766571 | 23.69795227 |
|  | 0.242304595 | -0.132548014 | G3UZ30;A5A4Y9;Q8K1L5 | Protein phosphatase 1 regulatory subunit 11 | Ppp1r11 | 21.9932766 | 22.15279579 | 21.69655228 | 22.2401619 | 21.92001724 | NaN |
|  | 0.830528824 | -0.132431666 | A2A9X5;Q9JM14 | 5(3)-deoxyribonucleotidase, cytosolic type | Nt5c | 23.89827538 | 23.91250801 | 24.10844994 | 24.11902046 | 24.04909706 | 24.1484108 |
|  | 0.587163717 | -0.132394155 | P28063;G3UZW8;A0A494BAB6 | Proteasome subunit beta type-8;Proteasome subunit beta type | Psmb8 | 24.31451225 | 24.34102821 | 24.29593086 | 24.44044113 | 24.28142929 | 24.62678337 |
|  | 1.447455899 | -0.13222758 | P62874;H3BKR2;H3BLF7;Q61011 | Guanine nucleotide-binding protein G(I)/G(S)/G(T) subunit beta-1 | Gnb1 | 25.48545074 | 25.48741531 | 25.59186363 | 25.62862015 | 25.70136642 | 25.63142586 |
|  | 1.144455116 | -0.131800334 | Q8VE47;F6VRI6;D6RH76;A0A1L1SSH6 | Ubiquitin-like modifier-activating enzyme 5 | Uba5 | 23.05890465 | 23.12751389 | 23.1832428 | 23.33565331 | 23.22138786 | 23.20802116 |
|  | 0.227801518 | -0.130185445 | P05213;A0A2R8VHF3 | Tubulin alpha-1B chain | Tuba1b | 26.5325222 | 26.0150795 | 26.74406624 | 26.67020226 | 26.4836235 | 26.52839851 |
|  | 3.18070802 | -0.129896164 | P27773;F6Q404 | Protein disulfide-isomerase A3 | Pdia3 | 27.14724731 | 27.13349724 | 27.16681671 | 27.2644558 | 27.29684067 | 27.27595329 |
|  | 2.622753511 | -0.129751841 | Q61768;E9QAK5;P28738;P33175;A2ARD4 | Kinesin-1 heavy chain;Kinesin-like protein | Kif5b | 24.1165638 | 24.12462997 | 24.15583992 | 24.28996468 | 24.24008942 | 24.25623512 |
|  | 0.426693947 | -0.129407247 | A0A0E2WA25 | Chaperone protein HtpG | htpG | 27.94620323 | 27.71279716 | 28.05268669 | 27.89110756 | 28.17483711 | 28.03396416 |
|  | 0.352555125 | -0.129197439 | Q8BFY6 | Peflin | Pef1 | 22.00422287 | 22.2196312 | 22.44265747 | 22.41562843 | 22.45500565 | 22.18346977 |
|  | 0.909701162 | -0.129170736 | Q99KJ8 | Dynactin subunit 2 | Dctn2 | 24.87192345 | 24.73329544 | 24.86031723 | 24.98180962 | 25.01656914 | 24.85466957 |
|  | 0.282020709 | -0.128473918 | Q6XLQ8;O35887;G3V004;G3UXA8;G3UWV3;G3UWR0;G3UXA3;G3UY49 | Calumenin | Calu | 23.35259056 | 23.0731163 | 23.11734009 | 23.37079239 | 23.55379677 | 23.00387955 |
|  | 0.446030032 | -0.127674103 | Q921J2 | GTP-binding protein Rheb | Rheb | 23.55929184 | 23.30270958 | 23.39178658 | 23.73954201 | 23.43300819 | 23.4642601 |
|  | 0.346693225 | -0.127118429 | A0A171KXD3;Q9JIF0;A0A140LJF4;A0A140LHF7;Q6PAK3 | Protein arginine N-methyltransferase 1 | Prmt1 | 23.16012573 | 23.40301704 | 23.48086739 | 23.69795227 | 23.29963875 | 23.42777443 |
|  | 0.868541687 | -0.12646548 | A0A0R4J0P5;P97814 | Proline-serine-threonine phosphatase-interacting protein 1 | Pstpip1 | 22.89500618 | 22.79282951 | 22.78964996 | 22.89731598 | 22.89083862 | 23.06872749 |
|  | 0.549636193 | -0.125686646 | Q9D1J1;F6RCM5 | Adaptin ear-binding coat-associated protein 2 | Necap2 | 23.74827003 | 23.9057312 | 23.96648788 | 23.93894386 | 23.90554619 | 24.15305901 |
|  | 1.302181016 | -0.125676473 | B1ARU4;Q9QXZ0;A0A571BGC6;A0A571BF93;A0A0A0MQA6;F7ACR9;F6Q750;F6SHS0;F6YKN8;A0A5F8MP99;F6RCJ3;F6RL59;A0A0A0MQH5;S4R2C6;S4R2A8;S4R1Y6 | Microtubule-actin cross-linking factor 1 | Macf1 | 23.4432888 | 23.45136261 | 23.38099861 | 23.60206985 | 23.47345543 | 23.57715416 |
|  | 1.522782676 | -0.125514984 | Q9CZD3 | Glycine--tRNA ligase | Gars | 23.76141739 | 23.77906418 | 23.82425117 | 23.95907593 | 23.84919167 | 23.9330101 |
|  | 0.501682628 | -0.124804815 | O35685 | Nuclear migration protein nudC | Nudc | 23.59809875 | 23.4873848 | 23.68228149 | 23.84105301 | 23.76809883 | 23.53302765 |
|  | 1.137589704 | -0.123330434 | O08807;B1AZS9 | Peroxiredoxin-4 | Prdx4 | 31.57086372 | 31.52046967 | 31.63676834 | 31.64710236 | 31.67703247 | 31.77395821 |
|  | 0.813224334 | -0.123095194 | P46664 | Adenylosuccinate synthetase isozyme 2 | Adss | 24.27106285 | 24.42828751 | 24.22096062 | 24.48689461 | 24.37722969 | 24.42547226 |
|  | 0.310882743 | -0.12297376 | Q3TM89;Q6P8I4 | PEST proteolytic signal-containing nuclear protein | Pcnp | 22.83062744 | 22.69786644 | 22.95445061 | 23.03131866 | 23.14878464 | 22.67176247 |
|  | 2.025773669 | -0.122845332 | P84089;A0A1W2P7H9;A0A1W2P7T3;G3UW85 | Enhancer of rudimentary homolog | Erh | 25.20333099 | 25.11457825 | 25.16407585 | 25.27629089 | 25.28026199 | 25.2939682 |
|  | 2.911455592 | -0.121589661 | O08553;Q3TT92;E9PWE8;Q62188;Q6P1J1;P97427;Q71H75 | Dihydropyrimidinase-related protein 2 | Dpysl2 | 26.20784187 | 26.17529488 | 26.15823174 | 26.3020649 | 26.29652596 | 26.30754662 |
|  | 1.235848322 | -0.121526718 | Q3TML0;F7DBQ0;Q922R8 | Protein disulfide-isomerase A6 | Pdia6 | 25.84006882 | 25.75547028 | 25.8401165 | 25.86053085 | 25.96459579 | 25.9751091 |
|  | 0.689356757 | -0.12147967 | Q9ERN0;A0A1L1SUU4 | Secretory carrier-associated membrane protein 2 | Scamp2 | 24.17949677 | 24.1882782 | 24.29417801 | 24.47784615 | 24.31188202 | 24.23666382 |
|  | 0.55553239 | -0.121312459 | Q3UGC7;Q66JS6 | Eukaryotic translation initiation factor 3 subunit J-A;Eukaryotic translation initiation factor 3 subunit J-B | Eif3j1;Eif3j2 | 23.42547226 | 23.22510147 | 23.21267319 | 23.50639725 | 23.44240379 | 23.27838326 |
|  | 2.028433037 | -0.121102651 | P62880;E9QKR0;D3YZX3;D3Z1M1;D3Z1T4;E9PWM7;A0A0A6YVN9;V9GWY1 | Guanine nucleotide-binding protein G(I)/G(S)/G(T) subunit beta-2 | Gnb2 | 27.16009903 | 27.07255173 | 27.12317085 | 27.24209023 | 27.23059273 | 27.24644661 |
|  | 1.934948718 | -0.12091891 | Q9CW03 | Structural maintenance of chromosomes protein 3 | Smc3 | 28.61389542 | 28.61642265 | 28.69557762 | 28.75232124 | 28.77218819 | 28.76414299 |
|  | 1.357909623 | -0.120409012 | A2AMH3;A2AMH5;A2AMH4;Q6X893 | Choline transporter-like protein 1 | Slc44a1 | 22.64213753 | 22.70672417 | 22.6404171 | 22.85136604 | 22.76595688 | 22.73318291 |
|  | 0.230732373 | -0.11950175 | P48771 | Cytochrome c oxidase subunit 7A2, mitochondrial | Cox7a2 | 25.88443756 | 25.94480705 | 25.73943901 | 26.15185928 | 26.18652153 | 25.58880806 |
|  | 1.740971216 | -0.119492849 | P61028;REV__G3UYX5 | Ras-related protein Rab-8B | Rab8b | 25.06439209 | 25.13715744 | 25.07528877 | 25.22989655 | 25.23571396 | 25.16970634 |
|  | 0.721465294 | -0.119318008 | D3Z656;Q8CHC4;E9Q7S0;F7BQW7;D3Z1M7;F6VSS8;A0A338P6C7;A0A338P6V7;F7CD11 | Synaptojanin-1 | Synj1 | 22.68388939 | 22.61762047 | 22.7144165 | 22.92728806 | 22.75248337 | 22.69410896 |
|  | 2.686417716 | -0.11874644 | Q6IRU2;A0A571BEU1 | Tropomyosin alpha-4 chain | Tpm4 | 25.65572739 | 25.65439034 | 25.6069603 | 25.76329422 | 25.7613678 | 25.74865532 |
|  | 1.247368834 | -0.118478139 | P00493 | Hypoxanthine-guanine phosphoribosyltransferase | Hprt1 | 25.40833855 | 25.40207291 | 25.40846825 | 25.60294724 | 25.52268028 | 25.4486866 |
|  | 3.08972251 | -0.117682775 | P26041 | Moesin | Msn | 28.39239311 | 28.40649033 | 28.40576172 | 28.542593 | 28.5131073 | 28.50199318 |
|  | 0.658721293 | -0.117260615 | Q60972 | Histone-binding protein RBBP4 | Rbbp4 | 23.67281914 | 23.87502098 | 23.87820625 | 23.97149658 | 23.83922768 | 23.96710396 |
|  | 0.74930443 | -0.116973241 | O54833;A0A1D5RM74;A0A1D5RLE4;A0A1D5RM55 | Casein kinase II subunit alpha | Csnk2a2 | 22.44458008 | 22.39210129 | 22.54498291 | 22.61191559 | 22.46814346 | 22.65252495 |
|  | 1.064423357 | -0.116334915 | Q9ET22 | Dipeptidyl peptidase 2 | Dpp7 | 24.52145195 | 24.44606972 | 24.56307983 | 24.67707443 | 24.65108109 | 24.55145073 |
|  | 1.080584228 | -0.115825653 | Q9ES97 | Reticulon-3 | Rtn3 | 25.59960175 | 25.66223526 | 25.55104065 | 25.65127182 | 25.72364235 | 25.78544044 |
|  | 3.166449757 | -0.114829381 | P62918 | 60S ribosomal protein L8 | Rpl8 | 26.58720589 | 26.55441093 | 26.5519352 | 26.67168617 | 26.68552017 | 26.68083382 |
|  | 0.607525348 | -0.114778519 | Q11011;F6QYF8;E9Q039;E9Q6F4;F6V7K3;F2Z3V5;F7ANF4 | Puromycin-sensitive aminopeptidase | Npepps | 23.89753723 | 23.77283669 | 23.69826889 | 23.94761848 | 23.98259163 | 23.78276825 |
|  | 2.704582073 | -0.11430041 | P08113;F7C312 | Endoplasmin | Hsp90b1 | 26.71358299 | 26.74059677 | 26.7551899 | 26.8707943 | 26.84385872 | 26.83761787 |
|  | 0.282554043 | -0.114215215 | Q7TNV0;E9Q8Y1;D3YVJ6 | Protein DEK | Dek | 23.08290863 | 22.84758568 | 22.68069458 | NaN | 22.92108917 | 23.04813385 |
|  | 1.533588295 | -0.114103317 | Q9CY58;Q3UMP4;A0A0N4SV32;A0A0N4SUQ1;A0A0N4SUN8;A0A0N4SWH2;A0A0N4SV40;A0A0N4SVK5 | Plasminogen activator inhibitor 1 RNA-binding protein | Serbp1 | 26.04333115 | 26.0309906 | 26.0490551 | 26.09934807 | 26.21645355 | 26.14988518 |
|  | 2.097974132 | -0.114044825 | Q6ZWV3;I7HLV2;P86048;A0A1B0GXC3 | 60S ribosomal protein L10;60S ribosomal protein L10-like | Rpl10;Rpl10l | 26.38121223 | 26.35440636 | 26.39839172 | 26.48657227 | 26.52801132 | 26.4615612 |
|  | 0.825691443 | -0.113858541 | A2AL85;Q8BSY0;Q8CBM2;A2AL83;A2AL79;A2AL78;Q3TU40;A2AL81;A2AL71;A2AL77;Q9CR06;A2AL74;A2AL76;A2AL75 | Aspartyl/asparaginyl beta-hydroxylase | Asph | 23.45575714 | 23.48591232 | 23.34840775 | 23.57369232 | 23.44922256 | 23.60873795 |
|  | 0.174946415 | -0.113654137 | Q80VP1;D3Z4V3;D3Z550;Q91W69 | Epsin-1 | Epn1 | 22.03503227 | NaN | 22.14718437 | 21.98307991 | 22.42644501 | NaN |
|  | 2.156932362 | -0.113615672 | P70195 | Proteasome subunit beta type-7 | Psmb7 | 25.10225487 | 25.03707886 | 25.10241508 | 25.18854141 | 25.19042587 | 25.20362854 |
|  | 1.317917333 | -0.113590876 | A2A6U3;Q80UG5;A2A6U5;A8Y5D3 | Septin-9 | Septin9 | 24.48209953 | 24.44613266 | 24.57554054 | 24.60806084 | 24.59849548 | 24.63798904 |
|  | 0.47828054 | -0.113440196 | Q8BFY9;Q3TKD0;J3QMX2 | Transportin-1 | Tnpo1 | 23.15736771 | 22.98421097 | 23.07336044 | 23.11618233 | 23.07548523 | 23.36359215 |
|  | 0.644907642 | -0.113273621 | Z4YKB8;Q3TEA8;Z4YKA3;A2AM70;A2AM65;A2AM62;A2AM63;A2AM69 | Heterochromatin protein 1-binding protein 3 | Hp1bp3 | 23.72764778 | 23.79372215 | 23.85737801 | 24.01350403 | 23.77484894 | 23.93021584 |
|  | 0.661407713 | -0.113126119 | O08804;A0A1Y7VLD5;F7B9A0;I7HJI3 | Serine (or cysteine) peptidase inhibitor, clade B, member 6b | Serpinb6b | 23.37610245 | 23.27398109 | 23.53812981 | 23.51641083 | 23.490448 | 23.52073288 |
|  | 2.643421179 | -0.112997055 | Q8VDD5;A0A2R8VKI5;Q5SV64;Q3UH59;Q61879;F2Z494;A0A2R8W6V7;Q8BXF2 | Myosin-9 | Myh9 | 26.93764305 | 26.92388344 | 26.89469528 | 27.0506134 | 27.02928543 | 27.0153141 |
|  | 0 | -0.112718582 | Q9D1K7;F8WIU1;H7BXB9 | UPF0687 protein C20orf27 homolog | 1700037H04Rik | 22.12082291 | NaN | 21.96063232 | NaN | 22.1534462 | NaN |
|  | 0.821580951 | -0.112058004 | Q8BYC6;Q3V3K3;A0A0R4J1T3;F6RXB5;Q6ZQ29 | Serine/threonine-protein kinase TAO3 | Taok3 | 23.47047997 | 23.53492737 | 23.51665115 | 23.58451462 | 23.5370636 | 23.73665428 |
|  | 1.137543628 | -0.110190709 | Q8K1I7;F6QWW7;F6RQI2 | WAS/WASL-interacting protein family member 1 | Wipf1 | 24.54403877 | 24.64383507 | 24.58629036 | 24.63411522 | 24.75271797 | 24.71790314 |
|  | 0.333093435 | -0.110017141 | Q9ET01;Q3UEJ6 | Glycogen phosphorylase, liver form;Alpha-1,4 glucan phosphorylase | Pygl | 23.77976608 | 23.69657326 | 23.7424221 | 24.10077286 | 23.64295387 | 23.80508614 |
|  | 0.714017835 | -0.109898885 | P63325;A0A338P731;Q3UW83;A0A338P7K4;A0A3B2W864 | 40S ribosomal protein S10 | Rps10 | 25.97294235 | 25.9691925 | 26.14557648 | 26.16779327 | 26.06101608 | 26.18859863 |
|  | 0.677286204 | -0.109130224 | D3YUT3;D3Z5R8;D3Z722;Q9CZX8;D3YUG3;S4R223 | 40S ribosomal protein S19 | Rps19 | 26.08310318 | 26.3227005 | 26.21450806 | 26.26906586 | 26.3448925 | 26.33374405 |
|  | 0.914741268 | -0.109032949 | Q7TQE2;Q62523;A0A0N4SVD2;A0A0N4SUX7;A0A0N4SVP4 | Zyxin | Zyx | 23.3709259 | 23.31174469 | 23.31962013 | 23.33960152 | 23.48000526 | 23.50978279 |
|  | 0.760982297 | -0.108620962 | P35279;D3YV69;P61294;A0A1L1SRS6;Q8BHD0 | Ras-related protein Rab-6A;Ras-related protein Rab-6B | Rab6a;Rab6b | 27.88151169 | 27.99042702 | 27.99393845 | 28.17069054 | 28.02770424 | 27.99334526 |
|  | 0.345473734 | -0.108574867 | O54950;A0A2R8VHU2;D3YUS1;Q8BIQ9;Q91WG5 | 5-AMP-activated protein kinase subunit gamma-1 | Prkag1 | NaN | 23.36279106 | 23.24540901 | 23.31146622 | 23.51388359 | NaN |
|  | 0.37521967 | -0.108470917 | Q9WVQ5 | Methylthioribulose-1-phosphate dehydratase | Apip | 22.87434578 | 22.87805557 | 22.60584831 | 22.90800476 | 22.88110352 | NaN |
|  | 1.116030937 | -0.108179728 | E9PV41;F6XC54;D3Z074;E9PXV7;O08808 | Protein diaphanous homolog 1 | Diap1;Diaph1 | 24.38837433 | 24.37755966 | 24.26277161 | 24.42624092 | 24.49393272 | 24.43307114 |
|  | 0.550200073 | -0.107991536 | Q6P5E6;A2A9W5 | ADP-ribosylation factor-binding protein GGA2 | Gga2 | 22.23420906 | 22.46475792 | 22.46694946 | 22.5311718 | 22.41725349 | 22.54146576 |
|  | 1.106554465 | -0.107538223 | A2AL50;Q8C0I1;H3BKN2;A2AL49;H3BIY5 | Alkyldihydroxyacetonephosphate synthase, peroxisomal | Agps | 22.78761864 | 22.840765 | 22.82962227 | 22.98979759 | 22.84531021 | 22.94551277 |
|  | 2.62553995 | -0.107208252 | P60710;E9Q1F2;E9Q5F4;G3UZ07;F8WGM8;E9Q2D1;F6WX90;A0A0U1RQ96;E9Q3M9 | Actin, cytoplasmic 1;Actin, cytoplasmic 1, N-terminally processed | Actb | 33.03157425 | 33.04757309 | 33.00209045 | 33.15010071 | 33.12270355 | 33.13005829 |
|  | 1.926624566 | -0.106685638 | Q3TXS7;J3QN38;D6RGR5 | 26S proteasome non-ATPase regulatory subunit 1 | Psmd1 | 24.54963112 | 24.48609543 | 24.50476074 | 24.64987564 | 24.61234283 | 24.59832573 |
|  | 0.929402323 | -0.10659345 | Q91VW3;I7HPY0 | SH3 domain-binding glutamic acid-rich-like protein 3 | Sh3bgrl3 | 28.46905899 | 28.37600327 | 28.32890129 | 28.54465866 | 28.43101501 | 28.51807022 |
|  | 0.294768032 | -0.106400172 | Q8BZN6;A0A0R4J2B7;A0A087WRP5;A0A087WS26;Q8BLX9;A0A087WQA1 | Dedicator of cytokinesis protein 10 | Dock10 | 22.45956421 | 22.51376343 | 22.80108452 | 22.88341713 | 22.67348862 | 22.53670692 |
|  | 1.031040991 | -0.106379827 | O35704 | Serine palmitoyltransferase 1 | Sptlc1 | 22.96116257 | 22.96652412 | 23.05949974 | 23.06725121 | 23.17497826 | 23.06409645 |
|  | 1.077796236 | -0.105962118 | O70503 | Very-long-chain 3-oxoacyl-CoA reductase | Hsd17b12 | 25.33769608 | 25.42831802 | 25.4379673 | 25.56147957 | 25.44635391 | 25.51403427 |
|  | 0.634294203 | -0.105419159 | P97797;A2ANC1;A0A0A6YWR3;Q6F5F2;A0A0A6YYP6 | Tyrosine-protein phosphatase non-receptor type substrate 1 | Sirpa | 24.99407959 | 25.08029938 | 25.14685822 | 25.09188461 | 25.29529953 | 25.15031052 |
|  | 1.329449728 | -0.105067571 | B1AWE0;Q6PFA2;B1AWE1;B1AWD8;B1AWD9;O08585 | Clathrin light chain A | Clta | 25.57600212 | 25.50781822 | 25.61133003 | 25.70777702 | 25.66776848 | 25.63480759 |
|  | 0.554181967 | -0.104911804 | Q8BTU6;P10630;A0A338P6X5;E9Q561;D6RJ60 | Eukaryotic initiation factor 4A-II;Eukaryotic initiation factor 4A-II, N-terminally processed | Eif4a2 | 23.37703133 | 23.3521862 | 23.18156052 | NaN | 23.41763878 | 23.39937019 |
|  | 0.508559021 | -0.10476621 | Q8BU30;E9Q866;F6Q6R1 | Isoleucine--tRNA ligase, cytoplasmic | Iars | 23.57819176 | 23.47555923 | 23.30702591 | 23.51340103 | 23.51641083 | 23.64526367 |
|  | 0.821144497 | -0.103837967 | Q920E5;A0A0G2JEA5;A0A0G2JDJ5;A0A0G2JEB3 | Farnesyl pyrophosphate synthase | Fdps | 24.14957428 | 23.97009277 | 24.04685211 | 24.2053833 | 24.11195564 | 24.16069412 |
|  | 0.616100144 | -0.103683472 | Q9Z1Q9;G3UY93;G3UZ22;G3UYW2;G3UZX1 | Valine--tRNA ligase | Vars | 23.9452095 | 24.07977104 | 23.82065201 | 24.07365608 | 24.04226494 | 24.04076195 |
|  | 0.236286439 | -0.103535016 | D3YTP8;Q9CY46;Q9QXA5;A0A1B0GRR9;A0A1B0GQZ9 | U6 snRNA-associated Sm-like protein LSm4 | Lsm4 | 22.60711288 | 22.93012428 | 22.69272614 | 22.69674301 | 22.99696922 | NaN |
|  | 0.479386555 | -0.103522619 | Q922F4 | Tubulin beta-6 chain | Tubb6 | 24.06997299 | 24.07691956 | 24.11004448 | 24.3748436 | 24.10477638 | 24.0878849 |
|  | 0.4090929 | -0.10347875 | D3YZ98;E9QLS6;Q6PF93;E9Q824 | Phosphatidylinositol 3-kinase;Phosphatidylinositol 3-kinase catalytic subunit type 3 | Pik3c3 | 24.24096298 | 24.07740974 | 24.05482101 | 24.33817291 | 24.29586029 | 24.04959679 |
|  | 0.42668146 | -0.103409449 | E9QAI5;B2RQC6;G3UWN2;E9QAT6 | CAD protein;Glutamine-dependent carbamoyl-phosphate synthase;Aspartate carbamoyltransferase;Dihydroorotase | Cad | 21.99337959 | 22.22667503 | NaN | 22.28689575 | 22.18706894 | 22.1663456 |
|  | 0.164586991 | -0.103400548 | A0A0G2JGI9;F8VPN4;F6XXE6;F7CSZ6;E0CYU6;E0CX86 |  | Agl | 22.90904999 | 22.60862541 | 22.91889 | 22.91199684 | 22.54762077 | 23.28714943 |
|  | 0.129088905 | -0.103259087 | Q3UPF5;D3Z5I1;G3X9X5 | Zinc finger CCCH-type antiviral protein 1 | Zc3hav1 | 23.03118324 | NaN | 22.76941109 | 22.76237106 | NaN | 23.24474144 |
|  | 1.08361466 | -0.103167216 | O35601;A0A2I3BRH1;A0A2I3BPY6 | FYN-binding protein | Fyb | 23.80754471 | 23.78426743 | 23.77233315 | 23.85090828 | 23.97823334 | 23.84450531 |
|  | 1.727397706 | -0.103022893 | Q8CG29;P70248;G3V011;G3UZR3 | Unconventional myosin-If | Myo1f | 25.13473129 | 25.20366669 | 25.21144867 | 25.27582932 | 25.30924797 | 25.27383804 |
|  | 0.618641974 | -0.102697372 | Q8BYI6 | Lysophosphatidylcholine acyltransferase 2 | Lpcat2 | 23.67292786 | 23.62243271 | 23.66524887 | 23.88706779 | 23.74663162 | 23.63500214 |
|  | 0.433103375 | -0.102414131 | F6VRP8 | Lectin, galactoside-binding, soluble, 3 binding protein | Lgals3bp | 24.19753075 | 24.18025589 | NaN | 24.14662552 | 24.39813042 | 24.32916641 |
|  | 3.160035566 | -0.102177938 | P48678;D3YUF7 | Prelamin-A/C;Lamin-A/C | Lmna | 26.24894524 | 26.25403786 | 26.26099777 | 26.3669796 | 26.33643723 | 26.36709785 |
|  | 1.336870922 | -0.101899465 | Q9WVJ2;E9Q5I9;F6ZQQ3;E9Q0U1;F6PXS6 | 26S proteasome non-ATPase regulatory subunit 13 | Psmd13 | 24.30277824 | 24.29621124 | 24.33019257 | 24.3614521 | 24.39669418 | 24.47673416 |
|  | 0.453945499 | -0.101720174 | E9QJT5;P56376;Q8BMV3 | Acylphosphatase;Acylphosphatase-1 | Acyp1 | 22.37276077 | NaN | 22.39186478 | 22.59108162 | 22.51236534 | 22.34865189 |
|  | 0.396983995 | -0.101693471 | B2RXS4;Q3UH93 | Plexin-B2 | Plxnb2 | 22.99000549 | 23.33497238 | 23.23286438 | 23.30201149 | 23.34068871 | 23.22022247 |
|  | 0.8295218 | -0.101678848 | A0A0G2JG00;Q3TUE1;A0A0G2JGW9;A0A0G2JFY5;Q3UUU2;Q91WJ8;A0A0G2JFK2;A0A0G2JGV9 | Far upstream element-binding protein 1 | Fubp1 | 22.7727375 | 22.92059898 | 22.80968666 | 23.00624466 | 22.90912247 | 22.89269257 |
|  | 1.128676187 | -0.101583163 | Q4PJX1;A0A0A0MQB0 | Protein odr-4 homolog | Odr4;BC003331 | 22.73522949 | 22.64295387 | 22.7039814 | NaN | 22.8146801 | 22.77659607 |
|  | 0.40178577 | -0.10142835 | Q9CZW5 | Mitochondrial import receptor subunit TOM70 | Tomm70a | 22.36032677 | 22.17279816 | 22.37286568 | 22.29642105 | 22.57156563 | 22.34228897 |
|  | 1.188515213 | -0.100215912 | Q9EPL9;Q3TAW3;E9Q296;Q8C178;D3Z2N6;A0A0J9YVH1 | Peroxisomal acyl-coenzyme A oxidase 3;Acyl-coenzyme A oxidase | Acox3 | 23.69466209 | 23.58508682 | 23.68442345 | 23.77625465 | 23.77092361 | 23.71764183 |
|  | 0.238781154 | -0.100185394 | Q9CS42 | Ribose-phosphate pyrophosphokinase 2 | Prps2 | 23.9196167 | 23.85652351 | 23.81284142 | 24.27526093 | 23.72972298 | 23.88455391 |
|  | 0.875531609 | -0.100039164 | Q6ZWN5;F7CJS8;D3YWH9;Q9CXW7;D3Z673;D3YUV6 | 40S ribosomal protein S9 | Rps9 | 26.15821266 | 26.104496 | 26.09906769 | 26.28948975 | 26.2483654 | 26.1240387 |
|  | 0.510121263 | -0.099972407 | Q6P3A9 | ADP-ribosylation factor-like protein 11 | Arl11 | 23.27767372 | 23.2137413 | 22.99862289 | 23.23629951 | 23.26298714 | 23.29066849 |
|  | 1.067190805 | -0.099955877 | Q8VEM8;G5E902 | Phosphate carrier protein, mitochondrial | Slc25a3 | 27.58727837 | 27.52529526 | 27.47413635 | 27.61673737 | 27.6848526 | 27.58498764 |
|  | 0.747147052 | -0.099803289 | Q9D1Q6 | Endoplasmic reticulum resident protein 44 | Erp44 | 24.02013397 | 23.98789215 | 24.17157745 | 24.18805122 | 24.1780529 | 24.11290932 |
|  | 1.441663117 | -0.09960556 | Q921H8;H3BKL5;H3BJZ9;Q8VCH0;H3BKA1;H3BJC1;H3BJG8 | 3-ketoacyl-CoA thiolase A, peroxisomal;3-ketoacyl-CoA thiolase B, peroxisomal | Acaa1a;Acaa1b | 25.02276039 | 24.94207954 | 24.95633125 | 25.08208847 | 25.10333633 | 25.03456306 |
|  | 0.50593151 | -0.09883213 | A0A0R4J1G9;E9QN92;Q8CI59;D3YTP0 | Metalloreductase STEAP3 | Steap3 | NaN | 22.78052711 | 22.78060722 | NaN | 22.80569649 | 22.95310211 |
|  | 0.98867448 | -0.098674774 | P62751 | 60S ribosomal protein L23a | Rpl23a | 27.22296906 | 27.15325165 | 27.09768105 | 27.25610924 | 27.30817223 | 27.20564461 |
|  | 0.463314912 | -0.09828186 | Q62261;A0A0A0MQG2;E9Q397;Q3UGX2;P15508 | Spectrin beta chain, non-erythrocytic 1 | Sptbn1 | 23.08909798 | 23.00694656 | 22.79288864 | 23.01183319 | 23.0862484 | 23.08569717 |
|  | 1.322816732 | -0.097967148 | P14211 | Calreticulin | Calr | 27.67962646 | 27.67229462 | 27.58441353 | 27.76211548 | 27.71089363 | 27.75722694 |
|  | 0.274100141 | -0.097017924 | Q3UW53;E9PYV4;D3Z233;D3YYZ9 | Protein Niban | Fam129a | 25.0287571 | 25.32580757 | 25.47382736 | 25.29670143 | 25.48019028 | 25.34255409 |
|  | 0.773489096 | -0.096906026 | P63163;P27048;A0A0G2JGN4 | Small nuclear ribonucleoprotein-associated protein N;Small nuclear ribonucleoprotein-associated protein B | Snrpn;Snrpb | 24.35984421 | 24.31174469 | 24.22088623 | 24.38146019 | 24.47078896 | 24.33094406 |
|  | 0.331104842 | -0.096789042 | E9QP49;A0A494BBL8;A0A494BA91;Q99MS7;A0A494B9G7;G5E8Y6 | EH domain-binding protein 1-like protein 1 | Ehbp1l1 | 22.26307297 | 22.06608582 | 22.40421295 | 22.20415878 | 22.43341446 | 22.38616562 |
|  | 0.637458737 | -0.095897675 | P29452;A0A571BDI1;A0A571BF22 | Caspase-1;Caspase-1 subunit p20;Caspase-1 subunit p10 | Casp1 | 23.58279228 | 23.54933739 | 23.39519119 | 23.65883636 | 23.61840439 | 23.53777313 |
|  | 0.63726333 | -0.095698039 | P56135;F8WHP8 | ATP synthase subunit f, mitochondrial | Atp5j2 | 25.88199043 | 25.68688393 | 25.71816444 | 25.91806984 | 25.81919098 | 25.8368721 |
|  | 0.689851411 | -0.095314662 | P18181;F8WHM0 | CD48 antigen | Cd48 | 23.5892086 | 23.59957504 | 23.67130852 | 23.82434845 | 23.69178963 | 23.62989807 |
|  | 3.462728779 | -0.095239639 | Q9QXS1;E9Q3W4;A0A3B2W7J8;A0A0R4J218;A0A0R4J223;E9Q9J6;A0A0R4J221;E9PW24 | Plectin | Plec | 25.6738987 | 25.68618965 | 25.66503143 | 25.78161812 | 25.76311684 | 25.76610374 |
|  | 0.416443334 | -0.09519132 | P60122;D3YW60 | RuvB-like 1 | Ruvbl1 | 23.03326988 | 22.81659698 | 22.98438454 | 22.98702431 | 23.09285927 | NaN |
|  | 0.441371425 | -0.094859441 | Q9DBZ5;Q3TY56;A0A140LJ59 | Eukaryotic translation initiation factor 3 subunit K | Eif3k | 23.28362083 | 23.3925724 | 23.27198792 | 23.56767082 | 23.28079224 | 23.38429642 |
|  | 0.32549371 | -0.094647725 | O88545;D3Z0F5;F6QK86 | COP9 signalosome complex subunit 6 | Cops6 | 22.81647873 | 22.85149956 | 22.94343948 | NaN | 23.10554314 | 22.82469749 |
|  | 0.619616153 | -0.094148636 | P97494 | Glutamate--cysteine ligase catalytic subunit | Gclc | 22.65130043 | 22.79457474 | 22.59224701 | 22.83745766 | 22.73152542 | 22.75158501 |
|  | 0.528444694 | -0.094127019 | Q3U1J4;A0A494B987;A0A494BAF6;A0A494BBJ6 | DNA damage-binding protein 1 | Ddb1 | 23.65075111 | 23.40509605 | 23.45035553 | 23.59821129 | 23.63189697 | 23.55847549 |
|  | 1.851756936 | -0.093904495 | P26039;A2AIM2;E9PUM4;A0A1L1SQ51;Q71LX4;Q8CDM9;A0A1L1SRI1;A0A1L1SQP9;F6S1V7;F6SX70 | Talin-1 | Tln1 | 27.02770424 | 27.06732941 | 27.04280853 | 27.17588425 | 27.1340847 | 27.10958672 |
|  | 1.353556497 | -0.093679428 | Q00612;A3KG36;G3UWD6;P97324;REV__P46662 | Glucose-6-phosphate 1-dehydrogenase X;Glucose-6-phosphate 1-dehydrogenase | G6pdx | 26.34136772 | 26.37253761 | 26.2731266 | 26.43610954 | 26.43645859 | 26.39550209 |
|  | 0.638886272 | -0.093583425 | Q8C3J5;Q5SRI3;D6RGU3;Q3TMS1 | Dedicator of cytokinesis protein 2 | Dock2 | 24.1331234 | 24.22809982 | 24.26434898 | 24.29123116 | 24.21578598 | 24.39930534 |
|  | 0.710016537 | -0.093283971 | Q9D5V6 | Synapse-associated protein 1 | Syap1 | 23.98537445 | 23.90085793 | 24.07316399 | 24.0807457 | 24.02115059 | 24.13735199 |
|  | 0.621559285 | -0.093016307 | Q9JHJ0;A0A1L1SQ12;A0A1L1SR42 | Tropomodulin-3 | Tmod3 | 23.91706848 | 24.12896156 | 24.02123642 | 24.15923309 | 24.06324005 | 24.12384224 |
|  | 2.016574158 | -0.092927297 | Q9Z1Q5 | Chloride intracellular channel protein 1 | Clic1 | 27.27817154 | 27.30582619 | 27.25115013 | 27.34688759 | 27.38442802 | 27.38261414 |
|  | 0.583383307 | -0.092575709 | Q8R016;E9PZH4 | Bleomycin hydrolase | Blmh | 23.914608 | 23.92134285 | 23.99955177 | 23.94904518 | 23.99903488 | 24.16514969 |
|  | 2.852568498 | -0.092449188 | G5E924;Q8R081;G3UY38;G3UY56 | Heterogeneous nuclear ribonucleoprotein L | Hnrnpl | 24.75735474 | 24.73928452 | 24.72801018 | 24.82502747 | 24.85004997 | 24.82691956 |
|  | 1.116027155 | -0.092030843 | P17182;Q6PHC1;B0QZL1;B1ARR7;P21550;P17183;B1ARR6;A0A0N4SUX5;D3Z6E4;D3Z2S4;J3QPZ9;Q5SX59;A0A0N4SUI6;D3YVD3;Q5SX60;Q5SX61;A0A0N4SUW8 | Alpha-enolase | Eno1 | 28.98021889 | 29.08405685 | 28.96837425 | 29.12738419 | 29.09408569 | 29.08727264 |
|  | 2.713068687 | -0.091803869 | Q9WTR1 | Transient receptor potential cation channel subfamily V member 2 | Trpv2 | 24.69333267 | 24.70530128 | 24.6915226 | 24.80523491 | 24.76542091 | 24.79491234 |
|  | 1.016473147 | -0.091625849 | Q99LR1;D6RFU2;D6RI21;F7BHM8 | Monoacylglycerol lipase ABHD12 | Abhd12 | 25.00285912 | 25.07447243 | 24.98789215 | 25.17371368 | 25.10549545 | 25.06089211 |
|  | 2.007496145 | -0.091060003 | B7FAU9;Q8BTM8;B7FAV1;F6XC15;J3JS91;F6Z2C0;F7AVL7;A0A668KLG7 | Filamin-A | Flna | 27.4718132 | 27.42534447 | 27.43021774 | 27.53289413 | 27.5564003 | 27.51126099 |
|  | 0.84848568 | -0.08995374 | Q9WUA2;A0A087WS80;A0A087WPV4;A0A087WQ15 | Phenylalanine--tRNA ligase beta subunit | Farsb | 24.28686714 | 24.13946152 | 24.23972511 | 24.35749817 | 24.28199577 | 24.29642105 |
|  | 0.274475326 | -0.08931605 | Q9WU28;E9Q5Q8;E9Q093;E9PVG3;E9PZ62;H7BWX1 | Prefoldin subunit 5 | Pfdn5 | 23.16135597 | 23.07874489 | 22.89587402 | 22.92603874 | 23.24626541 | 23.23161888 |
|  | 2.979867907 | -0.089127858 | Q8R0X7;D6REF7;D3YZT4;D3YY13;D3Z1Z3 | Sphingosine-1-phosphate lyase 1 | Sgpl1 | 26.70846176 | 26.74072647 | 26.7167263 | 26.81884956 | 26.80538177 | 26.80906677 |
|  | 1.074446877 | -0.088859558 | O35639;Q3TET3;A0A0G2JDV9;A0A0G2JGL7 | Annexin A3 | Anxa3 | 26.16798401 | 26.18008423 | 26.12419701 | 26.31487465 | 26.22383499 | 26.20013428 |
|  | 0.311250345 | -0.088015874 | Q9CQN1 | Heat shock protein 75 kDa, mitochondrial | Trap1 | 23.63245201 | 23.76040268 | 23.61571312 | 23.82386208 | 23.54992485 | 23.89882851 |
|  | 0.781486255 | -0.087290446 | P08905 | Lysozyme C-2 | Lyz2 | 30.573349 | 30.56656075 | 30.69569397 | 30.65957451 | 30.6801815 | 30.75771904 |
|  | 1.354198139 | -0.087217331 | Q91YR9;REV__A0A0R4J177;REV__Q3TVC7 | Prostaglandin reductase 1 | Ptgr1 | 24.68917847 | 24.60585976 | 24.66383743 | 24.74355316 | 24.70872498 | 24.76824951 |
|  | 0.318130715 | -0.086130142 | P70699;F6R5R5;F6VEG4;A2AFL3;A2AFL5 | Lysosomal alpha-glucosidase | Gaa | 23.00343513 | 23.22001648 | 23.08613586 | 23.2734127 | 23.00713539 | 23.28742981 |
|  | 0.14492193 | -0.085903168 | A0A0R4J1R7;Q9CZL5 | Pterin-4-alpha-carbinolamine dehydratase 2 | Pcbd2 | 22.86183357 | 22.84964943 | 22.71948242 | 23.25003052 | 22.932127 | 22.50651741 |
|  | 0.241366883 | -0.085742315 | Q8VD75;A0A0J9YUA3 | Huntingtin-interacting protein 1 | Hip1 | 23.2389679 | 23.34258842 | 22.99922371 | 23.15069008 | 23.2193222 | 23.46799469 |
|  | 0.503679528 | -0.085323334 | Q91WS0 | CDGSH iron-sulfur domain-containing protein 1 | Cisd1 | 22.38897896 | 22.41975021 | 22.61479378 | 22.53236198 | 22.60356331 | 22.54356766 |
|  | 1.274802046 | -0.085167567 | P20029 | 78 kDa glucose-regulated protein | Hspa5 | 27.35590363 | 27.37890053 | 27.38055229 | 27.48975945 | 27.48469925 | 27.39640045 |
|  | 0.835068479 | -0.085132599 | P10852;A0A0U1RP98;A0A0U1RPK4;A0A0U1RPL8;A0A0U1RPG5;A0A0U1RP32;A0A0U1RPQ7 | 4F2 cell-surface antigen heavy chain | Slc3a2 | 24.55045319 | 24.51562881 | 24.58106995 | 24.55443954 | 24.70350647 | 24.64460373 |
|  | 1.409738934 | -0.084962845 | G3UZP7;P01899;P01897;Q8HWB2;P01900;P01898;P01896;G3UXE9;E9Q0G4;O19441;A7VMS6;E9PX63;Q4KN81;P14427;P01895;V9GXR0;V9GXI4 | H-2 class I histocompatibility antigen, D-B alpha chain;H-2 class I histocompatibility antigen, L-D alpha chain | H2-D1;H2-L | 25.36799622 | 25.4255352 | 25.34150314 | 25.45205307 | 25.48953056 | 25.44833946 |
|  | 0.247622793 | -0.084732056 | D3Z3W9;F8VQ94;P97484;E9Q6M9;A0A0B4J1F3;A0A0U1RP96;D3YYZ3;D3Z674;A0A087WSB3;D3Z678;F6PZL4 | Leukocyte immunoglobulin-like receptor subfamily B member 3 | Pira2;Lilrb3;Lilra6 | 23.35769844 | 23.25623512 | 23.01144218 | 23.40509605 | 23.35528183 | 23.11919403 |
|  | 0.242163969 | -0.084720612 | Q8BUU7;Q68FH4;B7ZCT5;B7ZCT4;B7ZCT3 | N-acetylgalactosamine kinase | Galk2 | 23.66698265 | 23.31202126 | 23.6971035 | 23.58233261 | 23.5817585 | 23.76617813 |
|  | 0.26573169 | -0.084619522 | D3Z7M9;O35381;F6UFG6;D3YYE1;Q64G17 | Acidic leucine-rich nuclear phosphoprotein 32 family member A | Anp32a | 23.22380638 | 23.10685349 | 23.18131638 | 23.50032997 | 23.12971687 | 23.13578796 |
|  | 1.776555666 | -0.084512711 | A0A1W2P6F6;A0A1W2P7Q9;Q60605;Q8CI43 | Myosin light polypeptide 6 | Myl6 | 28.11713791 | 28.08496857 | 28.1155014 | 28.22006607 | 28.1950264 | 28.15605354 |
|  | 0.615893031 | -0.084272385 | Q9JJ28 | Protein flightless-1 homolog | Flii | 23.75562286 | 23.70366478 | 23.8345108 | 23.93975067 | 23.83161354 | 23.77525139 |
|  | 0.385986342 | -0.084142685 | Q9DCC5;P23198;D3Z1A9;D3Z313 | Chromobox protein homolog 3 | Cbx3 | 24.02917862 | 24.00598907 | 24.17073822 | 24.09232712 | 24.30375481 | 24.06225204 |
|  | 0.147669878 | -0.084067663 | P15532;Q5NC80;Q5NC79 | Nucleoside diphosphate kinase A;Nucleoside diphosphate kinase | Nme1 | 25.08707428 | 25.07696152 | 25.22471809 | 25.62134361 | 24.95353699 | 25.06607628 |
|  | 1.222094814 | -0.083096186 | Q8C2Q7;O35737 | Heterogeneous nuclear ribonucleoprotein H;Heterogeneous nuclear ribonucleoprotein H, N-terminally processed | Hnrnph1 | 25.15545464 | 25.15479851 | 25.09285164 | 25.16913223 | 25.24568558 | 25.23757553 |
|  | 0.803931271 | -0.082832972 | F6SUM2;O70496;E9PYL4;A0A3B2W4I8 | H(+)/Cl(-) exchange transporter 7;Chloride channel protein | Clcn7 | 23.56453514 | 23.57091713 | 23.53492737 | 23.56197357 | 23.72223663 | 23.63466835 |
|  | 1.014848848 | -0.082241694 | Q9CQE5;A0A0U1RPU5;D3Z1B6 | Regulator of G-protein signaling 10 | Rgs10 | 24.01954079 | 24.14483833 | 24.10876846 | 24.1696682 | 24.18812561 | 24.16207886 |
|  | 0.316986328 | -0.080622991 | A0A087WRN1;A0A087WQA0;F8WI22;Q8K019;A0A087WRF8 | Bcl-2-associated transcription factor 1 | Bclaf1 | 22.87047577 | 23.12224579 | 22.88596916 | 23.00123596 | 23.16679001 | 22.95253372 |
|  | 0.218429174 | -0.080077489 | Q8C1E7;D3Z0U3 | Transmembrane protein 120A | Tmem120a | 23.03020668 | NaN | 23.08670235 | 23.11414909 | 22.96770287 | 23.33374405 |
|  | 1.195100317 | -0.079994837 | Q8BX70;A0A1L1SUY8;A0A1L1SS63 | Vacuolar protein sorting-associated protein 13C | Vps13c | 22.97970009 | 23.080616 | 23.02902794 | 23.12128258 | 23.08602142 | 23.12202454 |
|  | 0.485805927 | -0.079880079 | O35345;Q4FJZ2 | Importin subunit alpha-7;Importin subunit alpha | Kpna6 | 22.29972267 | 22.48187828 | 22.39675903 | NaN | 22.4630127 | 22.48232079 |
|  | 0.668964544 | -0.079874039 | Q99LF4 | tRNA-splicing ligase RtcB homolog | Rtcb | 23.76880455 | 23.7522583 | 23.8764267 | 23.93111801 | 23.90011978 | 23.80587387 |
|  | 0.386339793 | -0.079449336 | Q8BIJ7 | RUN and FYVE domain-containing protein 1 | Rufy1 | 22.86527252 | 22.99050903 | 23.00068665 | 23.06871223 | 23.13866425 | 22.88743973 |
|  | 0.326998221 | -0.079081853 | Q8C2Q3;E9QL13;J3QN51;J3QPT3;B0LM42;F7BGR7;J3QQ01 | RNA-binding protein 14 | Rbm14 | 22.93484688 | 23.1396637 | 23.00540543 | 23.04301643 | 23.26313019 | 23.01101494 |
|  | 1.319349924 | -0.078687032 | P60843 | Eukaryotic initiation factor 4A-I | Eif4a1 | 26.86832428 | 26.90265274 | 26.9082756 | 26.95858955 | 27.02009201 | 26.93663216 |
|  | 0.335166793 | -0.078368505 | A3KGU5;A3KGU7;E9Q447;A3KGU9;P16546;A3KGU4 | Spectrin alpha chain, non-erythrocytic 1 | Sptan1 | 23.00771713 | 23.05031013 | 23.05897141 | 23.2006321 | 23.2239666 | 22.92750549 |
|  | 1.431573698 | -0.078360875 | P47738;A0A0G2JEU1;A0A0G2JF60;A0A0G2JFQ0;D3YYF3;Q62148;A0A0E2W2Y8;P24549;O35945;A0A0E2W6F6 | Aldehyde dehydrogenase, mitochondrial | Aldh2 | 27.82189369 | 27.87009048 | 27.83267593 | 27.953825 | 27.88191986 | 27.92399788 |
|  | 0.426911887 | -0.077941259 | P45377 | Aldose reductase-related protein 2 | Akr1b8 | 25.46023369 | 25.62198639 | 25.72471046 | 25.70503807 | 25.67111969 | 25.66459656 |
|  | 0.615853258 | -0.077813466 | P20065 | Thymosin beta-4;Hematopoietic system regulatory peptide | Tmsb4x | 28.2365818 | 28.38763618 | 28.32530212 | 28.41183281 | 28.32500267 | 28.44612503 |
|  | 0.235117697 | -0.076345444 | D6RE33;A0A0R4J1Q0;G5E896;Q3UJB9;F6ZJ27;F6V5I7 | Enhancer of mRNA-decapping protein 4 | Edc4 | 22.74945831 | 22.33845901 | 22.40877914 | 22.56592941 | 22.60186577 | 22.55793762 |
|  | 1.202016802 | -0.076276779 | K3W4T3;Q9Z1G4;A2A599 | V-type proton ATPase subunit a;V-type proton ATPase 116 kDa subunit a isoform 1 | Atp6v0a1 | 24.61969376 | 24.63538933 | 24.60133171 | 24.67060661 | 24.75123596 | 24.66340256 |
|  | 0.165521405 | -0.075029373 | P67871;G3UXG7;G3UZJ5;G3UZA4;G3UXU2;G3UZX4;G3UWU5 | Casein kinase II subunit beta | Csnk2b | 23.19070625 | 23.3730526 | 22.88020706 | 23.31893158 | 23.03907204 | 23.31105042 |
|  | 0.142983359 | -0.074976921 | P97808;F6TWM7;F8WJA1;E0CXN1;A0A0J9YUX0;E0CYX7;E0CXM5 | FXYD domain-containing ion transport regulator 5 | Fxyd5 | NaN | 22.19937325 | 22.43132591 | NaN | 22.52981377 | 22.25083923 |
|  | 1.143046086 | -0.074849447 | A0A0R4J0I9;Q91ZX7;Q3U5J2;D3Z5M3 | Prolow-density lipoprotein receptor-related protein 1;Low-density lipoprotein receptor-related protein 1 85 kDa subunit;Low-density lipoprotein receptor-related protein 1 515 kDa subunit;Low-density lipoprotein receptor-related protein 1 intracellular domain | Lrp1 | 25.10673332 | 25.09655571 | 25.06780052 | 25.21537971 | 25.16357613 | 25.11668205 |
|  | 0.529372968 | -0.074826558 | Q9Z0N1;A2AAW9 | Eukaryotic translation initiation factor 2 subunit 3, X-linked | Eif2s3x | 25.21881866 | 25.41989136 | 25.36372566 | 25.38788223 | 25.39666176 | 25.44237137 |
|  | 0.681624164 | -0.074274699 | A0A2I3BRL8;Q91VM5;A0A2I3BQC0;S4R1F6;Q9DAE2 | RNA binding motif protein, X-linked-like-1 | Rbmxl1 | 24.51911354 | 24.60291862 | 24.57143784 | 24.71203613 | 24.64141083 | 24.56284714 |
|  | 1.027278092 | -0.07416598 | Q99L04 | Dehydrogenase/reductase SDR family member 1 | Dhrs1 | 23.97631264 | 24.05490303 | 24.07594109 | 24.13469124 | 24.11259079 | 24.08237267 |
|  | 0.836517698 | -0.073392232 | Q60668;F6ZV59;G5E8G0;G3X9W0;E9Q5B6;F6SHF3;Q9D3U4 | Heterogeneous nuclear ribonucleoprotein D0 | Hnrnpd | 26.16829109 | 26.13129997 | 26.16597366 | 26.15946388 | 26.29409027 | 26.23218727 |
|  | 0.336264117 | -0.072588603 | P70268;D6RH37 | Serine/threonine-protein kinase N1 | Pkn1 | 22.98421097 | 22.8903923 | 23.03887177 | 22.89834976 | 23.06818581 | 23.16470528 |
|  | 0.445663837 | -0.071936289 | Q5SVG5;Q5SVG4;O35643 | AP-1 complex subunit beta-1 | Ap1b1 | 24.18434715 | 24.2146759 | 24.10501671 | 24.27717781 | 24.32230568 | 24.12036514 |
|  | 0.370268002 | -0.071687698 | Q9WTX6;Q3TPM3;A0A0N4SUW1;D3Z2H3 | Cullin-1 | Cul1 | 23.38284492 | 23.22035599 | 23.11688042 | 23.35245514 | 23.31326675 | 23.26942253 |
|  | 0.274320506 | -0.071669896 | Q9ER00 | Syntaxin-12 | Stx12 | 24.81401634 | 24.83855629 | 24.53082657 | 24.72936058 | 24.82905197 | 24.83999634 |
|  | 0.288761427 | -0.071589788 | Q99L27;A0A2I3BQX8;A0A2I3BRT9 | GMP reductase 2 | Gmpr2 | 23.77223396 | 23.64603424 | 23.80183411 | 23.7602005 | 23.98320198 | 23.69146919 |
|  | 0.465853627 | -0.071350098 | Q61334 | B-cell receptor-associated protein 29 | Bcap29 | 22.8397274 | 22.95820999 | 22.90652084 | 22.96979332 | 23.07249451 | 22.8762207 |
|  | 0.319203896 | -0.071154277 | Q62189;D3Z0S6;D6RI83 | U1 small nuclear ribonucleoprotein A | Snrpa | 23.20269585 | 23.02440262 | 23.31257439 | 23.24803352 | 23.31478882 | 23.19031334 |
|  | 1.31096066 | -0.070866267 | P63017;Q504P4;P17156;D3Z5E2;Q61696;P16627;P17879 | Heat shock cognate 71 kDa protein | Hspa8 | 28.51675606 | 28.60105515 | 28.56503677 | 28.64516068 | 28.62435913 | 28.62592697 |
|  | 0.864826717 | -0.070782979 | Q8CI94 | Glycogen phosphorylase, brain form | Pygb | 24.24495888 | 24.34915161 | 24.27248764 | 24.36979485 | 24.31775856 | 24.39139366 |
|  | 0.256027626 | -0.070370356 | P51859;E0CXA0;E0CYW7 | Hepatoma-derived growth factor | Hdgf | 24.26004601 | 24.53445244 | 24.21423149 | 24.36024666 | 24.49466515 | 24.3649292 |
|  | 0.06480299 | -0.068363825 | Q8VDM6 | Heterogeneous nuclear ribonucleoprotein U-like protein 1 | Hnrnpul1 | 23.24381256 | 23.28234863 | 22.1831665 | 23.03257942 | 22.828228 | 23.05361176 |
|  | 1.059087657 | -0.068266551 | Q6ZQM8;D3YZ96;D6RH94 | UDP-glucuronosyltransferase 1-7C | Ugt1a7c | 25.62998199 | 25.69370461 | 25.72543907 | 25.77401924 | 25.73843384 | 25.74147224 |
|  | 0.081700662 | -0.068101247 | Q9ESY9 | Gamma-interferon-inducible lysosomal thiol reductase | Ifi30 | 23.3240242 | 22.64138794 | 23.34583855 | 23.51400375 | 22.89094925 | 23.11060143 |
|  | 0.580248917 | -0.067883174 | P63037;B1AXY1;B1AXY0;B1AXX9 | DnaJ homolog subfamily A member 1 | Dnaja1 | 23.81977654 | 23.75440025 | 23.86580276 | 23.96137428 | 23.82687187 | 23.85538292 |
|  | 1.774961633 | -0.067279816 | P37040;Q05DV1;E9Q997;E9PVT9;F6R7H8 | NADPH--cytochrome P450 reductase | Por | 26.29638672 | 26.3341713 | 26.29123116 | 26.36434364 | 26.39519119 | 26.36409378 |
|  | 0.267305682 | -0.067217509 | Q4VAA2;A0A087WNP6;A0A087WRM0;A0A087WS49;F8WGL9 | Protein CDV3 | Cdv3 | 24.55531883 | 24.41712379 | 24.62343788 | 24.55168533 | 24.75552177 | 24.49032593 |
|  | 0.884479555 | -0.067109426 | P23116;A0A494B9I0 | Eukaryotic translation initiation factor 3 subunit A | Eif3a | 24.71570587 | 24.70435143 | 24.73443413 | 24.82303619 | 24.71690941 | 24.8158741 |
|  | 0.778714238 | -0.066120783 | Q9WU78 | Programmed cell death 6-interacting protein | Pdcd6ip | 24.99528694 | 25.08614349 | 24.96455193 | 25.10169411 | 25.08840942 | 25.05424118 |
|  | 0.667155398 | -0.065883001 | P14824;F8WIT2 | Annexin A6;Annexin | Anxa6 | 25.29508972 | 25.19102859 | 25.19692993 | 25.34742928 | 25.24604797 | 25.28722 |
|  | 0.707120992 | -0.06532224 | F6UP77;F7CUP3;Q8JZV7 | Putative N-acetylglucosamine-6-phosphate deacetylase | Amdhd2 | 23.54155922 | 23.61065483 | 23.5946846 | 23.72088051 | 23.61178017 | 23.6102047 |
|  | 0.794316656 | -0.064704259 | Q8JZU2;F6VVY4 | Tricarboxylate transport protein, mitochondrial | Slc25a1 | 24.39923859 | 24.28778267 | 24.38442802 | 24.40522575 | 24.41079903 | 24.44953728 |
|  | 0.920783221 | -0.064522425 | O55143;Q8R429 | Sarcoplasmic/endoplasmic reticulum calcium ATPase 2 | Atp2a2 | 24.92220497 | 24.99757195 | 24.90384674 | 24.97648811 | 25.030653 | 25.01004982 |
|  | 0.135619245 | -0.06431516 | Q9QUR7;Q3ULQ2 | Peptidyl-prolyl cis-trans isomerase NIMA-interacting 1 | Pin1 | 23.65173721 | 23.40717316 | 23.45362473 | 23.78526497 | 23.65937996 | 23.26083565 |
|  | 0.390196638 | -0.064281464 | Q9EST5 | Acidic leucine-rich nuclear phosphoprotein 32 family member B | Anp32b | 25.30841446 | 25.31610107 | 25.20664978 | 25.23209572 | 25.35356522 | 25.43834877 |
|  | 0.42167834 | -0.064278285 | E9PY39;Q99LQ7 | Predicted gene 20431 | Gm20431 | 24.6725502 | 24.71114349 | 24.72525597 | 24.72847748 | 24.89059639 | 24.68271065 |
|  | 0.819534539 | -0.063596725 | O88746;Q3UDC3;A0A1D5RM45;A0A1D5RL90;A0A1D5RM84 | Target of Myb protein 1 | Tom1 | 23.93849564 | 23.82434845 | 23.89328384 | 23.92840958 | 23.97517586 | 23.94333267 |
|  | 0.578142985 | -0.063458761 | Q07797;E9Q5X5 | Galectin-3-binding protein | Lgals3bp | 25.38238335 | 25.29284477 | 25.40002251 | 25.42123985 | 25.48440742 | 25.35997963 |
|  | 0.451101908 | -0.063168844 | Q9CQF9;D3Z275;F7CIP8;A0A0N4SWD3 | Prenylcysteine oxidase | Pcyox1 | 23.70366478 | 23.66676521 | 23.65981483 | 23.62711716 | 23.76789665 | 23.82473755 |
|  | 0.29676923 | -0.063048681 | Q80YW0;A0A2R8VI37;A0A2R8VKC6;A0A2R8VHE0;A0A2R8W712 | Cytohesin-4 | Cyth4 | 22.43697739 | 22.53278923 | 22.33005524 | 22.6221199 | 22.44447899 | 22.422369 |
|  | 0.251846469 | -0.062349319 | P63094;Q6R0H7;A0A571BEI3;Z4YKV1;A0A571BE25;A0A571BEM2;A0A571BEG2;A2A610;Q66L47;Q8CGK7;A0A571BGH5 | Guanine nucleotide-binding protein G(s) subunit alpha isoforms short;Guanine nucleotide-binding protein G(s) subunit alpha isoforms XLas | Gnas | 23.2588253 | 23.39675903 | 23.57761574 | 23.43173409 | 23.53966904 | 23.44884491 |
|  | 0.392076564 | -0.06219991 | Q8R317 | Ubiquilin-1 | Ubqln1 | 23.96269989 | 23.8237648 | 24.01869202 | 24.05424118 | 23.99938011 | 23.93813515 |
|  | 0.359351496 | -0.062093099 | P99029;G3UZJ4;H3BJQ7;A0A494BAZ4 | Peroxiredoxin-5, mitochondrial | Prdx5 | 26.36917877 | 26.46365166 | 26.45885849 | 26.47024727 | 26.61498451 | 26.39273643 |
|  | 0.260192057 | -0.061976751 | P55264;A0A286YCD7;A0A286YCN3 | Adenosine kinase | Adk | 23.61470413 | 23.70419312 | 23.90985489 | 23.73727417 | 23.81264496 | 23.86476326 |
|  | 0.68894545 | -0.061784744 | Q921T2;J3QQ40;Q3UE61;D3YXF8 | Torsin-1A-interacting protein 1 | Tor1aip1 | 24.24814796 | 24.11203384 | 24.15390968 | 24.2382679 | 24.24147224 | 24.21970558 |
|  | 1.051992012 | -0.061778386 | Q8BPU7;A0A1Y7VIX9 | Engulfment and cell motility protein 1 | Elmo1 | 24.48259163 | 24.50827217 | 24.43052101 | 24.51044655 | 24.53261185 | 24.56366158 |
|  | 0.341103694 | -0.061670303 | Q9CZ44;A2AT02 | NSFL1 cofactor p47 | Nsfl1c | 24.18737221 | 24.09619331 | 24.02732468 | 24.07594109 | 24.27589989 | 24.14406013 |
|  | 0.143892991 | -0.061589559 | P63276 | 40S ribosomal protein S17 | Rps17 | 25.55024719 | 25.5681057 | 25.62511063 | 25.34928703 | 25.691576 | 25.88736916 |
|  | 1.268944915 | -0.061537425 | P10649;A2AE89;F6WHQ7;D3YVP5;Q80W21;P19639;D3YVP6;E9QAC8;P15626;D3YX76;Q8R5I6;D3YZ29;A2AE91;D3YVP9;D3YVP8;O35660;G5E8M7 | Glutathione S-transferase Mu 1 | Gstm1 | 28.1652832 | 28.11852455 | 28.13261414 | 28.22614288 | 28.20932007 | 28.16557121 |
|  | 0.237597136 | -0.061377525 | H3BLL3;Q921G6;H3BIX9;H3BLB4;H3BJU9 | Leucine-rich repeat and calponin homology domain-containing protein 4 | Lrch4 | NaN | 21.89463615 | 21.8239994 | 21.83422089 | NaN | 22.00716972 |
|  | 0.455636593 | -0.061352412 | A0A498WGD8;Q8CDN6;A0A494B955;A0A494BBI1;A0A494BB31 | Thioredoxin-like protein 1 | Txnl1 | 25.36479568 | 25.39460182 | 25.303545 | 25.38610649 | 25.51589966 | 25.34499359 |
|  | 1.12488647 | -0.061294556 | Q61205;D3Z2X5;Q8CA83;D3Z7E6 | Platelet-activating factor acetylhydrolase IB subunit gamma | Pafah1b3 | 22.94685173 | 22.96047401 | 22.93766975 | 22.99071693 | 23.05870628 | 22.97945595 |
|  | 1.757374039 | -0.060914993 | O89053;G3UYK8;A0A0U1RPY8;D3YW57;G3UX53;D3YXM2 | Coronin-1A;Coronin | Coro1a | 26.99042702 | 27.02284431 | 27.00667381 | 27.08547592 | 27.04353905 | 27.07367516 |
|  | 0.197801778 | -0.060529073 | Q3UDS7;A0A1L1SSF2;Q8VDL4 | ADP-dependent glucokinase | Adpgk | 23.40119362 | 23.0315876 | 23.06953049 | 23.21268845 | 23.24189377 | 23.22931671 |
|  | 0.355002604 | -0.060373942 | P01901;A0A494BA33;Q3TH01;A0A494BAT0;G3UXW2;P04223;P14428;P03991;P14426;A0A0B4J1G3;A0A494B9G8;A0A494B9G2 | H-2 class I histocompatibility antigen, K-B alpha chain;H-2 class I histocompatibility antigen, K-K alpha chain;H-2 class I histocompatibility antigen, K-Q alpha chain;H-2 class I histocompatibility antigen, K-W28 alpha chain | H2-K1 | 23.4453125 | 23.4299469 | 23.22754097 | 23.44480705 | 23.42534447 | 23.41377068 |
|  | 1.114882254 | -0.05991745 | P62806 | Histone H4 | Hist1h4a | 30.26662445 | 30.30964661 | 30.32420731 | 30.35437012 | 30.39447975 | 30.33138084 |
|  | 0.389880941 | -0.059861501 | Q64514;A0A087WRC0;Q05DJ9;A0A087WP19;A0A087WQR6 | Tripeptidyl-peptidase 2 | Tpp2 | 23.75816727 | 23.85243416 | 23.94091606 | 23.86003304 | 23.98372269 | 23.88734627 |
|  | 0.088296994 | -0.059628487 | Q9QZS3;Q05BE7;A0A1Y7VJJ7;D3Z7J1;F8WJ71;D3Z549;D3Z7I6;D3Z1E8 | Protein numb homolog | Numb | NaN | 22.76480293 | 23.00792313 | NaN | 22.75633621 | 23.13564682 |
|  | 0.509052674 | -0.058675766 | A0A140LHL5;Q8VDQ8 | NAD-dependent protein deacetylase sirtuin-2 | Sirt2 | 23.72619247 | 23.77384377 | 23.83238792 | 23.91533661 | 23.78596306 | 23.80715179 |
|  | 0.685241062 | -0.058303197 | Q8VEK3 | Heterogeneous nuclear ribonucleoprotein U | Hnrnpu | 25.98133087 | 26.05312347 | 26.02669144 | 26.132967 | 26.0826149 | 26.02047348 |
|  | 0.488869739 | -0.057739258 | Q7TMB8;A0A0R4J119;A0A0U1RQ05;G3UZI5 | Cytoplasmic FMR1-interacting protein 1 | Cyfip1 | 24.77364159 | 24.73748016 | 24.70149803 | 24.88855362 | 24.73814964 | 24.75913429 |
|  | 0.078319434 | -0.057681402 | D3Z7C0;Q9Z2Q5 | 39S ribosomal protein L40, mitochondrial | Mrpl40 | 21.94973946 | 21.70708275 | 21.6749115 | 22.27514648 | 21.79950523 | 21.43012619 |
|  | 0.608383612 | -0.057616552 | P11438 | Lysosome-associated membrane glycoprotein 1 | Lamp1 | 28.24118042 | 28.33432579 | 28.25583839 | 28.32336998 | 28.3933754 | 28.28744888 |
|  | 0.071096116 | -0.057392756 | A0A0R4J0B4;Q99KK2;A0A0N4SW65;A0A0N4SWC3 | N-acylneuraminate cytidylyltransferase | Cmas | 22.50008774 | 23.16375351 | 22.95007896 | 23.08068085 | 22.77671814 | NaN |
|  | 0.530296186 | -0.056851705 | Q6R5N8 | Toll-like receptor 13 | Tlr13 | 24.03347015 | 24.02276039 | 24.14219093 | 24.16292381 | 24.0694828 | 24.13656998 |
|  | 0.343009234 | -0.056826909 | Q80ZP8;Q3TMX5;Q9CXI5;F6USD5;F6T4L3;F7C1S6 | Mesencephalic astrocyte-derived neurotrophic factor | Manf | 24.83990097 | 24.84047699 | 24.74555588 | 24.9666214 | 24.87366104 | 24.75613213 |
|  | 0.212714474 | -0.056467056 | E9QAS4;E9QAS5;Q6PDQ2;F6WR45 | Chromodomain-helicase-DNA-binding protein 4 | Chd4 | 22.18585968 | 21.97962952 | 22.29527283 | 22.14587975 | 22.18682671 | 22.29745674 |
|  | 0.638852178 | -0.056460063 | Q9ES52;F7BZA7 | Phosphatidylinositol 3,4,5-trisphosphate 5-phosphatase 1 | Inpp5d | 24.1153717 | 24.02123642 | 24.14359283 | 24.1380558 | 24.13210487 | 24.17942047 |
|  | 0.621140464 | -0.056276957 | Q8BWY3 | Eukaryotic peptide chain release factor subunit 1 | Etf1 | 24.07537079 | 23.97744751 | 23.97727394 | 24.11481667 | 24.04535294 | 24.03875351 |
|  | 0.873113635 | -0.056019465 | P14901 | Heme oxygenase 1 | Hmox1 | 26.80193329 | 26.73233986 | 26.72572517 | 26.82323074 | 26.82977676 | 26.77504921 |
|  | 0.386854702 | -0.055992126 | Q60692 | Proteasome subunit beta type-6 | Psmb6 | 25.47041702 | 25.41302872 | 25.54291725 | 25.45236778 | 25.52384567 | 25.61812592 |
|  | 0.573571409 | -0.054854711 | P27870;E9PXI0;Q8VDU4 | Proto-oncogene vav | Vav1 | 24.5225296 | 24.3801384 | 24.4759922 | 24.52163124 | 24.4996624 | 24.52193069 |
|  | 0.708030823 | -0.054769516 | Q8BGQ7 | Alanine--tRNA ligase, cytoplasmic | Aars | 24.65910721 | 24.5952549 | 24.62226486 | 24.6369381 | 24.73809814 | 24.66589928 |
|  | 1.940543655 | -0.05475235 | P61620;Q9CYJ6;Q9JLR1;A0A0A6YVQ7;A2ATT9 | Protein transport protein Sec61 subunit alpha isoform 1;Protein transport protein Sec61 subunit alpha isoform 2 | Sec61a1;Sec61a2 | 24.58846474 | 24.62120247 | 24.59115028 | 24.66296959 | 24.64201736 | 24.66008759 |
|  | 0.104378847 | -0.054505348 | P18052;Q91V35;A0A2R8VHH6 | Receptor-type tyrosine-protein phosphatase alpha;Receptor-type tyrosine-protein phosphatase | Ptpra | 23.57369232 | 23.38113022 | 23.09337616 | 23.35393524 | 23.45387459 | NaN |
|  | 0.555466821 | -0.054348628 | P24668 | Cation-dependent mannose-6-phosphate receptor | M6pr | 25.8018837 | 25.81967926 | 25.81357574 | 25.92111588 | 25.78121758 | 25.89585114 |
|  | 0.913543041 | -0.053425471 | P05201;F7ALS6 | Aspartate aminotransferase, cytoplasmic | Got1 | 25.08505058 | 25.04109573 | 25.02702904 | 25.14460564 | 25.07377815 | 25.09506798 |
|  | 0.283310491 | -0.053216298 | Q8VEA8;D3YZQ6;D3Z4X4 | Ras-related protein Rab-7b | Rab7b;5430435G22Rik | 23.07677269 | 23.0297699 | 23.21163368 | 23.05553246 | 23.19673538 | 23.22555733 |
|  | 0.365896846 | -0.053201675 | Q9EQH2;A0A1Y7VMT6;A0A1Y7VNY4 | Endoplasmic reticulum aminopeptidase 1 | Erap1 | 23.43936348 | 23.43389893 | 23.54639626 | 23.62299156 | 23.47072792 | 23.4855442 |
|  | 0.454238618 | -0.053136826 | Q9CPV4;E9Q197;F6ZTG3;E9Q055;E9Q2R6;F7BB55 | Glyoxalase domain-containing protein 4 | Glod4 | 23.83151817 | 23.91168594 | 23.77806282 | 23.91387749 | 23.83055115 | 23.93624878 |
|  | 0.186818722 | -0.052954356 | O88544;F6QTS1;D3YV99;D3Z1R9;D6RG47 | COP9 signalosome complex subunit 4 | Cops4 | 25.59684753 | 25.46123314 | 25.44002914 | 25.70885658 | 25.37630272 | 25.57181358 |
|  | 2.066042379 | -0.052919388 | P29341;Q9D4E6;Q62029;A0A2I3BR37;F6ZAX1;V9GXG3;Q8C7D3;A2A5N3 | Polyadenylate-binding protein 1 | Pabpc1 | 25.59383011 | 25.60175705 | 25.62128639 | 25.6725235 | 25.65567398 | 25.64743423 |
|  | 0.363516103 | -0.052669525 | A0A571BG95;E9PUE7;Q5SSL4;H3BKV6;H3BKT3;H3BJY3;H3BL84 | Active breakpoint cluster region-related protein | Abr | 24.72520447 | 24.6971035 | 24.58101082 | 24.79258156 | 24.71941948 | 24.64932632 |
|  | 2.02377516 | -0.052610397 | Q02053;P31254 | Ubiquitin-like modifier-activating enzyme 1 | Uba1 | 25.90547752 | 25.92073059 | 25.9061203 | 25.97072983 | 25.94344521 | 25.97598457 |
|  | 0.732472006 | -0.05260849 | P58252 | Elongation factor 2 | Eef2 | 27.5178833 | 27.57492065 | 27.59270477 | 27.59277725 | 27.66227722 | 27.58827972 |
|  | 1.761384245 | -0.052321116 | O88342;A0A0J9YU05 | WD repeat-containing protein 1 | Wdr1 | 27.60406494 | 27.60165787 | 27.60123253 | 27.62909126 | 27.67404556 | 27.66078186 |
|  | 0.489121162 | -0.052161535 | O55029 | Coatomer subunit beta | Copb2 | 24.20314407 | 24.2053833 | 24.32065392 | 24.34224892 | 24.28890991 | 24.25450706 |
|  | 0.514376641 | -0.051959356 | P30681;A0A1B0GQX9 | High mobility group protein B2 | Hmgb2 | 24.10445595 | 23.9998951 | 24.00864029 | 24.03170586 | 24.12123489 | 24.11592865 |
|  | 0.342444194 | -0.051672618 | O35286;Q497W9;A0A0G2JG10;A0A0G2JGQ5 | Pre-mRNA-splicing factor ATP-dependent RNA helicase DHX15 | Dhx15 | 23.212183 | 23.02193069 | 23.18948364 | 23.20523453 | 23.15860176 | 23.2147789 |
|  | 0.21278389 | -0.051406225 | Q9D1P4;A0A1L1STZ1;A0A1L1SU22;A0A1L1STQ1;A0A1L1SSG7 | Cysteine and histidine-rich domain-containing protein 1 | Chordc1 | 23.01387978 | 22.87271309 | 23.11181259 | 22.92524338 | 23.10442352 | 23.12295723 |
|  | 0.32455281 | -0.051364899 | Q9R1P0;E9PW69;E9Q0X0 | Proteasome subunit alpha type-4;Proteasome subunit alpha type | Psma4 | 25.37805557 | 25.29862595 | 25.46035957 | 25.34374046 | 25.45117378 | 25.49622154 |
|  | 0.139907454 | -0.050581614 | P26043;A0A5F8MPB9;Q7TSG6 | Radixin | Rdx | 23.88324928 | 23.552742 | 23.59593773 | 23.89689064 | 23.64118958 | 23.64559364 |
|  | 0.289530379 | -0.05048116 | Q3UZ39;E9Q9T1;A0A087WPK3;A0A087WSF5;G5E8E1;A0A087WNU6;A0A087WPT0 | Leucine-rich repeat flightless-interacting protein 1 | Lrrfip1 | 24.36773109 | 24.32964516 | 24.34231758 | 24.45682335 | 24.25832176 | 24.4759922 |
|  | 0.558335911 | -0.050312042 | P09411;S4R2M7 | Phosphoglycerate kinase 1;Phosphoglycerate kinase | Pgk1 | 28.50775146 | 28.59665489 | 28.60002899 | 28.57823563 | 28.66753006 | 28.60960579 |
|  | 0.543225763 | -0.049890518 | Q5SUA5;Q5SYD0 | Unconventional myosin-Ig | Myo1g | 24.08707428 | 24.05167198 | 24.12352562 | 24.20082664 | 24.13053322 | 24.08058357 |
|  | 0.673089225 | -0.049841563 | P17427;A0A0J9YUA7 | AP-2 complex subunit alpha-2 | Ap2a2 | 23.47097588 | 23.48836517 | 23.53290939 | 23.51568985 | 23.52276993 | 23.60331535 |
|  | 0.196094741 | -0.049746831 | P58044;G3XA48;H3BLF8;H3BLP1 | Isopentenyl-diphosphate Delta-isomerase 1 | Idi1 | 21.93099213 | 21.78739929 | 21.84237862 | 21.77567291 | 21.86215401 | 22.07218361 |
|  | 0.429502473 | -0.049590429 | Q60865;F6YLI0 | Caprin-1 | Caprin1 | 24.79728889 | 24.83831406 | 24.76643181 | 24.93943596 | 24.81259727 | 24.79877281 |
|  | 0.174576559 | -0.049507141 | Q60676;F7BX26 | Serine/threonine-protein phosphatase 5;Serine/threonine-protein phosphatase | Ppp5c | 23.13889885 | 23.40301704 | 23.19195557 | 23.21434975 | 23.43580818 | 23.23223495 |
|  | 0.611396575 | -0.049484889 | E9PVC5;E9PVC6;E9Q9E1;Q6NZJ6;A0A0J9YUS5;E9Q770;A0A0J9YVC1;A0A0J9YUC1;A0A0J9YUL1;D3Z439;A0A0J9YU44;A0A0J9YTV0;D3YWY7;D3YWM1 | Eukaryotic translation initiation factor 4 gamma 1 | Eif4g1 | 24.1929493 | 24.16890335 | 24.24183655 | 24.29094887 | 24.19370079 | 24.2674942 |
|  | 0.513211048 | -0.049372355 | P42932;H3BL49;H3BJB6;H3BKG2;H3BLL1;H3BKR8 | T-complex protein 1 subunit theta | Cct8 | 25.46730995 | 25.37895012 | 25.39054108 | 25.50966263 | 25.47370338 | 25.4015522 |
|  | 0.167997607 | -0.049134572 | D3Z7P0;Q8K3W0;E9Q0U3;A0A0J9YV56;E9Q8Q1 | BRCA1-A complex subunit BRE | Bre | 23.13824272 | 22.84745216 | 23.02038765 | 23.0693512 | 23.16413689 | 22.91999817 |
|  | 0.179252801 | -0.048882167 | Q9QZD8 | Mitochondrial dicarboxylate carrier | Slc25a10 | 23.00713539 | 22.71019745 | 23.021101 | 22.99474335 | 22.92101669 | 22.9693203 |
|  | 0.162635778 | -0.048879623 | P32067;A2AR07;D6RI87;F6SXM5 | Lupus La protein homolog | Ssb | 23.4913044 | 23.62934303 | 23.86740494 | 23.70123482 | 23.76182365 | 23.67163277 |
|  | 0.115329004 | -0.048774719 | P27046;F6QMB7 | Alpha-mannosidase 2 | Man2a1 | 23.13186836 | 22.85849762 | 23.06759644 | 22.80986214 | 23.21658516 | 23.17783928 |
|  | 0.185739933 | -0.048125585 | P12382;P47857;A0A1W2P7T1;A0A0E2W9U1 | ATP-dependent 6-phosphofructokinase, liver type | Pfkl | 23.67271233 | 23.46923828 | 23.63145256 | 23.79153824 | 23.5434494 | 23.58279228 |
|  | 0.18107424 | -0.047716777 | P70333 | Heterogeneous nuclear ribonucleoprotein H2 | Hnrnph2 | 24.23095894 | 24.2657795 | 24.23169136 | 24.45832634 | 24.11354637 | 24.29970741 |
|  | 0.334532393 | -0.047667185 | Q8VIJ6 | Splicing factor, proline- and glutamine-rich | Sfpq | 25.59934807 | 25.70147324 | 25.54998398 | 25.71583748 | 25.68824577 | 25.58972359 |
|  | 0.246802791 | -0.046591441 | P70362;E9Q475;E9Q646;E9Q0Y6 | Ubiquitin fusion degradation protein 1 homolog | Ufd1l | 23.21884918 | 23.34177399 | 23.18083191 | 23.40483665 | 23.25810623 | 23.21828651 |
|  | 0.211060695 | -0.046560923 | G3X922;D4AFX7;A0A087WRC9;A0A1L1STR9;A0A087WS25 | DnaJ heat shock protein family (Hsp40) member C13 | Dnajc13 | 22.76330376 | 22.93533325 | 22.74509239 | 22.7534008 | 22.86789513 | 22.96211624 |
|  | 0.185685862 | -0.046486537 | Q9D0I9 | Arginine--tRNA ligase, cytoplasmic | Rars | 24.6365509 | 24.60896492 | 24.43173409 | 24.74591446 | 24.51893425 | 24.55186081 |
|  | 0.551574416 | -0.045901616 | Q99K48 | Non-POU domain-containing octamer-binding protein | Nono | 24.7092514 | 24.69168282 | 24.74622154 | 24.81821632 | 24.76324272 | 24.70340157 |
|  | 0.146279307 | -0.045667648 | A0A0J9YUF8;A0A0J9YTU3;Q5SXY1;A0A0J9YV86;A0A0J9YUR2;A0A0J9YV47;A0A0J9YUG8 | Cytospin-B | Specc1 | 23.49142838 | 23.29319572 | 23.59650612 | 23.64987564 | 23.3998909 | 23.46836662 |
|  | 0.541423845 | -0.04559962 | Q99PT1 | Rho GDP-dissociation inhibitor 1 | Arhgdia | 28.41542435 | 28.47603035 | 28.43117523 | 28.49113655 | 28.5402298 | 28.42806244 |
|  | 0.553610509 | -0.045403798 | Q64737;D6RCG1;A0A338P6X4;A0A338P676;A0A338P6W0 | Trifunctional purine biosynthetic protein adenosine-3;Phosphoribosylamine--glycine ligase;Phosphoribosylformylglycinamidine cyclo-ligase;Phosphoribosylglycinamide formyltransferase | Gart | 24.05490303 | 24.03464699 | 24.02276039 | 24.06997299 | 24.02951622 | 24.14903259 |
|  | 0.545837071 | -0.045356115 | P14115 | 60S ribosomal protein L27a | Rpl27a | 26.98370171 | 27.0866909 | 27.09012794 | 27.11485672 | 27.10499573 | 27.07673645 |
|  | 0.418256486 | -0.045324326 | Q6A028 | Switch-associated protein 70 | Swap70 | 23.73902702 | 23.71931458 | 23.73634529 | 23.69083023 | 23.79302788 | 23.84680176 |
|  | 0.048343773 | -0.045050303 | Q80YQ8;D6RII5 | Protein RMD5 homolog A | Rmnd5a;Rmnd5b | 21.38732338 | 21.92299461 | 21.07201958 | 21.30891418 | 21.30111885 | 21.90745544 |
|  | 0.251469062 | -0.045020739 | Q8BZA9 | Fructose-2,6-bisphosphatase TIGAR | Tigar | 22.63880539 | 22.80762291 | 22.7763958 | 22.76375008 | 22.80817413 | NaN |
|  | 0.091927374 | -0.044039726 | Q7TS64;Q99MK8;F6Y9P3;F6QY34 | Beta-adrenergic receptor kinase 1 | Adrbk1 | NaN | 22.84900093 | 22.58203506 | 22.55285835 | 22.84237862 | 22.8834362 |
|  | 0.575171939 | -0.043649038 | Q8JZQ9;A0A0G2JG48 | Eukaryotic translation initiation factor 3 subunit B | Eif3b | 24.02191353 | 23.98215675 | 23.90774918 | 24.00530243 | 24.02115059 | 24.01631355 |
|  | 0.163238712 | -0.043417613 | Q60737;A2ANR6;Q6NSS6 | Casein kinase II subunit alpha | Csnk2a1 | 23.30688667 | 23.24966812 | 23.25767517 | 23.47766113 | 23.13758659 | 23.32923508 |
|  | 0.116028063 | -0.043323517 | Q9EPL8 | Importin-7 | Ipo7 | 22.95739555 | 22.63020897 | 22.52027702 | 22.67861176 | 22.79229355 | 22.76694679 |
|  | 0.58098432 | -0.043229421 | P60335;A0A087WR61;A0A087WSI1;A0A1L1SUS5;A0A1L1SRP9;A0A1L1SQ99;A0A087WRH3;A0A0R4J044;P57724 | Poly(rC)-binding protein 1 | Pcbp1 | 26.31030655 | 26.33169746 | 26.31518555 | 26.34302902 | 26.31810379 | 26.42574501 |
|  | 0.197520667 | -0.043153127 | Q5XJY5 | Coatomer subunit delta | Arcn1 | 24.78471565 | 24.8747406 | 24.6618824 | 24.9269619 | 24.78795624 | 24.7358799 |
|  | 0.463661791 | -0.043052673 | A0A0U1RPL0;Q7TQH0;E9Q5Q0;Q3TGG2;J3QP59 | Ataxin-2-like protein | Atxn2l | 22.74330711 | 22.77998543 | 22.69672203 | 22.72033691 | 22.82629013 | 22.80254555 |
|  | 0.115135431 | -0.042949677 | E9PUF7;Q61210;F6ZN61;A0A0U1RPP2;A0A0U1RPN7 | Rho guanine nucleotide exchange factor 1 | Arhgef1 | 23.42585564 | 23.27398109 | 23.61784554 | 23.38666534 | 23.39375114 | 23.66611481 |
|  | 0.654560327 | -0.042833328 | Q99KK7;A0A494BBC1;A0A494BA16;A0A494BBB3;A0A494B918 | Dipeptidyl peptidase 3 | Dpp3 | 24.54957199 | 24.49625206 | 24.54863167 | 24.54226875 | 24.55987549 | 24.62081146 |
|  | 0.298156933 | -0.042486827 | P68510 | 14-3-3 protein eta | Ywhah | 27.57650566 | 27.57909966 | 27.65047836 | 27.74040413 | 27.63367271 | 27.55946732 |
|  | 0.332506377 | -0.042476018 | Q9CQW1 | Synaptobrevin homolog YKT6 | Ykt6 | 23.79024506 | 23.76668358 | 23.86881828 | 23.89263535 | 23.76577377 | 23.89476585 |
|  | 0.348799529 | -0.042116165 | D3Z2H9;D3YVR0;S4R2U0;A2AIM5 | Tropomyosin 3, related sequence 7 | Tpm3-rs7 | 27.73188591 | 27.77919006 | 27.72429466 | 27.77391815 | 27.8746109 | 27.71319008 |
|  | 0.379804509 | -0.041886012 | Q3U8S1;A2APM5;A2APM3;A2APM4;E9QKM8;Q80X37;A2APM1;A2APM2;P15379 | CD44 antigen | Cd44 | 25.2975769 | 25.43240166 | 25.38165855 | 25.42572784 | 25.3649292 | 25.44663811 |
|  | 0.12111631 | -0.041301092 | Q8VE70;F8WI55;E0CY82 | Programmed cell death protein 10 | Pdcd10 | 23.26642227 | 23.65468979 | 23.58909416 | 23.5946846 | 23.55695534 | 23.48246956 |
|  | 0.08211671 | -0.04117775 | J3QMM7;K3W4M4;Q9CZ42;J3QN06;J3QPU6 | ATP-dependent (S)-NAD(P)H-hydrate dehydratase | Carkd | 22.5646286 | 22.75331879 | 22.91923523 | 22.58297539 | 22.71108055 | 23.06665993 |
|  | 0.086804308 | -0.041069984 | A0A0R4J0U7;Q8R395 | COMM domain-containing protein 5 | Commd5 | NaN | 23.9225235 | 23.64052773 | 24.00975227 | 23.67917061 | 23.77886391 |
|  | 0.218727886 | -0.040792465 | D3YZ62;D3Z4J3;Q99104;F6TDE5;B8JK05;F6Z2S4;A0A1L1SUF0;D3Z135;G5E8G6;G3X9Y9;P21271 | Unconventional myosin-Va | Myo5a | 23.49874878 | 23.67109299 | 23.43860245 | 23.54262352 | 23.6135807 | 23.57461739 |
|  | 0.2644302 | -0.040592829 | Q80VP0 | Tectonin beta-propeller repeat-containing protein 1 | Tecpr1 | 23.13836861 | 23.09039116 | 23.06849861 | 23.23000717 | 23.15673637 | 23.03229332 |
|  | 0.124570785 | -0.04046313 | Q8VBT0;F6V084 | Thioredoxin-related transmembrane protein 1 | Tmx1 | 23.76395226 | 23.86881828 | 23.83508873 | 24.05812836 | 23.87032509 | 23.66079521 |
|  | 0.36788166 | -0.040082296 | P62821 | Ras-related protein Rab-1A | Rab1A | 27.40673447 | 27.50627518 | 27.54008293 | 27.54502678 | 27.54738235 | 27.48093033 |
|  | 0.141176686 | -0.039355596 | Q9QZ08;Q9D997;D3YXG2 | N-acetyl-D-glucosamine kinase | Nagk | 24.18955994 | 24.0052166 | 24.02335358 | 23.95118141 | 24.23994446 | 24.14507103 |
|  | 0.952437159 | -0.039011637 | P54116 | Erythrocyte band 7 integral membrane protein | Stom | 26.5886364 | 26.60427666 | 26.60059547 | 26.5997448 | 26.65717316 | 26.65362549 |
|  | 0.13866213 | -0.038888931 | Q9R059;F2Z455;D6RJ68 | Four and a half LIM domains protein 3 | Fhl3 | 22.68852806 | 22.72795868 | 22.55435753 | 22.51814079 | 22.80179596 | 22.76757431 |
|  | 0.107233376 | -0.038650513 | Q8R010;Q8R3V2 | Aminoacyl tRNA synthase complex-interacting multifunctional protein 2 | Aimp2 | 23.6417408 | 23.7470417 | 23.73179626 | 23.97167206 | 23.53658867 | 23.72826958 |
|  | 0.532127478 | -0.037476858 | Q9WVK4 | EH domain-containing protein 1 | Ehd1 | 24.85152817 | 24.90094948 | 24.79615021 | 24.88171005 | 24.90058136 | 24.87876701 |
|  | 0.481824245 | -0.036176046 | Q9DBG3;H3BKM0;H3BIY9;H3BJ06;Q5SWR0 | AP-2 complex subunit beta;AP complex subunit beta | Ap2b1 | 24.76430702 | 24.73453712 | 24.67965317 | 24.78126717 | 24.71999359 | 24.78576469 |
|  | 0.326695017 | -0.036041896 | Q924B0;O55023;Q80ZJ2;A0A0A6YW07;D3Z703 | Inositol monophosphatase 1 | Impa1 | 24.68083382 | 24.54315376 | 24.66752434 | 24.67319679 | 24.68249702 | 24.64394379 |
|  | 0.683478321 | -0.036013921 | Q9D8E6 | 60S ribosomal protein L4 | Rpl4 | 26.73621559 | 26.71842575 | 26.72702408 | 26.79600143 | 26.71790314 | 26.77580261 |
|  | 0.100407608 | -0.035887082 | Q8BXZ1;A0A494BB11;A0A494B9J5;A0A494B9P2 | Protein disulfide-isomerase TMX3 | Tmx3 | 24.42835045 | 24.26999283 | NaN | 24.55590439 | 24.28524399 | 24.31402779 |
|  | 0.411713095 | -0.035687764 | Q8CIN4 | Serine/threonine-protein kinase PAK 2;PAK-2p27;PAK-2p34 | Pak2 | 24.26613617 | 24.28277206 | 24.31215858 | 24.2556591 | 24.36873055 | 24.34374046 |
|  | 0.118653079 | -0.035620372 | Q3UKN6;P81117 | Nucleobindin-2;Nesfatin-1 | Nucb2 | 23.60648155 | 23.5847435 | 23.40197563 | 23.50433731 | 23.45525551 | 23.74046898 |
|  | 0.220982074 | -0.03538386 | Q5EBP8;P49312 | Heterogeneous nuclear ribonucleoprotein A1;Heterogeneous nuclear ribonucleoprotein A1, N-terminally processed | Hnrnpa1 | 25.30983734 | 25.28238487 | 25.46276283 | 25.43135071 | 25.39276886 | 25.33701706 |
|  | 0.071784001 | -0.035277685 | A0A1W2P6G5 | Myosin, light polypeptide 6, alkali, smooth muscle and non-muscle | Myl6 | 24.53807068 | 24.74719429 | 24.48209953 | 24.51177406 | 24.43599892 | 24.92542458 |
|  | 0.092740349 | -0.035170873 | Q6P1F6;Q9CWU3;Q925E7;F6RV17;A0A668KLA9;Q6ZWR4;A0A286YDJ9;A0A494B941;G3UXS9;A0A494B8Z0;Q8BG02 | Serine/threonine-protein phosphatase 2A 55 kDa regulatory subunit B alpha isoform;Serine/threonine-protein phosphatase 2A 55 kDa regulatory subunit B | Ppp2r2a | 23.87248611 | 23.94637108 | 23.59821129 | 23.85138512 | 23.69008446 | 23.98111153 |
|  | 0.108309134 | -0.034803708 | G3X9H7;Q8R1S4;A0A2I3BQ92;A0A2I3BPM9;A0A2I3BQ67 | Metastasis suppressor protein 1 | Mtss1 | 23.22076797 | 23.19598579 | 23.02286148 | 23.28898048 | NaN | 23.07370377 |
|  | 0.198195027 | -0.034090042 | Q922Q8 | Leucine-rich repeat-containing protein 59 | Lrrc59 | 25.66966057 | 25.72811508 | 25.705513 | 25.77165413 | 25.61079597 | 25.82310867 |
|  | 0.719099177 | -0.033157984 | E9Q3Q6;Q61490;E9Q4G8;F6QH25;Q5MPX5 | CD166 antigen | Alcam | 25.20724678 | 25.1774826 | 25.16836739 | 25.24416161 | 25.18449974 | 25.22390938 |
|  | 0.110787283 | -0.032685598 | Q9Z2I9 | Succinyl-CoA ligase [ADP-forming] subunit beta, mitochondrial | Sucla2 | 24.38033676 | 24.15753746 | 24.23513031 | 24.19302368 | 24.21933556 | 24.45870209 |
|  | 0.41966846 | -0.032144547 | O08528;E9Q5B5;E9Q9M6 | Hexokinase-2;Hexokinase | Hk2 | 24.59411621 | 24.65162849 | 24.66372871 | 24.6435585 | 24.64455032 | 24.71779823 |
|  | 0.060608739 | -0.031644185 | E9Q5H2;E9PZF5;P97822;E9Q0X5 | Acidic leucine-rich nuclear phosphoprotein 32 family member E | Anp32e | 23.24813461 | 23.13671112 | 23.34610939 | 23.25551414 | 23.58026314 | 22.9901104 |
|  | 0.389135728 | -0.03162384 | Q8CIH5;E9PXZ8;Q9QZE2 | 1-phosphatidylinositol 4,5-bisphosphate phosphodiesterase gamma-2 | Plcg2 | 24.16783142 | 24.11155701 | 24.16943741 | 24.16913223 | 24.23549461 | 24.13907051 |
|  | 0.191465083 | -0.03160731 | Q9R0E1;F6W3Q8 | Procollagen-lysine,2-oxoglutarate 5-dioxygenase 3 | Plod3 | 22.3719902 | 22.338377 | 22.26842308 | 22.46821785 | 22.29401016 | 22.3113842 |
|  | 0.128338428 | -0.030632655 | P23780;A0A1L1SSJ7 | Beta-galactosidase | Glb1 | 23.95739555 | 23.86561394 | 23.89910698 | 24.06266403 | 23.77976608 | 23.97158432 |
|  | 0.160462844 | -0.030476888 | Q9CQR6;A0A0N4SVL9;A0A0N4SVE2;A0A0N4SW66 | Serine/threonine-protein phosphatase 6 catalytic subunit;Serine/threonine-protein phosphatase 6 catalytic subunit, N-terminally processed | Ppp6c | 23.72098541 | 23.61840439 | 23.62823105 | 23.65556335 | 23.5952549 | 23.80823326 |
|  | 0.143022519 | -0.030024211 | Q922D8;A0A1W2P733;A0A1W2P7L5 | C-1-tetrahydrofolate synthase, cytoplasmic;Methylenetetrahydrofolate dehydrogenase;Methenyltetrahydrofolate cyclohydrolase;Formyltetrahydrofolate synthetase;C-1-tetrahydrofolate synthase, cytoplasmic, N-terminally processed | Mthfd1 | 24.42764664 | 24.42322922 | 24.58880806 | 24.56505775 | 24.39865303 | 24.56604576 |
|  | 0.113345452 | -0.029879888 | Q9CQX2 | Cytochrome b5 type B | Cyb5b | 24.42764664 | 24.5547924 | 24.27070618 | 24.46749687 | 24.35467529 | 24.52061272 |
|  | 0.06323559 | -0.029747009 | Q8BRF7;A0A1W2P6R7 | Sec1 family domain-containing protein 1 | Scfd1 | 22.58251762 | 23.03756523 | 22.90150261 | 22.69701767 | 23.01304436 | 22.90076447 |
|  | 1.130591184 | -0.029501597 | Q9DBJ1 | Phosphoglycerate mutase 1 | Pgam1 | 28.44150162 | 28.43626785 | 28.44537544 | 28.4719677 | 28.49056244 | 28.44911957 |
|  | 0.134411929 | -0.029453913 | Q8BJY1;F7BA91 | 26S proteasome non-ATPase regulatory subunit 5 | Psmd5 | 24.34800339 | 24.49679947 | 24.57548332 | 24.51002502 | 24.42066193 | 24.57796097 |
|  | 0.181658614 | -0.029356639 | P35585;A0A1D5RLW6;D3YZ71;A0A0R4IZX6;A0A0R4J1L4;Q9WVP1;A0A1D5RLL9;A0A1D5RMJ1;A0A1L1SUA0 | AP-1 complex subunit mu-1 | Ap1m1 | 23.95162582 | 23.92741394 | 23.83961296 | 24.01767349 | 23.84134102 | 23.94770813 |
|  | 0.340543729 | -0.029257456 | P80318;E9Q133;Q3U0I3;F6Q609;F6ZVG8 | T-complex protein 1 subunit gamma | Cct3 | 25.69229507 | 25.67090416 | 25.75549698 | 25.75552177 | 25.68608284 | 25.76486397 |
|  | 0.079332085 | -0.029121399 | Q69ZK0;I7HPV9 | Phosphatidylinositol 3,4,5-trisphosphate-dependent Rac exchanger 1 protein | Prex1 | 22.15881729 | 22.20642662 | 21.89252472 | 22.188097 | 21.94563866 | 22.21139717 |
|  | 0.080494337 | -0.029038747 | A0A0G2JGX4;Q8VCE0;Q6PIC6;Q9Z1W8 | Sodium/potassium-transporting ATPase subunit alpha-3 | Atp1a3 | 23.65305138 | 23.42201042 | 23.52456284 | 23.74160004 | 23.57807732 | 23.36706352 |
|  | 0.467031341 | -0.028786977 | Q3TW96;A0A0R4J1F6;A0A0R4J085;Q3UHZ7;Q91YN5 | UDP-N-acetylhexosamine pyrophosphorylase-like protein 1 | Uap1l1 | 26.07846832 | 26.11231232 | 26.14080811 | 26.13992882 | 26.10495567 | 26.17306519 |
|  | 0.081406132 | -0.028207143 | Q99MR6;A0A0G2JG65;A0A0G2JDF8;A0A0G2JDN3;A0A1Y7VNN4;A0A0G2JDF1 | Serrate RNA effector molecule homolog | Srrt | 22.45022964 | 22.51691628 | 22.27869606 | 22.57537842 | 22.50719643 | 22.24788857 |
|  | 1.012985443 | -0.028191884 | G5E8R2;E9Q453;E9Q456;E9Q450;E9Q448;Q8BP43 | Tropomyosin 1, alpha | Tpm1 | 24.80134201 | 24.76956177 | 24.78790665 | 24.79778481 | 24.82977676 | 24.81582451 |
|  | 0.149120426 | -0.027922948 | Q7TNS2 | MICOS complex subunit Mic10 | Minos1 | 24.27824211 | 24.3521862 | 24.40691376 | 24.26685143 | 24.38396645 | 24.47029305 |
|  | 0.250407846 | -0.027359645 | G3X8T3;P16675;A2A5J8;A2A5J9 | Carboxypeptidase;Lysosomal protective protein;Lysosomal protective protein 32 kDa chain;Lysosomal protective protein 20 kDa chain | Ctsa | 26.25208855 | 26.25661278 | 26.28617859 | 26.3022213 | 26.35954285 | 26.2151947 |
|  | 0.209475874 | -0.027152379 | Q922B2;Q8BJY7 | Aspartate--tRNA ligase, cytoplasmic | Dars | 24.64372444 | 24.72426796 | 24.57271004 | 24.63428116 | 24.71894836 | 24.66893005 |
|  | 0.549312826 | -0.026896795 | P11499;E9Q3D6;E9PX27;E9Q0C3;D3Z1R1 | Heat shock protein HSP 90-beta | Hsp90ab1 | 27.85679626 | 27.90305519 | 27.85275459 | 27.89960098 | 27.92184258 | 27.87185287 |
|  | 0.155625429 | -0.026866913 | Q9Z2X1;J3QMT0;J3QM80;J3QP45;J3QNH2;J3QMV8 | Heterogeneous nuclear ribonucleoprotein F;Heterogeneous nuclear ribonucleoprotein F, N-terminally processed | Hnrnpf | 25.22177124 | 25.10041046 | 25.18763542 | 25.14934158 | 25.13739204 | 25.30368423 |
|  | 0.148309093 | -0.026672363 | Q99KI3 | ER membrane protein complex subunit 3 | Emc3 | 22.71582031 | 22.81378174 | 22.86196518 | 22.92591286 | 22.7632637 | 22.78240776 |
|  | 0.080127252 | -0.026237488 | B1AVH7 | TBC1 domain family member 2A | Tbc1d2 | 22.74118805 | 23.01830101 | 22.8775692 | 22.77972603 | 23.06312561 | 22.87291908 |
|  | 0.085877103 | -0.026060104 | P35821 | Tyrosine-protein phosphatase non-receptor type 1 | Ptpn1 | 23.9197979 | 23.74909019 | 23.6227684 | 23.69200325 | 23.91195869 | 23.76587486 |
|  | 0.078971865 | -0.025859197 | P62317 | Small nuclear ribonucleoprotein Sm D2 | Snrpd2 | 24.67335892 | 25.0098362 | 24.96547699 | 24.97057533 | 24.93862915 | 24.81704521 |
|  | 0.134126772 | -0.025744756 | Q9D0R2;A0A2I3BPK8;Q8BLY2 | Threonine--tRNA ligase, cytoplasmic | Tars | 23.80675888 | 23.66903877 | 23.62967682 | 23.79718971 | 23.7449913 | 23.64052773 |
|  | 0.083487957 | -0.025728226 | A0A1L1STE4;Q9Z1X4;Q45VK5;A0A1L1SU19;A0A1L1SQR7;A0A1L1SR62;A0A1L1SQ69;A0A1L1SSU8 | Interleukin enhancer-binding factor 3 | Ilf3 | 22.58026314 | 22.70841026 | 22.48308372 | 22.46520615 | 22.76797676 | 22.6157589 |
|  | 0.21459617 | -0.025405884 | Q9CZN7;G3UZ26;G3UYY1;P50431 | Serine hydroxymethyltransferase | Shmt2 | 24.01945496 | 24.17027855 | 24.05986214 | 24.10077286 | 24.12683678 | 24.09820366 |
|  | 0.051625424 | -0.025323868 | Q9DB29;A0A1Y7VLY5;A0A1Y7VKA0;A0A1Y7VKC6 | Isoamyl acetate-hydrolyzing esterase 1 homolog | Iah1 | 23.03728104 | 22.9711113 | 22.85045052 | 22.66414261 | 23.08668709 | 23.18398476 |
|  | 0.087389521 | -0.025312424 | A2AH25;Q5FWK3 | Rho GTPase-activating protein 1 | Arhgap1 | 24.08780289 | 24.36613083 | 24.03573799 | 24.19445229 | 24.17295074 | 24.19820595 |
|  | 0.202211979 | -0.025223732 | E9QNG1;B2RR82;Q9Z0R6;A0A1W2P775;A0A1W2P7G8 | Intersectin-2 | Itsn2 | NaN | 22.08079529 | 22.00274849 | 22.08832169 | 22.04566956 | NaN |
|  | 0.188017843 | -0.02521642 | P49710;E9Q4E5 | Hematopoietic lineage cell-specific protein | Hcls1 | 26.68632317 | 26.67559433 | 26.63353348 | 26.60837173 | 26.77680779 | 26.68592072 |
|  | 0.026008855 | -0.024765968 | P42669 | Transcriptional activator protein Pur-alpha | Pura | 23.00641632 | 23.31962013 | 23.7072506 | NaN | 23.13938332 | 23.59900665 |
|  | 0.194059544 | -0.024756749 | Q62418 | Drebrin-like protein | Dbnl | 24.72551727 | 24.65550995 | 24.70798874 | 24.68597603 | 24.80891991 | 24.66839027 |
|  | 0.129773479 | -0.023958842 | Q9JHU9 | Inositol-3-phosphate synthase 1 | Isyna1 | 23.55543518 | 23.67066002 | 23.76009941 | 23.69529915 | 23.62477684 | 23.73799515 |
|  | 0.083217348 | -0.023456573 | G3X8Y3;A0A0A6YW80;Q80UM3;A0A0A6YX86;A0A0A6YXF4;E9PZ53;Q9DBB4 | N-alpha-acetyltransferase 15, NatA auxiliary subunit | Naa15 | 23.66817284 | 23.74314308 | 23.60862541 | 23.87717628 | 23.63644028 | 23.57669449 |
|  | 0.172913548 | -0.023442586 | A0A0G2JEC4;Q9JK48;A0A0G2JF57;A0A0G2JE45 | Endophilin-B1 | Sh3glb1 | 23.34529877 | 23.18051338 | 23.23075485 | 23.30591202 | 23.26956558 | 23.25141716 |
|  | 0.131146686 | -0.023435593 | P24527 | Leukotriene A-4 hydrolase | Lta4h | 24.2624855 | 24.23855972 | 24.23659134 | 24.30410194 | 24.14312553 | 24.36071587 |
|  | 0.185642349 | -0.023317973 | Q921M7;A0A2I3BRN5;A0A2I3BPH9;A0A2I3BQK1 | Protein FAM49B | Fam49b | 24.49466515 | 24.54533577 | 24.45870209 | 24.59843826 | 24.4582634 | 24.51195526 |
|  | 0.300174263 | -0.02324295 | P27546;A0A140T8T5;A0A0G2JFH2;E9PZ43;A0A0G2JDN7;A0A0G2JG35;Q78TF3;A0A0G2JFK3;A0A0G2JDY5;A0A0G2JFT4;A0A0G2JE57;A0A0G2JDU1 | Microtubule-associated protein 4 | Map4 | 24.30187225 | 24.35716248 | 24.29984856 | 24.38205338 | 24.29572105 | 24.35083771 |
|  | 0.04718721 | -0.021935463 | P00416 | Cytochrome c oxidase subunit 3 | mt-Co3 | 23.07043266 | 23.09069633 | 23.44240379 | 23.22770309 | NaN | 23.21852303 |
|  | 0.131238572 | -0.02189064 | P35550;A0A140LIR6 | rRNA 2-O-methyltransferase fibrillarin | Fbl | 24.4596405 | 24.48511314 | 24.62282372 | 24.58686256 | 24.57039452 | 24.4759922 |
|  | 0.045828416 | -0.021772385 | Q9ES46 | Beta-parvin | Parvb | 23.14647102 | 23.29670143 | 23.5853157 | 23.55238914 | 23.32526016 | 23.21615601 |
|  | 0.447669979 | -0.021746318 | Q8BHN3 | Neutral alpha-glucosidase AB | Ganab | 24.87150002 | 24.9216156 | 24.94042206 | 24.94006348 | 24.93080139 | 24.92791176 |
|  | 0.079740732 | -0.021678925 | O55234;Q8BTY5 | Proteasome subunit beta type-5 | Psmb5 | 23.99464035 | 24.07993317 | 23.81127357 | 24.06513214 | 23.88147736 | 24.00427437 |
|  | 0.072212653 | -0.021318436 | P46737;E9Q0P6;A3KGA8 | Lys-63-specific deubiquitinase BRCC36 | Brcc3 | 22.45467949 | 22.62807465 | 22.39830017 | 22.57929802 | 22.45070839 | NaN |
|  | 0.070497857 | -0.021088918 | Q8R307 | Vacuolar protein sorting-associated protein 18 homolog | Vps18 | 24.75989532 | 24.76425552 | 24.4767952 | 24.64779091 | 24.77545166 | 24.64097023 |
|  | 0.045623193 | -0.020723343 | Q6PGL7;A0A0N4SUJ0;A0A0N4SV74 | WASH complex subunit FAM21 | Fam21 | 22.84668732 | 22.62880898 | 23.11564255 | 22.94152451 | 22.75378799 | 22.95799637 |
|  | 0.036665789 | -0.020462036 | Q8BH24 | Transmembrane 9 superfamily member 4 | Tm9sf4 | 23.50966263 | 23.21411133 | 23.31851768 | 23.69721031 | 23.14434052 | 23.26212692 |
|  | 0.097567266 | -0.020395279 | P56212;E9Q4B9;E9Q827;E9PXD7 | cAMP-regulated phosphoprotein 19 | Arpp19 | 23.9205265 | 23.76607704 | 23.80331421 | 23.91259956 | 23.90536308 | 23.73314095 |
|  | 0.157275305 | -0.020386378 | Q8BTS0;Q61656;S4R1I6;B1ARB9;B1ARC0;S4R1E3 | Probable ATP-dependent RNA helicase DDX5 | Ddx5 | 25.67720985 | 25.63170242 | 25.70688248 | 25.7788887 | 25.64463234 | 25.65343285 |
|  | 0.124720336 | -0.020338058 | O35226 | 26S proteasome non-ATPase regulatory subunit 4 | Psmd4 | 24.74883461 | 24.88762474 | 24.71214104 | 24.84071732 | 24.75190163 | 24.81699562 |
|  | 0.088724593 | -0.020327886 | Q9D8B3 | Charged multivesicular body protein 4b | Chmp4b | 25.17077637 | 25.21408272 | 24.98810959 | 25.19106674 | 25.05895424 | 25.18393135 |
|  | 0.216892717 | -0.020036697 | A0A0A0MQF6;P16858;S4R257;A0A1D5RLD8;S4R1W1;S4R1W8;A0A0R4J0X7;S4R2G5;Q64467;S4R1N5 | Glyceraldehyde-3-phosphate dehydrogenase | Gapdh;Gm3839 | 30.94055748 | 30.9143486 | 30.93269539 | 31.01404953 | 30.89352417 | 30.94013786 |
|  | 0.068582861 | -0.019833883 | Q3UIA2;E9QAJ9;A0A0U1RNM6;A0A0U1RPK6;Q5SSM3 | Rho GTPase-activating protein 17 | Arhgap17 | 23.66220856 | 23.71628189 | 23.87895393 | 23.89726067 | 23.63123131 | 23.78845406 |
|  | 0.05505848 | -0.019775391 | Q80XR5;P26369;Q3KQM4;A0A140LJK3;A0A140LJ08 | Splicing factor U2AF 65 kDa subunit | U2af2 | 23.43618965 | 23.61133003 | 23.75021553 | 23.49533653 | 23.5817585 | 23.77996635 |
|  | 0.141022625 | -0.019735972 | Q63850;A0A140LJH5;A0A140LJ77 | Nuclear pore glycoprotein p62 | Nup62 | 22.9109726 | 22.78948975 | NaN | 22.83618736 | 22.87850571 | 22.89520836 |
|  | 0.120871186 | -0.019278208 | Q68FL6;E9QB02;F6W0G8 | Methionine--tRNA ligase, cytoplasmic | Mars | 23.55987549 | 23.43517113 | 23.43033028 | 23.50954056 | 23.55461693 | 23.41905403 |
|  | 0.189508563 | -0.018709183 | P50247;A2ALT5 | Adenosylhomocysteinase | Ahcy | 24.61380386 | 24.65212059 | 24.66795731 | 24.71988869 | 24.60178566 | 24.66833496 |
|  | 0.079865629 | -0.017528534 | B7ZCU2;B7ZCU5;B7ZCU4;B7ZCU3;B7ZCU0;J3QNK8;Q8CBW3;A0A087WNT3;A0A087WP64;A0A087WPE6;A0A087WNU9 | Abl interactor 1 | Abi1 | 24.1914444 | 24.26076317 | 24.31007957 | 24.13288879 | 24.34251976 | 24.33946419 |
|  | 0.087818458 | -0.017328262 | P31938 | Dual specificity mitogen-activated protein kinase kinase 1 | Map2k1 | 24.36786461 | 24.42751884 | 24.22780609 | 24.33265305 | 24.31015015 | 24.43237114 |
|  | 0.074178448 | -0.017301559 | E9Q137 | Testis-expressed protein 264 homolog | Tex264 | 24.19610405 | 23.97289848 | 24.10149384 | 24.16346169 | 24.00727272 | 24.15166664 |
|  | 0.225928716 | -0.017251333 | Q9CWJ9 | Bifunctional purine biosynthesis protein PURH;Phosphoribosylaminoimidazolecarboxamide formyltransferase;IMP cyclohydrolase | Atic | 25.05767441 | 25.07520676 | 25.14612007 | 25.08711624 | 25.11286926 | 25.13076973 |
|  | 0.355444019 | -0.017199834 | Q9WVA4;A0A0A6YXG6;Q9R1Q8 | Transgelin-2 | Tagln2 | 27.59405899 | 27.64883423 | 27.59697533 | 27.62971687 | 27.64718819 | 27.61456299 |
|  | 0.13623376 | -0.01686732 | P62137 | Serine/threonine-protein phosphatase PP1-alpha catalytic subunit | Ppp1ca | 25.1529808 | 25.0977211 | 25.08638573 | 25.11108017 | 25.20720863 | 25.06940079 |
|  | 0.154821073 | -0.016614278 | Q9JKW0;A0A0U1RPY6 | ADP-ribosylation factor-like protein 6-interacting protein 1 | Arl6ip1 | 24.45588303 | 24.47667122 | 24.48591232 | 24.52522087 | 24.41125107 | 24.53183746 |
|  | 0.112509448 | -0.01655515 | F8VQ28;A0A1D5RMM8;Q8VI36;A0A0J9YV30 | Paxillin | Pxn | 23.13048744 | 23.1203804 | 23.1987896 | 23.09266663 | 23.25465012 | 23.15200615 |
|  | 0.042204216 | -0.015983582 | Q80XI4 | Phosphatidylinositol 5-phosphate 4-kinase type-2 beta | Pip4k2b | 22.05864143 | 22.34247971 | 22.44189835 | 22.40673065 | 22.20520401 | 22.27903557 |
|  | 0.140106559 | -0.015195847 | P31786;Q4VWZ5;M0QWU8;D3Z563 | Acyl-CoA-binding protein | Dbi | 27.43737984 | 27.42278099 | 27.490448 | 27.40381241 | 27.52305412 | 27.46932983 |
|  | 0.056780796 | -0.01489385 | A0A494BA97;Q62422 | Osteoclast-stimulating factor 1 | Ostf1 | 26.77127647 | 26.94737434 | 26.99410057 | 26.80045319 | 26.96201515 | 26.9949646 |
|  | 0.068395489 | -0.014769236 | P70460 | Vasodilator-stimulated phosphoprotein | Vasp | 24.09466553 | 23.92152405 | 23.98676491 | 23.91788864 | 24.01741791 | 24.11195564 |
|  | 0.144693382 | -0.014628092 | H3BKH6;Q9R0P3;H3BLJ9;H3BJL6;H3BJP2;H3BK43;H3BJC6;H3BL99 | S-formylglutathione hydrolase | Esd | 28.00474548 | 27.95637512 | 27.98777199 | 27.93702507 | 28.05744743 | 27.99830437 |
|  | 0.062687272 | -0.01368014 | Q6ZQI3 | Malectin | Mlec | 24.13500404 | 23.93984032 | 23.98259163 | 24.1272316 | 23.99178886 | 23.97945595 |
|  | 0.109183886 | -0.013669332 | Q8BG32;G3UYH2;G3UYI4;G3UYL3;G3UYL8;G3UX15;G3UWW7;G3UXL5;G3UX67;G3UWV7;G3UZ28;G3UZ33 | 26S proteasome non-ATPase regulatory subunit 11 | Psmd11 | 24.40047836 | 24.36151886 | 24.31153679 | 24.36652946 | 24.30966377 | 24.43834877 |
|  | 0.04163577 | -0.013604482 | Q3UZG4;P31230;A0A0G2JDW6;A0A0G2JEU9;A0A0G2JDH0 | Aminoacyl tRNA synthase complex-interacting multifunctional protein 1;Endothelial monocyte-activating polypeptide 2 | Aimp1 | 24.10245514 | 24.16292381 | 24.32058525 | 24.16476631 | 24.38264847 | 24.07936287 |
|  | 0.185663742 | -0.013471603 | Q7M6Y3;A0A1L1SUR7;A0A140LHG9 | Phosphatidylinositol-binding clathrin assembly protein | Picalm | 24.68474579 | 24.64784622 | 24.59286118 | 24.66833496 | 24.64278793 | 24.6547451 |
|  | 0.138586477 | -0.013461431 | Q8VCT3;E9PYF1 | Aminopeptidase B | Rnpep | 25.19087791 | 25.09373856 | 25.09752083 | 25.17371368 | 25.12994576 | 25.11886215 |
|  | 0.046021834 | -0.013417562 | Q3U1Z5 | G-protein-signaling modulator 3 | Gpsm3 | 23.92840958 | 23.95109177 | 24.03892136 | 23.99351692 | 24.14499283 | 23.82016563 |
|  | 0.091638987 | -0.01339976 | Q8BH43;B1AUN0 | Wiskott-Aldrich syndrome protein family member 2 | Wasf2 | 24.31423569 | 24.31554985 | 24.32663155 | 24.4086628 | 24.35494423 | 24.23300934 |
|  | 0.07062382 | -0.013228734 | O70252;D3YX62;D3YXN4;D3Z4A2 | Heme oxygenase 2 | Hmox2 | 24.24670029 | 24.27632523 | 24.12691689 | 24.24670029 | 24.30159378 | 24.14133453 |
|  | 0.133059633 | -0.012940725 | P47962;D3YYV8 | 60S ribosomal protein L5 | Rpl5 | 25.69394302 | 25.78526497 | 25.71777344 | 25.71884346 | 25.79133987 | 25.72562027 |
|  | 0.071315745 | -0.0126489 | A0A494BBA8;Q642K5;A0A494B9Z0;P62862;P35545 | 40S ribosomal protein S30 | Fau | 25.90193939 | 25.91412926 | 25.94272804 | 25.96007156 | 26.02106667 | 25.81560516 |
|  | 0.141875748 | -0.012305578 | P35564 | Calnexin | Canx | 26.10645485 | 26.1543541 | 26.11392403 | 26.1934948 | 26.10133362 | 26.11682129 |
|  | 0.076309227 | -0.011634827 | H9H9R4;Q9JHJ3;H3BK59;H3BKY1 | Glycosylated lysosomal membrane protein | Glmp | 23.42611313 | 23.56639481 | 23.50203133 | 23.51014519 | 23.44884491 | 23.57045364 |
|  | 0.083907278 | -0.011421839 | Q9R1P4;A0A1B0GS70 | Proteasome subunit alpha type-1 | Psma1 | 25.55036545 | 25.53516579 | 25.57071304 | 25.54654312 | 25.49176407 | 25.65220261 |
|  | 0.038630444 | -0.011267344 | P11404 | Fatty acid-binding protein, heart | Fabp3 | 23.66427231 | 23.44796181 | 23.78236771 | 23.67238808 | 23.62778473 | 23.62823105 |
|  | 0.224868431 | -0.011249542 | P68040 | Guanine nucleotide-binding protein subunit beta-2-like 1;Guanine nucleotide-binding protein subunit beta-2-like 1, N-terminally processed | Gnb2l1 | 26.28963089 | 26.35627174 | 26.32555008 | 26.33960152 | 26.32846451 | 26.33713531 |
|  | 0.037237247 | -0.010924657 | Q6GQT9 | Nodal modulator 1 | Nomo1 | 23.15941811 | 23.15550041 | 23.14356232 | 23.31644821 | 22.97729111 | 23.19751549 |
|  | 0.218009463 | -0.010864258 | P80315;G5E839;G3UYW5;G3UXF3;G3UXG2 | T-complex protein 1 subunit delta | Cct4 | 25.47082138 | 25.41786385 | 25.44729996 | 25.45161438 | 25.47871017 | 25.4382534 |
|  | 0.153367499 | -0.010447184 | P62827;Q14AA6;Q61820 | GTP-binding nuclear protein Ran | Ran;1700009N14Rik | 26.67626762 | 26.66315842 | 26.74445152 | 26.71214104 | 26.70067978 | 26.7023983 |
|  | 0.031418862 | -0.010199865 | P47811;A0A3B2WB60;B2KF34 | Mitogen-activated protein kinase 14 | Mapk14 | 23.54686737 | 23.524086 | 23.22180176 | 23.41673851 | 23.50373077 | 23.40288544 |
|  | 0.075298037 | -0.0100015 | Q9R112;H3BLH2;F6ZKZ3 | Sulfide:quinone oxidoreductase, mitochondrial | Sqrdl | 24.15112495 | 24.2487278 | 24.08990479 | 24.16553307 | 24.18578529 | 24.16844368 |
|  | 0.078940623 | -0.00983429 | F8VQC1;E9Q740 | Signal recognition particle subunit SRP72 | Srp72 | 24.27483368 | 24.27362633 | 24.23936081 | 24.35709572 | 24.22758484 | 24.23264313 |
|  | 0.044255192 | -0.009793599 | P51125;A0A338P6J9;Q8CE80;Q921U7;Q8C281;Q8CE04;A0A1Y7VJN8 | Calpastatin | Cast | 24.24350739 | 24.0839138 | 24.07226563 | 24.05440712 | 24.23293686 | 24.14172363 |
|  | 0.021829779 | -0.009419123 | Q62376;A0A1B0GRR8;A0A1B0GR69;A0A1B0GR44;A0A1B0GRV8 | U1 small nuclear ribonucleoprotein 70 kDa | Snrnp70 | 24.11441994 | 24.00940895 | 23.83951759 | 23.85128975 | 23.90591431 | 24.2343998 |
|  | 0.02697368 | -0.009283066 | Q9CX86 | Heterogeneous nuclear ribonucleoprotein A0 | Hnrnpa0 | 23.31934547 | 23.31299019 | 23.20858574 | 23.42123985 | 23.07169342 | 23.37583733 |
|  | 0.036404546 | -0.009159088 | A2AJI1;A8Y5P4;A2AJI0;F6WF36 | MAP7 domain-containing protein 1 | Map7d1 | 22.99470901 | 23.24341965 | 22.98514938 | 23.08534241 | 23.06409645 | 23.10131645 |
|  | 0.048202816 | -0.009065628 | Q9CQV8;A2A5N1 | 14-3-3 protein beta/alpha;14-3-3 protein beta/alpha, N-terminally processed | Ywhab | 26.85816193 | 26.84122086 | 26.90586853 | 26.84182167 | 26.99722672 | 26.79339981 |
|  | 0.058432104 | -0.008060455 | P80314;A0A1W2P7B7;A0A1W2P828;A0A1W2P6Q3;A0A1W2P8B6;A0A1W2P871 | T-complex protein 1 subunit beta | Cct2 | 27.90155983 | 27.89463806 | 27.78075409 | 27.90500832 | 27.81408882 | 27.88203621 |
|  | 0.051011747 | -0.008017222 | Q8K124 | Pleckstrin homology domain-containing family O member 2 | Plekho2 | 25.05142212 | 25.08606339 | 24.96939087 | 24.96186066 | 25.09655571 | 25.07251167 |
|  | 0.020309719 | -0.007971446 | P61202;A2AQE4 | COP9 signalosome complex subunit 2 | Cops2 | 22.86774445 | 23.10905647 | 22.91836166 | 22.96040344 | 23.16636086 | 22.79231262 |
|  | 0.036634061 | -0.00777626 | P61961;H7BWZ1;D3YW97 | Ubiquitin-fold modifier 1 | Ufm1 | 25.26481247 | 25.21660042 | 25.21100235 | 25.30445099 | 25.09892654 | 25.31236649 |
|  | 0.026619174 | -0.007680893 | Q9DC23 | DnaJ homolog subfamily C member 10 | Dnajc10 | 22.40807915 | 22.24010468 | 22.12587547 | 22.27696419 | 22.1714859 | 22.34865189 |
|  | 0.020893086 | -0.007338206 | Q3UND0;A0A0N4SVW8 | Src kinase-associated phosphoprotein 2 | Skap2 | 23.76951218 | 23.7488842 | 23.74693871 | 23.75266647 | 23.96956635 | 23.56511688 |
|  | 0.037589029 | -0.007099152 | B1AQF4;Q9D7X3;Q3V2Y9;H3BKL8;H3BKD1 | Dual specificity protein phosphatase 3 | Dusp3 | 25.3363018 | 25.28947258 | 25.4573555 | 25.32961082 | 25.44815063 | 25.32666588 |
|  | 0.086017965 | -0.007046382 | P52480;A0A1L1SU37;A0A1L1SQV8;A0A1L1SSN6;A0A1L1STV8;A0A1L1SUV0;A0A1L1ST52;A0A1L1SVH2;E9Q509;G3X925;P53657 | Pyruvate kinase PKM | Pkm | 30.01916313 | 30.05465508 | 30.03891563 | 30.09358215 | 29.99967575 | 30.04061508 |
|  | 0.058947957 | -0.006746292 | P70315 | Wiskott-Aldrich syndrome protein homolog | Was | 24.48492813 | 24.47382736 | 24.51189423 | 24.5120163 | 24.55391312 | 24.42495918 |
|  | 0.048514324 | -0.006514231 | Q9D883;G3UW94;A0A494BA95;A0A494B9X9;E9Q809;A0A494B947;F8WH71;E9Q5J3;E9PWX6;E9PWM2 | Splicing factor U2AF 35 kDa subunit | U2af1 | 23.56697464 | 23.52229118 | 23.5532093 | 23.54427528 | 23.48296165 | 23.63478088 |
|  | 0.059451918 | -0.0054334 | Q91V41;Q50HX3;A0A2R8VHW9 | Ras-related protein Rab-14 | Rab14 | 26.64306259 | 26.57542419 | 26.63519478 | 26.5997448 | 26.67006683 | 26.60017014 |
|  | 0.012606584 | -0.004631678 | Q3MIA8;G3UXW9;Q99LD4;A0A140LJB7;B1ATU4 | COP9 signalosome complex subunit 1 | Gps1 | 23.52145195 | 23.36332512 | 23.13583374 | 23.43834877 | 23.30605125 | 23.29010582 |
|  | 0.012178872 | -0.00445048 | Q8BI72 | CDKN2A-interacting protein | Cdkn2aip | 20.86143494 | 20.61243248 | 20.88293266 | 20.840765 | 20.73933601 | NaN |
|  | 0.031820386 | -0.004350026 | Q8BGD9;B2RWE8 | Eukaryotic translation initiation factor 4B | Eif4b | 24.69317245 | 24.59940338 | 24.61666679 | 24.71229935 | 24.61172485 | 24.59826851 |
|  | 0.052384963 | -0.004074732 | Q62465 | Synaptic vesicle membrane protein VAT-1 homolog | Vat1 | 27.47405815 | 27.49205399 | 27.41264153 | 27.48631096 | 27.45787239 | 27.44679451 |
|  | 0.039726793 | -0.003602982 | P07356;B0V2N5;B0V2N7;B0V2N8 | Annexin A2;Annexin | Anxa2 | 28.2312336 | 28.14146042 | 28.14160728 | 28.18909836 | 28.17250252 | 28.16350937 |
|  | 0.03388825 | -0.003116608 | P06800;S4R1M0;A0A0A6YXM4 | Receptor-type tyrosine-protein phosphatase C;Protein-tyrosine-phosphatase | Ptprc | 25.28467941 | 25.28838158 | 25.28859329 | 25.26345253 | 25.35225296 | 25.25529861 |
|  | 0.031304899 | -0.002861659 | Q07076;A0A2C9F2D2;A0A286YCW4 | Annexin A7 | Anxa7 | 25.83472824 | 25.8595829 | 25.89693642 | 25.9153595 | 25.83306313 | 25.85140991 |
|  | 0.005974402 | -0.00252978 | P30999;E9Q986;E9Q8Z6;E9Q8Z5;G3X9V2;E9Q904;E9Q901;E9Q903;D3Z2H2;E9Q8Z9;E9Q8Z4;E9Q905;E9Q907;E9Q906;D3Z7H6;E9Q8Z8;D3Z2H7 | Catenin delta-1 | Ctnnd1 | 22.3288784 | 22.5987339 | 22.60598373 | 22.72048378 | 22.43941307 | 22.38128853 |
|  | 0.03095954 | -0.002068837 | Q9WUM4;E9PX03;E9PZJ0;E9PVJ1;B9EIZ7;Q920M5 | Coronin-1C | Coro1c | 26.54166412 | 26.57029343 | 26.55265427 | 26.5391655 | 26.59860992 | 26.53304291 |
|  | 0.011420434 | -0.001784007 | O08997 | Copper transport protein ATOX1 | Atox1 | 27.03422737 | 27.02379799 | 27.08790398 | 27.04697609 | 27.13447571 | 26.96982956 |
|  | 0.004015127 | -0.00169309 | Q91YI4;Q5F2D9 | Beta-arrestin-2 | Arrb2 | 23.01156044 | 22.74057198 | 23.15295029 | 22.98615646 | 22.84794998 | 23.07605553 |
|  | 0.005549739 | -0.001357396 | P99024;A0A1D5RM76 | Tubulin beta-5 chain | Tubb5 | 28.53133202 | 28.63270187 | 28.50335884 | 28.61076736 | 28.41856384 | 28.64213371 |
|  | 0.002403576 | -0.00097084 | Q9D1M0 | Protein SEC13 homolog | Sec13 | 24.69683838 | 24.88054276 | 24.92356682 | 24.65283203 | 25.03921509 | 24.81181335 |
|  | 0.002739117 | -0.000785192 | Q3TF41;Q8BSH9;P28656 | Nucleosome assembly protein 1-like 1 | Nap1l1 | 25.40791702 | 25.20037651 | 25.25273895 | 25.30799866 | 25.15784645 | 25.39754295 |
|  | 0.013547625 | -0.000776927 | Q8CIE6;F8WHL2;F6XJN3 | Coatomer subunit alpha;Xenin;Proxenin;Coatomer subunit alpha | Copa | 24.46438408 | 24.45976448 | 24.41021729 | 24.44726753 | 24.45795059 | 24.4314785 |
|  | 0.002695696 | -0.000747363 | Q9DCF9 | Translocon-associated protein subunit gamma | Ssr3 | NaN | 24.30061531 | 24.21118927 | 24.31782722 | 24.32292366 | 24.12919807 |
|  | 0.00255587 | -0.000598907 | Q6R891 | Neurabin-2 | Ppp1r9b | 23.27980042 | 23.13007927 | 23.38139343 | 23.2230835 | 23.26713753 | 23.30284882 |
|  | 0.002523226 | -0.000407537 | Q5SW88;Q5SW87;Q5SW86 | RAB1A, member RAS oncogene family | Rab1 | 25.13727379 | 24.98997116 | 25.02478981 | 25.09462357 | 24.99765778 | 25.06097603 |
|  | 0.002657359 | -0.000348409 | Q6P5E4;G3UYG7;G3UY73;G3UZU8;G3UY35;G3UXP5;E9Q4X2 | UDP-glucose:glycoprotein glucosyltransferase 1 | Uggt1 | 24.67443657 | 24.57247925 | 24.70804024 | 24.64251328 | 24.63544464 | 24.67804337 |
|  | 0.000447257 | -0.000139236 | Q91Z50;P39749 | Flap endonuclease 1 | Fen1 | 23.57323074 | 23.51280022 | 23.37597084 | 23.48812103 | 23.34340096 | 23.63089752 |
|  | 0.000442218 | -0.000119527 | P70444;D3Z463 | BH3-interacting domain death agonist;BH3-interacting domain death agonist p15;BH3-interacting domain death agonist p13;BH3-interacting domain death agonist p11 | Bid | 24.04501915 | 23.88901901 | 23.78855515 | 23.81470108 | 23.96393394 | 23.94431686 |
|  | 0.00085879 | -8.2E-05 | Q8JZN2;Q91W50;A0A0G2JF72;A0A0G2JDJ7;A0A0G2JE62 | Cold shock domain-containing protein E1 | Csde1 | 23.00937462 | 23.06677437 | 23.068367 | 23.04919624 | 23.08995438 | 23.00561142 |
|  | 1.87E-05 | -3.18E-06 | Q8VC28;D6RGB1;G3X9Y6 | Aldo-keto reductase family 1 member C13 | Akr1c13 | 24.11933708 | 24.132967 | 24.24604797 | 24.17180634 | 24.22883415 | 24.0977211 |
|  | 0.000207825 | 0.000171661 | A0A1D5RMC1;Q6PDL0;A0A1D5RM94 | Cytoplasmic dynein 1 light intermediate chain 2 | Dync1li2 | 23.53848457 | 23.07724571 | 23.11526108 | 22.85435677 | 23.63333893 | 23.24278069 |
|  | 0.014929894 | 0.001517614 | Q9DB05;A0A1B0GR35;P28663 | Alpha-soluble NSF attachment protein | Napa | 24.38357162 | 24.33169746 | 24.37218857 | 24.338377 | 24.4199543 | 24.32457352 |
|  | 0.003106971 | 0.001628876 | Q9DBR7;A0A1W2P750 | Protein phosphatase 1 regulatory subunit 12A | Ppp1r12a | 21.65337944 | 21.79129982 | 22.0249939 | 21.58279228 | 22.04122925 | 21.840765 |
|  | 0.012046445 | 0.001710892 | O09061;A0A338P7C0;A0A338P7F1 | Proteasome subunit beta type-1 | Psmb1 | 25.7055912 | 25.72715378 | 25.73858833 | 25.80579948 | 25.71271896 | 25.64768219 |
|  | 0.014215692 | 0.001794815 | Q3UPL0;S4R2A9;S4R192;S4R256;S4R1T5;S4R1Y8 | Protein transport protein Sec31A | Sec31a | 23.88464737 | 23.88427544 | 23.79520988 | 23.85405159 | 23.80153847 | 23.90315819 |
|  | 0.011120417 | 0.001925151 | Q8BT60;V9GX86;B7ZCP8;V9GWY2;Q9D6C8;A0A0R4J0J1;Q3UYN2;Q1RLL3;Q9Z140;Q8BLR2;Q0VE82;Q9DC53;Q8JZW4 | Copine-3 | Cpne3 | 23.98346329 | 23.91670418 | 23.96208191 | 24.05928421 | 23.89513588 | 23.90205383 |
|  | 0.012294934 | 0.002225876 | Q61792;A2A6H0;A2A6G9;A2A6G7;A2A6G8;A2A6G6;A2A6H1;A2A6G5;E9Q0N6;A2A6G0;A2A6G4;Q9DC07 | LIM and SH3 domain protein 1 | Lasp1 | 26.68659019 | 26.8206768 | 26.75366211 | 26.67020226 | 26.82784081 | 26.75620842 |
|  | 0.032232664 | 0.002418518 | Q78PY7;Q3TJ56;E9Q3E9 | Staphylococcal nuclease domain-containing protein 1 | Snd1 | 25.07414627 | 25.04772568 | 25.00594521 | 25.00915337 | 25.05158806 | 25.05982018 |
|  | 0.017931319 | 0.002500534 | P97363 | Serine palmitoyltransferase 2 | Sptlc2 | 23.29796028 | 23.29193306 | 23.20028687 | 23.2521534 | 23.20705223 | 23.32347298 |
|  | 0.035978825 | 0.002697627 | Q63844;D3Z3G6;A0A0U1RPX4;D3Z6D8;A0A0U1RPZ0;A0A0E2WHU5 | Mitogen-activated protein kinase 3;Mitogen-activated protein kinase | Mapk3 | 25.2228775 | 25.24150848 | 25.1964798 | 25.258358 | 25.2104454 | 25.1839695 |
|  | 0.019494434 | 0.002773285 | Q64442 | Sorbitol dehydrogenase | Sord | 23.40210533 | 23.55800819 | 23.44101143 | 23.46026421 | 23.47766113 | 23.45487976 |
|  | 0.025555276 | 0.003079732 | Q6A0A9 | Constitutive coactivator of PPAR-gamma-like protein 1 | FAM120A | 23.88966942 | 23.81431007 | 23.77344131 | 23.83200073 | 23.85481262 | 23.78136826 |
|  | 0.005952639 | 0.003106753 | A0A0R4J1L2;Q91VK2;F6ZFU0;D3YY68;D3Z7N2 | Eukaryotic translation elongation factor 1 delta (guanine nucleotide exchange protein) | Eef1d | 25.08646774 | 25.41008759 | 25.13010216 | 25.06500816 | 25.34654999 | NaN |
|  | 0.007601756 | 0.003468831 | P47199;D3YUG9;V9GXY8;A0A0A6YXR4;D3YWU6;D3Z4Q4;D3Z2X0 | Quinone oxidoreductase | Cryz | 22.94579887 | 22.58993912 | 22.70491028 | 22.66670036 | 22.82012749 | NaN |
|  | 0.004292851 | 0.003599485 | Q99K85;Q3U6K9;E9Q6P1 | Phosphoserine aminotransferase | Psat1 | NaN | 22.78470612 | 22.24368095 | 22.67962265 | 22.61488342 | 22.23727608 |
|  | 0.010516991 | 0.003833135 | Q91XU3 | Phosphatidylinositol 5-phosphate 4-kinase type-2 gamma | Pip4k2c | 23.30173302 | 23.12366867 | 23.5114727 | 23.38508606 | 23.23534966 | 23.30493927 |
|  | 0.008603184 | 0.004341761 | Q8R574 | Phosphoribosyl pyrophosphate synthase-associated protein 2 | Prpsap2 | 22.52169228 | 22.52446747 | 22.6254673 | 22.87192345 | 22.44639778 | 22.34028053 |
|  | 0.07528552 | 0.004402161 | P80313;A0A0N4SV00;A0A0N4SUI8;A0A0N4SV22 | T-complex protein 1 subunit eta | Cct7 | 25.19076538 | 25.20672417 | 25.20571899 | 25.17831802 | 25.1752758 | 25.23640823 |
|  | 0.06067501 | 0.004613241 | P28867;Q1MX43;Q1MX42;Q1MX41;Q1MX40;REV__E9Q9T6;REV__Q6ZPL9;F8WGT6;Q02111 | Protein kinase C delta type;Protein kinase C delta type regulatory subunit;Protein kinase C delta type catalytic subunit;Protein kinase C | Prkcd | 24.0674324 | 24.05332947 | 24.02664948 | 24.09111786 | 24.02656555 | 24.01588821 |
|  | 0.012373878 | 0.004728317 | Q9CQQ7;A0A0G2JGX3 | ATP synthase F(0) complex subunit B1, mitochondrial | Atp5f1 | 25.48376083 | 25.68179893 | 25.52545929 | 25.51992416 | 25.76751709 | 25.38939285 |
|  | 0.053515725 | 0.004909515 | Q8BMJ2;A0A494BA98;A0A494BBF9;A0A494B919;A0A494BA30 | Leucine--tRNA ligase, cytoplasmic | Lars | 23.43529892 | 23.41403008 | 23.33851242 | 23.40015221 | 23.36786461 | 23.40509605 |
|  | 0.016986074 | 0.005146662 | Q9QWR8;A0A2R8VHH9;Q3UZX5;A0A2R8VHJ3;A0A2R8W742 | Alpha-N-acetylgalactosaminidase | Naga | 23.87464523 | 23.64306259 | 23.72254944 | 23.87698936 | 23.62142754 | 23.72640038 |
|  | 0.024816978 | 0.005270004 | Q8BJU0;A0A1W2P6P1;A0A1W2P7I5 | Small glutamine-rich tetratricopeptide repeat-containing protein alpha | Sgta | 23.40392685 | 23.32704353 | 23.29557991 | 23.46338654 | 23.26627922 | 23.28107452 |
|  | 0.026490197 | 0.006717046 | E9Q0U7;Q61699;D3Z3I9;A0A0J9YTZ7;D3Z027 | Heat shock protein 105 kDa | Hsph1 | 22.74088097 | 22.5532093 | 22.60995674 | 22.67848396 | 22.7052269 | 22.50018501 |
|  | 0.061004018 | 0.007387797 | P06151;A0A1B0GSX0;A0A1B0GSR9;D3YZQ9;A0A1B0GT41;A0A1B0GQX5;A0A1B0GSL7;A0A1B0GRW9;A0A1B0GS79;A0A1B0GRC1;A0A1B0GRS2;D3YVR7;D3YZE4;P00342;A0A1B0GSR2;A0A1B0GRE9 | L-lactate dehydrogenase A chain;L-lactate dehydrogenase | Ldha | 28.4202919 | 28.34138298 | 28.32349968 | 28.33615685 | 28.31449509 | 28.41235924 |
|  | 0.012914658 | 0.007767042 | Q80XN0;D3Z2Y8 | D-beta-hydroxybutyrate dehydrogenase, mitochondrial | Bdh1 | 22.75932693 | 22.99938011 | NaN | 23.02367401 | 22.60435677 | 22.98672867 |
|  | 0.064256475 | 0.008577347 | Q8VDM4;A0A338P6M5;E9Q2S8;A0A338P7H4;A0A338P6S9 | 26S proteasome non-ATPase regulatory subunit 2 | Psmd2 | 25.45192719 | 25.49445152 | 25.37388229 | 25.45519257 | 25.46774673 | 25.37158966 |
|  | 0.038986738 | 0.008845011 | P62245;F8WJ41;D3YVB4;D3Z712 | 40S ribosomal protein S15a | Rps15a | 25.95617676 | 25.88915825 | 26.08903694 | 25.96287537 | 26.0586853 | 25.88627625 |
|  | 0.033976959 | 0.008886337 | Q8BYK6 | YTH domain-containing family protein 3 | Ythdf3 | 22.30114746 | 22.50867081 | 22.32674026 | NaN | 22.39636803 | 22.34356499 |
|  | 0.053614321 | 0.009202957 | P17918 | Proliferating cell nuclear antigen | Pcna | 23.97823334 | 24.14156723 | 24.12911987 | 24.09393883 | 24.01988029 | 24.10749245 |
|  | 0.036010745 | 0.009618123 | P58389;A2AWF0;A2AWE9;B7ZDE0;D6RFC2;F6Z6I0 | Serine/threonine-protein phosphatase 2A activator | Ppp2r4 | 23.85309982 | 23.81597137 | 24.00701523 | 23.74960136 | 23.91643143 | 23.98119926 |
|  | 0.012015865 | 0.009845416 | P10810 | Monocyte differentiation antigen CD14 | Cd14 | 22.3565712 | NaN | 22.84251213 | 22.28990936 | 22.74954033 | 22.72963905 |
|  | 0.055874638 | 0.009918213 | G5E850;P56395;A0A494B9D8;E0CY88 | Cytochrome b5 | Cyb5a | 26.49954033 | 26.56181335 | 26.53051376 | 26.552742 | 26.60229492 | 26.40707588 |
|  | 0.025527878 | 0.009954453 | P11688 | Integrin alpha-5;Integrin alpha-5 heavy chain;Integrin alpha-5 light chain | Itga5 | 22.95888138 | 22.95095062 | 23.31547928 | 23.04456902 | 22.98872375 | 23.16215515 |
|  | 0.194722755 | 0.010032654 | Q61598;A0A1Y7VL99;A0A1Y7VLG4 | Rab GDP dissociation inhibitor beta | Gdi2 | 26.96708298 | 26.96344948 | 26.98945236 | 26.95238113 | 26.99851799 | 26.93898773 |
|  | 0.047417709 | 0.010126114 | P62192 | 26S protease regulatory subunit 4 | Psmc1 | 24.73407173 | 24.89295959 | 24.73768616 | 24.6768055 | 24.81782532 | 24.83970833 |
|  | 0.025706312 | 0.010203362 | O55126;Q7TMG8;A0A0G2JEV1 | Protein NipSnap homolog 2 | Gbas | 23.03902245 | 22.97026825 | 23.28220749 | 23.14788437 | NaN | 23.02604103 |
|  | 0.019993883 | 0.010351817 | P30204;A0A1B0GRS5 | Macrophage scavenger receptor types I and II | Msr1 | 25.56459236 | 25.63776779 | 25.36816406 | 25.80970573 | 25.42649651 | 25.30326653 |
|  | 0.025168517 | 0.01069514 | Q8CFZ0;G3UYP0;P63280 | SUMO-conjugating enzyme UBC9 | Ube2i | 23.76961327 | 23.79203415 | 23.5741539 | 23.49838448 | 23.92723465 | 23.67809677 |
|  | 0.059688163 | 0.01093928 | Q3U0V1;A0A3B2WCD8;A0A3B2W465 | Far upstream element-binding protein 2 | Khsrp | 24.20702171 | 24.38053513 | 24.28213692 | 24.23914337 | 24.24110794 | 24.3566246 |
|  | 0.116320369 | 0.011112849 | Q8BG05;A2AL12;A2AL13 | Heterogeneous nuclear ribonucleoprotein A3 | Hnrnpa3 | 26.60045433 | 26.64636421 | 26.68431664 | 26.58319473 | 26.66234398 | 26.65225792 |
|  | 0.21179856 | 0.011842728 | Q99KC8;F6TIL5;D3Z671;D3Z518 | von Willebrand factor A domain-containing protein 5A | Vwa5a | 25.58319473 | 25.61371994 | 25.54085159 | 25.57155228 | 25.55765724 | 25.57302856 |
|  | 0.321531139 | 0.01188151 | O70435;E0CX62;E0CZ34;F8WH02;E0CYL6 | Proteasome subunit alpha type-3 | Psma3 | 25.53753662 | 25.57224655 | 25.52599716 | 25.53011322 | 25.54510117 | 25.52492142 |
|  | 0.053095514 | 0.011901855 | P61089;A0A1W2P7Z3 | Ubiquitin-conjugating enzyme E2 N | Ube2n | 25.65436172 | 25.81433296 | 25.65750122 | 25.61790085 | 25.80582428 | 25.66676521 |
|  | 0.079490475 | 0.012083054 | P56380 | Bis(5-nucleosyl)-tetraphosphatase [asymmetrical] | Nudt2 | 22.60900879 | 22.53214836 | 22.61348915 | 22.52205086 | 22.53033829 | 22.666008 |
|  | 0.126092111 | 0.012587229 | P12815 | Programmed cell death protein 6 | Pdcd6 | 23.59194946 | 23.55086327 | 23.568367 | 23.57218933 | 23.60918999 | 23.49203873 |
|  | 0.106566708 | 0.012594223 | P97369;A8XU21 | Neutrophil cytosol factor 4 | Ncf4 | 24.51773262 | 24.43574333 | 24.50633621 | 24.42091942 | 24.53753662 | 24.46357346 |
|  | 0.054306859 | 0.013165792 | Q8BH40;O70439 | Syntaxin-7 | Stx7 | 24.81528664 | 24.89661407 | 24.85105133 | 24.93494606 | 24.90692329 | 24.68158531 |
|  | 0.053695218 | 0.013185501 | P61226 | Ras-related protein Rap-2b | Rap2b | 25.56118774 | 25.35080338 | 25.49670792 | 25.43860245 | 25.36719704 | 25.56334305 |
|  | 0.075119129 | 0.013231913 | G3UYV7;P62858 | 40S ribosomal protein S28 | Rps28 | 25.7961998 | 25.77198219 | 25.95763779 | 25.81755829 | 25.79946518 | 25.86910057 |
|  | 0.152050606 | 0.013638814 | Q8R050;Q149F3;F7CE88 | Eukaryotic peptide chain release factor GTP-binding subunit ERF3A | Gspt1 | 24.02098274 | 24.12218285 | 24.11616707 | 24.08610344 | 24.06890869 | 24.06340408 |
|  | 0.103368421 | 0.014253616 | E9Q586;E9Q3M3;O08788;D3YX34;D3Z2M9;D3YYG9 | Dynactin subunit 1 | Dctn1 | 23.96243477 | 24.03498268 | 23.93238068 | 24.02259064 | 23.97561264 | 23.888834 |
|  | 0.162947409 | 0.01428922 | Q810B6 | Rabankyrin-5 | Ankfy1 | 23.52695274 | 23.57945824 | 23.53278923 | 23.57195854 | 23.47691917 | 23.54745483 |
|  | 0.123204683 | 0.014962514 | Q91ZJ5 | UTP--glucose-1-phosphate uridylyltransferase | Ugp2 | 24.37841988 | 24.40704346 | 24.49460411 | 24.35702705 | 24.4373951 | 24.44075775 |
|  | 0.16304203 | 0.014982859 | P18760;F8WGL3;A0A494B9A7 | Cofilin-1 | Cfl1 | 29.17923546 | 29.29412651 | 29.24422455 | 29.20759964 | 29.2401123 | 29.22492599 |
|  | 0.127319261 | 0.01499176 | P51881 | ADP/ATP translocase 2;ADP/ATP translocase 2, N-terminally processed | Slc25a5 | 28.35783386 | 28.3035202 | 28.44750404 | 28.35980225 | 28.33517647 | 28.36890411 |
|  | 0.222666014 | 0.015247345 | Q3U2G2;Q61316;A0A0N4SVU2 | Heat shock 70 kDa protein 4 | Hspa4 | 25.52037239 | 25.56949806 | 25.5389576 | 25.48299217 | 25.54465866 | 25.55543518 |
|  | 0.171874255 | 0.015371958 | P40142;A0A286YE28;E0CY51 | Transketolase | Tkt | 27.8595829 | 27.90506554 | 27.93994141 | 27.85632133 | 27.93488884 | 27.86726379 |
|  | 0.024869704 | 0.015496572 | F6RJ39;Q9JIX8;Q52KR6;B8JJ92;B8JJ91;B8JJ90;F6Q8C0;B8JJ89 | Apoptotic chromatin condensation inducer in the nucleus | Acin1 | 21.95255089 | 22.1646595 | 22.00710106 | 22.39707375 | 21.71753693 | 21.96321106 |
|  | 0.218687992 | 0.016101837 | Q76MZ3;G3UWL2;G3UXQ1;H3BK50;H3BJ83 | Serine/threonine-protein phosphatase 2A 65 kDa regulatory subunit A alpha isoform | Ppp2r1a | 26.13848495 | 26.17672157 | 26.11042213 | 26.09184265 | 26.1204834 | 26.1649971 |
|  | 0.118580984 | 0.016427358 | E9Q9E8;A0A0R4J0K5;Q18PI6;A0A0R4J1S4 | SLAM family member 5 | Cd84 | 24.2639904 | 24.18555832 | 24.15560913 | 24.26054955 | 24.13163376 | 24.16369247 |
|  | 0.037261483 | 0.016439438 | P61924 | Coatomer subunit zeta-1 | Copz1 | 24.77147865 | 24.68651009 | 24.93264961 | 24.94113922 | 24.52085304 | 24.87932777 |
|  | 0.048175308 | 0.016457876 | Q9CQR2 | 40S ribosomal protein S21 | Rps21 | 25.66993141 | 25.66765976 | 25.78486633 | 25.77140236 | 25.82912445 | 25.47255707 |
|  | 0.072111177 | 0.016472499 | Q9R1P1 | Proteasome subunit beta type-3 | Psmb3 | 24.7961998 | 24.90025902 | 24.70772552 | 24.69189644 | 24.7727375 | 24.8901329 |
|  | 0.195179233 | 0.016533534 | Q80X50;A0A0H2UH17;A0A0G2JDV6;A0A0G2JG47;A0A0G2JGD0;A0A0G2JDT1;A0A0G2JFN7;A0A0G2JE24;A0A0G2JEC6 | Ubiquitin-associated protein 2-like | Ubap2l | 23.89217377 | 23.8700428 | 23.89476585 | 23.8475666 | 23.9312973 | 23.82851791 |
|  | 0.043170741 | 0.017230034 | Q9CQ48;E0CYQ2 | NudC domain-containing protein 2 | Nudcd2 | NaN | 22.90052605 | 22.88742065 | 22.83882523 | 23.07146454 | 22.71994019 |
|  | 0.229206514 | 0.017343521 | Q9QUI0;A0A0A6YXF6;Q62159;H3BL56;A0A0G2JEP8;Q9CR99;A0A0A6YWJ1 | Transforming protein RhoA;Rho-related GTP-binding protein RhoC | Rhoa;Rhoc | 26.82529449 | 26.80378151 | 26.82493019 | 26.79004669 | 26.85495567 | 26.75697327 |
|  | 0.019332676 | 0.017704646 | P63168;Q80ZS7 | Dynein light chain 1, cytoplasmic | Dynll1 | 24.01682281 | 24.16721916 | 24.69349289 | 24.570858 | 23.83238792 | 24.421175 |
|  | 0.035691927 | 0.017869949 | Q99KH8;A2AD84;Q99JT2;D3Z359;Q9Z2W1 | Serine/threonine-protein kinase 24;Serine/threonine-protein kinase 24 35 kDa subunit;Serine/threonine-protein kinase 24 12 kDa subunit | Stk24 | 22.81428909 | 22.93077469 | 22.94760132 | 22.69856644 | 22.73297501 | 23.20751381 |
|  | 0.055315306 | 0.018182119 | Q569Z5;F8WHR6;A0A2R8VHK2 | Probable ATP-dependent RNA helicase DDX46 | Ddx46 | 22.22485161 | 22.11012459 | NaN | 22.09532547 | 22.0469017 | 22.30569077 |
|  | 0.276072764 | 0.018590927 | P08228 | Superoxide dismutase [Cu-Zn] | Sod1 | 27.90380096 | 27.85430145 | 27.88361168 | 27.8298378 | 27.9063282 | 27.84977531 |
|  | 0.120043127 | 0.01872762 | O54782;F6TMZ3;F6Z025 | Epididymis-specific alpha-mannosidase | Man2b2 | 24.40736771 | 24.39937019 | 24.39689064 | 24.35709572 | 24.49106026 | 24.2992897 |
|  | 0.49553893 | 0.019170761 | A0A0A0MQM0;P63242;Q8BGY2;J3QPS8 | Eukaryotic translation initiation factor 5A;Eukaryotic translation initiation factor 5A-1;Eukaryotic translation initiation factor 5A-2 | Eif5a;Eif5a2 | 25.82089615 | 25.86071968 | 25.86440849 | 25.81257248 | 25.83038139 | 25.84555817 |
|  | 0.47756277 | 0.019308726 | P47754;D6RCW7;A0A0N4SVM0 | F-actin-capping protein subunit alpha-2 | Capza2 | 26.67209053 | 26.64416504 | 26.64361382 | 26.61386108 | 26.66288757 | 26.62519455 |
|  | 0.350889685 | 0.019358953 | P47911;A0A0J9YU32 | 60S ribosomal protein L6 | Rpl6 | 26.30427742 | 26.24152756 | 26.24249077 | 26.25340652 | 26.22400093 | 26.25281143 |
|  | 0.01685626 | 0.019847234 | Q8R1G5;L7N466 | 5-formyltetrahydrofolate cyclo-ligase | Mthfsl | 23.04733467 | 22.94699478 | 22.57648659 | 23.28714943 | 22.38703346 | NaN |
|  | 0.128119403 | 0.020064036 | P62715;P63330 | Serine/threonine-protein phosphatase 2A catalytic subunit beta isoform;Serine/threonine-protein phosphatase 2A catalytic subunit alpha isoform | Ppp2cb;Ppp2ca | 24.38541603 | 24.55888367 | 24.40431595 | 24.4585762 | 24.40041161 | 24.42943573 |
|  | 0.287574354 | 0.020190557 | Q9CY64;A2ASB1;A2ASB8;A2ASB7 | Biliverdin reductase A | Blvra | 25.49222183 | 25.4461956 | 25.40950394 | 25.4516449 | 25.4355526 | 25.40015221 |
|  | 0.131760749 | 0.02067248 | Q9D0J8 | Parathymosin | Ptms | 25.7824173 | 25.72411156 | 25.67114639 | 25.75587845 | 25.75039291 | 25.60938644 |
|  | 0.249190963 | 0.020730972 | Q60854;F8WIV2;K7E6F1;E9Q108;E9Q0P9;E9Q3Y1;E9Q6X2;E9PYY0;E9PZQ9;E9Q4R2;E9Q5Q5;Q3UWK8 | Serpin B6 | Serpinb6;Serpinb6a | 27.41457748 | 27.33440971 | 27.32680321 | 27.3036499 | 27.35119247 | 27.35875511 |
|  | 0.186596831 | 0.021137873 | Q8K1X4;A0A2R8VHC4 | NCK associated protein 1 like | Nckap1l | 24.36546326 | 24.29389763 | 24.22258186 | 24.25681114 | 24.29873085 | 24.26298714 |
|  | 0.1895903 | 0.021568298 | Q9WV55;A0A3B2W837 | Vesicle-associated membrane protein-associated protein A | Vapa | 24.94641495 | 24.93552971 | 25.06015015 | 24.92456436 | 24.9814167 | 24.97140884 |
|  | 0.115500553 | 0.021589915 | Q61035;A0A494BAV2;A0A494B9A1 | Histidine--tRNA ligase, cytoplasmic | Hars | 23.73272705 | 23.94064713 | 23.82880974 | 23.8692894 | 23.80833054 | 23.75979424 |
|  | 0.169797453 | 0.021978378 | A0A087WP83;Q8VDJ3;A0A087WS92;A0A087WQY9;A0A087WPC5 | Vigilin | Hdlbp | 24.15861702 | 24.15676689 | 24.15776825 | 24.19031334 | 24.17873573 | 24.03816795 |
|  | 0.159411557 | 0.022277196 | Q9QUH0;A0A1Y7VM65 | Glutaredoxin-1 | Glrx | 24.77756119 | 24.70419312 | 24.77213287 | 24.66909218 | 24.69667816 | 24.82128525 |
|  | 0.211575394 | 0.022615433 | P62960;A2BGG7;A0A0A0MQD2;B2RUF0;Q9Z2C8 | Nuclease-sensitive element-binding protein 1 | Ybx1 | 26.27257538 | 26.17516136 | 26.24943352 | 26.17820549 | 26.26819038 | 26.18292809 |
|  | 0.192696688 | 0.022836685 | Q9JLJ2 | 4-trimethylaminobutyraldehyde dehydrogenase | Aldh9a1 | 25.578825 | 25.43895149 | 25.47184372 | 25.47661018 | 25.50121117 | 25.4432888 |
|  | 0.032874483 | 0.023070653 | Q8K4Z3 | NAD(P)H-hydrate epimerase | Apoa1bp | 23.18358994 | 23.66405487 | NaN | 23.51953316 | 23.17197418 | 23.51074791 |
|  | 0.20518488 | 0.023391088 | Q8BTZ7 | Mannose-1-phosphate guanyltransferase beta | Gmppb | 23.65512657 | 23.74005699 | 23.80360985 | 23.72452736 | 23.71208954 | 23.69200325 |
|  | 0.123003612 | 0.023401896 | Q9JL62;D3Z1H9;D3Z1H8 | Glycolipid transfer protein | Gltp | 25.63989449 | 25.62781334 | 25.49658775 | 25.66180038 | 25.48277664 | 25.54951286 |
|  | 0.104159979 | 0.024447123 | P60766;A0A2R8VH29;G3UZM2;D3Z3L1;F2Z463;D3YX61;Q8R527;Q9ER71 | Cell division control protein 42 homolog | Cdc42 | 27.17321777 | 27.23908806 | 27.25790787 | 27.03988457 | 27.3002491 | 27.25673866 |
|  | 0.1295945 | 0.024490039 | A0A087WNV1;A0A087WSR7;A0A087WR52;Q8K2K6;A0A087WRL1 | Arf-GAP domain and FG repeat-containing protein 1 | Agfg1 | 22.49052238 | NaN | 22.34405136 | 22.44939804 | 22.34177399 | 22.38721848 |
|  | 0.26798347 | 0.02454567 | Q9WUM3;A0A494B9Y4;D3YUG6;A0A494BAI1 | Coronin-1B | Coro1b | 25.39610672 | 25.47515678 | 25.44941139 | 25.4187336 | 25.46307564 | 25.36522865 |
|  | 0.052070019 | 0.024984678 | P10711;E9PYD5;B7ZCS4;F6XMY4;Q9QVN7 | Transcription elongation factor A protein 1 | Tcea1 | 22.97361755 | 22.58359528 | 22.8618145 | 22.86336517 | NaN | 22.69935036 |
|  | 0.054998541 | 0.02507782 | F8WJB9;E9PVP4;A0A1Y7VJA2;P70429 | Ena/VASP-like protein | Evl | 22.27170372 | 22.34843445 | 22.36265755 | 21.99469185 | 22.48397064 | 22.42889977 |
|  | 0.147187225 | 0.0250899 | P49138;A0A087WSN7 | MAP kinase-activated protein kinase 2 | Mapkapk2 | 23.00463486 | 23.15279579 | 23.01824951 | 23.01942253 | 23.04751778 | NaN |
|  | 0.110668514 | 0.025344213 | A2AU62;Q64012;A2AU61;A2AU60 | RNA-binding protein Raly | Raly | 24.14662552 | 24.13233948 | 24.12321091 | 24.16928482 | 24.2115593 | 23.94529915 |
|  | 0.061227705 | 0.025501251 | O70591;F8WJ30 | Prefoldin subunit 2 | Pfdn2 | 23.47097588 | 23.86031723 | 23.92650986 | 23.73396873 | 23.67852592 | 23.76880455 |
|  | 0.101197379 | 0.02550443 | A6H8H2;E9Q449 | DENN domain-containing protein 4C | Dennd4c | 22.10939026 | 22.02877426 | 21.89289474 | 21.91342163 | 21.92679977 | 22.11432457 |
|  | 0.147066047 | 0.025959651 | Q9DC51;A2AE32;P20612;Q3V3I2;P50149;P18872;A2AE31;Q8C040;D3Z2M7;Q8BHK8;F6QPU5 | Guanine nucleotide-binding protein G(k) subunit alpha | Gnai3 | 23.88753319 | 23.93192863 | 23.97805977 | 23.83084106 | 24.02555084 | 23.86325073 |
|  | 0.095967535 | 0.026025136 | P47955 | 60S acidic ribosomal protein P1 | Rplp1 | 26.46351051 | 26.55588913 | 26.53842545 | 26.32271767 | 26.64182472 | 26.51520729 |
|  | 0.039326158 | 0.02624925 | Q3USX5;Q8R4K2 | Interleukin-1 receptor-associated kinase 4 | Irak4 | 22.44245529 | 21.88933372 | 22.08793259 | 22.21543121 | NaN | 22.01188469 |
|  | 0.085895284 | 0.026285807 | Q9D8V0;A3KGR9;Q6PGJ8 | Minor histocompatibility antigen H13 | Hm13;H13 | 24.69535255 | 24.57888222 | 24.66416359 | 24.57680893 | 24.81548309 | 24.46724892 |
|  | 0.146069464 | 0.026316325 | A0A087WQS2;Q9CQC6;A0A087WPF9;A0A087WS48 | Basic leucine zipper and W2 domain-containing protein 1 | Bzw1 | 23.64570427 | 23.67227936 | 23.64460373 | 23.72307014 | 23.66036034 | 23.5002079 |
|  | 0.111485087 | 0.026550293 | Q8VDQ1;Q3TXN1;D6RGL6 | Prostaglandin reductase 2 | Ptgr2 | 22.30591202 | 22.34543419 | 22.4767704 | 22.38213348 | 22.44944954 | 22.21688271 |
|  | 0.511368252 | 0.026556015 | O88958;A0A494B9X2;D3Z0R5;A0A494BB68;A0A494BA15 | Glucosamine-6-phosphate isomerase 1 | Gnpda1 | 25.65867233 | 25.63167572 | 25.62025261 | 25.61641502 | 25.57354736 | 25.64097023 |
|  | 0.163621826 | 0.026599884 | Q8BMD8;A0A0G2JFB9 | Calcium-binding mitochondrial carrier protein SCaMC-1 | Slc25a24 | 24.59553909 | 24.59656334 | 24.64008713 | 24.49947929 | 24.5543232 | 24.69858742 |
|  | 0.398603675 | 0.02660052 | P68372;Q9D6F9;G3UZR1 | Tubulin beta-4B chain;Tubulin beta-4A chain | Tubb4b;Tubb4a | 28.31371689 | 28.34646606 | 28.32414436 | 28.31168365 | 28.25133133 | 28.34151077 |
|  | 0.287766635 | 0.026881536 | E9PZ00;Q8BFQ1;K3W4L3;J3QPG5;Q61207 | Prosaposin | Psap | 31.2443943 | 31.22934532 | 31.15532875 | 31.13350868 | 31.22066498 | 31.19425011 |
|  | 0.047524999 | 0.027112961 | Q9D967 | Magnesium-dependent phosphatase 1 | Mdp1 | NaN | 22.03647614 | 22.31021881 | 21.89544868 | 22.26410484 | 22.27915001 |
|  | 0.162288655 | 0.027338664 | P68181;H6TMF5;A0A0G2JFT9 | cAMP-dependent protein kinase catalytic subunit beta | Prkacb | 23.76080894 | 23.59411621 | 23.75684547 | 23.61952591 | 23.68185425 | 23.72837448 |
|  | 0.064677021 | 0.027591387 | Q8R5H1 | Ubiquitin carboxyl-terminal hydrolase 15 | Usp15 | NaN | 21.86234283 | 22.09934425 | 21.77499008 | 22.05626106 | 22.02850533 |
|  | 0.418692756 | 0.027698517 | P57780;A0A1L1SV25;E9Q2W9;A0A1L1SVJ6;A0A1Y7VMW4 | Alpha-actinin-4 | Actn4 | 26.0943222 | 26.07285881 | 26.13286972 | 26.08745956 | 26.10061073 | 26.02888489 |
|  | 0.428767379 | 0.027905146 | Q6P9Q6;A0A6I8MWZ0;Q80YW9;Q80YW6;Q80YW7 | FK506-binding protein 15;Peptidyl-prolyl cis-trans isomerase | Fkbp15 | 24.57640457 | 24.54686737 | 24.55397224 | 24.49588585 | 24.51490784 | 24.58273506 |
|  | 0.182691003 | 0.028584162 | Q91V92;Q3V117;Q3TS02 | ATP-citrate synthase | Acly | 23.41390038 | 23.46450806 | 23.43987083 | 23.51809311 | 23.3198967 | 23.39453697 |
|  | 0.171754157 | 0.029109955 | Q8CGC7;A0A0A6YWA4;A0A0A6YWH3 | Bifunctional glutamate/proline--tRNA ligase;Glutamate--tRNA ligase;Proline--tRNA ligase | Eprs | 24.52575874 | 24.50057411 | 24.44126511 | 24.3521862 | 24.47258759 | 24.55549431 |
|  | 0.044251136 | 0.02958552 | Q6PFB2;Q8VE37 | Regulator of chromosome condensation | Rcc1 | 23.40340614 | 22.83103371 | 22.86902618 | 23.27084923 | 22.84327888 | 22.90058136 |
|  | 0.145159234 | 0.029747645 | Q9JII5;Q3UGB5;D3Z4J1 | DAZ-associated protein 1 | Dazap1 | 23.01365852 | 23.19200134 | 23.19559479 | 23.12093544 | 23.1752224 | 23.01585388 |
|  | 0.155806522 | 0.029919942 | Q6P069 | Sorcin | Sri | 24.88460159 | 24.74021149 | 24.73003387 | 24.82298851 | 24.65354347 | 24.78855515 |
|  | 0.266731038 | 0.030262629 | Q8K2Q9 | Shootin-1 | Kiaa1598 | 23.97805977 | 23.93382072 | 23.82803345 | 23.86825371 | 23.89836884 | 23.88250351 |
|  | 0.148714282 | 0.030743917 | Q9JKC8;H7BWY2;A0A286YDZ6;D3YXV9;D3YWU3;Q8R2R9;D6RI63 | AP-3 complex subunit mu-1 | Ap3m1 | 24.36753082 | 24.35702705 | 24.30841446 | 24.39682388 | 24.37961006 | 24.16430664 |
|  | 0.136205494 | 0.031768799 | Q68FL4;F8WGT1;F8WI65;H3BKT5;H3BL31;D3YYM7;A0A0N4SUY4;E9PX77;D3YX97;F7ATQ6;D3Z2Q0 | Putative adenosylhomocysteinase 3;Adenosylhomocysteinase | Ahcyl2 | 23.92804718 | 24.0521698 | 23.98633003 | 24.05845833 | 24.00966644 | 23.80311584 |
|  | 0.154863154 | 0.032395681 | Q99JZ4;P36536;A0A1W2P869;A0A1W2P6N3;A0A1W2P720 | GTP-binding protein SAR1a | Sar1a | 24.76430702 | 24.6238842 | 24.67195702 | 24.52414513 | 24.74309158 | 24.69572449 |
|  | 0.147633932 | 0.032529195 | Q9DBH5 | Vesicular integral-membrane protein VIP36 | Lman2 | 24.23812294 | 24.07439041 | 24.29291534 | 24.26463509 | 24.14211273 | 24.10109329 |
|  | 0.081299734 | 0.032875061 | Q3UM45;F6TGJ2;A0A087WRA7 | Protein phosphatase 1 regulatory subunit 7 | Ppp1r7 | 22.91067886 | 23.12374687 | 23.36439514 | 23.05105782 | 23.03573799 | 23.21339989 |
|  | 0.208766214 | 0.033360163 | Q80WJ7;F6QHD1;E9PUX0;F6ZQL0;F6ZSG0;F6QFT1 | Protein LYRIC | Mtdh | 24.58353806 | 24.37669945 | 24.45882607 | 24.44859314 | 24.45920181 | 24.41118813 |
|  | 0.331227428 | 0.033827464 | Q9Z1Z0;A0A0J9YUG0 | General vesicular transport factor p115 | Uso1 | 24.46201324 | 24.49155045 | 24.38060188 | 24.45951462 | 24.37125778 | 24.40191078 |
|  | 0.424831214 | 0.034213384 | A0A1D5RLG3;Q80UJ7 | Rab3 GTPase-activating protein catalytic subunit | Rab3gap1 | 21.29075241 | NaN | 21.35804749 | 21.3226223 | 21.26779556 | 21.28014183 |
|  | 0.091670097 | 0.034453074 | F6ZDS4;Q7M739;F6RX08 | Nucleoprotein TPR | Tpr | 22.64627647 | 22.63145256 | 22.70967293 | 22.88768005 | 22.53734779 | 22.45901489 |
|  | 0.270830122 | 0.034578959 | Q9ER72;A0A140LIB6 | Cysteine--tRNA ligase, cytoplasmic | Cars | 23.37703133 | 23.35555077 | 23.5118351 | 23.38613892 | 23.40262604 | 23.35191536 |
|  | 0.300523694 | 0.034905752 | O70310;F7APP3;A2AJH3;O70311 | Glycylpeptide N-tetradecanoyltransferase 1 | Nmt1 | 24.06142807 | 23.97184753 | 23.98294067 | 24.04218292 | 23.95571136 | 23.91360474 |
|  | 0.829074578 | 0.035084407 | Q6GT24;D3Z0Y2;O08709;Q8BG37;A0A0A6YXQ7 | Peroxiredoxin-6 | Prdx6 | 25.88175774 | 25.89282227 | 25.88002968 | 25.8429718 | 25.8859272 | 25.82045746 |
|  | 1.373371345 | 0.035121918 | Q99P72 | Reticulon-4 | Rtn4 | 26.78668594 | 26.77718353 | 26.80796242 | 26.74008179 | 26.76205254 | 26.76433182 |
|  | 0.204103815 | 0.035572052 | A0A1L1STE6;Q9D6R2 | Isocitrate dehydrogenase [NAD] subunit alpha, mitochondrial | Idh3a | 24.45205307 | 24.61705971 | 24.49344444 | 24.4368248 | 24.57623291 | 24.44278336 |
|  | 0.910779622 | 0.036738078 | P48025;E9PWE9;P43404 | Tyrosine-protein kinase SYK;Tyrosine-protein kinase | Syk | 25.46883392 | 25.51050758 | 25.47741318 | 25.43055344 | 25.47611618 | 25.43987083 |
|  | 0.104671978 | 0.037022273 | Q91VN4;E9Q4M4 | MICOS complex subunit Mic25 | Chchd6 | 22.69144821 | 22.86413956 | 22.79169655 | 22.70994568 | 22.9637394 | 22.56253242 |
|  | 0.212927956 | 0.037032445 | Q9WTI7 | Unconventional myosin-Ic | Myo1c | 23.81088257 | 23.61178017 | 23.69540596 | 23.69774055 | 23.70988274 | 23.59934807 |
|  | 0.366862076 | 0.037079493 | Q8VC04;A2A4M9;A2A4N0 | Transmembrane protein 106A | Tmem106a | 24.53261185 | 24.40756226 | 24.41441727 | 24.4307766 | 24.42085457 | 24.39172173 |
|  | 0.103405974 | 0.037150065 | Q9JHF7 | Hematopoietic prostaglandin D synthase | Hpgds | 25.20914459 | 25.2096653 | 24.89217377 | 24.92370224 | 25.10177422 | 25.17405701 |
|  | 0.077288077 | 0.037519455 | Q80XR8;J3QK07;D3Z1I2;E9PVA6;Q9JLQ2;F6U8T2;F7BIK4;F6SLJ2;F6WV69;D3Z409 | ARF GTPase-activating protein GIT2 | Git2 | NaN | 22.44460487 | 22.16171074 | NaN | 22.34161186 | 22.18966484 |
|  | 0.144292215 | 0.03758049 | Q9DCJ5 | NADH dehydrogenase [ubiquinone] 1 alpha subcomplex subunit 8 | Ndufa8 | 23.05903625 | 22.81690788 | 22.84919167 | 22.87891769 | 22.76324272 | 22.97023392 |
|  | 0.502442354 | 0.038332621 | Q3TRM8;E9Q3Z4;E9Q8S8;D6RFA3;D3Z2E4 | Hexokinase-3;Hexokinase | Hk3 | 25.73081207 | 25.73520851 | 25.78957367 | 25.76162148 | 25.66644096 | 25.71253395 |
|  | 0.113401031 | 0.038496017 | Q9CWZ7;A0A494BAE3;A0A494BA20;D3Z4B2;A0A494BBF5;A0A494BA49;A0A494BAX2 | Gamma-soluble NSF attachment protein | Napg | 23.23390198 | 23.20910645 | 23.43377113 | 23.07744217 | 23.25824928 | 23.42560005 |
|  | 0.16847622 | 0.038512548 | Q61166 | Microtubule-associated protein RP/EB family member 1 | Mapre1 | 24.90554619 | 24.9682045 | 25.13852501 | 24.93656349 | 25.06476212 | 24.89541245 |
|  | 0.353031392 | 0.038675308 | P42208;E9Q3V6;F6WYM0;D3YYB1;D3Z3C0;D3Z1S1;F6UKN5;D3YZU7;D3YV76;G3UYQ0 | Septin-2 | Septin2 | 25.0582943 | 25.18468857 | 25.15957832 | 25.12281609 | 25.04743385 | 25.11628532 |
|  | 0.236016951 | 0.038808823 | Q8BJW6;D3YZZ6;D6RFN2;D6RGA6 | Eukaryotic translation initiation factor 2A;Eukaryotic translation initiation factor 2A, N-terminally processed | Eif2a | 23.47555923 | 23.62834167 | 23.46973419 | 23.46811867 | 23.55952644 | 23.42956352 |
|  | 1.522848105 | 0.038874308 | P11983;F2Z483;A0A3B2WDE2 | T-complex protein 1 subunit alpha | Tcp1 | 25.18218994 | 25.20631409 | 25.19005013 | 25.13516045 | 25.16334724 | 25.16342354 |
|  | 0.153994108 | 0.038989385 | A0A087WRZ7;A0A0G2JDM3;E9PWG4;A0A0G2JDW2;P05977;P09542 | Myosin light chain 1/3, skeletal muscle isoform;Myosin light chain 3 | Myl1;Myl3 | 26.76179886 | 26.73918152 | 26.52229118 | 26.74059677 | 26.54988098 | 26.61582565 |
|  | 0.059024696 | 0.039032618 | P63024;B0QZN5;P63044 | Vesicle-associated membrane protein 3 | Vamp3 | 25.18703079 | 25.075737 | 24.73660278 | 24.75394249 | 25.32906342 | 24.79926682 |
|  | 0.39374279 | 0.039283752 | P51863 | V-type proton ATPase subunit d 1 | Atp6v0d1 | 26.17822456 | 26.3023777 | 26.2444706 | 26.20594215 | 26.16248322 | 26.23879623 |
|  | 0.206062863 | 0.040245692 | A0A0R4J078;Q8VCH8;A0A087WSK5 | UBX domain-containing protein 4 | Ubxn4 | 22.88412666 | 22.80939102 | 22.75372696 | 22.86640549 | 22.64869118 | 22.8114109 |
|  | 0.495179023 | 0.040353139 | Q9QUM9;E0CXB1;E0CYT2 | Proteasome subunit alpha type-6 | Psma6 | 26.16449738 | 26.2053833 | 26.19159508 | 26.19163132 | 26.16742897 | 26.08135605 |
|  | 0.216416805 | 0.040999095 | Q9QZ06;Q8C5G6;A9JEI5;F7AT44 | Toll-interacting protein | Tollip | 23.66448975 | 23.74406624 | 23.80813408 | 23.80124283 | 23.59057999 | 23.70186996 |
|  | 0.545813724 | 0.041623433 | P27659;A0A2R8VHN4;A0A087WQK0;A0A087WNS0;Q9CQ09;E9PWZ3 | 60S ribosomal protein L3 | Rpl3 | 25.94514275 | 26.04558182 | 25.97394943 | 25.93786621 | 25.92492676 | 25.97701073 |
|  | 0.482646312 | 0.04173851 | A2A813;Q99LX0;A2A815;A2A817;A2A816 | Protein deglycase DJ-1 | Park7 | 25.90345764 | 26.02533913 | 25.94891167 | 25.89314651 | 25.92861176 | 25.93073463 |
|  | 0.206369824 | 0.041812897 | P97379;A0A0J9YUY8;A0A0J9YTQ8 | Ras GTPase-activating protein-binding protein 2 | G3bp2 | 23.71931458 | 23.47716713 | 23.6417408 | 23.62889671 | 23.51749229 | 23.56639481 |
|  | 0.164095145 | 0.041976929 | Q8VBZ3 | Cleft lip and palate transmembrane protein 1 homolog | Clptm1 | 23.24767113 | 23.37477684 | 23.0626812 | 23.1317749 | 23.18304443 | 23.24437904 |
|  | 0.144228109 | 0.043118159 | Q3THK7 | GMP synthase [glutamine-hydrolyzing] | Gmps | 22.73378181 | 22.91323853 | 22.70790482 | 22.63393784 | 22.92114449 | 22.67048836 |
|  | 0.439088673 | 0.044019699 | O88325 | Alpha-N-acetylglucosaminidase | Naglu | 24.60845566 | 24.55180359 | 24.58880806 | 24.49087715 | 24.50845337 | 24.61767769 |
|  | 0.657202091 | 0.044101079 | P97855 | Ras GTPase-activating protein-binding protein 1 | G3bp1 | 24.37026024 | 24.29991722 | 24.351511 | 24.25673866 | 24.30026627 | 24.33238029 |
|  | 0.257290221 | 0.044549942 | Q9CYG7 | Mitochondrial import receptor subunit TOM34 | Tomm34 | 23.28827667 | 23.35110664 | 23.2990799 | 23.35917473 | 23.13972664 | 23.30591202 |
|  | 0.471892184 | 0.044718424 | A2AMW0;F7CAZ6 | F-actin-capping protein subunit beta | Capzb | 26.58132744 | 26.58204651 | 26.66383743 | 26.54406929 | 26.62449837 | 26.52448845 |
|  | 0.328079599 | 0.044761658 | O54988;A0A5F8MPV2 | STE20-like serine/threonine-protein kinase | Slk | 23.65937996 | 23.54827881 | 23.48628044 | 23.50275993 | 23.48922348 | 23.56767082 |
|  | 0.902127113 | 0.045566559 | P54071;D6RIL6;A0A0U1RP68;A0A0U1RPR1 | Isocitrate dehydrogenase [NADP], mitochondrial | Idh2 | 24.91966248 | 24.94127464 | 24.98272324 | 24.88664818 | 24.888834 | 24.9314785 |
|  | 0.527573467 | 0.045882543 | P14206;A0A1L1SUK3;A0A1L1SRW0 | 40S ribosomal protein SA | Rpsa | 27.14228821 | 27.24843788 | 27.18555832 | 27.16365433 | 27.10119247 | 27.17378998 |
|  | 0.160464646 | 0.046182632 | A0A494B952;Q91V64 | Isochorismatase domain-containing protein 1 | Isoc1 | 23.223629 | 23.40353584 | 23.42278099 | 23.17250824 | 23.46961021 | 23.26927948 |
|  | 0.502963812 | 0.046236674 | P62196;Q8K1K2;A0A0E2WI80 | 26S protease regulatory subunit 8 | Psmc5 | 24.43638039 | 24.42162514 | 24.33483696 | 24.33210564 | 24.40047836 | 24.32154846 |
|  | 1.092583094 | 0.046649933 | Q8BU88 | 39S ribosomal protein L22, mitochondrial | Mrpl22 | NaN | 22.25037766 | 22.27863884 | 22.21767998 | NaN | 22.21803665 |
|  | 0.146598061 | 0.047092438 | Q3THW5;P0C0S6;Q8R029;Q3UA95 | Histone H2A.V;Histone H2A.Z;Histone H2A | H2afv;H2afz | 25.29967308 | 25.5785656 | 25.29726028 | 25.20564461 | 25.46054649 | 25.36803055 |
|  | 1.245867992 | 0.04728508 | Q8BLF1;Q8BYQ0;D6RGP7 | Neutral cholesterol ester hydrolase 1 | Nceh1 | 26.28346252 | 26.26597595 | 26.30016327 | 26.26087189 | 26.2095356 | 26.23733902 |
|  | 0.21578852 | 0.047312419 | Q9CPX6 | Ubiquitin-like-conjugating enzyme ATG3 | Atg3 | 22.97479057 | 22.98039818 | NaN | 22.98697281 | 22.80207062 | 23.00180244 |
|  | 1.495198652 | 0.047924042 | P06745;A0A0U1RQ72;A0A0U1RP97;A0A0U1RQ18 | Glucose-6-phosphate isomerase | Gpi | 26.58677673 | 26.55681038 | 26.60724258 | 26.54017067 | 26.53204727 | 26.53483963 |
|  | 0.634623759 | 0.048482895 | Q01853 | Transitional endoplasmic reticulum ATPase | Vcp | 26.93269539 | 27.00624466 | 27.02178764 | 26.91957092 | 26.91581535 | 26.97989273 |
|  | 0.283008105 | 0.048724492 | A0A087WPL5;E9QNN1;O70133;Q3UR42;A0A0R4J2C3;A0A087WRT3 | ATP-dependent RNA helicase A | Dhx9 | 23.9216156 | 23.90297508 | 24.08472443 | 23.98224449 | 23.85004997 | 23.93084717 |
|  | 0.410085462 | 0.049455007 | P14685;F7B7L8 | 26S proteasome non-ATPase regulatory subunit 3 | Psmd3 | 24.04159737 | 23.94511986 | 23.88259697 | 23.8901329 | 23.95064735 | 23.88016891 |
|  | 0.459120567 | 0.049477259 | A0A0R4J1C8;P31996 | Macrosialin | Cd68 | 28.56034279 | 28.50502586 | 28.42113495 | 28.48250771 | 28.45187378 | 28.40369034 |
|  | 0.331807838 | 0.049488703 | Q91VC9;A0A286YDK2 | Growth hormone-inducible transmembrane protein | Ghitm | 23.51713181 | 23.41041183 | 23.5932045 | 23.45651054 | 23.40405655 | 23.51171494 |
|  | 0.082626428 | 0.049601237 | Q8BXC6;G3X955 | COMM domain-containing protein 2 | Commd2 | 22.56973457 | 22.77213287 | 22.6902771 | 22.93535042 | 22.70619774 | 22.24179268 |
|  | 0.213212014 | 0.049700419 | Q62095 | ATP-dependent RNA helicase DDX3Y | Ddx3y | 22.71466827 | 22.56899261 | 22.5151844 | 22.5138588 | 22.68168259 | 22.45420265 |
|  | 0.081712167 | 0.05001386 | A0A0R4J034;Q99K01;D3YZA7 | Pyridoxal-dependent decarboxylase domain-containing protein 1 | Pdxdc1 | 23.06496811 | 23.29080772 | 23.76466179 | 23.36746407 | 23.40820885 | 23.19472313 |
|  | 0.371189511 | 0.050021489 | P45952;D3Z2A5 | Medium-chain specific acyl-CoA dehydrogenase, mitochondrial | Acadm | 24.27717781 | 24.38093185 | 24.38343811 | 24.37650108 | 24.22354126 | 24.29144096 |
|  | 0.537954747 | 0.050093969 | Q62167;P16381 | ATP-dependent RNA helicase DDX3X;Putative ATP-dependent RNA helicase Pl10 | Ddx3x;D1Pas1 | 24.86405563 | 24.80405235 | 24.78236771 | 24.83156586 | 24.74534988 | 24.72327805 |
|  | 0.175800165 | 0.050258001 | Q9R1T2 | SUMO-activating enzyme subunit 1;SUMO-activating enzyme subunit 1, N-terminally processed | Sae1 | 23.4648819 | 23.27994347 | 23.47271156 | 23.18475533 | 23.40249634 | 23.47951126 |
|  | 0.461764199 | 0.050287882 | P16045;A0A2R8VHJ0 | Galectin-1 | Lgals1 | 30.55545044 | 30.5327816 | 30.50436401 | 30.56010437 | 30.47604942 | 30.40557861 |
|  | 0.445179232 | 0.050697962 | Q99JI6;A0A1W2P777 | Ras-related protein Rap-1b | Rap1b | 26.33791733 | 26.41126823 | 26.47880173 | 26.33796883 | 26.41256142 | 26.32536316 |
|  | 0.293520453 | 0.051130295 | Q6PB44;A0A0G2JEW5 | Tyrosine-protein phosphatase non-receptor type 23 | Ptpn23 | 22.24725151 | 22.42508698 | 22.26476288 | 22.2520237 | 22.338377 | 22.19330978 |
|  | 1.10782353 | 0.051160812 | Q8CI51;D9J2Z9;D9J300;D9J301;D9J302;A0A0G2JGZ5;A0A0G2JEJ0;Q9CRA2;D9J303;F8WJI6;E9Q8P5 | PDZ and LIM domain protein 5 | Pdlim5 | 24.00075531 | 24.0010128 | 23.94833183 | 23.95721817 | 23.92469978 | 23.91469955 |
|  | 0.254671286 | 0.05124855 | O08583;G3X9I4;Q9JJW6 | THO complex subunit 4;Aly/REF export factor 2 | Alyref;Alyref2 | 24.70482635 | 24.48031425 | 24.61548996 | 24.6408596 | 24.51062775 | 24.49539757 |
|  | 0.156976086 | 0.051448822 | Q60749;A0A087WSL3;A0A087WR32;A0A2R8VJR4;Q9R226;Q9WU01 | KH domain-containing, RNA-binding, signal transduction-associated protein 1 | Khdrbs1 | 23.95153809 | 24.09055138 | 23.71502495 | 23.79847717 | 23.82745361 | 23.97683716 |
|  | 0.628473978 | 0.051823934 | P29351;G3UYY5;G3UXM2;G3UZU6 | Tyrosine-protein phosphatase non-receptor type 6 | Ptpn6 | 25.51851273 | 25.49109077 | 25.56726456 | 25.44638443 | 25.5331459 | 25.44186592 |
|  | 0.21901209 | 0.051854451 | Q6PDI5;A2ALV7;A2ALV6;A2ALV8;A2ALV9;A2ALW1 | Proteasome-associated protein ECM29 homolog | Ecm29;AI314180 | 22.14385796 | 22.01709557 | 22.13855553 | 21.97697639 | 22.2122879 | 21.9546814 |
|  | 0.173669255 | 0.051867167 | P26516 | 26S proteasome non-ATPase regulatory subunit 7 | Psmd7 | 24.39786911 | 24.52133179 | 24.69290733 | 24.58399773 | 24.34055138 | 24.53195763 |
|  | 2.169759771 | 0.052033742 | Q9JHU4;F6ZX84 | Cytoplasmic dynein 1 heavy chain 1 | Dync1h1 | 25.56412697 | 25.5637207 | 25.57111931 | 25.50254822 | 25.53380013 | 25.50651741 |
|  | 0.437302411 | 0.052687327 | P27612;F7D1R5 | Phospholipase A-2-activating protein | Plaa | 23.04403496 | 23.05270004 | 23.10315895 | 23.03056145 | 23.08794975 | 22.92332077 |
|  | 0.277745745 | 0.052879333 | Q9CR00;A0A0G2JGN6 | 26S proteasome non-ATPase regulatory subunit 9 | Psmd9 | 23.60478592 | 23.55917549 | 23.46973419 | 23.45487976 | 23.61885262 | 23.40132523 |
|  | 1.898726734 | 0.053054174 | Q61233;D3YZ25;D3YVW8;D3Z7D9;D3Z311 | Plastin-2 | Lcp1 | 28.39415169 | 28.41094398 | 28.42642403 | 28.34129906 | 28.36527824 | 28.36577988 |
|  | 0.323901322 | 0.053110123 | O89086;Q8BG13;S4R2M6 | RNA-binding protein 3 | Rbm3 | 25.96146393 | 25.94935608 | 25.80784035 | 25.89113045 | 25.76184845 | 25.90635109 |
|  | 0.736191412 | 0.054230372 | P46638;G3UY29;E9Q3P9;F8WGS1;P62492;G3UZD3;G3UZL4;E9Q6B3;Q9WTL2 | Ras-related protein Rab-11B;Ras-related protein Rab-11A | Rab11b;Rab11a | 26.42337418 | 26.34670258 | 26.36666298 | 26.34319878 | 26.35519791 | 26.27565193 |
|  | 0.304566102 | 0.054281235 | Q8C0E3 | Tripartite motif-containing protein 47 | Trim47 | 22.51019287 | 22.40036011 | 22.43002319 | 22.2806797 | 22.39220619 | 22.50484657 |
|  | 0.328075534 | 0.054309209 | A2ATI9;Q99JX3;A2ATI8;A2ATI6 | Golgi reassembly-stacking protein 2 | Gorasp2 | 23.22610092 | 23.43109512 | 23.29109001 | 23.30521584 | 23.2010498 | 23.27909279 |
|  | 0.280647192 | 0.054650625 | G5E8V9;E9QAY5 | Arfaptin-1 | Arfip1 | 24.17797661 | 24.2473526 | 24.09538841 | 24.21475029 | 23.99515724 | 24.14685822 |
|  | 0.325361488 | 0.055138906 | Q99KP6 | Pre-mRNA-processing factor 19 | Prpf19 | 23.92506218 | 23.84794998 | 23.81009865 | 23.92524338 | 23.72546387 | 23.76698685 |
|  | 0.191838148 | 0.055978139 | Q921K2;P11103;A0A0A6YY63 | Poly [ADP-ribose] polymerase 1 | Parp1 | 22.71895981 | 22.97587585 | 22.76476288 | 22.73508453 | 22.64372444 | 22.91285515 |
|  | 0.611129133 | 0.056238174 | P45376;D3YVJ7 | Aldose reductase | Akr1b1;Akr1b3 | 26.44362068 | 26.55607986 | 26.54147148 | 26.49814034 | 26.42551994 | 26.44879723 |
|  | 1.483981187 | 0.056849798 | Q9D8U8 | Sorting nexin-5 | Snx5 | 26.11898041 | 26.16401863 | 26.17136765 | 26.08310318 | 26.09391975 | 26.10679436 |
|  | 0.181952629 | 0.056861242 | Q61187;A0A1B0GRX2;A0A1B0GS10;A0A1B0GS09;D3Z2V5;D3Z0S9 | Tumor susceptibility gene 101 protein | Tsg101 | 23.13183784 | 23.22535133 | 23.09160042 | 23.2731266 | 22.88689995 | 23.11817932 |
|  | 0.160813413 | 0.057488124 | P70202 | Latexin | Lxn | 24.17888832 | 24.10765266 | 24.49856567 | 24.14258003 | 24.14631462 | 24.32374763 |
|  | 2.042545987 | 0.057725906 | Q91VI7;A0A1B0GSG5;A0A1B0GRG4;A0A1B0GRY7 | Ribonuclease inhibitor | Rnh1 | 27.89585114 | 27.92863464 | 27.89954376 | 27.83791733 | 27.85352898 | 27.85940552 |
|  | 0.899450055 | 0.057788213 | Q6ZWQ9;D3YV37 | Myosin, light chain 12A, regulatory, non-sarcomeric | Myl12a | 26.44854546 | 26.44577026 | 26.49324608 | 26.38396645 | 26.45586777 | 26.37436295 |
|  | 0.958353711 | 0.057910919 | P84096;A0A1B0GSL4 | Rho-related GTP-binding protein RhoG | Rhog | 27.33483696 | 27.32268333 | 27.27896881 | 27.24726105 | 27.21900368 | 27.29649162 |
|  | 0.209202057 | 0.057953517 | P24547;A0A0A6YY72;A0A0A6YXS4 | Inosine-5-monophosphate dehydrogenase 2 | Impdh2 | 24.12518311 | 24.30827522 | 24.1331234 | 24.30910873 | 24.04201508 | 24.04159737 |
|  | 0.438151707 | 0.058018366 | Q9JMA1;E9PYI8 | Ubiquitin carboxyl-terminal hydrolase 14;Ubiquitin carboxyl-terminal hydrolase | Usp14 | 24.56592941 | 24.59860992 | 24.42649651 | 24.43103218 | 24.48689461 | 24.49905396 |
|  | 0.705061471 | 0.058516184 | Q9D8W5;B1AT36;Q3TRH2 | 26S proteasome non-ATPase regulatory subunit 12 | Psmd12 | 24.35790062 | 24.30646896 | 24.31982613 | 24.20232201 | 24.31734467 | 24.28898048 |
|  | 0.427905707 | 0.058692932 | P09055 | Integrin beta-1 | Itgb1 | 24.07806015 | 24.09996986 | 24.01444054 | 23.91250801 | 24.09498596 | 24.00889778 |
|  | 0.381676009 | 0.059435527 | P17426;A0A140LIG7;A0A140LHG0;A0A140LHA6 | AP-2 complex subunit alpha-1 | Ap2a1 | 27.69677162 | 27.55340004 | 27.61484337 | 27.66343117 | 27.51292038 | 27.5103569 |
|  | 1.552122791 | 0.059657415 | P13020;A0A0J9YUQ8;A0A0J9YUJ8 | Gelsolin | Gsn | 28.08502007 | 28.13838768 | 28.0866394 | 28.0411911 | 28.0486927 | 28.0411911 |
|  | 1.161959574 | 0.060154597 | G3UXZ5;P97371;G3UXY0;G3X9K9;G3UWN9;G3UXR1 | Proteasome activator complex subunit 1 | Psme1 | 25.06081009 | 25.04518509 | 25.06455612 | 25.04272461 | 24.98281097 | 24.96455193 |
|  | 1.069994469 | 0.060585022 | Q9CQI6;A0A1D5RLP1 | Coactosin-like protein | Cotl1 | 28.50039864 | 28.52133179 | 28.48108292 | 28.39693069 | 28.47954178 | 28.4445858 |
|  | 0.264930752 | 0.060621262 | Q99KE1 | NAD-dependent malic enzyme, mitochondrial | Me2 | 22.84166718 | 22.92103386 | 22.64010811 | 22.70849419 | 22.81380081 | 22.69865036 |
|  | 0.364649144 | 0.061129252 | A0A1B0GT81;Q07813;A0A1B0GS13;A0A1B0GTA4;A0A1B0GT35 | Apoptosis regulator BAX | Bax | 24.73649979 | 24.68330002 | 24.7928791 | 24.80099678 | 24.62215233 | 24.60614204 |
|  | 0.432866252 | 0.061398188 | P43277 | Histone H1.3 | Hist1h1d | 26.82941437 | 26.82650757 | 26.66830826 | 26.73259735 | 26.75149155 | 26.65594673 |
|  | 0.24167195 | 0.061445236 | E9Q7G1;D3YZZ5 | Transmembrane p24 trafficking protein 7 | Tmed7 | 24.36646271 | 24.39551735 | 24.51791382 | 24.18744659 | 24.45888901 | 24.44922256 |
|  | 0.298038288 | 0.061535835 | A0A140LIU9;P50637 | Translocator protein | Tspo | 24.13758659 | 24.14297104 | 23.96710396 | NaN | 24.06406403 | 23.97797203 |
|  | 0.25845338 | 0.061932882 | Q9Z0M5 | Lysosomal acid lipase/cholesteryl ester hydrolase | Lipa | 27.39713478 | 27.43069649 | 27.46411896 | 27.51638031 | 27.19582367 | 27.3939476 |
|  | 0.299673404 | 0.062264125 | Q9EPB4;A0A0U1RQ20 | Apoptosis-associated speck-like protein containing a CARD | Pycard | 24.62706184 | 24.4790802 | 24.66030502 | 24.64619827 | 24.43039322 | 24.5030632 |
|  | 0.446406589 | 0.062335968 | Q3UE92;Q6P1B1;S4R1I3;A0A494BBG8;S4R228 | Xaa-Pro aminopeptidase 1 | Xpnpep1 | 23.81225395 | 23.68431664 | 23.6858139 | 23.6866703 | 23.58313751 | 23.72556877 |
|  | 0.339075899 | 0.062989235 | H3BKD4;H3BKE6;E9QMI7;E9QMJ1;H3BL41;H3BJY2;E9QN63;Q9QWY8 | Arf-GAP with SH3 domain, ANK repeat and PH domain-containing protein 1 | Asap1 | 22.053545 | 22.03935623 | 22.01331711 | 22.0697937 | NaN | 21.87504005 |
|  | 0.411432737 | 0.063041687 | Q3ULJ0;D3Z0L6;E0CXN5;P13707 | Glycerol-3-phosphate dehydrogenase 1-like protein | Gpd1l | 23.34719276 | 23.56732368 | 23.49935722 | 23.40807915 | 23.40470695 | 23.41196251 |
|  | 0.156752941 | 0.063127518 | Q9CRD2 | ER membrane protein complex subunit 2 | Emc2 | 22.79659653 | 23.31534195 | 23.09469604 | 22.98450661 | 23.02744293 | 23.00530243 |
|  | 0.184789858 | 0.063810349 | O35593 | 26S proteasome non-ATPase regulatory subunit 14 | Psmd14 | 24.77560234 | 24.50045204 | 24.5217514 | 24.39682388 | 24.4855442 | 24.72400665 |
|  | 1.826460997 | 0.063952764 | Q9DCR2;A0A286YD58;A0A286YDU3 | AP-3 complex subunit sigma-1 | Ap3s1 | 23.77926445 | 23.83016396 | 23.79966354 | 23.73355484 | 23.74909019 | 23.73458862 |
|  | 0.464420516 | 0.064017614 | P21279;A0A494BBL5;P30677;P21278;A0A0G2JG40;O70443;P27600 | Guanine nucleotide-binding protein G(q) subunit alpha | Gnaq | 23.71690941 | 23.76263428 | 23.74273109 | 23.69890594 | 23.7644577 | 23.56685829 |
|  | 0.212980074 | 0.064135869 | A0A0R4IZY0;Q8C1A5 | Thimet oligopeptidase | Thop1 | 22.64897537 | 22.45332336 | 22.57239723 | 22.375494 | 22.40947914 | 22.69731522 |
|  | 0.362470514 | 0.064204534 | P63087;A0A0G2JGC1;A0A0G2JFF1 | Serine/threonine-protein phosphatase PP1-gamma catalytic subunit | Ppp1cc | 23.42316437 | 23.60997772 | 23.52193069 | 23.44783592 | 23.36999512 | 23.54462814 |
|  | 0.180585496 | 0.064479828 | G3UX35;A0A0R4J170;Q3TKT4;E9QAB8;H3BJK2;F2Z4A9;H3BLH0;Q6DIC0 | Transcription activator BRG1;Probable global transcription activator SNF2L2 | Smarca4;Smarca2 | 21.61427689 | 21.45653534 | NaN | NaN | 21.56922531 | 21.37262726 |
|  | 0.185891508 | 0.064627647 | D3Z0M9 | RNA helicase | Ddx23 | NaN | 21.57579422 | 21.48433876 | 21.64570427 | 21.43593407 | 21.31467819 |
|  | 0.549870437 | 0.06465594 | Q61081;A0A1L1STC0 | Hsp90 co-chaperone Cdc37;Hsp90 co-chaperone Cdc37, N-terminally processed | Cdc37 | 24.34577179 | 24.31547928 | 24.26040459 | 24.17592239 | 24.32991982 | 24.22184563 |
|  | 0.270345852 | 0.064841588 | P70245;A2AC29 | 3-beta-hydroxysteroid-Delta(8),Delta(7)-isomerase | Ebp | 24.75399208 | 24.7163868 | 24.89342308 | 24.86655617 | 24.71082878 | 24.59189224 |
|  | 0.291750173 | 0.065226237 | Q02819;A0A1C7CYU3;A0A1B0GR41;A0A1B0GR92;A0A1B0GS16;A0A1B0GT83 | Nucleobindin-1 | Nucb1 | 24.00418854 | 23.85642815 | 23.81313515 | 23.93696785 | 23.84325981 | 23.69784546 |
|  | 0.273518563 | 0.065601985 | Q9D1E6 | Tubulin-folding cofactor B | Tbcb | 23.24569893 | 23.00317764 | 23.13780594 | 23.19428825 | 22.9865551 | 23.0090332 |
|  | 0.843930463 | 0.06591479 | O08992;Q3TMX0;H3BLG5;A2AKJ9;A2AKJ6;A2AKJ5 | Syntenin-1 | Sdcbp | 24.57403946 | 24.61739731 | 24.66638565 | 24.50675964 | 24.56319618 | 24.59012222 |
|  | 0.300040495 | 0.066045761 | G3X9U9;Q9CQ92 | Mitochondrial fission 1 protein | Fis1 | 23.54886627 | 23.6105423 | 23.35447502 | 23.51653099 | 23.43923569 | 23.35997963 |
|  | 0.122058947 | 0.066109975 | Q3UID0;Q6PDG5;A0A1W2P6N7;Q3UNN4;P97496 | SWI/SNF complex subunit SMARCC2 | Smarcc2 | 22.59564209 | 22.20952415 | 22.48736 | 22.49928474 | 22.04423523 | 22.55067635 |
|  | 1.111278008 | 0.066555659 | Q9DBC7;A2AI69;D3Z0V6;P12849;D3Z068 | cAMP-dependent protein kinase type I-alpha regulatory subunit;cAMP-dependent protein kinase type I-alpha regulatory subunit, N-terminally processed | Prkar1a | 24.73184776 | 24.77489853 | 24.78994751 | 24.72536087 | 24.7166481 | 24.65501785 |
|  | 0.296734577 | 0.066678365 | B2RPU8;D3Z5B1;Q9D1L0 | Coiled-coil-helix-coiled-coil-helix domain-containing protein 2 | Zbed5;Chchd2 | 24.49393272 | 24.38922882 | 24.61318588 | 24.31139755 | 24.53034973 | 24.45456505 |
|  | 0.727720883 | 0.066738129 | Q9CZM2;B8JKK2 | 60S ribosomal protein L15;Ribosomal protein L15 | Rpl15 | 26.69564438 | 26.60710144 | 26.59917641 | 26.55749702 | 26.6205883 | 26.52362251 |
|  | 0.682816887 | 0.066982269 | Q6ZWX6 | Eukaryotic translation initiation factor 2 subunit 1 | Eif2s1 | 25.12813568 | 25.16779327 | 25.2149353 | 25.15692139 | 25.12036514 | 25.03263092 |
|  | 0.598816549 | 0.067409515 | O35382;Q8C391;Q9CXE1 | Exocyst complex component 4 | Exoc4 | 22.52288818 | 22.39639282 | 22.53250504 | 22.45573235 | 22.42319107 | 22.37063408 |
|  | 0.259104471 | 0.068007787 | S4R1X1;Q7TMQ7;D3Z101;D3Z0V8 | WD repeat-containing protein 91 | Wdr91 | 23.68731117 | 23.5926342 | 23.41712379 | 23.3754406 | 23.50627518 | 23.61133003 |
|  | 0.342150076 | 0.068187078 | P70206;Q80UG2 | Plexin-A1 | Plxna1 | 21.9671917 | 22.10919952 | 22.02779579 | 21.82493019 | 22.05430794 | 22.02038765 |
|  | 0.396283061 | 0.068206151 | A2A5V3;A2A5V2;P55194;S4R2D3;A0A2R8W6I7;S4R2T6;A2A5V1 | SH3 domain-binding protein 1 | Sh3bp1 | 23.08020973 | 22.94157791 | 23.18731117 | 22.98055458 | 22.99279213 | 23.03113365 |
|  | 0.648417587 | 0.068391164 | Q99JI4;A0A286YDW8 | 26S proteasome non-ATPase regulatory subunit 6 | Psmd6 | 24.3558197 | 24.42515182 | 24.47994423 | 24.37828827 | 24.38798141 | 24.28947258 |
|  | 0.482645587 | 0.068715413 | Q99KV1;A0A338P778 | DnaJ homolog subfamily B member 11 | Dnajb11 | 25.1273098 | 25.1958046 | 25.1315937 | 25.19824219 | 25.03090668 | 25.01941299 |
|  | 0.18245876 | 0.068785667 | Q63829 | COMM domain-containing protein 3 | Commd3 | 22.84622955 | 23.05556488 | 23.20113945 | 23.02440262 | 22.90664864 | NaN |
|  | 1.720637795 | 0.069016139 | Q9D031;Q01730;A2AUR7;B1AYQ0;E0CXG5;A0A0A6YWZ2 | Ras suppressor protein 1 | Rsu1 | 24.24930763 | 24.27490616 | 24.27753258 | 24.22956657 | 24.18533134 | 24.17980003 |
|  | 1.325833106 | 0.069080353 | Q60864 | Stress-induced-phosphoprotein 1 | Stip1 | 24.7090416 | 24.73293495 | 24.72826958 | 24.68126297 | 24.67373657 | 24.60800552 |
|  | 0.566026623 | 0.069259008 | Q7TNC4;E9Q715;Q05CX5 | Putative RNA-binding protein Luc7-like 2 | Luc7l2 | 22.93109894 | 23.0866375 | 22.99081993 | 22.99353409 | 22.90732765 | 22.8999176 |
|  | 0.443077781 | 0.069367091 | P70168 | Importin subunit beta-1 | Kpnb1 | 24.97289848 | 25.0484314 | 24.98050308 | 25.05680656 | 24.87135887 | 24.86556625 |
|  | 0.262540923 | 0.069719315 | P56391;A0A140LIU3 | Cytochrome c oxidase subunit 6B1 | Cox6b1 | 25.41502953 | 25.25551414 | 25.52874184 | 25.24898148 | 25.47001266 | 25.27113342 |
|  | 0.586603981 | 0.069864909 | P09405 | Nucleolin | Ncl | 25.3773613 | 25.26602936 | 25.41522408 | 25.22887039 | 25.29379272 | 25.32635689 |
|  | 1.847155482 | 0.069895426 | P05064;D3YWI1;D3Z510;A0A0U1RPN8 | Fructose-bisphosphate aldolase A;Fructose-bisphosphate aldolase | Aldoa | 29.53378487 | 29.50642776 | 29.48910904 | 29.43841171 | 29.45906448 | 29.42215919 |
|  | 0.185879331 | 0.070075353 | Q8BP92;D6RHL9 | Reticulocalbin-2 | Rcn2 | 22.39065933 | 22.76759338 | 22.33919334 | 22.34199142 | 22.51438904 | 22.43083954 |
|  | 0.471608498 | 0.070100149 | A0A0J9YTY0;A0A0J9YUL3;Q8C1B7;A0A0J9YUV6;A0A0J9YVA6;A2A3W1 | Septin-11 | Septin11 | 24.58617592 | 24.68287086 | 24.77836227 | 24.67044449 | 24.60941505 | 24.55724907 |
|  | 0.482041041 | 0.071264267 | Q9WUP7;A0A087WP81;A0A087WRL3 | Ubiquitin carboxyl-terminal hydrolase isozyme L5 | Uchl5 | 22.8616066 | 22.80607033 | 22.9917202 | 22.88134575 | 22.77715874 | 22.78709984 |
|  | 1.065064785 | 0.071447372 | P97429;A0A0N4SW89;D3Z0S1;A0A0N4SV57;F7ANV6;S4R1F2 | Annexin A4;Annexin | Anxa4 | 27.11574936 | 27.18602943 | 27.15672874 | 27.04092979 | 27.07907867 | 27.12415695 |
|  | 1.24624844 | 0.071834564 | Q9D8N0 | Elongation factor 1-gamma | Eef1g | 26.0577774 | 25.98489761 | 26.04458046 | 25.95349312 | 25.9851799 | 25.93307877 |
|  | 0.334720569 | 0.072202047 | Q9WUK2 | Eukaryotic translation initiation factor 4H | Eif4h | 25.24427032 | 25.35793304 | 25.15603256 | 25.23994446 | 25.04660225 | 25.25508308 |
|  | 0.794869869 | 0.072752635 | Q9JIF7 | Coatomer subunit beta | Copb1 | 24.82667732 | 24.80626678 | 24.91278267 | 24.78111839 | 24.72686768 | 24.8194828 |
|  | 0.265088117 | 0.0729599 | Q7TSV4;A0A0G2JG04;A0A0G2JF47 | Phosphoglucomutase-2 | Pgm2 | 23.51653099 | 23.74540138 | 23.73127747 | 23.59172058 | 23.73200226 | 23.4506073 |
|  | 0.544466037 | 0.073005676 | Q9QZQ8;Q8CCK0 | Core histone macro-H2A.1 | H2afy | 25.23596954 | 25.28506851 | 25.32378387 | 25.12091827 | 25.30566978 | 25.19921684 |
|  | 0.400981523 | 0.073055903 | Q5SUH7;Q5SUH6;Q99KN9 | Clathrin interactor 1 | Clint1 | 23.97771072 | 24.08990479 | 24.18305969 | 24.03783226 | 24.07879448 | 23.91488075 |
|  | 0.332682174 | 0.073478699 | A0A498WGK6;Q9ET30 | Transmembrane 9 superfamily member 3 | Tm9sf3 | 23.87427139 | 24.00855446 | 23.8338356 | 23.96331596 | 23.82629013 | 23.70661926 |
|  | 0.438360373 | 0.073806763 | A0A0G2JGQ4;P54729 | NEDD8 ultimate buster 1 | Nub1 | 23.24001694 | 23.14474487 | 23.0287571 | 23.13279533 | 22.99967194 | 23.05963135 |
|  | 0.664075662 | 0.074135462 | P99027;A0A5F8MPY2 | 60S acidic ribosomal protein P2 | Rplp2 | 27.52118301 | 27.59825325 | 27.52073288 | 27.40007019 | 27.55061531 | 27.46707726 |
|  | 0.811259621 | 0.074402491 | B2M1R6;H3BKD0;H3BKI8;Q8BT23;H3BLP7;A0A286YE41;H3BJ43;H3BJS9 |  | Hnrnpk | 26.15790367 | 26.07863045 | 26.03122139 | 25.97757912 | 26.04974174 | 26.01722717 |
|  | 0.223622676 | 0.074897766 | Q9CYN2;A0A140LJ01;A0A140LHG8;A0A140LHR3;A0A140LJG6;A0A140LIK0;A0A140LHW5 | Signal peptidase complex subunit 2 | Spcs2 | 24.9009037 | 24.68088913 | 25.10964584 | 24.86079216 | 24.86811256 | 24.73784065 |
|  | 0.701038646 | 0.075174967 | Q9JHK5;Q8CAG6;Q5F270 | Pleckstrin | Plek | 25.4952755 | 25.50233459 | 25.48888588 | 25.4255352 | 25.33340454 | 25.50203133 |
|  | 0.178716597 | 0.075205803 | Q8VD04;A2AEW9;A2AEW8;A2AEW6;A2AEW5 | GRIP1-associated protein 1 | Gripap1 | 22.06217003 | 22.02401352 | NaN | 21.74390221 | 22.00377655 | 22.15597916 |
|  | 0.213657814 | 0.075683594 | O88983;Q9D0J1;Q8BS59 | Syntaxin-8 | Stx8 | 22.89003944 | 22.96465683 | 22.86056328 | 22.6896801 | 23.09733582 | 22.70119286 |
|  | 0.51135251 | 0.075735092 | Q9JJU8 | SH3 domain-binding glutamic acid-rich-like protein | Sh3bgrl | 25.56978798 | 25.44871902 | 25.60608482 | 25.3788166 | 25.49612999 | 25.52243996 |
|  | 0.560094139 | 0.075839996 | O88685;A2AGN7;B7ZCF1 | 26S protease regulatory subunit 6A | Psmc3 | 24.42098236 | 24.39649773 | 24.30187225 | 24.34184265 | 24.20164871 | 24.34834099 |
|  | 0.522944497 | 0.07587115 | Q9R062;K3W4S6;V9GX26 | Glycogenin-1 | Gyg1;Gyg | 24.5469265 | 24.52516174 | 24.52939796 | 24.34773254 | 24.45870209 | 24.56743813 |
|  | 0.324241907 | 0.076000849 | Q9DAU1 | Protein canopy homolog 3 | Cnpy3 | 23.01335144 | 23.1972599 | 23.23513031 | 23.2010498 | 23.04505157 | 22.97163773 |
|  | 0.503085718 | 0.076065063 | Q9QUR6 | Prolyl endopeptidase | Prep | 24.33960152 | 24.27717781 | 24.3398056 | 24.35930824 | 24.14421654 | 24.22486496 |
|  | 0.335986378 | 0.076186498 | D3YZ86;A0A571BDP7;Q8VDV3;D3Z585 | Guanine nucleotide exchange factor for Rab-3A | Rab3il1 | 23.26313019 | 23.06012535 | 22.96100426 | 22.97744751 | 23.00316048 | 23.07509232 |
|  | 0.590673184 | 0.076190313 | Q9CQU0 | Thioredoxin domain-containing protein 12 | Txndc12 | 23.43948936 | 23.55051231 | 23.55344391 | 23.5135231 | 23.43936348 | 23.36198807 |
|  | 0.48246641 | 0.076998393 | P34022;H7BX22 | Ran-specific GTPase-activating protein | Ranbp1 | 25.72852898 | 25.73781586 | 25.79230881 | 25.61823654 | 25.80842972 | 25.6009922 |
|  | 1.319388351 | 0.07708931 | Q8QZT1 | Acetyl-CoA acetyltransferase, mitochondrial | Acat1 | 24.3601799 | 24.38291168 | 24.34075546 | 24.33039856 | 24.2469902 | 24.27519035 |
|  | 0.380864899 | 0.077123642 | Q91ZE0 | Trimethyllysine dioxygenase, mitochondrial | Tmlhe | 23.47233963 | 23.7644577 | 23.64482498 | 23.54274178 | 23.56465149 | 23.54285812 |
|  | 0.386025501 | 0.077355067 | G3XA66;Q8R5C0;A0A6I8MWZ8;P47802;G3XA75;D3YVW0;D3Z3F4;G3UYJ5;G3UXX9;G3UXB5 | Metaxin-1 | Mtx1 | 22.64739609 | 22.69308853 | 22.59217834 | NaN | 22.66340256 | 22.47032928 |
|  | 0.171736048 | 0.078542074 | A0A338P6G6;A0A384DV79;A0A338P6E8 | High mobility group AT-hook 1 | Hmga1 | 25.26977921 | 25.00962257 | 24.99632263 | 25.19786835 | 25.12234116 | 24.71988869 |
|  | 0.412691213 | 0.078779856 | Q6PGF7 | Exocyst complex component 8 | Exoc8 | 22.52599716 | 22.31324005 | 22.41580963 | 22.23356628 | 22.38958359 | 22.3955574 |
|  | 0.531285428 | 0.078875224 | O88668;K4DI63;J3QP41 | Protein CREG1 | Creg1 | 27.3731842 | 27.41239929 | 27.41997147 | 27.31406212 | 27.21724892 | 27.43761826 |
|  | 1.499054031 | 0.079123497 | Q9Z0H8 | CAP-Gly domain-containing linker protein 2 | Clip2 | 22.4568615 | 22.47281075 | 22.41941452 | NaN | 22.36575699 | 22.37538719 |
|  | 1.314778456 | 0.079784393 | P63323;F7AEH4;A0A1W2P7A1 | 40S ribosomal protein S12 | Rps12 | 26.65212059 | 26.65499115 | 26.6159668 | 26.56093979 | 26.60554886 | 26.51723671 |
|  | 0.238848343 | 0.079825083 | G3X928;Q6NZC7 | SEC23-interacting protein | Sec23ip | 22.72754288 | 22.84450531 | 22.80899811 | 22.60412979 | 22.5703373 | 22.96710396 |
|  | 1.208976931 | 0.079853694 | A2AVJ7;Q99PL5 | Ribosome-binding protein 1 | Rrbp1 | 26.03277779 | 25.97600555 | 25.99922943 | 25.9348793 | 25.96113205 | 25.87244034 |
|  | 0.437953709 | 0.07995669 | G3XA17;F7CBP1;F6TW20 | Eukaryotic translation initiation factor 4, gamma 2 | Eif4g2 | 23.07380295 | 23.21219826 | 23.09026146 | 23.17116547 | 22.95404243 | 23.01118469 |
|  | 0.388236916 | 0.079964956 | Q05D44 | Eukaryotic translation initiation factor 5B | Eif5b | 24.35917473 | 24.3212738 | 24.08358955 | 24.1633091 | 24.1666069 | 24.19422722 |
|  | 0.544657059 | 0.080231667 | Q80SY5 | Pre-mRNA-splicing factor 38B | Prpf38b | 19.24525642 | 19.34661293 | NaN | 19.23838997 | NaN | 19.19301605 |
|  | 0.251152007 | 0.0807031 | Q91WQ3;A2A7S7;F6VXZ2 | Tyrosine--tRNA ligase, cytoplasmic;Tyrosine--tRNA ligase, cytoplasmic, N-terminally processed;Tyrosine--tRNA ligase | Yars | 23.24759865 | 23.08401108 | 23.29684067 | 23.33197021 | 23.10009766 | 22.95427322 |
|  | 1.171606764 | 0.081505458 | A0A286YDF5;Q69ZN7;A0A286YCZ3;A0A286YE65;A0A286YDV5 | Myoferlin | Myof | 24.57473183 | 24.53118324 | 24.58422661 | 24.42527962 | 24.50742531 | 24.51292038 |
|  | 0.173951385 | 0.081748962 | A0A0U1RNT6;A0A0U1RNK6;Q3THS6;A0A0U1RQB0;A0A0U1RQ95 | S-adenosylmethionine synthase isoform type-2 | Mat2a | 23.41170502 | 23.43758583 | 23.94654846 | 23.44594383 | 23.57126427 | 23.53338432 |
|  | 0.64033368 | 0.082038244 | G8JL74;G3UXA6;Q8BHD7;G3UZ01;G3UY95;F7C521;G3UXZ8 | Polypyrimidine tract-binding protein 3 | Ptbp3 | 25.26176834 | 25.29379272 | 25.18929672 | 25.11473656 | 25.26391792 | 25.12008858 |
|  | 0.572025937 | 0.082153956 | Q91XH5;G3UXX3;Q64105;G3UZ79 | Sepiapterin reductase | Spr | 24.64641762 | 24.73205376 | 24.7635479 | 24.72821808 | 24.54356766 | 24.62377167 |
|  | 0.392828185 | 0.082236608 | Q8CEE7;A0A0R4J1N8;D3YVJ8 | Retinol dehydrogenase 13 | Rdh13 | 23.05470467 | 22.91261673 | 22.90431595 | 23.0222702 | 22.80443764 | 22.79821968 |
|  | 0.245022579 | 0.082988103 | B7ZC46;B1AQY9;B1AQZ0;Q8CHH9;E0CYM4 | Septin-8 | Septin8 | 24.50464058 | 24.28050995 | 24.16200256 | 24.17050743 | 24.12044334 | 24.40723801 |
|  | 0.994055517 | 0.083065033 | P07901;B7ZC50;A2A6A2;B7ZC49 | Heat shock protein HSP 90-alpha | Hsp90aa1 | 25.78907776 | 25.77160454 | 25.75964165 | 25.6449337 | 25.76638031 | 25.65981483 |
|  | 1.063437613 | 0.083174388 | Q9JKR6;A0A1L1SQ34;E0CYZ2;F6TRP3 | Hypoxia up-regulated protein 1 | Hyou1 | 25.25878906 | 25.28630257 | 25.19095421 | 25.17721558 | 25.11624527 | 25.19306183 |
|  | 0.48864844 | 0.083291372 | O35343;A0A0B4J1E7;D3YTN1 | Importin subunit alpha-3 | Kpna4 | 23.65785408 | 23.5400238 | 23.47333145 | 23.53148079 | 23.51773262 | 23.37212181 |
|  | 0.281969168 | 0.08388265 | E9Q9C3;Q9QZQ1;E9Q852;E9PYX7;F7C3I9;D3Z7L2;D3YUD2 | Afadin | Mllt4 | 22.64216042 | 22.76530838 | 22.64103508 | 22.80909729 | 22.42429352 | 22.56346512 |
|  | 0.839205475 | 0.084353129 | P35979 | 60S ribosomal protein L12 | Rpl12 | 26.44037819 | 26.50548744 | 26.47804642 | 26.47131729 | 26.32666588 | 26.37286949 |
|  | 0.453877654 | 0.084582647 | P70158;D3Z1B4 | Acid sphingomyelinase-like phosphodiesterase 3a | Smpdl3a | 25.06266403 | 25.12218285 | 25.04168129 | 24.99563217 | 24.85595322 | 25.12119484 |
|  | 0.391993673 | 0.084585508 | Q8C5R8 | ribose-phosphate diphosphokinase | Prps1l1 | NaN | 23.07478333 | 23.10959816 | 22.95475388 | 22.92717934 | 23.14088249 |
|  | 0.969558762 | 0.085524877 | P57759;F8WJI4;F8WIM7;D6RG87 | Endoplasmic reticulum resident protein 29 | Erp29 | 26.01041412 | 26.0680275 | 26.05318451 | 26.02282333 | 25.89279747 | 25.95943069 |
|  | 0.194867376 | 0.085950851 | P61021 | Ras-related protein Rab-5B | Rab5b | 24.28947258 | 24.36359215 | 24.44651222 | 23.99774361 | 24.28107452 | 24.56290627 |
|  | 0.286432268 | 0.085974058 | Q9D771 | Transmembrane protein 206 | Tmem206 | 23.21899605 | 23.24495888 | 23.38561249 | NaN | 23.32676888 | 23.06766129 |
|  | 0.774442274 | 0.086446126 | O09131;A0A494BAB1;A0A494B9X6;A0A494BAY2;A0A494BB82;Q8K2Q2 | Glutathione S-transferase omega-1 | Gsto1 | 25.45469093 | 25.48077583 | 25.429533 | 25.4127388 | 25.42271614 | 25.27020645 |
|  | 0.384083091 | 0.086995443 | Q9CXW3;A0A0A6YY29 | Calcyclin-binding protein | Cacybp | 23.70197487 | 23.69985962 | 23.69498062 | 23.58129883 | 23.79014587 | 23.46438408 |
|  | 0.335545065 | 0.08710289 | H7BX88;P47934;B7ZDD7;A2AWJ5;F7BF80 | Carnitine O-acetyltransferase | Crat | 22.08345985 | 21.85186195 | NaN | 21.92947578 | 21.79225349 | 21.91994476 |
|  | 0.273307064 | 0.087406158 | O55022 | Membrane-associated progesterone receptor component 1 | Pgrmc1 | 24.02267647 | 23.78925133 | 23.78526497 | 23.97893333 | 23.70419312 | 23.65184784 |
|  | 0.54948018 | 0.087515513 | Q64324;F8WGM5;A0A140LJ60 | Syntaxin-binding protein 2 | Stxbp2 | 23.91012955 | 23.89393234 | 23.70946121 | 23.75480843 | 23.79768562 | 23.69848251 |
|  | 0.268214899 | 0.087751389 | Q9D0M5;D6RIN4 | Dynein light chain 2, cytoplasmic | Dynll2 | 24.35763168 | 24.67868805 | 24.46730995 | 24.56261444 | 24.42867088 | 24.24909019 |
|  | 1.118885508 | 0.087851842 | Q3UJB0;A0A494B9S9 | Splicing factor 3b, subunit 2 | Sf3b2 | 23.3924427 | 23.32085991 | 23.32567024 | 23.30061531 | 23.27141953 | 23.20338249 |
|  | 1.77004083 | 0.087901433 | E9Q3X0;Q9EQK5;D3Z2N7 | Major vault protein | Mvp | 25.29991722 | 25.31582642 | 25.30723381 | 25.17679787 | 25.24807549 | 25.2343998 |
|  | 0.729193234 | 0.087937673 | H3BJU7;H3BJ45;H3BJ40;H3BKH9;H3BJX8;Q60875;H3BL15;H3BLF9;H3BJ59 | Rho guanine nucleotide exchange factor 2 | Arhgef2 | 23.54191399 | 23.42726326 | 23.54297638 | 23.3543396 | 23.40379715 | 23.49020386 |
|  | 0.403429765 | 0.087951024 | Q9JHS3;D3YTS4 | Ragulator complex protein LAMTOR2 | Lamtor2 | 24.24198151 | 24.45563316 | 24.39047623 | 24.32168579 | 24.22780609 | NaN |
|  | 0.411265132 | 0.088024775 | O55091;A0A3Q4EC12;A0A3Q4L314 | Protein IMPACT | Impact | 23.03241158 | 22.83851624 | 22.84118843 | 22.68733215 | 22.8860817 | 22.87462807 |
|  | 0.605115833 | 0.088214238 | P70441 | Na(+)/H(+) exchange regulatory cofactor NHE-RF1 | Slc9a3r1 | 24.84153366 | 24.8749733 | 24.80208206 | 24.63344955 | 24.8426857 | 24.77781105 |
|  | 0.20566816 | 0.088555018 | A2AN08;F6SSP6;Z4YMA7;Z4YLP1 | E3 ubiquitin-protein ligase UBR4 | Ubr4 | 22.8006897 | 22.70241928 | 23.08712387 | 22.92083549 | 22.53715706 | 22.86657524 |
|  | 0.619765509 | 0.08869489 | A0A0A0MQN4;Q9D906;S4R2D5;S4R2T5;S4R2P7;S4R1T0;S4R1N8;S4R1U3;S4R1W9;S4R1K1 | Ubiquitin-like modifier-activating enzyme ATG7 | Atg7 | 23.2482357 | 23.28291512 | 23.41660881 | 23.19746971 | 23.1801033 | 23.30410194 |
|  | 0.263051927 | 0.088750839 | A0A1B0GRT6;A0A1B0GRH2;P49446 | Receptor-type tyrosine-protein phosphatase epsilon | Ptpre | 22.20421982 | 21.91225243 | 22.36367226 | 22.02887535 | 22.06812096 | 22.11689568 |
|  | 0.867945314 | 0.089195887 | Q2TBE6;A0A494BBQ4 | Phosphatidylinositol 4-kinase type 2-alpha | Pi4k2a | 22.78801727 | 22.81428909 | 22.72602654 | 22.60788155 | 22.71548653 | 22.73737717 |
|  | 0.782474212 | 0.089551926 | P62334 | 26S protease regulatory subunit 10B | Psmc6 | 24.23000717 | 24.28263092 | 24.33353996 | 24.17721558 | 24.27433586 | 24.12597084 |
|  | 0.23655874 | 0.089658737 | Q3U816;Q9Z2G9 | Oxidoreductase HTATIP2 | Htatip2 | 23.07685471 | 22.59233665 | 22.90644646 | 22.84310722 | 22.68547249 | 22.77808189 |
|  | 0.114605994 | 0.08967495 | Q6NXL1 | Sec24 related gene family, member D (S. cerevisiae) | Sec24d | 21.92990875 | NaN | 22.47038078 | 22.20726013 | 22.30474281 | 21.81940651 |
|  | 0.921039192 | 0.089928945 | Q9QYB1 | Chloride intracellular channel protein 4 | Clic4 | 24.61172485 | 24.63034248 | 24.68570709 | 24.47753716 | 24.56726456 | 24.61318588 |
|  | 0.080718482 | 0.090251923 | Q9CQD1 | Ras-related protein Rab-5A | Rab5a | 23.5825634 | 23.12540245 | 24.12636566 | 23.65665436 | 23.00321198 | 23.90370941 |
|  | 0.965711746 | 0.090337118 | P60229 | Eukaryotic translation initiation factor 3 subunit E | Eif3e | 24.52258873 | 24.62176132 | 24.65080643 | 24.46743584 | 24.52707291 | 24.52963638 |
|  | 1.036386932 | 0.090431213 | P55302;F6WMD1 | Alpha-2-macroglobulin receptor-associated protein | Lrpap1 | 24.68602943 | 24.73799515 | 24.76819992 | 24.62137032 | 24.70493126 | 24.59462929 |
|  | 1.162162824 | 0.091534932 | P50580 | Proliferation-associated protein 2G4 | Pa2g4 | 25.77046967 | 25.78264236 | 25.75059891 | 25.72665977 | 25.60698891 | 25.69545746 |
|  | 0.356024575 | 0.092102687 | Q91V76;A0A1L1SSF8;A0A1L1ST86 | Ester hydrolase C11orf54 homolog |  | 22.98273277 | 23.12916756 | 23.21563911 | 23.13525581 | 23.06017494 | 22.85580063 |
|  | 0.372083918 | 0.092236837 | Q8VE96;A0A0G2JEG3;D6RHL1 | Solute carrier family 35 member F6 | Slc35f6 | 22.62685013 | 22.96859932 | 22.82613373 | 22.67643929 | 22.77617455 | 22.69225883 |
|  | 0.949517878 | 0.093118032 | Q9EP69;A0A5F8MPK9;A0A1L1SR59 | Phosphatidylinositide phosphatase SAC1 | Sacm1l | 24.21007347 | 24.25868034 | 24.18623734 | 24.20396423 | 24.10325623 | 24.0684166 |
|  | 0.261005015 | 0.093592962 | O35127 | Protein C10 | Grcc10 | 22.19084167 | 22.07613754 | 22.12410927 | NaN | 22.21377182 | 21.85976791 |
|  | 0.46762879 | 0.093647639 | Q8QZY1;A0A2R8VH97 | Eukaryotic translation initiation factor 3 subunit L | Eif3l | 24.72353935 | 24.52073288 | 24.552742 | 24.62421799 | 24.45299721 | 24.43885612 |
|  | 1.743915595 | 0.094132741 | E9Q616;A0A494BBD5;G5E8K8;A0A494B8Y7 |  | Ahnak | 28.2469902 | 28.27061653 | 28.27092934 | 28.15262413 | 28.21410179 | 28.13941193 |
|  | 0.248985018 | 0.094420751 | Q9Z2I8;A0A0N4SWD1 | Succinyl-CoA ligase [GDP-forming] subunit beta, mitochondrial | Suclg2 | 23.29473877 | 23.2465992 | 23.62142754 | 23.25753021 | 23.1524868 | 23.46948624 |
|  | 1.086696113 | 0.09457016 | Q9Z1T1;A0A338P6V5;A0A571BEW4;A0A571BES1;Q9JME5 | AP-3 complex subunit beta-1 | Ap3b1 | 24.72707558 | 24.73153687 | 24.75694656 | 24.5693531 | 24.705513 | 24.65698242 |
|  | 0.538022178 | 0.094866117 | P46061;A0A2R8W753;A0A2R8VHK9 | Ran GTPase-activating protein 1 | Rangap1 | 22.16112518 | 22.06779289 | 21.96599579 | NaN | 21.98801422 | 21.95219612 |
|  | 2.916088638 | 0.095092138 | Q68FD5;Q5SXR6;F6Z1R4 | Clathrin heavy chain 1;Clathrin heavy chain | Cltc | 27.21326637 | 27.23278999 | 27.20601654 | 27.11644363 | 27.13887596 | 27.1114769 |
|  | 2.230282491 | 0.096067429 | P38647 | Stress-70 protein, mitochondrial | Hspa9 | 26.50857353 | 26.52248573 | 26.46866226 | 26.41926384 | 26.39326096 | 26.39899445 |
|  | 0.621566621 | 0.096410116 | Q7TQI3;D3YWF6;D3Z7K0 | Ubiquitin thioesterase OTUB1 | Otub1 | 24.06931877 | 23.94046783 | 24.02968407 | 24.0287571 | 23.8894825 | 23.83200073 |
|  | 0.777782581 | 0.096499125 | Q9CPY7;A0A0G2JEM7 | Cytosol aminopeptidase | Lap3 | 23.74108505 | 23.67130852 | 23.59274864 | 23.54663086 | 23.52264977 | 23.64636421 |
|  | 0.716103031 | 0.096698125 | Q99MN1;Q8R2P8 | Lysine--tRNA ligase | Kars | 23.83373833 | 23.94931221 | 23.94368935 | 23.90545464 | 23.79133987 | 23.739851 |
|  | 0.698956268 | 0.096775055 | A0A286YCV9;E9Q4K7;A0A286YD28;A2AKH9 | Kinesin-like protein | Kif13b | 23.41519165 | 23.38929367 | 23.32704353 | 23.17308807 | 23.29852104 | 23.36959457 |
|  | 0.659073212 | 0.096874873 | Q8BH80;Q9QY76 | Vesicle-associated membrane protein-associated protein B | Vapb | 25.33156013 | 25.38558006 | 25.26753044 | 25.27820587 | 25.29820633 | 25.11763382 |
|  | 0.175245367 | 0.097026825 | O09126 | Semaphorin-4D | Sema4d | 22.82826614 | 22.21308899 | 22.45038033 | 22.44907188 | 22.19096184 | 22.56062126 |
|  | 2.38516622 | 0.097053528 | Q9Z204;A0A2I3BRM6;A0A2I3BQH3;A0A2I3BQW7 | Heterogeneous nuclear ribonucleoproteins C1/C2 | Hnrnpc | 24.7817173 | 24.79887199 | 24.75964165 | 24.65927124 | 24.69396973 | 24.69582939 |
|  | 0.438662041 | 0.097138723 | Q3TDN2;D3Z2H4;D3YUP4;D6RE51;D3Z4G9 | FAS-associated factor 2 | Faf2 | 22.87672615 | 22.70153046 | 23.00094414 | 22.69204521 | 22.77150726 | 22.8242321 |
|  | 1.217883335 | 0.097581228 | Q8K183 | Pyridoxal kinase | Pdxk | 26.34622765 | 26.28332138 | 26.26459885 | 26.16775513 | 26.17656898 | 26.25708008 |
|  | 0.734097221 | 0.097954432 | Q9D1G1;A0A494B945;A0A494BA38;A0A494BBL7 | Ras-related protein Rab-1B | Rab1b | 25.93494606 | 25.94026566 | 25.90517998 | 25.88741684 | 25.89059639 | 25.70851517 |
|  | 0.820122958 | 0.098066966 | A0A0R4J052;G5E8T9;Q99KB8;E9PYA3;E9Q2H8;D3YUX8;D3YWI0 | Hydroxyacylglutathione hydrolase, mitochondrial | Hagh | 22.78416634 | 22.84504128 | 22.9180336 | 22.69361877 | 22.73239517 | 22.82702637 |
|  | 0.71438638 | 0.098796844 | D3Z6S1;Q8BM55 | Transmembrane protein 214 | Tmem214 | 22.48927307 | 22.37785149 | 22.57049942 | 22.39694214 | 22.32292366 | 22.42136765 |
|  | 0.341119993 | 0.098857244 | Q8CI11;A0A2I3BRV9;A0A2I3BPZ6;A0A2I3BR82;A0A2I3BR32 | Guanine nucleotide-binding protein-like 3 | Gnl3 | NaN | 22.16937637 | 22.36067581 | 22.09464836 | 22.09638786 | 22.30747032 |
|  | 0.713965236 | 0.100434621 | Q7TPV4 | Myb-binding protein 1A | Mybbp1a | 23.87549019 | 23.98797798 | 24.05573082 | 23.86627388 | 23.81196022 | 23.93966103 |
|  | 0.390137436 | 0.100493749 | A0A0R4J1Z3;Q9CR67;Q9CZM3;E0CXY5;A0A0J9YV78 | Transmembrane protein 33 | Tmem33 | 23.75857544 | 23.73262405 | 23.42649651 | 23.52097321 | 23.51520729 | 23.58003426 |
|  | 0.197324408 | 0.100678762 | Q8VHM5;F7B5B5;A2AW41 | Heterogeneous nuclear ribonucleoprotein R | Hnrnpr | 23.02077866 | 23.41183281 | 23.47333145 | 23.41531944 | 22.94988251 | 23.23870468 |
|  | 0.933017306 | 0.100851059 | Q11136;G3UXC5 | Xaa-Pro dipeptidase | Pepd | 25.04676819 | 24.96552086 | 24.91145706 | 24.92560577 | 24.87946892 | 24.81611824 |
|  | 1.14753765 | 0.100914637 | P40124;B1ARS0;D3YTR7;A0A286YCS6;Q9CYT6 | Adenylyl cyclase-associated protein 1 | Cap1 | 28.7514286 | 28.82699203 | 28.73818207 | 28.65509224 | 28.73075294 | 28.62801361 |
|  | 0.718293549 | 0.101249059 | Q9CZ30;B1AYJ9 | Obg-like ATPase 1 | Ola1 | 24.07201958 | 24.09281158 | 23.89504242 | 23.94243813 | 23.921978 | 23.89171028 |
|  | 0.699809519 | 0.101733526 | Q9CR98;A0A0N4SWI4 | Protein FAM136A | Fam136a | 22.61665726 | 22.53169632 | 22.64304161 | 22.60790443 | 22.45651054 | 22.42177963 |
|  | 0.407572123 | 0.102079391 | Q6W4W7;O70566;E9Q4U7 | Protein diaphanous homolog 2 | Diap2;Diaph2 | 22.6135807 | 22.81401634 | 22.80968666 | 22.53692055 | 22.80766296 | 22.58646202 |
|  | 0.865386808 | 0.102132797 | P63101;A0A2I3BQ03;D3YXN6;D3YXF4;D3YW45 | 14-3-3 protein zeta/delta | Ywhaz | 28.05356789 | 28.17735863 | 28.07423782 | 27.92093468 | 28.04092979 | 28.03690147 |
|  | 1.062596512 | 0.103366852 | Q61599;D3YWL7;A0A0N4SVH4 | Rho GDP-dissociation inhibitor 2 | Arhgdib | 27.42342186 | 27.48523712 | 27.47738266 | 27.31440926 | 27.32018852 | 27.44134331 |
|  | 0.244691946 | 0.1038119 | Q9JHW2;A0A338P6G0;A0A338P7A1;A0A338P6Z4 | Omega-amidase NIT2 | Nit2 | 23.1687336 | 23.03318596 | 22.92391968 | 23.05578041 | 23.12196159 | 22.63666153 |
|  | 0.716744732 | 0.104273478 | Q8C129 | Leucyl-cystinyl aminopeptidase | Lnpep | 24.19550514 | 24.07283783 | 24.06291199 | 23.90407753 | 24.0560627 | 24.0582943 |
|  | 0.683509973 | 0.104368846 | Q8BH95 | Enoyl-CoA hydratase, mitochondrial | Echs1 | 24.77876282 | 24.94730759 | 24.85133743 | 24.65605545 | 24.80715179 | 24.80109406 |
|  | 0.871219802 | 0.10441939 | Q9CX00 | IST1 homolog | Ist1 | 23.10653496 | 23.20775223 | 23.07713127 | 23.03219223 | NaN | 23.01991463 |
|  | 0.577331086 | 0.105172475 | P20491 | High affinity immunoglobulin epsilon receptor subunit gamma | Fcer1g | 25.969347 | 26.06806755 | 26.12880516 | 26.07510567 | 25.92755127 | 25.84804535 |
|  | 1.314762643 | 0.105228424 | B9EJ86;A0A0R4J150;A0A5F8MPC5;G5E833;Q9ER64;D3YWU9 | Oxysterol-binding protein | Osbpl8 | 24.90145493 | 24.97460747 | 24.96168327 | 24.83605194 | 24.89471817 | 24.79129028 |
|  | 0.372764144 | 0.105291367 | O55142 | 60S ribosomal protein L35a | Rpl35a | 25.34719276 | 25.28478622 | 25.54760361 | 25.12324905 | 25.42335701 | 25.31710243 |
|  | 0.240929609 | 0.105427424 | P22437 | Prostaglandin G/H synthase 1 | Ptgs1 | 23.16186333 | 23.68088913 | 23.48246956 | 23.22208214 | 23.49801826 | 23.28883934 |
|  | 2.584505817 | 0.105854034 | O70404;A0A0R4J0R1;A0A0U1RPE8 | Vesicle-associated membrane protein 8 | Vamp8 | 25.16180992 | 25.11171722 | 25.15611076 | 25.03728867 | 25.0372467 | 25.03754044 |
|  | 0.298519624 | 0.10631752 | P26645 | Myristoylated alanine-rich C-kinase substrate | Marcks | 25.14619827 | 25.30480003 | 25.00821304 | 24.85314751 | 25.2556591 | 25.03145218 |
|  | 0.344523549 | 0.106679281 | Q8K2Q0 | COMM domain-containing protein 9 | Commd9 | 23.58703423 | 23.3614521 | 23.77906418 | 23.46948624 | 23.54427528 | 23.39375114 |
|  | 0.625190987 | 0.10678037 | A0A0G2JEA9;A0A0G2JFX7;Q9CWZ3;A0A0N4SUH6 | RNA-binding protein 8A | Rbm8a | 22.85694122 | 22.8500309 | 22.89480209 | 22.85289001 | 22.81786537 | 22.61067772 |
|  | 0.918596985 | 0.107183456 | P97370 | Sodium/potassium-transporting ATPase subunit beta-3 | Atp1b3 | 25.11076164 | 24.99899292 | 24.92700768 | 24.92401886 | 24.88739204 | 24.90380096 |
|  | 1.169199586 | 0.107808431 | E9Q855;Q3UXS0;O35609 | Secretory carrier-associated membrane protein 3 | Scamp3 | 24.75154305 | 24.72228813 | 24.76627922 | 24.71612549 | 24.62605858 | 24.57450104 |
|  | 0.705083648 | 0.107932409 | Q9QXX4 | Calcium-binding mitochondrial carrier protein Aralar2 | Slc25a13 | 23.30563354 | 23.35164642 | 23.32759094 | 23.30591202 | 23.08474159 | 23.27042007 |
|  | 0.796044482 | 0.107950211 | Q543K9;P23492;A0A2I3BQH2;Q9D8C9;A0A2I3BS22 | Purine nucleoside phosphorylase | Pnp | 26.28268433 | 26.31103325 | 26.38042068 | 26.32452202 | 26.13975334 | 26.18601227 |
|  | 1.045777605 | 0.108008067 | P63085;A0A338P781;A0A338P736;E9PXX5;E9Q3I6;Q6P5G0;Q61532 | Mitogen-activated protein kinase 1 | Mapk1 | 24.84244537 | 24.81787491 | 24.84139061 | 24.7424221 | 24.79926682 | 24.63599777 |
|  | 1.054022629 | 0.10807991 | Q9DB20;A0A338P7G3;A0A338P776;F7D3P8;F6XVM5 | ATP synthase subunit O, mitochondrial | Atp5o | 25.99658012 | 25.92415619 | 26.03938103 | 25.9005127 | 25.92436028 | 25.81100464 |
|  | 1.699445114 | 0.108673096 | P14869;S4R1N1;D3YVM5 | 60S acidic ribosomal protein P0 | Rplp0 | 27.40551758 | 27.39468384 | 27.44561195 | 27.30817223 | 27.3482399 | 27.26338196 |
|  | 1.113336387 | 0.10918808 | D6RHA2;A0A8Z1SL50;P82343;A0A8Z1S8W2;G3XA46;A0A0E2WEF3 | N-acylglucosamine 2-epimerase | Renbp | 25.66690063 | 25.72301865 | 25.73562241 | 25.68040466 | 25.56938171 | 25.54819107 |
|  | 0.326703592 | 0.109576543 | Q9EPE9 | Manganese-transporting ATPase 13A1 | Atp13a1 | 23.06956291 | 22.8038063 | 23.19266319 | 22.79316711 | 22.88914871 | 23.05498695 |
|  | 0.724349803 | 0.110018412 | A0A0R4J2B0;V9GX43;S4R2U7;S4R1S7;E9QA47;Q9Z0H4;S4R2L5;S4R2J2;A3KGT0;S4R2U1 | CUGBP Elav-like family member 2 | Celf2 | 23.52802658 | 23.30688667 | 23.39047623 | 23.30688667 | 23.24979782 | 23.33864975 |
|  | 0.263385512 | 0.110019684 | O88842;Q3TNB8;A0A0R4J1D9 | FYVE, RhoGEF and PH domain-containing protein 3 | Fgd3 | 23.55894279 | 23.04159737 | 23.23915672 | 23.15952492 | 23.05215263 | 23.29796028 |
|  | 1.432018082 | 0.110397975 | Q9R1P3 | Proteasome subunit beta type-2 | Psmb2 | 24.98758888 | 24.95371437 | 24.92433739 | 24.82289124 | 24.80582428 | 24.9057312 |
|  | 0.778593584 | 0.110644658 | Q9DCS3;A2A845 | Trans-2-enoyl-CoA reductase, mitochondrial | Mecr | 23.1811657 | 23.06394768 | 23.20927048 | 22.97636414 | 23.01125336 | 23.13483238 |
|  | 0.762576602 | 0.111666361 | Q8BFZ9;A0A1B0GSD8;A0A1B0GT70;A0A1B0GQZ0;A0A1B0GT43;A0A1B0GRQ1;A0A1B0GRG7;E9PUH5 | Erlin-2 | Erlin2 | 23.60986519 | 23.57126427 | 23.39414406 | 23.42354965 | 23.42585564 | 23.39086914 |
|  | 0.231843824 | 0.111731211 | Q9QYS9;A0A3B2WCH2 | Protein quaking | Qki | 22.96089745 | 22.97435379 | 23.52993393 | 23.01619339 | 23.02140617 | 23.09239197 |
|  | 0.488843191 | 0.111800512 | Q3ULB1;Q921W7;P47226;D6RH72;B1AXB9 | Testin | Tes | 23.52240944 | 23.58371162 | 23.33128738 | 23.2389679 | 23.43961716 | 23.42342186 |
|  | 0.780396076 | 0.11199824 | Q9D0T1 | NHP2-like protein 1;NHP2-like protein 1, N-terminally processed | Nhp2l1 | 24.06496811 | 24.21637917 | 24.14903259 | 24.11378479 | 23.94234848 | 24.03825188 |
|  | 0.450719884 | 0.112163544 | E9Q912;A0A0G2JGC8;A0A0G2JF70 |  | Rap1gds1 | 23.1237793 | 22.79022598 | 23.06360245 | 22.81928825 | 22.91674042 | 22.90508842 |
|  | 0.965702052 | 0.112632751 | Q9DCH4 | Eukaryotic translation initiation factor 3 subunit F | Eif3f | 24.19565392 | 24.1649971 | 24.02115059 | 24.02732468 | 24.0205574 | 23.99602127 |
|  | 0.812466589 | 0.112716039 | Q8BGS2 | BolA-like protein 2 | Bola2 | 23.70535469 | 23.50008774 | 23.5926342 | 23.44126511 | 23.49338341 | 23.52528 |
|  | 0.483199039 | 0.113039653 | B1AVH5;Q8C0P5 | Coronin;Coronin-2A | Coro2a | 22.62780762 | 22.7776413 | 22.43771362 | 22.50237083 | 22.54568863 | 22.45598412 |
|  | 0.69433329 | 0.113076528 | P54822;E9Q242;E9Q3T7;E9Q0A0;A0A140LHU1;A0A0G2JFI8;A0A0G2JFL8 | Adenylosuccinate lyase | Adsl | 23.81626511 | 23.9269619 | 24.0284214 | 23.74642563 | 23.79669571 | 23.88929749 |
|  | 0.511271174 | 0.113917669 | Q8R1Q8 | Cytoplasmic dynein 1 light intermediate chain 1 | Dync1li1 | 24.26585007 | 24.11314774 | 24.23425293 | 24.14024162 | 23.92333984 | 24.20791626 |
|  | 1.89806557 | 0.114298503 | P05202 | Aspartate aminotransferase, mitochondrial | Got2 | 26.61680794 | 26.53931236 | 26.58118439 | 26.44238853 | 26.46057701 | 26.49144363 |
|  | 0.998267781 | 0.114355723 | Q9DAS9;A0A0N4SW28;A0A0N4SVT3 | Guanine nucleotide-binding protein G(I)/G(S)/G(O) subunit gamma-12 | Gng12 | 25.22254562 | 25.07189751 | 25.12119484 | 25.0845623 | 24.98871613 | 24.99929237 |
|  | 0.861472596 | 0.114476522 | D3Z7C6;Q9R0Q7 | Prostaglandin E synthase 3 | Ptges3 | 25.73858833 | 25.70806694 | 25.64364243 | 25.53225517 | 25.69232178 | 25.52229118 |
|  | 0.365638218 | 0.114481608 | Q62348 | Translin | Tsn | 24.05191994 | 24.04776764 | 23.70123482 | 23.89226532 | 23.70007133 | 23.86514091 |
|  | 0.609849668 | 0.11511294 | Q9JKB1;A0A2I3BQ39;P58321 | Ubiquitin carboxyl-terminal hydrolase isozyme L3;Ubiquitin carboxyl-terminal hydrolase isozyme L4 | Uchl3;Uchl4 | 24.20082664 | 24.2849617 | 24.45475388 | 24.12880516 | 24.26699448 | 24.19940376 |
|  | 0.212909802 | 0.116018295 | Q8C2E7 | WASH complex subunit strumpellin | Kiaa0196 | 23.56685829 | 23.26999283 | 23.60184288 | 23.6568737 | 23.02607536 | 23.40769005 |
|  | 0.513027377 | 0.116134008 | Q60631;B1AT92;B1AT95 | Growth factor receptor-bound protein 2 | Grb2 | 25.17546654 | 25.00139999 | 25.1222229 | 24.81806946 | 25.09872627 | 25.03389168 |
|  | 0.954023463 | 0.116785049 | Q9JMG1 | Endothelial differentiation-related factor 1 | Edf1 | 22.83265686 | 22.84791183 | 22.85071754 | 22.66895294 | 22.67096329 | 22.84101486 |
|  | 0.632804445 | 0.116804123 | O55106;F8WH41;F6Z700 | Striatin | Strn | 22.8764267 | 22.81411362 | 22.86697197 | 22.85117531 | 22.57984924 | 22.77607536 |
|  | 2.075951128 | 0.116839727 | P51150;A0A0N4SVG9;A0A0N4SVR6 | Ras-related protein Rab-7a | Rab7a | 26.83809853 | 26.89642906 | 26.85721207 | 26.77089882 | 26.71423912 | 26.75608253 |
|  | 0.886232905 | 0.116936366 | H7BX01;P58281;E0CXD1;F6U775;P58682 | Dynamin-like 120 kDa protein, mitochondrial;Dynamin-like 120 kDa protein, form S1 | Opa1 | 23.22618866 | 23.28573799 | 23.17557335 | 23.20127487 | 23.11621475 | 23.01920128 |
|  | 1.75640995 | 0.117252986 | Q3T9X3;P39054;F8WIV5;G3UZZ3 | Dynamin-2 | Dnm2 | 24.35561752 | 24.31527328 | 24.35810089 | 24.27646828 | 24.18616295 | 24.21460152 |
|  | 0.798246677 | 0.118111293 | Q91VC3;A0A0N4SVP8;E9PV04;A2AFK7;A0A0N4SVM5;D6RJ11 | Eukaryotic initiation factor 4A-III;Eukaryotic initiation factor 4A-III, N-terminally processed | Eif4a3;Gm8994 | 24.05994415 | 24.23717499 | 24.0858593 | 24.03347015 | 24.06414604 | 23.93102837 |
|  | 1.650612241 | 0.118218104 | Q91YI0;E0CY49;F7D439;E0CYV3 | Argininosuccinate lyase | Asl | 24.20060158 | 24.13367271 | 24.10836983 | 24.01954079 | 24.00496101 | 24.06348801 |
|  | 1.683488105 | 0.118288676 | P68373;Q9JJZ2 | Tubulin alpha-1C chain | Tuba1c | 29.01113319 | 28.98340225 | 28.992939 | 28.89769936 | 28.81692886 | 28.91798019 |
|  | 0.35238475 | 0.118595123 | Q9D1M4 | Eukaryotic translation elongation factor 1 epsilon-1 | Eef1e1 | 22.82378387 | 22.97363472 | 23.14354706 | 23.05981255 | 22.82320213 | 22.7021656 |
|  | 1.161957086 | 0.119099935 | G3UVV4;P17710;D3YYR4;D3Z365;D3Z105;B4YB29 | Hexokinase;Hexokinase-1 | Hk1 | 25.33956718 | 25.46454048 | 25.43342209 | 25.31222916 | 25.23428917 | 25.33371162 |
|  | 0.601249399 | 0.119510015 | Q9WUM5;A0A0N4SVU4 | Succinyl-CoA ligase [ADP/GDP-forming] subunit alpha, mitochondrial | Suclg1 | 24.60450363 | 24.60138893 | 24.7577095 | 24.48314667 | 24.67825699 | 24.44366837 |
|  | 0.805409885 | 0.119888941 | D3Z645;Q9QZ88;D3YYD5;D3YW98 | Vacuolar protein sorting-associated protein 29 | Vps29 | 25.61969376 | 25.51168442 | 25.3867321 | 25.37620163 | 25.36769676 | 25.41454506 |
|  | 0.597073592 | 0.120328267 | Q99NB9;G5E866;A0A087WNS2 | Splicing factor 3B subunit 1 | Sf3b1 | 23.07748985 | 23.28742981 | 23.14617538 | 23.11437225 | 23.11697578 | 22.91876221 |
|  | 0.474155205 | 0.120462418 | Q06185;Q8BTB6 | ATP synthase subunit e, mitochondrial | Atp5i;Atp5k | 25.53649902 | 25.82885742 | 25.57735634 | 25.63209152 | 25.5300827 | 25.41915131 |
|  | 0.738475433 | 0.120605469 | O35344 | Importin subunit alpha-4 | Kpna3 | 23.17464256 | 23.40769005 | 23.36305809 | 23.16235542 | 23.18321228 | 23.23800659 |
|  | 0.642980688 | 0.12067159 | Q91VH2 | Sorting nexin-9 | Snx9 | 23.21443748 | 23.12068176 | 23.02889252 | 23.03805161 | 22.87295723 | 23.09098816 |
|  | 0.421422382 | 0.121121724 | Q61191;B1AUX2;F6SJS2 | Host cell factor 1;HCF N-terminal chain 1;HCF N-terminal chain 2;HCF N-terminal chain 3;HCF N-terminal chain 4;HCF N-terminal chain 5;HCF N-terminal chain 6;HCF C-terminal chain 1;HCF C-terminal chain 2;HCF C-terminal chain 3;HCF C-terminal chain 4;HCF C-terminal chain 5;HCF C-terminal chain 6 | Hcfc1 | 22.92176056 | 23.07051468 | 22.67476082 | 22.76809883 | 22.83918953 | 22.69638252 |
|  | 1.154590454 | 0.121278763 | O08547;E9Q6R3;A0A0G2JF08;D6RES2 | Vesicle-trafficking protein SEC22b | Sec22b | 24.23330307 | 24.2311058 | 24.16538048 | 24.14693642 | 24.11680031 | 24.00221634 |
|  | 0.409196461 | 0.12134997 | P70303;A2AEQ5 | CTP synthase 2 | Ctps2 | 23.13955498 | 23.17350006 | 23.16524124 | 22.80319595 | 23.07887459 | 23.23217583 |
|  | 1.084555474 | 0.121660233 | A0A498WGK2;Q8BP47;A0A494BAX5;A0A494BB89;A0A494B927;A0A494BAW6 | Asparagine--tRNA ligase, cytoplasmic | Nars | 24.61414146 | 24.53219604 | 24.61694717 | 24.45801353 | 24.39283562 | 24.54745483 |
|  | 1.719012919 | 0.121717453 | P17742;A0A1L1SST0;A0A1L1SRX5 | Peptidyl-prolyl cis-trans isomerase A;Peptidyl-prolyl cis-trans isomerase A, N-terminally processed | Ppia | 30.07559013 | 30.01942825 | 29.96788025 | 29.89879417 | 29.88624382 | 29.91270828 |
|  | 0.201688663 | 0.122150103 | Q8R5L3 | Vam6/Vps39-like protein | Vps39 | NaN | 22.56351089 | 22.84404564 | 22.50266266 | 22.35887909 | 22.88334274 |
|  | 0.517292639 | 0.122213364 | Q9D6Y7;A0A1B0GT40 | Mitochondrial peptide methionine sulfoxide reductase | Msra | 23.09060097 | NaN | 22.96650696 | NaN | 22.97032166 | 22.84235954 |
|  | 1.392437387 | 0.122244517 | Q9Z2U1;A0A0G2JF97 | Proteasome subunit alpha type-5 | Psma5 | 25.83841133 | 25.92082024 | 25.81882668 | 25.68832588 | 25.74375916 | 25.77923965 |
|  | 0.171104375 | 0.122702916 | P85094 | Isochorismatase domain-containing protein 2A, mitochondrial | Isoc2a | 23.50045204 | 23.55555153 | 23.69072533 | 23.71732903 | 22.92918587 | 23.73210526 |
|  | 0.309275739 | 0.122752508 | Q9QYA2;G3UY77 | Mitochondrial import receptor subunit TOM40 homolog | Tomm40 | 23.61492729 | 23.74334717 | 23.6521759 | 23.86135864 | 23.37145805 | 23.40937614 |
|  | 1.153303639 | 0.122838974 | P40336;A0A1W2P7Z9;A0A1W2P7R7 | Vacuolar protein sorting-associated protein 26A | Vps26a | 24.9810257 | 25.04606056 | 25.13273239 | 24.94940186 | 24.88269043 | 24.95920944 |
|  | 0.326976685 | 0.122926712 | Q3B7Z2;Q5QNQ6;Q5F209;Q8K0C7;Q5QNQ4 | Oxysterol-binding protein 1 | Osbp | 23.22143173 | 22.98203468 | 23.11087227 | 23.09844398 | 23.14066315 | 22.70645142 |
|  | 0.265970718 | 0.12365977 | A2ADY9 | Protein DDI1 homolog 2 | Ddi2 | 23.8358593 | 23.83595657 | 23.53278923 | 23.92279625 | 23.44632149 | 23.46450806 |
|  | 0.713754103 | 0.123881658 | Q9CPQ1 | Cytochrome c oxidase subunit 6C | Cox6c | 25.79808044 | 25.75315094 | 25.60484314 | 25.67411423 | 25.61781693 | 25.4924984 |
|  | 2.101995104 | 0.124053319 | Q3U4W8;P56399;D3YYA5;D3Z4K7 | Ubiquitin carboxyl-terminal hydrolase;Ubiquitin carboxyl-terminal hydrolase 5 | Usp5 | 24.57074356 | 24.6132431 | 24.6163311 | 24.43993378 | 24.47747612 | 24.51074791 |
|  | 2.146419678 | 0.124994914 | P62259;D6REF3;F6WA09 | 14-3-3 protein epsilon | Ywhae | 27.26517105 | 27.24308968 | 27.28700829 | 27.145401 | 27.17378998 | 27.10109329 |
|  | 1.763880617 | 0.125069936 | A0A5F8MPS7;P24638;B7ZCF5;B7ZCF4 | Lysosomal acid phosphatase | Acp2 | 24.03069496 | 24.00290298 | 23.97079468 | 23.84661102 | 23.92985344 | 23.85271835 |
|  | 1.614450999 | 0.12528801 | Q9WV54;D3Z505 | Acid ceramidase;Acid ceramidase subunit alpha;Acid ceramidase subunit beta | Asah1 | 25.46294975 | 25.39237595 | 25.4996624 | 25.31913757 | 25.30218697 | 25.35779953 |
|  | 0.382288892 | 0.125858943 | Q14CH1 | Molybdenum cofactor sulfurase | Mocos | 22.53775024 | 22.35113525 | 22.22852516 | 22.14008522 | 22.45713615 | 22.14261246 |
|  | 1.237455018 | 0.125979106 | Q8BH59;V9GXX9 | Calcium-binding mitochondrial carrier protein Aralar1 | Slc25a12 | 24.0146122 | 23.90343475 | 24.02462196 | 23.89513588 | 23.86825371 | 23.80134201 |
|  | 0.469409714 | 0.126714071 | E9Q2M9;E9PV60 | WD repeat- and FYVE domain-containing protein 4 | Wdfy4 | 22.42884827 | 22.42270279 | 22.20883942 | 22.3954792 | 22.20514488 | 22.07962418 |
|  | 0.668218521 | 0.126885096 | O89079;D3Z315;F6YFR7;E9Q6I5;F6XIG5 | Coatomer subunit epsilon | Cope | 24.61605072 | 24.50427628 | 24.58124161 | 24.55800819 | 24.28876877 | 24.47413635 |
|  | 0.370085029 | 0.127230326 | P27601;Q9D034 | Guanine nucleotide-binding protein subunit alpha-13 | Gna13 | 23.75358391 | 23.45022964 | 23.2844677 | 23.28630257 | 23.43007469 | 23.39021301 |
|  | 0.791017687 | 0.127597809 | Q9D892 | Inosine triphosphate pyrophosphatase | Itpa | 23.65184784 | 23.4836998 | 23.42034149 | 23.4379673 | 23.34163857 | 23.39348984 |
|  | 0.992571051 | 0.12762324 | Q8BKC5 | Importin-5 | Ipo5 | 24.3554821 | 24.39080429 | 24.46905136 | 24.17850876 | 24.34041595 | 24.31354332 |
|  | 0.870576418 | 0.12769254 | A0A087WQE6;P83940;A0A087WNT1;A0A087WPE4 | Transcription elongation factor B polypeptide 1 | Tceb1 | 24.79624939 | 24.92175102 | 24.7165966 | 24.66112137 | 24.7489872 | 24.64141083 |
|  | 0.380261336 | 0.127707163 | O54962 | Barrier-to-autointegration factor;Barrier-to-autointegration factor, N-terminally processed | Banf1 | 24.22714424 | 24.40931129 | 24.33183289 | 24.21400833 | 23.95951843 | 24.41164017 |
|  | 0.882618559 | 0.128038406 | Q8K003 | Translation machinery-associated protein 7 | Tma7 | 23.34732819 | 23.42867088 | 23.44758415 | 23.15975571 | 23.33279037 | 23.34692192 |
|  | 0.252244087 | 0.1280454 | O08915;D3YW40 | AH receptor-interacting protein | Aip | 22.64037323 | 22.85713196 | 22.46513176 | NaN | 22.35205078 | 22.70028305 |
|  | 0.224377585 | 0.128862381 | Q9JK23;A0A3B2W3V4;A0A3B2WB82 | Proteasome assembly chaperone 1 | Psmg1 | 22.28491974 | NaN | 21.74951935 | 21.98901939 | 21.91877937 | 21.75727272 |
|  | 0.217393435 | 0.128968557 | G3X956;Q920B9 | FACT complex subunit SPT16 | Supt16;Supt16h | 22.45971489 | 22.74042702 | 22.34378052 | 22.02005005 | 22.4342308 | 22.7027359 |
|  | 0.172815061 | 0.129524231 | Q9R0Q3;F6V6T4;Q8BPI2 | Transmembrane emp24 domain-containing protein 2 | Tmed2 | 25.542593 | 24.88627625 | 24.77655602 | 24.97941208 | 25.18078613 | 24.65665436 |
|  | 0.522206983 | 0.129688263 | P97287 | Induced myeloid leukemia cell differentiation protein Mcl-1 homolog | Mcl1 | 22.7694912 | 22.51544952 | 22.64713287 | 22.64341545 | 22.36554337 | 22.53404999 |
|  | 0.392315015 | 0.130382856 | Z4YJY0;Q3TCJ1;D3Z4D8;D6RJ39 | BRISC complex subunit Abro1 | Fam175b | 23.05758286 | 22.78733826 | 22.72494507 | 22.76516724 | 22.68731117 | NaN |
|  | 0.62850597 | 0.130787532 | Q9CPQ8 | ATP synthase subunit g, mitochondrial | Atp5l | 25.35826874 | 25.55835724 | 25.32141113 | 25.29445839 | 25.37540817 | 25.17580795 |
|  | 1.699424512 | 0.130820592 | O88456;A0A0R4J1C2;Q9D7J7 | Calpain small subunit 1 | Capns1 | 25.17668343 | 25.23231506 | 25.21278381 | 25.01576042 | 25.11660194 | 25.09695816 |
|  | 2.88647482 | 0.131518046 | Q9JKF1;A0A0U1RNG5;A0A0U1RPU3;Q3UQP1;A0A0U1RPI2 | Ras GTPase-activating-like protein IQGAP1 | Iqgap1 | 26.81347847 | 26.79859924 | 26.83496857 | 26.67935944 | 26.66532898 | 26.70780373 |
|  | 2.811093919 | 0.131571452 | Q9D1A2;A0A494B9S3;A0A494B9U6 | Cytosolic non-specific dipeptidase | Cndp2 | 27.74823189 | 27.72377396 | 27.74919128 | 27.60823059 | 27.58319473 | 27.63505745 |
|  | 0.945458553 | 0.131814957 | Q99LP6 | GrpE protein homolog 1, mitochondrial | Grpel1 | 24.89258957 | 24.82468796 | 24.72764778 | 24.59894943 | 24.74919128 | 24.70133972 |
|  | 1.188636327 | 0.13189888 | A0A0A6YX18;Q8BVE3;A0A0A6YWP6;A0A0A6YVU0;A0A0A6YW86 | V-type proton ATPase subunit H | Atp6v1h | 25.96807098 | 25.87009048 | 25.97771072 | 25.83740044 | 25.85393333 | 25.72884178 |
|  | 2.017220882 | 0.131970088 | Q99KI0;A0A2R8W744;A0A2R8VHM8;A0A2R8VJW0 | Aconitate hydratase, mitochondrial | Aco2 | 25.53204727 | 25.56964302 | 25.51749229 | 25.42943573 | 25.43342209 | 25.36041451 |
|  | 0.640928571 | 0.132319768 | Q61263;A0A087WNN8;A0A087WSJ5 | Sterol O-acyltransferase 1 | Soat1 | 24.60969734 | 24.60614204 | 24.85495567 | 24.64377975 | 24.50185013 | 24.52820587 |
|  | 0.687418937 | 0.132623037 | Q80W54;I7HIP5 | CAAX prenyl protease 1 homolog | Zmpste24 | 23.86523628 | 24.04626846 | 23.81362534 | 23.8429718 | 23.67281914 | 23.81147003 |
|  | 1.175889725 | 0.133286158 | O88531;B1B0P8;B1B0P9 | Palmitoyl-protein thioesterase 1 | Ppt1 | 24.99744225 | 24.94891167 | 24.87965584 | 24.78151703 | 24.88911247 | 24.75552177 |
|  | 1.851214972 | 0.133331299 | Q9QXT0;A0A1W2P729 | Protein canopy homolog 2 | Cnpy2 | 25.01588821 | 25.022295 | 25.09768105 | 24.8754425 | 24.93247032 | 24.92795753 |
|  | 0.31124133 | 0.133460999 | Q3UEB3 | Poly(U)-binding-splicing factor PUF60 | Puf60 | 22.73603439 | NaN | 22.69342804 | 22.57514763 | 22.81058884 | 22.35807419 |
|  | 1.251327711 | 0.133930842 | B1AV77;B1ATI0;P47740;A0A140LJF9;P47739 | Aldehyde dehydrogenase;Fatty aldehyde dehydrogenase | Aldh3a2 | 23.97657394 | 23.89716911 | 23.85557365 | 23.84642029 | 23.73293495 | 23.74816895 |
|  | 0.39936293 | 0.134075801 | Q9Z2N8;D3YVN1;A0A0A6YWG8;A0A0A6YWR1 | Actin-like protein 6A | Actl6a | 23.66676521 | 23.72442436 | NaN | 23.35957718 | 23.61593819 | 23.7090416 |
|  | 0.686230672 | 0.134154002 | Q8CHK3 | Lysophospholipid acyltransferase 7 | Mboat7 | 23.0697937 | 22.89816475 | 22.81376076 | 22.71672058 | 22.87958908 | 22.78294754 |
|  | 1.98150138 | 0.134642283 | P35278;Q8C266;A2A5F5;A2A5F6 | Ras-related protein Rab-5C | Rab5c | 26.31582642 | 26.27184677 | 26.29354668 | 26.17058372 | 26.10811043 | 26.19859886 |
|  | 0.878294022 | 0.134706497 | A2AI52;Q80U87 | Ubiquitin carboxyl-terminal hydrolase;Ubiquitin carboxyl-terminal hydrolase 8 | Usp8 | 22.43814468 | 22.28294182 | 22.30005836 | 22.30374146 | 22.12751389 | 22.18577003 |
|  | 0.376660339 | 0.135294278 | M0QWS4;Q9CR09 | Ubiquitin-fold modifier-conjugating enzyme 1 | Ufc1 | NaN | 23.41982651 | 23.37782478 | 23.04738426 | 23.32209778 | 23.42111206 |
|  | 0.801758835 | 0.135404587 | Q9JI11;A0A2I3BQE0;Q9JI10;A2A5M9;Q8CDG4;F7BYZ4 | Serine/threonine-protein kinase 4;Serine/threonine-protein kinase 4 37kDa subunit;Serine/threonine-protein kinase 4 18kDa subunit | Stk4 | 23.53076744 | 23.34867859 | 23.33892059 | 23.2133255 | 23.2351017 | 23.36372566 |
|  | 1.54849642 | 0.136339188 | O88569;A0A0N4SUM2 | Heterogeneous nuclear ribonucleoproteins A2/B1 | Hnrnpa2b1 | 27.10279465 | 27.10389519 | 27.16557121 | 27.03695297 | 27.00592422 | 26.92036629 |
|  | 0.32927487 | 0.136946996 | E9QKE4;A0A0A6YWM5;Q8BMG7 | Rab3 GTPase-activating protein non-catalytic subunit | Rab3gap2 | 22.35995293 | 22.52013397 | 22.59423065 | 22.46318626 | 22.04576874 | 22.55452156 |
|  | 1.288833603 | 0.136952082 | Q9JII6;B1AXW3 | Alcohol dehydrogenase [NADP(+)] | Akr1a1 | 29.04429436 | 29.17859459 | 29.10529518 | 29.02725601 | 28.97092628 | 28.91914558 |
|  | 1.870122274 | 0.13719813 | P24369 | Peptidyl-prolyl cis-trans isomerase B | Ppib | 26.78281784 | 26.78006554 | 26.75748062 | 26.69471359 | 26.58663368 | 26.62742233 |
|  | 0.26372608 | 0.13722229 | Q8BFW7 | Lipoma-preferred partner homolog | Lpp | 23.02862358 | 23.15815353 | 23.50081635 | 23.04628563 | 23.37610245 | 22.85353851 |
|  | 0.299931673 | 0.137528737 | Q8BJF9;A0A338P7L8 | Charged multivesicular body protein 2b | Chmp2b | 22.87447739 | 22.66262054 | 22.65497398 | 22.85599136 | 22.27005005 | 22.65344429 |
|  | 1.997030141 | 0.137718836 | Q9JHI5 | Isovaleryl-CoA dehydrogenase, mitochondrial | Ivd | 23.92623901 | 23.90701485 | 23.95180321 | 23.83942032 | 23.78636169 | 23.74611855 |
|  | 0.460569263 | 0.138106028 | O88587;D3Z227 | Catechol O-methyltransferase | Comt | 23.51593018 | 23.43440819 | 23.47072792 | 23.10442352 | 23.35863876 | 23.54368591 |
|  | 0.230965707 | 0.138201714 | Q99NH8 | Triggering receptor expressed on myeloid cells 2 | Trem2 | NaN | 22.77756119 | 22.57165718 | 22.83786201 | 22.50811577 | 22.26324463 |
|  | 2.100945098 | 0.138650258 | Q8R1B4;M0QWV3 | Eukaryotic translation initiation factor 3 subunit C | Eif3c | 24.10437584 | 24.11378479 | 24.11020279 | 23.92524338 | 24.02191353 | 23.96525574 |
|  | 0.460285884 | 0.139284134 | Q4LDD4;E9PUB0;D3YWW7 | Arf-GAP with Rho-GAP domain, ANK repeat and PH domain-containing protein 1 | Arap1 | 23.338377 | 23.31893158 | 23.06598663 | 23.29445839 | 22.98834229 | 23.02264214 |
|  | 0.415319165 | 0.139384588 | P47941;A0A338P6Q0;A0A338P675 | Crk-like protein | Crkl | 23.03915596 | 22.72054672 | 22.62557983 | 22.78180885 | 22.54443932 | 22.64088058 |
|  | 1.565077194 | 0.139690399 | P62962;Q5SX49;J3QMC2 | Profilin-1;Profilin | Pfn1 | 28.76701927 | 28.81531143 | 28.85355759 | 28.69776535 | 28.71138573 | 28.60766602 |
|  | 1.71201558 | 0.140564601 | Q8K310;A0A494BAZ2;A0A494BAZ7;A0A494BAD4;A0A494B9X1;A0A494BAL7;A0A494B9B1;A0A494BAC5;A0A494BB50;A0A494B968;A0A494B9C9;A0A494B8Y5;A0A494B9Q4 | Matrin-3 | Matr3 | 23.70450974 | 23.75246239 | 23.71983719 | 23.62622643 | 23.51677132 | 23.61211777 |
|  | 1.604375436 | 0.140570323 | Q99N69;A0A494B990 | Leupaxin | Lpxn | 24.78885269 | 24.87455177 | 24.84421921 | 24.67718315 | 24.75633621 | 24.65239334 |
|  | 1.383229187 | 0.140664419 | Q9CQ80;A2A4J8;A8XY17;A2A4K0;E9PXS9 | Vacuolar protein-sorting-associated protein 25 | Vps25 | 23.59331894 | 23.50494385 | 23.45525551 | 23.38876915 | 23.32937241 | 23.41338348 |
|  | 3.015900004 | 0.14103953 | P53994;Q3TEG7;P59279;G3UXQ7;G3V022;A0A1D5RMH1 | Ras-related protein Rab-2A;Ras-related protein Rab-2B | Rab2a;Rab2b | 25.24604797 | 25.2853508 | 25.29834557 | 25.14250374 | 25.13551331 | 25.1286087 |
|  | 1.891181964 | 0.141196569 | Q9CQC9 | GTP-binding protein SAR1b | Sar1b | 22.96687508 | 22.91336632 | 23.00223351 | 22.77898407 | 22.84486961 | 22.83503151 |
|  | 2.965424473 | 0.14183108 | Q80T06;P57776;A0A0R4J1E2;E9QN08;D3YUQ9;D3YZT9 | Elongation factor 1-delta | Eef1d | 26.49455833 | 26.46916008 | 26.43680763 | 26.32601357 | 26.32134247 | 26.32767677 |
|  | 0.884191481 | 0.141959508 | Q9D832 | DnaJ homolog subfamily B member 4 | Dnajb4 | 22.08540726 | 22.20755959 | 22.02806664 | NaN | 21.97410774 | 21.95599556 |
|  | 2.002525739 | 0.142024358 | P48036 | Annexin A5 | Anxa5 | 28.87361336 | 28.87026596 | 28.91780853 | 28.72211266 | 28.79847717 | 28.71502495 |
|  | 0.48828153 | 0.14214325 | Q8BGU5 | Cyclin-Y | Ccny | 22.64632034 | 23.00219917 | 22.62526703 | 22.60564423 | 22.56376648 | 22.67794609 |
|  | 0.332652158 | 0.142227173 | Q8VCF0 | Mitochondrial antiviral-signaling protein | Mavs | 23.21528244 | 23.63267326 | 23.22180176 | 23.37065887 | 23.26913643 | 23.00328064 |
|  | 1.921207966 | 0.142330805 | Q8C2Q8;Q91VR2;A2AKU9;A2AKV1;A2AKV2;A2AKV3;A2AKV0 | ATP synthase subunit gamma;ATP synthase subunit gamma, mitochondrial | Atp5c1 | 25.75417137 | 25.80008316 | 25.85193253 | 25.64875221 | 25.69173622 | 25.63870621 |
|  | 1.493592202 | 0.142354965 | P62075;A0A1W2P756;A0A1W2P7H2 | Mitochondrial import inner membrane translocase subunit Tim13 | Timm13 | 24.27241516 | 24.31312943 | 24.29340744 | 24.17615128 | 24.20813942 | 24.06759644 |
|  | 2.033390721 | 0.142490387 | Q05816 | Fatty acid-binding protein, epidermal | Fabp5 | 28.70266151 | 28.67754555 | 28.67397881 | 28.55464554 | 28.58491516 | 28.48715401 |
|  | 0.532247447 | 0.142692566 | P48758;A0A338P684 | Carbonyl reductase [NADPH] 1 | Cbr1 | 22.97510529 | 22.92145157 | 23.0827446 | 22.72889328 | 23.06621742 | 22.75611305 |
|  | 0.82495049 | 0.143505096 | Q80ZX0;F6VJC5;F6YIN5;A2AA71;Q3U2P1 | Sec24 related gene family, member B (S. cerevisiae) | Sec24b | 22.3461647 | 22.14379501 | 22.28308296 | 22.00617599 | 22.17301178 | 22.16333961 |
|  | 0.465519212 | 0.143623034 | Q9JIH2 | Nuclear pore complex protein Nup50 | Nup50 | 22.01699257 | 21.8821125 | 22.00130463 | 21.66864967 | NaN | 21.97771072 |
|  | 0.970759968 | 0.144496282 | A0A2I3BQL9;A0A0R4J0G0;Q8BH04;A0A2I3BS39;A0A2I3BRX1;A0A2I3BQ75;A0A2I3BQC6 | Phosphoenolpyruvate carboxykinase [GTP], mitochondrial | Pck2 | 23.08053589 | 23.0427494 | 23.18007278 | 22.89655876 | 22.90389442 | 23.06941605 |
|  | 2.028697531 | 0.14521726 | Q3TVK3;Q9Z2W0;Q8BPW9;A0A087WS31;A0A087WSE6;A0A087WRC1;A0A087WSU0;A0A087WNX3;A0A087WSD3 | Aspartyl aminopeptidase | Dnpep | 24.05357933 | 23.9908371 | 24.02960014 | 23.92886162 | 23.84842682 | 23.86107635 |
|  | 0.724099689 | 0.145243327 | E9PWZ6;A0A1D5RLW4;A0A8V5KX73;E9PZW8;Q9QY06 | Unconventional myosin-IXb | Myo9b | 24.03422737 | 24.25803375 | 23.95562172 | 23.91488075 | 23.93192863 | 23.96534348 |
|  | 0.546957864 | 0.145494461 | Q9WTX5;E9PUV4 | S-phase kinase-associated protein 1 | Skp1 | 23.80695534 | 23.92352104 | 23.76658249 | 23.62900925 | 23.53587723 | 23.89568901 |
|  | 0.644815649 | 0.146601359 | Q61333;D3Z4L9;G3XA62;G3UWJ7;G3UXC6 | Tumor necrosis factor alpha-induced protein 2 | Tnfaip2 | 22.44811249 | 22.37867165 | 22.58258629 | 22.48620605 | 22.21080208 | 22.27255821 |
|  | 0.41963534 | 0.146617254 | Q7TMY8;A2AFQ0;F6XP90 | E3 ubiquitin-protein ligase HUWE1 | Huwe1 | 21.51210022 | 21.11407089 | 21.53055382 | 21.19892502 | 21.15930939 | 21.35863876 |
|  | 2.374086328 | 0.14676857 | Q9WUA3;Q8C605;D3YUA3;F6YL81 | ATP-dependent 6-phosphofructokinase, platelet type;ATP-dependent 6-phosphofructokinase | Pfkp | 24.62466431 | 24.58926582 | 24.59815407 | 24.44796181 | 24.42348671 | 24.50032997 |
|  | 2.61388321 | 0.14704895 | P17751;H7BXC3 | Triosephosphate isomerase | Tpi1 | 28.23813057 | 28.16413307 | 28.19713593 | 28.0514431 | 28.0484848 | 28.05832481 |
|  | 3.624450135 | 0.147320429 | Q52KG9;P80317;Q61390;B1AT05 | T-complex protein 1 subunit zeta | Cct6a | 25.44695282 | 25.41979408 | 25.45722961 | 25.29921913 | 25.29628181 | 25.28651428 |
|  | 0.459187885 | 0.147502899 | Q9CY27;G3UWE1;A0A5F8MQC8;Q52L67;A0A5F8MPU6;A0A1D5RLB4;Q3TAN8;A0A1D5RLH3 | Very-long-chain enoyl-CoA reductase | Tecr | 24.21586037 | 23.93966103 | 23.92243195 | 23.73158836 | 23.83200073 | 24.07185555 |
|  | 0.596143963 | 0.147687912 | D6RG49;Q80XP8 | Protein FAM76B | Fam76b | 20.68543053 | 20.4661274 | 20.39686394 | 20.2324543 | 20.45281982 | 20.420084 |
|  | 2.545750794 | 0.147815069 | Q61335 | B-cell receptor-associated protein 31 | Bcap31 | 26.24190903 | 26.2107048 | 26.1978302 | 26.09844398 | 26.03491974 | 26.0736351 |
|  | 0.589107522 | 0.148294449 | Q9Z1N5;G3UXI6 | Spliceosome RNA helicase Ddx39b | Ddx39b | 23.99541664 | 24.2657795 | 24.22059059 | 24.04410172 | 23.86919594 | 24.12360573 |
|  | 0.651545799 | 0.148877462 | Q9Z0Y1;E9Q919 | Dynactin subunit 3 | Dctn3 | 23.43211555 | 23.66568184 | 23.47481728 | 23.23908424 | 23.49484825 | 23.39204979 |
|  | 0.145981465 | 0.150047302 | Q9DC61;A2AIW9 | Mitochondrial-processing peptidase subunit alpha | Pmpca | 22.29445839 | NaN | 22.61874199 | 22.62370491 | NaN | 21.98940086 |
|  | 1.350702204 | 0.150369644 | P46460;G3UX86;G3UX98 | Vesicle-fusing ATPase | Nsf | 24.20486069 | 24.18328857 | 24.33831024 | 24.12746811 | 24.062994 | 24.08488846 |
|  | 1.156132341 | 0.15069135 | P61804 | Dolichyl-diphosphooligosaccharide--protein glycosyltransferase subunit DAD1 | Dad1 | 24.94641495 | 24.84751892 | 24.80015564 | 24.71492004 | 24.63804436 | 24.78905106 |
|  | 1.167472957 | 0.150695165 | Q8VDP3;E9PUI4;D3Z5P6 | Protein-methionine sulfoxide oxidase MICAL1 | Mical1 | 23.54710197 | 23.61829376 | 23.57888222 | 23.50675964 | 23.46650124 | 23.31893158 |
|  | 1.208778383 | 0.150922139 | P62627;A2AVR9 | Dynein light chain roadblock-type 1 | Dynlrb1 | 24.60873795 | 24.71040726 | 24.53990555 | 24.4084034 | 24.51436615 | 24.48351479 |
|  | 0.698520867 | 0.151442846 | Q922H4;D3Z3L0;D3Z3M0;D3Z2L8;D3YVK2;D3Z5Z5;D3Z0T7 | Mannose-1-phosphate guanyltransferase alpha | Gmppa | 22.58897972 | 22.72579765 | 22.66789246 | 22.42419052 | 22.69040489 | 22.41374588 |
|  | 1.192015443 | 0.151691437 | Q9Z0P5;A0A1L1SU53;A0A1L1STC8;A0A087WRG4 | Twinfilin-2 | Twf2 | 25.01907349 | 25.00508881 | 25.03136826 | 24.79530907 | 24.82055473 | 24.98459244 |
|  | 0.567522451 | 0.151847204 | E9QP59;D3YU56;Q9WU40 | Inner nuclear membrane protein Man1 | Lemd3 | 20.42028999 | 20.64825249 | 20.40509605 | 20.41493225 | 20.44151688 | 20.1616478 |
|  | 2.089093531 | 0.152272542 | O08529 | Calpain-2 catalytic subunit | Capn2 | 25.75989532 | 25.76065636 | 25.82476044 | 25.59083748 | 25.62856483 | 25.66909218 |
|  | 0.645661159 | 0.152457555 | P35235 | Tyrosine-protein phosphatase non-receptor type 11 | Ptpn11 | 21.87346458 | 22.20015335 | 22.05940056 | 21.89437675 | 21.80534172 | 21.97592735 |
|  | 0.943141585 | 0.152557373 | Q8C7X2;Z4YJW0;A0A1Y7VP81 | ER membrane protein complex subunit 1 | Emc1 | 23.47580719 | 23.26728058 | 23.45500565 | 23.17433929 | 23.2896843 | 23.27639771 |
|  | 1.312747117 | 0.152669271 | P49722;Q8BKE0 | Proteasome subunit alpha type-2 | Psma2 | 24.74021149 | 24.81562996 | 24.80945969 | 24.53937149 | 24.67060661 | 24.69731522 |
|  | 1.86376914 | 0.152955373 | P62774;A0A0J9YV46 | Myotrophin | Mtpn | 26.40861511 | 26.39865303 | 26.36664772 | 26.1882782 | 26.30358124 | 26.22319031 |
|  | 0.505294673 | 0.153364182 | Q9ERK4;E9Q1T9;E9QAX7;F6ZEW4 | Exportin-2 | Cse1l | 28.32444382 | 28.09446335 | 28.21752548 | 28.27253151 | 28.02527618 | 27.87853241 |
|  | 0.658838205 | 0.153612137 | D3Z041;P41216;D3Z457;D3YVF6;A0A1B0GRS8;F6WNZ2 | Long-chain-fatty-acid--CoA ligase 1 | Acsl1 | 23.24064255 | 23.35447502 | 23.20327759 | 23.19415283 | 22.92257881 | 23.2208271 |
|  | 1.115487676 | 0.154010773 | P29758 | Ornithine aminotransferase, mitochondrial | Oat | 24.63256264 | 24.42393494 | 24.46842957 | 24.3269062 | 24.37112617 | 24.36486244 |
|  | 3.746315409 | 0.154133479 | P48774;E9PVM7;E9PV63 | Glutathione S-transferase Mu 5 | Gstm5 | 22.39152527 | 22.36847687 | 22.37618256 | 22.20913696 | 22.22337914 | 22.24126816 |
|  | 1.403780095 | 0.154289246 | Q04447 | Creatine kinase B-type | Ckb | 28.34100151 | 28.40730286 | 28.39136887 | 28.30264854 | 28.1399498 | 28.23420715 |
|  | 0.619134285 | 0.154408455 | Q9QZB9;H3BJ75 | Dynactin subunit 5 | Dctn5 | 22.17273712 | 22.23022652 | NaN | 21.90936089 | 22.18719101 | 22.0446682 |
|  | 1.833733979 | 0.154599508 | Q9QZD9;A2AE03 | Eukaryotic translation initiation factor 3 subunit I | Eif3i | 24.67696762 | 24.6654644 | 24.61840439 | 24.54014206 | 24.52318764 | 24.43370819 |
|  | 2.142203714 | 0.154845556 | Q9D7P6;D3Z7W0;D3Z0K9 | Iron-sulfur cluster assembly enzyme ISCU, mitochondrial | Iscu | 23.44151688 | 23.45688629 | 23.37504196 | 23.24219894 | 23.26441956 | 23.30228996 |
|  | 1.80917163 | 0.15514946 | P80316;E0CZA1 | T-complex protein 1 subunit epsilon | Cct5 | 25.28996468 | 25.3692627 | 25.38396645 | 25.18196106 | 25.23994446 | 25.15583992 |
|  | 2.053429612 | 0.156697591 | O54774;A0A1W2P6Q6 | AP-3 complex subunit delta-1 | Ap3d1 | 24.17744446 | 24.2540741 | 24.24168968 | 24.07822418 | 24.02419853 | 24.10069275 |
|  | 1.165773183 | 0.15687116 | Q6ZWZ7;Q9CPR4;B2RY53 | 60S ribosomal protein L17 | Rpl17 | 25.58336639 | 25.38811111 | 25.41451454 | 25.28901482 | 25.33756065 | 25.2888031 |
|  | 1.248434702 | 0.157548904 | O35075;A0A338P6T5 | Down syndrome critical region protein 3 homolog | Dscr3 | 22.85832787 | 22.8726368 | 22.90883064 | 22.69106483 | 22.83352661 | 22.64255714 |
|  | 0.206680033 | 0.157639503 | Q921F4;V9GXB6 | Heterogeneous nuclear ribonucleoprotein L-like | Hnrnpll | 22.95404243 | NaN | 22.75113487 | 22.94779778 | 22.44210052 | NaN |
|  | 1.112121669 | 0.157718658 | A2AKI5;P43406;F6RWM8;F6W6Q6 | Integrin alpha-V;Integrin alpha-V heavy chain;Integrin alpha-V light chain | Itgav | 23.33660698 | 23.19088554 | 23.41053963 | 23.14558411 | 23.1316185 | 23.18767357 |
|  | 0.544672303 | 0.157770793 | P62835;A0A0G2JE52;A0A0G2JED9 | Ras-related protein Rap-1A | Rap1a | 24.80493927 | 24.54126358 | 24.91506386 | 24.59900665 | 24.70524788 | 24.4836998 |
|  | 0.278990635 | 0.158308665 | Q8CH18;A0A1W2P7Q7;A0A1W2P765 | Cell division cycle and apoptosis regulator protein 1 | Ccar1 | 21.64535141 | 22.2268219 | NaN | 21.84068871 | 21.72075462 | 21.77189064 |
|  | 0.562376141 | 0.158892949 | Q78IS1 | Transmembrane emp24 domain-containing protein 3 | Tmed3 | 22.64910698 | NaN | 22.88323021 | 22.48293686 | 22.68251801 | 22.65637207 |
|  | 0.908028473 | 0.159221013 | Q9EPU0 | Regulator of nonsense transcripts 1 | Upf1 | 24.28700829 | 24.23125267 | 24.41144562 | 24.08569717 | 24.27490616 | 24.0914402 |
|  | 0.39521541 | 0.15970993 | Q9CQ10;A0A0N4SVS3 | Charged multivesicular body protein 3 | Chmp3 | 23.10187912 | 23.57091713 | 23.15398598 | 23.24251938 | 22.95597649 | 23.14915657 |
|  | 1.573060753 | 0.15990448 | P0DP28;P0DP27;P0DP26;A0A3Q4EHJ0;Q9D6P8;G3UX57;P20801 | Calmodulin-like protein 3 | Calml3 | 28.67209053 | 28.69142342 | 28.71338654 | 28.50286674 | 28.62111282 | 28.47320747 |
|  | 1.051324525 | 0.160070419 | P63028;D3YU75 | Translationally-controlled tumor protein | Tpt1 | 26.95349312 | 26.85424232 | 27.01935005 | 26.86655617 | 26.68431664 | 26.79600143 |
|  | 1.198506377 | 0.160242081 | P97384;D3Z7U0 | Annexin A11;Annexin | Anxa11 | 24.53504562 | 24.49240494 | 24.36279106 | 24.25932693 | 24.27653885 | 24.3736496 |
|  | 0.428355006 | 0.160366694 | D3YUK4;Q9DCS9 | NADH dehydrogenase [ubiquinone] 1 beta subcomplex subunit 10 | Ndufb10 | 23.33483696 | 23.05903625 | 23.19539833 | 23.06399727 | 22.78290749 | 23.26126671 |
|  | 1.85037608 | 0.160642624 | P18242;F8WIR1;F6Y6L6;A0A1B0GT66 | Cathepsin D | Ctsd | 29.64938164 | 29.70450974 | 29.73805428 | 29.48158455 | 29.55054092 | 29.5778923 |
|  | 1.724693454 | 0.1613458 | P53810;J3QQ30;J3QPW1;F8WGG5 | Phosphatidylinositol transfer protein alpha isoform | Pitpna | 26.49725723 | 26.50966263 | 26.46368217 | 26.25437927 | 26.34082413 | 26.39136124 |
|  | 1.140082786 | 0.16154925 | Q9CQI3;A0A2I3BR94;A0A2I3BPS1;D3YY16 | Glia maturation factor beta | Gmfb | 26.02235794 | 25.93271828 | 25.96060181 | 25.691576 | 25.84445763 | 25.89499664 |
|  | 2.042337938 | 0.162481308 | P20060 | Beta-hexosaminidase subunit beta | Hexb | 26.0645771 | 26.16107941 | 26.16050339 | 25.96842384 | 25.98620033 | 25.9440918 |
|  | 1.191051876 | 0.162778854 | Q9CX30;D3YY42;A0A140LHN0;D3Z5F9 | Protein YIF1B | Yif1b | 23.00262833 | 23.01852226 | 22.9014473 | NaN | 22.85367203 | 22.76916885 |
|  | 1.327603908 | 0.162825267 | Q9CX34 | Suppressor of G2 allele of SKP1 homolog | Sugt1 | 23.86957169 | 24.04251671 | 23.89901352 | 23.77786064 | 23.7361393 | 23.80862617 |
|  | 0.799409803 | 0.163350423 | P28352;D3Z124;D3Z6R9 | DNA-(apurinic or apyrimidinic site) lyase;DNA-(apurinic or apyrimidinic site) lyase, mitochondrial | Apex1 | 24.09595299 | 23.84823608 | 23.84852219 | 23.68324661 | 23.77826309 | 23.84115028 |
|  | 1.471569102 | 0.163660685 | Q9CQ60;D3Z4X1;Q8CBG6;F6X8L5 | 6-phosphogluconolactonase | Pgls | 25.17253113 | 25.01729012 | 25.06509018 | 24.93979454 | 24.94953537 | 24.87459946 |
|  | 0.951646952 | 0.163902283 | Q64152 | Transcription factor BTF3 | Btf3 | 25.01120186 | 25.00165749 | 25.20821381 | 24.98858643 | 24.90559387 | 24.835186 |
|  | 1.448246909 | 0.163984934 | Q9R0P5 | Destrin | Dstn | 24.9732933 | 24.94873238 | 24.83243561 | 24.7817688 | 24.78586388 | 24.69487381 |
|  | 0.446739003 | 0.164382299 | Q9D8S9 | BolA-like protein 1 | Bola1 | 22.94785118 | 22.56579018 | 22.81088257 | 22.82057571 | 22.57260513 | 22.43819618 |
|  | 0.842494798 | 0.164463679 | P17225;Q8CB58;E9QMW9;F7DCW4;E9Q279;F7AXP1;E9Q0W3 | Polypyrimidine tract-binding protein 1 | Ptbp1 | 24.85785294 | 24.78216743 | 24.83311081 | 24.70783043 | 24.4895916 | 24.78231812 |
|  | 0.406293358 | 0.164673487 | Q9Z2M7;Q9D6D9;Q91W01;A0A2R8W6J8;O35621 | Phosphomannomutase 2 | Pmm2 | 24.0464344 | 24.09442329 | 24.25292015 | 23.88688087 | 24.27519035 | 23.73768616 |
|  | 1.341042795 | 0.165435791 | Q791V5;A2AFW6;Q9D050 | Mitochondrial carrier homolog 2 | Mtch2 | 24.66898537 | 24.67449188 | 24.82254982 | 24.5333252 | 24.61284828 | 24.52354622 |
|  | 1.582344888 | 0.167285283 | Q9D1D4;A0A1Y7VM54 | Transmembrane emp24 domain-containing protein 10 | Tmed10 | 25.92994499 | 26.01250267 | 25.97795105 | 25.88492775 | 25.74031448 | 25.79330063 |
|  | 0.457583927 | 0.168551763 | Q8CG76 | Aflatoxin B1 aldehyde reductase member 2 | Akr7a2 | 23.38548088 | 23.09836388 | 23.34299469 | 23.31865501 | 22.86644363 | 23.13608551 |
|  | 0.865170044 | 0.169285456 | Q91YW3 | DnaJ homolog subfamily C member 3 | Dnajc3 | 23.1893177 | 23.25034904 | 23.23164749 | 22.92288589 | 23.01595688 | 23.2246151 |
|  | 0.994824127 | 0.169668833 | Q6PAR5;F7ADT6;F7ADQ2;E9Q0D1;F7ADS7;F6X819 | GTPase-activating protein and VPS9 domain-containing protein 1 | Gapvd1 | 22.3923111 | 22.36668968 | 22.17764282 | 22.09793091 | 22.2277317 | 22.10197449 |
|  | 0.972304668 | 0.170646032 | P08207 | Protein S100-A10 | S100a10 | 26.83303833 | 26.84959602 | 26.86029434 | 26.81824112 | 26.53473473 | 26.67801476 |
|  | 0.461756793 | 0.171046257 | Q9ESW8;A0A1B0GSY1;A0A1B0GSC4 | Pyroglutamyl-peptidase 1 | Pgpep1 | NaN | 22.92315865 | 22.86094284 | 22.85715103 | 22.58485794 | NaN |
|  | 0.637324077 | 0.171105067 | Q9CRB9;F6QFL0;S4R238;Q9D9P1;D3Z0L4 | MICOS complex subunit Mic19 | Chchd3 | 23.43096733 | 23.70144653 | 23.55555153 | 23.44303703 | 23.51965332 | 23.21195984 |
|  | 2.499918452 | 0.171341578 | Q64727 | Vinculin | Vcl | 24.00958061 | 24.01648331 | 24.07618523 | 23.87455177 | 23.88408852 | 23.82958412 |
|  | 0.473576533 | 0.172093074 | O08795;A0A1L1ST83 | Glucosidase 2 subunit beta | Prkcsh | 24.70961952 | 24.52522087 | 24.41021729 | 24.56697464 | 24.43701553 | 24.12478828 |
|  | 0.898721622 | 0.172247569 | P38060;V9GXD2;V9GWZ9;D3YVU9;Q8JZS7 | Hydroxymethylglutaryl-CoA lyase, mitochondrial | Hmgcl | 24.18078613 | 23.96763229 | 23.98433304 | 23.9045372 | 23.95127106 | 23.7602005 |
|  | 1.165124806 | 0.172664007 | Q6DFW4;A0A0A0MQ76;A0A087WQ46;A0A087WSU5;A0A087WSL8;A0A087WP00;A0A087WNW0 | Nucleolar protein 58 | Nop58 | 23.32882309 | 23.3998909 | 23.50324631 | 23.18278694 | 23.33333588 | 23.19784546 |
|  | 0.825689136 | 0.173957825 | Q6PD26 | GPI transamidase component PIG-S | Pigs | 22.18758202 | 22.38200188 | 22.13018799 | 21.94406509 | 22.15186691 | 22.0819664 |
|  | 1.732768569 | 0.174212774 | Q9JM76;H7BWZ3;A0A0G2JFK7;D3Z2F7;D3Z2F8 | Actin-related protein 2/3 complex subunit 3 | Arpc3 | 27.23917961 | 27.24127197 | 27.13133812 | 27.07673645 | 27.03012657 | 26.98228836 |
|  | 1.526266457 | 0.175435702 | Q64261;A0A0G2JGH2;A0A0G2JGA8 | Cyclin-dependent kinase 6 | Cdk6 | 21.98307991 | 21.96366882 | NaN | 21.80254555 | 21.73632431 | 21.85494614 |
|  | 0 | 0.17551899 | Q8BLN5;F7BJL0 | Lanosterol synthase | Lss | 22.32953644 | 21.48306084 | NaN | NaN | 21.73077965 | NaN |
|  | 2.486090622 | 0.175658544 | Q91Z25;Q9WV32;F6VVE6;F6THG2;D3Z6S0 | Actin-related protein 2/3 complex subunit 1B | Arpc1b | 27.00066948 | 26.98294067 | 27.01169395 | 26.80574989 | 26.87502098 | 26.7875576 |
|  | 2.012552721 | 0.176121394 | P57746 | V-type proton ATPase subunit D | Atp6v1d | 25.07349205 | 25.00782776 | 24.9908371 | 24.90324974 | 24.80945969 | 24.8310833 |
|  | 0.995941171 | 0.176182429 | Q99K70;B1AWT2;B1AWT3;Q7TT45;B1AWT4 | Ras-related GTP-binding protein C;Ras-related GTP-binding protein D | Rragc;Rragd | 23.8439312 | 23.93039513 | 23.87539673 | 23.55191994 | 23.81078339 | 23.75847244 |
|  | 0.663162782 | 0.176468531 | P06797;A0A1Y7VP49;A0A1Y7VNM3;A0A1Y7VNQ4 | Cathepsin L1;Cathepsin L1 heavy chain;Cathepsin L1 light chain | Ctsl | 24.65954399 | 24.55818367 | 24.49588585 | 24.6145916 | 24.30820656 | 24.26140976 |
|  | 0.47686856 | 0.17739741 | A0A286YDI8;G3X972;A0A286YD08;A0A2I3BQS4 | Sec24 related gene family, member C (S. cerevisiae) | Sec24c | 22.78849411 | 23.07885933 | 22.63464737 | 22.75658035 | 22.46587944 | 22.74734879 |
|  | 2.539326216 | 0.177797318 | Q9DB77;A0A140LI98 | Cytochrome b-c1 complex subunit 2, mitochondrial | Uqcrc2 | 25.35746384 | 25.30016327 | 25.33469963 | 25.11402321 | 25.15591812 | 25.18899345 |
|  | 1.989875704 | 0.177797953 | P10126;D3YZ68;D3Z3I8 | Elongation factor 1-alpha 1 | Eef1a1 | 30.91627121 | 30.8060112 | 30.85682678 | 30.69420052 | 30.71284676 | 30.63866806 |
|  | 2.310013497 | 0.177897771 | Q99JY0;D3YXU1 | Trifunctional enzyme subunit beta, mitochondrial;3-ketoacyl-CoA thiolase | Hadhb | 24.55695534 | 24.48879433 | 24.55695534 | 24.31299019 | 24.38416481 | 24.37185669 |
|  | 0.877516911 | 0.178064346 | Q8BMA6;A2AAN2 | Signal recognition particle subunit SRP68 | Srp68 | 23.24286842 | 23.45651054 | 23.24569893 | 23.23952103 | 23.02340317 | 23.14796066 |
|  | 0.285585876 | 0.178076426 | Q5UE59;E9Q7C9;Q7TNF4;Q8CD76;A0A5F8MPZ2;O88447;D3YXZ3;Q91YS4;O88448;F6UYN4 | Kinesin light chain 1 | Klc1 | 22.96278763 | 23.07783318 | 23.12444115 | 23.08577919 | 22.38492775 | 23.16012573 |
|  | 1.850467449 | 0.178245544 | D3Z158;Q8BML9;A0A140LHZ5;A0A0A6YY08;A0A140LIS6;A0A140LIN2;A0A140LHJ3;A0A140LHB3;A0A140LJH2;A0A140LID3;A0A140LJK5;A0A140LJH1;A0A140LIZ4;A0A140LIR0;F6TDS3;A0A140LII8 | glutamine--tRNA ligase | Qars | 24.17820549 | 24.15668869 | 24.08432007 | 23.99808884 | 23.89763069 | 23.98875809 |
|  | 1.031074835 | 0.178286235 | P99026 | Proteasome subunit beta type-4 | Psmb4 | 25.31205559 | 25.25353241 | 25.31741333 | 24.95890045 | 25.19336319 | 25.19587898 |
|  | 0.500653783 | 0.178596497 | P70279;A2ALA0 | Surfeit locus protein 6 | Surf6 | NaN | 22.48007965 | 22.21115875 | NaN | 22.17298126 | 22.16106415 |
|  | 1.047791594 | 0.178698858 | Q3U422 | NADH dehydrogenase [ubiquinone] flavoprotein 3, mitochondrial | Ndufv3 | 21.91714096 | 21.72379875 | 21.93286705 | 21.70066261 | 21.59532356 | 21.74172401 |
|  | 1.39343946 | 0.179408391 | Q8R5A3;S4R2K5;F2Z4B7;G5E867;F2Z3U3 | Amyloid beta A4 precursor protein-binding family B member 1-interacting protein | Apbb1ip | 24.67540741 | 24.50057411 | 24.5550251 | 24.42643166 | 24.4299469 | 24.33640289 |
|  | 1.394021075 | 0.179484685 | P46471;Q8BVQ9 | 26S protease regulatory subunit 7 | Psmc2 | 24.56981659 | 24.67125511 | 24.51773262 | 24.45123672 | 24.32765961 | 24.44145393 |
|  | 0.73961941 | 0.179490407 | P97300;H3BIX4;Z4YLB7;A0A0A0MQN8;H3BKA7 | Neuroplastin | Nptn | 23.51881409 | 23.53148079 | 23.32800293 | 23.35676003 | 23.38205338 | 23.10101318 |
|  | 0.869499478 | 0.180618286 | Q9ES28;A0A0R4J0X8;D3Z0V2;A0A1B0GRS3;A0A1B0GSX4 | Rho guanine nucleotide exchange factor 7 | Arhgef7 | 23.56022644 | 23.53907585 | 23.45701027 | 23.48873329 | 23.17313385 | 23.35259056 |
|  | 1.520444912 | 0.181329727 | O55135;A6PWZ2;D6RG53;D6RJJ3;B1AZQ4 | Eukaryotic translation initiation factor 6 | Eif6 | 24.00547409 | 24.10293579 | 23.96859932 | 23.91688728 | 23.78964996 | 23.82648277 |
|  | 0.986202921 | 0.18151919 | A0A0N4SW73;Q8R361 | Rab11 family-interacting protein 5 | Rab11fip5 | 22.76255417 | 22.65073013 | 22.62459755 | 22.63597488 | 22.48007965 | 22.37726974 |
|  | 0.492301771 | 0.181608836 | G3X8U3 | Queuosine 5'-phosphate N-glycosylase/hydrolase | 2210016F16Rik | NaN | 22.60313416 | 22.88929749 | 22.59809875 | 22.69304466 | 22.40267754 |
|  | 1.563403042 | 0.181776047 | A0A0R4J259;Q7TMK9;G3UZI2;G3V018;G3UZ48;G3UXJ6;G3UWM1;G3XA76 | Heterogeneous nuclear ribonucleoprotein Q | Syncrip | 24.86207008 | 24.87970161 | 24.76718903 | 24.67637634 | 24.57605934 | 24.7111969 |
|  | 1.926931872 | 0.181894938 | Q9CXW4;A2BH06 | 60S ribosomal protein L11 | Rpl11 | 26.74291039 | 26.80992699 | 26.86726379 | 26.65376091 | 26.58462906 | 26.63602638 |
|  | 0.712547089 | 0.182348251 | P55096;A0A0G2JDI9 | ATP-binding cassette sub-family D member 3 | Abcd3 | 22.85697937 | 22.80220985 | 22.91250801 | 22.85117531 | 22.707798 | 22.46567917 |
|  | 0.473594693 | 0.183235486 | P47856;D3YYD9;D3YYE0 | Glutamine--fructose-6-phosphate aminotransferase [isomerizing] 1 | Gfpt1 | 22.69444847 | 22.61492729 | 22.93183899 | 22.42567635 | NaN | 22.70199585 |
|  | 1.411277615 | 0.183698018 | P12787 | Cytochrome c oxidase subunit 5A, mitochondrial | Cox5a | 26.38977051 | 26.36377716 | 26.38292885 | 26.28926086 | 26.08324432 | 26.21287727 |
|  | 1.45570489 | 0.184001287 | Q6P542 | ATP-binding cassette sub-family F member 1 | Abcf1 | 24.09691811 | 23.94511986 | 24.00538826 | 23.88334274 | 23.75603104 | 23.85604858 |
|  | 0.640631196 | 0.184305827 | Q8K0C9 | GDP-mannose 4,6 dehydratase | Gmds | 23.35353279 | 23.58623314 | 23.49753189 | 23.0754509 | 23.3780899 | 23.43083954 |
|  | 0.459324649 | 0.184701284 | Q8K211;A8Y5P1 | High affinity copper uptake protein 1 | Slc31a1 | 24.3808651 | 24.44739532 | 24.57294083 | 24.47029305 | 24.42162514 | 23.95517921 |
|  | 0.945256212 | 0.184842428 | E9PV44;O35143 | ATPase inhibitor, mitochondrial | Atpif1 | 26.83629417 | 26.6547184 | 26.55360603 | 26.52495193 | 26.41939163 | 26.54574776 |
|  | 0.724621954 | 0.185086568 | Q7TQK5;E9QAD4 | Coiled-coil domain-containing protein 93 | Ccdc93 | 23.71460533 | 23.6216507 | 23.48701668 | 23.27440834 | 23.60365486 | 23.3899498 |
|  | 2.192877761 | 0.185438156 | Q20BD0;Q80XR6;Q99020 | Heterogeneous nuclear ribonucleoprotein A/B | Hnrnpab | 25.65444374 | 25.56743813 | 25.56383705 | 25.3719902 | 25.41992378 | 25.43749046 |
|  | 1.442855624 | 0.18570137 | P28271 | Cytoplasmic aconitate hydratase | Aco1 | 23.51280022 | 23.47877121 | 23.39689064 | 23.3474617 | 23.18300056 | 23.30089569 |
|  | 0.879922513 | 0.186296463 | D3YYK8;E9Q6X0;Q8R001;Q3TG90 | Microtubule-associated protein RP/EB family member 2 | Mapre2 | 22.47174454 | 22.54347229 | 22.30563354 | 22.32635689 | 22.31956482 | 22.11603928 |
|  | 1.274638601 | 0.186394374 | Q9Z2Y8;A0A1B0GRP7;Q6P8V7;A0A1B0GRR5;Q80ZV3;A0A1B0GSP5;A0A1B0GSA1;A0A1B0GQY5 | Proline synthase co-transcribed bacterial homolog protein | Prosc | 24.1484108 | 24.16890335 | 24.14374924 | 23.88994789 | 23.90875626 | 24.10317612 |
|  | 0.62884054 | 0.186458588 | Q9D881;P19536;F7C106;A0A0A6YVR0 | Cytochrome c oxidase subunit 5B, mitochondrial | Cox5b | 25.74658012 | 25.60803413 | 25.75241089 | 25.49722672 | 25.74093246 | 25.3094902 |
|  | 0.896720622 | 0.186644236 | P54728 | UV excision repair protein RAD23 homolog B | Rad23b | 24.65430832 | 24.38284492 | 24.50833321 | 24.25479507 | 24.44107437 | 24.2896843 |
|  | 2.339115979 | 0.18706131 | Q569Z6;Q8BZN7;F6YSQ2;F6YH92 | Thyroid hormone receptor-associated protein 3 | Thrap3 | 23.30396271 | 23.3858757 | 23.29600143 | 23.11714935 | 23.13730431 | 23.17020226 |
|  | 1.030431524 | 0.187296549 | Q3TPJ8;O88487;A2BFF8;A2BFF9;A2BFF5 | Cytoplasmic dynein 1 intermediate chain 2 | Dync1i2 | 24.38238335 | 24.39584541 | 24.38699532 | 24.0556488 | 24.35090446 | 24.19678116 |
|  | 1.06267936 | 0.1874307 | Q8C0C7;E9PWY9;D6RIJ2 | Phenylalanine--tRNA ligase alpha subunit | Farsa | 24.03800011 | 23.91870689 | 24.14654732 | 23.93202019 | 23.75755692 | 23.85138512 |
|  | 1.264986885 | 0.187639872 | O54984;A0A1B0GRE1 | ATPase Asna1 | Asna1 | 24.46985817 | 24.41989136 | 24.39185333 | 24.23337555 | 24.12888336 | 24.35642433 |
|  | 1.170892991 | 0.187878291 | P46467;Q8BPY9 | Vacuolar protein sorting-associated protein 4B | Vps4b | 23.65501785 | 23.61717224 | 23.50494385 | 23.39793396 | 23.30312729 | 23.51243782 |
|  | 1.172201754 | 0.187918981 | Q4FE56;P70398;F8VPU6;E9PWA9;G3UY52;G3UZS3 | Ubiquitin carboxyl-terminal hydrolase;Probable ubiquitin carboxyl-terminal hydrolase FAF-X | Usp9x | 23.06974411 | 23.04748344 | 23.07375336 | 22.97750092 | 22.72957802 | 22.92014503 |
|  | 1.112861831 | 0.187927882 | Q9D8L3;Q62186 | Translocon-associated protein subunit delta | Ssr4 | 24.44865608 | 24.61778831 | 24.46619034 | 24.2493782 | 24.28093338 | 24.43853951 |
|  | 0.611832516 | 0.1892217 | Q9QZ23;A0A0N4SUH8;D3Z285 | NFU1 iron-sulfur cluster scaffold homolog, mitochondrial | Nfu1 | 22.82298851 | 22.8599205 | 23.0537281 | 22.94854546 | 22.67551422 | 22.54491234 |
|  | 1.373868416 | 0.189664841 | Q00PI9;A0A494BAL6 | Heterogeneous nuclear ribonucleoprotein U-like protein 2 | Hnrnpul2 | 23.65359688 | 23.55039406 | 23.60489845 | 23.52157211 | 23.39086914 | 23.32745361 |
|  | 2.458584793 | 0.189723969 | Q8CGK3 | Lon protease homolog, mitochondrial | Lonp1 | 23.63931465 | 23.60625458 | 23.64855957 | 23.40483665 | 23.4963131 | 23.42380714 |
|  | 1.341239013 | 0.189974467 | P61982 | 14-3-3 protein gamma;14-3-3 protein gamma, N-terminally processed | Ywhag | 27.64485168 | 27.62791061 | 27.54200363 | 27.53110886 | 27.35925865 | 27.35447502 |
|  | 0.786144834 | 0.190038045 | P70670;Q60817 | Nascent polypeptide-associated complex subunit alpha, muscle-specific form;Nascent polypeptide-associated complex subunit alpha | Naca | 26.20256424 | 26.46729469 | 26.14775085 | 26.14369011 | 26.12480736 | 25.97899818 |
|  | 2.025361969 | 0.190193812 | P19783;M0QWX7;D6RG40 | Cytochrome c oxidase subunit 4 isoform 1, mitochondrial | Cox4i1 | 25.88602066 | 25.77690887 | 25.80597305 | 25.68115616 | 25.60986519 | 25.6072998 |
|  | 0.791582443 | 0.191079458 | A0A0J9YUM4;A0A0J9YU62;O88712;A0A0J9YUR5;A0A0J9YVI3;A0A0J9YU66;A0A0J9YTW3;A0A0J9YVC3 | C-terminal-binding protein 1 | Ctbp1 | 23.28587914 | 23.09571075 | 23.19111252 | 23.17421722 | 22.9861908 | 22.83905602 |
|  | 1.814171065 | 0.191942851 | P61161 | Actin-related protein 2 | Actr2 | 27.61097908 | 27.57130814 | 27.65867233 | 27.46715546 | 27.45591354 | 27.342062 |
|  | 1.632899945 | 0.192112605 | Q5RKN9;P47753;A0A0G2JE27 | F-actin-capping protein subunit alpha-1 | Capza1 | 25.7843914 | 25.69935608 | 25.69184303 | 25.56871414 | 25.44404793 | 25.58649063 |
|  | 1.1379075 | 0.192750295 | Q9CZ04;D3Z440;D3Z0S0;D3YVI6 | COP9 signalosome complex subunit 7a | Cops7a | 23.68239021 | 23.7470417 | 23.59582329 | 23.37742805 | 23.60569 | 23.46388626 |
|  | 1.313034195 | 0.192875544 | B1AU25;Q9Z0X1 | Apoptosis-inducing factor 1, mitochondrial | Aifm1 | 24.39008141 | 24.3085537 | 24.16284752 | 24.11052132 | 24.0587883 | 24.11354637 |
|  | 0.757296169 | 0.193351746 | E0CXB2;E9QKZ2;Q91YE6;E0CY46;F6UT58 | Importin-9 | Ipo9 | 25.1696682 | 24.8154335 | 24.94109535 | 24.88980865 | 24.74750328 | 24.70882988 |
|  | 1.131293042 | 0.193716049 | Q80SW1 | Putative adenosylhomocysteinase 2 | Ahcyl1 | 22.93669891 | NaN | 22.94922256 | 22.66470528 | 22.85380363 | 22.72922516 |
|  | 0.695825852 | 0.193939845 | A6PWC3;A2A9Q2;Q8BHG1;Q3V3G9 | Nardilysin | Nrd1 | 22.64396667 | 22.48040009 | 22.25600433 | 22.33319855 | 22.14851952 | 22.3168335 |
|  | 1.326055529 | 0.19410642 | Q9EQQ9 | Protein O-GlcNAcase | Mgea5 | 22.51393127 | 22.53179169 | 22.51472664 | 22.45798874 | 22.22966957 | 22.29047203 |
|  | 1.442731598 | 0.19425265 | O89023 | Tripeptidyl-peptidase 1 | Tpp1 | 26.22055435 | 26.1159687 | 26.25192642 | 26.07287788 | 26.02028275 | 25.9125309 |
|  | 1.280782117 | 0.194273631 | E9PUD2;Q8K1M6;A0A2U3TZ67 | Dynamin-1-like protein | Dnm1l | 23.03387451 | 23.15124893 | 23.11553192 | 22.84523392 | 23.03008842 | 22.84251213 |
|  | 0.882788162 | 0.194529215 | P49586;D3Z3T5;Q811Q9 | Choline-phosphate cytidylyltransferase A | Pcyt1a | 22.97921181 | 22.91544724 | 22.8591423 | 22.8594265 | 22.53654099 | 22.77424622 |
|  | 1.893062048 | 0.19467926 | Q61990;B2M1R7;A0A2R8VI25;A0A2R8W6L5;A0A2R8VI12;A0A2R8VHG2;A0A2R8VI71;A0A2R8VKN0;A0A2R8VI73;A0A2R8W6U6;A0A2R8W6H3 | Poly(rC)-binding protein 2 | Pcbp2 | 24.73686028 | 24.81313515 | 24.87262726 | 24.65839958 | 24.58806419 | 24.59212112 |
|  | 2.284838196 | 0.195026398 | Q8R1F1;A2ARS6 | Niban-like protein 1 | Fam129b | 25.6685257 | 25.66348457 | 25.70427322 | 25.48747635 | 25.5385437 | 25.42518425 |
|  | 1.87933208 | 0.195224126 | Q6P8X1;A0A1W2P701;A0A494B972;Q80ZJ7;A0A1W2P6Y0 | Sorting nexin-6;Sorting nexin-6, N-terminally processed | Snx6 | 23.6076107 | 23.50397301 | 23.48775291 | 23.38574409 | 23.33388138 | 23.29403877 |
|  | 1.341239695 | 0.195838928 | Q99J77 | Sialic acid synthase | Nans | 24.11727715 | 24.03707886 | 23.95881081 | 23.74046898 | 23.89217377 | 23.89300728 |
|  | 1.207007289 | 0.1959095 | Q8K2Q7;A0A0A6YWK4;A0A0A6YWP0;A0A0A6YVS9 | BRO1 domain-containing protein BROX | Brox | 25.01184273 | 24.95925331 | 24.78949928 | 24.79014587 | 24.71779823 | 24.66492271 |
|  | 0.779875605 | 0.195939382 | P59108;A0A0R4J1D0;A0A1D5RLP0 | Copine-2 | Cpne2 | 22.25945663 | 21.92230606 | 22.09019661 | 21.98478508 | 21.92553329 | 21.77382278 |
|  | 1.111507361 | 0.195944468 | A0A2I3BRW0;Q9D958;A0A2I3BQG3 | Signal peptidase complex subunit 1 | Spcs1 | 24.4342804 | 24.45004082 | 24.63089752 | 24.32923508 | 24.39060593 | 24.20754433 |
|  | 1.113254638 | 0.196068446 | Q9CR16 | Peptidyl-prolyl cis-trans isomerase D | Ppid | 23.35622215 | 23.26670837 | 23.07985115 | 23.00805855 | 23.04966164 | 23.05685616 |
|  | 0.856104739 | 0.196877797 | Q78IK2 | Up-regulated during skeletal muscle growth protein 5 | Usmg5 | 24.71035576 | 24.7136631 | 24.57559776 | 24.65288734 | 24.43179703 | 24.32429886 |
|  | 1.484716094 | 0.197057088 | Q64433;Q9JI95 | 10 kDa heat shock protein, mitochondrial | Hspe1;Cpn10-rs1 | 27.05961418 | 27.1399498 | 27.09908676 | 27.01137352 | 26.8190937 | 26.87701225 |
|  | 0.63629146 | 0.197506587 | O35350;A0A494BAS0;A0A494BAC4 | Calpain-1 catalytic subunit | Capn1 | 23.20739555 | 23.10567093 | 23.26054955 | 23.2315464 | 22.9755249 | 22.77402496 |
|  | 1.150024314 | 0.198582331 | Q62318 | Transcription intermediary factor 1-beta | Trim28 | 26.05784035 | 25.99864769 | 25.95426941 | 25.91988945 | 25.83231544 | 25.66280556 |
|  | 1.20591218 | 0.19971784 | Q3TAI4;A2AUD5;Q9CYZ2;V9GWU5;Q3TUJ9;Q8BKP1;F6VQ81 | Tumor protein D54 | Tpd52l2 | 23.94055748 | 24.15753746 | 23.93966103 | 23.78256798 | 23.78646278 | 23.86957169 |
|  | 1.469760004 | 0.199786504 | Q8R5C5;E0CZD4;E0CYB4 | Beta-centractin | Actr1b | 23.83402824 | 23.85671425 | 23.80616951 | 23.54557228 | 23.60104942 | 23.75093079 |
|  | 1.600413722 | 0.200041453 | P42227;B7ZC18 | Signal transducer and activator of transcription 3;Signal transducer and activator of transcription | Stat3 | 23.37822151 | 23.40678406 | 23.50118065 | NaN | 23.22921562 | 23.22815895 |
|  | 1.468601747 | 0.200106939 | Q99JR1 | Sideroflexin-1 | Sfxn1 | 23.96013832 | 24.07855034 | 23.97254944 | 23.90380096 | 23.76860428 | 23.73851204 |
|  | 0.891667057 | 0.200110753 | Q8BU14 | Translocation protein SEC62 | Sec62 | 24.05779839 | 23.97394943 | 24.0484314 | 23.91205025 | 23.625 | 23.94279671 |
|  | 1.27241969 | 0.200433095 | Q9D6Z1;F7CHQ7;F6U250;F6V095;E0CXZ0;A2APD7;F7CHP9;F6USW7 | Nucleolar protein 56 | Nop56 | 23.86003304 | 23.69285393 | 23.77695847 | 23.58462906 | 23.47531128 | 23.6686058 |
|  | 1.43590536 | 0.200483958 | P14152;A0A5F8MPN8;B1ATQ3 | Malate dehydrogenase, cytoplasmic | Mdh1 | 27.10898781 | 27.19187737 | 27.00891876 | 26.89238167 | 26.84337997 | 26.97257042 |
|  | 2.18978404 | 0.201144536 | Q8K2C9 | Very-long-chain (3R)-3-hydroxyacyl-CoA dehydratase 3 | Hacd3 | 23.67820358 | 23.77152824 | 23.76981354 | 23.49435997 | 23.54898453 | 23.57276726 |
|  | 0.380416076 | 0.201642354 | Q8VH51;E9Q8F0;F7AA45;B7ZD61;B7ZD63 | RNA-binding protein 39 | Rbm39 | 23.28093338 | 23.10720444 | 23.10776329 | 23.34638023 | 22.60222816 | 22.94236565 |
|  | 0.951763127 | 0.201681137 | Q04750 | DNA topoisomerase 1 | Top1 | 22.55688667 | 22.527668 | 22.48049927 | 22.46667671 | 22.35568428 | 22.13764954 |
|  | 0.774081119 | 0.201682091 | Q8BVQ0;Q9ERF3;D6RDC7;D6RJ72 | WD repeat-containing protein 61;WD repeat-containing protein 61, N-terminally processed | Wdr61 | NaN | 23.42059708 | 23.38653374 | 23.29375839 | 23.03039169 | 23.28149986 |
|  | 1.358743102 | 0.201778412 | E0CZ22;D3Z624;E0CYC5 | Maestro heat-like repeat family member 1 | Mroh1 | 23.45287132 | 23.37623596 | 23.44644737 | 23.29249382 | 23.09368134 | 23.28404427 |
|  | 0.492355302 | 0.202075958 | Q8VDD8;A0A3B2WDA2 | WAS protein family homolog 1 | Wash1 | 22.89810944 | 23.3998909 | 22.95601273 | 23.00432587 | 22.72398758 | 22.91947174 |
|  | 0.77303909 | 0.202250163 | Q9ERE7;D3YVR4;F6SWV4 | LDLR chaperone MESD | Mesdc2 | 23.2132225 | 23.07520676 | 23.31658554 | 22.86846161 | 22.93820763 | 23.19159508 |
|  | 1.089196217 | 0.202388763 | Q9D0W5 | Peptidyl-prolyl cis-trans isomerase-like 1 | Ppil1 | 23.53302765 | 23.72348595 | 23.64801025 | 23.55894279 | 23.41041183 | 23.32800293 |
|  | 1.383534615 | 0.203261058 | Q62446;A0A1Y7VLK0;A0A1Y7VMJ9;A0A1Y7VP01;A0A1Y7VJ86 | Peptidyl-prolyl cis-trans isomerase FKBP3 | Fkbp3 | 23.4368248 | 23.21682358 | 23.39819527 | 23.13595963 | 23.13879013 | 23.16731071 |
|  | 1.364959309 | 0.203781128 | A0A1D5RLS1;Q60902 | Epidermal growth factor receptor substrate 15-like 1 | Eps15l1 | 22.41931152 | 22.56092453 | 22.4616642 | 22.35102654 | 22.31174469 | 22.16778564 |
|  | 1.970724387 | 0.20423762 | P62983;A0A0A6YW67;E9Q9J0;E9Q4P0;E9Q5F6;E9QNP0;Q5SX22;P62984;P0CG49;P0CG50 | Ubiquitin-40S ribosomal protein S27a;Ubiquitin;40S ribosomal protein S27a;Ubiquitin-60S ribosomal protein L40;Ubiquitin;60S ribosomal protein L40;Polyubiquitin-B;Ubiquitin;Polyubiquitin-C;Ubiquitin;Ubiquitin-related 1;Ubiquitin-related 2 | Rps27a;Gm8797;Uba52;Kxd1;Ubc;Ubb | 28.15508842 | 28.2352581 | 28.19788742 | 27.96587181 | 28.06825256 | 27.94139671 |
|  | 1.643853492 | 0.205352147 | Q6PB66;F6V2A3 | Leucine-rich PPR motif-containing protein, mitochondrial | Lrpprc | 25.05845833 | 24.98015404 | 25.14300919 | 24.9077034 | 24.79659653 | 24.86126518 |
|  | 0.452508609 | 0.205398242 | Q9CQU3 | Protein RER1 | Rer1 | 23.4506073 | 23.91743279 | 23.55051231 | 23.3824501 | 23.48578835 | NaN |
|  | 0.99718318 | 0.206013997 | P42125;A0A3Q4EC00;A0A452J8A5 | Enoyl-CoA delta isomerase 1, mitochondrial | Eci1 | 23.00075531 | 22.83454895 | 22.98844719 | 22.89683533 | 22.65678596 | 22.65208817 |
|  | 1.600983638 | 0.2067674 | Q91XD6 | Vacuolar protein-sorting-associated protein 36 | Vps36 | 23.01063919 | 22.80577469 | 22.91409683 | 22.70096016 | 22.70699883 | 22.70224953 |
|  | 2.059139411 | 0.207221349 | Q9EQH3 | Vacuolar protein sorting-associated protein 35 | Vps35 | 25.71053886 | 25.570858 | 25.6624527 | 25.45763779 | 25.41302872 | 25.45151901 |
|  | 1.076073956 | 0.207353592 | P48024 | Eukaryotic translation initiation factor 1 | Eif1 | 25.29073906 | 25.25349617 | 25.31295586 | 25.08731842 | 24.92011642 | 25.22769547 |
|  | 1.832168203 | 0.207397461 | Q8BVY0;A0A2R8VHW2 | Ribosomal L1 domain-containing protein 1 | Rsl1d1 | 23.38850594 | 23.44960022 | 23.35393524 | 23.19810104 | 23.25824928 | 23.11349869 |
|  | 0.538309982 | 0.208082835 | Q3TYL7;Q9QYJ3 | DnaJ homolog subfamily B member 1 | Dnajb1 | 21.71962738 | 21.42603493 | 21.16980553 | 21.14761925 | 21.19141388 | 21.3521862 |
|  | 2.030239537 | 0.209700267 | Q62087;H3BL07 | Serum paraoxonase/lactonase 3 | Pon3 | 25.22530746 | 25.25425339 | 25.12174797 | 24.95739555 | 24.9903183 | 25.02449417 |
|  | 0.340153669 | 0.209774017 | Q91VJ4;A0A3B2WD34;A0A3B2WAZ4;A0A3B2W7T8;A0A3B2WD06;A0A3B2W7M3;A0A3B2W7L2 | Serine/threonine-protein kinase 38 | Stk38 | 23.6866703 | 23.29977798 | 23.25078201 | 23.62878609 | 22.94642448 | 23.03269768 |
|  | 0.979519432 | 0.210024516 | P13439 | Uridine 5-monophosphate synthase;Orotate phosphoribosyltransferase;Orotidine 5-phosphate decarboxylase | Umps | 23.05692291 | 22.81540489 | 23.11093712 | 22.81111717 | 22.70036697 | 22.84170723 |
|  | 0.567228196 | 0.210927327 | D3YUV1;Q99J45;A0A0J9YUZ6;A0A0J9YTX8;A0A0J9YUQ4 | Nuclear receptor-binding protein | Nrbp1 | 21.95921898 | 22.41066933 | 22.11111069 | 21.81041145 | 22.13999176 | 21.8978138 |
|  | 1.991490756 | 0.211403529 | Q9DCN2;F2Z456 | NADH-cytochrome b5 reductase 3;NADH-cytochrome b5 reductase 3 membrane-bound form;NADH-cytochrome b5 reductase 3 soluble form;NADH-cytochrome b5 reductase | Cyb5r3 | 26.39473343 | 26.48914719 | 26.41701126 | 26.17142487 | 26.29263496 | 26.20262146 |
|  | 1.461439388 | 0.212315241 | D3YTQ9;P62843 | 40S ribosomal protein S15 | Rps15 | 26.22589493 | 26.1193161 | 26.0914402 | 26.0397377 | 25.88987732 | 25.87009048 |
|  | 1.032828909 | 0.212452571 | Q61206;A0A1L1SVK0;A0A1L1SRD0;A0A1L1SQ76;A0A1L1SQV7;A0A1L1SSQ5 | Platelet-activating factor acetylhydrolase IB subunit beta | Pafah1b2 | 24.01290894 | 24.21267319 | 24.13735199 | 24.02512932 | 23.76293945 | 23.93750763 |
|  | 1.279682236 | 0.212514877 | Q9DCL9;D3Z6P1;D6RCU8 | Multifunctional protein ADE2;Phosphoribosylaminoimidazole-succinocarboxamide synthase;Phosphoribosylaminoimidazole carboxylase | Paics | 24.31713676 | 24.46662521 | 24.42181778 | 24.25039101 | 24.06126404 | 24.25638008 |
|  | 0.784651817 | 0.213202794 | O35857 | Mitochondrial import inner membrane translocase subunit TIM44 | Timm44 | 22.28305626 | 22.24263573 | 22.36559677 | 22.19973373 | 21.84391212 | 22.20803452 |
|  | 0.302497684 | 0.213232994 | Q9JJA4;D3Z369 | Ribosome biogenesis protein WDR12 | Wdr12 | NaN | 22.1853466 | 22.70482635 | NaN | 22.22057724 | 22.24312973 |
|  | 0.754289186 | 0.213680903 | Q9CQS8;E9PW43 | Protein transport protein Sec61 subunit beta | Sec61b;Gm10320 | 25.20396423 | 25.25533485 | 25.0377903 | 24.76106262 | 25.15031052 | 24.94467354 |
|  | 0.84800414 | 0.213868777 | Q9D0B6 | Protein PBDC1 | Pbdc1 | 23.31506538 | 23.19535446 | 23.13166428 | 23.11109543 | NaN | 22.8892231 |
|  | 1.423684439 | 0.214581807 | Q99L13 | 3-hydroxyisobutyrate dehydrogenase, mitochondrial | Hibadh | 23.67605209 | 23.60172844 | 23.46288681 | 23.33183289 | 23.33565331 | 23.42943573 |
|  | 1.443686104 | 0.215122223 | Q9D2M8;A6X925;B2KF55 | Ubiquitin-conjugating enzyme E2 variant 2 | Ube2v2 | 23.47716713 | 23.29529953 | 23.3754406 | 23.21297073 | 23.21209526 | 23.07747459 |
|  | 1.887800649 | 0.215295156 | Q6ZQ38;D3YWC5 | Cullin-associated NEDD8-dissociated protein 1 | Cand1 | 24.02208328 | 24.06997299 | 24.10085297 | 23.86192703 | 23.76557159 | 23.91952515 |
|  | 1.051476939 | 0.215770086 | Q5SSZ5;A0A5F8MP98;A0A5F8MPF2;A0A2R8VHQ0;Q8CGB6 | Tensin-3 | Tns3 | 23.75755692 | 23.981287 | 24.05250168 | 23.66046906 | 23.6971035 | 23.78646278 |
|  | 0.800077725 | 0.216241837 | Q8C872;Q62351 | Transferrin receptor protein 1 | Tfrc | 22.86894989 | 22.59848404 | 22.78805542 | 22.69031906 | 22.55609131 | 22.36035347 |
|  | 0.825679927 | 0.216257731 | Q9EPQ7;D3YU00 | StAR-related lipid transfer protein 5 | Stard5 | 22.15758324 | 22.34975815 | 22.16462898 | 22.11146164 | NaN | 21.90400314 |
|  | 0.961436731 | 0.218149185 | P61979;H3BLL4;A0A286YDM3;H3BK96;A0A286YCM2;A0A286YEC4;A0A286YDH1 | Heterogeneous nuclear ribonucleoprotein K | Hnrnpk | 24.52957726 | 24.71471024 | 24.80833054 | 24.39139366 | 24.40561485 | 24.60116196 |
|  | 1.025109823 | 0.218440374 | Q9CQ62 | 2,4-dienoyl-CoA reductase, mitochondrial | Decr1 | 23.79263115 | 23.9368782 | 23.96208191 | 23.66514015 | 23.83229065 | 23.53883934 |
|  | 0.837795359 | 0.219443639 | O70194;A0A2R8VK20 | Eukaryotic translation initiation factor 3 subunit D | Eif3d | 24.3801384 | 24.31271362 | 24.46998215 | 24.19947815 | 23.95925331 | 24.34577179 |
|  | 0.47634872 | 0.220430374 | A0A0R4J0Q5;P21619 | Lamin-B2 | Lmnb2 | 22.48402023 | 22.77058029 | 22.1524868 | 22.42539597 | 22.11746597 | 22.20293427 |
|  | 1.429570967 | 0.220493317 | Q91YR1;D3Z2H0 | Twinfilin-1 | Twf1 | 24.43587112 | 24.32477951 | 24.26391792 | 24.03271484 | 24.20985031 | 24.12052345 |
|  | 1.911139779 | 0.221022288 | P43275 | Histone H1.1 | Hist1h1a | 26.00994301 | 26.02405167 | 26.06597519 | 25.90065002 | 25.73520851 | 25.80104446 |
|  | 1.371507585 | 0.221100489 | A0A1B0GS58;Q9CQM9;A0A1B0GT04;A0A1B0GSD3 | Glutaredoxin-3 | Glrx3 | 24.32278633 | 24.35917473 | 24.46151352 | 24.0777359 | 24.28333855 | 24.11909866 |
|  | 0.781429909 | 0.221316338 | Q8K021;Q3TSA8;D3YTP4 | Secretory carrier-associated membrane protein 1 | Scamp1 | 23.56337166 | NaN | 23.45663452 | 23.13509941 | 23.44240379 | 23.28855705 |
|  | 0.663513951 | 0.221557617 | Q9Z1F9;H3BLM2;H3BLR3;A2BH29 | SUMO-activating enzyme subunit 2 | Uba2 | 23.51135254 | 23.27056313 | 23.60941505 | 23.33019257 | 23.01797867 | 23.37848663 |
|  | 0.532888541 | 0.221749624 | Q9D554 | Splicing factor 3A subunit 3 | Sf3a3 | NaN | 22.40080261 | 22.82774353 | 22.40654945 | 22.29049873 | 22.48052216 |
|  | 1.224725369 | 0.222305934 | Q3UPH1 | Protein PRRC1 | Prrc1 | 23.7135582 | 23.80469322 | 23.61571312 | 23.37967682 | 23.48111534 | 23.60625458 |
|  | 0.592608335 | 0.223151525 | Q6P9R2 | Serine/threonine-protein kinase OSR1 | Oxsr1 | 23.67529869 | 23.63555527 | 23.55156898 | 23.69285393 | 23.12507248 | 23.37504196 |
|  | 0.965660935 | 0.223445892 | Q9DB15 | 39S ribosomal protein L12, mitochondrial | Mrpl12 | 24.35944366 | 24.18918228 | 24.30493927 | 24.06266403 | 23.89393234 | 24.22663116 |
|  | 1.30387715 | 0.224309285 | Q9D023;A0A0A6YY89;G5E869 | Mitochondrial pyruvate carrier 2 | Mpc2;Zfp142 | 23.15950966 | 23.06863022 | 23.30632973 | 22.92232323 | 23.0356369 | 22.90358162 |
|  | 1.233727778 | 0.224884669 | P51807;A0A338P7B7 | Dynein light chain Tctex-type 1 | Dynlt1 | 24.55015945 | 24.43841171 | 24.46749687 | 24.31236649 | 24.10565567 | 24.36339188 |
|  | 1.622786652 | 0.225052516 | Q91YS7;Q63932;M0QWN2;A0A1W2P7V9 | Dual specificity mitogen-activated protein kinase kinase 2 | Map2k2 | 22.82975769 | 22.93610573 | 22.80112457 | 22.72081757 | 22.5553894 | 22.61562347 |
|  | 1.215068919 | 0.226015727 | P48410;A2ALN0 | ATP-binding cassette sub-family D member 1 | Abcd1 | 22.90767479 | 22.94899178 | 23.0953083 | 22.79034615 | 22.63038635 | 22.85319519 |
|  | 1.387359592 | 0.226593653 | Q6P5F9;F6YA11;A2AKT6 | Exportin-1 | Xpo1 | 23.41776848 | 23.51195526 | 23.29080772 | 23.24433517 | 23.1931591 | 23.10325623 |
|  | 0.589946944 | 0.226996104 | Q91WC9 | Sn1-specific diacylglycerol lipase beta | Daglb | 22.70843124 | 22.79161835 | 22.91987228 | 22.27227402 | 22.65326881 | 22.81339073 |
|  | 1.585018316 | 0.227039337 | Q61937;Q5SQB0;Q9DAY9;Q5SQB5 | Nucleophosmin | Npm1 | 26.80648804 | 26.98239708 | 26.97945595 | 26.64663887 | 26.68832588 | 26.7522583 |
|  | 1.307065775 | 0.228302002 | P32921 | Tryptophan--tRNA ligase, cytoplasmic;T1-TrpRS;T2-TrpRS | Wars | 23.88026237 | 24.10988426 | 24.11036301 | 23.78994751 | 23.86079216 | 23.76486397 |
|  | 0.685732309 | 0.228412628 | P50427 | Steryl-sulfatase | Sts | 23.2148838 | 23.31215858 | 23.23178101 | 23.32154846 | 22.87988853 | 22.87214851 |
|  | 0.719186332 | 0.228942871 | P52825;A2A8E7;A2A8E8;A2A8E9 | Carnitine O-palmitoyltransferase 2, mitochondrial | Cpt2 | 22.11034775 | 21.7743454 | 22.150877 | 21.61629677 | 21.87237358 | 21.86007118 |
|  | 0.778624153 | 0.229224523 | Q8BKZ9;A2AWH8;A2AWH7 | Pyruvate dehydrogenase protein X component, mitochondrial | Pdhx | 22.59998322 | 22.58691978 | 22.85997772 | 22.47231483 | 22.62024117 | 22.26665115 |
|  | 1.157919703 | 0.229316711 | O09117 | Synaptophysin-like protein 1 | Sypl1 | 25.61885262 | 25.61607933 | NaN | 25.3719902 | 25.50636673 | 25.28609085 |
|  | 1.14692595 | 0.230458577 | P34884 | Macrophage migration inhibitory factor | Mif | 28.23036385 | 28.47869492 | 28.25092506 | 27.99183273 | 28.11321831 | 28.16355705 |
|  | 1.693613241 | 0.23204422 | Q9WV85 | Nucleoside diphosphate kinase 3 | Nme3 | 22.89352608 | 22.87071991 | 23.03266335 | 22.64216042 | 22.76642227 | 22.69219398 |
|  | 0.486994936 | 0.232344309 | Q921M3 | Splicing factor 3B subunit 3 | Sf3b3 | 23.40989304 | 23.66101265 | 24.10685349 | 23.5731144 | 23.45838928 | 23.44922256 |
|  | 0.946970832 | 0.23330307 | P25206 | DNA replication licensing factor MCM3 | Mcm3 | 22.78931046 | 23.05072594 | 22.99717522 | 22.54686737 | 22.81050873 | 22.7799263 |
|  | 0.546858097 | 0.233303706 | Q8BTX9;A0A1D5RLG0;A0A1D5RM62 | Inactive hydroxysteroid dehydrogenase-like protein 1 | Hsdl1 | 23.15622711 | 23.15017891 | 22.74314308 | 22.74147606 | NaN | 22.82494926 |
|  | 1.211134506 | 0.233880361 | Q8VEE0;B2KGF0;M0QWJ2;A0A087WQM3;M0QWQ0 | Ribulose-phosphate 3-epimerase | Rpe | 23.72910118 | 23.96895027 | 23.79728889 | 23.49399376 | 23.68634987 | 23.61335564 |
|  | 1.238961018 | 0.234695435 | P62315;A0A3Q4L381;A0A3Q4L2W0 | Small nuclear ribonucleoprotein Sm D1 | Snrpd1 | 24.79243279 | 24.59183502 | 24.83975792 | 24.42898941 | 24.58897972 | 24.50197029 |
|  | 0.591194608 | 0.234824498 | Q99J83 | Autophagy protein 5 | Atg5 | 23.29712105 | 22.84879112 | 23.41544914 | 23.03007317 | 22.93779564 | 22.88901901 |
|  | 1.707156309 | 0.235397339 | P62082 | 40S ribosomal protein S7 | Rps7 | 26.37303543 | 26.28467941 | 26.31018448 | 25.97458458 | 26.13267326 | 26.15444946 |
|  | 0.69121313 | 0.236155192 | A0A286YDA2;A0A286YDT3;E9Q5C9;A0A286YDV7 |  | Nolc1 | 21.52955246 | 21.62725067 | 21.33226967 | NaN | 21.13508224 | 21.38532257 |
|  | 1.167206917 | 0.237531026 | Q9D8Y7 | Tumor necrosis factor alpha-induced protein 8-like protein 2 | Tnfaip8l2 | 24.11688042 | 24.10900879 | 23.84440994 | 23.77404404 | 23.8493824 | 23.73427963 |
|  | 0.333614639 | 0.238235156 | Q9R1Q9;Q3TKX1;B7FAU3;F6ZE56;F6X9J0;B7FAU7 | V-type proton ATPase subunit S1 | Atp6ap1 | 23.25551414 | NaN | 22.7095871 | 22.45259476 | 22.97603226 | 22.80431938 |
|  | 0.36648211 | 0.238374074 | A0A0G2JFT8;Q9D394 | Protein RUFY3 | Rufy3 | 23.03567123 | 23.04159737 | 23.03610611 | 22.91557503 | 23.20129013 | 22.28138733 |
|  | 1.214725585 | 0.24016126 | Q91YH5;E9PYT3;A0A494BAX8;A0A494BAU1;A0A494BAW9;A0A494B9P1;Q8BH66 | Atlastin-3 | Atl3 | 24.29375839 | 24.19587898 | 24.1791172 | 23.83855629 | 23.97447586 | 24.13523865 |
|  | 0.959574459 | 0.24041748 | Q8BJS4;E0CY39 | SUN domain-containing protein 2 | Sun2 | 21.99772644 | 21.87533951 | 21.66453171 | 21.57316017 | 21.73156738 | 21.51161766 |
|  | 1.171570256 | 0.242008209 | Q8K157;F6XWR4 | Aldose 1-epimerase | Galm | 23.16361618 | 23.1368351 | 23.2854557 | 22.95891762 | 23.09881401 | 22.80215073 |
|  | 0.510648865 | 0.242311478 | A0A494B9X3;E9Q9M1;Q3V1L4;A0A494BBP6;G3X9J6;A0A494BBK9;A0A494BAU4;A0A494BAN2;A0A494BBI8;A0A494BBM7 | Cytosolic purine 5-nucleotidase | Nt5c2 | 22.96786118 | 22.7614994 | 22.62586975 | 22.23271751 | 22.86485863 | 22.53071976 |
|  | 0.930339535 | 0.243316015 | P50544;B1AR28 | Very long-chain specific acyl-CoA dehydrogenase, mitochondrial | Acadvl | 23.51520729 | 23.80823326 | 23.73902702 | 23.27540207 | 23.53183746 | 23.52528 |
|  | 0.879603578 | 0.243674596 | P40240 | CD9 antigen | Cd9 | 25.49148941 | 25.40421867 | 25.45098495 | 25.06270599 | 25.09538841 | 25.45757484 |
|  | 0.526883183 | 0.243798574 | P31750;D3Z783;D3YXX3;D3YYP9;Q9WUA6 | RAC-alpha serine/threonine-protein kinase | Akt1 | 22.68093109 | 23.20354652 | 22.66290474 | 22.66576958 | 22.40979004 | 22.74042702 |
|  | 1.358004359 | 0.247168223 | Q6PAH4;Q61124;D3Z448;D3YUU4 | Battenin | Cln3 | 21.99538231 | 22.11495972 | 22.05982971 | 21.67585945 | 21.80774117 | 21.94506645 |
|  | 0.637321354 | 0.247517268 | Q99LE6 | ATP-binding cassette sub-family F member 2 | Abcf2 | 22.88444328 | 22.62272263 | 22.56890106 | 22.17295074 | 22.48949242 | 22.67107201 |
|  | 1.552313419 | 0.24804306 | Q8R5J9 | PRA1 family protein 3 | Arl6ip5 | 24.76592636 | 24.82036018 | 24.62215233 | 24.5552597 | 24.50330544 | 24.40574455 |
|  | 0.317563332 | 0.248290062 | O70378;M0QWP2;M0QWY0;M0QWS0;M0QWI7;M0QWE2;M0QWC9 | ER membrane protein complex subunit 8 | Emc8 | 23.62800789 | NaN | 23.19464874 | 23.35487747 | NaN | 22.97119904 |
|  | 0.8993174 | 0.249536514 | Q9QYR9;O55137;Q32Q92;Q8BWN8;A0A1Y7VMZ4;Q91YQ6;Q6Q2Z6;Q9QYR7 | Acyl-coenzyme A thioesterase 2, mitochondrial;Acyl-coenzyme A thioesterase 1 | Acot2;Acot1 | 23.50081635 | 23.61481667 | 23.46936226 | 23.1989994 | 23.1196537 | 23.51773262 |
|  | 1.168906196 | 0.249710083 | Q6SJQ0 | CMRF35-like molecule 8 | Cd300a | 23.17185211 | 23.21949959 | 23.01991463 | 22.72826958 | 22.94647789 | 22.98738861 |
|  | 0.561150004 | 0.249716441 | A0A2R8VI07;Q8C6B0;H3BJI7;A0A2R8VK72;Q9D7S5;Q76I24;Q5I0W6;Q76I26;A0A2R8VHN2;G3X9G9 | Methyltransferase like 7A1 | Mettl7a1;Methig1;mCG_20149;UbiE2;Mettl7a2 | 24.09176254 | 23.46811867 | 23.99040413 | 23.57484818 | 23.5460434 | 23.68024445 |
|  | 1.48239525 | 0.25073115 | E9PZF0;Q01768 | Nucleoside diphosphate kinase;Nucleoside diphosphate kinase B | Gm20390;Nme2 | 28.85290337 | 28.75627327 | 28.9600544 | 28.70417976 | 28.52883911 | 28.58401871 |
|  | 0.801799806 | 0.25389417 | Q9D172;A0A1W2P7B6;A0A1W2P870 | ES1 protein homolog, mitochondrial | D10Jhu81e | 24.41583633 | 24.41370773 | 24.29221344 | 24.31561852 | 23.84737587 | 24.19708061 |
|  | 1.362222588 | 0.254025141 | P54775;A0A140LIZ5 | 26S protease regulatory subunit 6B | Psmc4 | 24.20433807 | 24.42867088 | 24.34360504 | 23.97788429 | 24.06027412 | 24.17638016 |
|  | 0.879055609 | 0.25505956 | E9PVA8 | Stalled ribosome sensor GCN1 | Gcn1l1 | 23.49753189 | 23.19010162 | 23.61930084 | 23.14142799 | 23.1316185 | 23.26870918 |
|  | 0.65216669 | 0.25559934 | P45878 | Peptidyl-prolyl cis-trans isomerase FKBP2 | Fkbp2 | 24.93557549 | 24.8802166 | 25.02462196 | 24.34881401 | 24.83040619 | 24.89439583 |
|  | 1.203059928 | 0.256868998 | Q8CBB7;P22892 | AP-1 complex subunit gamma-1 | Ap1g1 | 23.02445221 | 23.32991982 | 23.18161964 | 22.87843132 | 22.86955261 | 23.01740074 |
|  | 0.912188255 | 0.257254918 | Q9D1K2;A0A0N4SVE1;F7B2B4 | V-type proton ATPase subunit F | Atp6v1f | 24.72134972 | 24.83238792 | 24.71863556 | 24.51424408 | 24.71145821 | 24.27490616 |
|  | 1.413070929 | 0.259377797 | O35114 | Lysosome membrane protein 2 | Scarb2 | 24.23819542 | 24.13891411 | 24.15876961 | 24.0294323 | 23.96446228 | 23.76385117 |
|  | 1.397100401 | 0.260620753 | P50396;B7FAU8;D6RI86 | Rab GDP dissociation inhibitor alpha | Gdi1 | 23.8496685 | 23.8735199 | 24.02250671 | 23.53385925 | 23.76961327 | 23.66036034 |
|  | 1.420784404 | 0.261845271 | Q9CR51 | V-type proton ATPase subunit G 1 | Atp6v1g1 | 26.61301804 | 26.60823059 | 26.56556702 | 26.40094948 | 26.43437576 | 26.16595459 |
|  | 1.462733465 | 0.26230971 | A0A087WNZ7;G5E870;A0A087WP92;A0A087WRV6;A0A0B4J1N9;A0A087WS65;Q3TP48;A0A087WSG4;A0A087WQ02 | E3 ubiquitin-protein ligase TRIP12 | Trip12 | 22.16922379 | NaN | 22.01699257 | 21.88338089 | 21.76963234 | 21.83938217 |
|  | 1.139555944 | 0.262912114 | Q8R180;A0A2I3BPM1;A0A1Y7VJM4;A0A1Y7VNF4;Q8R2E9 | ERO1-like protein alpha | Ero1l | 23.2990799 | 23.51376343 | 23.20605469 | 23.19190979 | 22.99764061 | 23.04061127 |
|  | 1.292526942 | 0.264108658 | Q8JZR0;A0A286YCG4;A0A286YD68 | Long-chain-fatty-acid--CoA ligase 5 | Acsl5 | 22.70450974 | 22.71290779 | 22.85382271 | 22.32802963 | 22.58451462 | 22.56637001 |
|  | 0.807550956 | 0.265053749 | Q03958;G3UYF9;A0A3Q4EBT3 | Prefoldin subunit 6 | Pfdn6 | 23.43491745 | 23.46737289 | NaN | 23.30396271 | 23.06822014 | NaN |
|  | 1.314799953 | 0.265753428 | Q3TE85;A0A338P6F6;P17095 | High mobility group protein HMG-I/HMG-Y | Hmga1 | 25.10817146 | 25.10333633 | 24.96265411 | 24.81509209 | 24.92111588 | 24.64069366 |
|  | 0.811344067 | 0.267115275 | Q8BFQ8 | Parkinson disease 7 domain-containing protein 1 | Pddc1 | 23.13397026 | 23.23666382 | 22.93081093 | 22.72594261 | NaN | 22.94079018 |
|  | 1.230117214 | 0.267832438 | F7D432;E9PWE0;E0CYV0;P23506;F6TXE3;F6V9F1 | Protein-L-isoaspartate O-methyltransferase;Protein-L-isoaspartate(D-aspartate) O-methyltransferase | Pcmt1 | 24.99670982 | 25.09329414 | 25.02989578 | 24.82201576 | 24.5825634 | 24.91182327 |
|  | 0.473618336 | 0.268934886 | D5MCW4;Q9CQ89 | Protein CutA | Cuta | 22.82211304 | 22.83618736 | 23.55613708 | 22.78242874 | 22.73983002 | 22.88537407 |
|  | 0.978909377 | 0.269528707 | Q9DCM0 | Persulfide dioxygenase ETHE1, mitochondrial | Ethe1 | 22.97032166 | 23.09274673 | 23.15262604 | 23.03288269 | 22.72538185 | 22.64884377 |
|  | 1.222966903 | 0.270118395 | Q8K4M5;F7BCN0;F7BZY0;Q8VI86;G8JL54 | COMM domain-containing protein 1 | Commd1;Gm28048 | 22.69321632 | 22.90181541 | 22.85940742 | 22.49419022 | NaN | 22.60186577 |
|  | 1.27658376 | 0.270470937 | P97742;A0A494BAD3;A0A494BAC7 | Carnitine O-palmitoyltransferase 1, liver isoform | Cpt1a | 23.93903351 | 24.11370468 | 24.11553192 | 23.74211502 | 23.67346764 | 23.94127464 |
|  | 0.957555742 | 0.272077243 | Q9Z2L7;J3QMV5 | Cytokine receptor-like factor 3 | Crlf3 | 22.42879677 | 22.3996563 | 22.64937019 | NaN | 22.13248062 | 22.3085804 |
|  | 0.940399274 | 0.272364299 | P26883;F6X9I3 | Peptidyl-prolyl cis-trans isomerase FKBP1A | Fkbp1a | 26.06570816 | 25.98680687 | 25.97969627 | 25.66662979 | 25.99539566 | 25.55309296 |
|  | 0.93349502 | 0.274911245 | Q8VE99 | Coiled-coil domain-containing protein 115 | Ccdc115 | 23.8233757 | 23.62544632 | NaN | 23.48812103 | 23.56255722 | 23.29782104 |
|  | 1.244291629 | 0.275855382 | P04117;A0A0A6YW05;A0A0A6YXB9;A0A0A6YXI2;P24526;O08716 | Fatty acid-binding protein, adipocyte | Fabp4 | 24.43510818 | 24.73458862 | 24.41299629 | 24.23724747 | 24.26806641 | 24.24981308 |
|  | 0.709902921 | 0.277043025 | Q8VDK1;D3YY53;D3Z2Y2;D3Z3I3 | Nitrilase homolog 1 | Nit1 | 22.22429085 | 22.25796127 | 22.40452385 | 22.31118965 | 21.72434044 | 22.02011681 |
|  | 1.376256165 | 0.279506048 | P23591;A0A2R8VI39;A0A2R8VHD0;A0A2R8W6P6;A0A2R8VKL9;A0A2R8W6N0 | GDP-L-fucose synthase | Tsta3 | 23.73013687 | 23.67012024 | 23.74601555 | 23.5666256 | 23.48259163 | 23.25853729 |
|  | 1.368179307 | 0.279689789 | Q91X76;A0A2I3BQR1;A0A2I3BRL0;A0A2I3BR81;A0A2I3BPH8 |  | Nt5dc2 | 22.3303299 | 22.30524445 | 22.15619469 | 21.84774017 | 22.1196537 | 21.98530579 |
|  | 1.135265214 | 0.281568527 | P49962;D3YZX8 | Signal recognition particle 9 kDa protein | Srp9 | 24.13469124 | NaN | 23.99533081 | NaN | 23.8241539 | 23.74273109 |
|  | 1.279678434 | 0.281819979 | Q8BHS6 | Armadillo repeat-containing X-linked protein 3 | Armcx3 | 23.09413338 | 22.87408257 | 22.93714905 | 22.64354706 | 22.72972298 | NaN |
|  | 1.065767208 | 0.282353719 | Q6ZQ58;Z4YJT3;Q9D423 | La-related protein 1 | Larp1 | 22.40457535 | 22.31862831 | 22.65802956 | 22.04423523 | 22.20090103 | 22.2890358 |
|  | 1.233056831 | 0.28506724 | P09671;A0A3B2WBF0 | Superoxide dismutase [Mn], mitochondrial | Sod2 | 25.47877121 | 25.6159668 | 25.82970428 | 25.28376198 | 25.4043808 | 25.38109779 |
|  | 0.489488965 | 0.285586039 | Q7TT50;A0A1Y7VLI0 | Serine/threonine-protein kinase MRCK beta | Cdc42bpb | 22.59137917 | 22.92784882 | 23.06536293 | 22.29283142 | 22.44518471 | 22.98981667 |
|  | 0.96260244 | 0.287876765 | B2RUP2;A0A0R4J257;A2A855;A2A858 | Protein unc-13 homolog D | Unc13d | 21.31848907 | 21.4453125 | 21.22823143 | 21.17444611 | 20.9111557 | NaN |
|  | 0.809941128 | 0.290410995 | A0A1W2P7C8;A0A0F6AIX5;A0A0F6AIX6;Q8BVA5;A0A1W2P7T7;A0A1W2P820;A0A1W2P6L7;A0A1W2P818;A0A1W2P6J8;A0A1W2P6H0 | UPF0554 protein C2orf43 homolog |  | NaN | 23.06982613 | 23.31934547 | NaN | 22.94037819 | 22.86797142 |
|  | 0.9813209 | 0.293792725 | E9Q1S3;Q01405 | Protein transport protein Sec23A | Sec23a | 22.57697105 | 22.50830841 | 22.53850746 | 22.20368195 | 22.50695419 | 22.03177261 |
|  | 0.940695364 | 0.296627045 | Q91V61;Q3U4F0;A0A494BB84 | Sideroflexin-3 | Sfxn3 | 24.75450325 | 24.53552055 | 24.52796745 | 24.06882668 | 24.35682678 | 24.50245667 |
|  | 0.614484063 | 0.296771049 | Q91WD5;D3YXT0;A0A0A6YW30 | NADH dehydrogenase [ubiquinone] iron-sulfur protein 2, mitochondrial | Ndufs2 | 22.88678741 | NaN | 22.88792229 | NaN | 22.77168846 | 22.40947914 |
|  | 1.296917513 | 0.296823502 | Q5KU39 | Vacuolar protein sorting-associated protein 41 homolog | Vps41 | 22.86580276 | 23.08551979 | 22.95650864 | 22.5933857 | 22.57918167 | 22.84479332 |
|  | 1.20529432 | 0.298354467 | P60670 | Nuclear protein localization protein 4 homolog | Nploc4 | 23.55309296 | 23.34055138 | 23.47803116 | 23.23835564 | 22.96342278 | 23.27483368 |
|  | 1.151198669 | 0.299741745 | A0A0R4J092;Q8K2I4;D6RGR1 | Beta-mannosidase | Manba | 22.55760956 | 22.82890511 | 22.74057198 | 22.45332336 | 22.54340172 | 22.23113632 |
|  | 0.864310996 | 0.302441597 | A0A087WNM1;Q91Z67;A0A087WRV4;A0A087WSQ1;A0A087WS59;D3YZW1;Q91Z69;A0A087WNR5 | SLIT-ROBO Rho GTPase-activating protein 2 | Srgap2 | 22.97465134 | 22.7853241 | 23.12918282 | 22.55718994 | NaN | 22.76403236 |
|  | 1.27316066 | 0.303063711 | Q9D1L9;G3UW70 | Ragulator complex protein LAMTOR5 | Lamtor5 | 24.85604858 | 24.64113426 | 24.74995995 | 24.28940201 | 24.61071014 | 24.43783951 |
|  | 1.130042029 | 0.307266871 | Q9QXK3 | Coatomer subunit gamma-2 | Copg2 | 22.70400238 | 22.42854309 | 22.77368164 | 22.45390129 | 22.20293427 | 22.32759094 |
|  | 1.094138301 | 0.308328311 | O35841 | Apoptosis inhibitor 5 | Api5 | 22.91301918 | 22.74457932 | NaN | 22.67473793 | 22.42308807 | 22.46358681 |
|  | 0.829538042 | 0.311346054 | P16254;A2AUM6 | Signal recognition particle 14 kDa protein;Signal recognition particle 14 kDa protein, N-terminally processed | Srp14 | NaN | 23.34556961 | 22.95009613 | 22.82797623 | 22.75515556 | 22.92632866 |
|  | 0.870721917 | 0.314207713 | Q9EQ06;A8Y5N4 | Estradiol 17-beta-dehydrogenase 11 | Hsd17b11 | 24.73541641 | 24.74985695 | 24.72961998 | 24.61762047 | 24.08982468 | 24.56482506 |
|  | 0.427756759 | 0.315501213 | Z4YKT6;Q99J47 | Dehydrogenase/reductase SDR family member 7B | Dhrs7b | 22.77352142 | 22.44182205 | NaN | NaN | 22.06966209 | 22.51467896 |
|  | 1.050920367 | 0.31738917 | P97823;J3QP56;D3YUG4;D3Z111;D3Z269;J3QQ63 | Acyl-protein thioesterase 1 | Lypla1 | 23.72723198 | 24.09248924 | 24.14211273 | 23.58210373 | 23.77112579 | 23.65643692 |
|  | 0.979780199 | 0.319373449 | Q60931;J3QMG3 | Voltage-dependent anion-selective channel protein 3 | Vdac3 | 25.01767349 | 25.47360992 | 25.43080902 | 24.89910698 | 25.00611687 | 25.05874825 |
|  | 0.573881205 | 0.320005417 | A0A494BAB5;Q9D5T0;A0A494B9L7;A0A494B9Y0 | ATPase family AAA domain-containing protein 1 | Atad1 | 21.66916847 | 21.43002319 | NaN | 21.0571537 | 21.40202713 | NaN |
|  | 1.183831463 | 0.329852422 | Q91WC0;F2Z420;F2Z438;D6RCY6 | Histone-lysine N-methyltransferase setd3 | Setd3 | 22.09741592 | 21.98735428 | 22.32542419 | 21.7072506 | 21.97627831 | 21.73710823 |
|  | 0.284597186 | 0.334622383 | A0A1D5RLS2;A0A1D5RM23;Q9CQF3;A0A1D5RLT7 | Cleavage and polyadenylation specificity factor subunit 5 | Nudt21 | NaN | 23.34921837 | 23.4124794 | 22.61578178 | NaN | 23.47667122 |
|  | 1.055435076 | 0.334648768 | P00375 | Dihydrofolate reductase | Dhfr | 22.87479591 | 23.1982193 | 22.95939445 | 22.74336815 | 22.4552803 | 22.82981491 |
|  | 0 | 0.353340785 | Q8JZN5;A0A0G2JDY4;A0A0G2JF25 | Acyl-CoA dehydrogenase family member 9, mitochondrial | Acad9 | 23.20069122 | 23.21776962 | 23.27241516 | NaN | 22.87695122 | NaN |
|  | 0 | 0.355764389 | Q9D6U8 | Protein FAM162A | Fam162a | NaN | NaN | 22.61649895 | 22.09126282 | 22.38205338 | 22.30888748 |
|  | 1.043691937 | 0.35683314 | Q9EQG9 | Collagen type IV alpha-3-binding protein | Col4a3bp | 22.3126297 | 22.62714005 | 22.41846275 | 21.9859314 | NaN | 22.20589066 |
|  | 0.999389898 | 0.358181636 | Q80X90 | Filamin-B | Flnb | 22.54872513 | 22.64989662 | 22.68221855 | 21.96073914 | 22.51645851 | 22.32909775 |
|  | 1.161101892 | 0.359971364 | Q5SUS9;Q61545;Q5SUT0;Q5SUS8 | RNA-binding protein EWS | Ewsr1 | 24.48332977 | 24.13351631 | 24.08553505 | 23.72546387 | 23.94333267 | 23.9536705 |
|  | 0.523171233 | 0.368540764 | A2A7S8 | Uncharacterized protein KIAA1522 | Kiaa1522 | 22.45766258 | 22.12707329 | 21.68260384 | 21.64912987 | 21.79201508 | NaN |
|  | 0.952487187 | 0.374789556 | Q9D2N9;A0A0G2JEL2 | Vacuolar protein sorting-associated protein 33A | Vps33a | 22.89126396 | 23.10899162 | 23.44997787 | 22.70410919 | 22.94740486 | 22.67435074 |
|  | 0.530681635 | 0.378225962 | E9Q1J7;Q99MN9;D3YZC1;A0A087WQV1 | Propionyl-CoA carboxylase beta chain, mitochondrial | Pccb | 23.15305901 | 23.15313721 | 23.27042007 | 22.32198906 | 23.39086914 | 22.7290802 |
|  | 1.0051378 | 0.382235845 | A0A0G2JEK2;P63254 | Cysteine-rich protein 1 | Crip1 | 24.51641083 | 24.98028374 | 24.71769524 | 24.15228462 | 24.55917549 | 24.35622215 |
|  | 0.746571061 | 0.39181455 | Q9CQH7 | Transcription factor BTF3 homolog 4 | Btf3l4 | 24.8191433 | 24.33080864 | 24.3698616 | 24.44442749 | 24.0878849 | 23.8120575 |
|  | 1.070767858 | 0.395723025 | Q3UGR5 | Haloacid dehalogenase-like hydrolase domain-containing protein 2 | Hdhd2 | 23.77735901 | NaN | 23.57126427 | 23.19479752 | 23.15333748 | 23.48763084 |
|  | 0.601970101 | 0.398070971 | Q7M6W1;A3QM89;Q8K0T0 | Reticulon;Reticulon-1 | Rtn1 | 25.12888336 | 25.65766335 | 24.93350601 | NaN | 24.86660385 | 24.81728935 |
|  | 0.48173755 | 0.402656555 | H3BKN0;Q1HFZ0 | tRNA (cytosine(34)-C(5))-methyltransferase | Nsun2 | NaN | 22.28045273 | 22.2696228 | NaN | 22.18764305 | 21.55711937 |
|  | 1.041913128 | 0.40693601 | P62897 | Cytochrome c, somatic | Cycs | 24.09273148 | 24.05862427 | 23.93238068 | 23.5685997 | 23.9501133 | 23.34421539 |
|  | 0.827792135 | 0.411452293 | Q9CXE7;E9PXY3 | Transmembrane emp24 domain-containing protein 5 | Tmed5 | NaN | 23.07626724 | 23.33128738 | NaN | 22.66628838 | 22.91836166 |
|  | 0.76522134 | 0.412220637 | Q8BFZ3 | Beta-actin-like protein 2 | Actbl2 | 30.0351181 | 29.72997475 | 29.4667263 | 29.51872635 | 29.51615524 | 28.96027565 |
|  | 0.807758116 | 0.4469649 | Q9D7S9 | Charged multivesicular body protein 5 | Chmp5 | 24.27646828 | 24.20672417 | 24.96247864 | 24.13273239 | 24.10813141 | 23.86391258 |
|  | 0.574715352 | 0.656689962 | Q3UV17 | Keratin, type II cytoskeletal 2 oral | Krt76 | 26.92592239 | 26.86431503 | 26.95681953 | 26.74611855 | 25.24343491 | 26.78743362 |
|  | 0.448722197 | 0.883074443 | Q9D125 | 28S ribosomal protein S25, mitochondrial | Mrps25 | 31.34101295 | 32.09106064 | 31.47058105 | 29.51084709 | 32.28578949 | 30.45679474 |
