# Supplementary material for: Intracellular iron accumulation facilitates mycobacterial infection in old mouse macrophages
Source: GeroScience. 2023 Dec 30;46(2):2739–54. doi: 10.1007/s11357-023-01048-1 (PMC10828278; doi:10.1007/s11357-023-01048-1)
Supplement: Supplementary file 5 — Supplementary file5 (DOCX 75 KB) [file 11357_2023_1048_MOESM5_ESM.docx]

**Supplementary Table 2A: Proteins Unique in *M.avium*-infected Old BMMs *vs* *M.avium*-infected Young BMMs**

| **Protein IDs** | **Protein names** | **Gene names** | ***M.avium*-infected Old BMMs** | | | ***M.avium*-infected Young BMMs** | | |
| --- | --- | --- | --- | --- | --- | --- | --- | --- |
|  |  |  | **LFQ intensity 1967_br2_tr1** | **LFQ intensity 1967_br2_tr2** | **LFQ intensity 1967_br2_tr3** | **LFQ intensity 1967_br4_tr1** | **LFQ intensity 1967_br4_tr2** | **LFQ intensity 1967_br4_tr3** |
| F8VQ05;A0A0J9YUH4;A0A0J9YUJ6 | FRY like transcription coactivator | Fryl | 180320000 | 189320000 | 0 | 140080000 | 0 | 0 |
| E9PVZ8;E9QAH1;Q9CTU9 | Golgi autoantigen, golgin subfamily b, macrogolgin 1 | Golgb1 | 157030000 | 207090000 | 176710000 | 0 | 0 | 0 |
| A0A1W2P8C6;P41731 | CD63 antigen | Cd63 | 68790000 | 69058000 | 65660000 | 0 | 0 | 0 |
| Q6PE70;G5E8F8;O70309;A0A338P795 | Integrin beta;Integrin beta-5 | Itgb5 | 47304000 | 46916000 | 51279000 | 0 | 0 | 0 |
| A0A286YCX6;E9PY51;A0A286YCE0 | Trafficking protein particle complex 8 | Trappc8 | 45352000 | 42186000 | 40241000 | 0 | 0 | 0 |
| A0A1D5RL86;P61514 | 60S ribosomal protein L37a | Rpl37a | 39815000 | 36496000 | 43011000 | 0 | 5099600 | 0 |
| Q78XF5 | Oligosaccharyltransferase complex subunit OSTC | Ostc | 36952000 | 45967000 | 0 | 0 | 0 | 0 |
| A0A140LIU9;P50637 | Translocator protein | Tspo | 30473000 | 20835000 | 0 | 0 | 0 | 18942000 |
| Q9R0T7;Q9QUK9 | Trypsin-4; Trypsin-5 | Try4;Try5 | 30219000 | 25488000 | 24496000 | 41651000 | 0 | 0 |
| P53996;A0A0N4SVS6 | Cellular nucleic acid-binding protein | Cnbp | 27943000 | 24852000 | 28454000 | 0 | 0 | 0 |
| Q99LM2;A2A6D9;B0QZI8;A2A6E1 | CDK5 regulatory subunit-associated protein 3 | Cdk5rap3 | 24354000 | 19537000 | 23044000 | 0 | 0 | 0 |
| Q923L7 | Ear6 protein | Ear6 | 22870000 | 15643000 | 18009000 | 0 | 0 | 0 |
| Q91WK5 | Glycine cleavage system H protein, mitochondrial | Gcsh | 21247000 | 21288000 | 23075000 | 0 | 0 | 0 |
| P62878;A0A2R8W6R3;A0A2R8W6G1 | E3 ubiquitin-protein ligase RBX1;E3 ubiquitin-protein ligase RBX1, N-terminally processed | Rbx1 | 20620000 | 22062000 | 22716000 | 0 | 0 | 0 |
| Q810Q5;A2AK37 | Normal mucosa of esophagus-specific gene 1 protein | Nmes1;AA467197 | 19123000 | 23436000 | 13535000 | 0 | 0 | 0 |
| Q6PGC1;Q8BN72 | ATP-dependent RNA helicase Dhx29 | Dhx29 | 18077000 | 0 | 18711000 | 0 | 0 | 0 |
| Q9DCG9;Q8VCR4 | Multifunctional methyltransferase subunit TRM112-like protein | Trmt112 | 17981000 | 15548000 | 14649000 | 0 | 18737000 | 0 |
| Q9DCF9 | Translocon-associated protein subunit gamma | Ssr3 | 17248000 | 19080000 | 0 | 19716000 | 0 | 0 |
| M0QWS4;Q9CR09 | Ubiquitin-fold modifier-conjugating enzyme 1 | Ufc1 | 15858000 | 17863000 | 16840000 | 0 | 0 | 0 |
| P17665 | Cytochrome c oxidase subunit 7C, mitochondrial | Cox7c | 15767000 | 15556000 | 15655000 | 0 | 0 | 0 |
| Q8BNI4;J3QMQ7;J3QMF4;Q3U957 | Derlin-2 | Derl2 | 14690000 | 0 | 13777000 | 0 | 0 | 15159000 |
| Q9D771 | Transmembrane protein 206 | Tmem206 | 13936000 | 11188000 | 10620000 | 0 | 0 | 12170000 |
| Q8K273 | Membrane magnesium transporter 1 | Mmgt1 | 13638000 | 0 | 14580000 | 0 | 0 | 0 |
| Q9D8L5 | Coiled-coil domain-containing protein 91 | Ccdc91 | 13626000 | 17206000 | 0 | 0 | 0 | 0 |
| E0CY91;E0CYW1;E0CY38;A0A1Y7VKT9;E0CZB3;Q9EPV8 | Ubiquitin-like protein 5 | Ubl5 | 13093000 | 15348000 | 11889000 | 0 | 13298000 | 0 |
| A0A286YCJ4;A0A3B2WC92;A0A286YE25;Q9QYY8;A0A3B2WBA7 | Spastin | Spast | 12975000 | 10079000 | 0 | 0 | 0 | 0 |
| O70378;M0QWP2;M0QWY0;M0QWS0;M0QWI7;M0QWE2;M0QWC9 | ER membrane protein complex subunit 8 | Emc8 | 12669000 | 9540300 | 7908000 | 0 | 0 | 7576000 |
| O09047 | C3a anaphylatoxin chemotactic receptor | C3ar1 | 12613000 | 11727000 | 12344000 | 0 | 0 | 0 |
| P35991;A2BDW0 | Tyrosine-protein kinase BTK | Btk | 12588000 | 11212000 | 11157000 | 12592000 | 0 | 0 |
| Q8VE99 | Coiled-coil domain-containing protein 115 | Ccdc115 | 12381000 | 10563000 | 0 | 13484000 | 0 | 0 |
| Q9WV03 | Protein FAM50A | Fam50a | 12250000 | 0 | 12911000 | 0 | 0 | 0 |
| A2AW05;Q08943 | FACT complex subunit SSRP1 | Ssrp1 | 12009000 | 0 | 0 | 0 | 7852000 | 0 |
| E9PUQ9;F7AC58;E2JF22 | Piezo-type mechanosensitive ion channel component;Piezo-type mechanosensitive ion channel component 1 | Piezo1 | 11919000 | 0 | 9834300 | 14974000 | 0 | 0 |
| A0A087WRC6;A0A087WR57;Q91XC8 | Death-associated protein 1 | Dap | 11787000 | 10798000 | 9102200 | 6800300 | 0 | 0 |
| D3Z3A0;Q9DCL8 | Protein phosphatase inhibitor 2 | Ppp1r2 | 11381000 | 0 | 12936000 | 0 | 13869000 | 0 |
| F8WHU8;Q9D8T7 | SRA stem-loop-interacting RNA-binding protein, mitochondrial | Slirp | 11088000 | 0 | 9983400 | 10915000 | 0 | 0 |
| G5E895;D3Z494;S4R2G9 | Aldo-keto reductase family 1, member B10 (aldose reductase) | Akr1b10 | 10464000 | 9490800 | 10938000 | 0 | 0 | 0 |
| P18052;Q91V35;A0A2R8VHH6 | Receptor-type tyrosine-protein phosphatase alpha;Receptor-type tyrosine-protein phosphatase | Ptpra | 10012000 | 10173000 | 11612000 | 0 | 10383000 | 0 |
| Q3THE2;Q9CQ19;D3Z249 | Myosin regulatory light chain 12B | Myl12b | 9956200 | 9325100 | 9690800 | 0 | 0 | 0 |
| Q3UMB9 | WASH complex subunit 7 | Kiaa1033 | 9803600 | 10869000 | 10731000 | 0 | 0 | 0 |
| O89051;A0A2I3BQ30;A0A2I3BPW7 | Integral membrane protein 2B;BRI2, membrane form;BRI2 intracellular domain;BRI2C, soluble form;Bri23 peptide | Itm2b | 9625600 | 9277200 | 8337400 | 0 | 0 | 9424000 |
| Q921L6;Q60598 | Src substrate cortactin | Cttn | 9595200 | 11631000 | 15030000 | 0 | 0 | 0 |
| E9QKC6;Q3UHH4;Q9ESN6 | Tripartite motif-containing protein 2 | Trim2 | 9412100 | 9572800 | 9452000 | 0 | 10173000 | 0 |
| A2ADH1;Q9CQY5 | Magnesium transporter protein 1 | Magt1 | 9310800 | 11326000 | 0 | 0 | 0 | 0 |
| D3YZ09;Q9CY66 | H/ACA ribonucleoprotein complex subunit 1 | Gar1 | 9060700 | 6007400 | 8298800 | 0 | 5826400 | 0 |
| O54950;A0A2R8VHU2;D3YUS1;Q8BIQ9;Q91WG5 | 5-AMP-activated protein kinase subunit gamma-1 | Prkag1 | 9045900 | 10144000 | 8644000 | 11686000 | 0 | 0 |
| Q9CRY7 | Glycerophosphodiester phosphodiesterase domain-containing protein 1 | Gdpd1 | 8792200 | 8920000 | 9845900 | 0 | 0 | 0 |
| Q921F4;V9GXB6 | Heterogeneous nuclear ribonucleoprotein L-like | Hnrnpll | 8720500 | 8878200 | 10394000 | 0 | 7750000 | 0 |
| Q9EP89;A0A1L1SVF9 | Serine beta-lactamase-like protein LACTB, mitochondrial | Lactb | 8562800 | 8574600 | 9135900 | 0 | 0 | 9660900 |
| Q3UHD6;A0A0G2JF85;A0A0G2JG07;A0A0G2JGD5 | Sorting nexin-27 | Snx27 | 8451500 | 6484400 | 0 | 0 | 0 | 0 |
| Q9CQ48;E0CYQ2 | NudC domain-containing protein 2 | Nudcd2 | 8369500 | 7950000 | 7148700 | 0 | 0 | 0 |
| Q9CX30;D3YY42;A0A140LHN0;D3Z5F9 | Protein YIF1B | Yif1b | 8332700 | 8155800 | 7535600 | 7495500 | 0 | 0 |
| Q3UDE2;F2Z423 | Tubulin--tyrosine ligase-like protein 12 | Ttll12 | 8175400 | 6979800 | 5239400 | 0 | 0 | 0 |
| Q9JKB3 | Y-box-binding protein 3 | Ybx3 | 8100300 | 5531500 | 6056100 | 0 | 0 | 0 |
| G3X928;Q6NZC7 | SEC23-interacting protein | Sec23ip | 8052300 | 7642100 | 8440000 | 0 | 0 | 0 |
| A0A2R8W6X5;Q9R060 | Cytosolic Fe-S cluster assembly factor NUBP1 | Nubp1 | 8009500 | 7427000 | 0 | 0 | 0 | 8980800 |
| O55242 | Sigma non-opioid intracellular receptor 1 | Sigmar1 | 7953700 | 8685500 | 8468600 | 0 | 0 | 0 |
| A0A0R4J1R7;Q9CZL5 | Pterin-4-alpha-carbinolamine dehydratase 2 | Pcbd2 | 7838600 | 9668200 | 7527100 | 0 | 0 | 0 |
| Q9R190 | Metastasis-associated protein MTA2 | Mta2 | 7616700 | 0 | 8149200 | 0 | 0 | 0 |
| Q923D4 | Splicing factor 3B subunit 5 | Sf3b5 | 7556100 | 8194900 | 6924700 | 0 | 0 | 0 |
| Q8R1G6;A0A087WPL1;F7C957;E9Q996 | PDZ and LIM domain protein 2 | Pdlim2 | 7553700 | 8821800 | 9587100 | 0 | 0 | 0 |
| A0A0R4J0P1;Q9D7B6;D3YTT4;D6RDD5 | Isobutyryl-CoA dehydrogenase, mitochondrial | Acad8 | 7354700 | 9581900 | 6896300 | 0 | 0 | 0 |
| Q9R0M6;A2AFP4;A2AFP5 | Ras-related protein Rab-9A | Rab9a | 7317400 | 7125900 | 7110100 | 0 | 0 | 0 |
| Q8C6U2;Q3UU83;A0A1Y7VLY9 | PQ-loop repeat-containing protein 3 | Pqlc3 | 7266300 | 9536700 | 0 | 0 | 0 | 0 |
| Q99LE1 | RILP-like protein 2 | Rilpl2 | 7223900 | 6609300 | 6037100 | 0 | 6547500 | 0 |
| Q60996;A0A1Y7VIR0;Q91V89;Q7TNL5 | Serine/threonine-protein phosphatase 2A 56 kDa regulatory subunit gamma isoform | Ppp2r5c;Ppp2r5d | 7215200 | 7055500 | 8130600 | 0 | 0 | 0 |
| O35459;F7B227 | Delta(3,5)-Delta(2,4)-dienoyl-CoA isomerase, mitochondrial | Ech1 | 7136000 | 7013500 | 6556500 | 0 | 0 | 6361400 |
| A0A087WQT6;O89110 | Caspase-8;Caspase-8 subunit p18;Caspase-8 subunit p10 | Casp8 | 7132100 | 8077000 | 0 | 0 | 0 | 0 |
| Q9CPP6 | NADH dehydrogenase [ubiquinone] 1 alpha subcomplex subunit 5 | Ndufa5 | 7127100 | 7234600 | 7118100 | 0 | 0 | 5721900 |
| Q91YJ2 | Sorting nexin-4 | Snx4 | 7101600 | 6969300 | 5895300 | 0 | 0 | 0 |
| E9Q6W2;Q9D2R6 | Cytochrome c oxidase assembly factor 3 homolog, mitochondrial | Coa3 | 7070600 | 10072000 | 6811400 | 0 | 0 | 0 |
| Q922Q1;E0CZH6 | Mitochondrial amidoxime reducing component 2 | Mtarc2 | 7065600 | 6263800 | 6258300 | 4798800 | 0 | 0 |
| Q8K469;P11928;Q05BJ7;Q8VI95;Q924S2 | 2-5-oligoadenylate synthase 1A | Oas1g;Oas1a | 6977600 | 6998400 | 7527700 | 0 | 6703800 | 0 |
| P59325;A0A1Y7VNP4;A0A1Y7VJG2;Q8BVV6;A0A1Y7VLQ0;A0A1Y7VLL6;A0A1Y7VLK2 | Eukaryotic translation initiation factor 5 | Eif5 | 6937700 | 7416300 | 7299400 | 0 | 0 | 0 |
| Q80VD1;A0A3Q4EBV4;Q3TJZ6 | Protein FAM98B | Fam98b | 6931200 | 0 | 6318200 | 0 | 0 | 0 |
| Q3UPF5;D3Z5I1;G3X9X5 | Zinc finger CCCH-type antiviral protein 1 | Zc3hav1 | 6915300 | 6996600 | 6957400 | 0 | 0 | 0 |
| D3Z3Y4;Q921W2;P70318;A0A0U1RPD3;A0A0N4SVR8;A0A0U1RPE1;A0A0N4SUX1;D6RGU1;A0A0U1RPB0;D3YY02;G5E8L2;Q80ZW7;D3Z4H6;P52912 | Nucleolysin TIAR | Tial1 | 6864200 | 7637800 | 6912800 | 0 | 5416700 | 0 |
| A2AFJ1;Q60973;A2AFI9;F6ZLC6;F6U539 | Histone-binding protein RBBP7 | Rbbp7 | 6750400 | 7793700 | 9357700 | 7125400 | 0 | 0 |
| Q8K021;Q3TSA8;D3YTP4 | Secretory carrier-associated membrane protein 1 | Scamp1 | 6746700 | 7932400 | 10383000 | 9302100 | 0 | 0 |
| Q9D6Y7;A0A1B0GT40 | Mitochondrial peptide methionine sulfoxide reductase | Msra | 6729700 | 0 | 6778000 | 0 | 0 | 9342100 |
| A0A087WNV1;A0A087WSR7;A0A087WR52;Q8K2K6;A0A087WRL1 | Arf-GAP domain and FG repeat-containing protein 1 | Agfg1 | 6698000 | 0 | 6158000 | 0 | 4933700 | 0 |
| G3X9I1;G5E911;Q91VU0;E9Q6F0 | Protein FAM3C | Fam3c | 6680200 | 7000600 | 6564800 | 0 | 0 | 0 |
| Q8K3J1;A0A494BAW8 | NADH dehydrogenase [ubiquinone] iron-sulfur protein 8, mitochondrial | Ndufs8 | 6624800 | 5296500 | 7572600 | 0 | 0 | 0 |
| A2AUE1;G5E8T0;P60904 | DnaJ homolog subfamily C member 5 | Dnajc5 | 6585500 | 0 | 4795900 | 0 | 0 | 0 |
| P30993 | C5a anaphylatoxin chemotactic receptor 1 | C5ar1 | 6570500 | 6122900 | 6883100 | 6082900 | 0 | 0 |
| Q9CQV4 | Protein FAM134C | Fam134c | 6570400 | 0 | 6399300 | 7143000 | 0 | 0 |
| Q4PJX1;A0A0A0MQB0 | Protein odr-4 homolog | Odr4;BC003331 | 6528800 | 7364300 | 6427200 | 0 | 0 | 5800800 |
| A0A1Y7VN19;Q9CXR1 | Dehydrogenase/reductase SDR family member 7 | Dhrs7 | 6408000 | 6082800 | 0 | 6369400 | 0 | 0 |
| J3QJX3;Q9Z2G6 | Protein sel-1 homolog 1 | Sel1l | 6383100 | 6075400 | 0 | 0 | 0 | 0 |
| Z4YKC4;A0A0R4J112;A2AMI2;A0A0N4SVL0;Q80XI3;A2AMI7 | Eukaryotic translation initiation factor 4 gamma 3 | Eif4g3 | 6377300 | 6510800 | 5928500 | 0 | 0 | 0 |
| D3Z191;Q80X71;D3Z6E0;D3Z0M2 | Transmembrane protein 106B | Tmem106b | 6375200 | 5935400 | 6077800 | 0 | 5527600 | 0 |
| Q9ESW4 | Acylglycerol kinase, mitochondrial | Agk | 6322100 | 5949000 | 5625700 | 0 | 0 | 0 |
| Q60855;F7D1J2 | Receptor-interacting serine/threonine-protein kinase 1 | Ripk1 | 6304600 | 6017100 | 7005000 | 0 | 5888300 | 0 |
| A0A2R8VKS6;Q9D1C8 | Vacuolar protein sorting-associated protein 28 homolog | Vps28 | 6261500 | 5922600 | 6308400 | 0 | 0 | 0 |
| Q7TMF3;A0A0R4J275;F6RBR6 | NADH dehydrogenase [ubiquinone] 1 alpha subcomplex subunit 12 | Ndufa12 | 6260500 | 7863400 | 5098400 | 0 | 3081900 | 0 |
| E9QKA4;A2A8V8;A2A8V9;E9PUK6;Q52KI8;F6T4M4;A2A983;F6UK16 | Serine/arginine repetitive matrix protein 1 | Srrm1 | 6258800 | 8482000 | 0 | 6364200 | 4930000 | 0 |
| Q9JJV2;D3YWS3 | Profilin-2;Profilin | Pfn2 | 6222400 | 6262400 | 0 | 0 | 0 | 7434200 |
| P0DOV2;P0DOV1;Q8CGE8 | Interferon-activable protein 204 | Ifi204 | 6212400 | 7353300 | 0 | 0 | 0 | 0 |
| P97808;F6TWM7;F8WJA1;E0CXN1;A0A0J9YUX0;E0CYX7;E0CXM5 | FXYD domain-containing ion transport regulator 5 | Fxyd5 | 6153800 | 4111900 | 0 | 0 | 0 | 0 |
| Q8BH69 | Selenide, water dikinase 1 | Sephs1 | 6146000 | 5261600 | 6158000 | 0 | 5821700 | 0 |
| P00375 | Dihydrofolate reductase | Dhfr | 6122100 | 6477400 | 5414600 | 0 | 7683100 | 0 |
| Q9CXY6 | Interleukin enhancer-binding factor 2 | Ilf2 | 6111600 | 6732400 | 7603000 | 0 | 0 | 0 |
| Q8BHS6 | Armadillo repeat-containing X-linked protein 3 | Armcx3 | 6094800 | 8495300 | 7042200 | 0 | 6817600 | 0 |
| Q80TM9;F6ZL69;F6YR29 | Nischarin | Nisch | 6071800 | 0 | 5381800 | 0 | 0 | 0 |
| B1AU76;B1AU75;Q99MD9 | Nuclear autoantigenic sperm protein | Nasp | 6045800 | 10687000 | 4707300 | 0 | 0 | 0 |
| A0A338P6P6;D4AFX6;Q6ZQK5 | Arf-GAP with coiled-coil, ANK repeat and PH domain-containing protein 2 | Acap2 | 6003900 | 7067100 | 7250900 | 6922800 | 0 | 0 |
| Q8C522;A0A1L1SSA0 | Endonuclease domain-containing 1 protein | Endod1 | 6000600 | 6567700 | 5606600 | 7368200 | 0 | 0 |
| Q8BQ47 | Protein canopy homolog 4 | Cnpy4 | 5992700 | 0 | 5973300 | 4074600 | 0 | 0 |
| F7CTF8;E0CYI3;E0CYB9;Q6PCP5;F7B1A4;F6VAL0 | Mitochondrial fission factor | Mff | 5942000 | 5702100 | 0 | 0 | 6040000 | 0 |
| Q8R164 | Valacyclovir hydrolase | Bphl | 5884600 | 8022700 | 7739100 | 0 | 0 | 0 |
| A0A2I3BS49;Q9CXU4;Q9WTQ8 | Mitochondrial import inner membrane translocase subunit Tim23 | Timm23 | 5861700 | 0 | 5720000 | 0 | 0 | 0 |
| A0A1L1SVG6;Q8BY89 | Choline transporter-like protein 2 | Slc44a2 | 5859200 | 4707100 | 4896100 | 0 | 4975300 | 0 |
| F8WJG3;P62996 | Transformer-2 protein homolog beta | Tra2b | 5822400 | 5260500 | 6755800 | 0 | 0 | 0 |
| Q6PGH1 | Protein BUD31 homolog | Bud31 | 5805900 | 4091300 | 4821000 | 2359700 | 0 | 0 |
| F6SPQ1;O35448;E9PVM9;E9Q0T0;G5E8W7 | Lysosomal thioesterase PPT2 | Ppt2 | 5774500 | 4936600 | 5222500 | 0 | 0 | 0 |
| Q8BPX9 | Solute carrier family 15 member 3 | Slc15a3 | 5772900 | 5663000 | 8367600 | 0 | 0 | 0 |
| P48774;E9PVM7;E9PV63 | Glutathione S-transferase Mu 5 | Gstm5 | 5540500 | 5471200 | 5465900 | 0 | 0 | 0 |
| Q9ERL7;D3Z2F6 | Glia maturation factor gamma | Gmfg | 5475700 | 0 | 5724600 | 0 | 0 | 0 |
| Q9CYA0 | Cysteine-rich with EGF-like domain protein 2 | Creld2 | 5467300 | 0 | 6724900 | 0 | 0 | 0 |
| E0CZE0;Q8VBW6 | NEDD8-activating enzyme E1 regulatory subunit | Nae1 | 5456000 | 6716500 | 5420100 | 0 | 0 | 0 |
| A0A1L1SVC7;Q9CQ02;A0A1L1SRX4;A0A1L1SRM6;A0A1L1ST08;A0A1L1SRJ9 | COMM domain-containing protein 4 | Commd4 | 5454400 | 6902900 | 5706700 | 0 | 0 | 0 |
| P54818;A0A1Y7VM63 | Galactocerebrosidase | Galc | 5451500 | 7811400 | 9222500 | 0 | 0 | 0 |
| F7B209;Q80UP5;Q6PD24 | Ankyrin repeat domain-containing protein 13A | Ankrd13a | 5388700 | 3626100 | 0 | 0 | 0 | 0 |
| Q9D404 | 3-oxoacyl-[acyl-carrier-protein] synthase, mitochondrial | Oxsm | 5340400 | 6297100 | 0 | 0 | 0 | 0 |
| P47877;D3YU40 | Insulin-like growth factor-binding protein 2 | Igfbp2 | 5339300 | 0 | 4965700 | 0 | 0 | 0 |
| Z4YN00;Q9QZN4;A2A7G9;A2A7H1;A2A7H3 | F-box only protein 6 | Fbxo6 | 5332100 | 5110800 | 4925000 | 0 | 0 | 5185900 |
| Q6A0A2;A0A087WPU9 | La-related protein 4B | Larp4b | 5303200 | 5064100 | 5363400 | 0 | 0 | 0 |
| Q9CXF4 | TBC1 domain family member 15 | Tbc1d15 | 5279000 | 4836300 | 5587700 | 0 | 0 | 0 |
| Q9ERS2 | NADH dehydrogenase [ubiquinone] 1 alpha subcomplex subunit 13 | Ndufa13 | 5238000 | 6549300 | 4808400 | 0 | 0 | 0 |
| Q99KN2;G3UYN9;G3UYR5 | Probable cytosolic iron-sulfur protein assembly protein CIAO1 | Ciao1 | 5230900 | 5159800 | 0 | 0 | 0 | 0 |
| Q3ULG5;P97311 | DNA helicase;DNA replication licensing factor MCM6 | Mcm6 | 5210800 | 0 | 0 | 0 | 0 | 0 |
| Q80VA0 | N-acetylgalactosaminyltransferase 7 | Galnt7 | 5205200 | 6239000 | 5952800 | 0 | 7305800 | 0 |
| Q8CGY8 | UDP-N-acetylglucosamine--peptide N-acetylglucosaminyltransferase 110 kDa subunit | Ogt | 5199700 | 0 | 5115900 | 0 | 5132200 | 0 |
| Q7TT50;A0A1Y7VLI0 | Serine/threonine-protein kinase MRCK beta | Cdc42bpb | 5179200 | 6311200 | 7424800 | 6797400 | 0 | 0 |
| Q8BVL3;D3Z3J1;H3BKG9;D3Z7S9 | Sorting nexin-17 | Snx17 | 5138800 | 5098700 | 0 | 0 | 0 | 0 |
| A0A494BA44;Q04207;Q4U113;A0A494B901 | Transcription factor p65 | Rela | 5106300 | 7075200 | 6184100 | 0 | 0 | 0 |
| Q91ZN5;A0A286YE16;Q9D1L5 | Adenosine 3-phospho 5-phosphosulfate transporter 1 | Slc35b2 | 5104400 | 5164100 | 4946700 | 0 | 0 | 0 |
| Q8BR63 | Protein FAM177A1 | Fam177a1 | 5100300 | 4946100 | 5707000 | 0 | 0 | 0 |
| A0A3Q4EGT0;A0A3Q4EII3;A0A3Q4EC50;Q8JZY2;A0A3Q4EHW7;Q3UTM6 | COMM domain-containing protein 10 | Commd10 | 5027500 | 4547100 | 4903500 | 0 | 0 | 0 |
| Q8BP92;D6RHL9 | Reticulocalbin-2 | Rcn2 | 4957900 | 5678100 | 7112200 | 0 | 0 | 7590900 |
| P32233 | Developmentally-regulated GTP-binding protein 1 | Drg1 | 4950000 | 5588000 | 0 | 0 | 0 | 0 |
| Q91WK1 | SPRY domain-containing protein 4 | Spryd4 | 4904600 | 4113500 | 4899300 | 0 | 0 | 0 |
| Q9Z1K5;H7BWY7;H9KV21 | E3 ubiquitin-protein ligase ARIH1 | Arih1 | 4883600 | 4860100 | 5431300 | 0 | 0 | 0 |
| A0A087WNZ7;G5E870;A0A087WP92;A0A087WRV6;A0A0B4J1N9;A0A087WS65;Q3TP48;A0A087WSG4;A0A087WQ02 | E3 ubiquitin-protein ligase TRIP12 | Trip12 | 4883000 | 3900600 | 0 | 0 | 4677400 | 0 |
| Q8CHW4;A0A338P6D9;F6T9C3 | Translation initiation factor eIF-2B subunit epsilon | Eif2b5 | 4882300 | 4053700 | 3909100 | 0 | 0 | 0 |
| A0A0R4J007;P70261 | Paladin | Pald1 | 4822500 | 5027500 | 0 | 0 | 0 | 0 |
| Q9DAR7;Q3TBW9;D6RFQ0 | m7GpppX diphosphatase | Dcps | 4812200 | 4533000 | 5864500 | 0 | 5825700 | 0 |
| Q9QXW9 | Large neutral amino acids transporter small subunit 2 | Slc7a8 | 4744800 | 5498500 | 4785300 | 0 | 0 | 0 |
| Q8BJ48;D3Z6C9 | N-acetylglucosamine-1-phosphodiester alpha-N-acetylglucosaminidase | Nagpa | 4742300 | 6807000 | 0 | 0 | 0 | 0 |
| P0C0A3;B1AZ42;B1AZ41 | Charged multivesicular body protein 6 | Chmp6 | 4722700 | 4342500 | 0 | 0 | 0 | 0 |
| D3Z6I4;Q921W4;D3YU21;D3YZD6;A0A338P7D6;A0A338P6Y1;A0A338P785;F7BGV1 | Quinone oxidoreductase-like protein 1 | Cryzl1 | 4653600 | 5414000 | 4783900 | 0 | 0 | 0 |
| Q922V4;D3Z4V1;F8WI31 | Pleiotropic regulator 1 | Plrg1 | 4621500 | 4487400 | 0 | 0 | 0 | 0 |
| A0A0U1RQ06;P62743 | AP-2 complex subunit sigma | Ap2s1 | 4608900 | 3887800 | 4349700 | 3859500 | 0 | 0 |
| Q80VP1;D3Z4V3;D3Z550;Q91W69 | Epsin-1 | Epn1 | 4581700 | 4911300 | 0 | 0 | 0 | 6501200 |
| A0A1Y7VKP8;A0A1Y7VM38;P52503 | NADH dehydrogenase [ubiquinone] iron-sulfur protein 6, mitochondrial | Ndufs6 | 4571400 | 4936700 | 0 | 0 | 0 | 0 |
| Q9JLQ0;A0A3B2W812 | CD2-associated protein | Cd2ap | 4469400 | 0 | 6334900 | 0 | 0 | 0 |
| Q9CR60 | Vesicle transport protein GOT1B | Golt1b | 4460600 | 4000700 | 9320100 | 0 | 0 | 0 |
| D3Z079;Q8K400;D3Z2Q2;F6WXQ4 | Syntaxin-binding protein 5 | Stxbp5 | 4460000 | 3097500 | 4022400 | 0 | 0 | 4668200 |
| P70295;Q3U3K9;A0A0N4SVA6 | Ancient ubiquitous protein 1 | Aup1 | 4451200 | 4194700 | 4491900 | 4557600 | 0 | 0 |
| Q80XR8;J3QK07;D3Z1I2;E9PVA6;Q9JLQ2;F6U8T2;F7BIK4;F6SLJ2;F6WV69;D3Z409 | ARF GTPase-activating protein GIT2 | Git2 | 4430000 | 5358300 | 6945900 | 0 | 0 | 0 |
| A0A0R4J0Q5;P21619 | Lamin-B2 | Lmnb2 | 4422500 | 4628100 | 3957100 | 0 | 4415500 | 0 |
| Q3U0I9;Q8CFD4;A0A0G2JEQ9;A0A0G2JDT3;A0A0G2JDJ6 | Sorting nexin-8 | Snx8 | 4354900 | 0 | 4693000 | 0 | 0 | 0 |
| A0A087WRH2;D3Z3C1;Q9WUD8;J3QPY3 | Fas apoptotic inhibitory molecule 1 | Faim;Gm6432 | 4354400 | 6083200 | 5060000 | 4104400 | 0 | 0 |
| Q8BP48;A0A0G2JF71;A0A0G2JFL1 | Methionine aminopeptidase 1 | Metap1 | 4326500 | 5163400 | 6244100 | 0 | 0 | 6229000 |
| Q9D1K7;F8WIU1;H7BXB9 | UPF0687 protein C20orf27 homolog | 1700037H04Rik | 4294900 | 3693300 | 0 | 0 | 0 | 0 |
| Q9DBD5;Q9D4T3 | Proline-, glutamic acid- and leucine-rich protein 1 | Pelp1 | 4285500 | 4076800 | 0 | 0 | 0 | 0 |
| Q80SW1 | Putative adenosylhomocysteinase 2 | Ahcyl1 | 4276600 | 7402500 | 9275900 | 0 | 0 | 0 |
| Q5SUF2 | Luc7-like protein 3 | Luc7l3 | 4269500 | 4094000 | 3980800 | 0 | 0 | 0 |
| Q3U2C5 | E3 ubiquitin-protein ligase RNF149 | Rnf149 | 4255300 | 5482700 | 6135800 | 0 | 0 | 0 |
| A2AWA9;A2AWB0;A0A3Q4L343;A0A0A6YWT4;A0A0A6YY35;A2AWA7 | Rab GTPase-activating protein 1 | Rabgap1 | 4139700 | 4524100 | 0 | 0 | 0 | 0 |
| A0A494B9R1;Q62084;A0A494B933;A0A494BB30 | Protein phosphatase 1 regulatory subunit 14B | Ppp1r14b | 4065300 | 5363800 | 4584400 | 2999400 | 0 | 0 |
| Q8CH18;A0A1W2P7Q7;A0A1W2P765 | Cell division cycle and apoptosis regulator protein 1 | Ccar1 | 4053800 | 3977800 | 4730800 | 0 | 3933700 | 0 |
| Q3USX5;Q8R4K2 | Interleukin-1 receptor-associated kinase 4 | Irak4 | 4030100 | 4213000 | 4066000 | 0 | 0 | 0 |
| E0CXA3;E0CX79;E0CXR3;E0CZD9;E0CXM9;A0A494B9Y9;Q9DB43 | Zinc finger protein-like 1 | Zfpl1 | 4016800 | 4647200 | 4182100 | 0 | 0 | 0 |
| A2A7A7;Q8CFX1 | GDH/6PGL endoplasmic bifunctional protein;Glucose 1-dehydrogenase;6-phosphogluconolactonase | H6pd | 3975300 | 3894400 | 4000800 | 0 | 0 | 0 |
| Q3TH73 | Protein tweety homolog 2 | Ttyh2 | 3967900 | 3973700 | 4598300 | 0 | 0 | 0 |
| P28658 | Ataxin-10 | Atxn10 | 3946900 | 4948100 | 3877800 | 0 | 0 | 0 |
| Q9QYE6;A0A1Y7VMD3 | Golgin subfamily A member 5 | Golga5 | 3915500 | 0 | 4071000 | 0 | 0 | 0 |
| Q91X51 | Golgi reassembly-stacking protein 1 | Gorasp1 | 3905200 | 3065600 | 0 | 0 | 0 | 0 |
| P11152 | Lipoprotein lipase | Lpl | 3885300 | 3824100 | 4210800 | 3640800 | 0 | 0 |
| Q9D6U8 | Protein FAM162A | Fam162a | 3869000 | 5301600 | 4923200 | 0 | 0 | 4088700 |
| Q9D4H1 | Exocyst complex component 2 | Exoc2 | 3859200 | 0 | 3566000 | 0 | 0 | 0 |
| A0A0U1RQ61;Q14C51 | Pentatricopeptide repeat domain-containing protein 3, mitochondrial | Ptcd3 | 3831400 | 3242300 | 0 | 0 | 0 | 0 |
| D3YYK8;E9Q6X0;Q8R001;Q3TG90 | Microtubule-associated protein RP/EB family member 2 | Mapre2 | 3815000 | 3512400 | 4034300 | 5136500 | 0 | 0 |
| O08759;A0A0J9YUK0;A0A0J9YVG1;A0A0J9YUY4 | Ubiquitin-protein ligase E3A | Ube3a | 3807200 | 2486500 | 2978600 | 0 | 0 | 0 |
| Q9QYF9;Q8VCV2;Q8CBD0 | Protein NDRG3 | Ndrg3 | 3788300 | 3389900 | 3331400 | 0 | 0 | 3065000 |
| H3BIX0;H3BL37;O08784;F6R3V4;H3BK88 | Treacle protein | Tcof1 | 3701700 | 3952800 | 3338500 | 0 | 0 | 0 |
| A2AAN0;O35250 | Exocyst complex component 7 | Exoc7 | 3690900 | 3338600 | 0 | 0 | 0 | 0 |
| H3BLL3;Q921G6;H3BIX9;H3BLB4;H3BJU9 | Leucine-rich repeat and calponin homology domain-containing protein 4 | Lrch4 | 3680200 | 3458300 | 3322300 | 0 | 0 | 4027700 |
| Q569Z5;F8WHR6;A0A2R8VHK2 | Probable ATP-dependent RNA helicase DDX46 | Ddx46 | 3675000 | 3491900 | 4297700 | 0 | 0 | 0 |
| A0A286YDA2;A0A286YDT3;E9Q5C9;A0A286YDV7 | | Nolc1 | 3669800 | 3952000 | 4537800 | 0 | 2379500 | 0 |
| Q9QZB7;A0A1Y7VL71 | Actin-related protein 10 | Actr10 | 3609300 | 3948700 | 3584500 | 0 | 4419100 | 0 |
| Q9JIH2 | Nuclear pore complex protein Nup50 | Nup50 | 3566100 | 3576600 | 3586200 | 0 | 2944100 | 0 |
| Q64261;A0A0G2JGH2;A0A0G2JGA8 | Cyclin-dependent kinase 6 | Cdk6 | 3533900 | 3101600 | 3277600 | 3886000 | 0 | 0 |
| D3YUB9;G3UY42;D3Z5F7;Q8CCS6;D3Z055;G3UWS5 | Polyadenylate-binding protein 2 | Pabpn1;Gm20521 | 3516200 | 3057100 | 3758900 | 0 | 0 | 0 |
| Q3U6F1;E9Q7W0;A0A0J9YTV5;P31266 | Recombining binding protein suppressor of hairless | Rbpj | 3500900 | 2259700 | 2597900 | 0 | 0 | 0 |
| A2ATP5;A2ATP6;Q8C854;F6XJA1;G8JL68 | Myelin expression factor 2 | Myef2 | 3391700 | 3420200 | 2694900 | 0 | 0 | 0 |
| A0A0J9YUD5;B9EJ54;E9Q880 | Nucleoporin 205 | Nup205 | 3351800 | 3027100 | 3200000 | 0 | 0 | 0 |
| Q9D1H8 | 39S ribosomal protein L53, mitochondrial | Mrpl53 | 3350700 | 0 | 3414900 | 0 | 0 | 0 |
| F8VPX1;Q6A4J8;E9PXY8;G3UWR8;E0CY04 | Ubiquitin carboxyl-terminal hydrolase;Ubiquitin carboxyl-terminal hydrolase 7 | Usp7 | 3343300 | 0 | 2687700 | 0 | 0 | 3069600 |
| Q9CPN8;A6X8Z3;Q5SF07 | Insulin-like growth factor 2 mRNA-binding protein 3 | Igf2bp3 | 3335000 | 0 | 4124900 | 0 | 0 | 0 |
| P52633;D3YU28 | Signal transducer and transcription activator 6 | Stat6 | 3304300 | 3822300 | 4227200 | 0 | 0 | 0 |
| Q8VD62 | UPF0696 protein C11orf68 homolog | Bles03 | 3300700 | 2977100 | 2786500 | 0 | 0 | 0 |
| Q6P6P5;Q3V0N8;Q924N4;A2AGJ9;F8WIJ0;Q9JIS8;A0A076FR46;Q91V14 | Solute carrier family 12 member 6;Solute carrier family 12 member 4 | Slc12a6;Slc12a4 | 3281000 | 3288300 | 3439400 | 2299300 | 0 | 0 |
| E9PX53;E9QPR5;Q8K2V1;E0CXK8;E0CYV2;F6TLH3;F6V294;E0CX37 | Serine/threonine-protein phosphatase 4 regulatory subunit 1 | Ppp4r1 | 3216800 | 2543400 | 0 | 0 | 0 | 0 |
| Q8C0L6;A0A1B0GRJ2;Q3TXR6 | Peroxisomal N(1)-acetyl-spermine/spermidine oxidase | Paox | 3204200 | 0 | 2760100 | 0 | 0 | 3058700 |
| Q9DBR1 | 5-3 exoribonuclease 2 | Xrn2 | 3197900 | 3178600 | 4088100 | 0 | 0 | 0 |
| Q80U93;A0A0A6YW83 | Nuclear pore complex protein Nup214 | Nup214 | 3143500 | 2799200 | 3516900 | 2798200 | 0 | 0 |
| F8VQE9;Q8VHH5;A0A0G2JER6;A0A1D5RMG4;A0A0G2JDW1;A0A087WRF2;Q8BXK8 | Arf-GAP with GTPase, ANK repeat and PH domain-containing protein 3 | Agap3 | 3131300 | 3226900 | 3520400 | 0 | 0 | 3920600 |
| H3BJ71;H3BKL6;H3BL19;H3BJI6;H3BK44;E9PY90;H3BK48;E9Q3S2;H3BKH2;H3BJS0;A0A1W2P711;Q91ZV0;H3BJ35 | Melanoma inhibitory activity protein 2 | Ctage5;Mia2 | 3106800 | 3366500 | 3295200 | 0 | 0 | 0 |
| P18155 | Bifunctional methylenetetrahydrofolate dehydrogenase/cyclohydrolase, mitochondrial;NAD-dependent methylenetetrahydrofolate dehydrogenase;Methenyltetrahydrofolate cyclohydrolase | Mthfd2 | 3100200 | 3506700 | 4550900 | 0 | 0 | 0 |
| Q3TFQ1;Q3UBW1 | SPRY domain-containing protein 7 | Spryd7 | 3081700 | 3232800 | 3514300 | 3598800 | 0 | 3663600 |
| A0A494BAB5;Q9D5T0;A0A494B9L7;A0A494B9Y0 | ATPase family AAA domain-containing protein 1 | Atad1 | 3078800 | 3334100 | 3468500 | 0 | 0 | 2099400 |
| Q924L1 | LETM1 domain-containing protein 1 | Letmd1 | 3069500 | 3224800 | 2982100 | 2341300 | 0 | 0 |
| Q9QXD8 | LIM domain-containing protein 1 | Limd1 | 3051500 | 4101800 | 4086800 | 0 | 0 | 4403500 |
| O08917;G3UZZ5;G3UWW8;G3UYU4 | Flotillin-1 | Flot1 | 3010400 | 2874000 | 2922100 | 0 | 0 | 0 |
| D3Z061;Q8C7R4 | Ubiquitin-like modifier-activating enzyme 6 | Uba6 | 3004400 | 2873800 | 2812400 | 0 | 0 | 2664800 |
| Q99PG2 | Opioid growth factor receptor | Ogfr | 2983100 | 2092200 | 3411300 | 0 | 0 | 0 |
| Q5SU48;Q7TN98;Q5SU47;F6T0J8;D3Z1R6;D3Z5R9;A0A0R4J102;E9Q5X2;A0A0H2UH22;E9Q969;Q812E0;Q7TN99 | Cytoplasmic polyadenylation element-binding protein 4 | Cpeb4 | 2922300 | 2894100 | 0 | 0 | 0 | 0 |
| Q8BL80;D3Z1Y5;A0A2I3BRZ0 | Rho GTPase-activating protein 22 | Arhgap22 | 2922200 | 3127400 | 4187100 | 0 | 3898500 | 0 |
| Q8C4Q6 | Axin interactor, dorsalization-associated protein | Aida | 2899200 | 1446000 | 1764400 | 0 | 0 | 0 |
| Q8K2L8;Q8C0C3 | Trafficking protein particle complex subunit 12 | Trappc12 | 2891700 | 2655000 | 2069100 | 0 | 0 | 0 |
| P50428 | Arylsulfatase A | Arsa | 2889000 | 3070700 | 2791900 | 0 | 0 | 0 |
| B1AQD9;Q9Z2R6 | Protein unc-119 homolog A | Unc119 | 2859500 | 0 | 3916000 | 0 | 4413100 | 0 |
| Q9ER38;A0A087WRX4;A0A087WQB9;M0QWH2 | Torsin-3A | Tor3a | 2837600 | 2758700 | 3088700 | 0 | 2149200 | 0 |
| Q8K1X1;G5E8J3;A0A0U1RQ40 | WD repeat-containing protein 11 | Wdr11 | 2815200 | 2505400 | 2831500 | 0 | 0 | 0 |
| Q80YR5;F6WIZ2 | Scaffold attachment factor B2 | Safb2 | 2793200 | 2328700 | 3160400 | 0 | 2944100 | 0 |
| Q8BTS4 | Nuclear pore complex protein Nup54 | Nup54 | 2764600 | 0 | 4424500 | 0 | 0 | 0 |
| P97465;A0A0N4SW01;A0A0N4SW29 | Docking protein 1 | Dok1 | 2759000 | 3224800 | 3768900 | 0 | 0 | 3578700 |
| Q9D1I6 | 39S ribosomal protein L14, mitochondrial | Mrpl14 | 2745800 | 1820600 | 2197900 | 2365300 | 0 | 0 |
| Q8CI71 | Coiled-coil domain-containing protein 132 | Ccdc132 | 2741100 | 2599200 | 1685900 | 0 | 0 | 0 |
| Q61881;D3Z6N3;D3Z335;D3Z0J6;D3Z2K9 | DNA replication licensing factor MCM7 | Mcm7 | 2630000 | 3507700 | 0 | 0 | 0 | 0 |
| Q99LJ0 | CTTNBP2 N-terminal-like protein | Cttnbp2nl | 2580200 | 0 | 2843400 | 3063800 | 0 | 0 |
| Q8R4R6;A2ATJ2 | Nucleoporin NUP53 | Nup35 | 2517600 | 3206500 | 3177900 | 0 | 0 | 0 |
| Q8CBE3;H3BK66;Q05BF4;H3BJR7;H3BJS1 | WD repeat-containing protein 37 | Wdr37 | 2400600 | 1919900 | 2464600 | 2163400 | 0 | 0 |
| Q3UVG3 | Protein FAM91A1 | Fam91a1 | 2378600 | 3100800 | 3472400 | 3256800 | 0 | 0 |
| A2A9I0;O35166 | Golgi SNAP receptor complex member 2 | Gosr2 | 2283200 | 0 | 2843500 | 0 | 0 | 0 |
| A0A2U3TZ82;Q99KW3;A0A2R8VI29;G5E8W1 | TRIO and F-actin-binding protein | Triobp | 2216900 | 0 | 2095800 | 0 | 0 | 0 |
| F7AMS7;Q5SXG1;Q5SXG3;G3X9G2;Q9JM52;J3QM71 | Misshapen-like kinase 1 | Mink1 | 2167500 | 2866700 | 2316400 | 3172000 | 0 | 0 |
| Q8CB96;A0A0N4SVF1 | Ras association domain-containing protein 4 | Rassf4 | 2152200 | 2761300 | 0 | 0 | 0 | 0 |
| G3UX35;A0A0R4J170;Q3TKT4;E9QAB8;H3BJK2;F2Z4A9;H3BLH0;Q6DIC0 | Transcription activator BRG1;Probable global transcription activator SNF2L2 | Smarca4;Smarca2 | 2108100 | 2582700 | 2720700 | 2851000 | 0 | 0 |
| S4R1D4;B2RXW8;B2RXQ2 | Protein tyrosine phosphatase, receptor type, f polypeptide (PTPRF),-interacting protein (liprin), alpha 1 | Ppfia1 | 2094800 | 1772500 | 0 | 0 | 0 | 0 |
| B2RY04 | Dedicator of cytokinesis protein 5 | Dock5 | 2065300 | 2354100 | 2123100 | 0 | 0 | 0 |
| Q9WVL3 | Solute carrier family 12 member 7 | Slc12a7 | 2048300 | 2140600 | 2596000 | 0 | 0 | 0 |
| Q60770;A0A0G2JEI5 | Syntaxin-binding protein 3 | Stxbp3 | 1893400 | 1793400 | 2048500 | 0 | 0 | 0 |
| F6RTE1;Q9D0B0 | Serine/arginine-rich splicing factor 9 | Srsf9 | 1840600 | 0 | 1956800 | 0 | 0 | 0 |
| B9EKI5;Q3TTA7 | E3 ubiquitin-protein ligase CBL-B | Cblb | 1726400 | 1581500 | 1752000 | 0 | 0 | 0 |
| P22366;F6SPW1 | Myeloid differentiation primary response protein MyD88 | Myd88 | 1706600 | 1756800 | 1604300 | 0 | 0 | 0 |
| G5E8R4;Q922D4;A0A286YE88;A0A286YD27;A0A286YCG3 | Serine/threonine-protein phosphatase 6 regulatory subunit 3 | Ppp6r3 | 1631500 | 0 | 1704100 | 0 | 0 | 0 |
| Q8BVU5 | ADP-ribose pyrophosphatase, mitochondrial | Nudt9 | 1492800 | 1956800 | 2763100 | 0 | 0 | 0 |
| Q8BI72 | CDKN2A-interacting protein | Cdkn2aip | 1475200 | 1520200 | 1653900 | 1707800 | 0 | 0 |
| Q9D666;D3Z5Q2;D3Z3N5;D3YUW9;F7BX07;Q3TSM1;F6XYA0 | SUN domain-containing protein 1 | Sun1 | 1405900 | 0 | 1434500 | 0 | 0 | 0 |
| A2A7S8 | Uncharacterized protein KIAA1522 | Kiaa1522 | 1326400 | 4350000 | 520970 | 1408400 | 0 | 0 |
| B1AZA5;D3YWD3;B1AZA8 | Transmembrane protein 245 | Tmem245 | 1259800 | 0 | 1589300 | 0 | 0 | 0 |
| A0A0R4J0Q6;Q80VI1 | E3 ubiquitin-protein ligase TRIM56 | Trim56 | 1045700 | 962900 | 865710 | 1210900 | 0 | 0 |
| Q3T9A5;Q8BZM1;D6RGR3;D3Z0L3 | Glomulin | Glmn | 1033000 | 0 | 1349600 | 0 | 0 | 0 |
| A0A087WSB8;Q9DCB1 | High mobility group nucleosome-binding domain-containing protein 3 | Hmgn3 | 1013600 | 829740 | 0 | 0 | 0 | 0 |
| Q6P1H6 | Ankyrin repeat and LEM domain-containing protein 2 | Ankle2 | 1003900 | 1170800 | 1319800 | 0 | 0 | 0 |
| B0V2V6;A0A494BAK1;Q3UH70;Q9ESU6 | Bromodomain-containing protein 4 | Brd4 | 720530 | 1145600 | 1068700 | 1032700 | 0 | 0 |
| Q80SY5 | Pre-mRNA-splicing factor 38B | Prpf38b | 707160 | 662190 | 806030 | 0 | 489350 | 0 |
| A0A0R4J005;A0A5F8MPJ3;E9QP62;Q9D7B2;Q99JW4 | LIM and senescent cell antigen-like-containing domain protein 1 | Lims1 | 0 | 2976600 | 3646000 | 0 | 0 | 0 |
| Z4YL78;A0A0R4J0K2;K3W4R5;A2AGT5 | Cytoskeleton-associated protein 5 | Ckap5 | 0 | 3568200 | 3119300 | 0 | 0 | 0 |
| A6PWP8;A2AGQ2;A0A0R4J1F4;A2AGQ7;A2AGQ9;A2AGR0;A2AGQ5;A2AGQ6;E9QN47;A2AGQ4;Q80U28;A2AGR1;A2AGQ3;A2AGQ8 | MAP kinase-activating death domain protein | Madd | 0 | 1071800 | 1387300 | 0 | 0 | 0 |
| A0A0U1RQA5;A0A0U1RP20;A0A0U1RQA0;O35316 | Sodium- and chloride-dependent taurine transporter | Slc6a6 | 0 | 19292000 | 21266000 | 0 | 16825000 | 0 |
| A0A1B0GRG3;A0A1B0GSK8;O35566 | CD151 antigen | Cd151 | 0 | 2661100 | 3099600 | 0 | 0 | 0 |
| A0A1Y7VLY2;P59764 | Dedicator of cytokinesis protein 4 | Dock4 | 0 | 1745800 | 1521600 | 0 | 0 | 0 |
| A0A286YDB3;Q80XQ2 | TBC1 domain family member 5 | Tbc1d5 | 0 | 3014000 | 3742200 | 3224500 | 0 | 0 |
| A0A494B8Y8;Q91XB7;A0A494BA71;A0A494BA88;A0A494B9Y3 | Protein YIF1A | Yif1a | 0 | 3051200 | 2599400 | 0 | 0 | 0 |
| J3QMC5;A2ANY6 | Midasin | Mdn1 | 0 | 8216000 | 7752900 | 0 | 0 | 0 |
| B1APX2 | RIKEN cDNA 5031439G07 gene | 5031439G07Rik | 0 | 4505200 | 4540500 | 0 | 0 | 0 |
| D6RFN5;D3YW25;O70131 | Ninjurin-1 | Ninj1 | 0 | 2320300 | 2845100 | 0 | 3044300 | 0 |
| F6T9K1;D3YY09;Q9D6N5;D3YYC2;G3UXH5 | Dr1-associated corepressor | Drap1 | 0 | 4119800 | 5761900 | 0 | 5297700 | 0 |
| D6RG49;Q80XP8 | Protein FAM76B | Fam76b | 0 | 703180 | 695650 | 1329100 | 0 | 0 |
| E9PVP3;Q5F2B1;F6XX36;Q8R0J2;Q9R0Q9;F6ZGG4 | Mannose-P-dolichol utilization defect 1 protein | Mpdu1 | 0 | 7484500 | 9340400 | 0 | 5743100 | 0 |
| F7C134;E9Q9Y2;Q8CHB6;E9Q2B2;Q5RIM6;Q60974;Q3UV08 | Nuclear receptor corepressor 1 | Ncor1 | 0 | 1389400 | 1578400 | 0 | 1560900 | 0 |
| P62305;E9Q4F4;E9Q0W8 | Small nuclear ribonucleoprotein E | Snrpe | 0 | 16552000 | 15102000 | 29202000 | 0 | 0 |
| E9Q8I7;E9Q3K1;E9PZN0 | Nuclear transcription factor, X-box binding-like 1 | Nfxl1 | 0 | 19810000 | 20263000 | 0 | 0 | 0 |
| F6TCF9;Q60739 | BAG family molecular chaperone regulator 1 | Bag1 | 0 | 7023000 | 9200600 | 0 | 0 | 0 |
| F6XC25;Q8BRN9 | Coiled-coil and C2 domain-containing protein 1B | Cc2d1b | 0 | 4998300 | 5039100 | 0 | 0 | 6089600 |
| Q8C4V1;G3X9N1;D3Z5T4 | Rho GTPase-activating protein 24 | Arhgap24 | 0 | 2515400 | 2771000 | 0 | 0 | 0 |
| O35598;D3Z1E6;E9PYF2 | Disintegrin and metalloproteinase domain-containing protein 10 | Adam10 | 0 | 4377900 | 3981600 | 0 | 0 | 0 |
| S4R1L5;S4R2P8;O88738 | Baculoviral IAP repeat-containing protein 6 | Birc6 | 0 | 1113100 | 1442900 | 0 | 0 | 0 |
| O88851 | Putative hydrolase RBBP9 | Rbbp9 | 0 | 4102200 | 4148500 | 0 | 0 | 4196600 |
| Q05CL8;A2AMD0 | La-related protein 7 | Larp7 | 0 | 2441700 | 1623900 | 0 | 0 | 0 |
| Q3TCD4;Q9WUR2;E9PYC6;E9Q858;E9PUY9;E9PYA9;E9PVM6;E9Q7A8;E9PY42;Q78JN3 | Enoyl-CoA delta isomerase 2, mitochondrial | Eci2 | 0 | 3635800 | 4036100 | 0 | 0 | 0 |
| Q3UCV8;A0A2I3BRS5 | Ubiquitin thioesterase otulin | Otulin | 0 | 3351900 | 2899300 | 0 | 0 | 0 |
| Q5SUR0 | Phosphoribosylformylglycinamidine synthase | Pfas | 0 | 5418000 | 6051500 | 0 | 0 | 4301800 |
| Q6NZM8;Q60967;A0A0G2JFM1;A0A0G2JFK8 | Bifunctional 3-phosphoadenosine 5-phosphosulfate synthase 1;Sulfate adenylyltransferase;Adenylyl-sulfate kinase | Papss1 | 0 | 3194500 | 2835900 | 0 | 0 | 0 |
| Q6PB93 | Polypeptide N-acetylgalactosaminyltransferase 2;Polypeptide N-acetylgalactosaminyltransferase 2 soluble form | Galnt2 | 0 | 3993700 | 4133000 | 0 | 3786600 | 0 |
| Q7TMR0;A0A140LHY2 | Lysosomal Pro-X carboxypeptidase | Prcp | 0 | 4500700 | 4571900 | 0 | 0 | 0 |
| Q80VJ3 | 2-deoxynucleoside 5-phosphate N-hydrolase 1 | Dnph1 | 0 | 2731400 | 2687500 | 2844900 | 0 | 0 |
| Q8BTV2;A0A494B9T0 | Cleavage and polyadenylation specificity factor subunit 7 | Cpsf7 | 0 | 2069700 | 1928300 | 0 | 0 | 0 |
| Q8BWW3;Q9CYR6 | Phosphoacetylglucosamine mutase | Pgm3 | 0 | 6226000 | 5664700 | 0 | 0 | 0 |
| Q8CDM8;Q80YR2 | Protein FAM160B1 | Fam160b1 | 0 | 2312800 | 2493600 | 0 | 0 | 0 |
| Q8QZZ7;D3YXX8;A0A0J9YVB8;D3Z5E8;G3X9K8 | EKC/KEOPS complex subunit Tprkb | Tprkb | 0 | 2653000 | 2974300 | 2682500 | 0 | 0 |
| Q91WD5;D3YXT0;A0A0A6YW30 | NADH dehydrogenase [ubiquinone] iron-sulfur protein 2, mitochondrial | Ndufs2 | 0 | 7301700 | 7711900 | 0 | 0 | 0 |
| Q91WG2 | Rab GTPase-binding effector protein 2 | Rabep2 | 0 | 1610300 | 1748800 | 0 | 1955400 | 0 |
| Q91WT8;D3YXZ5;D3Z113 | RNA-binding protein 47 | Rbm47 | 0 | 3696700 | 3927200 | 0 | 0 | 0 |
| Q91YL3;F7CAK3;D6RJ84;B7ZCR1;F6V4H6 | Uridine-cytidine kinase-like 1;Uridine-cytidine kinase | Uckl1 | 0 | 2450300 | 3111500 | 0 | 0 | 3107800 |
| Q99J36 | THUMP domain-containing protein 1 | Thumpd1 | 0 | 5488400 | 5217400 | 0 | 0 | 0 |
| Q99K23;A0A1B0GSM1;A0A1B0GSK1 | Ufm1-specific protease 2 | Ufsp2 | 0 | 10297000 | 13037000 | 0 | 0 | 11194000 |
| Q99M71 | Mammalian ependymin-related protein 1 | Epdr1 | 0 | 1697300 | 1774200 | 0 | 0 | 0 |
| Q99MR8;A0A0G2JFQ5;A0A0G2JF64 | Methylcrotonoyl-CoA carboxylase subunit alpha, mitochondrial | Mccc1 | 0 | 4579100 | 4568400 | 0 | 0 | 0 |
| Q9CXE7;E9PXY3 | Transmembrane emp24 domain-containing protein 5 | Tmed5 | 0 | 14752000 | 14390000 | 0 | 0 | 0 |
| Q9D0G0 | 28S ribosomal protein S30, mitochondrial | Mrps30 | 0 | 1772900 | 2905900 | 0 | 0 | 0 |
| Q9DAW6 | U4/U6 small nuclear ribonucleoprotein Prp4 | Prpf4 | 0 | 6399800 | 5533600 | 0 | 0 | 0 |
| Q9JJA4;D3Z369 | Ribosome biogenesis protein WDR12 | Wdr12 | 0 | 5157500 | 4587700 | 0 | 0 | 0 |
| Q9JLV5;E9Q4T8;F6R0N8;F6ZZK0 | Cullin-3 | Cul3 | 0 | 1980500 | 2048100 | 0 | 0 | 0 |
| Q9R257;A0A140T8J4 | Heme-binding protein 1 | Hebp1 | 0 | 5263800 | 5390100 | 0 | 0 | 0 |
| Q9WUD1;A0A494BBF6;A0A494BBI6 | STIP1 homology and U box-containing protein 1 | Stub1 | 0 | 8952400 | 4225000 | 0 | 0 | 0 |
