# Supplementary material for: Intracellular iron accumulation facilitates mycobacterial infection in old mouse macrophages
Source: GeroScience. 2023 Dec 30;46(2):2739–54. doi: 10.1007/s11357-023-01048-1 (PMC10828278; doi:10.1007/s11357-023-01048-1)
Supplement: Supplementary file 6 — Supplementary file6 (DOCX 30 KB) [file 11357_2023_1048_MOESM6_ESM.docx]

**Supplementary Table 2B: Proteins Unique in *M.avium*-infected Young BMMs *vs M.avium*-infected Old BMMs**

| **Protein IDs** | **Protein names** | **Gene names** | ***M.avium*-infected Old BMMs** | | | ***M.avium*-infected Young BMMs** | | |
| --- | --- | --- | --- | --- | --- | --- | --- | --- |
|  |  |  | **LFQ intensity 1967_br2_tr1** | **LFQ intensity 1967_br2_tr2** | **LFQ intensity 1967_br2_tr3** | **LFQ intensity 1967_br4_tr1** | **LFQ intensity 1967_br4_tr2** | **LFQ intensity 1967_br4_tr3** |
| Q9CZU3 | Superkiller viralicidic activity 2-like 2 | Skiv2l2 | 48596000 | 0 | 0 | 36588000 | 46382000 | 0 |
| A0A3B2W7N5;F6U1P7;B2RWC4 | Leucine rich repeat containing 73 | Lrrc73 | 13816000 | 0 | 0 | 18619000 | 22562000 | 0 |
| A0A498WFS2;Q922Y1 | UBX domain-containing protein 1 | Ubxn1 | 12928000 | 0 | 0 | 0 | 9301500 | 14121000 |
| P63168;Q80ZS7 | Dynein light chain 1, cytoplasmic | Dynll1 | 10238000 | 0 | 0 | 19810000 | 19395000 | 36137000 |
| Q99J56 | Derlin-1 | Derl1 | 10047000 | 0 | 0 | 10027000 | 10004000 | 11123000 |
| B1ASZ3;Q64516;Q8C635;Q9WU65 | Glycerol kinase | Gyk;Gk | 9593200 | 0 | 0 | 12629000 | 10355000 | 11573000 |
| Q6NSR8;F6T2H5 | Probable aminopeptidase NPEPL1 | Npepl1 | 8251500 | 0 | 0 | 0 | 5692100 | 5798300 |
| A0A0N4SVK3;P62322 | U6 snRNA-associated Sm-like protein LSm5 | Lsm5 | 8163600 | 0 | 0 | 4633400 | 4798400 | 4623500 |
| Q80SZ7 | Guanine nucleotide-binding protein G(I)/G(S)/G(O) subunit gamma-5 | Gng5 | 7800300 | 0 | 0 | 14337000 | 15678000 | 13504000 |
| Q922K7;E9QN31;A0A0N4SW16 | Probable 28S rRNA (cytosine-C(5))-methyltransferase | Nop2 | 7052400 | 0 | 0 | 5498000 | 5115600 | 6373900 |
| Q9ES57;M0QWY6 | Cell surface glycoprotein CD200 receptor 1 | Cd200r1 | 4742000 | 0 | 0 | 6074200 | 6119500 | 0 |
| Q9CQA3 | Succinate dehydrogenase [ubiquinone] iron-sulfur subunit, mitochondrial | Sdhb | 4552900 | 0 | 0 | 3937400 | 3296200 | 3555600 |
| Q6KAR6;A0A1Y7VKY4 | Exocyst complex component 3 | Exoc3 | 4336000 | 0 | 0 | 4248700 | 0 | 3838600 |
| Q80TL7;B9EKJ3;A0A1W2P878 | Protein MON2 homolog | Mon2 | 1853900 | 0 | 0 | 1479800 | 1724900 | 0 |
| A0A0B4J1G1;E9Q415;A0A0B4J1E6;P08101 | Low affinity immunoglobulin gamma Fc region receptor II | Fcgr2b;Fcgr2 | 0 | 5235500 | 0 | 6572100 | 0 | 7575000 |
| A0A0R4J100;Q8CIM5 | G-protein coupled receptor 84 | Gpr84 | 0 | 0 | 0 | 0 | 8642100 | 7805200 |
| A0A1B0GT75;A0A1B0GR63;A0A1B0GSH4 | Charged multivesicular body protein 2A | Chmp2a | 0 | 0 | 0 | 10833000 | 5075900 | 0 |
| A0A2I3BPG9;P83882 | 60S ribosomal protein L36a | Rpl36a | 0 | 0 | 0 | 50485000 | 56484000 | 44809000 |
| A0A3Q4EH04;A0A3Q4L335;A0A3Q4L393;Q8BL97 | Serine/arginine-rich splicing factor 7 | Srsf7 | 0 | 0 | 0 | 12409000 | 12666000 | 10923000 |
| A0A494BBA8;Q642K5;A0A494B9Z0;P62862;P35545 | 40S ribosomal protein S30 | Fau | 0 | 0 | 0 | 77685000 | 67883000 | 65193000 |
| Q7M6W1;A3QM89;Q8K0T0 | Reticulon;Reticulon-1 | Rtn1 | 0 | 0 | 43438000 | 29247000 | 26945000 | 32843000 |
| Q9Z2C5;B1AW21;Q3UDN6 | Myotubularin | Mtm1 | 0 | 0 | 0 | 4425900 | 0 | 5295200 |
| B2RUP2;A0A0R4J257;A2A855;A2A858 | Protein unc-13 homolog D | Unc13d | 0 | 0 | 0 | 2297400 | 2194400 | 0 |
| D3YU60;E9QJW0;Q91VS7 | Microsomal glutathione S-transferase 1 | Mgst1 | 0 | 0 | 0 | 34473000 | 35226000 | 30299000 |
| D3YVK1;P15105 | Glutamine synthetase | Glul | 0 | 0 | 0 | 9300300 | 10742000 | 0 |
| D3YXK2;S4R1M2;S4R2Q5 | Scaffold attachment factor B1 | Safb | 0 | 0 | 6182300 | 0 | 7047600 | 8890600 |
| E9PYK3 | Protein mono-ADP-ribosyltransferase PARP4 | Parp4 | 0 | 0 | 0 | 0 | 4033800 | 4338600 |
| E9Q512;H3BJG4;Q8BVJ9;H3BJH8 | Thyroid hormone receptor interactor 11 | Trip11 | 0 | 0 | 2173800 | 1785900 | 1787800 | 0 |
| E9Q557;E9PZW0 | Desmoplakin | Dsp | 0 | 0 | 5004200 | 5817700 | 5512500 | 6698100 |
| Q8VDG7;E9QNW6;D3Z3Q4 | Platelet-activating factor acetylhydrolase 2, cytoplasmic | Pafah2 | 0 | 0 | 2285000 | 2816300 | 2762700 | 0 |
| F7CJN9;Q921I1;F7BAE9 | Serotransferrin | Trf;Tf | 0 | 0 | 0 | 167810000 | 147710000 | 184500000 |
| G3UXL2;Q9D7G0 | Ribose-phosphate pyrophosphokinase 1 | Prps1l3;Prps1 | 0 | 0 | 0 | 10549000 | 6533100 | 6752300 |
| H3BJL1;Q8K0Q5 | Rho GTPase-activating protein 18 | Arhgap18 | 0 | 0 | 0 | 1020600 | 1015600 | 0 |
| H3BL08;Q8C172 | Ceramide synthase 6 | Cers6 | 0 | 0 | 0 | 7680600 | 5147600 | 6144500 |
| Q6URW6;K3W4R2;A0A140LI60 | Myosin-14 | Myh14 | 0 | 0 | 0 | 39229000 | 39248000 | 0 |
| O35682;A0A0N4SW94 | Myeloid-associated differentiation marker | Myadm | 0 | 0 | 5705100 | 9619700 | 17914000 | 17623000 |
| O88811 | Signal transducing adapter molecule 2 | Stam2 | 0 | 219600000 | 0 | 193660000 | 156590000 | 190790000 |
| O88845 | A-kinase anchor protein 10, mitochondrial | Akap10 | 0 | 0 | 6258600 | 9243500 | 8009800 | 0 |
| Q8BTU6;P10630;A0A338P6X5;E9Q561;D6RJ60 | Eukaryotic initiation factor 4A-II;Eukaryotic initiation factor 4A-II, N-terminally processed | Eif4a2 | 0 | 0 | 0 | 10073000 | 10590000 | 11511000 |
| P11031 | Activated RNA polymerase II transcriptional coactivator p15 | Sub1 | 0 | 0 | 0 | 5856200 | 0 | 6558800 |
| P16254;A2AUM6 | Signal recognition particle 14 kDa protein;Signal recognition particle 14 kDa protein, N-terminally processed | Srp14 | 0 | 0 | 0 | 7971600 | 8383000 | 8610100 |
| P31324;H3BK84 | cAMP-dependent protein kinase type II-beta regulatory subunit | Prkar2b | 0 | 0 | 7189900 | 9451200 | 7799400 | 10369000 |
| P51855;A2AQN9;Q3UEE2 | Glutathione synthetase | Gss | 0 | 0 | 0 | 6834800 | 6493900 | 7342400 |
| P51885 | Lumican | Lum | 0 | 0 | 0 | 7144000 | 7227600 | 7955000 |
| P53395 | Lipoamide acyltransferase component of branched-chain alpha-keto acid dehydrogenase complex, mitochondrial | Dbt | 0 | 0 | 3604000 | 2770400 | 3906100 | 2816000 |
| P56382 | ATP synthase subunit epsilon, mitochondrial | Atp5e | 0 | 0 | 0 | 38853000 | 41702000 | 38442000 |
| P62141;A0A0J9YUU8;A0A0J9YUG2 | Serine/threonine-protein phosphatase PP1-beta catalytic subunit | Ppp1cb | 0 | 0 | 0 | 13238000 | 14656000 | 0 |
| P97333 | Neuropilin-1 | Nrp1 | 0 | 3721600 | 0 | 0 | 4141100 | 4640800 |
| P99028 | Cytochrome b-c1 complex subunit 6, mitochondrial | Uqcrh | 0 | 12864000 | 0 | 8915000 | 0 | 8526300 |
| Q02257 | Junction plakoglobin | Jup | 0 | 0 | 0 | 7585500 | 7268400 | 6713000 |
| Q31125 | Zinc transporter SLC39A7 | Slc39a7 | 0 | 0 | 0 | 9471100 | 11165000 | 9811000 |
| Q3TM89;Q6P8I4 | PEST proteolytic signal-containing nuclear protein | Pcnp | 0 | 0 | 3487700 | 0 | 7600800 | 8276300 |
| Q3UMT1;F6XWD4 | Protein phosphatase 1 regulatory subunit 12C | Ppp1r12c | 0 | 0 | 0 | 1946400 | 1839800 | 0 |
| Q5SQ20;Q9EQ61 | Pescadillo homolog | Pes1 | 0 | 0 | 0 | 2285200 | 3070500 | 0 |
| Q64523;Q6GSS7 | Histone H2A type 2-C;Histone H2A type 2-A | Hist2h2ac;Hist2h2aa1 | 0 | 0 | 0 | 126120000 | 88692000 | 83324000 |
| Q6P1Y9;Q5PPR2;Q8R3S6 | Exocyst complex component 1 | Exoc1 | 0 | 0 | 0 | 12883000 | 0 | 13452000 |
| Q6PA06;E9QND8 | Atlastin-2 | Atl2 | 0 | 0 | 0 | 0 | 2772100 | 2995100 |
| Q6PHN9 | Ras-related protein Rab-35 | Rab35 | 0 | 0 | 0 | 8540500 | 5489500 | 6174300 |
| Q6ZWV7 | 60S ribosomal protein L35 | Rpl35 | 0 | 0 | 0 | 109820000 | 103530000 | 99530000 |
| Q6ZWY3;D6RH49;D3YYB0 | 40S ribosomal protein S27-like;40S ribosomal protein S27 | Rps27l | 0 | 0 | 0 | 13527000 | 13231000 | 14504000 |
| Q80ZJ1 | Ras-related protein Rap-2a | Rap2a | 0 | 0 | 7300100 | 0 | 7297800 | 6899400 |
| Q8C754;G3UY33 | Vacuolar protein sorting-associated protein 52 homolog | Vps52 | 0 | 0 | 0 | 0 | 12885000 | 11351000 |
| Q8CJ40 | Rootletin | Crocc | 0 | 0 | 37958000 | 37063000 | 31885000 | 0 |
| Q8K003 | Translation machinery-associated protein 7 | Tma7 | 0 | 0 | 7006600 | 7524600 | 4890000 | 5532000 |
| Q8K211;A8Y5P1 | High affinity copper uptake protein 1 | Slc31a1 | 0 | 41544000 | 0 | 0 | 28729000 | 16381000 |
| Q99JP6;J3QQ00;Q501M9 | Homer protein homolog 3 | Homer3 | 0 | 2910800 | 0 | 3250100 | 0 | 3253600 |
| Q9CQW9 | Interferon-induced transmembrane protein 3 | Ifitm3 | 0 | 0 | 0 | 31920000 | 28096000 | 29984000 |
| Q9CTN4 | Rho-related BTB domain-containing protein 3 | Rhobtb3 | 0 | 0 | 0 | 56260000 | 48487000 | 69195000 |
| Q9CYN9 | Renin receptor | Atp6ap2 | 0 | 4351500 | 0 | 3853700 | 4767400 | 5332400 |
| Q9D0L7;D3Z5T2 | Armadillo repeat-containing protein 10 | Armc10 | 0 | 0 | 0 | 4138000 | 4940300 | 0 |
| Q9D8S4;A0A1L1SS58 | Oligoribonuclease, mitochondrial | Rexo2 | 0 | 0 | 0 | 15361000 | 11987000 | 16670000 |
| Q9DAV6;Q9D6A7;I7HJI5 | R86 | Serpinb9b | 0 | 0 | 0 | 5297900 | 5219400 | 5123700 |
| Q9EP72 | ER membrane protein complex subunit 7 | Emc7 | 0 | 7357900 | 0 | 8459700 | 0 | 5181900 |
| Q9JIP4;B1PL19 | Pannexin-1 | Panx1 | 0 | 2613800 | 0 | 3479600 | 0 | 4781700 |
| P45591 | Cofilin-2 | Cfl2 | 0 | 0 | 0 | 7920400 | 0 | 8061700 |
