# Supplementary material for: Intracellular iron accumulation facilitates mycobacterial infection in old mouse macrophages
Source: GeroScience. 2023 Dec 30;46(2):2739–54. doi: 10.1007/s11357-023-01048-1 (PMC10828278; doi:10.1007/s11357-023-01048-1)
Supplement: Supplementary file 7 — Supplementary file7 (DOCX 30 KB) [file 11357_2023_1048_MOESM7_ESM.docx]

**Supplementary Table 2C: Proteins with Differential Abundance (*M.avium*-infected Old BMMs *vs* *M.avium*-infected Young BMMs)**

| **-LOG(P-value)** | **Fold Change (Log2)** | **Protein IDs** | **Protein names** | **Gene names** | ***M.avium*-infected Old BMMs** | | | ***M.avium*-infected Young BMMs** | | |
| --- | --- | --- | --- | --- | --- | --- | --- | --- | --- | --- |
|  |  |  |  |  | **LFQ intensity 1967_br2_tr1** | **LFQ intensity 1967_br2_tr2** | **LFQ intensity 1967_br2_tr3** | **LFQ intensity 1967_br4_tr1** | **LFQ intensity 1967_br4_tr2** | **LFQ intensity 1967_br4_tr3** |
| 2.617003998 | -2.292580922 | Q80YQ8;D6RII5 | Protein RMD5 homolog A | Rmnd5a;Rmnd5b | 19.37436867 | 19.0943203 | 19.00730705 | 21.70885086 | 21.19364166 | NaN |
| 3.866325636 | -2.219575246 | E9Q0F0 | Keratin 78 | Krt78 | 25.7613678 | 25.97596359 | 25.5116539 | 28.04286003 | 28.0483799 | 27.8164711 |
| 1.139671363 | -2.131884893 | E9PZF4;Q80XB4;E9Q641;F7CXG1 | Nebulin-related-anchoring protein | Nrap | 30.43579102 | 30.4292202 | 28.64023781 | 30.67614937 | 32.57623672 | 32.64851761 |
| 3.455200889 | -1.883491516 | P15532;Q5NC80;Q5NC79 | Nucleoside diphosphate kinase A;Nucleoside diphosphate kinase | Nme1 | 23.1769886 | 23.0432682 | 22.99708939 | 25.03279877 | 25.18268204 | 24.65233994 |
| 1.916194327 | -1.80281798 | A0A3Q4EIB3;A0A3Q4EG45;E9PZA6 | Vomeronasal 2, receptor 113 | Vmn2r113 | NaN | 23.32251167 | 23.17602921 | 25.46098328 | 25.10481644 | 24.59046555 |
| 2.20215248 | -1.623612404 | Q64669 | NAD(P)H dehydrogenase [quinone] 1 | Nqo1 | 22.17517662 | 21.90657425 | NaN | 23.664814 | 23.37782478 | 23.95082474 |
| 3.412464985 | -1.581617991 | Q6ZWY8;A0A0N4SVF0 | Thymosin beta-10 | Tmsb10 | 26.35749817 | 26.17704582 | 26.47749138 | 27.93500137 | 27.71384621 | 28.10804176 |
| 2.581769474 | -1.551973343 | P62892 | 60S ribosomal protein L39 | Rpl39 | 24.32024002 | 24.44777298 | 24.53249168 | 26.26327324 | 26.15141487 | 25.5417366 |
| 3.493883171 | -1.548572222 | E9PV44;O35143 | ATPase inhibitor, mitochondrial | Atpif1 | 24.71439552 | 24.65512657 | 24.68618965 | 26.12827301 | 26.33934593 | NaN |
| 4.837335795 | -1.546961466 | G3X8R0;A0A494BBE3;Q60870 | Receptor expression-enhancing protein;Receptor expression-enhancing protein 5 | Reep5 | 23.80134201 | 23.67378998 | 23.664814 | 25.33817291 | 25.19212151 | 25.25053596 |
| 2.779873177 | -1.451624552 | F6RJ39;Q9JIX8;Q52KR6;B8JJ92;B8JJ91;B8JJ90;F6Q8C0;B8JJ89 | Apoptotic chromatin condensation inducer in the nucleus | Acin1 | 20.84643936 | 20.82919693 | 20.58389473 | 22.10255051 | 22.30705261 | NaN |
| 2.69817445 | -1.442610741 | Q8BMK4 | Cytoskeleton-associated protein 4 | Ckap4 | 21.0428009 | 21.16103363 | 21.14500999 | 22.38047028 | 22.73731422 | NaN |
| 3.526563731 | -1.435916265 | Q9JJI8 | 60S ribosomal protein L38 | Rpl38 | 24.74688721 | 24.50300217 | 24.40054321 | 25.95823669 | 26.11115837 | 25.88878632 |
| 2.840449595 | -1.399706523 | Q6IFX2 | Keratin, type I cytoskeletal 42 | Krt42 | 23.5961647 | 23.00573158 | 23.31589508 | 24.77951622 | 24.73804665 | 24.59934807 |
| 4.254192595 | -1.35659345 | Q9Z1R9 | Protease, serine 1 (trypsin 1) | Prss1 | 30.33372879 | 30.54765701 | 30.35457993 | 31.80578232 | 31.70479012 | 31.79517365 |
| 1.238048539 | -1.337272326 | Q60963;E9Q330;E9Q6J0 | Platelet-activating factor acetylhydrolase | Pla2g7 | 22.01065636 | NaN | 22.05649185 | 23.10896111 | 24.05531693 | 22.94826126 |
| 2.895283472 | -1.31954066 | Q9CPT4 | Myeloid-derived growth factor | Mydgf | 23.07846832 | 23.43109512 | 23.49899292 | 24.74857712 | 24.45926476 | 24.75933647 |
| 2.154117684 | -1.309363047 | O70250 | Phosphoglycerate mutase 2 | Pgam2 | 23.75133896 | 23.6284523 | 23.73024178 | 25.51665115 | 24.70213318 | 24.81933784 |
| 4.398181948 | -1.280948639 | Q8CGP6;C0HKE7;C0HKE1;C0HKE2;C0HKE3;C0HKE4;C0HKE5;C0HKE6;C0HKE9;A0A0N4SV66;Q8CGP4;C0HKE8;Q8BFU2;Q8CGP5;Q8CGP7;Q8R1M2;G3UWL7 | Histone H2A type 1-H;Histone H2A;Histone H2A type 3;Histone H2A type 1-F;Histone H2A type 1-K;Histone H2A.J | Hist1h2ah;Hist1h2aa;Hist3h2a;Hist1h2af;Hist1h2ak;H2afj | 28.83270645 | 28.95900536 | 28.96372414 | 30.17724037 | 30.29373169 | 30.1273098 |
| 3.624008129 | -1.249657949 | P15864;I7HFT9;Q07133 | Histone H1.2 | Hist1h1c | 27.361269 | 27.47305298 | 27.21891212 | 28.68708992 | 28.46645546 | 28.64866257 |
| 1.280994048 | -1.12443924 | A3KGL9;F6W687;P09602;Q5XK38;B7ZCQ3 | Non-histone chromosomal protein HMG-17 | Hmgn2 | 21.93268585 | 21.37469864 | 21.98046684 | 22.54070854 | 23.23340416 | NaN |
| 3.915043987 | -1.108048121 | P30115;A0A087WQI6;Q6P8Q0;P13745;D3Z6A6;D3YZV3;P24472;P10648 | Glutathione S-transferase A3 | Gsta3 | 25.08050346 | 25.04239082 | 25.04376793 | 26.31044388 | 26.10415649 | 26.07620621 |
| 2.397722087 | -1.101537069 | A0A0R4J027;P54987 | Cis-aconitate decarboxylase | Irg1 | 23.31617165 | 23.50008774 | 23.49472618 | 24.66876793 | 24.19212151 | 24.75470734 |
| 2.747063721 | -1.069428126 | E9Q421;Q8BXX3;Q8BL63;Q9CXY9 | GPI-anchor transamidase | Pigk | 21.47343063 | 21.46796989 | 21.68867683 | 22.5632782 | 22.6622963 | NaN |
| 4.323897146 | -1.047999064 | P61358;A2A4Q0 | 60S ribosomal protein L27 | Rpl27 | 25.31444359 | 25.30295372 | 25.31796646 | 26.25302696 | 26.38592529 | 26.44040871 |
| 2.977830054 | -1.035831134 | D3Z7C0;Q9Z2Q5 | 39S ribosomal protein L40, mitochondrial | Mrpl40 | NaN | 20.41874695 | 20.60458183 | 21.50562096 | 21.52348709 | 21.61337852 |
| 1.901602826 | -0.979902903 | Q3UV17 | Keratin, type II cytoskeletal 2 oral | Krt76 | 27.77536392 | 27.40356827 | 27.14365196 | 28.54159737 | 28.15228462 | 28.56841087 |
| 1.365803382 | -0.974561691 | Q3TTY5 | Keratin, type II cytoskeletal 2 epidermal | Krt2 | 26.26441956 | 26.35867119 | 26.13881683 | 26.67033577 | 27.21196747 | 27.80328941 |
| 2.13773596 | 0.971213341 | Q6PAH4;Q61124;D3Z448;D3YUU4 | Battenin | Cln3 | 22.55327988 | 22.38847923 | 22.71613503 | 21.46777153 | 21.69506454 | NaN |
| 4.067549352 | 0.975489299 | O70251;A0A087WS46;G3UX43;M0QWK5;M0QWH8;G3UZ47 | Elongation factor 1-beta | Eef1b;Eef1b2 | 26.51978874 | 26.54767609 | 26.43869781 | 25.43023491 | 25.54724884 | 25.602211 |
| 1.478261055 | 1.010913213 | E9QMX7;Q640N3 | Rho GTPase-activating protein 30 | Arhgap30 | 23.93544006 | 23.3001976 | 23.91788864 | 22.24263573 | 22.84483147 | 23.03331947 |
| 2.21649493 | 1.038870494 | D3Z7M9;O35381;F6UFG6;D3YYE1;Q64G17 | Acidic leucine-rich nuclear phosphoprotein 32 family member A | Anp32a | 23.85071754 | 23.7205677 | 24.1152935 | 22.55683899 | 23.09173012 | 22.92139816 |
| 2.79181804 | 1.039418221 | P00416 | Cytochrome c oxidase subunit 3 | mt-Co3 | 24.03405952 | 23.94136429 | 23.86712265 | 22.81260681 | 23.00358772 | NaN |
| 1.656026035 | 1.043865204 | O70404;A0A0R4J0R1;A0A0U1RPE8 | Vesicle-associated membrane protein 8 | Vamp8 | 25.79525948 | 25.31848335 | 26.30199432 | 24.84350014 | 24.74401474 | 24.69662666 |
| 2.150070795 | 1.091796875 | Q9R0Q3;F6V6T4;Q8BPI2 | Transmembrane emp24 domain-containing protein 2 | Tmed2 | 25.89504242 | 25.85155296 | 25.58683395 | 24.40749741 | 25.05448914 | 24.59605217 |
| 3.487716671 | 1.222342809 | Q9D0M5;D6RIN4 | Dynein light chain 2, cytoplasmic | Dynll2 | 25.39832687 | 25.56465149 | 25.75490952 | 24.30445099 | 24.35265732 | 24.39375114 |
| 2.588474128 | 1.258879344 | A0A0U1RP13;Q8VDP6;A0A0U1RNI6;A0A0U1RPV3;A0A0U1RQ57;A0A0U1RP60;Q05BY1 | CDP-diacylglycerol--inositol 3-phosphatidyltransferase | Cdipt | 25.40610123 | 24.91255379 | 25.45863914 | 23.9207077 | 24.14172363 | 23.93822479 |
| 3.20705529 | 1.28263092 | P63213 | Guanine nucleotide-binding protein G(I)/G(S)/G(O) subunit gamma-2 | Gng2 | 25.80272293 | 25.99524498 | 25.99688148 | 24.63931465 | 24.45525551 | 24.85238647 |
| 3.376972338 | 1.321831385 | Q9CQN6 | Transmembrane protein 14C | Tmem14c | 24.57824898 | 24.40151978 | 24.43853951 | 23.23232269 | 22.93292046 | 23.28757095 |
| 2.533748769 | 1.351870855 | P45878 | Peptidyl-prolyl cis-trans isomerase FKBP2 | Fkbp2 | 25.89295959 | 25.71792984 | 26.3495903 | 24.54710197 | 24.81484795 | 24.54291725 |
| 2.775335682 | 1.362817764 | P27661 | Histone H2AX | H2afx | 25.10033035 | 25.29217911 | 25.39326096 | 24.09273148 | 23.58233261 | 24.02225304 |
| 4.538446469 | 1.433216731 | Q3U9N4;P28798;H3BJE0;H3BJ90;H3BLC9 | Granulins;Acrogranin;Granulin-1;Granulin-2;Granulin-3;Granulin-4;Granulin-5;Granulin-6;Granulin-7 | Grn | 28.21609116 | 28.27426529 | 28.25921059 | 26.7551899 | 26.74791145 | 26.94681549 |
| 3.285473561 | 1.450494766 | A8DUK4;P02089;P02088;E9Q223;P02104 | Hemoglobin subunit beta-2;Hemoglobin subunit beta-1;Hemoglobin subunit epsilon-Y2 | Hbbt1;Hbb-b2;Hbb-b1;Hbb-bs;Hbb-y | 28.42282104 | 28.25705338 | 28.12558746 | 26.87970161 | 26.9755249 | 26.59875107 |
| 3.619411189 | 1.509579341 | P62897 | Cytochrome c, somatic | Cycs | 25.27415848 | 25.44830894 | 25.30448532 | 23.61930084 | 23.97640038 | 23.9025135 |
| 3.771583222 | 1.521137238 | A0A0G2JEK2;P63254 | Cysteine-rich protein 1 | Crip1 | 25.6235218 | 25.53195763 | 25.84864235 | 24.2677803 | 24.08950233 | 24.08342743 |
| 3.802674119 | 1.568623861 | Q8R5L1;O35658 | Complement component 1 Q subcomponent-binding protein, mitochondrial | C1qbp | 25.56975746 | 25.74868011 | 25.58009148 | 23.87895393 | 24.20828819 | 24.10541534 |
| 4.925277733 | 2.643924713 | Q61878 | Bone marrow proteoglycan;Eosinophil granule major basic protein | Prg2 | 28.50963974 | 28.37264442 | 28.49067879 | 25.97790718 | 25.66903877 | 25.79424286 |
| 3.751927272 | 3.204519908 | P49290 | Eosinophil peroxidase;Eosinophil peroxidase light chain;Eosinophil peroxidase heavy chain | Epx | 25.54512978 | 25.41228676 | 25.5932045 | 21.86955261 | 22.6545372 | 22.4129715 |
| 3.094906235 | 4.148032506 | P11247;F7DC05 | Myeloperoxidase;Myeloperoxidase light chain;Myeloperoxidase heavy chain | Mpo | 24.43415451 | 24.45801353 | 24.4730835 | 20.03992653 | 19.6873455 | 21.19388199 |
