# Supplementary material for: Intracellular iron accumulation facilitates mycobacterial infection in old mouse macrophages
Source: GeroScience. 2023 Dec 30;46(2):2739–54. doi: 10.1007/s11357-023-01048-1 (PMC10828278; doi:10.1007/s11357-023-01048-1)
Supplement: Supplementary file 8 — Supplementary file8 (DOCX 616 KB) [file 11357_2023_1048_MOESM8_ESM.docx]

| **Significant** | **-LOG(P-value)** | **Fold Change (Log2)** | **Protein IDs** | **Protein names** | **Gene names** | ***M.avium*-infected old BMMs** | | | ***M.avium*-infected Young BMMs** | | |
| --- | --- | --- | --- | --- | --- | --- | --- | --- | --- | --- | --- |
|  |  |  |  |  |  | LFQ intensity 1967_br2_tr1 | LFQ intensity 1967_br2_tr2 | LFQ intensity 1967_br2_tr3 | LFQ intensity 1967_br4_tr1 | LFQ intensity 1967_br4_tr2 | LFQ intensity 1967_br4_tr3 |
| **+** | 2.620488952 | -0.898681005 | P50446;Q3UV11;Q9Z331;D3Z6R0 | Keratin, type II cytoskeletal 6A;Keratin, type II cytoskeletal 6B | Krt6a;Krt6b | 24.63787842 | 24.30528641 | 24.41293144 | 25.45145607 | 25.42460823 | 25.17607498 |
| **+** | 2.305858649 | -0.882251104 | P55258;Q3TYH2;Q8K386 | Ras-related protein Rab-8A | Rab8a | 23.93490028 | 23.47357941 | 23.72338295 | 24.6031456 | 24.73148537 | 24.44398499 |
| **+** | 3.393239967 | -0.876296361 | Q3U3M9;Q9JKF4 | C-type lectin domain family 6 member A | Clec4n;Clec6a | 22.05105782 | 22.02248955 | 22.1213932 | NaN | 22.98492241 | 22.89763069 |
| **+** | 3.016650597 | -0.859976451 | P16460 | Argininosuccinate synthase | Ass1 | 23.87032509 | 23.6327858 | 23.84833145 | 24.76111412 | 24.62734032 | 24.54291725 |
| **+** | 3.499792143 | -0.832377752 | A0A087WRZ7;A0A0G2JDM3;E9PWG4;A0A0G2JDW2;P05977;P09542 | Myosin light chain 1/3, skeletal muscle isoform;Myosin light chain 3 | Myl1;Myl3 | 25.89409447 | 25.92129707 | 26.03124237 | 26.88168716 | 26.78231812 | 26.67976189 |
| **+** | 1.383215821 | -0.789775848 | G3X8Y8;Q9QUN7 | Toll-like receptor;Toll-like receptor 2 | Tlr2 | 21.91404152 | 22.23829651 | NaN | NaN | 22.83101463 | 22.90087509 |
| **+** | 4.834357339 | -0.780474981 | Q922U2;P07744 | Keratin, type II cytoskeletal 5 | Krt5 | 27.8696785 | 27.90821838 | 27.88291168 | 28.69981956 | 28.69242096 | 28.60999298 |
| **+** | 2.652794236 | -0.754992167 | P17897 | Lysozyme C-1 | Lyz1 | 24.71759033 | 24.9583683 | 25.04122162 | 25.6725769 | 25.73727417 | 25.57230568 |
| **+** | 3.525317724 | -0.695192337 | Q8K354;A0A338P797;A0A1B0GRG8 | Carbonyl reductase [NADPH] 3 | Cbr3 | 24.42271614 | 24.52049255 | 24.49112129 | 25.26920891 | 25.1578064 | 25.09289169 |
| **+** | 2.194246903 | -0.685851415 | A0A0R4J2B2;Q6WVG3 | BTB/POZ domain-containing protein KCTD12 | Kctd12 | 24.42892456 | 24.27027893 | 24.68570709 | 25.07332802 | 25.12486649 | 25.24427032 |
| **+** | 3.623027207 | -0.661931992 | Q32NZ6 | Transmembrane channel-like protein 5 | Tmc5 | 29.26918983 | 29.18539238 | 29.28502464 | 29.82926369 | 29.97787476 | 29.91826439 |
| **+** | 1.434024207 | -0.660909017 | A0A0G2JDW7;Q6ZWU9;A0A0G2JG29;A0A0G2JEX7 | 40S ribosomal protein S27 | Rps27 | 25.56578445 | 25.04071999 | 25.07009697 | 26.13610077 | 25.69803047 | 25.82519722 |
| **+** | 2.158950034 | -0.651596705 | A0A1L1SRX2;O08739;F6XQD0;D3Z4N3;D3YU73 | AMP deaminase 3 | Ampd3 | 22.52649879 | 22.562603 | 22.77782059 | 23.15744591 | 23.47407341 | 23.19019318 |
| **+** | 1.454912759 | -0.649644216 | Q9CQD1 | Ras-related protein Rab-5A | Rab5a | 23.00309181 | 23.3269062 | 23.44227791 | 23.77956581 | 24.22567558 | 23.71596718 |
| **+** | 1.384606922 | -0.649230957 | O35955 | Proteasome subunit beta type-10 | Psmb10 | NaN | 21.80017662 | 21.76093102 | 22.29510498 | 22.56446457 | NaN |
| **+** | 1.304279575 | -0.647236506 | Q9JIW9;F6QC68 | Ras-related protein Ral-B | Ralb | 22.45848846 | 22.92174339 | 22.72592163 | 23.46076393 | 23.60783577 | 22.97926331 |
| **+** | 5.387639305 | -0.64720281 | P62631 | Elongation factor 1-alpha 2 | Eef1a2 | 23.9762249 | 23.97018051 | 23.9358902 | 24.61093521 | 24.63017654 | 24.58279228 |
| **+** | 2.545534429 | -0.63931338 | P01029 | Complement C4-B;Complement C4 beta chain;Complement C4 alpha chain;C4a anaphylatoxin;Complement C4 gamma chain | C4b | 24.95033646 | 24.75445175 | 24.97648811 | 25.55838776 | 25.403862 | 25.63696671 |
| **+** | 1.571064984 | -0.628979365 | P32261;A0A0A6YXS8;A0A0A6YX49;A0A0A6YWH7 | Antithrombin-III | Serpinc1 | 23.58279228 | 23.99213409 | 23.58657646 | 24.13437843 | 24.34935379 | 24.56470871 |
| **+** | 2.624919922 | -0.626054128 | Q9Z2K1 | Keratin, type I cytoskeletal 16 | Krt16 | 24.09458351 | 24.20441246 | 24.1985054 | 24.89180183 | 24.85908508 | 24.62477684 |
| **+** | 3.711515629 | -0.625858943 | P19973;A2A6J7;A2A6J4;A0A1B0GRF5 | Lymphocyte-specific protein 1 | Lsp1 | 25.25893211 | 25.23534966 | 25.30413818 | 25.83079338 | 25.86983109 | 25.97537231 |
| **+** | 3.636433206 | -0.619772593 | P08228 | Superoxide dismutase [Cu-Zn] | Sod1 | 26.83581161 | 26.85578728 | 26.91843414 | 27.50506401 | 27.55471802 | 27.40956879 |
| **+** | 4.990924163 | -0.615649541 | P24270;A2AL20 | Catalase | Cat | 26.4449482 | 26.41060448 | 26.41874886 | 27.051548 | 27.06763649 | 27.00206566 |
| **+** | 3.137691277 | -0.610493342 | Q9D379;E9PWK1;F6YTS6;D3Z4M3 | Epoxide hydrolase 1 | Ephx1 | 23.79788399 | 23.84287643 | 23.69731522 | 24.29165268 | 24.43936348 | 24.43853951 |
| **+** | 1.707161561 | -0.608537038 | V9GX81 | Maestro heat-like repeat family member 6 | Mroh6 | 31.73036385 | 31.9693222 | 31.86060524 | 32.59450531 | 32.32942963 | NaN |
| **+** | 2.329020145 | -0.60282135 | A0A0R4J0P5;P97814 | Proline-serine-threonine phosphatase-interacting protein 1 | Pstpip1 | 22.68365288 | 22.46048927 | 22.52217102 | 23.03526688 | 23.31437492 | 23.12513542 |
| **+** | 1.384622392 | -0.594086329 | Q9D0F3;D3Z5B9 | Protein ERGIC-53 | Lman1 | 23.48012924 | 23.59923363 | 23.56185722 | 23.85842133 | 24.52037239 | 24.04468536 |
| **+** | 1.71127796 | -0.590622266 | P97494 | Glutamate--cysteine ligase catalytic subunit | Gclc | 22.63506889 | 22.49964905 | NaN | 23.15109253 | 23.32017136 | 23.00267982 |
| **+** | 2.055005757 | -0.588539124 | P61924 | Coatomer subunit zeta-1 | Copz1 | 24.19557953 | 24.45318413 | 24.18555832 | 24.76379967 | 25.03904724 | 24.79709244 |
| **+** | 2.294330902 | -0.588294347 | Q91VB8;P01942;A7M7S6;P06467 | Hemoglobin subunit alpha | haemaglobin alpha 2;Hba | 27.83599281 | 28.01712036 | 28.08278847 | 28.61820984 | 28.66421127 | 28.41836357 |
| **+** | 2.870365204 | -0.586137136 | P68404;Q4VA93;P20444;Q3TQ39;Q2NKI4;P63318 | Protein kinase C beta type | Prkcb | 22.48012924 | 22.55606651 | 22.50341606 | 23.06174088 | 23.23511505 | 23.0011673 |
| **+** | 1.588822925 | -0.583214442 | Q3UUQ7 | GPI inositol-deacylase | Pgap1 | 21.32251167 | 21.46183968 | 21.73554039 | 22.17188263 | 22.23841476 | 21.85923767 |
| **+** | 2.242769089 | -0.579528173 | D3YTP8;Q9CY46;Q9QXA5;A0A1B0GRR9;A0A1B0GQZ9 | U6 snRNA-associated Sm-like protein LSm4 | Lsm4 | 22.14814758 | 21.97956085 | NaN | 22.58721733 | 22.62169456 | 22.72123528 |
| **+** | 2.19108858 | -0.577060699 | A0A1W2P6H1;Z4YJE4;E9QQ96;H7BX05;A2AAJ9;J9JIB2 | Obscurin | Obscn | 24.91360474 | NaN | 24.87258148 | 25.51192474 | NaN | 25.42838287 |
| **+** | 2.745095395 | -0.57178688 | Q9D2R0 | Acetoacetyl-CoA synthetase | Aacs | 22.03634071 | 22.13260651 | 22.01903152 | 22.59509468 | 22.53962135 | 22.76862335 |
| **+** | 2.52308246 | -0.571704229 | P56135;F8WHP8 | ATP synthase subunit f, mitochondrial | Atp5j2 | 25.49277306 | 25.23472786 | 25.3824501 | 26.01107407 | 25.9644413 | 25.84954834 |
| **+** | 2.110393216 | -0.569284439 | A0A0E2WA25 | Chaperone protein HtpG | htpG | 27.44419098 | 27.66023827 | 27.78637505 | 28.17145348 | 28.3091259 | 28.11807823 |
| **+** | 2.409697587 | -0.565855026 | P62900;A0A0A6YX26;A0A0A6YXL3 | 60S ribosomal protein L31 | Rpl31 | 26.5532093 | 26.4856205 | 26.50168228 | 27.07948494 | 27.23890686 | 26.91968536 |
| **+** | 1.482117494 | -0.565227509 | O08797 | SPI6 | Serpinb9 | 22.78672028 | 22.87565804 | 22.3372612 | 23.18821716 | 23.34827232 | 23.15883255 |
| **+** | 1.530128467 | -0.564271291 | P97298;Q5ND37;B7ZC25;F6S1M4;E9PWS2 | Pigment epithelium-derived factor | Serpinf1 | 23.54356766 | 23.05334663 | 23.3269062 | 23.96305275 | 23.68410301 | 23.96947861 |
| **+** | 1.821739706 | -0.56409963 | P56212;E9Q4B9;E9Q827;E9PXD7 | cAMP-regulated phosphoprotein 19 | Arpp19 | 23.23139954 | 23.40184593 | 23.17797661 | 24.03875351 | 23.84345245 | 23.621315 |
| **+** | 1.59386023 | -0.557053884 | A2AQU8;Q9D975 | Sulfiredoxin-1 | Srxn1 | 24.1820755 | 23.77695847 | 24.29705048 | 24.61178017 | 24.69975281 | 24.61571312 |
| **+** | 1.23738511 | -0.554416656 | P43274 | Histone H1.4 | Hist1h1e | 26.46932983 | 27.02453613 | 26.84194183 | 27.5676136 | 27.10858917 | 27.322855 |
| **+** | 4.158573356 | -0.554385503 | Q8BG07 | Phospholipase D4 | Pld4 | 25.17001152 | 25.22493935 | 25.25796127 | 25.74273109 | 25.76311684 | 25.81022072 |
| **+** | 2.271983949 | -0.551046371 | Q9D6Y9;F6ZHD8;G3UW30 | 1,4-alpha-glucan-branching enzyme | Gbe1 | 22.97741318 | 23.1227684 | 22.82362938 | 23.56674194 | 23.58588982 | 23.42431831 |
| **+** | 3.72491905 | -0.549863815 | P35700;B1AXW5;B1AXW6;B1AXW4 | Peroxiredoxin-1 | Prdx1 | 31.51643753 | 31.44835281 | 31.43126488 | 32.00951767 | 32.07396317 | 31.96216583 |
| **+** | 1.860209624 | -0.545941671 | Q9D1R9;A0A0G2JGY8;A0A0G2JEY6 | 60S ribosomal protein L34 | Rpl34 | 25.43182945 | 25.81699562 | 25.77846336 | 26.29598427 | 26.22668457 | 26.14244461 |
| **+** | 2.60900082 | -0.543460846 | Q8VED5 | Keratin, type II cytoskeletal 79 | Krt79 | 25.60413551 | 25.59650612 | 25.72379875 | 26.07030106 | 26.17683601 | 26.30768585 |
| **+** | 2.180568102 | -0.536728541 | A2A547;P84099 | Ribosomal protein L19;60S ribosomal protein L19 | Rpl19 | 26.15826988 | 25.85133743 | 25.85835075 | 26.53232956 | 26.45535088 | 26.49046326 |
| **+** | 2.087071009 | -0.532587687 | Q6NXH9 | Keratin, type II cytoskeletal 73 | Krt73 | 28.94756889 | 28.95351982 | 29.01478195 | 29.70938301 | 29.34794426 | 29.45630646 |
| **+** | 2.995645637 | -0.52645429 | A2A513;P02535;B1ATJ5;Q61897;Q62168;Q61765;Q6IFX3;Q8VCW2;Q9Z320;Q497I4 | Keratin, type I cytoskeletal 10 | Krt10 | 26.27430153 | 26.12143326 | 26.0759201 | 26.65853691 | 26.69524574 | 26.69723511 |
| **+** | 1.90867799 | -0.526169459 | Q9CQ10;A0A0N4SVS3 | Charged multivesicular body protein 3 | Chmp3 | 22.578722 | 22.68393135 | 22.78200722 | 23.07837105 | 23.41866875 | 23.12612915 |
| **+** | 2.527686717 | -0.517129262 | E9QPD7;G5E8R3;Q05920;A0A286YCC5;A0A494B912 | Pyruvate carboxylase;Pyruvate carboxylase, mitochondrial | Pcx;Pc | 22.30056 | 22.52988625 | 22.35032463 | 22.91044235 | 22.84224319 | 22.97947311 |
| **+** | 1.213534778 | -0.506320953 | Q6NZB0;A2ALF0;A2ALF3;F6TQL3;F7CXJ2;F6QIL6;D3Z4I5 | DnaJ homolog subfamily C member 8 | Dnajc8 | 22.22922897 | 22.34082413 | NaN | 22.9102211 | 22.67247391 | NaN |
| **+** | 3.305986781 | -0.505087535 | A0A3Q4EBK4;Q6P1B9;O08539;A0A3Q4EBR8 | Myc box-dependent-interacting protein 1 | Bin1 | 23.88362312 | 23.89235878 | 23.85728264 | 24.28911972 | 24.41415787 | 24.44524956 |
| **+** | 1.457079966 | -0.504401525 | A2AMH3;A2AMH5;A2AMH4;Q6X893 | Choline transporter-like protein 1 | Slc44a1 | 22.06167603 | 22.51888466 | 22.50688171 | 22.83794022 | 22.97608566 | 22.78662109 |
| **+** | 1.75264281 | -0.500612895 | P26043;A0A5F8MPB9;Q7TSG6 | Radixin | Rdx | 23.54285812 | 23.10694885 | 23.35299301 | 23.86022377 | 23.78466606 | 23.85974884 |
| **+** | 2.540147711 | -0.496658325 | O09167 | 60S ribosomal protein L21 | Rpl21 | 26.4587326 | 26.27854347 | 26.45846748 | 26.91102219 | 26.80673409 | 26.96796227 |
| **+** | 2.243834163 | -0.49187088 | P84104 | Serine/arginine-rich splicing factor 3 | Srsf3 | 24.82987404 | 24.85481262 | 24.91150284 | 25.53302765 | 25.26885223 | 25.26992226 |
| **+** | 2.498904842 | -0.487277985 | P04104 | Keratin, type II cytoskeletal 1 | Krt1 | 28.09913635 | 27.93769836 | 28.13731384 | 28.50982857 | 28.48857307 | 28.63758087 |
| **+** | 1.580657845 | -0.48625501 | Q9D881;P19536;F7C106;A0A0A6YVR0 | Cytochrome c oxidase subunit 5B, mitochondrial | Cox5b | 25.14534378 | 25.04109573 | 25.04084587 | 25.51150322 | 25.82040977 | 25.35413742 |
| **+** | 0.98798469 | -0.480850856 | A0A0G2JGD2;P07091 | Protein S100-A4 | S100a4 | 29.58448601 | 29.36268997 | 28.90979195 | 29.71968269 | 29.98046494 | 29.59937286 |
| **+** | 2.889508872 | -0.474404017 | D3YUT3;D3Z5R8;D3Z722;Q9CZX8;D3YUG3;S4R223 | 40S ribosomal protein S19 | Rps19 | 25.9583683 | 25.82905197 | 25.99455452 | 26.34440041 | 26.4111557 | 26.44963074 |
| **+** | 2.759250416 | -0.46392568 | P47963 | 60S ribosomal protein L13 | Rpl13 | 26.62533379 | 26.81139755 | 26.67949295 | 27.21549034 | 27.11624527 | 27.17626572 |
| **+** | 1.397073969 | -0.463733037 | P97450;E9QAD6 | ATP synthase-coupling factor 6, mitochondrial | Atp5j | 24.99933624 | 25.48250008 | 25.17945862 | 25.65294075 | 25.80700493 | 25.59254837 |
| **+** | 1.823931 | -0.457566579 | Q76LS9 | Protein FAM63A | Fam63a | 23.29501915 | 23.41789627 | 23.5741539 | 23.73541641 | 23.99170303 | 23.93264961 |
| **+** | 2.405257884 | -0.454037348 | P62301;Q921R2;A0A0U1RQ71 | 40S ribosomal protein S13 | Rps13 | 25.44230843 | 25.51593018 | 25.6395359 | 26.06776047 | 25.89675331 | 25.99537277 |
| **+** | 1.893085733 | -0.453756968 | Q9D2V7;G3X9L5;E9PYU1 | Coronin-7 | Coro7 | 23.88464737 | 24.12036514 | 24.07128334 | 24.36766434 | 24.62895393 | 24.44094849 |
| **+** | 1.440396055 | -0.449672063 | Q3TWW8;A0A0A6YXX6 | Serine/arginine-rich splicing factor 6 | Srsf6 | 24.11870193 | 24.57149506 | 24.45349884 | 24.7634964 | 24.93247032 | 24.7967453 |
| **+** | 1.228614679 | -0.449305852 | Q9CQH7 | Transcription factor BTF3 homolog 4 | Btf3l4 | 23.59571075 | 23.98015404 | 23.6557827 | 23.99464035 | 24.41969872 | 24.16522598 |
| **+** | 3.393160967 | -0.445363363 | A0A1B0GR11;Q93092 | Transaldolase | Taldo1 | 27.37980843 | 27.37947845 | 27.42558479 | 27.91027832 | 27.77987862 | 27.83080482 |
| **+** | 1.767276955 | -0.444922129 | Q60676;F7BX26 | Serine/threonine-protein phosphatase 5;Serine/threonine-protein phosphatase | Ppp5c | 22.88498306 | 22.88838768 | 22.9560833 | 23.15497589 | 23.37158966 | 23.53765488 |
| **+** | 3.386580075 | -0.442614873 | P99029;G3UZJ4;H3BJQ7;A0A494BAZ4 | Peroxiredoxin-5, mitochondrial | Prdx5 | 26.39329338 | 26.3998909 | 26.35580254 | 26.8411026 | 26.75264168 | 26.88308716 |
| **+** | 2.525867159 | -0.442086538 | Q923D2;E9PZC3;E9PZC4;A0A0U1RPU7 | Flavin reductase (NADPH) | Blvrb | 27.18120384 | 27.03820992 | 27.19554138 | 27.64175415 | 27.48876381 | 27.61069679 |
| **+** | 1.294834667 | -0.439637502 | Q2TBE6;A0A494BBQ4 | Phosphatidylinositol 4-kinase type 2-alpha | Pi4k2a | 22.35156631 | 22.32300568 | 22.53547287 | 22.68230438 | 22.71519279 | 23.13146019 |
| **+** | 1.008972873 | -0.43746376 | Q8C2Q3;E9QL13;J3QN51;J3QPT3;B0LM42;F7BGR7;J3QQ01 | RNA-binding protein 14 | Rbm14 | 22.75158501 | 22.79808044 | 23.05077553 | 23.03759766 | 23.22568893 | 23.64954567 |
| **+** | 2.224904868 | -0.435091019 | Q8BH02;A0A0A6YWQ0 | Torsin-4A | Tor4a | 22.0444355 | 22.08076286 | NaN | 22.52611732 | 22.46926308 | NaN |
| **+** | 2.062221389 | -0.435009638 | P62320;A0A1W2P7K5 | Small nuclear ribonucleoprotein Sm D3 | Snrpd3 | 24.47097588 | 24.39897919 | 24.66811943 | 24.95482445 | 24.87192345 | 25.01635551 |
| **+** | 5.297158946 | -0.434999466 | E9QN37;A1L314;Q5RKV8 | Macrophage-expressed gene 1 protein | Mpeg1 | 26.56974411 | 26.53596687 | 26.56726456 | 26.98836899 | 26.98239708 | 27.00720787 |
| **+** | 1.108811027 | -0.434517543 | O70152;A2BDX2;F8WII3 | Dolichol-phosphate mannosyltransferase subunit 1 | Dpm1 | 22.88285828 | 22.88992882 | 22.52692986 | 23.12507248 | NaN | 23.27710724 |
| **+** | 1.461345011 | -0.433665593 | P28667 | MARCKS-related protein | Marcksl1 | 23.17511559 | 23.3899498 | 23.48234558 | 23.77625465 | 23.78935051 | NaN |
| **+** | 1.422269372 | -0.433050791 | Q9CQN1 | Heat shock protein 75 kDa, mitochondrial | Trap1 | 23.50615501 | 23.40807915 | 23.36372566 | 23.65009499 | 23.81450462 | 24.11251259 |
| **+** | 1.836646207 | -0.432401021 | Q91WS0 | CDGSH iron-sulfur domain-containing protein 1 | Cisd1 | 22.33210564 | 22.53924179 | 22.208601 | 22.76502609 | 22.73892403 | 22.87320137 |
| **+** | 1.320425572 | -0.429657618 | Q9D8V0;A3KGR9;Q6PGJ8 | Minor histocompatibility antigen H13 | Hm13;H13 | 24.27781677 | 24.17508507 | 23.8490963 | 24.37391472 | 24.57421303 | 24.64284325 |
| **+** | 2.102153672 | -0.429618835 | Q61549;A0A3B2WB94 | EGF-like module-containing mucin-like hormone receptor-like 1 | Emr1 | 23.87164116 | 24.00829887 | 23.74396324 | 24.38185692 | 24.23644447 | 24.29445839 |
| **+** | 3.89145705 | -0.425296148 | O89086;Q8BG13;S4R2M6 | RNA-binding protein 3 | Rbm3 | 25.52608681 | 25.59052277 | 25.58457184 | 25.96298599 | 25.98185349 | 26.03223038 |
| **+** | 3.677921315 | -0.425133387 | Q3TQP6;P06801 | Malic enzyme;NADP-dependent malic enzyme | Me1 | 22.76233101 | 22.68699074 | 22.70351791 | 23.1319313 | 23.10795593 | 23.18835258 |
| **+** | 1.268229133 | -0.41987737 | D3YU17;Q8VCM8 | Nicalin | Ncln | 22.81262589 | 22.56725311 | 22.34174728 | 23.09529305 | 22.84712791 | 23.03883743 |
| **+** | 1.302069256 | -0.419050217 | Q6P5E6;A2A9W5 | ADP-ribosylation factor-binding protein GGA2 | Gga2 | 21.85707474 | 22.00261116 | 22.26135254 | 22.38474464 | 22.64544106 | 22.34800339 |
| **+** | 1.728931884 | -0.415949504 | Q9R0E1;F6W3Q8 | Procollagen-lysine,2-oxoglutarate 5-dioxygenase 3 | Plod3 | 21.98374176 | 22.30632973 | 22.179739 | 22.52458763 | 22.51111031 | 22.68196106 |
| **+** | 1.736713476 | -0.415887833 | D3Z0B9;A0A1B0GSU0;Q571I9 | Aldehyde dehydrogenase family 16 member A1 | Aldh16a1 | 23.56453514 | 23.52516174 | 23.67454529 | 24.04851532 | 24.14771271 | 23.81567764 |
| **+** | 2.107285554 | -0.415550232 | Q8CBB6;Q8CGP2;Q8CGP1;Q6ZWY9;Q64525;Q64478;Q64475;P10854;P10853;Q9D2U9;Q8CGP0;Q64524;P70696 | Histone H2B;Histone H2B type 1-P;Histone H2B type 1-K;Histone H2B type 1-C/E/G;Histone H2B type 2-B;Histone H2B type 1-H;Histone H2B type 1-B;Histone H2B type 1-M;Histone H2B type 1-F/J/L;Histone H2B type 3-A;Histone H2B type 3-B;Histone H2B type 2-E;Histone H2B type 1-A | Hist1h2br;Hist1h2bp;Hist1h2bk;Hist1h2bc;Hist2h2bb;Hist1h2bh;Hist1h2bb;Hist1h2bm;Hist1h2bf;Hist3h2ba;Hist3h2bb;Hist2h2be;Hist1h2ba | 30.41820335 | 30.29548454 | 30.18588829 | 30.66593933 | 30.81680679 | 30.66348076 |
| **+** | 2.715579549 | -0.414017359 | Q9R0P9 | Ubiquitin carboxyl-terminal hydrolase isozyme L1 | Uchl1 | 27.0812149 | 27.19441605 | 27.00945282 | 27.54450989 | 27.50370026 | 27.4789257 |
| **+** | 1.323433558 | -0.413236618 | Q8CFZ0;G3UYP0;P63280 | SUMO-conjugating enzyme UBC9 | Ube2i | 23.47494125 | 23.84718513 | 23.50748634 | 24.09498596 | 23.85405159 | 24.12028503 |
| **+** | 1.718413513 | -0.409488678 | Q61333;D3Z4L9;G3XA62;G3UWJ7;G3UXC6 | Tumor necrosis factor alpha-induced protein 2 | Tnfaip2 | 22.20871925 | 21.95840454 | 22.27073479 | 22.51224518 | 22.49882126 | 22.65525818 |
| **+** | 2.165614584 | -0.406694412 | Q61753;F6ZSB7 | D-3-phosphoglycerate dehydrogenase | Phgdh | 23.53124428 | 23.34177399 | 23.54710197 | 23.85775757 | 23.96481514 | 23.81763077 |
| **+** | 2.142068872 | -0.401820501 | P49138;A0A087WSN7 | MAP kinase-activated protein kinase 2 | Mapkapk2 | 22.86419678 | 22.76007843 | 22.88929749 | 23.26913643 | 23.34163857 | 23.1082592 |
| **+** | 1.314163763 | -0.400488536 | Q9JK81;F8WGG3;F7A3N3 | UPF0160 protein MYG1, mitochondrial | Myg1 | 22.5107975 | 22.72346687 | 22.57107925 | 23.08302116 | 23.17214203 | 22.75164604 |
| **+** | 1.400896729 | -0.400463104 | A2A5V3;A2A5V2;P55194;S4R2D3;A0A2R8W6I7;S4R2T6;A2A5V1 | SH3 domain-binding protein 1 | Sh3bp1 | 22.87594032 | 22.73661423 | 22.7720108 | 23.07095528 | 23.44833946 | 23.06665993 |
| **+** | 3.617393665 | -0.399136225 | Q9CR57;A0A1L1SUF6 | 60S ribosomal protein L14 | Rpl14 | 26.8636055 | 26.8894825 | 26.83171082 | 27.22398186 | 27.24381638 | 27.31440926 |
| **+** | 1.472512938 | -0.396735509 | E9QAS4;E9QAS5;Q6PDQ2;F6WR45 | Chromodomain-helicase-DNA-binding protein 4 | Chd4 | 21.460289 | 21.6182251 | 21.79066277 | 22.12965393 | 22.06723404 | 21.86249542 |
| **+** | 4.030027495 | -0.395814896 | Q64337;D3YZJ1;F6VD69 | Sequestosome-1 | Sqstm1 | 25.65365219 | 25.66641426 | 25.68960571 | 26.05957413 | 26.02981186 | 26.10773087 |
| **+** | 2.324627622 | -0.392983754 | P47713;Q9DBX5;A0A087WPN8 | Cytosolic phospholipase A2;Phospholipase A2;Lysophospholipase | Pla2g4a | 22.53746605 | 22.54498291 | 22.4921608 | 23.04253387 | 22.81221581 | 22.89881134 |
| **+** | 1.098895328 | -0.391866048 | O35900;O35901 | U6 snRNA-associated Sm-like protein LSm2 | Lsm2 | 22.36383247 | 22.82607651 | 22.71229935 | 22.90749168 | 22.95939445 | 23.21092033 |
| **+** | 2.384587415 | -0.391762416 | P63037;B1AXY1;B1AXY0;B1AXX9 | DnaJ homolog subfamily A member 1 | Dnaja1 | 23.65731049 | 23.56290627 | 23.6025219 | 23.91314697 | 24.11592865 | 23.96895027 |
| **+** | 2.908636196 | -0.383782069 | O09172;H3BJA3;A0A0G2JDI4;F6VNW5 | Glutamate--cysteine ligase regulatory subunit | Gclm | 25.94942284 | 26.03814697 | 25.90341187 | 26.39076996 | 26.34935379 | 26.30220413 |
| **+** | 2.146995932 | -0.382455826 | Q64281;A0A1W2P6F1;A0A1W2P7X1 | Leukocyte immunoglobulin-like receptor subfamily B member 4 | Lilrb4 | 25.26094246 | 25.30459023 | 25.16257858 | 25.50345802 | 25.71187782 | 25.6601429 |
| **+** | 1.546285906 | -0.375680923 | Q3U2S8;D3Z019;D3YZ46 | Voltage-gated hydrogen channel 1 | Hvcn1 | 23.84507942 | 23.81899643 | 24.0444355 | 24.10781097 | 24.34055138 | 24.38719177 |
| **+** | 1.727167132 | -0.374194463 | Q4LDD4;E9PUB0;D3YWW7 | Arf-GAP with Rho-GAP domain, ANK repeat and PH domain-containing protein 1 | Arap1 | 23.0750103 | 22.99688148 | 23.04485321 | 23.38784981 | 23.5892086 | 23.26226997 |
| **+** | 1.690982769 | -0.369458516 | A0A571BEI2;Q6PIU9 | Uncharacterized protein FLJ45252 homolog |  | 22.29117393 | 22.21397781 | 22.22743797 | 22.42575455 | 22.67128754 | 22.74392319 |
| **+** | 2.615354859 | -0.368824641 | Q9D1Q6 | Endoplasmic reticulum resident protein 44 | Erp44 | 23.89550591 | 23.97333717 | 24.04101181 | 24.2736969 | 24.35366631 | 24.38896561 |
| **+** | 1.346903979 | -0.368141492 | Q61074;A0A0J9YVG0;A0A0J9YV96;A0A0J9YUB0 | Protein phosphatase 1G | Ppm1g | 22.82150841 | 22.66914749 | 22.57507896 | 23.1382122 | NaN | 22.97522736 |
| **+** | 1.852316203 | -0.368095398 | P70362;E9Q475;E9Q646;E9Q0Y6 | Ubiquitin fusion degradation protein 1 homolog | Ufd1l | 23.03716278 | 22.9755249 | 23.23796272 | 23.38942528 | 23.52348709 | 23.44202423 |
| **+** | 2.089172771 | -0.366650899 | P21956 | Lactadherin | Mfge8 | 25.15120125 | 25.12699509 | 25.32563591 | 25.6025219 | 25.48526764 | 25.61599541 |
| **+** | 2.510361323 | -0.36607933 | P97372;G3X9V0;E0CZ90 | Proteasome activator complex subunit 2 | Psme2 | 24.7644577 | 24.78461647 | 24.66389275 | 25.02415657 | 25.17401886 | 25.11302948 |
| **+** | 4.081221737 | -0.365089417 | P62830;A2A6F8 | 60S ribosomal protein L23 | Rpl23 | 26.36053085 | 26.35605431 | 26.3278141 | 26.72961998 | 26.67357445 | 26.73647308 |
| **+** | 1.674988258 | -0.362742106 | Q8BJW6;D3YZZ6;D6RFN2;D6RGA6 | Eukaryotic translation initiation factor 2A;Eukaryotic translation initiation factor 2A, N-terminally processed | Eif2a | 23.01650047 | 23.07903862 | 23.19368553 | 23.47951126 | 23.30507851 | 23.59286118 |
| **+** | 2.964654218 | -0.361306508 | Q91X52;A2AC16 | L-xylulose reductase | Dcxr | 22.64805412 | 22.74962234 | 22.6911087 | 23.10298347 | 23.07182312 | 22.9978981 |
| **+** | 2.170318546 | -0.360768 | Q61152 | Tyrosine-protein phosphatase non-receptor type 18 | Ptpn18 | 22.99674416 | 23.10610199 | 23.20200729 | 23.38798141 | 23.50554848 | 23.49362755 |
| **+** | 3.239566756 | -0.358343124 | Q9ESP1 | Stromal cell-derived factor 2-like protein 1 | Sdf2l1 | 24.21896744 | 24.20851135 | 24.29747009 | 24.58697701 | 24.56894684 | 24.64405441 |
| **+** | 2.80694789 | -0.357174555 | P62806 | Histone H4 | Hist1h4a | 29.90440559 | 29.81050873 | 29.81676102 | 30.27086258 | 30.17664528 | 30.15569115 |
| **+** | 2.012611058 | -0.356894175 | B7ZNP3;Q3V4B5 | COMM domain-containing protein 6 | Commd6 | 23.16109467 | 23.3566246 | 23.41325569 | 23.64998436 | 23.67658997 | 23.67508316 |
| **+** | 4.442600154 | -0.354093552 | P63087;A0A0G2JGC1;A0A0G2JFF1 | Serine/threonine-protein phosphatase PP1-gamma catalytic subunit | Ppp1cc | 22.97917747 | 22.97172546 | 23.0249424 | 23.33469963 | 23.35353279 | 23.34989357 |
| **+** | 1.506002653 | -0.35028712 | Q9D554 | Splicing factor 3A subunit 3 | Sf3a3 | 22.35847664 | 22.02695274 | 22.207201 | 22.45181465 | 22.57991982 | 22.61175728 |
| **+** | 1.604875457 | -0.349486033 | Q9Z130;D3YTQ3;F6VQH5 | Heterogeneous nuclear ribonucleoprotein D-like | Hnrnpdl | 22.24138451 | 22.25037766 | 22.05645943 | 22.55592728 | NaN | 22.50852585 |
| **+** | 2.066519647 | -0.34646225 | P67984 | 60S ribosomal protein L22 | Rpl22 | 26.30817223 | 26.54217911 | 26.37549019 | 26.78768349 | 26.72299194 | 26.75455284 |
| **+** | 3.043877099 | -0.345671972 | Q9WVK4 | EH domain-containing protein 1 | Ehd1 | 25.19242287 | 25.1331234 | 25.17043114 | 25.44262505 | 25.55867958 | 25.53168869 |
| **+** | 2.710614008 | -0.345199585 | P08905 | Lysozyme C-2 | Lyz2 | 30.27941132 | 30.25146675 | 30.30083847 | 30.58324814 | 30.5707283 | 30.71333885 |
| **+** | 1.199547352 | -0.344722748 | Q3TBU7;Q3U2K8;Q80WC7 | Arf-GAP domain and FG repeat-containing protein 2 | Agfg2 | 22.08621597 | 22.34548759 | 22.41884995 | 22.71824837 | 22.71783066 | 22.44864273 |
| **+** | 2.165279079 | -0.338663737 | A0A0N4SVU1;P28665;P28666 | Murinoglobulin-1;Murinoglobulin-2 | Mug1;Mug2 | 29.91868973 | 29.70472336 | 29.84607315 | 30.17462349 | 30.1887207 | 30.12213326 |
| **+** | 1.220563702 | -0.337366104 | P05213;A0A2R8VHF3 | Tubulin alpha-1B chain | Tuba1b | 26.62407875 | 26.23169136 | 26.44015503 | 26.89388657 | 26.72741318 | 26.68672371 |
| **+** | 1.749805341 | -0.332050323 | Q9CPQ8 | ATP synthase subunit g, mitochondrial | Atp5l | 25.13707924 | 24.98819542 | 24.88702011 | 25.42399788 | 25.31021881 | 25.27422905 |
| **+** | 4.185913546 | -0.323794683 | O88844;A0A087WPT4;A0A087WRS9;D3YVY3;A0A087WRM4 | Isocitrate dehydrogenase [NADP] cytoplasmic | Idh1 | 26.15805817 | 26.18288994 | 26.13322258 | 26.45949936 | 26.50055885 | 26.48549652 |
| **+** | 2.271369736 | -0.323548635 | Q9DBS1 | Transmembrane protein 43 | Tmem43 | 24.56389427 | 24.62566948 | 24.52282906 | 24.81063652 | 24.88646317 | 24.98593903 |
| **+** | 1.341856319 | -0.322631836 | Q9JIK5 | Nucleolar RNA helicase 2 | Ddx21 | 22.60993385 | 22.34670448 | 22.55102921 | 22.78458595 | 22.97821617 | 22.71276093 |
| **+** | 1.245862173 | -0.321582794 | E9Q4Q2;D3YZC9;D3YZD0;Q64213;D3YVH4;F8WHF9;D6RDB7 | Splicing factor 1 | Sf1 | 23.23361015 | 23.00540543 | 22.97964859 | 23.25781822 | 23.36024666 | 23.56534767 |
| **+** | 2.066800249 | -0.320550919 | P19783;M0QWX7;D6RG40 | Cytochrome c oxidase subunit 4 isoform 1, mitochondrial | Cox4i1 | 25.45763779 | 25.38840675 | 25.47082138 | 25.64074898 | 25.79101563 | 25.84675407 |
| **+** | 1.24288981 | -0.319434484 | Q62086 | Serum paraoxonase/arylesterase 2 | Pon2 | 22.66968727 | 22.29170799 | 22.4930172 | 22.75741577 | 22.90688705 | 22.74841309 |
| **+** | 1.918126989 | -0.311896642 | O88544;F6QTS1;D3YV99;D3Z1R9;D6RG47 | COP9 signalosome complex subunit 4 | Cops4 | 25.38409805 | 25.33698273 | 25.43240166 | 25.58029366 | 25.80899429 | 25.69988441 |
| **+** | 2.42405845 | -0.311807632 | B1ARA3;P61255;B1ARA5 | 60S ribosomal protein L26 | Rpl26 | 26.08602142 | 25.97039986 | 26.12277603 | 26.34612656 | 26.41833115 | 26.35016251 |
| **+** | 1.861793341 | -0.311440786 | P68510 | 14-3-3 protein eta | Ywhah | 26.89966011 | 27.13799667 | 27.08182335 | 27.38442802 | 27.3482399 | 27.32113457 |
| **+** | 2.272334317 | -0.311294556 | Q9WVE8;A0A2R8W6S4;A0A2R8W750;Q3UP40;A0A338P6P7 | Protein kinase C and casein kinase substrate in neurons protein 2 | Pacsin2 | 23.28813553 | 23.42687988 | 23.27511787 | 23.65709114 | 23.58485794 | 23.68206787 |
| **+** | 3.734050539 | -0.310686747 | Q542I8;P11835;M0QWA7;D3YYP8;D3Z1S4;M0QWJ6 | Integrin beta;Integrin beta-2 | Itgb2 | 26.07691956 | 26.12577438 | 26.08778381 | 26.38251495 | 26.39744568 | 26.44257736 |
| **+** | 2.238449677 | -0.309689204 | Q8BMS9;A2APB1;A2AVI0 | Ras association domain-containing protein 2 | Rassf2 | 23.16098785 | 23.2428093 | 23.07788086 | 23.46973419 | 23.52659416 | 23.41441727 |
| **+** | 2.16926656 | -0.309020996 | Q5EBP8;P49312 | Heterogeneous nuclear ribonucleoprotein A1;Heterogeneous nuclear ribonucleoprotein A1, N-terminally processed | Hnrnpa1 | 25.14262009 | 25.04322624 | 25.15452766 | 25.40408897 | 25.51436615 | 25.34898186 |
| **+** | 1.284300444 | -0.295886993 | Q91YI4;Q5F2D9 | Beta-arrestin-2 | Arrb2 | 23.03733063 | 23.03219223 | 23.01415253 | 23.10827446 | 23.43720627 | 23.42585564 |
| **+** | 5.480887073 | -0.295593262 | P61027;Q9DD03;D3YUS4 | Ras-related protein Rab-10 | Rab10 | 25.24430656 | 25.25237846 | 25.26004601 | 25.56086731 | 25.53892899 | 25.54371452 |
| **+** | 2.774242037 | -0.294006983 | P62281;A0A1B0GRR3;A0A1B0GSE8 | 40S ribosomal protein S11 | Rps11 | 26.23875999 | 26.23405266 | 26.23578835 | 26.57247925 | 26.56608963 | 26.45205307 |
| **+** | 2.164863383 | -0.2890625 | P30935 | Somatostatin receptor type 3 | Sstr3 | 23.62444115 | 23.49228287 | 23.51448631 | 23.75674438 | 23.88427544 | 23.85737801 |
| **+** | 2.645157984 | -0.288251877 | P99024;A0A1D5RM76 | Tubulin beta-5 chain | Tubb5 | 28.3867321 | 28.33398438 | 28.42802238 | 28.61171722 | 28.71829605 | 28.68348122 |
| **+** | 2.100039654 | -0.287303289 | F8WJK8;Q99L47;E9Q1V0;E9Q1X9 | Hsc70-interacting protein | St13 | 24.9185257 | 24.81210709 | 25.0069294 | 25.21704483 | 25.16882706 | 25.21360016 |
| **+** | 1.449059864 | -0.28703626 | P09581 | Macrophage colony-stimulating factor 1 receptor | Csf1r | 23.72525597 | 23.42149734 | 23.59434319 | 23.82221031 | 23.91570282 | 23.86429214 |
| **+** | 2.435919091 | -0.285772324 | P47791 | Glutathione reductase, mitochondrial | Gsr | 24.49679947 | 24.39512634 | 24.55268288 | 24.76116371 | 24.78401756 | 24.75674438 |
| **+** | 2.01979062 | -0.285727183 | Q61510;Q5SU71;Q5SU72 | E3 ubiquitin/ISG15 ligase TRIM25 | Trim25 | 22.60584831 | 22.68978691 | 22.70484734 | 22.85775757 | 22.959095 | 23.04081154 |
| **+** | 2.169423793 | -0.283732732 | O35345;Q4FJZ2 | Importin subunit alpha-7;Importin subunit alpha | Kpna6 | 22.35183525 | 22.26665115 | 22.41454506 | 22.56908607 | 22.68927383 | 22.62586975 |
| **+** | 3.191475847 | -0.281242371 | A0A0B4J1M6;E9PUV2;E9PVR4 | Fc receptor, IgG, low affinity III | Fcgr3 | 22.86079216 | 22.92041588 | 22.83159447 | 23.16475105 | 23.12647438 | 23.16530418 |
| **+** | 1.717461518 | -0.280426661 | Q91V12;E9PYH2;A0A0E2W844 | Cytosolic acyl coenzyme A thioester hydrolase | Acot7 | 24.69242859 | 24.75363541 | 24.76931 | 24.92038918 | 24.98185349 | 25.15441132 |
| **+** | 2.142964488 | -0.279163996 | P63328;E9Q6P2;G3X8U7;E0CZ78;P48453 | Serine/threonine-protein phosphatase 2B catalytic subunit alpha isoform | Ppp3ca | 23.63677216 | 23.58003426 | 23.71565437 | 23.88072968 | 24.00066948 | 23.88855362 |
| **+** | 2.257336549 | -0.273820241 | Q8BP67 | 60S ribosomal protein L24 | Rpl24 | 26.16006088 | 26.13345718 | 26.06874466 | 26.47730446 | 26.36906242 | 26.33735657 |
| **+** | 2.63195547 | -0.270801544 | P08113;F7C312 | Endoplasmin | Hsp90b1 | 26.76395226 | 26.66898537 | 26.69776535 | 26.93910027 | 27.03286171 | 26.97114563 |
| **+** | 2.243422833 | -0.268643061 | P62242 | 40S ribosomal protein S8 | Rps8 | 26.24530411 | 26.3037014 | 26.20778656 | 26.54908752 | 26.57393837 | 26.43969536 |
| **+** | 2.502655887 | -0.267455419 | P70699;F6R5R5;F6VEG4;A2AFL3;A2AFL5 | Lysosomal alpha-glucosidase | Gaa | 23.09474564 | 22.97237396 | 22.99681282 | 23.32731628 | 23.27198792 | 23.26699448 |
| **+** | 2.183670073 | -0.262606303 | A0A1L1SQA8;P62852 | 40S ribosomal protein S25 | Rps25 | 26.18237877 | 26.26844025 | 26.20698547 | 26.53701782 | 26.51285934 | 26.39574623 |
| **+** | 2.960350825 | -0.261334101 | P01027;H3BL60 | Complement C3;Complement C3 beta chain;C3-beta-c;Complement C3 alpha chain;C3a anaphylatoxin;Acylation stimulating protein;Complement C3b alpha chain;Complement C3c alpha chain fragment 1;Complement C3dg fragment;Complement C3g fragment;Complement C3d fragment;Complement C3f fragment;Complement C3c alpha chain fragment 2 | C3 | 25.61862946 | 25.70186996 | 25.60379601 | 25.90403175 | 25.89198685 | 25.91227913 |
| **+** | 1.707182322 | -0.258822123 | F6VRP8 | Lectin, galactoside-binding, soluble, 3 binding protein | Lgals3bp | 23.79728889 | 24.03456306 | 23.92352104 | 24.18510437 | 24.17638016 | 24.17035484 |
| **+** | 3.490370298 | -0.257600149 | Q3U7R1;A0A1W2P784 | Extended synaptotagmin-1 | Esyt1 | 25.85178947 | 25.81311035 | 25.87258148 | 26.07549286 | 26.11874199 | 26.11604691 |
| **+** | 3.887305229 | -0.253672282 | Q9WUK2 | Eukaryotic translation initiation factor 4H | Eif4h | 24.92759514 | 24.93170357 | 24.90265274 | 25.19798088 | 25.14685822 | 25.1781292 |
| **+** | 1.688454965 | -0.253549576 | P62754 | 40S ribosomal protein S6 | Rps6 | 26.29205513 | 26.491045 | 26.32354164 | 26.61540604 | 26.67559433 | 26.57629013 |
| **+** | 2.104617837 | -0.253173828 | E9PVC5;E9PVC6;E9Q9E1;Q6NZJ6;A0A0J9YUS5;E9Q770;A0A0J9YVC1;A0A0J9YUC1;A0A0J9YUL1;D3Z439;A0A0J9YU44;A0A0J9YTV0;D3YWY7;D3YWM1 | Eukaryotic translation initiation factor 4 gamma 1 | Eif4g1 | 24.0765934 | 24.10645485 | 24.18131638 | 24.42136765 | 24.29333687 | 24.40918159 |
| **+** | 1.70638003 | -0.251876195 | O08807;B1AZS9 | Peroxiredoxin-4 | Prdx4 | 31.48754692 | 31.39953041 | 31.37859917 | 31.69295311 | 31.5651989 | 31.76315308 |
| **+** | 1.86757541 | -0.250562668 | O35379;D3YZY1;E9Q1I5 | Multidrug resistance-associated protein 1 | Abcc1 | 22.42203522 | 22.41061783 | 22.56792641 | 22.77926445 | 22.67908478 | 22.69391823 |
| **+** | 3.156565279 | -0.248958588 | B1AQR8;G3X9T7;O08573 | Galectin;Galectin-9 | Lgals9 | 24.47667122 | 24.41164017 | 24.39813042 | 24.65807343 | 24.6925869 | 24.68265724 |
| **+** | 1.877709002 | -0.246232033 | D5MCW4;Q9CQ89 | Protein CutA | Cuta | NaN | 22.56971169 | 22.56980515 | 22.84462166 | NaN | 22.78735924 |
| **+** | 1.665649805 | -0.239758809 | Q07113 | Cation-independent mannose-6-phosphate receptor | Igf2r | 24.51207542 | 24.60195541 | 24.61042976 | 24.71413422 | 24.91319275 | 24.81641006 |
| **+** | 4.156506126 | -0.23698616 | P27773;F6Q404 | Protein disulfide-isomerase A3 | Pdia3 | 27.06866264 | 27.051754 | 27.06229401 | 27.30530357 | 27.31579018 | 27.27257538 |
| **+** | 3.266226935 | -0.235776901 | A3KGU5;A3KGU7;E9Q447;A3KGU9;P16546;A3KGU4 | Spectrin alpha chain, non-erythrocytic 1 | Sptan1 | 22.64559364 | 22.6408596 | 22.69578743 | 22.90839005 | 22.91517258 | 22.86600876 |
| **+** | 2.815612931 | -0.234752973 | Q5SV41;P53808 | Phosphatidylcholine transfer protein | Pctp | 23.41351318 | 23.45400047 | 23.42943573 | 23.64768219 | 23.68645668 | NaN |
| **+** | 2.181684445 | -0.234027227 | E9Q604;G5E8F1;E9Q5K8;Q3U1U4;P05555;A0A0R4J1B4;D6RJ73 | Integrin alpha-M | Itgam | 26.09490585 | 26.01994324 | 25.95688629 | 26.24265289 | 26.29965591 | 26.23150826 |
| **+** | 3.058879958 | -0.233678818 | Q9WVA4;A0A0A6YXG6;Q9R1Q8 | Transgelin-2 | Tagln2 | 27.48968315 | 27.43698311 | 27.49526024 | 27.68170547 | 27.69783211 | 27.74342537 |
| **+** | 1.729726439 | -0.23070399 | Q9CYN2;A0A140LJ01;A0A140LHG8;A0A140LHR3;A0A140LJG6;A0A140LIK0;A0A140LHW5 | Signal peptidase complex subunit 2 | Spcs2 | 24.5909214 | 24.56993294 | 24.46836662 | 24.77364159 | 24.85505104 | 24.6926403 |
| **+** | 3.788452684 | -0.229891459 | Q3UPH1 | Protein PRRC1 | Prrc1 | 23.23007965 | 23.22247887 | 23.2239666 | 23.42892456 | 23.45136261 | 23.48591232 |
| **+** | 2.528162451 | -0.229182561 | P14901 | Heme oxygenase 1 | Hmox1 | 27.3627739 | 27.33039856 | 27.41788101 | 27.58161545 | 27.64828491 | 27.56870079 |
| **+** | 2.417953591 | -0.223079046 | Q9ET22 | Dipeptidyl peptidase 2 | Dpp7 | 24.25364113 | 24.29579163 | 24.3658638 | 24.54910278 | 24.49387169 | 24.54155922 |
| **+** | 2.03548483 | -0.220649083 | O70145;A0A087WPH0 | Neutrophil cytosol factor 2 | Ncf2 | 25.04435158 | 25.17150116 | 25.09273148 | 25.3788166 | 25.28316116 | 25.3085537 |
| **+** | 3.064057723 | -0.217227936 | P24668 | Cation-dependent mannose-6-phosphate receptor | M6pr | 25.57885361 | 25.60073662 | 25.52984428 | 25.76364899 | 25.80506134 | 25.79240799 |
| **+** | 2.074344626 | -0.214237213 | Q920E5;A0A0G2JEA5;A0A0G2JDJ5;A0A0G2JEB3 | Farnesyl pyrophosphate synthase | Fdps | 23.99023056 | 23.93939209 | 24.05191994 | 24.15313721 | 24.21437836 | 24.25673866 |
| **+** | 1.984989978 | -0.209679286 | Q9JM76;H7BWZ3;A0A0G2JFK7;D3Z2F7;D3Z2F8 | Actin-related protein 2/3 complex subunit 3 | Arpc3 | 26.85851669 | 26.70028305 | 26.79054451 | 26.98489761 | 26.99690437 | 26.99658012 |
| **+** | 2.091437393 | -0.206023534 | Q8BQ30;A0A087WRI9 | Phostensin | Ppp1r18 | 23.25954247 | 23.25196648 | 23.17894936 | 23.49789619 | 23.42739105 | 23.38324165 |
| **+** | 2.086756585 | -0.203353246 | P62264;D3YVF4;D3Z7I1;A0A494BAS4 | 40S ribosomal protein S14 | Rps14 | 26.48300743 | 26.5839119 | 26.47832489 | 26.75633621 | 26.67532539 | 26.72364235 |
| **+** | 2.967166313 | -0.190039317 | Q8K2B3;A0A1Y7VJ55 | Succinate dehydrogenase [ubiquinone] flavoprotein subunit, mitochondrial | Sdha | 23.88632393 | 23.9221611 | 23.93544006 | 24.0705471 | 24.12075996 | 24.12273598 |
| **+** | 2.803177902 | -0.179339727 | P26041 | Moesin | Msn | 28.23817635 | 28.24318123 | 28.304739 | 28.45939827 | 28.42658424 | 28.43813324 |
| **+** | 3.35488629 | -0.168842316 | P17182;Q6PHC1;B0QZL1;B1ARR7;P21550;P17183;B1ARR6;A0A0N4SUX5;D3Z6E4;D3Z2S4;J3QPZ9;Q5SX59;A0A0N4SUI6;D3YVD3;Q5SX60;Q5SX61;A0A0N4SUW8 | Alpha-enolase | Eno1 | 28.9949646 | 28.96554184 | 28.9464817 | 29.1476841 | 29.12364006 | 29.14219093 |
| **+** | 4.285055946 | -0.163399378 | Q8VDD5;A0A2R8VKI5;Q5SV64;Q3UH59;Q61879;F2Z494;A0A2R8W6V7;Q8BXF2 | Myosin-9 | Myh9 | 26.95958519 | 26.94848824 | 26.93145561 | 27.11296844 | 27.10289574 | 27.11386299 |
| **+** | 3.203076495 | 0.168276469 | P28867;Q1MX43;Q1MX42;Q1MX41;Q1MX40;REV__E9Q9T6;REV__Q6ZPL9;F8WGT6;Q02111 | Protein kinase C delta type;Protein kinase C delta type regulatory subunit;Protein kinase C delta type catalytic subunit;Protein kinase C | Prkcd | 24.15259552 | 24.12036514 | 24.18025589 | 23.98320198 | 23.9825058 | 23.98267937 |
| **+** | 3.596388708 | 0.169796626 | Q8BFR5;A0A0U1RNQ6;A0A0U1RPC4 | Elongation factor Tu, mitochondrial | Tufm | 25.62396812 | 25.60907745 | 25.65572739 | 25.4573555 | 25.46248245 | 25.45954514 |
| **+** | 3.659817748 | 0.17277654 | Q9JKF1;A0A0U1RNG5;A0A0U1RPU3;Q3UQP1;A0A0U1RPI2 | Ras GTPase-activating-like protein IQGAP1 | Iqgap1 | 26.78119278 | 26.80771637 | 26.78019142 | 26.60540771 | 26.60823059 | 26.63713264 |
| **+** | 2.574849396 | 0.190400441 | Q62087;H3BL07 | Serum paraoxonase/lactonase 3 | Pon3 | 25.16691208 | 25.08375168 | 25.09345627 | 24.9397049 | 24.90187073 | 24.93134308 |
| **+** | 3.13497009 | 0.199319204 | Q91Z25;Q9WV32;F6VVE6;F6THG2;D3Z6S0 | Actin-related protein 2/3 complex subunit 1B | Arpc1b | 27.09275055 | 27.03433228 | 27.03967476 | 26.83978081 | 26.87549019 | 26.85352898 |
| **+** | 3.006705391 | 0.200621923 | P26443;F7CFA5 | Glutamate dehydrogenase 1, mitochondrial | Glud1 | 26.82420158 | 26.89700699 | 26.84995461 | 26.645401 | 26.64924431 | 26.6746521 |
| **+** | 3.643300876 | 0.20163091 | Q99KC8;F6TIL5;D3Z671;D3Z518 | von Willebrand factor A domain-containing protein 5A | Vwa5a | 25.72254944 | 25.71630859 | 25.71874046 | 25.52067375 | 25.48864174 | 25.54339027 |
| **+** | 2.167883421 | 0.203400294 | P62962;Q5SX49;J3QMC2 | Profilin-1;Profilin | Pfn1 | 28.78547096 | 28.78799438 | 28.78497124 | 28.66180038 | 28.54620552 | 28.5402298 |
| **+** | 2.089621171 | 0.204797745 | Q99LB4;D3YTL5;P24452;D3YZN3;D3YU77;D3Z4K5;D3Z014 | Macrophage-capping protein | Capg | 29.14671326 | 29.13985252 | 29.08268738 | 28.95127106 | 28.84502602 | 28.95856285 |
| **+** | 2.239989587 | 0.206998189 | P45376;D3YVJ7 | Aldose reductase | Akr1b1;Akr1b3 | 26.65362549 | 26.58233261 | 26.69882584 | 26.43561745 | 26.4077549 | 26.47041702 |
| **+** | 2.210729214 | 0.207935333 | Q9R1P0;E9PW69;E9Q0X0 | Proteasome subunit alpha type-4;Proteasome subunit alpha type | Psma4 | 25.55978775 | 25.68645668 | 25.64713287 | 25.44483757 | 25.40278816 | 25.42194557 |
| **+** | 2.956633164 | 0.211926142 | P08249;A0A0G2JF23;A0A0G2JGY4 | Malate dehydrogenase, mitochondrial | Mdh2 | 27.39631844 | 27.37625313 | 27.32311249 | 27.12858772 | 27.16057968 | 27.17073822 |
| **+** | 2.243696871 | 0.213370641 | Q91YQ5;A0A0N4SUJ8 | Dolichyl-diphosphooligosaccharide--protein glycosyltransferase subunit 1 | Rpn1 | 25.73665428 | 25.76369858 | 25.80328941 | 25.57718468 | 25.59957504 | 25.48677063 |
| **+** | 2.962596198 | 0.21522522 | P14869;S4R1N1;D3YVM5 | 60S acidic ribosomal protein P0 | Rplp0 | 27.47506523 | 27.51983261 | 27.55808067 | 27.31570435 | 27.28568649 | 27.30591202 |
| **+** | 2.115955153 | 0.216796239 | D3Z041;P41216;D3Z457;D3YVF6;A0A1B0GRS8;F6WNZ2 | Long-chain-fatty-acid--CoA ligase 1 | Acsl1 | 23.69890594 | 23.76182365 | 23.65654564 | 23.5400238 | 23.43262482 | 23.4942379 |
| **+** | 2.883314412 | 0.217392604 | P62814;Q91YH6;A0A0U1RNU9 | V-type proton ATPase subunit B, brain isoform | Atp6v1b2 | 27.23908806 | 27.221035 | 27.19713593 | 27.04603958 | 26.99636459 | 26.962677 |
| **+** | 3.529022895 | 0.218022664 | Q9ER72;A0A140LIB6 | Cysteine--tRNA ligase, cytoplasmic | Cars | 23.65108109 | 23.67809677 | 23.64954567 | 23.45437813 | 23.40976334 | 23.46051407 |
| **+** | 3.035643097 | 0.218588511 | Q99JY9;A0A087WRA1;A0A087WQ14;A0A087WP86;A0A087WS98;Q641P0;A0A087WQ83;A0A087WPR6 | Actin-related protein 3 | Actr3 | 27.32945633 | 27.37757683 | 27.30234337 | 27.1120739 | 27.14014435 | 27.10139275 |
| **+** | 2.673552555 | 0.21948115 | P50580 | Proliferation-associated protein 2G4 | Pa2g4 | 25.95923042 | 25.95422554 | 25.9017086 | 25.67203712 | 25.75765991 | 25.72702408 |
| **+** | 4.115973013 | 0.220867157 | P51881 | ADP/ATP translocase 2;ADP/ATP translocase 2, N-terminally processed | Slc25a5 | 28.59327507 | 28.5932045 | 28.55705833 | 28.36998558 | 28.35072899 | 28.36022186 |
| **+** | 2.401931473 | 0.22149531 | Q60931;J3QMG3 | Voltage-dependent anion-selective channel protein 3 | Vdac3 | 25.31475449 | 25.21215439 | 25.27391052 | 25.08151817 | 25.04955482 | 25.00526047 |
| **+** | 1.796796719 | 0.224948883 | P17426;A0A140LIG7;A0A140LHG0;A0A140LHA6 | AP-2 complex subunit alpha-1 | Ap2a1 | 27.76509094 | 27.85108757 | 27.69391823 | 27.51178932 | 27.51269341 | 27.61076736 |
| **+** | 2.173072081 | 0.225257874 | Q9DB77;A0A140LI98 | Cytochrome b-c1 complex subunit 2, mitochondrial | Uqcrc2 | 25.24067116 | 25.20612907 | 25.32168579 | 25.05973816 | 24.97670555 | 25.05626869 |
| **+** | 1.9806455 | 0.227423986 | Q9Z1T1;A0A338P6V5;A0A571BEW4;A0A571BES1;Q9JME5 | AP-3 complex subunit beta-1 | Ap3b1 | 24.84565544 | 24.76567268 | 24.76971436 | 24.65151787 | 24.5182724 | 24.52898026 |
| **+** | 2.292718429 | 0.228595098 | P63260;B1ATY1;G3UYG0;E9Q606 | Actin, cytoplasmic 2;Actin, cytoplasmic 2, N-terminally processed | Actg1 | 28.88579369 | 28.88232803 | 28.79736328 | 28.58003426 | 28.61943436 | 28.68023109 |
| **+** | 2.310970548 | 0.229939143 | P97449;A0A0U1RNS3 | Aminopeptidase N | Anpep | 28.21664619 | 28.21094704 | 28.17202568 | 28.02078056 | 27.89481163 | 27.99420929 |
| **+** | 2.654239074 | 0.230019887 | Q9CZ30;B1AYJ9 | Obg-like ATPase 1 | Ola1 | 24.08432007 | 24.06357002 | 24.09047127 | 23.82861519 | 23.9121418 | 23.80754471 |
| **+** | 1.847257601 | 0.231614431 | P18242;F8WIR1;F6Y6L6;A0A1B0GT66 | Cathepsin D | Ctsd | 29.57958412 | 29.59832573 | 29.49085045 | 29.27008247 | 29.29028511 | 29.41354942 |
| **+** | 1.719868907 | 0.232628504 | F6UP77;F7CUP3;Q8JZV7 | Putative N-acetylglucosamine-6-phosphate deacetylase | Amdhd2 | 23.95810318 | 23.83258057 | 23.75633621 | 23.60512543 | 23.59457207 | 23.64943695 |
| **+** | 3.160013362 | 0.23573939 | Q9DBG5;A0A3B2WCW2 | Perilipin-3 | Plin3 | 25.49539757 | 25.51734161 | 25.54842567 | 25.27020645 | 25.26040459 | 25.32333565 |
| **+** | 1.994755932 | 0.236318588 | Q02819;A0A1C7CYU3;A0A1B0GR41;A0A1B0GR92;A0A1B0GS16;A0A1B0GT83 | Nucleobindin-1 | Nucb1 | 24.11481667 | 23.99860573 | 24.0771637 | 23.87483406 | 23.75103188 | 23.85576439 |
| **+** | 2.954014773 | 0.236641566 | P14152;A0A5F8MPN8;B1ATQ3 | Malate dehydrogenase, cytoplasmic | Mdh1 | 27.18139267 | 27.15122032 | 27.22416687 | 26.97092628 | 26.91170883 | 26.96422005 |
| **+** | 2.834620135 | 0.236684799 | Q61335 | B-cell receptor-associated protein 31 | Bcap31 | 26.1352787 | 26.10880852 | 26.17502785 | 25.86372375 | 25.90032768 | 25.94500923 |
| **+** | 1.981038698 | 0.236900965 | P62317 | Small nuclear ribonucleoprotein Sm D2 | Snrpd2 | 24.95544434 | 24.93737221 | 24.9223423 | 24.79743767 | 24.62243271 | 24.68458557 |
| **+** | 2.363754094 | 0.237059911 | Q9CQ60;D3Z4X1;Q8CBG6;F6X8L5 | 6-phosphogluconolactonase | Pgls | 25.08306122 | 25.13277054 | 25.14953613 | 24.92737007 | 24.81431007 | 24.91250801 |
| **+** | 2.338587031 | 0.23919042 | O70435;E0CX62;E0CZ34;F8WH02;E0CYL6 | Proteasome subunit alpha type-3 | Psma3 | 25.75595474 | 25.78464127 | 25.65608215 | 25.50899696 | 25.50691032 | 25.46319962 |
| **+** | 1.654965249 | 0.239343007 | P84084 | ADP-ribosylation factor 5 | Arf5 | 24.77334023 | 24.95575523 | 24.77936554 | 24.64267731 | 24.54710197 | 24.60065269 |
| **+** | 3.748081397 | 0.239477158 | Q99P72 | Reticulon-4 | Rtn4 | 26.97015762 | 26.97081757 | 26.9648819 | 26.6936512 | 26.74599075 | 26.74778366 |
| **+** | 2.234307071 | 0.241702398 | Q9D0R2;A0A2I3BPK8;Q8BLY2 | Threonine--tRNA ligase, cytoplasmic | Tars | 23.88352966 | 23.89476585 | 23.92940331 | 23.66644096 | 23.58394051 | 23.73221016 |
| **+** | 2.354784464 | 0.243302663 | Q9Z2I0 | LETM1 and EF-hand domain-containing protein 1, mitochondrial | Letm1 | 23.94136429 | 23.90517998 | 23.93255997 | 23.75806618 | 23.61874199 | 23.67238808 |
| **+** | 2.922295709 | 0.244720459 | Q9QYB1 | Chloride intracellular channel protein 4 | Clic4 | 24.78071785 | 24.7056694 | 24.79575539 | 24.52957726 | 24.4952755 | 24.52312851 |
| **+** | 1.854559629 | 0.245157878 | P61620;Q9CYJ6;Q9JLR1;A0A0A6YVQ7;A2ATT9 | Protein transport protein Sec61 subunit alpha isoform 1;Protein transport protein Sec61 subunit alpha isoform 2 | Sec61a1;Sec61a2 | 24.83566666 | 24.82721138 | 24.8737545 | 24.71009254 | 24.51899338 | 24.57207298 |
| **+** | 2.289467962 | 0.245922089 | O88456;A0A0R4J1C2;Q9D7J7 | Calpain small subunit 1 | Capns1 | 25.20899582 | 25.19080353 | 25.27589989 | 24.93800163 | 24.94908905 | 25.05084229 |
| **+** | 1.694666487 | 0.246708552 | P27546;A0A140T8T5;A0A0G2JFH2;E9PZ43;A0A0G2JDN7;A0A0G2JG35;Q78TF3;A0A0G2JFK3;A0A0G2JDY5;A0A0G2JFT4;A0A0G2JE57;A0A0G2JDU1 | Microtubule-associated protein 4 | Map4 | 24.39904404 | 24.50106049 | 24.44499588 | 24.21704483 | 24.09248924 | 24.29544067 |
| **+** | 2.17153876 | 0.247413635 | Q63829 | COMM domain-containing protein 3 | Commd3 | 23.04904747 | 23.08605385 | 23.0728054 | 22.86680222 | 22.87053108 | 22.72833252 |
| **+** | 1.702221017 | 0.247442881 | P45377 | Aldose reductase-related protein 2 | Akr1b8 | 25.74806595 | 25.83855629 | 25.88418198 | 25.67314339 | 25.49347496 | 25.56185722 |
| **+** | 2.05862155 | 0.24830246 | A2BGI8;Q9D868;A2BGI9 | Peptidyl-prolyl cis-trans isomerase;Peptidyl-prolyl cis-trans isomerase H | Ppih | 22.10504723 | 22.07247734 | 22.22814369 | 21.84861755 | 21.92136192 | 21.8907814 |
| **+** | 3.160300904 | 0.248539607 | P56480 | ATP synthase subunit beta, mitochondrial | Atp5b | 28.45254135 | 28.47934914 | 28.41747856 | 28.23949814 | 28.18385506 | 28.18039703 |
| **+** | 1.909381209 | 0.249452591 | Q3UZG4;P31230;A0A0G2JDW6;A0A0G2JEU9;A0A0G2JDH0 | Aminoacyl tRNA synthase complex-interacting multifunctional protein 1;Endothelial monocyte-activating polypeptide 2 | Aimp1 | 24.42905235 | 24.4616394 | 24.3558197 | 24.07005501 | 24.20433807 | 24.2237606 |
| **+** | 2.282749823 | 0.250616074 | A0A1B0GS58;Q9CQM9;A0A1B0GT04;A0A1B0GSD3 | Glutaredoxin-3 | Glrx3 | 24.50379181 | 24.48591232 | 24.42540741 | 24.28093338 | 24.23337555 | 24.14895439 |
| **+** | 1.69013904 | 0.251397451 | Q6ZQ38;D3YWC5 | Cullin-associated NEDD8-dissociated protein 1 | Cand1 | 24.20746994 | 24.18283272 | 24.02174377 | 23.82298851 | 23.89448738 | 23.94037819 |
| **+** | 2.22314164 | 0.252276738 | P84096;A0A1B0GSL4 | Rho-related GTP-binding protein RhoG | Rhog | 27.35245514 | 27.46053123 | 27.49495506 | 27.14957428 | 27.18262482 | 27.21891212 |
| **+** | 2.040029859 | 0.252827326 | Q80XN0;D3Z2Y8 | D-beta-hydroxybutyrate dehydrogenase, mitochondrial | Bdh1 | 23.30326653 | 23.36238861 | 23.19961357 | 23.0117836 | 23.08444977 | 23.01055336 |
| **+** | 1.695201901 | 0.255449295 | Q8CGK3 | Lon protease homolog, mitochondrial | Lonp1 | 23.55168533 | 23.64052773 | 23.47097588 | 23.28263092 | 23.38850594 | 23.22570419 |
| **+** | 1.535527562 | 0.257044474 | B1AU25;Q9Z0X1 | Apoptosis-inducing factor 1, mitochondrial | Aifm1 | 24.03598976 | 24.16943741 | 24.14600372 | 23.85566902 | 23.97543716 | 23.74919128 |
| **+** | 2.732525653 | 0.257776896 | P0DP28;P0DP27;P0DP26;A0A3Q4EHJ0;Q9D6P8;G3UX57;P20801 | Calmodulin-like protein 3 | Calml3 | 28.75098038 | 28.82562828 | 28.76040268 | 28.57286072 | 28.50354958 | 28.48727036 |
| **+** | 1.741780533 | 0.260916392 | A0A494BAJ6;Q9WUL7 | ADP-ribosylation factor-like protein 3 | Arl3 | 22.45983887 | 22.60104942 | 22.55599785 | 22.29600143 | NaN | 22.26008987 |
| **+** | 1.814060309 | 0.261189143 | O35075;A0A338P6T5 | Down syndrome critical region protein 3 homolog | Dscr3 | 22.73374176 | 22.71929359 | 22.83393097 | 22.60367775 | 22.42477989 | 22.47494125 |
| **+** | 1.474044327 | 0.263414383 | O35685 | Nuclear migration protein nudC | Nudc | 23.88874054 | 23.89744568 | 23.95624352 | 23.77112579 | 23.49899292 | 23.68206787 |
| **+** | 1.55025853 | 0.264485041 | Q9CPQ3;A0A2R8VHM4;A0A2R8VHJ4 | Mitochondrial import receptor subunit TOM22 homolog | Tomm22 | 23.9208889 | 23.86126518 | 24.05424118 | 23.76830101 | 23.58210373 | 23.6925354 |
| **+** | 1.811139413 | 0.265829086 | B1AVH5;Q8C0P5 | Coronin;Coronin-2A | Coro2a | 22.64216042 | 22.79614067 | 22.74347115 | 22.47704315 | 22.53487968 | 22.37236214 |
| **+** | 1.742225883 | 0.266246796 | Q91VA7;A0A668KL51;V9GXV0 | Isocitrate dehydrogenase [NAD] subunit, mitochondrial | Idh3b | 24.05573082 | 24.03481483 | 24.05796242 | 23.76638031 | 23.6738987 | 23.90948868 |
| **+** | 1.573589532 | 0.266768773 | Q9D023;A0A0A6YY89;G5E869 | Mitochondrial pyruvate carrier 2 | Mpc2;Zfp142 | 22.94574547 | 23.09585571 | 23.02961731 | 22.75857544 | 22.86821556 | 22.64412117 |
| **+** | 2.444926008 | 0.268542608 | Q99KI0;A0A2R8W744;A0A2R8VHM8;A0A2R8VJW0 | Aconitate hydratase, mitochondrial | Aco2 | 25.66979599 | 25.58832169 | 25.70065308 | 25.38732338 | 25.33408546 | 25.43173409 |
| **+** | 2.833396927 | 0.268599828 | Q61599;D3YWL7;A0A0N4SVH4 | Rho GDP-dissociation inhibitor 2 | Arhgdib | 27.77824974 | 27.72728348 | 27.71738052 | 27.42005157 | 27.51975822 | 27.47730446 |
| **+** | 2.300013848 | 0.269685109 | P31786;Q4VWZ5;M0QWU8;D3Z563 | Acyl-CoA-binding protein | Dbi | 27.65061569 | 27.66064453 | 27.68090248 | 27.32808685 | 27.48615837 | 27.36886215 |
| **+** | 1.875865785 | 0.270167669 | Q9DCW4;A0A0U1RNP5;A0A0N4SVE0;A0A0U1RNR3;A0A0U1RNK9;A0A0N4SWE9;A0A0U1RQB4 | Electron transfer flavoprotein subunit beta | Etfb | 25.09635544 | 25.02647972 | 24.9306221 | 24.75709915 | 24.81513977 | 24.67071533 |
| **+** | 2.072752181 | 0.270353317 | O88569;A0A0N4SUM2 | Heterogeneous nuclear ribonucleoproteins A2/B1 | Hnrnpa2b1 | 26.96565247 | 27.13104439 | 27.11217308 | 26.76509094 | 26.7983532 | 26.83436584 |
| **+** | 2.590547835 | 0.271736781 | Q8C129 | Leucyl-cystinyl aminopeptidase | Lnpep | 24.12376213 | 24.03766632 | 24.16752625 | 23.8475666 | 23.81107903 | 23.85509872 |
| **+** | 2.018794117 | 0.272019068 | O09159 | Lysosomal alpha-mannosidase | Man2b1 | 26.27218437 | 26.30098343 | 26.25837517 | 25.92036629 | 25.98161316 | 26.11350632 |
| **+** | 2.57171306 | 0.272633235 | Q9EPU0 | Regulator of nonsense transcripts 1 | Upf1 | 24.42790222 | 24.4913044 | 24.55162621 | 24.19708061 | 24.25832176 | 24.19753075 |
| **+** | 2.386976796 | 0.274017334 | P62245;F8WJ41;D3YVB4;D3Z712 | 40S ribosomal protein S15a | Rps15a | 26.19631195 | 26.22042465 | 26.13600349 | 25.88853073 | 25.98591805 | 25.85623932 |
| **+** | 2.050485381 | 0.27437973 | E9PZF0;Q01768 | Nucleoside diphosphate kinase;Nucleoside diphosphate kinase B | Gm20390;Nme2 | 28.8944931 | 29.05330849 | 29.01826286 | 28.77067757 | 28.71105766 | 28.66119003 |
| **+** | 1.79201606 | 0.275818507 | O09131;A0A494BAB1;A0A494B9X6;A0A494BAY2;A0A494BB82;Q8K2Q2 | Glutathione S-transferase omega-1 | Gsto1 | 25.55599213 | 25.55024719 | 25.64240265 | 25.34793472 | 25.1848774 | 25.38837433 |
| **+** | 2.844027699 | 0.276233037 | Q9WTR1 | Transient receptor potential cation channel subfamily V member 2 | Trpv2 | 25.01017761 | 24.93889809 | 24.93692398 | 24.72364235 | 24.69699669 | 24.63666153 |
| **+** | 1.398634378 | 0.277065277 | A0A0R4J078;Q8VCH8;A0A087WSK5 | UBX domain-containing protein 4 | Ubxn4 | 23.00814438 | 22.88912964 | 22.8038063 | 22.6689949 | 22.48431396 | 22.71657562 |
| **+** | 2.671505529 | 0.278375626 | D6RHA2;A0A8Z1SL50;P82343;A0A8Z1S8W2;G3XA46;A0A0E2WEF3 | N-acylglucosamine 2-epimerase | Renbp | 25.67521858 | 25.76250839 | 25.73665428 | 25.50503349 | 25.40785217 | 25.42636871 |
| **+** | 2.415528532 | 0.27859815 | P06800;S4R1M0;A0A0A6YXM4 | Receptor-type tyrosine-protein phosphatase C;Protein-tyrosine-phosphatase | Ptprc | 25.51285934 | 25.64099693 | 25.49368858 | 25.27020645 | 25.27476311 | 25.26678085 |
| **+** | 1.423840288 | 0.278735479 | Q9Z110;D3Z0B4;H3BKJ8;H3BLE8 | Delta-1-pyrroline-5-carboxylate synthase;Glutamate 5-kinase;Gamma-glutamyl phosphate reductase | Aldh18a1 | 23.85680771 | 23.99023056 | 23.87389565 | 23.47109985 | 23.67346764 | 23.74015999 |
| **+** | 2.22636565 | 0.280666351 | Q8BMS1 | Trifunctional enzyme subunit alpha, mitochondrial;Long-chain enoyl-CoA hydratase;Long chain 3-hydroxyacyl-CoA dehydrogenase | Hadha | 25.2577095 | 25.15240097 | 25.25411034 | 24.89268303 | 24.91008377 | 25.01945496 |
| **+** | 2.04356733 | 0.280972799 | P20108 | Thioredoxin-dependent peroxide reductase, mitochondrial | Prdx3 | 25.52919006 | 25.47889519 | 25.37298584 | 25.21771049 | 25.10465622 | 25.21578598 |
| **+** | 2.652930132 | 0.281257629 | P63323;F7AEH4;A0A1W2P7A1 | 40S ribosomal protein S12 | Rps12 | 26.90230751 | 26.93910027 | 26.97956467 | 26.60710144 | 26.72195053 | 26.64814758 |
| **+** | 2.085887353 | 0.282107671 | Q61425 | Hydroxyacyl-coenzyme A dehydrogenase, mitochondrial | Hadh | 24.38863754 | 24.43485451 | 24.56296349 | 24.18343925 | 24.22184563 | 24.13484764 |
| **+** | 1.658877673 | 0.282953262 | P62075;A0A1W2P756;A0A1W2P7H2 | Mitochondrial import inner membrane translocase subunit Tim13 | Timm13 | 24.43071365 | 24.40106392 | 24.37775803 | 24.27241516 | 24.05017662 | 24.03808403 |
| **+** | 2.191439121 | 0.283930143 | Q9JMG1 | Endothelial differentiation-related factor 1 | Edf1 | 22.90855598 | 22.94218636 | 22.98161697 | 22.61802483 | 22.70235443 | NaN |
| **+** | 3.550716119 | 0.284404755 | O89023 | Tripeptidyl-peptidase 1 | Tpp1 | 26.12911987 | 26.13018036 | 26.14460564 | 25.83963776 | 25.89478874 | 25.81626511 |
| **+** | 1.604507865 | 0.284922282 | A0A0G2JGQ4;P54729 | NEDD8 ultimate buster 1 | Nub1 | 23.08642769 | 23.31091309 | 23.19989777 | 22.82483292 | 22.99458885 | 22.92304993 |
| **+** | 2.999678465 | 0.286783218 | P68181;H6TMF5;A0A0G2JFT9 | cAMP-dependent protein kinase catalytic subunit beta | Prkacb | 23.67238808 | 23.73376083 | 23.78576469 | 23.43185997 | 23.44909668 | 23.4506073 |
| **+** | 2.402321241 | 0.287433624 | Q9QZQ8;Q8CCK0 | Core histone macro-H2A.1 | H2afy | 25.56246948 | 25.42636871 | 25.48440742 | 25.21174622 | 25.2469902 | 25.15220833 |
| **+** | 1.687487008 | 0.289196014 | Q9EQ06;A8Y5N4 | Estradiol 17-beta-dehydrogenase 11 | Hsd17b11 | 24.68281746 | 24.81889915 | 24.73536301 | 24.52557945 | 24.52133179 | 24.32258034 |
| **+** | 2.153258925 | 0.289547602 | Q91XV3 | Brain acid soluble protein 1 | Basp1 | 27.27488708 | 27.23480225 | 27.31838036 | 26.95015907 | 26.92116165 | 27.08810616 |
| **+** | 4.104763297 | 0.289936066 | Q60930;A0A286YCR8;D3YZT5;D3YUN8 | Voltage-dependent anion-selective channel protein 2 | Vdac2 | 28.00088501 | 28.03616714 | 28.01749229 | 27.72987747 | 27.70272827 | 27.75213051 |
| **+** | 1.503980876 | 0.290278117 | Q8R1Q8 | Cytoplasmic dynein 1 light intermediate chain 1 | Dync1li1 | 24.12084007 | 24.2191143 | 24.26018906 | 23.94225883 | 23.75938797 | 24.02766228 |
| **+** | 1.305168872 | 0.291201274 | A2AH25;Q5FWK3 | Rho GTPase-activating protein 1 | Arhgap1 | 24.44752121 | 24.31243706 | 24.4639473 | 24.27027893 | 24.13053322 | 23.94948959 |
| **+** | 4.24605608 | 0.29186058 | Q61233;D3YZ25;D3YVW8;D3Z7D9;D3Z311 | Plastin-2 | Lcp1 | 28.55376625 | 28.5997448 | 28.59384537 | 28.29355621 | 28.27639771 | 28.30182076 |
| **+** | 1.452658312 | 0.292945226 | Q8BXZ1;A0A494BB11;A0A494B9J5;A0A494B9P2 | Protein disulfide-isomerase TMX3 | Tmx3 | 24.32608223 | 24.38475609 | 24.57166862 | 24.05200386 | 24.24481392 | 24.10685349 |
| **+** | 1.969345454 | 0.295307795 | E9Q1W0;E9Q1T1;A0A0G2JGS4;Q6PHZ2;E9Q1V9;Q8CCM0;E9PXV3;F6RWZ9;F8WIS9;P11798;E9QAJ4;D6RDQ8;A0A286YCB8;A0A286YDL6;A0A286YDK9;F6WHR9 | Calcium/calmodulin-dependent protein kinase type II subunit delta | Camk2d | 24.51797295 | 24.57911301 | 24.63134193 | 24.16783142 | 24.34502792 | 24.32964516 |
| **+** | 1.404554814 | 0.298002243 | Q78IK2 | Up-regulated during skeletal muscle growth protein 5 | Usmg5 | 24.53534317 | 24.42239571 | 24.44272041 | NaN | 24.2657795 | 24.07185555 |
| **+** | 2.219438884 | 0.2997214 | Q8R5J9 | PRA1 family protein 3 | Arl6ip5 | 24.54456902 | 24.73830414 | 24.66258812 | 24.34928703 | 24.34475708 | 24.35225296 |
| **+** | 2.479611672 | 0.300669988 | P61161 | Actin-related protein 2 | Actr2 | 27.63464165 | 27.70892334 | 27.71705437 | 27.34511185 | 27.34688759 | 27.46660995 |
| **+** | 1.984072051 | 0.30101649 | D3Z7C6;Q9R0Q7 | Prostaglandin E synthase 3 | Ptges3 | 25.99774361 | 25.90825272 | 25.77791214 | 25.61675262 | 25.60387993 | 25.56022644 |
| **+** | 3.225975481 | 0.301515579 | Q99N15;A2AFQ2;O08756 | 3-hydroxyacyl-CoA dehydrogenase type-2 | Hsd17b10 | 25.16065598 | 25.15193748 | 25.10541534 | 24.88543892 | 24.79902077 | 24.82900238 |
| **+** | 1.941745229 | 0.302094777 | P70303;A2AEQ5 | CTP synthase 2 | Ctps2 | 23.33851242 | 23.23068047 | 23.10844994 | 22.89809227 | 22.92194176 | 22.95132446 |
| **+** | 3.692364615 | 0.302506765 | Q8BKC5 | Importin-5 | Ipo5 | 24.66779518 | 24.60670662 | 24.64256859 | 24.31824112 | 24.36652946 | 24.32477951 |
| **+** | 2.100979579 | 0.304513931 | Q9ESY9 | Gamma-interferon-inducible lysosomal thiol reductase | Ifi30 | 23.17852402 | 23.29712105 | 23.34759712 | 23.03863716 | 22.95583534 | 22.91522789 |
| **+** | 1.574480393 | 0.306055705 | P62748;A0A1Y7VMK3;P84075;A2A7R5;E9PV73;D3Z2Z8;D3YVA2;Q91X97 | Hippocalcin-like protein 1;Neuron-specific calcium-binding protein hippocalcin | Hpcal1;Hpca | 24.29921913 | 24.28333855 | 24.17440033 | 23.78666115 | 24.04101181 | 24.01111794 |
| **+** | 1.859375963 | 0.308436076 | Q99NB9;G5E866;A0A087WNS2 | Splicing factor 3B subunit 1 | Sf3b1 | 23.32526016 | 23.24662781 | 23.34583855 | 22.90652084 | 22.95737648 | 23.12852097 |
| **+** | 1.713768047 | 0.309574127 | Q58A65 | C-Jun-amino-terminal kinase-interacting protein 4 | Spag9 | 22.83711243 | 22.66457558 | 22.69704056 | 22.5320282 | 22.31619835 | 22.42177963 |
| **+** | 2.715339227 | 0.310221354 | A0A498WGK2;Q8BP47;A0A494BAX5;A0A494BB89;A0A494B927;A0A494BAW6 | Asparagine--tRNA ligase, cytoplasmic | Nars | 24.92442703 | 24.90724373 | 24.79902077 | 24.58726311 | 24.5327301 | 24.58003426 |
| **+** | 1.606290835 | 0.311999639 | Q8BVY0;A0A2R8VHW2 | Ribosomal L1 domain-containing protein 1 | Rsl1d1 | 23.41712379 | 23.63798904 | 23.35043335 | 23.19460297 | 23.1336422 | 23.14130211 |
| **+** | 1.30691445 | 0.312996864 | Q8R3V5;A2AWI7;A2AWI9 | Endophilin-B2 | Sh3glb2 | 22.566185 | 22.3743 | 22.31752396 | 22.0908432 | 22.12183571 | NaN |
| **+** | 2.583523426 | 0.313525518 | P70296;D3Z1V4;D6RHS6;E9QLE5;Q8VIN1 | Phosphatidylethanolamine-binding protein 1;Hippocampal cholinergic neurostimulating peptide | Pebp1 | 27.14530373 | 27.15605354 | 27.2099247 | 26.79983521 | 26.93988419 | 26.83098602 |
| **+** | 1.474097748 | 0.315465927 | P23591;A0A2R8VI39;A0A2R8VHD0;A0A2R8W6P6;A0A2R8VKL9;A0A2R8W6N0 | GDP-L-fucose synthase | Tsta3 | 23.62834167 | 23.84775925 | 23.92804718 | 23.55952644 | 23.48664856 | 23.41157532 |
| **+** | 1.220618717 | 0.315577825 | Q9D880 | Mitochondrial import inner membrane translocase subunit TIM50 | Timm50 | 23.47753716 | 23.78735924 | 23.84565544 | 23.31423569 | 23.3925724 | 23.45701027 |
| **+** | 3.651759589 | 0.316433589 | O08529 | Calpain-2 catalytic subunit | Capn2 | 25.9077034 | 25.84254074 | 25.85581017 | 25.52945709 | 25.58078194 | 25.54651451 |
| **+** | 1.778416367 | 0.319096247 | B1AWE0;Q6PFA2;B1AWE1;B1AWD8;B1AWD9;O08585 | Clathrin light chain A | Clta | 25.80289459 | 26.01797104 | 25.8554554 | 25.4855442 | 25.65072441 | 25.58276367 |
| **+** | 3.323859207 | 0.319526672 | Q61171;D3Z4A4 | Peroxiredoxin-2 | Prdx2 | 27.53904724 | 27.58992195 | 27.61435318 | 27.22250938 | 27.29473877 | 27.2674942 |
| **+** | 2.163628707 | 0.32045873 | Q9CZ13;A0A0A6YW82;A0A0A6YWX6;A0A0A6YVZ0 | Cytochrome b-c1 complex subunit 1, mitochondrial | Uqcrc1 | 25.20765495 | 25.12884331 | 25.19584274 | 24.97070694 | 24.8150425 | 24.78521538 |
| **+** | 1.828722887 | 0.321828206 | P57716 | Nicastrin | Ncstn | 24.07357407 | 24.10429573 | 24.17310333 | 23.66123009 | 23.81293869 | 23.91131973 |
| **+** | 2.50552516 | 0.325828552 | P28474;A0A0G2JGS3;Q64437 | Alcohol dehydrogenase class-3 | Adh5 | 25.41322327 | 25.51117134 | 25.46254539 | 25.14487648 | 25.20582962 | 25.05874825 |
| **+** | 2.643950216 | 0.32685407 | Q9JHF5;F6XRE6;F6ZFB8;A0A494B9E3 | V-type proton ATPase subunit a | Tcirg1 | 25.81048965 | 25.76375008 | 25.69373131 | 25.38922882 | 25.40418625 | 25.49399376 |
| **+** | 4.391632663 | 0.327301661 | P21981;G3UXE8;Q9D7I9 | Protein-glutamine gamma-glutamyltransferase 2 | Tgm2 | 27.75321579 | 27.72318649 | 27.77517509 | 27.43396378 | 27.40916443 | 27.42654419 |
| **+** | 1.469297803 | 0.328766505 | Q9D172;A0A1W2P7B6;A0A1W2P870 | ES1 protein homolog, mitochondrial | D10Jhu81e | 24.26363182 | 24.5354023 | 24.35124207 | 24.12178802 | 24.11957359 | 23.92261505 |
| **+** | 1.901647395 | 0.330106735 | A0A0A0MQA5;P68368;A0A087WQS4;A0A087WRB4;A0A087WSB0;A0A087WSL5;Q3UX10;A0A087WS35 | Tubulin alpha-4A chain | Tuba4a | 25.52414513 | 25.3899498 | 25.56281853 | 25.12734985 | 25.27156067 | 25.08768272 |
| **+** | 3.460040411 | 0.330119451 | Q6DFW4;A0A0A0MQ76;A0A087WQ46;A0A087WSU5;A0A087WSL8;A0A087WP00;A0A087WNW0 | Nucleolar protein 58 | Nop58 | 23.61537743 | 23.57957458 | 23.58932304 | 23.31395912 | 23.25954247 | 23.22041512 |
| **+** | 3.209232976 | 0.330555598 | Q7TPR4;A1BN54 | Alpha-actinin-1 | Actn1 | 26.83833885 | 26.80069923 | 26.82055473 | 26.47795296 | 26.44029808 | 26.54967499 |
| **+** | 1.680849428 | 0.331918716 | Q9WV55;A0A3B2W837 | Vesicle-associated membrane protein-associated protein A | Vapa | 25.16993523 | 25.13578796 | 25.15719032 | 24.9908371 | 24.68741798 | 24.78890228 |
| **+** | 1.398360797 | 0.332154592 | O54833;A0A1D5RM74;A0A1D5RLE4;A0A1D5RM55 | Casein kinase II subunit alpha | Csnk2a2 | 22.47330666 | 22.53618622 | 22.30120277 | 22.14435577 | 22.06513214 | NaN |
| **+** | 1.547303327 | 0.3360672 | Q8BFW7 | Lipoma-preferred partner homolog | Lpp | 23.20099068 | 23.31934547 | 23.26927948 | 22.74071503 | 22.99903488 | 23.04166412 |
| **+** | 1.981835043 | 0.338026047 | Q8BFZ9;A0A1B0GSD8;A0A1B0GT70;A0A1B0GQZ0;A0A1B0GT43;A0A1B0GRQ1;A0A1B0GRG7;E9PUH5 | Erlin-2 | Erlin2 | 23.59126472 | 23.7010231 | 23.75928688 | 23.43834877 | 23.24601936 | 23.35312843 |
| **+** | 2.651034552 | 0.338051478 | Q9DB20;A0A338P7G3;A0A338P776;F7D3P8;F6XVM5 | ATP synthase subunit O, mitochondrial | Atp5o | 26.1521492 | 26.08170128 | 26.14941978 | 25.79728889 | 25.71221924 | 25.8596077 |
| **+** | 2.993663997 | 0.339058558 | Q9EQH3 | Vacuolar protein sorting-associated protein 35 | Vps35 | 25.7957058 | 25.66370201 | 25.70131302 | 25.37388229 | 25.38007355 | 25.38958931 |
| **+** | 1.423214401 | 0.34237353 | Q99KF1;A0A286YDS5 | Transmembrane emp24 domain-containing protein 9 | Tmed9 | 25.51668167 | 25.63619232 | 25.74095726 | 25.17417145 | 25.46932983 | 25.22320938 |
| **+** | 1.669479431 | 0.344120026 | Q99JY0;D3YXU1 | Trifunctional enzyme subunit beta, mitochondrial;3-ketoacyl-CoA thiolase | Hadhb | 24.87070084 | 24.71460533 | 24.65212059 | 24.26906586 | 24.44518471 | 24.49081612 |
| **+** | 1.39652472 | 0.346090953 | Q9EPL8 | Importin-7 | Ipo7 | 22.98534012 | 22.94494057 | 22.89733505 | 22.40082932 | 22.59723473 | 22.79127884 |
| **+** | 1.296089351 | 0.348007202 | Q07417 | Short-chain specific acyl-CoA dehydrogenase, mitochondrial | Acads | 24.47969627 | 24.35816956 | 24.40723801 | 23.8303566 | 24.14336014 | 24.22736549 |
| **+** | 1.698344378 | 0.349849065 | Q9QZD8 | Mitochondrial dicarboxylate carrier | Slc25a10 | 23.15557861 | 23.29319572 | 23.183424 | 23.01483345 | 22.83965111 | 22.72816658 |
| **+** | 1.322244102 | 0.350353241 | O09126 | Semaphorin-4D | Sema4d | 22.62220955 | 22.77235413 | 22.92945671 | 22.54658508 | 22.4697094 | 22.25666618 |
| **+** | 2.681631951 | 0.35080719 | P29416 | Beta-hexosaminidase subunit alpha | Hexa | 26.53113937 | 26.45860672 | 26.38891602 | 26.07071114 | 26.09331512 | 26.16221428 |
| **+** | 2.871351512 | 0.352113724 | A2AMW0;F7CAZ6 | F-actin-capping protein subunit beta | Capzb | 26.91307831 | 26.94022179 | 26.80143929 | 26.51550865 | 26.52643013 | 26.55645943 |
| **+** | 1.164613283 | 0.353155136 | P70279;A2ALA0 | Surfeit locus protein 6 | Surf6 | 22.21397781 | 22.62352753 | 22.5273819 | 22.05956459 | 22.00549126 | 22.24036598 |
| **+** | 1.68613551 | 0.358359019 | A0A0A0MQM0;P63242;Q8BGY2;J3QPS8 | Eukaryotic translation initiation factor 5A;Eukaryotic translation initiation factor 5A-1;Eukaryotic translation initiation factor 5A-2 | Eif5a;Eif5a2 | 26.34395981 | 26.42993164 | 26.16204071 | 25.85052681 | 26.04074097 | 25.96958733 |
| **+** | 2.189047525 | 0.358436584 | P30204;A0A1B0GRS5 | Macrophage scavenger receptor types I and II | Msr1 | 26.09115791 | 26.13102531 | 26.07136536 | 25.760149 | 25.61523628 | 25.84285355 |
| **+** | 2.932884428 | 0.35967954 | Q9WV54;D3Z505 | Acid ceramidase;Acid ceramidase subunit alpha;Acid ceramidase subunit beta | Asah1 | 25.39715195 | 25.32457352 | 25.41696358 | 25.0646801 | 24.95500183 | 25.03996849 |
| **+** | 2.350507442 | 0.360205332 | Q08857;A0A0G2JFB7 | Platelet glycoprotein 4 | Cd36 | 27.33892059 | 27.30530357 | 27.26472473 | 26.86407852 | 27.05723953 | 26.90701485 |
| **+** | 3.656107939 | 0.361246109 | P63038;D3Z2F2;D3Z7J9 | 60 kDa heat shock protein, mitochondrial | Hspd1 | 27.11436081 | 27.09305382 | 27.15865517 | 26.80217934 | 26.7358284 | 26.74432373 |
| **+** | 2.333584112 | 0.361435572 | F8VQC1;E9Q740 | Signal recognition particle subunit SRP72 | Srp72 | 24.44290924 | 24.50917816 | 24.44012451 | 24.11680031 | 24.197155 | 23.99394989 |
| **+** | 2.635364914 | 0.362333298 | P35564 | Calnexin | Canx | 26.51589966 | 26.5268631 | 26.45126724 | 26.04789162 | 26.15079498 | 26.20834351 |
| **+** | 2.08292201 | 0.363438288 | O54774;A0A1W2P6Q6 | AP-3 complex subunit delta-1 | Ap3d1 | 24.30709457 | 24.35769844 | 24.21170807 | 23.84507942 | 24.04809952 | 23.89300728 |
| **+** | 1.187968527 | 0.36571312 | S4R1X1;Q7TMQ7;D3Z101;D3Z0V8 | WD repeat-containing protein 91 | Wdr91 | 23.70440483 | 23.71030235 | 23.71962738 | 23.21170807 | 23.19070625 | 23.63478088 |
| **+** | 1.732930236 | 0.365791321 | Q9QZM0;Q99NB8 | Ubiquilin-2 | Ubqln2 | 23.2478447 | 23.15913963 | 23.29544067 | 23.01125336 | 22.88147736 | 22.71232033 |
| **+** | 3.691342717 | 0.366525014 | P62983;A0A0A6YW67;E9Q9J0;E9Q4P0;E9Q5F6;E9QNP0;Q5SX22;P62984;P0CG49;P0CG50 | Ubiquitin-40S ribosomal protein S27a;Ubiquitin;40S ribosomal protein S27a;Ubiquitin-60S ribosomal protein L40;Ubiquitin;60S ribosomal protein L40;Polyubiquitin-B;Ubiquitin;Polyubiquitin-C;Ubiquitin;Ubiquitin-related 1;Ubiquitin-related 2 | Rps27a;Gm8797;Uba52;Kxd1;Ubc;Ubb | 28.33794403 | 28.38895035 | 28.37836266 | 27.96389008 | 28.04510307 | 27.99668884 |
| **+** | 1.199746554 | 0.367599487 | G3X9H7;Q8R1S4;A0A2I3BQ92;A0A2I3BPM9;A0A2I3BQ67 | Metastasis suppressor protein 1 | Mtss1 | NaN | 22.97114563 | 22.99441528 | 22.71150017 | NaN | 22.51886177 |
| **+** | 1.504405909 | 0.367908478 | Q8VD04;A2AEW9;A2AEW8;A2AEW6;A2AEW5 | GRIP1-associated protein 1 | Gripap1 | 22.17145538 | 22.15158844 | 22.16137123 | 21.75156403 | 21.62229919 | 22.0068264 |
| **+** | 2.316590723 | 0.369740168 | A2AU62;Q64012;A2AU61;A2AU60 | RNA-binding protein Raly | Raly | 24.1649971 | 24.29858971 | 24.09691811 | 23.7957058 | 23.78346825 | 23.87211037 |
| **+** | 1.730706371 | 0.369890213 | Q61937;Q5SQB0;Q9DAY9;Q5SQB5 | Nucleophosmin | Npm1 | 27.31432152 | 27.0348568 | 27.13476944 | 26.86301613 | 26.69192314 | 26.81933784 |
| **+** | 2.10467315 | 0.370552699 | Q62093 | Serine/arginine-rich splicing factor 2 | Srsf2 | 25.68877792 | 25.75692177 | 25.65839958 | 25.42854309 | 25.19696808 | 25.36693001 |
| **+** | 1.901287494 | 0.373706182 | Q62048;D3Z375 | Astrocytic phosphoprotein PEA-15 | Pea15;Pea15a | 25.31703377 | 25.36265755 | 25.3998909 | 24.91793442 | 24.88855362 | 25.15197563 |
| **+** | 1.159213198 | 0.375106176 | E9Q634;A0A1L1STM1 | Unconventional myosin-Ie | Myo1e | 23.6563282 | 23.22390938 | 23.51086998 | 23.07535362 | 23.23984146 | 22.95059395 |
| **+** | 1.761117877 | 0.37646548 | Q61263;A0A087WNN8;A0A087WSJ5 | Sterol O-acyltransferase 1 | Soat1 | 24.9432869 | 24.74806595 | 24.80252457 | 24.32930374 | 24.59366035 | 24.44151688 |
| **+** | 1.286534122 | 0.376943588 | P84089;A0A1W2P7H9;A0A1W2P7T3;G3UW85 | Enhancer of rudimentary homolog | Erh | 25.51092911 | 25.54427528 | 25.45444107 | 25.24008942 | 24.85761452 | 25.28111076 |
| **+** | 2.536485117 | 0.378629684 | Q9CRD0;A0A0J9YUB6;A0A0J9YUK7;A0A0J9YTV6;A0A0J9YTV7 | OCIA domain-containing protein 1 | Ociad1 | 23.80607033 | 23.6496563 | 23.61784554 | 23.3212738 | 23.31035805 | 23.30605125 |
| **+** | 2.562635208 | 0.379461924 | Q8CBB7;P22892 | AP-1 complex subunit gamma-1 | Ap1g1 | 23.19160843 | 23.36024666 | 23.24994278 | 22.90780449 | 22.82989311 | 22.92571449 |
| **+** | 1.956247953 | 0.379769007 | Q922Q4;A2ABZ3;A2ABZ0;A2ABZ2;Q922W5 | Pyrroline-5-carboxylate reductase 2 | Pycr2 | 23.34570503 | 23.55203819 | 23.43096733 | 22.94966888 | 23.08477402 | 23.15496063 |
| **+** | 1.435047519 | 0.380310376 | E9QP59;D3YU56;Q9WU40 | Inner nuclear membrane protein Man1 | Lemd3 | 20.24051094 | 20.39383125 | 20.19556427 | 19.80006599 | 19.99258423 | NaN |
| **+** | 2.392766949 | 0.380690893 | Q05D44 | Eukaryotic translation initiation factor 5B | Eif5b | 24.42284393 | 24.39702034 | 24.34394455 | 23.92542458 | 24.12376213 | 23.97254944 |
| **+** | 2.88513004 | 0.382226944 | A0A1L1STE6;Q9D6R2 | Isocitrate dehydrogenase [NAD] subunit alpha, mitochondrial | Idh3a | 24.66930962 | 24.64306259 | 24.77208138 | 24.34204483 | 24.33647156 | 24.25925636 |
| **+** | 2.900083706 | 0.382630666 | Q9CZU6;Q80X68 | Citrate synthase, mitochondrial;Citrate synthase | Cs;Csl | 26.70516968 | 26.59889221 | 26.66370201 | 26.30535507 | 26.20209694 | 26.31241989 |
| **+** | 2.198682316 | 0.383964539 | P51569;Q8BGZ6;A2BDV6 | Alpha-galactosidase A | Gla | 25.76258469 | 25.78011703 | 25.59428596 | 25.24927139 | 25.39744568 | 25.338377 |
| **+** | 1.601357984 | 0.384111404 | Q9CQI3;A0A2I3BR94;A0A2I3BPS1;D3YY16 | Glia maturation factor beta | Gmfb | 26.05854225 | 26.10189438 | 25.90825272 | 25.82286644 | 25.52387619 | 25.5696125 |
| **+** | 1.843602579 | 0.385389328 | A0A171KXD3;Q9JIF0;A0A140LJF4;A0A140LHF7;Q6PAK3 | Protein arginine N-methyltransferase 1 | Prmt1 | 23.65818214 | 23.72608757 | 23.72858238 | 23.14094353 | 23.43249893 | 23.38324165 |
| **+** | 2.129108903 | 0.385863622 | P40336;A0A1W2P7Z9;A0A1W2P7R7 | Vacuolar protein sorting-associated protein 26A | Vps26a | 25.16065598 | 25.34807014 | 25.39855385 | 24.86400795 | 24.94248199 | 24.94319916 |
| **+** | 1.373317672 | 0.387318929 | Q8C872;Q62351 | Transferrin receptor protein 1 | Tfrc | 22.82596016 | 22.73640633 | 22.81113625 | 22.34892082 | 22.21347427 | 22.64915085 |
| **+** | 2.69189435 | 0.389639537 | Q8CAQ8;E9Q800;E9QAY6;A0A1B0GX08;A0A0U1RQ14;E9PVS5 | MICOS complex subunit Mic60 | Immt | 24.71224594 | 24.8207016 | 24.70793533 | 24.42226791 | 24.28383255 | 24.3658638 |
| **+** | 2.23866719 | 0.389713923 | Q99J83 | Autophagy protein 5 | Atg5 | 23.06749725 | 23.08901596 | 23.15847778 | 22.74564743 | 22.81321335 | 22.58698845 |
| **+** | 2.575448247 | 0.390128454 | Q60932 | Voltage-dependent anion-selective channel protein 1 | Vdac1 | 26.26542091 | 26.32211494 | 26.17276001 | 25.81401634 | 25.83479881 | 25.94109535 |
| **+** | 4.072086501 | 0.393152873 | Q8VEB4;A0A1D5RLZ5 | Group XV phospholipase A2 | Pla2g15 | 23.68367577 | 23.7113533 | 23.69327927 | 23.28630257 | 23.34813881 | 23.27440834 |
| **+** | 1.543902095 | 0.394767125 | Q8BIG7;H3BJ37 | Catechol O-methyltransferase domain-containing protein 1 | Comtd1 | 24.53136253 | 24.62148285 | 24.68388939 | 24.25781822 | 24.01137352 | 24.38324165 |
| **+** | 2.672679905 | 0.396292369 | P62259;D6REF3;F6WA09 | 14-3-3 protein epsilon | Ywhae | 27.38327408 | 27.34265518 | 27.34502792 | 26.97059631 | 26.86159706 | 27.0498867 |
| **+** | 2.950137196 | 0.396429062 | Q8R0H9;A0A2R8VI72 | ADP-ribosylation factor-binding protein GGA1 | Gga1 | 23.27753258 | 23.17689705 | 23.2414875 | 22.8633461 | 22.76186371 | 22.88142014 |
| **+** | 1.352086006 | 0.396744092 | Q3U422 | NADH dehydrogenase [ubiquinone] flavoprotein 3, mitochondrial | Ndufv3 | 21.86359215 | 21.71225739 | 22.00970078 | 21.40758705 | 21.52262497 | NaN |
| **+** | 2.609772006 | 0.397179921 | P62137 | Serine/threonine-protein phosphatase PP1-alpha catalytic subunit | Ppp1ca | 25.37122536 | 25.4107666 | 25.48907089 | 24.9395256 | 25.10149384 | 25.03850365 |
| **+** | 2.83758749 | 0.397380829 | P09671;A0A3B2WBF0 | Superoxide dismutase [Mn], mitochondrial | Sod2 | 26.42207527 | 26.47756767 | 26.45528793 | 26.12677765 | 25.96276474 | 26.073246 |
| **+** | 1.220090462 | 0.400069555 | A0A0J9YUF8;A0A0J9YTU3;Q5SXY1;A0A0J9YV86;A0A0J9YUR2;A0A0J9YV47;A0A0J9YUG8 | Cytospin-B | Specc1 | 23.69359779 | 23.9392128 | 23.56022644 | 23.43631554 | 23.43923569 | 23.11727715 |
| **+** | 1.291240397 | 0.400348028 | F7CUQ1;Q91WX5;O08734 | Bcl-2 homologous antagonist/killer | Bak1 | 23.80646324 | 23.52049255 | 23.34028053 | 23.17327118 | 23.23518944 | 23.05773163 |
| **+** | 2.074863514 | 0.40382576 | P22437 | Prostaglandin G/H synthase 1 | Ptgs1 | 23.57207298 | 23.47432137 | 23.46188927 | 22.98896599 | 23.24445152 | 23.06338882 |
| **+** | 2.98437019 | 0.405421575 | Q9Z2M7;Q9D6D9;Q91W01;A0A2R8W6J8;O35621 | Phosphomannomutase 2 | Pmm2 | 24.14398193 | 24.24966812 | 24.19925499 | 23.81704521 | 23.7204628 | 23.83913231 |
| **+** | 2.982124614 | 0.40681076 | Q9D0K2;Q3UJQ9 | Succinyl-CoA:3-ketoacid coenzyme A transferase 1, mitochondrial;Succinyl-CoA:3-ketoacid-coenzyme A transferase | Oxct1 | 25.37278557 | 25.32875633 | 25.40872765 | 24.95553398 | 24.8950882 | 25.03921509 |
| **+** | 2.342164679 | 0.406867345 | O09117 | Synaptophysin-like protein 1 | Sypl1 | 25.68045998 | 25.65253067 | 25.61980438 | 25.18002701 | NaN | 25.30810165 |
| **+** | 1.759275684 | 0.408266703 | Q9CR51 | V-type proton ATPase subunit G 1 | Atp6v1g1 | 26.6320076 | 26.67868805 | 26.40174675 | 26.27002907 | 26.15437317 | 26.06324005 |
| **+** | 1.025828353 | 0.40845871 | Q8BFQ8 | Parkinson disease 7 domain-containing protein 1 | Pddc1 | 23.02729034 | NaN | 23.04286766 | 22.76152039 | 22.4917202 | NaN |
| **+** | 1.236665924 | 0.408496857 | Q8R5L3 | Vam6/Vps39-like protein | Vps39 | 22.81638145 | 22.65890121 | 22.90571213 | NaN | 22.25133133 | 22.51900482 |
| **+** | 3.109183635 | 0.409953435 | P17439;A0A0G2JDK2 | Glucosylceramidase | Gba | 25.72782898 | 25.57432747 | 25.64229202 | 25.24048996 | 25.2303009 | 25.2437973 |
| **+** | 3.260674008 | 0.411554337 | P84078;P61205 | ADP-ribosylation factor 1;ADP-ribosylation factor 3 | Arf1;Arf3 | 27.08263588 | 26.96752167 | 27.0519619 | 26.66030502 | 26.58376884 | 26.62338257 |
| **+** | 1.491979445 | 0.411828995 | P00405 | Cytochrome c oxidase subunit 2 | Mtco2 | 26.73995399 | 26.45771599 | 26.72142792 | 26.2530098 | 26.06165504 | 26.36894608 |
| **+** | 1.368012282 | 0.413017273 | P85094 | Isochorismatase domain-containing protein 2A, mitochondrial | Isoc2a | 23.83807373 | 23.55426407 | 23.78745842 | 23.3755722 | 23.25159073 | NaN |
| **+** | 4.206381817 | 0.413304647 | F6WR04;O70370 | Cathepsin S | Ctss | 29.00646019 | 29.01241302 | 29.03388596 | 28.56354523 | 28.60999298 | 28.63930702 |
| **+** | 4.930372382 | 0.414183299 | Q8BIJ6;E9PWN2;E9PWN3 | Isoleucine--tRNA ligase, mitochondrial | Iars2 | 23.15027237 | 23.1972599 | 23.15775299 | 22.74769783 | 22.76484299 | 22.75019455 |
| **+** | 2.232071169 | 0.414663951 | Q9JIG7 | Coiled-coil domain-containing protein 22 | Ccdc22 | 23.39780426 | 23.52874184 | 23.32855034 | 22.90275383 | 23.05424118 | 23.05410957 |
| **+** | 1.340012167 | 0.414900462 | Q7TS64;Q99MK8;F6Y9P3;F6QY34 | Beta-adrenergic receptor kinase 1 | Adrbk1 | 22.93887138 | 23.08024216 | 22.73106956 | 22.47343063 | 22.33862305 | 22.69342804 |
| **+** | 1.789804111 | 0.415029526 | P97807;H3BKG7 | Fumarate hydratase, mitochondrial | Fh | 24.70038795 | 24.63854027 | 24.70165825 | 24.06496811 | 24.33285713 | 24.39767265 |
| **+** | 1.410973984 | 0.415312449 | Q9CQW1 | Synaptobrevin homolog YKT6 | Ykt6 | 24.27980042 | 24.04934692 | 24.16737175 | 23.65009499 | 23.6115551 | 23.98893166 |
| **+** | 2.471805645 | 0.415677389 | P62715;P63330 | Serine/threonine-protein phosphatase 2A catalytic subunit beta isoform;Serine/threonine-protein phosphatase 2A catalytic subunit alpha isoform | Ppp2cb;Ppp2ca | 24.86277962 | 24.82284164 | 24.70582771 | 24.28714943 | 24.42476845 | 24.43249893 |
| **+** | 2.799595662 | 0.416595459 | P11438 | Lysosome-associated membrane glycoprotein 1 | Lamp1 | 28.47996712 | 28.40450287 | 28.43141365 | 28.1208992 | 27.98505974 | 27.96013832 |
| **+** | 4.618521675 | 0.416994731 | A0A7N9VR94 | AHNAK nucleoprotein 2 | Ahnak2 | 27.58970833 | 27.56034279 | 27.59398842 | 27.17455292 | 27.18451691 | 27.13398552 |
| **+** | 3.727763815 | 0.418750763 | Q6NZD2;Q9WV80;D3YWH1 | Sorting nexin-1 | Snx1 | 25.21656418 | 25.27366066 | 25.19418907 | 24.8275013 | 24.83301544 | 24.76764488 |
| **+** | 1.372164944 | 0.418946266 | Q8CGA3 | Large neutral amino acids transporter small subunit 4 | Slc43a2 | 22.89115334 | 23.09789848 | 23.12235641 | 22.7239666 | 22.51241302 | NaN |
| **+** | 2.430926758 | 0.420186361 | Q3TCN2 | Putative phospholipase B-like 2;Putative phospholipase B-like 2 28 kDa form;Putative phospholipase B-like 2 40 kDa form;Putative phospholipase B-like 2 15 kDa form | Plbd2 | 24.1848774 | 24.25832176 | 24.29593086 | 23.70956612 | 23.85357666 | 23.91542816 |
| **+** | 2.326369738 | 0.424119314 | Q791V5;A2AFW6;Q9D050 | Mitochondrial carrier homolog 2 | Mtch2 | 24.92374802 | 24.95287132 | 24.81509209 | 24.3973465 | 24.42675209 | 24.5952549 |
| **+** | 1.280945688 | 0.424744924 | Q91X76;A0A2I3BQR1;A0A2I3BRL0;A0A2I3BR81;A0A2I3BPH8 | 5'-nucleotidase domain containing 2 | Nt5dc2 | 22.10373688 | 22.21053505 | 22.13599014 | 21.85821342 | 21.42162514 | 21.89618874 |
| **+** | 1.508663919 | 0.428002675 | P35486 | Pyruvate dehydrogenase E1 component subunit alpha, somatic form, mitochondrial | Pdha1 | 24.17767334 | 24.30103493 | 24.09095573 | 23.83190346 | 23.91870689 | 23.53504562 |
| **+** | 1.504901097 | 0.428520838 | Q8C0E3 | Tripartite motif-containing protein 47 | Trim47 | 22.63719177 | 22.48505211 | 22.52348709 | NaN | 21.99268723 | 22.24742508 |
| **+** | 2.934649209 | 0.439671834 | Q99L45;E0CXJ3 | Eukaryotic translation initiation factor 2 subunit 2 | Eif2s2 | 25.91209602 | 25.98626518 | 26.04428864 | 25.50254822 | 25.50621414 | 25.61487198 |
| **+** | 2.726106728 | 0.439706167 | Q8BMF4 | Dihydrolipoyllysine-residue acetyltransferase component of pyruvate dehydrogenase complex, mitochondrial | Dlat | 24.78016663 | 24.79183578 | 24.77680779 | 24.32587624 | 24.24887276 | 24.4549427 |
| **+** | 1.019822552 | 0.441878637 | Q8CFQ9;P56959;G3UXT7;G3UZD2;Q91VQ2 | RNA-binding protein FUS | Fus | 24.83422089 | 24.38837433 | 24.82221031 | 24.50336647 | 24.02157593 | 24.19422722 |
| **+** | 2.926514103 | 0.443359375 | Q9D0M3;A0A2R8VHK1 | Cytochrome c1, heme protein, mitochondrial | Cyc1 | 24.54274178 | 24.49185562 | 24.62611389 | 24.05034447 | 24.10261536 | 24.17767334 |
| **+** | 3.034012534 | 0.44404157 | P53810;J3QQ30;J3QPW1;F8WGG5 | Phosphatidylinositol transfer protein alpha isoform | Pitpna | 26.65703773 | 26.58348083 | 26.74752808 | 26.18415833 | 26.23192978 | 26.23983383 |
| **+** | 1.730012389 | 0.445438385 | P26645 | Myristoylated alanine-rich C-kinase substrate | Marcks | 25.58514404 | 25.37779236 | 25.74077797 | 25.19944191 | 25.0294323 | 25.13852501 |
| **+** | 2.143277085 | 0.448188146 | Q8BKZ9;A2AWH8;A2AWH7 | Pyruvate dehydrogenase protein X component, mitochondrial | Pdhx | 22.64321709 | 22.74166107 | 22.91261673 | 22.24898911 | 22.3898716 | 22.31406975 |
| **+** | 1.401836509 | 0.450490316 | P05132 | cAMP-dependent protein kinase catalytic subunit alpha | Prkaca | 22.59388733 | 23.06733322 | 22.83978653 | 22.30449295 | 22.34131241 | 22.50373077 |
| **+** | 3.579672741 | 0.451131821 | P55772;Q8CDV7;F7B9M9;D6RHQ2;D6RFA9 | Ectonucleoside triphosphate diphosphohydrolase 1 | Entpd1 | 24.49874878 | 24.5354023 | 24.56092453 | 24.02132034 | 24.0872364 | 24.1331234 |
| **+** | 1.413128802 | 0.451871236 | P62274 | 40S ribosomal protein S29 | Rps29 | 25.71090698 | 25.73812485 | 25.71544456 | 25.40918159 | 25.42726326 | 24.97241783 |
| **+** | 1.865355986 | 0.452979406 | G3X8X7;Q920Q4;A2BI90 | Vacuolar protein sorting-associated protein 16 homolog | Vps16 | 22.67357445 | 22.4817791 | 22.72088051 | 22.32972717 | 22.07910347 | 22.10846519 |
| **+** | 3.906327655 | 0.454618454 | Q8BWT1;A0A494B9J2 | 3-ketoacyl-CoA thiolase, mitochondrial | Acaa2 | 25.91806984 | 25.97412491 | 25.98865128 | 25.5387516 | 25.46357346 | 25.5146656 |
| **+** | 1.213163865 | 0.459466934 | Q924Z4;D3Z4M2;D3YTM0;D3Z0Z2 | Ceramide synthase 2 | Cers2 | 24.32409286 | 24.19512939 | NaN | 23.72088051 | 23.65130043 | 24.02825165 |
| **+** | 2.984290194 | 0.461701075 | Q9DB29;A0A1Y7VLY5;A0A1Y7VKA0;A0A1Y7VKC6 | Isoamyl acetate-hydrolyzing esterase 1 homolog | Iah1 | 23.59536934 | 23.54073334 | 23.61605072 | 23.18826294 | 23.0260067 | 23.15278053 |
| **+** | 1.514733114 | 0.465752284 | A0A494B9X3;E9Q9M1;Q3V1L4;A0A494BBP6;G3X9J6;A0A494BBK9;A0A494BAU4;A0A494BAN2;A0A494BBI8;A0A494BBM7 | Cytosolic purine 5-nucleotidase | Nt5c2 | 22.9430275 | 23.03194046 | 23.23258591 | 22.45329857 | 22.53129196 | 22.82570648 |
| **+** | 2.882282799 | 0.467472712 | Q60648 | Ganglioside GM2 activator | Gm2a | 25.53635216 | 25.43990135 | 25.61248398 | 25.06064606 | 25.01077652 | 25.11489677 |
| **+** | 3.275079094 | 0.468077342 | Q60597;Z4YJV4;B2RXT3;E9Q7L0;Q5SVY0;Q5SVY1 | 2-oxoglutarate dehydrogenase, mitochondrial | Ogdh | 24.62878609 | 24.65354347 | 24.53237343 | 24.13014221 | 24.09273148 | 24.18759727 |
| **+** | 2.145833841 | 0.468315125 | Q5SQX6;F6QD74 | Cytoplasmic FMR1-interacting protein 2 | Cyfip2 | 23.14749527 | 23.14432526 | 23.3448925 | 22.7269001 | 22.64191818 | 22.86294937 |
| **+** | 2.384220356 | 0.468624433 | Q63810 | Calcineurin subunit B type 1 | Ppp3r1 | 23.42751884 | 23.34448624 | 23.27965927 | NaN | 22.85477448 | 22.90908623 |
| **+** | 1.68843735 | 0.470260938 | Q8BGB7 | Enolase-phosphatase E1 | Enoph1 | 22.87784958 | 22.63435936 | NaN | 22.23908424 | 22.25268936 | 22.36575699 |
| **+** | 1.728769279 | 0.471197764 | O35350;A0A494BAS0;A0A494BAC4 | Calpain-1 catalytic subunit | Capn1 | 23.5932045 | 23.43262482 | 23.24051094 | 22.92288589 | 22.84779739 | 23.08206367 |
| **+** | 3.667174736 | 0.474017461 | Q9CVB6;D3YXG6;A0A087WRT2 | Actin-related protein 2/3 complex subunit 2 | Arpc2 | 27.1300621 | 27.07439041 | 27.07510567 | 26.57614517 | 26.68204117 | 26.59931946 |
| **+** | 2.821938159 | 0.474642436 | Q91VD9;A0A087WQ77;A0A087WSU3 | NADH-ubiquinone oxidoreductase 75 kDa subunit, mitochondrial | Ndufs1 | 22.79125977 | 22.76506615 | 22.78719902 | 22.21554947 | 22.42201042 | 22.28203773 |
| **+** | 1.784821602 | 0.474672953 | Q9QYJ0 | DnaJ homolog subfamily A member 2 | Dnaja2 | 24.21556473 | 24.41693115 | 24.53023148 | 24.0413475 | 23.77856255 | 23.91879845 |
| **+** | 1.003653829 | 0.474708557 | A0A0N4SW73;Q8R361 | Rab11 family-interacting protein 5 | Rab11fip5 | 22.91883469 | 22.94225883 | 22.47027969 | 21.98213959 | 22.45726204 | 22.46784592 |
| **+** | 2.342995039 | 0.47532018 | A0A0A0MQ90;P97352 | Protein S100-A13 | S100a13 | 24.86159706 | 25.03900528 | 24.83045387 | 24.39008141 | 24.53712273 | 24.37789154 |
| **+** | 1.473196831 | 0.476273855 | P46062;E9Q0Y4;Q3V403 | Signal-induced proliferation-associated protein 1 | Sipa1 | 21.78304863 | 21.91012955 | 22.10440826 | 21.51277542 | 21.3997345 | NaN |
| **+** | 1.134843776 | 0.476558367 | Q9DC61;A2AIW9 | Mitochondrial-processing peptidase subunit alpha | Pmpca | 22.78016663 | 22.36418152 | 22.67895508 | 22.03795052 | NaN | 22.22446823 |
| **+** | 2.538305454 | 0.476594925 | P51863 | V-type proton ATPase subunit d 1 | Atp6v0d1 | 26.39907646 | 26.57229042 | 26.51600456 | 25.94411469 | 25.99263191 | 26.12084007 |
| **+** | 2.235667949 | 0.478162766 | Q9CQ80;A2A4J8;A8XY17;A2A4K0;E9PXS9 | Vacuolar protein-sorting-associated protein 25 | Vps25 | 23.62265587 | 23.71900177 | 23.5302906 | 23.2854557 | 23.09240723 | 23.05959702 |
| **+** | 0.946062756 | 0.478775024 | Q8K157;F6XWR4 | Aldose 1-epimerase | Galm | 23.18794441 | 22.83774757 | 23.25479507 | 22.72905922 | 22.2286129 | 22.88648987 |
| **+** | 2.154495323 | 0.482102712 | Q8K4Z3 | NAD(P)H-hydrate epimerase | Apoa1bp | 23.82074928 | 23.58118439 | 23.64625359 | 23.13458252 | 23.14286041 | 23.32443619 |
| **+** | 1.913618149 | 0.485401789 | Q8VC04;A2A4M9;A2A4N0 | Transmembrane protein 106A | Tmem106a | 24.64542961 | 24.5260582 | 24.26842308 | 23.97657394 | 23.9955883 | 24.01154327 |
| **+** | 0.955366041 | 0.486888568 | Q9Z1D1 | Eukaryotic translation initiation factor 3 subunit G | Eif3g | 24.03632545 | 24.24894524 | 24.28149986 | NaN | 23.96128654 | 23.44278336 |
| **+** | 2.109333925 | 0.487200419 | Q99JI4;A0A286YDW8 | 26S proteasome non-ATPase regulatory subunit 6 | Psmd6 | 24.96697235 | 24.79798126 | 24.91520119 | 24.23797607 | 24.47097588 | 24.50960159 |
| **+** | 1.257353427 | 0.488707542 | Q9R0A0 | Peroxisomal membrane protein PEX14 | Pex14 | 22.4970932 | NaN | 22.1318531 | 21.93459511 | 21.83368111 | 21.70902061 |
| **+** | 2.575072856 | 0.489690781 | Q9CRD2 | ER membrane protein complex subunit 2 | Emc2 | 22.9717598 | 23.174963 | 23.13671112 | 22.53741837 | 22.60354042 | 22.67340279 |
| **+** | 2.18167288 | 0.490700404 | Q8R1G5;L7N466 | 5-formyltetrahydrofolate cyclo-ligase | Mthfsl | 23.07934761 | 22.81975746 | 22.81995201 | 22.48787498 | 22.35702705 | 22.40205383 |
| **+** | 3.53346564 | 0.494515737 | O08749 | Dihydrolipoyl dehydrogenase, mitochondrial | Dld | 24.93490028 | 24.90550041 | 24.8451767 | 24.4655056 | 24.36672974 | 24.36979485 |
| **+** | 1.894430232 | 0.494626363 | P48962;Q3V132 | ADP/ATP translocase 1 | Slc25a4 | 25.73719597 | 25.91729736 | 25.92919922 | 25.18729591 | 25.52109146 | 25.39142609 |
| **+** | 2.22294844 | 0.498603821 | A0A0J9YUM4;A0A0J9YU62;O88712;A0A0J9YUR5;A0A0J9YVI3;A0A0J9YU66;A0A0J9YTW3;A0A0J9YVC3 | C-terminal-binding protein 1 | Ctbp1 | 23.46849251 | 23.43135071 | 23.24218559 | 22.80177498 | 22.84015083 | 23.00429153 |
| **+** | 4.378705753 | 0.49881045 | P67778;Q5SQG5 | Prohibitin | Phb | 25.7759037 | 25.80766869 | 25.78206825 | 25.30155754 | 25.32381821 | 25.24383354 |
| **+** | 4.635955713 | 0.499132156 | Q9CR62;Q5SX46;Q5SX48 | Mitochondrial 2-oxoglutarate/malate carrier protein | Slc25a11 | 25.65250397 | 25.71717072 | 25.67763901 | 25.19332504 | 25.15988731 | 25.19670486 |
| **+** | 1.847749977 | 0.49924914 | Q7TNS2 | MICOS complex subunit Mic10 | Minos1 | 24.614254 | 24.37185669 | 24.76693726 | 24.15437317 | 24.04676819 | 24.05415916 |
| **+** | 1.636072803 | 0.500125249 | F6W4D3;D3YY36;Q9DBD0 | Inhibitor of carbonic anhydrase | 1300017J02Rik;Ica | 25.23355865 | 24.99330139 | 25.27710724 | 24.69901085 | 24.63671684 | NaN |
| **+** | 2.445245461 | 0.503745397 | Q99K70;B1AWT2;B1AWT3;Q7TT45;B1AWT4 | Ras-related GTP-binding protein C;Ras-related GTP-binding protein D | Rragc;Rragd | 24.09635544 | 23.90306664 | 24.17942047 | 23.54191399 | 23.56104088 | 23.56465149 |
| **+** | 1.252533997 | 0.504886309 | Q8R1V4;Q5SVW9 | Transmembrane emp24 domain-containing protein 4 | Tmed4 | 23.52276993 | 23.95571136 | 23.75052071 | NaN | 23.29080772 | 23.18542099 |
| **+** | 1.586088822 | 0.506790161 | P56387 | Dynein light chain Tctex-type 3 | Dynlt3 | 23.88799667 | NaN | 23.72150612 | 23.29768181 | NaN | 23.29824066 |
| **+** | 1.554908806 | 0.51050059 | Q6P3A9 | ADP-ribosylation factor-like protein 11 | Arl11 | 23.76273727 | 23.62097931 | 23.3998909 | 23.30005836 | 22.96481514 | 22.98723221 |
| **+** | 2.739556333 | 0.512887637 | Q9Z2X2;A2AG83 | 26S proteasome non-ATPase regulatory subunit 10 | Psmd10 | 22.5314579 | 22.43776512 | 22.45080757 | 22.00147629 | 21.9194355 | NaN |
| **+** | 2.423735265 | 0.513570786 | Q99LC3;A0A087WR38 | NADH dehydrogenase [ubiquinone] 1 alpha subcomplex subunit 10, mitochondrial | Ndufa10 | 22.77070236 | 22.79934692 | 22.88639832 | 22.1888504 | 22.27491951 | 22.45196533 |
| **+** | 3.320088206 | 0.519882838 | A0A0R4J083;P51174 | Long-chain specific acyl-CoA dehydrogenase, mitochondrial | Acadl | 25.64105225 | 25.59934807 | 25.51132202 | 25.11791039 | 25.00710106 | 25.06706238 |
| **+** | 1.281881479 | 0.520532608 | P97315 | Cysteine and glycine-rich protein 1 | Csrp1 | 24.75327873 | 24.97254944 | 25.03603172 | 24.71145821 | 24.12581253 | 24.36299133 |
| **+** | 2.681446996 | 0.520563126 | Q9CPU4 | Microsomal glutathione S-transferase 3 | Mgst3 | 25.75926018 | 25.99703407 | 25.83749771 | 25.3858757 | 25.33514214 | 25.31108475 |
| **+** | 2.118316906 | 0.523495356 | A0A2R8VI07;Q8C6B0;H3BJI7;A0A2R8VK72;Q9D7S5;Q76I24;Q5I0W6;Q76I26;A0A2R8VHN2;G3X9G9 |  | Mettl7a1;Methig1;mCG_20149;UbiE2;Mettl7a2 | 23.54615974 | 23.85471725 | 23.65053368 | 23.17888832 | 23.05989456 | 23.24214172 |
| **+** | 2.043321364 | 0.524702708 | O08795;A0A1L1ST83 | Glucosidase 2 subunit beta | Prkcsh | 24.94908905 | 25.06960487 | 24.86135864 | 24.44474411 | 24.26999283 | 24.5912075 |
| **+** | 1.166673911 | 0.524877548 | P70202 | Latexin | Lxn | 24.95779228 | 24.35796738 | 24.38053513 | 24.14289284 | 24.09337616 | 23.88539314 |
| **+** | 1.326897302 | 0.525502841 | Q80SY3 | V-type proton ATPase subunit d 2 | Atp6v0d2 | 22.91203308 | 23.12015915 | 23.30744171 | 22.50035477 | NaN | 22.67506218 |
| **+** | 2.068583187 | 0.532663345 | Q8VE96;A0A0G2JEG3;D6RHL1 | Solute carrier family 35 member F6 | Slc35f6 | 22.86296844 | 23.1305809 | 22.91340256 | 22.28850174 | 22.50193405 | 22.51852608 |
| **+** | 1.434769177 | 0.533889771 | Q99JX4;A2A702;A2A701 | Eukaryotic translation initiation factor 3 subunit M | Eif3m | 24.81122589 | 24.67804337 | 24.24575806 | 24.04226494 | 24.09498596 | 23.9961071 |
| **+** | 2.868953931 | 0.541412354 | A0A1B0GR60;Q9CPX4;P29391;P49945;A0A1Y7VNT9;A0A1B0GRH4 | Ferritin;Ferritin light chain 1 | Ftl1 | 25.92892838 | 25.93012428 | 25.90150261 | 25.37706375 | 25.49637413 | 25.26288033 |
| **+** | 1.58238892 | 0.54572169 | Q80X95;Q6NTA4;G3UYP1 | Ras-related GTP-binding protein A;Ras-related GTP-binding protein B | Rraga;Rragb | 23.6115551 | 23.86372375 | 23.6028614 | 23.04773331 | NaN | 23.24625015 |
| **+** | 1.794413281 | 0.552171707 | Q9JKB1;A0A2I3BQ39;P58321 | Ubiquitin carboxyl-terminal hydrolase isozyme L3;Ubiquitin carboxyl-terminal hydrolase isozyme L4 | Uchl3;Uchl4 | 24.43013763 | 24.74755287 | 24.75475693 | 24.05556488 | 23.96305275 | 24.25731468 |
| **+** | 1.061153376 | 0.553858121 | Q3UMA3;Q99LI8;B1ATY9;F6VV02 | Hepatocyte growth factor-regulated tyrosine kinase substrate | Hgs | 23.4434166 | 23.24890137 | 23.82347298 | NaN | 22.90475655 | 22.9980545 |
| **+** | 3.407966479 | 0.559560458 | Q62095 | ATP-dependent RNA helicase DDX3Y | Ddx3y | 23.11083984 | 23.07603836 | 23.15279579 | 22.5714035 | 22.53592491 | NaN |
| **+** | 3.143907111 | 0.559800466 | P09528;A0A494BA92;A0A494B9D4;A0A494BAP3 | Ferritin heavy chain;Ferritin heavy chain, N-terminally processed | Fth1 | 26.01039124 | 26.1908226 | 26.02290916 | 25.4913044 | 25.5368557 | 25.51656151 |
| **+** | 4.580797179 | 0.560180028 | P10639 | Thioredoxin | Txn | 28.11058235 | 28.08024788 | 28.13609123 | 27.5262661 | 27.58870888 | 27.5314064 |
| **+** | 1.637166889 | 0.563168844 | Q3ULB1;Q921W7;P47226;D6RH72;B1AXB9 | Testin | Tes | 23.63156509 | 23.85804176 | 23.68260384 | 23.38837433 | 23.19242287 | 22.90190697 |
| **+** | 2.442935076 | 0.563379606 | Q6SJQ0 | CMRF35-like molecule 8 | Cd300a | 22.90598869 | 23.03545189 | 22.96865273 | 22.34332085 | 22.46998215 | NaN |
| **+** | 3.162392311 | 0.563394547 | Q9Z1G3;A0A2I3BPD0;A0A2I3BQD9 | V-type proton ATPase subunit C 1 | Atp6v1c1 | 25.70777702 | 25.52984428 | 25.6201973 | 24.99821663 | 25.07189751 | 25.09752083 |
| **+** | 2.839465766 | 0.564737956 | P62331 | ADP-ribosylation factor 6 | Arf6 | 25.12656212 | 24.92596817 | 24.91966248 | 24.3767662 | 24.44903374 | 24.45217896 |
| **+** | 2.49961162 | 0.571092606 | O54890 | Integrin beta-3 | Itgb3 | 22.68367577 | 22.75029564 | 22.97938538 | 22.21475029 | 22.24086189 | 22.24446678 |
| **+** | 3.195949531 | 0.572309494 | Q99P91;Q8BVA0 | Transmembrane glycoprotein NMB | Gpnmb | 31.07399559 | 31.03839302 | 30.93220329 | 30.50975227 | 30.36828041 | 30.44963074 |
| **+** | 2.260566355 | 0.574447632 | G3X8U3 | Queuosine 5'-phosphate N-glycosylase/hydrolase | 2210016F16Rik | 22.98757935 | 22.90703392 | 23.04620171 | 22.55931473 | 22.22555733 | 22.43260002 |
| **+** | 3.835986951 | 0.577616374 | Q61838 | Alpha-2-macroglobulin;Alpha-2-macroglobulin 165 kDa subunit;Alpha-2-macroglobulin 35 kDa subunit | A2m | 27.54303551 | 27.62470627 | 27.66444778 | 27.05299759 | 27.05299759 | 26.99334526 |
| **+** | 1.108480543 | 0.579236984 | P35282;A0A1W2P6Z4 | Ras-related protein Rab-21 | Rab21 | 24.6390934 | 24.8411026 | 24.94766426 | 24.52282906 | 23.77986526 | 24.38745499 |
| **+** | 2.499178706 | 0.590646744 | P26883;F6X9I3 | Peptidyl-prolyl cis-trans isomerase FKBP1A | Fkbp1a | 25.96166229 | 25.76002312 | 25.98702431 | 25.33949852 | 25.19861794 | 25.39865303 |
| **+** | 2.370533115 | 0.595102946 | Q9D1P4;A0A1L1STZ1;A0A1L1SU22;A0A1L1STQ1;A0A1L1SSG7 | Cysteine and histidine-rich domain-containing protein 1 | Chordc1 | 23.39335823 | 23.53124428 | 23.49191666 | 22.73947906 | 22.83720779 | 23.05452347 |
| **+** | 3.183303992 | 0.601727168 | Q6ZWZ7;Q9CPR4;B2RY53 | 60S ribosomal protein L17 | Rpl17 | 25.87499809 | 25.87354279 | 25.77127647 | 25.21486092 | 25.33831024 | 25.16146469 |
| **+** | 1.86942363 | 0.602474213 | A0A087WR20;P49935 | Pro-cathepsin H;Cathepsin H mini chain;Cathepsin H;Cathepsin H heavy chain;Cathepsin H light chain | Ctsh | 23.52599716 | 23.77384377 | 23.62354851 | 23.26284409 | 23.01568413 | 22.83743858 |
| **+** | 3.18595733 | 0.605292002 | A0A0R4J092;Q8K2I4;D6RGR1 | Beta-mannosidase | Manba | 22.66550827 | 22.70096016 | 22.73365784 | 22.02658081 | 22.04386902 | 22.21380043 |
| **+** | 3.125994309 | 0.608503977 | O55142 | 60S ribosomal protein L35a | Rpl35a | 25.89094543 | 26.00328827 | 25.78917694 | 25.31395912 | 25.24325371 | 25.30068588 |
| **+** | 1.680520672 | 0.613051732 | Q9CQR4 | Acyl-coenzyme A thioesterase 13;Acyl-coenzyme A thioesterase 13, N-terminally processed | Acot13 | 25.54683685 | 25.17214966 | 25.03330231 | 24.65654564 | 24.52055359 | 24.73603439 |
| **+** | 4.192227902 | 0.617268244 | Q8VEM8;G5E902 | Phosphate carrier protein, mitochondrial | Slc25a3 | 28.02353287 | 27.99490929 | 28.0980835 | 27.38632011 | 27.43571281 | 27.44268799 |
| **+** | 1.392319796 | 0.617424647 | Q9DB15 | 39S ribosomal protein L12, mitochondrial | Mrpl12 | 24.34590721 | 24.69736862 | 24.82313347 | 23.70577621 | 24.14359283 | 24.16476631 |
| **+** | 2.142773513 | 0.620528539 | Q04750 | DNA topoisomerase 1 | Top1 | 22.86996651 | 23.22152138 | 22.86364937 | 22.31791115 | 22.42782593 | 22.34781456 |
| **+** | 2.46611208 | 0.62165451 | Q8BWY3 | Eukaryotic peptide chain release factor subunit 1 | Etf1 | 24.55566978 | 24.50893784 | 24.55145073 | 23.93840599 | 24.07675743 | 23.7359314 |
| **+** | 4.461211264 | 0.628774007 | Q3UGC7;Q66JS6 | Eukaryotic translation initiation factor 3 subunit J-A;Eukaryotic translation initiation factor 3 subunit J-B | Eif3j1;Eif3j2 | 23.95712852 | 23.87998199 | 23.90976334 | 23.31492805 | 23.24514771 | 23.30047607 |
| **+** | 2.652831069 | 0.63035965 | Q9JKW0;A0A0U1RPY6 | ADP-ribosylation factor-like protein 6-interacting protein 1 | Arl6ip1 | 24.79793167 | 24.95304871 | 24.90044212 | 24.18412018 | 24.16691208 | 24.40931129 |
| **+** | 2.714882825 | 0.632105509 | Q9CY27;G3UWE1;A0A5F8MQC8;Q52L67;A0A5F8MPU6;A0A1D5RLB4;Q3TAN8;A0A1D5RLH3 | Very-long-chain enoyl-CoA reductase | Tecr | 24.46201324 | 24.41196251 | 24.56337166 | 23.99541664 | 23.79223251 | 23.75338173 |
| **+** | 3.072288461 | 0.632675807 | Q9JI75;A0A1Y7VMA0;A0A1Y7VL95 | Ribosyldihydronicotinamide dehydrogenase [quinone] | Nqo2 | 23.4314785 | 23.40249634 | 23.47047997 | 22.84278107 | 22.89339638 | 22.67024994 |
| **+** | 2.645669714 | 0.636112213 | Q9DB73;G3UZG6;G3UXV8;G3UZ57 | NADH-cytochrome b5 reductase 1 | Cyb5r1 | 24.24554062 | 24.15390968 | 24.34448624 | 23.51797295 | 23.56139183 | 23.75623512 |
| **+** | 2.194624536 | 0.638161341 | A0A0R4J069;Q9JLI6;F7D651;F6ZFP8;D3Z7G6;F7BB44 | Selenocysteine lyase | Scly | 23.82289124 | 24.09240723 | 24.02960014 | 23.16784668 | 23.39047623 | 23.47209167 |
| **+** | 1.385587649 | 0.638519287 | Q9R059;F2Z455;D6RJ68 | Four and a half LIM domains protein 3 | Fhl3 | 23.18663025 | 23.22488022 | 23.06798935 | 22.52396584 | 22.15668869 | 22.88328743 |
| **+** | 3.988053703 | 0.651196798 | Q9CQW2;A0A0N4SVB8 | ADP-ribosylation factor-like protein 8B | Arl8b | 27.07479858 | 27.104496 | 26.99539566 | 26.35388565 | 26.43985367 | 26.42736053 |
| **+** | 3.583810559 | 0.660370509 | Q62348 | Translin | Tsn | 24.34435081 | 24.4184761 | 24.37052727 | 23.61840439 | 23.75969315 | 23.77414513 |
| **+** | 2.761321903 | 0.667133967 | A0A494BA97;Q62422 | Osteoclast-stimulating factor 1 | Ostf1 | 27.51420021 | 27.63747787 | 27.40689659 | 26.92116165 | 26.90276718 | 26.73324394 |
| **+** | 1.38791687 | 0.675608953 | Q9D1K2;A0A0N4SVE1;F7B2B4 | V-type proton ATPase subunit F | Atp6v1f | 24.64784622 | 25.40999031 | 25.14802361 | 24.41892624 | 24.44524956 | 24.31485748 |
| **+** | 1.845759483 | 0.680465698 | Q9QZ23;A0A0N4SUH8;D3Z285 | NFU1 iron-sulfur cluster scaffold homolog, mitochondrial | Nfu1 | 23.57727051 | 23.32099724 | 23.60997772 | 22.70800972 | 23.09350395 | 22.6653347 |
| **+** | 2.753744937 | 0.693761826 | P42125;A0A3Q4EC00;A0A452J8A5 | Enoyl-CoA delta isomerase 1, mitochondrial | Eci1 | 23.20386124 | 23.17264557 | 23.40431595 | 22.46016502 | 22.66392517 | 22.57544708 |
| **+** | 2.400322575 | 0.698513031 | A0A087WPT7;Q6P549;A0A1B0GR64;A0A1B0GST7 | Phosphatidylinositol 3,4,5-trisphosphate 5-phosphatase 2 | Inppl1 | 23.77293777 | 24.16875076 | 24.00667381 | 23.32937241 | 23.27084923 | 23.25260162 |
| **+** | 1.526675374 | 0.71690623 | P12265;Q99KJ6;D3YY48 | Beta-glucuronidase | Gusb | 23.22156525 | 23.1580162 | 23.16896439 | 22.05006027 | 22.57329941 | 22.77446747 |
| **+** | 2.700262551 | 0.718930562 | Q9R0X4;Q32MW3 | Acyl-coenzyme A thioesterase 9, mitochondrial;Acyl-coenzyme A thioesterase 10, mitochondrial | Acot9;Acot10 | 23.84823608 | 23.7743454 | 23.67927742 | 23.21751785 | 23.00187111 | 22.92567825 |
| **+** | 2.340926499 | 0.732186317 | F7D5L2;Q99J09 | Methylosome protein 50 | Wdr77 | 22.68965912 | 22.72978592 | 22.89221001 | 21.96912575 | 22.10760498 | NaN |
| **+** | 2.276871468 | 0.736782074 | Q99L13 | 3-hydroxyisobutyrate dehydrogenase, mitochondrial | Hibadh | 23.90058136 | 23.56720734 | 23.74611855 | 23.18647957 | 22.90459251 | 22.91248894 |
| **+** | 2.289825572 | 0.762166977 | P97821 | Dipeptidyl peptidase 1;Dipeptidyl peptidase 1 exclusion domain chain;Dipeptidyl peptidase 1 heavy chain;Dipeptidyl peptidase 1 light chain | Ctsc | 23.04320145 | 22.90069199 | 23.16657639 | 22.23645973 | 22.31285286 | NaN |
| **+** | 2.493580033 | 0.76828448 | G3UZ30;A5A4Y9;Q8K1L5 | Protein phosphatase 1 regulatory subunit 11 | Ppp1r11 | 22.75664139 | 22.64167595 | 22.72167397 | 22.09358597 | 21.70973587 | 22.01181602 |
| **+** | 2.244909172 | 0.771518071 | D3YWT0;D3Z569;D3YTS1;Q9R0P6 | Signal peptidase complex catalytic subunit SEC11;Signal peptidase complex catalytic subunit SEC11A | Sec11a | 24.56743813 | 24.75281906 | 24.31319809 | 23.66003418 | 23.7751503 | 23.88371658 |
| **+** | 2.909911292 | 0.772278468 | Q00519;A0A3B2WBF8;A0A3B2W454;A0A3B2WDA8 | Xanthine dehydrogenase/oxidase;Xanthine dehydrogenase;Xanthine oxidase | Xdh | 22.83549309 | 22.81362534 | 22.97887993 | 22.07378578 | 22.25349617 | 21.983881 |
| **+** | 3.12094604 | 0.776383082 | P68254 | 14-3-3 protein theta | Ywhaq | 26.44259453 | 26.49180984 | 26.55673599 | 25.75169563 | 25.5743866 | 25.83590889 |
| **+** | 1.955174759 | 0.787274043 | Q8CGB9;Q9JHR7 | Insulin-degrading enzyme | Ide | 26.40608597 | 26.33488655 | 26.51086998 | 25.89578247 | 25.6768589 | 25.317379 |
| **+** | 1.726279411 | 0.788596153 | Q9CQU3 | Protein RER1 | Rer1 | 23.95295906 | 24.27724838 | 23.95562172 | 23.40392685 | NaN | 23.14276695 |
| **+** | 1.587147727 | 0.791508357 | P56394 | Cytochrome c oxidase copper chaperone | Cox17 | 23.99248123 | 24.08180237 | 24.26563644 | 23.53848457 | 23.10511208 | NaN |
| **+** | 2.893735345 | 0.79611969 | A0A0R4J138;P50429 | Arylsulfatase B | Arsb | 24.23447227 | 24.04510307 | 24.19046402 | 23.27795792 | 23.52109146 | 23.28263092 |
| **+** | 3.439172642 | 0.813527743 | Q569Z6;Q8BZN7;F6YSQ2;F6YH92 | Thyroid hormone receptor-associated protein 3 | Thrap3 | 23.41312599 | 23.4284153 | 23.40833855 | 22.54394531 | 22.66225243 | NaN |
| **+** | 3.360545097 | 0.827065786 | P34884 | Macrophage migration inhibitory factor | Mif | 29.09607315 | 29.28550911 | 29.03614044 | 28.31838036 | 28.34117126 | 28.27697372 |
| **+** | 0.83265712 | 0.839071274 | Q9CQV6;M0QWC2;Q91VR7 | Microtubule-associated proteins 1A/1B light chain 3B;Microtubule-associated proteins 1A/1B light chain 3A | Map1lc3b;Map1lc3a | NaN | 25.01941299 | 24.55952644 | NaN | 23.66947174 | 24.23132515 |
| **+** | 0.827624329 | 0.886188189 | Q9CW03 | Structural maintenance of chromosomes protein 3 | Smc3 | 29.01552582 | 29.10321999 | 29.02104568 | 28.77350998 | 27.54730797 | NaN |
| **+** | 2.04317986 | 0.89104112 | P61971 | Nuclear transport factor 2 | Nutf2 | 25.31558418 | 25.47252655 | 25.33449554 | NaN | 24.66117477 | 24.30514717 |
| **+** | 3.576908256 | 0.912562052 | Q9EQ32 | Phosphoinositide 3-kinase adapter protein 1 | Pik3ap1 | 22.79247093 | 22.75961113 | 22.98394966 | 21.94241905 | 21.97515869 | 21.88076782 |
| **+** | 2.262279471 | 0.922494253 | Q99L43;A2AMQ5;Q6PBC0;F6S4G2 | Phosphatidate cytidylyltransferase 2;Phosphatidate cytidylyltransferase | Cds2 | 23.75460434 | 23.73169136 | 23.93517113 | 23.05596352 | 22.57279015 | 23.02523041 |
| **+** | 2.784196905 | 0.924647649 | Q9CPU0;A0A494BAF7;A0A494BBE7;A0A494B970 | Lactoylglutathione lyase | Glo1 | 24.22479248 | 24.36031342 | 23.95907593 | 23.31851768 | 23.24580193 | 23.20591927 |
| **+** | 1.943132631 | 0.925561587 | Q71FD7;A2ADE7;A2ADF3;A2ADF2;A2ADF1;A2ADE8;A2ADE9;A2ADE5;A2ADE6 | Filamin-binding LIM protein 1 | Fblim1 | 23.27568626 | 23.61279297 | 23.27511787 | NaN | 22.57043076 | 22.35417747 |
| **+** | 2.92183138 | 0.925601959 | P97370 | Sodium/potassium-transporting ATPase subunit beta-3 | Atp1b3 | 25.79335022 | 25.54819107 | 25.73008537 | 24.83764076 | 24.5946846 | 24.86249542 |
|  | 0.784786056 | -0.560944557 | Q9JLJ5;Q4V9V3 | Elongation of very long chain fatty acids protein 1;Elongation of very long chain fatty acids protein | Elovl1 | NaN | 21.51753998 | 22.03848648 | 22.33565331 | NaN | 22.34226227 |
|  | 0.506035374 | -0.560905457 | P10833;A0A1B0GRG1;A0A1B0GRT5;P62071 | Ras-related protein R-Ras | Rras | NaN | 22.63076401 | 22.59784889 | 22.75733376 | 23.59309006 | NaN |
|  | 0.614945614 | -0.467921257 | O70591;F8WJ30 | Prefoldin subunit 2 | Pfdn2 | NaN | 22.79020691 | 23.57103157 | 23.45287132 | 23.82425117 | 23.66849899 |
|  | 0.79433989 | -0.35227108 | F6TCV0;D3YX27;Q9JIY5;D3YX28;F6XUR8;S4R1B3;S4R1A8;A2RT60 | Serine protease HTRA2, mitochondrial | Htra2 | NaN | 22.5866909 | 22.90382004 | 23.06812096 | NaN | 23.12693214 |
|  | 0.542104808 | -0.234588623 | Q8JZZ5;P53811 | Phosphatidylinositol transfer protein beta isoform | Pitpnb | NaN | 23.38007355 | 23.38692856 | 23.85490799 | 23.63123131 | 23.36812973 |
|  | 0.260878507 | -0.190238953 | Q9Z2H6;A0A0N4SVJ7 | C-type lectin domain family 4 member D | Clec4d | NaN | 21.7177887 | 21.68701172 | NaN | 21.62729454 | 22.15798378 |
|  | 0.290017518 | -0.167970657 | G3UVU2;D3YW09;Q62203;D3Z5A6 | Splicing factor 3A subunit 2 | Sf3a2 | NaN | 22.54410934 | 22.15316772 | 22.60102654 | 22.43219185 | NaN |
|  | 0.2514551 | -0.117963791 | Q00623 | Apolipoprotein A-I;Proapolipoprotein A-I;Truncated apolipoprotein A-I | Apoa1 | NaN | 25.74824524 | 26.01718521 | 26.10543633 | 25.89592171 | NaN |
|  | 0.268227979 | -0.084545135 | Q9JKN1 | Zinc transporter 7 | Slc30a7 | NaN | 23.15849304 | 22.96924973 | NaN | 23.21403885 | 23.08279419 |
|  | 0.154903574 | -0.080717087 | Q99P65;D6RI95;D3YWS9 | Equilibrative nucleoside transporter 3 | Slc29a3 | NaN | 22.30435371 | 22.54229164 | 22.3678112 | 22.37954521 | 22.76476288 |
|  | 0.434247437 | -0.072831154 | Q3V3R1 | Monofunctional C1-tetrahydrofolate synthase, mitochondrial | Mthfd1l | NaN | 22.94997215 | 22.8493824 | NaN | 23.01067352 | 22.93434334 |
|  | 0.17862353 | 0.059825261 | Q9WVA2;Q4FZG7 | Mitochondrial import inner membrane translocase subunit Tim8 A | Timm8a1 | NaN | 22.72561073 | 23.00329781 | 22.83730507 | 22.70081139 | 22.87577057 |
|  | 0.18070241 | 0.081178665 | E9QJT5;P56376;Q8BMV3 | Acylphosphatase;Acylphosphatase-1 | Acyp1 | NaN | 22.32396889 | 22.43478966 | 22.05897141 | 22.48123741 | 22.35439301 |
|  | 0.219706645 | 0.095790863 | A0A1B0GT56;P51829 | Adenylate cyclase type 7 | Adcy7 | NaN | 21.26807976 | 21.34851646 | 21.06118202 | 21.36383247 | NaN |
|  | 0.131342283 | 0.100709597 | A0A2R8VHP3 | Predicted pseudogene 5478 | Gm5478 | NaN | 24.32991982 | 24.14957428 | 24.55168533 | 23.96199226 | 23.90343475 |
|  | 0.107036096 | 0.101793607 | P14069 | Protein S100-A6 | S100a6 | NaN | 29.35514641 | 28.53715897 | 28.99112892 | 28.6313839 | 28.91056442 |
|  | 0.289404016 | 0.129638672 | Q9DBL1;E9Q5L3;A0A140LHL4 | Short/branched chain specific acyl-CoA dehydrogenase, mitochondrial | Acadsb | NaN | 23.43822098 | 23.11265564 | 23.17053795 | 23.12106133 | NaN |
|  | 1.166009769 | 0.130148888 | Q9ES56;Q3V493 | Trafficking protein particle complex subunit 4 | Trappc4 | NaN | 22.6383419 | 22.68183136 | 22.5584507 | NaN | 22.50142479 |
|  | 0.695393538 | 0.143396378 | Q9D0J4 | ADP-ribosylation factor-like protein 2 | Arl2 | NaN | 22.44929695 | 22.58403206 | 22.31506538 | 22.48517418 | 22.31956482 |
|  | 0.539447413 | 0.152099609 | Q8BKS9;A0A0N4SUH4;A0A494BBI7 | Pumilio domain-containing protein KIAA0020 | Kiaa0020 | NaN | 21.18107414 | 21.38258171 | NaN | 21.09584045 | 21.16361618 |
|  | 0.790973808 | 0.17241319 | Q9D967 | Magnesium-dependent phosphatase 1 | Mdp1 | NaN | 22.32429886 | 22.2116642 | 22.07404709 | 21.99607277 | 22.21658516 |
|  | 1.19595038 | 0.23049736 | A0A023T778;Q9CQL1;P61327;G3UZW7 | Protein mago nashi homolog 2;Protein mago nashi homolog | Magohb;Magoh | NaN | 23.83499336 | 23.85965538 | NaN | 23.67669868 | 23.55695534 |
|  | 0.928781861 | 0.235602379 | Q9D7M1 | Glucose-induced degradation protein 8 homolog | Gid8 | NaN | 22.19355011 | 22.28717613 | 22.08037186 | NaN | 21.92914963 |
|  | 0 | 0.267669678 | A0A0U1RQA5;A0A0U1RP20;A0A0U1RQA0;O35316 | Sodium- and chloride-dependent taurine transporter | Slc6a6 | NaN | 24.20149994 | 24.34204483 | NaN | 24.00410271 | NaN |
|  | 1.185861142 | -0.118985494 | P60710;E9Q1F2;E9Q5F4;G3UZ07;F8WGM8;E9Q2D1;F6WX90;A0A0U1RQ96;E9Q3M9 | Actin, cytoplasmic 1;Actin, cytoplasmic 1, N-terminally processed | Actb | 33.0141983 | 32.99270248 | 32.93803406 | 33.01866531 | 33.15132523 | 33.13190079 |
|  | 0.497829868 | -0.785469055 | A0A0U1RNK7;E9PX48;A2A9M5;A2A9M4;Q8R1A4;A0A0U1RNY4;A2AW79;F6ZJ55 | Dedicator of cytokinesis protein 7 | Dock7 | 31.99283791 | 30.52654648 | 32.09860992 | NaN | 32.39593506 | 32.25366592 |
|  | 0.291741 | -0.110626856 | P07724 | Serum albumin | Alb | 31.81955147 | 32.09326553 | 31.75704384 | 31.88758087 | 31.88656425 | 32.22759628 |
|  | 1.162128678 | -0.27153333 | Q6ZQ06 | Centrosomal protein of 162 kDa | Cep162 | 31.57384682 | 31.56074333 | 31.34509087 | 31.92609787 | 31.67025185 | 31.69793129 |
|  | 2.184801325 | 0.122615814 | Q8C253;P16110 | Galectin;Galectin-3 | Lgals3 | 31.34069443 | 31.35757065 | 31.28267288 | 31.19137001 | 31.21013451 | 31.211586 |
|  | 2.394230633 | 0.138118108 | E9PZ00;Q8BFQ1;K3W4L3;J3QPG5;Q61207 | Prosaposin | Psap | 31.19454384 | 31.16602707 | 31.18960381 | 31.06291199 | 31.07048035 | 31.00242805 |
|  | 0.396776539 | 0.115212758 | P68134;P68033;P63268;P62737;A0A1D5RM20;D3Z2K3;A0A494B9T3;D3YZY0 | Actin, alpha skeletal muscle;Actin, alpha cardiac muscle 1;Actin, gamma-enteric smooth muscle;Actin, aortic smooth muscle | Acta1;Actc1;Actg2;Acta2 | 31.13687325 | 31.18783569 | 31.14108276 | 30.82373238 | 31.2446785 | 31.05174255 |
|  | 2.05837194 | 0.117672602 | P10126;D3YZ68;D3Z3I8 | Elongation factor 1-alpha 1 | Eef1a1 | 30.91876221 | 30.85942078 | 30.87927818 | 30.79079247 | 30.77956581 | 30.73408508 |
|  | 1.090170503 | -0.044099808 | A0A0A0MQF6;P16858;S4R257;A0A1D5RLD8;S4R1W1;S4R1W8;A0A0R4J0X7;S4R2G5;Q64467;S4R1N5 | Glyceraldehyde-3-phosphate dehydrogenase | Gapdh;Gm3839 | 30.89301872 | 30.91812134 | 30.88634682 | 30.92238235 | 30.97555351 | 30.93185043 |
|  | 2.055783207 | 0.164131165 | P20152;A0A0A6YWC8;A2AKJ2;P31001;G5E846;A0A2R8W6R6;G3X981;P15331;D3YZ35;A0A0R4J036;P46660;P08553;P08551 | Vimentin | Vim | 30.64426804 | 30.71522141 | 30.66805458 | 30.47072792 | 30.56347275 | 30.50094986 |
|  | 1.825140279 | 0.134710948 | P16045;A0A2R8VHJ0 | Galectin-1 | Lgals1 | 30.47798157 | 30.54176331 | 30.47585678 | 30.40730286 | 30.32130814 | 30.36285782 |
|  | 0.039636102 | 0.065127055 | Q8BFZ3 | Beta-actin-like protein 2 | Actbl2 | 30.28812981 | 28.36385918 | 29.32023239 | 29.3696537 | 29.22740555 | 29.17978096 |
|  | 0.1467651 | -0.427829742 | Q9D125 | 28S ribosomal protein S25, mitochondrial | Mrps25 | 30.28140259 | 30.4674263 | 29.90382957 | 32.76424026 | 29.31029701 | 29.86161041 |
|  | 1.414072098 | -0.125260035 | P52480;A0A1L1SU37;A0A1L1SQV8;A0A1L1SSN6;A0A1L1STV8;A0A1L1SUV0;A0A1L1ST52;A0A1L1SVH2;E9Q509;G3X925;P53657 | Pyruvate kinase PKM | Pkm | 30.05348969 | 30.01025391 | 29.96071815 | 30.10299492 | 30.19612885 | 30.10111809 |
|  | 0.11746325 | -0.016378403 | P17742;A0A1L1SST0;A0A1L1SRX5 | Peptidyl-prolyl cis-trans isomerase A;Peptidyl-prolyl cis-trans isomerase A, N-terminally processed | Ppia | 30.00918579 | 30.13711739 | 29.9971199 | 30.03393745 | 30.11122894 | 30.04739189 |
|  | 0.87959561 | -0.490564982 | A0A1W2P768;P84228;F8WI35;A0A8I4SYN6 | Histone H3.2;Histone H3 | Hist1h3b;H3f3a | 29.66788673 | 28.89052773 | 29.60766602 | 29.74331284 | 29.90856171 | 29.98590088 |
|  | 0.378908234 | -0.060571671 | Q6GQT1;A0A0N4SUX6 | Alpha-2-macroglobulin-P | A2mp | 29.64452553 | 29.86164093 | 29.80882263 | 29.8482399 | 29.84674644 | 29.80171776 |
|  | 1.362661472 | -0.099785487 | P05064;D3YWI1;D3Z510;A0A0U1RPN8 | Fructose-bisphosphate aldolase A;Fructose-bisphosphate aldolase | Aldoa | 29.48316193 | 29.49598503 | 29.52583694 | 29.59853745 | 29.65790558 | 29.54789734 |
|  | 1.715628535 | 0.164223989 | Q62426 | Cystatin-B | Cstb | 29.38521004 | 29.4705143 | 29.45244217 | 29.23740196 | 29.23690033 | 29.34119225 |
|  | 1.673444417 | 0.195267359 | P10605 | Cathepsin B;Cathepsin B light chain;Cathepsin B heavy chain | Ctsb | 29.25502777 | 29.17693138 | 29.25743675 | 28.94335556 | 29.09088516 | 29.0693531 |
|  | 3.309583673 | -0.151911418 | P18760;F8WGL3;A0A494B9A7 | Cofilin-1 | Cfl1 | 29.15723419 | 29.14882278 | 29.17297935 | 29.29180145 | 29.30730247 | 29.33566666 |
|  | 1.014101021 | 0.079421997 | P68373;Q9JJZ2 | Tubulin alpha-1C chain | Tuba1c | 29.09717941 | 29.09866142 | 29.01648331 | 28.94879532 | 29.03462029 | 28.99064255 |
|  | 0.806753632 | -0.043247223 | Q9JII6;B1AXW3 | Alcohol dehydrogenase [NADP(+)] | Akr1a1 | 29.06843185 | 29.0112133 | 29.08436012 | 29.11167717 | 29.10594559 | 29.07612419 |
|  | 1.38232784 | 0.145233154 | P48036 | Annexin A5 | Anxa5 | 28.93837166 | 28.95961189 | 28.80375099 | 28.75273705 | 28.74877548 | 28.76452255 |
|  | 0.761017362 | -0.154776891 | Q8CIB9 | N-acetyltransferase ESCO2 | Esco2 | 28.89994812 | 29.1779995 | 29.00466537 | 29.26389694 | 29.10224533 | 29.18080139 |
|  | 0.883552087 | 0.028572083 | P40124;B1ARS0;D3YTR7;A0A286YCS6;Q9CYT6 | Adenylyl cyclase-associated protein 1 | Cap1 | 28.69255447 | 28.68992424 | 28.69434929 | 28.69318581 | 28.64406204 | 28.65386391 |
|  | 1.162188115 | 0.209576289 | A0A0R4J1C8;P31996 | Macrosialin | Cd68 | 28.65242767 | 28.4368248 | 28.40701866 | 28.31777573 | 28.32988548 | 28.21988106 |
|  | 0.925011519 | 0.155379613 | Q05816 | Fatty acid-binding protein, epidermal | Fabp5 | 28.62655258 | 28.79113388 | 28.7827549 | 28.47030067 | 28.59775734 | 28.66624451 |
|  | 2.124798498 | -0.140396118 | P63017;Q504P4;P17156;D3Z5E2;Q61696;P16627;P17879 | Heat shock cognate 71 kDa protein | Hspa8 | 28.58895874 | 28.56292725 | 28.52234268 | 28.66637993 | 28.73615074 | 28.69288635 |
|  | 0.816262173 | 0.120494843 | P06151;A0A1B0GSX0;A0A1B0GSR9;D3YZQ9;A0A1B0GT41;A0A1B0GQX5;A0A1B0GSL7;A0A1B0GRW9;A0A1B0GS79;A0A1B0GRC1;A0A1B0GRS2;D3YVR7;D3YZE4;P00342;A0A1B0GSR2;A0A1B0GRE9 | L-lactate dehydrogenase A chain;L-lactate dehydrogenase | Ldha | 28.54233551 | 28.39362144 | 28.35758209 | 28.35476875 | 28.34307861 | 28.23420715 |
|  | 1.967362916 | 0.159460704 | P10107;A0A494BBD8 | Annexin A1 | Anxa1 | 28.52290344 | 28.5956955 | 28.61473846 | 28.38488007 | 28.41114616 | 28.45892906 |
|  | 1.493590023 | -0.117954254 | P09411;S4R2M7 | Phosphoglycerate kinase 1;Phosphoglycerate kinase | Pgk1 | 28.5175457 | 28.62957954 | 28.58724213 | 28.70391655 | 28.71979713 | 28.66451645 |
|  | 0.332813149 | -0.040252686 | Q99PT1 | Rho GDP-dissociation inhibitor 1 | Arhgdia | 28.47857857 | 28.38784027 | 28.50888443 | 28.43049812 | 28.53423119 | 28.53133202 |
|  | 2.033063694 | 0.124361038 | Q04447 | Creatine kinase B-type | Ckb | 28.37654114 | 28.32765961 | 28.35077095 | 28.2028923 | 28.27190781 | 28.20708847 |
|  | 0.241309068 | 0.031611125 | Q9CQI6;A0A1D5RLP1 | Coactosin-like protein | Cotl1 | 28.35955238 | 28.24422455 | 28.2994194 | 28.3213501 | 28.29508972 | 28.19192314 |
|  | 0.93820103 | -0.090850194 | P68372;Q9D6F9;G3UZR1 | Tubulin beta-4B chain;Tubulin beta-4A chain | Tubb4b;Tubb4a | 28.3259449 | 28.42666435 | 28.2908783 | 28.4090023 | 28.47637939 | 28.43065643 |
|  | 0.800651303 | -0.077662786 | Q9DBJ1 | Phosphoglycerate mutase 1 | Pgam1 | 28.28621483 | 28.40718079 | 28.36573792 | 28.42326164 | 28.38743019 | 28.48143005 |
|  | 1.108844045 | -0.12709109 | Q91VW3;I7HPY0 | SH3 domain-binding glutamic acid-rich-like protein 3 | Sh3bgrl3 | 28.26901245 | 28.30756378 | 28.25970459 | 28.3717308 | 28.50775146 | 28.33807182 |
|  | 1.550549498 | 0.093685786 | E9Q616;A0A494BBD5;G5E8K8;A0A494B8Y7 | AHNAK nucleoprotein (desmoyokin) | Ahnak | 28.23228645 | 28.23402405 | 28.1695919 | 28.09993935 | 28.15436363 | 28.10054207 |
|  | 1.425207419 | 0.106572469 | P17751;H7BXC3 | Triosephosphate isomerase | Tpi1 | 28.2063427 | 28.20806503 | 28.17126274 | 28.02575111 | 28.10479546 | 28.13540649 |
|  | 0.016476261 | -0.016373316 | Q9ERK4;E9Q1T9;E9QAX7;F6ZEW4 | Exportin-2 | Cse1l | 28.20531654 | 28.00474548 | 28.17640877 | 28.11356544 | 28.72100449 | 27.60102081 |
|  | 0.999453646 | -0.098082225 | A0A1W2P6F6;A0A1W2P7Q9;Q60605;Q8CI43 | Myosin light polypeptide 6 | Myl6 | 28.20284462 | 28.08156967 | 28.20973969 | 28.22669411 | 28.26736069 | 28.29434586 |
|  | 2.455049957 | -0.15514946 | H3BKH6;Q9R0P3;H3BLJ9;H3BJL6;H3BJP2;H3BK43;H3BJC6;H3BL99 | S-formylglutathione hydrolase | Esd | 28.17006874 | 28.09617424 | 28.1120739 | 28.30160141 | 28.27972221 | 28.26244164 |
|  | 0.77567946 | -0.033859253 | P07356;B0V2N5;B0V2N7;B0V2N8 | Annexin A2;Annexin | Anxa2 | 28.10614395 | 28.14160728 | 28.0995388 | 28.1677742 | 28.11921692 | 28.16187668 |
|  | 0.149001284 | -0.033355713 | P35279;D3YV69;P61294;A0A1L1SRS6;Q8BHD0 | Ras-related protein Rab-6A;Ras-related protein Rab-6B | Rab6a;Rab6b | 28.10054207 | 27.85590553 | 27.91798019 | 28.03133774 | 27.91245079 | 28.03070641 |
|  | 0.302981012 | 0.036826452 | Q9DCD0 | 6-phosphogluconate dehydrogenase, decarboxylating | Pgd | 28.08952141 | 28.10324669 | 28.23781204 | 28.10559464 | 28.0823822 | 28.13212395 |
|  | 0.176688705 | 0.034449259 | P20065 | Thymosin beta-4;Hematopoietic system regulatory peptide | Tmsb4x | 28.03149414 | 28.06794548 | 28.03128433 | 27.91125107 | 27.96427536 | 28.15184975 |
|  | 1.753812637 | 0.092126846 | Q91VI7;A0A1B0GSG5;A0A1B0GRG4;A0A1B0GRY7 | Ribonuclease inhibitor | Rnh1 | 28.02019691 | 27.96614838 | 28.03559113 | 27.89700699 | 27.93432808 | 27.91422081 |
|  | 1.365378272 | -0.110289892 | P10649;A2AE89;F6WHQ7;D3YVP5;Q80W21;P19639;D3YVP6;E9QAC8;P15626;D3YX76;Q8R5I6;D3YZ29;A2AE91;D3YVP9;D3YVP8;O35660;G5E8M7 | Glutathione S-transferase Mu 1 | Gstm1 | 27.98983002 | 28.09898758 | 28.06131554 | 28.15267181 | 28.13021088 | 28.19812012 |
|  | 0.04410955 | 0.007586161 | P63101;A0A2I3BQ03;D3YXN6;D3YXF4;D3YW45 | 14-3-3 protein zeta/delta | Ywhaz | 27.97880173 | 28.02173424 | 27.8649044 | 27.88721848 | 27.94547653 | 28.00998688 |
|  | 0.651354589 | 0.014404297 | P13020;A0A0J9YUQ8;A0A0J9YUJ8 | Gelsolin | Gsn | 27.97486877 | 27.99561119 | 27.97852898 | 27.96851158 | 27.9819622 | 27.95532227 |
|  | 0.465598274 | -0.020644506 | P11499;E9Q3D6;E9PX27;E9Q0C3;D3Z1R1 | Heat shock protein HSP 90-beta | Hsp90ab1 | 27.92104912 | 27.96350479 | 27.92178535 | 27.94341087 | 27.98223305 | 27.94262886 |
|  | 0.209752736 | -0.024734497 | P47738;A0A0G2JEU1;A0A0G2JF60;A0A0G2JFQ0;D3YYF3;Q62148;A0A0E2W2Y8;P24549;O35945;A0A0E2W6F6 | Aldehyde dehydrogenase, mitochondrial | Aldh2 | 27.82523346 | 27.91985512 | 27.76919556 | 27.85845757 | 27.84385872 | 27.88617134 |
|  | 2.983584067 | -0.087928772 | P80314;A0A1W2P7B7;A0A1W2P828;A0A1W2P6Q3;A0A1W2P8B6;A0A1W2P871 | T-complex protein 1 subunit beta | Cct2 | 27.82110214 | 27.85055161 | 27.81866646 | 27.91570282 | 27.91940117 | 27.91900253 |
|  | 0.020421036 | -0.002892812 | P14211 | Calreticulin | Calr | 27.77435875 | 27.83207321 | 27.76122856 | 27.83942032 | 27.82832527 | 27.70859337 |
|  | 1.284542953 | -0.102560679 | P40142;A0A286YE28;E0CY51 | Transketolase | Tkt | 27.76122856 | 27.83647346 | 27.83056259 | 27.93111801 | 27.85572815 | 27.94910049 |
|  | 2.424397165 | 0.118306478 | Q9D1A2;A0A494B9S3;A0A494B9U6 | Cytosolic non-specific dipeptidase | Cndp2 | 27.7326622 | 27.75964165 | 27.74963951 | 27.61034393 | 27.6118927 | 27.66478729 |
|  | 0.235132221 | 0.025318782 | D3Z2H9;D3YVR0;S4R2U0;A2AIM5 |  | Tpm3-rs7 | 27.68845749 | 27.60484314 | 27.62804985 | 27.67606544 | 27.55771637 | 27.61161232 |
|  | 1.369321737 | -0.0525678 | P58252 | Elongation factor 2 | Eef2 | 27.66566849 | 27.64595222 | 27.64416504 | 27.71423912 | 27.67222595 | 27.72702408 |
|  | 4.021189962 | 0.128939946 | Q03265;D3Z6F5;D6RJ16;A0A0E2WDP1 | ATP synthase subunit alpha, mitochondrial;ATP synthase subunit alpha | Atp5a1 | 27.66091728 | 27.66559982 | 27.68525314 | 27.53660393 | 27.54811668 | 27.5402298 |
|  | 0.011211823 | -0.001199722 | O88342;A0A0J9YU05 | WD repeat-containing protein 1 | Wdr1 | 27.62428856 | 27.72968483 | 27.66180038 | 27.63948059 | 27.68404961 | 27.69584274 |
|  | 0.813979233 | -0.310107867 | Q6IFZ6;Q6IFZ9 | Keratin, type II cytoskeletal 1b | Krt77 | 27.60349846 | 27.65969276 | 27.59149361 | 28.14413834 | NaN | 27.71253395 |
|  | 0.239044721 | 0.028608322 | P62821 | Ras-related protein Rab-1A | Rab1A | 27.54369926 | 27.44181824 | 27.46987343 | 27.52731133 | 27.43292809 | 27.40932655 |
|  | 1.448083509 | 0.157061895 | P59999;Q3TX55;E9PWA7 | Actin-related protein 2/3 complex subunit 4 | Arpc4 | 27.52790833 | 27.43610954 | 27.39263916 | 27.25799751 | 27.35632324 | 27.27115059 |
|  | 2.370521888 | -0.070768356 | B7FAU9;Q8BTM8;B7FAV1;F6XC15;J3JS91;F6Z2C0;F7AVL7;A0A668KLG7 | Filamin-A | Flna | 27.495718 | 27.53326416 | 27.51103592 | 27.58584595 | 27.59220695 | 27.57427025 |
|  | 0.205107048 | 0.014085134 | P09103;E9Q8G8 | Protein disulfide-isomerase | P4hb | 27.47126961 | 27.50620079 | 27.4968605 | 27.49274254 | 27.42958069 | 27.50975227 |
|  | 0.997455741 | 0.163974126 | Q9Z0M5 | Lysosomal acid lipase/cholesteryl ester hydrolase | Lipa | 27.44963074 | 27.46279335 | 27.59398842 | 27.29070282 | 27.46076393 | 27.26302338 |
|  | 1.470636022 | -0.08919398 | P20029 | 78 kDa glucose-regulated protein | Hspa5 | 27.38829231 | 27.42758369 | 27.35934258 | 27.45230484 | 27.51945877 | 27.47103691 |
|  | 1.434071101 | 0.248980204 | P70349;B0R1E3 | Histidine triad nucleotide-binding protein 1 | Hint1 | 27.38632011 | 27.39198303 | 27.5382328 | 27.31034088 | 27.09416199 | 27.16509247 |
|  | 0.503505513 | 0.078989665 | O88668;K4DI63;J3QP41 | Protein CREG1 | Creg1 | 27.35925865 | 27.55208206 | 27.45371819 | 27.40194321 | 27.42878151 | 27.29736519 |
|  | 0.772126157 | -0.074269613 | Q05144;A0A2R8VHH0 | Ras-related C3 botulinum toxin substrate 2 | Rac2 | 27.3583374 | 27.22269249 | 27.30138397 | 27.33500671 | 27.40543747 | 27.36477852 |
|  | 0.641766284 | -0.109100342 | P99027;A0A5F8MPY2 | 60S acidic ribosomal protein P2 | Rplp2 | 27.35640717 | 27.50415421 | 27.38038635 | 27.40153503 | 27.60229492 | 27.56441879 |
|  | 0.336085207 | 0.050626119 | Q9Z1Q5 | Chloride intracellular channel protein 1 | Clic1 | 27.35161209 | 27.30712891 | 27.39190102 | 27.39713478 | 27.19919777 | 27.30243111 |
|  | 2.380528745 | -0.125401815 | Q62465 | Synaptic vesicle membrane protein VAT-1 homolog | Vat1 | 27.32302666 | 27.32431602 | 27.3609333 | 27.44624329 | 27.44221497 | 27.49602318 |
|  | 0.443259348 | 0.030083338 | Q9Z0J0 | Epididymal secretory protein E1 | Npc2 | 27.30730247 | 27.32242584 | 27.29500198 | 27.2228775 | 27.29754066 | 27.31406212 |
|  | 1.569310134 | 0.156448364 | Q60854;F8WIV2;K7E6F1;E9Q108;E9Q0P9;E9Q3Y1;E9Q6X2;E9PYY0;E9PZQ9;E9Q4R2;E9Q5Q5;Q3UWK8 | Serpin B6 | Serpinb6;Serpinb6a | 27.30260468 | 27.4512825 | 27.37525749 | 27.22628212 | 27.24408913 | 27.18942833 |
|  | 1.221877965 | 0.227218628 | Q9CPW4;Q3UA72 | Actin-related protein 2/3 complex subunit 5 | Arpc5 | 27.29017639 | 27.46606636 | 27.16710472 | 27.08881378 | 27.09285164 | 27.06002617 |
|  | 1.218730108 | -0.085861206 | P14206;A0A1L1SUK3;A0A1L1SRW0 | 40S ribosomal protein SA | Rpsa | 27.27985382 | 27.26633263 | 27.21724892 | 27.37807274 | 27.28779984 | 27.35514641 |
|  | 1.026463525 | -0.108774821 | P61982 | 14-3-3 protein gamma;14-3-3 protein gamma, N-terminally processed | Ywhag | 27.25925636 | 27.30895233 | 27.14996147 | 27.34638023 | 27.32061958 | 27.37749481 |
|  | 1.8831453 | 0.091440837 | P50516;D3Z1B9;D3YWH3;D3YZ23 | V-type proton ATPase catalytic subunit A | Atp6v1a | 27.25799751 | 27.23708344 | 27.22775078 | 27.12612915 | 27.18829536 | 27.1340847 |
|  | 0.711405519 | 0.079788208 | P97429;A0A0N4SW89;D3Z0S1;A0A0N4SV57;F7ANV6;S4R1F2 | Annexin A4;Annexin | Anxa4 | 27.23662758 | 27.27239799 | 27.26811981 | 27.25817871 | 27.0866909 | 27.19291115 |
|  | 2.109021433 | 0.052935918 | Q68FD5;Q5SXR6;F6Z1R4 | Clathrin heavy chain 1;Clathrin heavy chain | Cltc | 27.21687889 | 27.24970436 | 27.22223282 | 27.18148804 | 27.17025948 | 27.1782608 |
|  | 1.00550175 | -0.120178858 | P62880;E9QKR0;D3YZX3;D3Z1M1;D3Z1T4;E9PWM7;A0A0A6YVN9;V9GWY1 | Guanine nucleotide-binding protein G(I)/G(S)/G(T) subunit beta-2 | Gnb2 | 27.17607498 | 27.01828957 | 27.18725777 | 27.27248764 | 27.24054527 | 27.22912598 |
|  | 1.133310055 | -0.297210693 | P30355;A0A0J9YUC8 | Arachidonate 5-lipoxygenase-activating protein | Alox5ap | 27.162117 | 27.12415695 | 27.14365196 | 27.19873047 | 27.59917641 | 27.52365112 |
|  | 0.911429176 | -0.087921778 | P62751 | 60S ribosomal protein L23a | Rpl23a | 27.13339806 | 27.19919777 | 27.06455612 | 27.2652607 | 27.20377922 | 27.19187737 |
|  | 1.019727444 | -0.073906581 | P60766;A0A2R8VH29;G3UZM2;D3Z3L1;F2Z463;D3YX61;Q8R527;Q9ER71 | Cell division control protein 42 homolog | Cdc42 | 27.11376381 | 27.08557701 | 27.15441132 | 27.13780212 | 27.20955276 | 27.22811699 |
|  | 0.740488058 | -0.08799998 | O08997 | Copper transport protein ATOX1 | Atox1 | 27.11078072 | 27.06157303 | 27.03401756 | 27.06609726 | 27.23726463 | 27.16700935 |
|  | 0.545582617 | 1.27215004 | P26350;A0A087WP98;A0A087WPN6;A0A087WQN2 | Prothymosin alpha;Prothymosin alpha, N-terminally processed;Thymosin alpha | Ptma | 27.10799026 | 26.67828369 | 26.59576607 | 26.21300697 | 26.83581161 | 23.51677132 |
|  | 0.184187248 | -0.033156077 | P14115 | 60S ribosomal protein L27a | Rpl27a | 27.06332207 | 27.22986031 | 27.0240078 | 27.10819054 | 27.11565018 | 27.19281769 |
|  | 2.077879852 | -0.148654302 | P43276 | Histone H1.5 | Hist1h1b | 27.05631065 | 27.00688744 | 26.9671917 | 27.19187737 | 27.13955879 | 27.14491653 |
|  | 0.884798369 | 0.110494614 | Q64433;Q9JI95 | 10 kDa heat shock protein, mitochondrial | Hspe1;Cpn10-rs1 | 27.05051041 | 27.00945282 | 27.05755043 | 26.94525337 | 27.01669502 | 26.82408142 |
|  | 0.954636781 | -0.072199504 | P60843 | Eukaryotic initiation factor 4A-I | Eif4a1 | 27.04280853 | 27.02812576 | 26.93021584 | 27.06999397 | 27.07101822 | 27.07673645 |
|  | 2.367954593 | -0.061468124 | P26039;A2AIM2;E9PUM4;A0A1L1SQ51;Q71LX4;Q8CDM9;A0A1L1SRI1;A0A1L1SQP9;F6S1V7;F6SX70 | Talin-1 | Tln1 | 27.03956985 | 27.02252769 | 27.00774193 | 27.07581902 | 27.08486748 | 27.09355736 |
|  | 0.796855837 | -0.063702265 | O89053;G3UYK8;A0A0U1RPY8;D3YW57;G3UX53;D3YXM2 | Coronin-1A;Coronin | Coro1a | 27.02019691 | 26.94290733 | 26.98283195 | 27.09938812 | 26.99798012 | 27.03967476 |
|  | 0.988424447 | 0.077447891 | Q61598;A0A1Y7VL99;A0A1Y7VLG4 | Rab GDP dissociation inhibitor beta | Gdi2 | 26.99658012 | 27.01467514 | 27.08780289 | 26.92195702 | 26.94290733 | 27.00185013 |
|  | 1.205098809 | 0.122198105 | Q9QUI0;A0A0A6YXF6;Q62159;H3BL56;A0A0G2JEP8;Q9CR99;A0A0A6YWJ1 | Transforming protein RhoA;Rho-related GTP-binding protein RhoC | Rhoa;Rhoc | 26.95249367 | 26.9971199 | 27.0514431 | 26.80747223 | 26.88878632 | 26.93820381 |
|  | 1.63908098 | 0.14217631 | P62827;Q14AA6;Q61820 | GTP-binding nuclear protein Ran | Ran;1700009N14Rik | 26.93505859 | 26.92025375 | 26.87020683 | 26.72572517 | 26.83472824 | 26.73853683 |
|  | 0.63535435 | 0.04999733 | P49710;E9Q4E5 | Hematopoietic lineage cell-specific protein | Hcls1 | 26.93370819 | 26.88145447 | 26.87349701 | 26.83376312 | 26.80156326 | 26.90334129 |
|  | 0.236579441 | -0.020709991 | Q01853 | Transitional endoplasmic reticulum ATPase | Vcp | 26.93168068 | 27.02379799 | 26.9770546 | 26.97585297 | 26.97683716 | 27.04197311 |
|  | 0.433952104 | 0.169110616 | P62855 | 40S ribosomal protein S26 | Rps26 | 26.91752434 | 26.91820717 | 27.38853836 | 26.95249367 | 26.97191238 | 26.79253197 |
|  | 0.21146792 | -0.028351466 | Q9D8E6 | 60S ribosomal protein L4 | Rpl4 | 26.89642906 | 26.89700699 | 26.75595474 | 26.89723778 | 26.90368652 | 26.83352089 |
|  | 0.910024962 | 0.06994311 | Q8R0X7;D6REF7;D3YZT4;D3YY13;D3Z1Z3 | Sphingosine-1-phosphate lyase 1 | Sgpl1 | 26.8872757 | 26.83496857 | 26.87244034 | 26.81041718 | 26.73285675 | 26.84158134 |
|  | 0.862010351 | -0.317587535 | Q9QWL7;Q99PS0 | Keratin, type I cytoskeletal 17 | Krt17 | 26.85887337 | 26.96708298 | 26.95204926 | 27.39402962 | 27.42846298 | 26.9082756 |
|  | 0.095657942 | -0.021394094 | P63028;D3YU75 | Translationally-controlled tumor protein | Tpt1 | 26.85388565 | 26.87220573 | 26.85554886 | 26.81775284 | 26.7875576 | 27.04051208 |
|  | 0.264044614 | 0.049886068 | P51150;A0A0N4SVG9;A0A0N4SVR6 | Ras-related protein Rab-7a | Rab7a | 26.85245705 | 26.9413414 | 26.72129822 | 26.7203846 | 26.85899162 | 26.78606224 |
|  | 1.075162031 | 0.256487528 | Q61792;A2A6H0;A2A6G9;A2A6G7;A2A6G8;A2A6G6;A2A6H1;A2A6G5;E9Q0N6;A2A6G0;A2A6G4;Q9DC07 | LIM and SH3 domain protein 1 | Lasp1 | 26.84804535 | 26.5452919 | 26.64622688 | 26.55929184 | 26.34521294 | 26.36559677 |
|  | 0.903745071 | 0.149981817 | Q9CQV8;A2A5N1 | 14-3-3 protein beta/alpha;14-3-3 protein beta/alpha, N-terminally processed | Ywhab | 26.84708977 | 26.87935066 | 26.85245705 | 26.78643608 | 26.55607986 | 26.78643608 |
|  | 2.557591934 | 0.14323171 | P08752;A0A0A6YWA9;B2RSH2;A0A0A6YXN0;A0A0A6YXC2 | Guanine nucleotide-binding protein G(i) subunit alpha-2 | Gnai2 | 26.82335281 | 26.80869865 | 26.80906677 | 26.63228607 | 26.70569611 | 26.67344093 |
|  | 1.654559171 | 0.171637217 | P47754;D6RCW7;A0A0N4SVM0 | F-actin-capping protein subunit alpha-2 | Capza2 | 26.81604385 | 26.88297081 | 26.72650337 | 26.61386108 | 26.66044235 | 26.63630295 |
|  | 1.287650164 | 0.186755498 | Q9WUU7 | Cathepsin Z | Ctsz | 26.79066849 | 26.87114716 | 26.95870018 | 26.77115059 | 26.60625458 | 26.68284416 |
|  | 0.438026806 | 0.061384837 | Q3TLP8;P63001;P60764;A2AC13 | Ras-related C3 botulinum toxin substrate 1;Ras-related C3 botulinum toxin substrate 3 | Rac1;Rac3 | 26.74804115 | 26.61876869 | 26.59106445 | 26.52299309 | 26.64320183 | 26.60752487 |
|  | 0.445671348 | 0.037616094 | P06745;A0A0U1RQ72;A0A0U1RP97;A0A0U1RQ18 | Glucose-6-phosphate isomerase | Gpi | 26.68618965 | 26.73531151 | 26.6685791 | 26.71581078 | 26.64910698 | 26.61231422 |
|  | 0.433350514 | -0.051927567 | G5E850;P56395;A0A494B9D8;E0CY88 | Cytochrome b5 | Cyb5a | 26.66803741 | 26.76988983 | 26.60484314 | 26.6971035 | 26.74931908 | 26.75213051 |
|  | 2.771814187 | -0.123657227 | P08207 | Protein S100-A10 | S100a10 | 26.65485382 | 26.66180038 | 26.65389824 | 26.74791145 | 26.79649734 | 26.79711533 |
|  | 0.386970091 | -0.041836421 | P62908;D3YV43;A0A140LI77 | 40S ribosomal protein S3 | Rps3 | 26.65212059 | 26.57715416 | 26.55978775 | 26.6607132 | 26.68552017 | 26.56833839 |
|  | 1.045766556 | 0.110745748 | P68040 | Guanine nucleotide-binding protein subunit beta-2-like 1;Guanine nucleotide-binding protein subunit beta-2-like 1, N-terminally processed | Gnb2l1 | 26.64471436 | 26.54537964 | 26.62435913 | 26.41744614 | 26.51601982 | 26.54874992 |
|  | 0.215060607 | -0.035608927 | Q9CXW4;A2BH06 | 60S ribosomal protein L11 | Rpl11 | 26.62421799 | 26.63450241 | 26.60964012 | 26.76268578 | 26.67033577 | 26.54216576 |
|  | 0.568424469 | -0.105622609 | P24369 | Peptidyl-prolyl cis-trans isomerase B | Ppib | 26.6180687 | 26.53569984 | 26.35051727 | 26.60893631 | 26.64718819 | 26.56502914 |
|  | 0.028905972 | 0.006328583 | F6YVP7;P62270;S4R1N6;A0A1Y7VKY1;A0A3Q4EGP3 | 40S ribosomal protein S18 | Gm10260;Rps18 | 26.61526489 | 26.71227264 | 26.63975525 | 26.52448845 | 26.7565918 | 26.66722679 |
|  | 1.495669954 | -0.186508179 | Q8C845;Q9D8Y0;Q9D4J1 | EF-hand domain-containing protein D2 | Efhd2 | 26.61245537 | 26.47436905 | 26.56706238 | 26.74201202 | 26.66478729 | 26.80661201 |
|  | 0.013930888 | -0.006331762 | P43277 | Histone H1.3 | Hist1h1d | 26.59249115 | 26.61498451 | 26.84505653 | 26.46271515 | 26.70754051 | 26.90127182 |
|  | 1.342765083 | 0.140821457 | P35979 | 60S ribosomal protein L12 | Rpl12 | 26.58949471 | 26.60470009 | 26.55929184 | 26.42439842 | 26.53333855 | 26.37328529 |
|  | 1.770063435 | -0.167221069 | P97351 | 40S ribosomal protein S3a | Rps3a | 26.5721035 | 26.50969124 | 26.50756073 | 26.70266151 | 26.75773621 | 26.63062096 |
|  | 0.983070494 | -0.072392782 | A0A3B2WDD2;A0A3B2WBL1;Q5XJF6;P53026;A0A3B2W824;A0A3B2W820;A0A3B2WCA5;D6RE43 | Ribosomal protein;60S ribosomal protein L10a | Rpl10a | 26.56620598 | 26.60667801 | 26.53170395 | 26.63809967 | 26.59534073 | 26.68832588 |
|  | 1.010678317 | -0.18270874 | P62918 | 60S ribosomal protein L8 | Rpl8 | 26.55310822 | 26.58003426 | 26.3754406 | 26.78194237 | 26.58978081 | 26.68498611 |
|  | 1.567833715 | 0.142825445 | P47955 | 60S acidic ribosomal protein P1 | Rplp1 | 26.53784752 | 26.59860992 | 26.65771866 | 26.5017128 | 26.42471886 | 26.43926811 |
|  | 1.935614829 | 0.093935649 | Q9WUM4;E9PX03;E9PZJ0;E9PVJ1;B9EIZ7;Q920M5 | Coronin-1C | Coro1c | 26.52593803 | 26.57201576 | 26.55062866 | 26.42723083 | 26.48480606 | 26.45473862 |
|  | 1.884604139 | -0.151344299 | Q8BG05;A2AL12;A2AL13 | Heterogeneous nuclear ribonucleoprotein A3 | Hnrnpa3 | 26.52004242 | 26.5609684 | 26.45240021 | 26.63948059 | 26.69351959 | 26.65444374 |
|  | 0.42301375 | -0.067914327 | O35405 | Phospholipase D3 | Pld3 | 26.51780701 | 26.66220856 | 26.74342537 | 26.72116661 | 26.67290115 | 26.73311615 |
|  | 2.017289252 | 0.203531265 | Q99JI6;A0A1W2P777 | Ras-related protein Rap-1b | Rap1b | 26.51121712 | 26.55846024 | 26.64553833 | 26.39803314 | 26.33280563 | 26.37378311 |
|  | 1.400042967 | 0.226366679 | Q76MZ3;G3UWL2;G3UXQ1;H3BK50;H3BJ83 | Serine/threonine-protein phosphatase 2A 65 kDa regulatory subunit A alpha isoform | Ppp2r1a | 26.50948143 | 26.35674286 | 26.30940437 | 26.20297623 | 26.07567596 | 26.21787643 |
|  | 1.972347151 | -0.200029373 | P35980;A0A1B0GSS8;A0A1B0GQU8;A0A1B0GSF7;A0A1B0GSA8;A0A1B0GS28;A0A1B0GRZ3 | 60S ribosomal protein L18 | Rpl18 | 26.5060482 | 26.46712303 | 26.51738739 | 26.70411491 | 26.76496506 | 26.62156677 |
|  | 1.056648733 | -0.044881185 | Q00612;A3KG36;G3UWD6;P97324;REV__P46662 | Glucose-6-phosphate 1-dehydrogenase X;Glucose-6-phosphate 1-dehydrogenase | G6pdx | 26.49832344 | 26.44673157 | 26.48260689 | 26.4962368 | 26.53969765 | 26.526371 |
|  | 0.455606812 | 0.078344981 | E9Q9C5;P63082 | V-type proton ATPase 16 kDa proteolipid subunit | Atp6v0c | 26.48179245 | 26.62797928 | 26.42712021 | 26.50433731 | 26.44339943 | 26.35412025 |
|  | 0.062587825 | 0.022361755 | Q9CZM2;B8JKK2 | 60S ribosomal protein L15;Ribosomal protein L15 | Rpl15 | 26.47041702 | 26.53781891 | 26.41141319 | 26.53122902 | 26.21734047 | 26.60399437 |
|  | 0.902130932 | 0.114560445 | Q9DCN2;F2Z456 | NADH-cytochrome b5 reductase 3;NADH-cytochrome b5 reductase 3 membrane-bound form;NADH-cytochrome b5 reductase 3 soluble form;NADH-cytochrome b5 reductase | Cyb5r3 | 26.4652729 | 26.30413818 | 26.4366188 | 26.23418045 | 26.34589005 | 26.28227806 |
|  | 0.031413171 | -0.006024043 | P62889;A0A2I3BQF4 | 60S ribosomal protein L30 | Rpl30 | 26.44042587 | 26.48054504 | 26.4421196 | 26.56396675 | 26.47161102 | 26.34558487 |
|  | 0.195033129 | -0.032505671 | Q91V41;Q50HX3;A0A2R8VHW9 | Ras-related protein Rab-14 | Rab14 | 26.4335022 | 26.59988594 | 26.42186546 | 26.4720459 | 26.51187897 | 26.56884575 |
|  | 0.831085865 | 0.142801285 | P41105 | 60S ribosomal protein L28 | Rpl28 | 26.4223156 | 26.54034805 | 26.36811447 | 26.42149734 | 26.22147751 | 26.25939941 |
|  | 0.262545967 | -0.041001638 | P62702 | 40S ribosomal protein S4, X isoform | Rps4x | 26.42032433 | 26.36614609 | 26.29480934 | 26.49606895 | 26.38506889 | 26.32314682 |
|  | 1.23477982 | 0.095148087 | P38647 | Stress-70 protein, mitochondrial | Hspa9 | 26.41143036 | 26.50780296 | 26.47633171 | 26.32548332 | 26.38730621 | 26.39733124 |
|  | 0.38605052 | 0.060971578 | P51410;A0A0G2JES3;A0A140T8T4;D3Z629;D3YZT0;A0A0G2JFQ3 | 60S ribosomal protein L9 | Rpl9 | 26.40431595 | 26.44031334 | 26.26822662 | 26.35564995 | 26.3460083 | 26.22828293 |
|  | 0.380478402 | -0.061508814 | P54116 | Erythrocyte band 7 integral membrane protein | Stom | 26.40418625 | 26.44845009 | 26.50839233 | 26.44056702 | 26.63574982 | 26.46923828 |
|  | 0.348259662 | -0.024126053 | P25444;D3YVC1;D3YWJ3 | 40S ribosomal protein S2 | Rps2 | 26.39969635 | 26.46699905 | 26.39610672 | 26.44625854 | 26.41475487 | 26.47416687 |
|  | 1.197584587 | 0.068576177 | P47911;A0A0J9YU32 | 60S ribosomal protein L6 | Rpl6 | 26.39917374 | 26.34440041 | 26.36972809 | 26.28843498 | 26.27389145 | 26.34524727 |
|  | 0.701981533 | 0.057657242 | Q80T06;P57776;A0A0R4J1E2;E9QN08;D3YUQ9;D3YZT9 | Elongation factor 1-delta | Eef1d | 26.39188576 | 26.40679932 | 26.32505417 | 26.30547714 | 26.27556229 | 26.36972809 |
|  | 1.169847006 | -0.150466919 | P12970 | 60S ribosomal protein L7a | Rpl7a | 26.38885117 | 26.36724663 | 26.53666306 | 26.56639481 | 26.63685608 | 26.54091072 |
|  | 0.436126591 | -0.020587921 | Q6ZWQ9;D3YV37 | Myosin, light chain 12A, regulatory, non-sarcomeric | Myl12a | 26.36626434 | 26.3240242 | 26.37620163 | 26.35403633 | 26.39687347 | 26.37734413 |
|  | 1.091161143 | -0.093422572 | Q6ZWV3;I7HLV2;P86048;A0A1B0GXC3 | 60S ribosomal protein L10;60S ribosomal protein L10-like | Rpl10;Rpl10l | 26.36564636 | 26.41330338 | 26.45126724 | 26.49098396 | 26.45586777 | 26.56363297 |
|  | 0.9696597 | -0.223098755 | E9Q7Q3;D3Z6I8 | Tropomyosin 3, gamma | Tpm3 | 26.36447716 | 26.09711838 | 26.27870178 | 26.55539131 | 26.32352448 | 26.5306778 |
|  | 1.663373078 | 0.18905894 | Q9D3D9;D3Z7S4 | ATP synthase subunit delta, mitochondrial | Atp5d | 26.36031342 | 26.25255775 | 26.38320923 | 26.15425682 | 26.19274139 | 26.08190536 |
|  | 0.119680412 | 0.021901449 | Q6ZWN5;F7CJS8;D3YWH9;Q9CXW7;D3Z673;D3YUV6 | 40S ribosomal protein S9 | Rps9 | 26.33670998 | 26.1764946 | 26.21051979 | 26.30166245 | 26.21196747 | 26.14439011 |
|  | 0.537647769 | -0.120669047 | P47915;W4VSN7;A0A1L1SS27;A0A1L1STJ3;A0A1L1SUN1 | 60S ribosomal protein L29 | Rpl29;Gm3550 | 26.33429146 | 26.41060448 | 26.25172806 | 26.62589073 | 26.39196777 | 26.34077263 |
|  | 0.675276883 | -0.046236038 | P37040;Q05DV1;E9Q997;E9PVT9;F6R7H8 | NADPH--cytochrome P450 reductase | Por | 26.32860184 | 26.32433319 | 26.31099892 | 26.32813835 | 26.34661674 | 26.42788696 |
|  | 0.289682217 | -0.053679784 | P14148;F6XI62 | 60S ribosomal protein L7 | Rpl7 | 26.31884575 | 26.19646072 | 26.27175713 | 26.42665672 | 26.1987114 | 26.32273483 |
|  | 0.83734121 | 0.131783803 | P35278;Q8C266;A2A5F5;A2A5F6 | Ras-related protein Rab-5C | Rab5c | 26.31874084 | 26.19591713 | 26.31103325 | 26.23772049 | 26.02850533 | 26.164114 |
|  | 1.202303488 | 0.074333191 | Q543K9;P23492;A0A2I3BQH2;Q9D8C9;A0A2I3BS22 | Purine nucleoside phosphorylase | Pnp | 26.30582619 | 26.29279327 | 26.22070122 | 26.22184563 | 26.18161964 | 26.19285583 |
|  | 0.232606967 | -0.11030515 | A0A0R4J0F6;Q99KY4;D6RHK5;A0A0G2JGP6;A0A0G2JEW6 | Cyclin-G-associated kinase | Gak | 26.30150604 | 26.33449554 | 26.15402603 | 26.72181892 | 26.13803673 | 26.26108742 |
|  | 0.136054047 | 0.010069529 | Q3TW96;A0A0R4J1F6;A0A0R4J085;Q3UHZ7;Q91YN5 | UDP-N-acetylhexosamine pyrophosphorylase-like protein 1 | Uap1l1 | 26.29352951 | 26.25365829 | 26.25727844 | 26.2125988 | 26.29496765 | 26.26669121 |
|  | 1.959983629 | -0.159852982 | P60335;A0A087WR61;A0A087WSI1;A0A1L1SUS5;A0A1L1SRP9;A0A1L1SQ99;A0A087WRH3;A0A0R4J044;P57724 | Poly(rC)-binding protein 1 | Pcbp1 | 26.29245949 | 26.36537933 | 26.3969059 | 26.49564171 | 26.54685211 | 26.49180984 |
|  | 1.096318791 | 0.095448812 | P62911 | 60S ribosomal protein L32 | Rpl32 | 26.28744888 | 26.22337532 | 26.27912712 | 26.13643265 | 26.12815666 | 26.23901558 |
|  | 0.06229042 | -0.028600693 | P70670;Q60817 | Nascent polypeptide-associated complex subunit alpha, muscle-specific form;Nascent polypeptide-associated complex subunit alpha | Naca | 26.26842308 | 26.00757217 | 26.20897675 | 25.98888969 | 26.45583534 | 26.12604904 |
|  | 0.003789676 | -0.000711441 | Q9QUM9;E0CXB1;E0CYT2 | Proteasome subunit alpha type-6 | Psma6 | 26.26786995 | 26.29920197 | 26.21621132 | 26.35092163 | 26.15722847 | 26.27726746 |
|  | 1.475214395 | -0.238543193 | Q78ZM0;O70492;D3Z789;D3Z6Z0 | Sorting nexin-3 | Snx3 | 26.25284767 | 26.28123474 | 26.11368561 | 26.38475609 | 26.56127548 | 26.41736603 |
|  | 0.097936241 | -0.005450567 | O35639;Q3TET3;A0A0G2JDV9;A0A0G2JGL7 | Annexin A3 | Anxa3 | 26.24085426 | 26.1774826 | 26.21374893 | 26.20080757 | 26.22611618 | 26.22151375 |
|  | 1.272013352 | -0.068641663 | P46638;G3UY29;E9Q3P9;F8WGS1;P62492;G3UZD3;G3UZL4;E9Q6B3;Q9WTL2 | Ras-related protein Rab-11B;Ras-related protein Rab-11A | Rab11b;Rab11a | 26.23218727 | 26.25355148 | 26.19642448 | 26.29664803 | 26.328722 | 26.2627182 |
|  | 0.27831009 | 0.026141485 | P50518;A0A0N4SW07;A0A0N4SW34;A0A0N4SWA3;Q9D593 | V-type proton ATPase subunit E 1 | Atp6v1e1 | 26.19857979 | 26.22556496 | 26.21059418 | 26.11257172 | 26.23249817 | 26.21124458 |
|  | 0.10670004 | 0.042473475 | Q6ZWZ4;P47964 | 60S ribosomal protein L36 | Rpl36 | 26.18618202 | 26.49089241 | 26.67559433 | 26.44020271 | 26.39972878 | 26.38531685 |
|  | 0.938528678 | 0.136185964 | A2A813;Q99LX0;A2A815;A2A817;A2A816 | Protein deglycase DJ-1 | Park7 | 26.18215179 | 25.96093369 | 26.13398552 | 25.9669075 | 25.9647274 | 25.9368782 |
|  | 0.301991908 | 0.034658432 | Q8BLF1;Q8BYQ0;D6RGP7 | Neutral cholesterol ester hydrolase 1 | Nceh1 | 26.17337036 | 26.11076164 | 26.11903954 | 26.06404305 | 26.18406296 | 26.05109024 |
|  | 1.07000631 | -0.205146154 | P62082 | 40S ribosomal protein S7 | Rps7 | 26.16842461 | 26.14456558 | 26.32436752 | 26.40002251 | 26.30558205 | 26.54719162 |
|  | 1.296490857 | -0.114874522 | P48678;D3YUF7 | Prelamin-A/C;Lamin-A/C | Lmna | 26.16004181 | 26.09385872 | 26.07345009 | 26.28829384 | 26.19666862 | 26.18701172 |
|  | 0.027778122 | 0.005055745 | P57759;F8WJI4;F8WIM7;D6RG87 | Endoplasmic reticulum resident protein 29 | Erp29 | 26.12992477 | 25.97237396 | 26.0294323 | 25.97142982 | 26.03473091 | 26.11040306 |
|  | 2.469779045 | -0.183785756 | O08553;Q3TT92;E9PWE8;Q62188;Q6P1J1;P97427;Q71H75 | Dihydropyrimidinase-related protein 2 | Dpysl2 | 26.12346649 | 26.15855789 | 26.12287521 | 26.2691021 | 26.36223793 | 26.32491684 |
|  | 0.824240813 | -0.170103709 | P62774;A0A0J9YV46 | Myotrophin | Mtpn | 26.12020683 | 26.40177917 | 26.3724041 | 26.40019989 | 26.5004673 | 26.50403404 |
|  | 0.926496017 | 0.138503393 | B2M1R6;H3BKD0;H3BKI8;Q8BT23;H3BLP7;A0A286YE41;H3BJ43;H3BJS9 | Heterogeneous nuclear ribonucleoprotein K | Hnrnpk | 26.11040306 | 26.11181641 | 26.22809982 | 25.91150284 | 26.0110321 | 26.11227417 |
|  | 1.851599276 | -0.212758382 | P60867 | 40S ribosomal protein S20 | Rps20 | 26.10924721 | 26.0237751 | 26.0709362 | 26.35464287 | 26.20026588 | 26.28732491 |
|  | 0.106327872 | 0.027640025 | P62960;A2BGG7;A0A0A0MQD2;B2RUF0;Q9Z2C8 | Nuclease-sensitive element-binding protein 1 | Ybx1 | 26.09995079 | 26.18718338 | 26.26096153 | 26.10335541 | 26.04720497 | 26.31461525 |
|  | 1.643061301 | 0.222166061 | A0A0A6YX18;Q8BVE3;A0A0A6YWP6;A0A0A6YVU0;A0A0A6YW86 | V-type proton ATPase subunit H | Atp6v1h | 26.09591293 | 25.99187469 | 26.00812721 | 25.91298676 | 25.77567863 | 25.74075127 |
|  | 0.831739186 | -0.344275157 | P17047 | Lysosome-associated membrane glycoprotein 2 | Lamp2 | 26.09315491 | 26.4512825 | 25.86582565 | 26.65744591 | 26.38482285 | 26.40081978 |
|  | 0.585098531 | -0.166964849 | P26040 | Ezrin | Ezr | 26.09287262 | 26.17761612 | 26.3502636 | 26.3983593 | 26.53765488 | 26.18563271 |
|  | 0.739623179 | 0.080802282 | P27659;A0A2R8VHN4;A0A087WQK0;A0A087WNS0;Q9CQ09;E9PWZ3 | 60S ribosomal protein L3 | Rpl3 | 26.08982468 | 26.01441956 | 26.15690041 | 25.97672844 | 25.97860527 | 26.06340408 |
|  | 1.961913554 | 0.129727046 | P57780;A0A1L1SV25;E9Q2W9;A0A1L1SVJ6;A0A1Y7VMW4 | Alpha-actinin-4 | Actn4 | 26.07060814 | 26.14749908 | 26.12756538 | 25.95540047 | 26.01584625 | 25.98524475 |
|  | 2.134372213 | 0.165791829 | P20060 | Beta-hexosaminidase subunit beta | Hexb | 26.06900978 | 25.98578835 | 25.97635651 | 25.8582325 | 25.81494522 | 25.86060143 |
|  | 0.69896845 | -0.075157801 | G3X8T3;P16675;A2A5J8;A2A5J9 | Carboxypeptidase;Lysosomal protective protein;Lysosomal protective protein 32 kDa chain;Lysosomal protective protein 20 kDa chain | Ctsa | 26.06819153 | 26.13850403 | 26.04891014 | 26.08080673 | 26.2155838 | 26.18468857 |
|  | 1.232665877 | 0.172363917 | A0A338P786;P68037;A0A338P7E5;A0A338P702 | Ubiquitin-conjugating enzyme E2 L3 | Ube2l3 | 26.06650734 | 26.11109924 | 26.13100624 | 25.90520287 | 25.8365345 | 26.04978371 |
|  | 0.679122894 | -0.132517497 | P63325;A0A338P731;Q3UW83;A0A338P7K4;A0A3B2W864 | 40S ribosomal protein S10 | Rps10 | 26.0605011 | 26.18455505 | 26.00043297 | 26.07443047 | 26.27494049 | 26.29367065 |
|  | 0.13580131 | -0.037277222 | P20491 | High affinity immunoglobulin epsilon receptor subunit gamma | Fcer1g | 26.02922058 | 25.9362278 | 26.16031075 | 26.0458107 | 26.22727394 | 25.96450615 |
|  | 2.340811442 | 0.181069056 | Q3TRM8;E9Q3Z4;E9Q8S8;D6RFA3;D3Z2E4 | Hexokinase-3;Hexokinase | Hk3 | 26.01180077 | 25.97285461 | 25.97436523 | 25.80025482 | 25.85756874 | 25.75798988 |
|  | 2.365678968 | 0.179428736 | Q8BFR4;A0A1W2P8D3;A0A1W2P6W9 | N-acetylglucosamine-6-sulfatase | Gns | 26.01000786 | 25.90909958 | 25.98337555 | 25.79689407 | 25.79049492 | 25.77680779 |
|  | 0.148127683 | 0.057082494 | D3YTQ9;P62843 | 40S ribosomal protein S15 | Rps15 | 26.00817108 | 26.15273094 | 26.23189354 | 26.11905861 | 26.26879883 | 25.83369064 |
|  | 0.578311118 | -0.052587509 | Q9D8U8 | Sorting nexin-5 | Snx5 | 26.00210762 | 26.00361061 | 26.04095078 | 26.05306053 | 26.14102173 | 26.01034927 |
|  | 0.129384309 | -0.020031611 | P05202 | Aspartate aminotransferase, mitochondrial | Got2 | 26.00047493 | 26.09472466 | 25.95584297 | 26.00628853 | 26.11535263 | 25.98949623 |
|  | 0.847525746 | -0.073458354 | Q8VEK3 | Heterogeneous nuclear ribonucleoprotein U | Hnrnpu | 25.9975071 | 26.07232666 | 26.01962471 | 26.11600685 | 26.15402603 | 26.03980064 |
|  | 0.201789162 | 0.044898351 | Q62318 | Transcription intermediary factor 1-beta | Trim28 | 25.98211288 | 25.7727375 | 25.89451027 | 25.71774673 | 25.91006088 | 25.88685799 |
|  | 0.865709981 | 0.081168493 | Q6GT24;D3Z0Y2;O08709;Q8BG37;A0A0A6YXQ7 | Peroxiredoxin-6 | Prdx6 | 25.97521973 | 26.07496262 | 25.94661522 | 25.9321537 | 25.94237137 | 25.87876701 |
|  | 1.184040592 | -0.123116175 | P12787 | Cytochrome c oxidase subunit 5A, mitochondrial | Cox5a | 25.96496964 | 26.10789108 | 26.05046844 | 26.14590645 | 26.21515656 | 26.13161469 |
|  | 0.648083242 | -0.061413447 | Q9D8N0 | Elongation factor 1-gamma | Eef1g | 25.9642868 | 26.09333611 | 26.08362961 | 26.09407997 | 26.10225487 | 26.12915802 |
|  | 1.719661527 | 0.177588145 | Q9D1D4;A0A1Y7VM54 | Transmembrane emp24 domain-containing protein 10 | Tmed10 | 25.95905495 | 25.84467316 | 25.95863342 | 25.69609451 | 25.74360466 | 25.78989792 |
|  | 1.112326929 | -0.165246328 | Q60668;F6ZV59;G5E8G0;G3X9W0;E9Q5B6;F6SHF3;Q9D3U4 | Heterogeneous nuclear ribonucleoprotein D0 | Hnrnpd | 25.9471283 | 25.92899704 | 26.14689827 | 26.16781235 | 26.17392349 | 26.17702675 |
|  | 1.825080359 | -0.095676422 | Q02053;P31254 | Ubiquitin-like modifier-activating enzyme 1 | Uba1 | 25.94654846 | 25.89756012 | 25.88644028 | 25.98739243 | 25.99606323 | 26.03412247 |
|  | 0.58397736 | -0.103567759 | P14131 | 40S ribosomal protein S16 | Rps16 | 25.94431686 | 26.06062508 | 26.2153244 | 26.19659233 | 26.16524506 | 26.16913223 |
|  | 0.546222398 | -0.105705261 | G3UYV7;P62858 | 40S ribosomal protein S28 | Rps28 | 25.93134308 | 25.93512535 | 25.95451355 | 26.10619545 | 26.15414047 | 25.87776184 |
|  | 1.51110982 | -0.060318629 | Q922Q8 | Leucine-rich repeat-containing protein 59 | Lrrc59 | 25.9262619 | 25.94022179 | 25.92075348 | 25.96133041 | 25.98526573 | 26.02159691 |
|  | 0.786030268 | -0.093554815 | Q9CY58;Q3UMP4;A0A0N4SV32;A0A0N4SUQ1;A0A0N4SUN8;A0A0N4SWH2;A0A0N4SV40;A0A0N4SVK5 | Plasminogen activator inhibitor 1 RNA-binding protein | Serbp1 | 25.90228462 | 25.74763107 | 25.85178947 | 25.97587585 | 25.8705368 | 25.93595695 |
|  | 1.477144115 | -0.138271968 | P19253;A0A1B0GSQ6;A0A1B0GS68;A0A1B0GSB2;A0A1B0GTA1;A0A1B0GQW6;A0A1B0GSC2;A0A1B0GSF0;A0A1B0GSL5;A0A1B0GRH1;A0A1B0GT00;A0A1B0GSJ2 | 60S ribosomal protein L13a | Rpl13a | 25.89961243 | 25.99733543 | 25.8609333 | 26.06463814 | 26.07991219 | 26.02814674 |
|  | 0.553683412 | 0.030524572 | Q3TML0;F7DBQ0;Q922R8 | Protein disulfide-isomerase A6 | Pdia6 | 25.89522743 | 25.9579258 | 25.91278267 | 25.86038971 | 25.90260696 | 25.91136551 |
|  | 1.956235532 | -0.125365575 | Q8VDN2 | Sodium/potassium-transporting ATPase subunit alpha-1 | Atp1a1 | 25.88806725 | 25.89464951 | 25.92960548 | 26.07134438 | 25.98520088 | 26.0318737 |
|  | 0.486170358 | -0.053866069 | P48771 | Cytochrome c oxidase subunit 7A2, mitochondrial | Cox7a2 | 25.88522911 | 25.86116982 | 25.7684269 | 25.92073059 | 25.92840958 | 25.82728386 |
|  | 0.932792397 | -0.102541606 | A0A0J9YUZ4;P63158;A0A0J9YUD8;D3YZ18;D3YVC6 | High mobility group protein B1 | Hmgb1 | 25.87591171 | 25.83927727 | 25.84412193 | 25.99341011 | 25.85640526 | 26.01712036 |
|  | 1.564095194 | 0.08733813 | P80318;E9Q133;Q3U0I3;F6Q609;F6ZVG8 | T-complex protein 1 subunit gamma | Cct3 | 25.86109924 | 25.92998886 | 25.90694618 | 25.79337502 | 25.84345245 | 25.79919243 |
|  | 0.491291854 | -0.441890081 | P50543 | Protein S100-A11 | S100a11 | 25.85728264 | 26.80242729 | 25.72465897 | 26.77680779 | 26.17778587 | 26.75544548 |
|  | 0.347533441 | 0.038236618 | Q07076;A0A2C9F2D2;A0A286YCW4 | Annexin A7 | Anxa7 | 25.85083771 | 25.88203621 | 25.82121277 | 25.73469162 | 25.82573128 | 25.87895393 |
|  | 1.134784567 | -0.303619385 | A0A2I3BRQ3;Q61704 | Inter-alpha-trypsin inhibitor heavy chain H3 | Itih3 | 25.84450531 | 26.18457413 | 26.04710197 | 26.48437691 | 26.23994446 | 26.2627182 |
|  | 0.061351515 | -0.003183365 | Q9Z2U1;A0A0G2JF97 | Proteasome subunit alpha type-5 | Psma5 | 25.84239769 | 25.81953239 | 25.85526466 | 25.81609344 | 25.84383583 | 25.86681557 |
|  | 0.597676951 | 0.063866297 | P61089;A0A1W2P7Z3 | Ubiquitin-conjugating enzyme E2 N | Ube2n | 25.84090996 | 25.93249321 | 25.81132317 | 25.84680176 | 25.80570221 | 25.74062347 |
|  | 0.251250197 | -0.044255575 | Q6IRU2;A0A571BEU1 | Tropomyosin alpha-4 chain | Tpm4 | 25.82662964 | 26.03716278 | 25.84047699 | 25.92370224 | 25.93656349 | 25.9767704 |
|  | 0.849324903 | 0.093854268 | Q9ES97 | Reticulon-3 | Rtn3 | 25.8230114 | 25.7039814 | 25.76966286 | 25.59636497 | 25.71900177 | 25.6997261 |
|  | 0.983508672 | 0.138760885 | A2AVJ7;Q99PL5 | Ribosome-binding protein 1 | Rrbp1 | 25.82281685 | 25.93927956 | 25.97042084 | 25.84589386 | 25.68056679 | 25.78977394 |
|  | 1.911184072 | 0.083017985 | P29341;Q9D4E6;Q62029;A0A2I3BR37;F6ZAX1;V9GXG3;Q8C7D3;A2A5N3 | Polyadenylate-binding protein 1 | Pabpc1 | 25.80914116 | 25.80616951 | 25.86414909 | 25.74875641 | 25.73742867 | 25.74422073 |
|  | 1.001143648 | 0.099128723 | P07901;B7ZC50;A2A6A2;B7ZC49 | Heat shock protein HSP 90-alpha | Hsp90aa1 | 25.80784035 | 25.87335587 | 25.91472244 | 25.69731522 | 25.7972641 | 25.80395317 |
|  | 0.382941692 | 0.125776291 | Q9D1G1;A0A494B945;A0A494BA38;A0A494BBL7 | Ras-related protein Rab-1B | Rab1b | 25.79563141 | 25.68672371 | 25.76933479 | 25.87895393 | 25.42310143 | 25.57230568 |
|  | 1.019510239 | 0.083621343 | P62874;H3BKR2;H3BLF7;Q61011 | Guanine nucleotide-binding protein G(I)/G(S)/G(T) subunit beta-1 | Gnb1 | 25.78588867 | 25.79307747 | 25.77416992 | 25.66527557 | 25.66008759 | 25.77690887 |
|  | 0.069941712 | 0.003184001 | Q6ZQM8;D3YZ96;D6RH94 | UDP-glucuronosyltransferase 1-7C | Ugt1a7c | 25.78551483 | 25.79295349 | 25.80766869 | 25.76316643 | 25.80552864 | 25.80788994 |
|  | 1.941316024 | 0.090449015 | A2ACG7;Q9DBG6 | Dolichyl-diphosphooligosaccharide--protein glycosyltransferase subunit 2 | Rpn2 | 25.78419113 | 25.8202877 | 25.82660484 | 25.72382545 | 25.69117737 | 25.74473381 |
|  | 1.044373187 | 0.209859212 | Q9CQM5 | Thioredoxin domain-containing protein 17 | Txndc17 | 25.77813721 | 26.04366302 | 25.92861176 | 25.73435593 | 25.78508949 | 25.60138893 |
|  | 0.58106096 | -0.065811157 | Q8K183 | Pyridoxal kinase | Pdxk | 25.77685738 | 25.78613853 | 25.88495064 | 25.9421463 | 25.81518936 | 25.88804436 |
|  | 1.253807891 | 0.17698733 | Q5RKN9;P47753;A0A0G2JE27 | F-actin-capping protein subunit alpha-1 | Capza1 | 25.773592 | 25.7204895 | 25.64877892 | 25.47047997 | 25.64751625 | 25.49390221 |
|  | 0.018215008 | -0.002288183 | O09061;A0A338P7C0;A0A338P7F1 | Proteasome subunit beta type-1 | Psmb1 | 25.75956535 | 25.87766838 | 25.81254768 | 25.82832527 | 25.77336502 | 25.85495567 |
|  | 0.106976851 | -0.041802724 | Q06185;Q8BTB6 | ATP synthase subunit e, mitochondrial | Atp5i;Atp5k | 25.75095558 | 25.716465 | 25.51065826 | 25.73337364 | 25.89001656 | 25.48009682 |
|  | 0.427393741 | -0.118076324 | P62267 | 40S ribosomal protein S23 | Rps23 | 25.73575211 | 25.66098595 | 25.74599075 | 26.05814934 | 25.68201447 | 25.75679398 |
|  | 3.285817095 | -0.085865657 | Q9QXS1;E9Q3W4;A0A3B2W7J8;A0A0R4J218;A0A0R4J223;E9Q9J6;A0A0R4J221;E9PW24 | Plectin | Plec | 25.73551941 | 25.72772408 | 25.71871376 | 25.81967926 | 25.79946518 | 25.82040977 |
|  | 0.684377669 | -0.061640422 | Q8BTS0;Q61656;S4R1I6;B1ARB9;B1ARC0;S4R1E3 | Probable ATP-dependent RNA helicase DDX5 | Ddx5 | 25.73453712 | 25.67772102 | 25.65548134 | 25.73099327 | 25.81628799 | 25.70537949 |
|  | 1.4842406 | 0.117031733 | P29351;G3UYY5;G3UXM2;G3UZU6 | Tyrosine-protein phosphatase non-receptor type 6 | Ptpn6 | 25.72715378 | 25.65641022 | 25.69442177 | 25.60325813 | 25.51514816 | 25.60848427 |
|  | 1.019383658 | 0.069433212 | Q920A5 | Retinoid-inducible serine carboxypeptidase | Scpep1 | 25.72252274 | 25.65039635 | 25.72964478 | 25.59237671 | 25.64984894 | 25.65203857 |
|  | 0.027179605 | -0.004684448 | P43275 | Histone H1.1 | Hist1h1a | 25.72223663 | 25.80693054 | 25.63928604 | 25.68669701 | 25.70596123 | 25.78984833 |
|  | 0.561339432 | -0.078023275 | P47962;D3YYV8 | 60S ribosomal protein L5 | Rpl5 | 25.71897507 | 25.73897552 | 25.81462669 | 25.72949028 | 25.90937424 | 25.86778259 |
|  | 2.514409316 | 0.171309153 | Q9JL62;D3Z1H9;D3Z1H8 | Glycolipid transfer protein | Gltp | 25.71808624 | 25.70669746 | 25.74696541 | 25.5964489 | 25.54730797 | 25.51406479 |
|  | 1.887075147 | -0.188501358 | Q8K1B8;A0A494B9W3;A0A494BBJ8;A0A494B9F7 | Fermitin family homolog 3 | Fermt3 | 25.71384621 | 25.72208023 | 25.67812347 | 25.81249809 | 25.95409203 | 25.91296387 |
|  | 1.081764209 | 0.194603602 | Q9Z2U0;A0A338P7D7;Q9CWH6;B7ZMS4;A0A3Q4L361;A0A3Q4EHS3;A0A3Q4EG42 | Proteasome subunit alpha type-7 | Psma7 | 25.68992424 | 25.71069717 | 25.46578598 | 25.39260674 | 25.39917374 | 25.49081612 |
|  | 1.419175766 | 0.156407038 | D3Z645;Q9QZ88;D3YYD5;D3YW98 | Vacuolar protein sorting-associated protein 29 | Vps29 | 25.68768501 | 25.57487679 | 25.52219963 | 25.43049049 | 25.41602898 | 25.46902084 |
|  | 0.581899392 | 0.116909027 | D3YYM6;D3Z1S8;Q91V55;P97461 | 40S ribosomal protein S5;40S ribosomal protein S5, N-terminally processed | Rps5 | 25.68597603 | 25.72886658 | 25.7988472 | 25.71460533 | 25.4547863 | 25.69357109 |
|  | 0.121831994 | 0.027026494 | A0A494BAN1;A0A494BA56;A0A0G2JE32;A0A0G2JGL0;P62838;P61079;P61080;Q6ZWY6 | Ubiquitin-conjugating enzyme E2 D2;Ubiquitin-conjugating enzyme E2 D3;Ubiquitin-conjugating enzyme E2 D1;Ubiquitin-conjugating enzyme E2 D2B | Ube2d2;Ube2d3;Ube2d1;Ube2d2b | 25.67419434 | 25.72993088 | 25.77883911 | 25.74804115 | 25.80042839 | 25.5534153 |
|  | 0.501030199 | 0.137846629 | Q9EPN1;A0A5F8MPY6 | Neurobeachin | Nbea | 25.67090416 | 25.63981056 | 25.88278389 | 25.44774055 | 25.76602745 | 25.56619072 |
|  | 0.069408692 | -0.023762703 | A6PWS5 | Gelsolin | Gsn | 25.66435242 | 25.45779419 | 25.39597511 | 25.45929527 | NaN | 25.60031128 |
|  | 0.570949926 | 0.052933375 | P48025;E9PWE9;P43404 | Tyrosine-protein kinase SYK;Tyrosine-protein kinase | Syk | 25.65842628 | 25.71547127 | 25.75585365 | 25.70993423 | 25.60597229 | 25.65504456 |
|  | 0.137838132 | 0.011796951 | P34022;H7BX22 | Ran-specific GTPase-activating protein | Ranbp1 | 25.6542263 | 25.72608757 | 25.63879013 | 25.63292313 | 25.6905117 | 25.66027832 |
|  | 1.11785147 | 0.261140823 | Q9JHK5;Q8CAG6;Q5F270 | Pleckstrin | Plek | 25.64270592 | 25.84864235 | 25.6725769 | 25.29942894 | 25.60721588 | 25.47385788 |
|  | 0.040014923 | 0.014874776 | Q8QZY6 | Tetraspanin-14 | Tspan14 | 25.64149284 | 25.54586601 | 25.40220261 | 25.37855339 | 25.44373131 | 25.72265244 |
|  | 0.979371038 | -0.263964335 | P21460;A2APX3 | Cystatin-C | Cst3 | 25.63541603 | 25.20325661 | 25.40366554 | 25.68645668 | 25.70617104 | 25.64160347 |
|  | 1.386287313 | -0.107212067 | P62849;A0A286YEB7 | 40S ribosomal protein S24 | Rps24 | 25.63225746 | 25.71505165 | 25.72020149 | 25.82126045 | 25.75223351 | 25.81565285 |
|  | 2.765390551 | 0.129487991 | Q3U2G2;Q61316;A0A0N4SVU2 | Heat shock 70 kDa protein 4 | Hspa4 | 25.6231308 | 25.63699341 | 25.60226822 | 25.47078896 | 25.48483658 | 25.51830292 |
|  | 0.541274691 | 0.055446625 | Q9DBP5;A0A0R4J093 | UMP-CMP kinase | Cmpk1 | 25.61820984 | 25.74972916 | 25.70714569 | 25.67033577 | 25.59155083 | 25.64685822 |
|  | 0.957315555 | -0.158588409 | P63276 | 40S ribosomal protein S17 | Rps17 | 25.61627579 | 25.46979713 | 25.6996479 | 25.75880241 | 25.68437004 | 25.8183136 |
|  | 1.664731505 | 0.079282761 | Q9JHU4;F6ZX84 | Cytoplasmic dynein 1 heavy chain 1 | Dync1h1 | 25.61619186 | 25.58451462 | 25.55999184 | 25.52749062 | 25.51556969 | 25.47978973 |
|  | 0.37170062 | 0.034250259 | P80315;G5E839;G3UYW5;G3UXF3;G3UXG2 | T-complex protein 1 subunit delta | Cct4 | 25.59690475 | 25.55929184 | 25.57383728 | 25.47072792 | 25.59414482 | 25.56241035 |
|  | 1.114276001 | 0.078335444 | Q8VDM4;A0A338P6M5;E9Q2S8;A0A338P7H4;A0A338P6S9 | 26S proteasome non-ATPase regulatory subunit 2 | Psmd2 | 25.58783531 | 25.58674812 | 25.6257515 | 25.5213623 | 25.46920586 | 25.57476044 |
|  | 0.044772843 | -0.008073171 | Q9DCX2;B1ASE2 | ATP synthase subunit d, mitochondrial | Atp5h | 25.58657646 | 25.64485168 | 25.69498062 | 25.56549454 | 25.63729858 | 25.74783516 |
|  | 0.813137195 | 0.141727448 | Q8C2Q8;Q91VR2;A2AKU9;A2AKV1;A2AKV2;A2AKV3;A2AKV0 | ATP synthase subunit gamma;ATP synthase subunit gamma, mitochondrial | Atp5c1 | 25.58560371 | 25.72444916 | 25.73541641 | 25.63525009 | 25.41664124 | 25.56839561 |
|  | 0.035594067 | 0.005653381 | Q52KG9;P80317;Q61390;B1AT05 | T-complex protein 1 subunit zeta | Cct6a | 25.56054688 | 25.47797012 | 25.42860603 | 25.52390671 | 25.51785278 | 25.4084034 |
|  | 1.424144363 | 0.103642146 | Q8R1F1;A2ARS6 | Niban-like protein 1 | Fam129b | 25.55476189 | 25.6378231 | 25.55291748 | 25.4963131 | 25.49817085 | 25.44009209 |
|  | 0.045849068 | 0.008625031 | Q8VIJ6 | Splicing factor, proline- and glutamine-rich | Sfpq | 25.55420685 | 25.44830894 | 25.55262375 | 25.59807014 | 25.41193008 | 25.51926422 |
|  | 0.881361518 | -0.045499802 | Q9R1P4;A0A1B0GS70 | Proteasome subunit alpha type-1 | Psma1 | 25.54974937 | 25.53546143 | 25.602211 | 25.6312027 | 25.60608482 | 25.58663368 |
|  | 0.176526062 | -0.048498154 | Q8VEH3;F6QKK2 | ADP-ribosylation factor-like protein 8A | Arl8a | 25.54512978 | 25.58448601 | 25.66511345 | 25.50260735 | 25.83407593 | 25.60354042 |
|  | 0.732810453 | -0.151580811 | P01887 | Beta-2-microglobulin | B2m | 25.53851318 | 25.83173561 | 25.57299805 | 25.83006668 | 25.80754471 | 25.76037788 |
|  | 1.502813694 | -0.180134455 | Q9CQR2 | 40S ribosomal protein S21 | Rps21 | 25.53002357 | 25.60800552 | 25.54271126 | 25.64650154 | 25.75793839 | 25.8167038 |
|  | 1.19752686 | 0.117687861 | Q9WUM3;A0A494B9Y4;D3YUG6;A0A494BAI1 | Coronin-1B | Coro1b | 25.52948761 | 25.43834877 | 25.5142746 | 25.32892609 | 25.44833946 | 25.35178185 |
|  | 0.007351842 | 0.001026789 | G3UZP7;P01899;P01897;Q8HWB2;P01900;P01898;P01896;G3UXE9;E9Q0G4;O19441;A7VMS6;E9PX63;Q4KN81;P14427;P01895;V9GXR0;V9GXI4 | H-2 class I histocompatibility antigen, D-B alpha chain;H-2 class I histocompatibility antigen, L-D alpha chain | H2-D1;H2-L | 25.51821327 | 25.66419029 | 25.57181358 | 25.57626152 | 25.61605072 | 25.55882454 |
|  | 0.163851394 | -0.038671494 | O88958;A0A494B9X2;D3Z0R5;A0A494BB68;A0A494BA15 | Glucosamine-6-phosphate isomerase 1 | Gnpda1 | 25.51749229 | 25.52951622 | 25.33606339 | 25.50060463 | 25.60840034 | 25.39008141 |
|  | 0.08807647 | 0.022765477 | Q9EQU5;A2BE93;A2BE92 | Protein SET | Set | 25.51415443 | 25.4056797 | 25.67039108 | 25.6037674 | 25.48397636 | 25.43418503 |
|  | 1.167178974 | -0.091797511 | O70503 | Very-long-chain 3-oxoacyl-CoA reductase | Hsd17b12 | 25.51074791 | 25.55112839 | 25.56621933 | 25.64652824 | 25.68479919 | 25.57216072 |
|  | 0.949623895 | 0.105486552 | Q3U8S1;A2APM5;A2APM3;A2APM4;E9QKM8;Q80X37;A2APM1;A2APM2;P15379 | CD44 antigen | Cd44 | 25.50051308 | 25.47153473 | 25.50914764 | 25.46435356 | 25.29210854 | 25.4082737 |
|  | 0.930386217 | 0.19994545 | Q9EST5 | Acidic leucine-rich nuclear phosphoprotein 32 family member B | Anp32b | 25.49594688 | 25.51517868 | 25.35890579 | 25.37630272 | 25.30778885 | 25.08610344 |
|  | 0.680548707 | -0.094649633 | Q9JLJ2 | 4-trimethylaminobutyraldehyde dehydrogenase | Aldh9a1 | 25.4879055 | 25.41625404 | 25.37218857 | 25.53380013 | 25.60509682 | 25.42140007 |
|  | 0.048053959 | 0.011862437 | Q20BD0;Q80XR6;Q99020 | Heterogeneous nuclear ribonucleoprotein A/B | Hnrnpab | 25.4845295 | 25.41493225 | 25.61231422 | 25.38521767 | 25.49179459 | 25.59917641 |
|  | 1.248966725 | 0.106297175 | O35129;F6QPR1;F6Q8V7 | Prohibitin-2 | Phb2 | 25.47824669 | 25.51068878 | 25.56880188 | 25.42140007 | 25.46001434 | 25.35743141 |
|  | 0.982408405 | 0.113379796 | G3UZ60;G3UWI9;G3UWX9;Q9Z172;G3UZA7 | Small ubiquitin-related modifier 3;Small ubiquitin-related modifier | Sumo3 | 25.47580719 | 25.49038696 | 25.56584167 | 25.45202255 | 25.30514717 | 25.43472672 |
|  | 0.249238037 | 0.050904592 | Q9CY64;A2ASB1;A2ASB8;A2ASB7 | Biliverdin reductase A | Blvra | 25.47271156 | 25.54510117 | 25.28245544 | 25.38830948 | 25.41431999 | 25.34492493 |
|  | 0.853978075 | 0.073733012 | P09405 | Nucleolin | Ncl | 25.46945572 | 25.44688988 | 25.49564171 | 25.41496468 | 25.32474518 | 25.45107841 |
|  | 0.338468061 | -0.017275492 | Q9JJU8 | SH3 domain-binding glutamic acid-rich-like protein | Sh3bgrl | 25.46291924 | 25.43103218 | 25.45211601 | 25.4708519 | 25.43106461 | 25.4959774 |
|  | 0.280261338 | -0.149051666 | Q61703;G3X977;F6SGM1 | Inter-alpha-trypsin inhibitor heavy chain H2 | Itih2 | 25.46229553 | 25.13719559 | 25.23954391 | 25.81129837 | 25.23509407 | 25.23979759 |
|  | 3.33267671 | 0.161462148 | P80316;E0CZA1 | T-complex protein 1 subunit epsilon | Cct5 | 25.45324707 | 25.49484825 | 25.49332237 | 25.32024002 | 25.33070564 | 25.30608559 |
|  | 1.164781079 | 0.196711858 | P48024 | Eukaryotic translation initiation factor 1 | Eif1 | 25.44837189 | 25.41367531 | 25.20590401 | 25.18665314 | 25.17968559 | 25.1114769 |
|  | 0.362269662 | 0.043645859 | P00493 | Hypoxanthine-guanine phosphoribosyltransferase | Hprt1 | 25.43087196 | 25.49280357 | 25.47880173 | 25.49243546 | 25.33480263 | 25.44430161 |
|  | 0.696264746 | -0.13494873 | A0A498WGD8;Q8CDN6;A0A494B955;A0A494BBI1;A0A494BB31 | Thioredoxin-like protein 1 | Txnl1 | 25.43052101 | 25.2602253 | 25.28408051 | 25.46640778 | 25.33469963 | 25.5785656 |
|  | 0.998782065 | 0.244363149 | P56391;A0A140LIU3 | Cytochrome c oxidase subunit 6B1 | Cox6b1 | 25.41583633 | 25.57146645 | 25.53388977 | 25.38798141 | 25.05465508 | 25.34546661 |
|  | 0.899927679 | -0.046089172 | E9Q3X0;Q9EQK5;D3Z2N7 | Major vault protein | Mvp | 25.41538429 | 25.45312119 | 25.49451256 | 25.4952755 | 25.49179459 | 25.51421547 |
|  | 1.658012736 | -0.191226959 | P07141;D3Z090;D3YTW1;F6RNW8 | Macrophage colony-stimulating factor 1;Processed macrophage colony-stimulating factor 1 | Csf1 | 25.40820885 | 25.46229553 | 25.35947609 | 25.58072472 | 25.68442345 | 25.53851318 |
|  | 1.23134935 | -0.158953985 | Q60692 | Proteasome subunit beta type-6 | Psmb6 | 25.40480423 | 25.56302261 | 25.53798103 | 25.63466835 | 25.61672401 | 25.73127747 |
|  | 1.535890453 | -0.13288943 | Q9CWK8 | Sorting nexin-2 | Snx2 | 25.39718437 | 25.52034378 | 25.49823189 | 25.59965897 | 25.62853622 | 25.58623314 |
|  | 1.566710543 | -0.187178294 | Q9CPQ1 | Cytochrome c oxidase subunit 6C | Cox6c | 25.39659691 | 25.56019592 | 25.43332672 | 25.61705971 | 25.69643974 | 25.63815498 |
|  | 1.045013413 | 0.110811234 | O54734 | Dolichyl-diphosphooligosaccharide--protein glycosyltransferase 48 kDa subunit | Ddost | 25.39473343 | 25.46142006 | 25.29557991 | 25.27184677 | 25.29561615 | 25.25183678 |
|  | 0.740412127 | 0.07946523 | A0A3Q4EI12;P35293;A0A452J8C1;A0A3Q4EIF7 | Ras-related protein Rab-18 | Rab18 | 25.37871742 | 25.41338348 | 25.45512962 | 25.24843788 | 25.37570572 | 25.38469124 |
|  | 1.207035143 | 0.109560013 | Q63844;D3Z3G6;A0A0U1RPX4;D3Z6D8;A0A0U1RPZ0;A0A0E2WHU5 | Mitogen-activated protein kinase 3;Mitogen-activated protein kinase | Mapk3 | 25.36188698 | 25.29852104 | 25.40778732 | 25.21567535 | 25.22025871 | 25.30358124 |
|  | 0.743365546 | -0.049147288 | P42932;H3BL49;H3BJB6;H3BKG2;H3BLL1;H3BKR8 | T-complex protein 1 subunit theta | Cct8 | 25.36084938 | 25.38205338 | 25.34516335 | 25.45885849 | 25.41589928 | 25.3607502 |
|  | 0.451711357 | -0.072146734 | Q9D0J8 | Parathymosin | Ptms | 25.35940933 | 25.22394562 | 25.38801384 | 25.4889183 | 25.34414673 | 25.35474396 |
|  | 1.954727046 | 0.191934586 | Q99LC5 | Electron transfer flavoprotein subunit alpha, mitochondrial | Etfa | 25.35763168 | 25.41950417 | 25.37507629 | 25.24829292 | 25.11763382 | 25.21048164 |
|  | 1.931317778 | -0.132965088 | Q9CQQ7;A0A0G2JGX3 | ATP synthase F(0) complex subunit B1, mitochondrial | Atp5f1 | 25.35507965 | 25.31741333 | 25.41390038 | 25.48793602 | 25.48025131 | 25.51710129 |
|  | 0.64111145 | -0.078344345 | Q9Z0N1;A2AAW9 | Eukaryotic translation initiation factor 2 subunit 3, X-linked | Eif2s3x | 25.35494423 | 25.43010712 | 25.42146492 | 25.46422768 | 25.57372093 | 25.40360069 |
|  | 1.712875506 | 0.114014308 | Q6ZWQ5;Q3TGS7;Q3V2H3;O70493 | Sorting nexin-12 | Snx12 | 25.35407066 | 25.41567421 | 25.342453 | 25.24430656 | 25.23037338 | 25.29547501 |
|  | 0.101013731 | -0.019543966 | A0A087WR50;A0A087WS56;Q3UHL6;P11276;A0A087WSN6;B7ZNJ1;B9EHT6;Q4KL80;A0A087WS99;A0A087WSU6;A0A087WQE0;A0A087WQW8 | Fibronectin;Anastellin | Fn1 | 25.34915161 | 25.43837929 | 25.42678452 | 25.4279995 | 25.31236649 | 25.53258133 |
|  | 0.338310925 | -0.039346695 | G3UVV4;P17710;D3YYR4;D3Z365;D3Z105;B4YB29 | Hexokinase;Hexokinase-1 | Hk1 | 25.32573891 | 25.31575584 | 25.23040962 | 25.29624557 | 25.40454292 | 25.28915596 |
|  | 0.482705296 | 0.075166066 | A0A0R4J0Z1;P08003 | Protein disulfide-isomerase A4 | Pdia4 | 25.30542564 | 25.24807549 | 25.33800316 | 25.32738495 | 25.1114769 | 25.22714424 |
|  | 0.264643247 | 0.032354355 | P51660 | Peroxisomal multifunctional enzyme type 2;(3R)-hydroxyacyl-CoA dehydrogenase;Enoyl-CoA hydratase 2 | Hsd17b4 | 25.29438972 | 25.40499878 | 25.32299232 | 25.30931664 | 25.37006187 | 25.24593925 |
|  | 0.2762761 | -0.033738454 | B7ZCL8;P70290;A2AN84;B7ZCL9;B7ZCM0;B7ZCM1;D6RFD5 | 55 kDa erythrocyte membrane protein | Mpp1 | 25.27923393 | 25.15429497 | 25.16599274 | 25.17877388 | 25.24677277 | 25.27519035 |
|  | 1.320274784 | 0.102174759 | P61226 | Ras-related protein Rap-2b | Rap2b | 25.27102661 | 25.30702591 | 25.36091614 | 25.1631546 | 25.22092438 | 25.2483654 |
|  | 0.622100948 | -0.077334722 | Q9JKR6;A0A1L1SQ34;E0CYZ2;F6TRP3 | Hypoxia up-regulated protein 1 | Hyou1 | 25.25904083 | 25.17279816 | 25.12360573 | 25.34109497 | 25.22945786 | 25.21689606 |
|  | 3.524915622 | 0.070794423 | P14824;F8WIT2 | Annexin A6;Annexin | Anxa6 | 25.25691986 | 25.25648689 | 25.25731468 | 25.17691231 | 25.19741821 | 25.18400764 |
|  | 0.208254778 | 0.036233902 | A0A1D5RLW5;A0A1D5RM85;P62717;A0A1D5RM79;A0A1D5RME4;A0A1D5RMC7 | 60S ribosomal protein L18a | Rpl18a | 25.25623512 | 25.32680321 | 25.32765961 | 25.39253998 | 25.19182014 | 25.21763611 |
|  | 1.247143215 | 0.301961263 | Q9QUH0;A0A1Y7VM65 | Glutaredoxin-1 | Glrx | 25.24952316 | 25.32683754 | 25.09703827 | 24.91929817 | 25.08249283 | 24.76572418 |
|  | 2.130557908 | -0.092734655 | Q9JMH6;A0A1W2P6U1;Q9D8I4;A0A0U1RPS1;A0A0U1RPC6;A0A0M3HEP9 | Thioredoxin reductase 1, cytoplasmic | Txnrd1 | 25.24176407 | 25.24252701 | 25.21656418 | 25.35494423 | 25.29813576 | 25.32597923 |
|  | 0.001548501 | 0.000188192 | Q8BH80;Q9QY76 | Vesicle-associated membrane protein-associated protein B | Vapb | 25.23516655 | 25.3569603 | 25.26291466 | 25.30834579 | 25.28816986 | 25.25796127 |
|  | 0.428915358 | 0.041009267 | P11983;F2Z483;A0A3B2WDE2 | T-complex protein 1 subunit alpha | Tcp1 | 25.2309227 | 25.32085991 | 25.36823082 | 25.25259399 | 25.26781654 | 25.27657509 |
|  | 0.236450502 | 0.054560979 | Q3UW53;E9PYV4;D3Z233;D3YYZ9 | Protein Niban | Fam129a | 25.23044586 | 25.38334084 | 25.48111534 | 25.40200806 | 25.21500969 | 25.31420135 |
|  | 1.14219596 | 0.031058629 | Q8VCT3;E9PYF1 | Aminopeptidase B | Rnpep | 25.22883415 | 25.24923515 | 25.20780373 | 25.19767952 | 25.18967247 | 25.20534515 |
|  | 0.338635449 | 0.02245903 | E9PUA7;D3Z637;F8WHQ1;Q62393;D3Z125;D3Z7X7;D3Z2U2 | Tumor protein D52 | Tpd52 | 25.2277317 | 25.30344009 | 25.22332001 | 25.23399734 | 25.24092674 | 25.21219063 |
|  | 0.506132079 | -0.07178688 | Q9CWJ9 | Bifunctional purine biosynthesis protein PURH;Phosphoribosylaminoimidazolecarboxamide formyltransferase;IMP cyclohydrolase | Atic | 25.22188187 | 25.15514565 | 25.02445221 | 25.19185829 | 25.24894524 | 25.17603683 |
|  | 1.048979776 | 0.115545909 | G8JL74;G3UXA6;Q8BHD7;G3UZ01;G3UY95;F7C521;G3UXZ8 | Polypyrimidine tract-binding protein 3 | Ptbp3 | 25.21115112 | 25.20474815 | 25.17900276 | 25.02051544 | 25.18343925 | 25.04430962 |
|  | 0.382001619 | 0.062711716 | P63005;Q5SW16 | Platelet-activating factor acetylhydrolase IB subunit alpha | Pafah1b1 | 25.21059418 | 25.16855812 | 25.38502121 | 25.23033714 | 25.17672157 | 25.16897964 |
|  | 0.73306693 | 0.085196177 | Q9JHF7 | Hematopoietic prostaglandin D synthase | Hpgds | 25.19629288 | 25.15611076 | 25.03489876 | 25.01384544 | 25.03115845 | 25.08670998 |
|  | 1.103395083 | -0.062762578 | Q921H8;H3BKL5;H3BJZ9;Q8VCH0;H3BKA1;H3BJC1;H3BJG8 | 3-ketoacyl-CoA thiolase A, peroxisomal;3-ketoacyl-CoA thiolase B, peroxisomal | Acaa1a;Acaa1b | 25.19373894 | 25.11465836 | 25.17942047 | 25.23293686 | 25.23968887 | 25.20347977 |
|  | 1.740257064 | 0.145397822 | P42208;E9Q3V6;F6WYM0;D3YYB1;D3Z3C0;D3Z1S1;F6UKN5;D3YZU7;D3YV76;G3UYQ0 | Septin-2 | Septin2 | 25.19042587 | 25.25049973 | 25.27141953 | 25.10769272 | 25.03615761 | 25.13230133 |
|  | 0.041696338 | -0.022325516 | Q8R1I1 | Cytochrome b-c1 complex subunit 9 | Uqcr10 | 25.18672943 | 25.44002914 | 25.1315155 | 25.0807457 | 25.5828495 | 25.16165543 |
|  | 0.183563636 | -0.022496541 | D3YWR7;Q8BVI4;A0A0G2JGY0;A0A0G2JGJ1;D3Z1A1;D3Z099 | Dihydropteridine reductase | Qdpr | 25.17645645 | 25.20728302 | 25.26885223 | 25.19084167 | 25.31482315 | 25.2144165 |
|  | 0.181691226 | -0.030757268 | Q9D0E1;B8JK33;B8JK32;F6W322;A0A3Q4EH91;F7C9U3;A0A3Q4L2X3 | Heterogeneous nuclear ribonucleoprotein M | Hnrnpm | 25.17340851 | 25.31868935 | 25.20026588 | 25.35407066 | 25.22346687 | 25.20709801 |
|  | 0.478481559 | -0.097156525 | P40240 | CD9 antigen | Cd9 | 25.16725731 | 25.23169136 | 25.3403492 | 25.23341179 | 25.3176899 | 25.47966576 |
|  | 0.884504625 | -0.073226293 | P53994;Q3TEG7;P59279;G3UXQ7;G3V022;A0A1D5RMH1 | Ras-related protein Rab-2A;Ras-related protein Rab-2B | Rab2a;Rab2b | 25.16257858 | 25.11179543 | 25.09948921 | 25.13516045 | 25.20925522 | 25.24912643 |
|  | 0.506506315 | 0.118015925 | P70195 | Proteasome subunit beta type-7 | Psmb7 | 25.16054153 | 25.08070564 | 25.39404678 | 25.15564728 | 25.10529518 | 25.02030373 |
|  | 0.687467434 | -0.078926086 | B1AQF4;Q9D7X3;Q3V2Y9;H3BKL8;H3BKD1 | Dual specificity protein phosphatase 3 | Dusp3 | 25.15973282 | 25.15695953 | 25.27056313 | 25.22217751 | 25.25709915 | 25.34475708 |
|  | 0.718917652 | -0.061396281 | Q8CG29;P70248;G3V011;G3UZR3 | Unconventional myosin-If | Myo1f | 25.1590786 | 25.08857155 | 25.164114 | 25.22677803 | 25.13770294 | 25.23147202 |
|  | 1.329549945 | 0.131912231 | Q8K124 | Pleckstrin homology domain-containing family O member 2 | Plekho2 | 25.15834618 | 25.20086288 | 25.2047863 | 25.0044899 | 25.02017593 | 25.14359283 |
|  | 0.91258051 | 0.158572515 | O89017 | Legumain | Lgmn | 25.15711403 | 25.10189438 | 24.9671917 | 24.80498886 | 25.00135612 | 24.94413757 |
|  | 0.176424807 | 0.030881882 | P61164;A0A494BB86;A0A494BAH0 | Alpha-centractin | Actr1a | 25.1532917 | 25.10641479 | 25.21515656 | 25.04784966 | 25.09293175 | 25.241436 |
|  | 0.789246996 | -0.067495346 | P32020 | Non-specific lipid-transfer protein | Scp2 | 25.15294266 | 25.18325043 | 25.15568542 | 25.26449203 | 25.15514565 | 25.27472687 |
|  | 1.523801445 | -0.094104767 | Q3TF41;Q8BSH9;P28656 | Nucleosome assembly protein 1-like 1 | Nap1l1 | 25.15174294 | 25.12636566 | 25.17321777 | 25.28887367 | 25.20198631 | 25.24278069 |
|  | 0.263262896 | -0.093654633 | Q9CQS8;E9PW43 | Protein transport protein Sec61 subunit beta | Sec61b;Gm10320 | 25.14845085 | 25.0679245 | 24.71156311 | 25.04447746 | 25.0053463 | 25.1590786 |
|  | 0.407744777 | -0.072954178 | P61961;H7BWZ1;D3YW97 | Ubiquitin-fold modifier 1 | Ufm1 | 25.13484764 | 25.03380585 | 25.04489326 | 25.13848495 | 25.02778816 | 25.26613617 |
|  | 0.104223078 | 0.046042124 | O54965;Q8C4F9;Q8CB78;A0A0G2JEM4;A0A0G2JGT7 | E3 ubiquitin-protein ligase RNF13 | Rnf13 | 25.13183022 | 25.33827591 | 24.85552597 | 25.12392044 | 25.15189934 | 24.91168594 |
|  | 0.648647838 | 0.101807912 | P70168 | Importin subunit beta-1 | Kpnb1 | 25.13029861 | 25.05858231 | 24.94503021 | 25.03489876 | 24.89328384 | 24.90030479 |
|  | 1.613278713 | 0.21810468 | Q6P069 | Sorcin | Sri | 25.12900162 | 25.05668259 | 25.08160019 | 24.77052116 | 24.97193527 | 24.87051392 |
|  | 0.897925618 | -0.022481283 | Q78PY7;Q3TJ56;E9Q3E9 | Staphylococcal nuclease domain-containing protein 1 | Snd1 | 25.11707878 | 25.11565018 | 25.11925697 | 25.15842438 | 25.11842537 | 25.14258003 |
|  | 1.823682482 | 0.193742116 | Q6PB66;F6V2A3 | Leucine-rich PPR motif-containing protein, mitochondrial | Lrpprc | 25.10793114 | 24.97587585 | 25.03389168 | 24.83865166 | 24.80069923 | 24.89712143 |
|  | 1.0526496 | 0.216791789 | P70158;D3Z1B4 | Acid sphingomyelinase-like phosphodiesterase 3a | Smpdl3a | 25.10769272 | 25.04909706 | 25.28132248 | 24.92904091 | 24.81313515 | 25.04556084 |
|  | 0.553707729 | 0.101957321 | P61750;E9Q798;F6UFB9;D3YV25;E9Q2C2 | ADP-ribosylation factor 4 | Arf4 | 25.10717392 | 25.16134834 | 25.16007996 | 25.07001495 | 24.89082909 | 25.16188622 |
|  | 1.494025856 | -0.163768768 | P80313;A0A0N4SV00;A0A0N4SUI8;A0A0N4SV22 | T-complex protein 1 subunit eta | Cct7 | 25.09848404 | 25.18869209 | 25.13735199 | 25.37550735 | 25.22567558 | 25.31465149 |
|  | 0.462220973 | 0.088780085 | Q9DAS9;A0A0N4SW28;A0A0N4SVT3 | Guanine nucleotide-binding protein G(I)/G(S)/G(O) subunit gamma-12 | Gng12 | 25.08314323 | 25.0819664 | 25.16875076 | 24.89887619 | 25.00234413 | 25.16629982 |
|  | 1.217583389 | -0.072638194 | Q9Z2X1;J3QMT0;J3QM80;J3QP45;J3QNH2;J3QMV8 | Heterogeneous nuclear ribonucleoprotein F;Heterogeneous nuclear ribonucleoprotein F, N-terminally processed | Hnrnpf | 25.07810211 | 25.11366463 | 25.08212852 | 25.13084793 | 25.14647102 | 25.21449089 |
|  | 0.510126944 | -0.06227239 | P99026 | Proteasome subunit beta type-4 | Psmb4 | 25.07585907 | 25.12915802 | 25.13750839 | 25.07834625 | 25.2099247 | 25.2410717 |
|  | 0.420304244 | -0.083903631 | Q61166 | Microtubule-associated protein RP/EB family member 1 | Mapre1 | 25.07349205 | 24.9321537 | 24.85775757 | 25.14914894 | 25.00551796 | 24.96044731 |
|  | 1.688070842 | -0.203371684 | P19096;A0A0U1RNJ1;A0A0U1RPP5 | Fatty acid synthase;[Acyl-carrier-protein] S-acetyltransferase;[Acyl-carrier-protein] S-malonyltransferase;3-oxoacyl-[acyl-carrier-protein] synthase;3-oxoacyl-[acyl-carrier-protein] reductase;3-hydroxyacyl-[acyl-carrier-protein] dehydratase;Enoyl-[acyl-carrier-protein] reductase;Oleoyl-[acyl-carrier-protein] hydrolase | Fasn | 25.06829262 | 25.01333427 | 25.00418854 | 25.29610634 | 25.1314373 | 25.26838684 |
|  | 1.160415294 | 0.161057154 | D3Z7P3;D3Z7P4;F7B327;F6U529;F6RDM4 | Glutaminase kidney isoform, mitochondrial | Gls | 25.06431007 | 24.91346741 | 24.84627724 | 24.75999641 | 24.78895187 | 24.79193497 |
|  | 0.049771151 | 0.010461171 | Q8C2Q7;O35737 | Heterogeneous nuclear ribonucleoprotein H;Heterogeneous nuclear ribonucleoprotein H, N-terminally processed | Hnrnph1 | 25.06266403 | 25.04984474 | 25.18332481 | 25.07891655 | 24.99213409 | 25.19339943 |
|  | 0.681816523 | 0.188443502 | Q9D1M0 | Protein SEC13 homolog | Sec13 | 25.05017662 | 25.01699257 | 25.00761414 | 24.72239304 | 25.08614349 | 24.70091629 |
|  | 1.308328226 | -0.229471207 | Q07797;E9Q5X5 | Galectin-3-binding protein | Lgals3bp | 25.04992867 | 25.23783112 | 25.27710724 | 25.36422729 | 25.38656616 | 25.50248718 |
|  | 0.031198367 | -0.003848394 | Q9WU78 | Programmed cell death 6-interacting protein | Pdcd6ip | 25.04376793 | 25.12549782 | 25.12380219 | 25.11517334 | 25.14821815 | 25.04122162 |
|  | 0.678343854 | 0.163948695 | Q60631;B1AT92;B1AT95 | Growth factor receptor-bound protein 2 | Grb2 | 25.04105377 | 25.25896835 | 24.94225883 | 25.02470589 | 24.82842255 | 24.89730644 |
|  | 0.486796098 | 0.101472219 | Q9Z0P5;A0A1L1SU53;A0A1L1STC8;A0A087WRG4 | Twinfilin-2 | Twf2 | 25.0309906 | 24.98528862 | 24.8033638 | 24.88357544 | 24.90889359 | 24.72275734 |
|  | 0.238170786 | 0.118204753 | P70245;A2AC29 | 3-beta-hydroxysteroid-Delta(8),Delta(7)-isomerase | Ebp | 25.02951622 | 24.75276947 | 24.6378231 | 24.98659134 | 24.62527847 | 24.45362473 |
|  | 0.411633815 | 0.226469676 | Q3THW5;P0C0S6;Q8R029;Q3UA95 | Histone H2A.V;Histone H2A.Z;Histone H2A | H2afv;H2afz | 25.02669144 | 25.32529449 | 25.47398186 | 24.72769928 | 25.02301407 | 25.39584541 |
|  | 0.242173279 | 0.072952906 | Q99KJ8 | Dynactin subunit 2 | Dctn2 | 25.02643776 | 24.97320557 | 24.84895325 | 24.80040359 | 25.08711624 | 24.74221802 |
|  | 0.632628136 | -0.060391108 | Q6ZWX6 | Eukaryotic translation initiation factor 2 subunit 1 | Eif2s1 | 25.02271843 | 25.1510849 | 25.10385513 | 25.18869209 | 25.11565018 | 25.15448952 |
|  | 1.677157281 | 0.22739474 | A0A1W2P7X0;E9QMV2;Q4KML4 | Costars family protein ABRACL | Abracl | 25.02009201 | 25.17481804 | 25.17835617 | 24.92188835 | 24.83200073 | 24.93719292 |
|  | 0.063226823 | 0.00838534 | O55143;Q8R429 | Sarcoplasmic/endoplasmic reticulum calcium ATPase 2 | Atp2a2 | 25.02000618 | 25.06163406 | 25.09518814 | 25.00633049 | 25.01358986 | 25.13175201 |
|  | 1.743270376 | -0.193876266 | P97797;A2ANC1;A0A0A6YWR3;Q6F5F2;A0A0A6YYP6 | Tyrosine-protein phosphatase non-receptor type substrate 1 | Sirpa | 25.01979446 | 24.90380096 | 24.9306221 | 25.14810181 | 25.20594215 | 25.08180237 |
|  | 1.622866824 | -0.161483129 | O35604 | Niemann-Pick C1 protein | Npc1 | 25.01839447 | 24.91150284 | 24.89786148 | 25.13653183 | 25.1204834 | 25.05519295 |
|  | 1.875752079 | -0.19920667 | A0A0R4J1L2;Q91VK2;F6ZFU0;D3YY68;D3Z7N2 |  | Eef1d | 25.01512146 | 25.03686905 | 24.93629456 | 25.22964096 | 25.23246193 | 25.12380219 |
|  | 0.126562388 | 0.198846181 | E9QQ10;Q70FJ1;A0A571BE77 | A-kinase anchor protein 9 | Akap9 | 25.01124573 | 25.04651833 | 25.10565567 | 24.07332802 | 25.97690201 | 24.51665115 |
|  | 0.20323494 | 0.014268875 | G3UXZ5;P97371;G3UXY0;G3X9K9;G3UWN9;G3UXR1 | Proteasome activator complex subunit 1 | Psme1 | 24.99291229 | 24.9615078 | 25.01792717 | 25.00915337 | 24.93562126 | 24.98476601 |
|  | 1.321644879 | -0.149184545 | Q09014;F8WH69;S4R293 | Neutrophil cytosol factor 1 | Ncf1 | 24.98949623 | 25.0735321 | 25.09418106 | 25.12344742 | 25.26756668 | 25.21374893 |
|  | 0.776255413 | -0.086516698 | P05201;F7ALS6 | Aspartate aminotransferase, cytoplasmic | Got1 | 24.98415756 | 24.85024071 | 24.89869118 | 24.95593262 | 25.06328201 | 24.97342491 |
|  | 0.964771862 | 0.0775973 | P57746 | V-type proton ATPase subunit D | Atp6v1d | 24.98333168 | 24.96534348 | 24.87459946 | 24.89286804 | 24.83422089 | 24.86339378 |
|  | 0.10125355 | -0.00935936 | E9Q3Q6;Q61490;E9Q4G8;F6QH25;Q5MPX5 | CD166 antigen | Alcam | 24.98228836 | 25.0945034 | 25.03208351 | 25.03938103 | 25.03795815 | 25.05961418 |
|  | 0.879590173 | 0.063374837 | A0A0R4J0I9;Q91ZX7;Q3U5J2;D3Z5M3 | Prolow-density lipoprotein receptor-related protein 1;Low-density lipoprotein receptor-related protein 1 85 kDa subunit;Low-density lipoprotein receptor-related protein 1 515 kDa subunit;Low-density lipoprotein receptor-related protein 1 intracellular domain | Lrp1 | 24.98002243 | 24.99912071 | 25.02529716 | 24.94761848 | 24.88049698 | 24.98620033 |
|  | 0.298320764 | 0.046403249 | Q9QXT0;A0A1W2P729 | Protein canopy homolog 2 | Cnpy2 | 24.97543716 | 24.92025375 | 24.95446968 | 24.88269043 | 24.80994987 | 25.01831055 |
|  | 0.984943181 | 0.159648895 | E9Q1G8;E9Q9F5;A0A0R4J233;A0A1W2P6J7;Q8C650 | Septin | Septin10 | 24.97469521 | 25.03762436 | 24.90985489 | 24.77811241 | 24.72187233 | 24.94324303 |
|  | 0.939045841 | 0.157965978 | Q9JIF7 | Coatomer subunit beta | Copb1 | 24.97145271 | 24.94797516 | 24.90967178 | 24.70382309 | 24.93813515 | 24.71324348 |
|  | 0.525608066 | 0.128091176 | Q99JZ4;P36536;A0A1W2P869;A0A1W2P6N3;A0A1W2P720 | GTP-binding protein SAR1a | Sar1a | 24.97057533 | 24.69013786 | 24.96706009 | 24.84570313 | 24.66150284 | 24.73629379 |
|  | 0.055256872 | -0.007645289 | Q8C483;P26638;A2AFS0;A2AFS1 | Serine--tRNA ligase, cytoplasmic | Sars | 24.9591217 | 24.93719292 | 24.98546219 | 24.92401886 | 24.92116165 | 25.05953217 |
|  | 0.069919132 | 0.017452876 | Q9R1P3 | Proteasome subunit beta type-2 | Psmb2 | 24.95721817 | 24.86599159 | 24.79605103 | 24.98806572 | 24.84579849 | 24.73303795 |
|  | 0.336417563 | 0.094554901 | E9PY39;Q99LQ7 | Predicted gene 20431 | Gm20431 | 24.95482445 | 24.67502975 | 24.76607704 | 24.72956657 | 24.55156898 | 24.83113098 |
|  | 1.264953621 | 0.143477758 | Q9DBG3;H3BKM0;H3BIY9;H3BJ06;Q5SWR0 | AP-2 complex subunit beta;AP complex subunit beta | Ap2b1 | 24.95433617 | 24.82371712 | 24.97066307 | 24.80153847 | 24.72108841 | 24.7956562 |
|  | 0.39887701 | 0.138701757 | P97825 | Hematological and neurological expressed 1 protein;Hematological and neurological expressed 1 protein, N-terminally processed | Hn1 | 24.93992996 | 24.70229149 | 24.8727684 | 24.71596718 | 24.9144249 | 24.46849251 |
|  | 1.546290177 | -0.106576284 | Q8BHN3 | Neutral alpha-glucosidase AB | Ganab | 24.93975067 | 24.90407753 | 24.90380096 | 24.98459244 | 25.08042145 | 25.00234413 |
|  | 0.463367463 | 0.090907415 | P62869;A0A3B2WBM3 | Transcription elongation factor B polypeptide 2 | Tceb2 | 24.93732834 | 24.99636459 | 24.8649044 | 24.85095596 | 24.96851158 | 24.70640755 |
|  | 0.713577734 | -0.48551178 | Q64152 | Transcription factor BTF3 | Btf3 | 24.92985344 | 25.00324631 | 24.06365204 | 25.08176231 | 25.07062912 | 25.30089569 |
|  | 0.43184058 | 0.117717743 | Q9D8B3 | Charged multivesicular body protein 4b | Chmp4b | 24.91674995 | 25.02466393 | 24.98975563 | 25.02170181 | 24.64394379 | 24.91237068 |
|  | 1.145438095 | -0.148256938 | P10852;A0A0U1RP98;A0A0U1RPK4;A0A0U1RPL8;A0A0U1RPG5;A0A0U1RP32;A0A0U1RPQ7 | 4F2 cell-surface antigen heavy chain | Slc3a2 | 24.91579247 | 24.80252457 | 24.73065567 | 24.92134285 | 24.95455742 | 25.01784325 |
|  | 0.543746374 | 0.108508428 | Q8VDW0;D6RHT5 | ATP-dependent RNA helicase DDX39A | Ddx39a | 24.90614319 | 24.88804436 | 24.94547653 | 24.70735741 | 24.97731781 | 24.72946358 |
|  | 0.057041665 | -0.051649094 | A0A338P6G6;A0A384DV79;A0A338P6E8 | High mobility group AT-hook 1 | Hmga1 | 24.90159416 | 25.22802544 | 25.0123539 | 24.92329407 | 24.69375801 | 25.6798687 |
|  | 0.088571315 | -0.010587056 | Q99LR1;D6RFU2;D6RI21;F7BHM8 | Monoacylglycerol lipase ABHD12 | Abhd12 | 24.89522743 | 24.83465576 | 24.85486031 | 24.90076447 | 24.79580498 | 24.91993523 |
|  | 0.256282796 | 0.079781214 | F7D432;E9PWE0;E0CYV0;P23506;F6TXE3;F6V9F1 | Protein-L-isoaspartate O-methyltransferase;Protein-L-isoaspartate(D-aspartate) O-methyltransferase | Pcmt1 | 24.89397812 | 24.80538177 | 24.58520126 | 24.77067184 | 24.75867653 | 24.51586914 |
|  | 1.290885358 | 0.26188151 | Q3UPV6;P62482;E0CXZ9;A0A571BGH0;A0A571BF54;A0A571BEM7;E0CXI4;A0A5F8MPA4;A0A5F8MPK0;P63143;E0CYS2;A0A571BFX6;A0A571BE49 | Voltage-gated potassium channel subunit beta-2 | Kcnab2 | 24.88716125 | 24.98676491 | 24.7810173 | 24.64350319 | 24.74031448 | 24.48548126 |
|  | 0.252877308 | 0.088818868 | Q99KV1;A0A338P778 | DnaJ homolog subfamily B member 11 | Dnajb11 | 24.87900162 | 25.24158096 | 25.05366135 | 24.81509209 | 24.95929718 | 25.13339806 |
|  | 0.954165252 | -0.074512482 | Q3TWV4;P84091;A0A338P798;A0A338P6T4 | AP-2 complex subunit mu | Ap2m1 | 24.87750435 | 24.81592178 | 24.89031792 | 24.99200439 | 24.91497231 | 24.90030479 |
|  | 1.475266893 | 0.104721705 | P54071;D6RIL6;A0A0U1RP68;A0A0U1RPR1 | Isocitrate dehydrogenase [NADP], mitochondrial | Idh2 | 24.86485863 | 24.97022438 | 24.8907814 | 24.81083298 | 24.81455421 | 24.7863121 |
|  | 0.874203737 | 0.164510727 | P26516 | 26S proteasome non-ATPase regulatory subunit 7 | Psmd7 | 24.86386681 | 24.69651985 | 24.70872498 | 24.68212128 | 24.63732529 | 24.45613289 |
|  | 0.005626664 | 0.000999451 | Q8K2Q7;A0A0A6YWK4;A0A0A6YWP0;A0A0A6YVS9 | BRO1 domain-containing protein BROX | Brox | 24.86221123 | 24.77776146 | 24.79064369 | 24.8345108 | 24.7092514 | 24.88385582 |
|  | 0.817926805 | 0.130357107 | O35855;O88374;A0A1B0GX27;A0A1B0GST1;A0A1B0GQY4 | Branched-chain-amino-acid aminotransferase, mitochondrial;Branched-chain-amino-acid aminotransferase | Bcat2 | 24.83893967 | 24.99274063 | 24.74852753 | 24.72301865 | 24.70049477 | 24.76562309 |
|  | 1.928963058 | 0.122114182 | Q922B2;Q8BJY7 | Aspartate--tRNA ligase, cytoplasmic | Dars | 24.83662987 | 24.7767067 | 24.77062035 | 24.6925354 | 24.63627434 | 24.68880463 |
|  | 0.089508304 | 0.033347448 | P63024;B0QZN5;P63044 | Vesicle-associated membrane protein 3 | Vamp3 | 24.83045387 | 25.12541962 | 24.99459648 | 24.74755287 | 25.04260063 | 25.06027412 |
|  | 0.628748259 | -0.121936798 | Q80ZP8;Q3TMX5;Q9CXI5;F6USD5;F6T4L3;F7C1S6 | Mesencephalic astrocyte-derived neurotrophic factor | Manf | 24.83026123 | 24.88953018 | 24.90527153 | 25.02664948 | 25.12581253 | 24.83841133 |
|  | 2.116368425 | 0.131560008 | Q8BH95 | Enoyl-CoA hydratase, mitochondrial | Echs1 | 24.81733894 | 24.75755692 | 24.80877304 | 24.63167572 | 24.6607399 | 24.69657326 |
|  | 0.380378791 | -0.069339752 | P61028;REV__G3UYX5 | Ras-related protein Rab-8B | Rab8b | 24.81215668 | 24.72577667 | 24.65910721 | 24.92664528 | 24.739645 | 24.73876953 |
|  | 0.481267565 | -0.051314036 | B9EJ86;A0A0R4J150;A0A5F8MPC5;G5E833;Q9ER64;D3YWU9 | Oxysterol-binding protein | Osbpl8 | 24.81151962 | 24.72317505 | 24.80685806 | 24.86240005 | 24.87370682 | 24.75938797 |
|  | 1.518206433 | 0.154446284 | Q9R0P5 | Destrin | Dstn | 24.81063652 | 24.87633324 | 24.7983284 | 24.75261497 | 24.61946869 | 24.64987564 |
|  | 0.094424465 | 0.019647598 | Q62167;P16381 | ATP-dependent RNA helicase DDX3X;Putative ATP-dependent RNA helicase Pl10 | Ddx3x;D1Pas1 | 24.80114365 | 24.85571671 | 24.86603737 | 24.79248238 | 24.95712852 | 24.71434402 |
|  | 1.209545516 | 0.141899745 | A0A087WQE6;P83940;A0A087WNT1;A0A087WPE4 | Transcription elongation factor B polypeptide 1 | Tceb1 | 24.80069923 | 24.74770737 | 24.63223076 | 24.56732368 | 24.55555153 | 24.63206291 |
|  | 1.592114815 | 0.18936793 | Q9D051 | Pyruvate dehydrogenase E1 component subunit beta, mitochondrial | Pdhb | 24.79995918 | 24.88338852 | 24.85314751 | 24.60393715 | 24.61076736 | 24.7536869 |
|  | 0.022701794 | 0.006786982 | Q5XJY5 | Coatomer subunit delta | Arcn1 | 24.79624939 | 24.80945969 | 24.55923462 | 24.62678337 | 24.82420158 | 24.69359779 |
|  | 0.323098986 | 0.113635381 | Q9Z0L8 | Gamma-glutamyl hydrolase | Ggh | 24.79312706 | 24.96754456 | 25.01695061 | 24.5703373 | 25.00255966 | 24.86381912 |
|  | 0.128011322 | 0.01117897 | Q9R1P1 | Proteasome subunit beta type-3 | Psmb3 | 24.78646278 | 24.73402023 | 24.78765869 | 24.78974915 | 24.70508957 | 24.77976608 |
|  | 0.746826906 | -0.113742828 | Q3TE85;A0A338P6F6;P17095 | High mobility group protein HMG-I/HMG-Y | Hmga1 | 24.78596306 | 24.92980957 | 24.8426857 | 24.93242455 | 25.07606316 | 24.89119911 |
|  | 0.119734486 | 0.023431778 | O35226 | 26S proteasome non-ATPase regulatory subunit 4 | Psmd4 | 24.77479935 | 24.84747124 | 24.64553833 | 24.65458107 | 24.75557327 | 24.78735924 |
|  | 0.113674106 | -0.008572261 | Q9EQP2 | EH domain-containing protein 4 | Ehd4 | 24.77334023 | 24.83301544 | 24.76182365 | 24.77585411 | 24.82948685 | 24.78855515 |
|  | 1.350225137 | 0.228209178 | Q99N69;A0A494B990 | Leupaxin | Lpxn | 24.77238464 | 24.79624939 | 24.7112484 | 24.42508698 | 24.49411583 | 24.67605209 |
|  | 0.188776929 | 0.021718979 | A0A0J9YTY0;A0A0J9YUL3;Q8C1B7;A0A0J9YUV6;A0A0J9YVA6;A2A3W1 | Septin-11 | Septin11 | 24.76981354 | 24.72072411 | 24.73013687 | 24.75246239 | 24.76683617 | 24.63621902 |
|  | 1.958911421 | -0.188629786 | Q91YR9;REV__A0A0R4J177;REV__Q3TVC7 | Prostaglandin reductase 1 | Ptgr1 | 24.75801468 | 24.64526367 | 24.76562309 | 24.90145493 | 24.94310951 | 24.89022636 |
|  | 0.35393532 | -0.097897847 | A0A0N4SVQ1;Q62425 | Cytochrome c oxidase subunit NDUFA4 | Ndufa4 | 24.74909019 | 25.06142807 | 24.94677162 | 24.87872124 | 25.10581589 | 25.0664463 |
|  | 0.525209569 | -0.123581568 | Q5SW88;Q5SW87;Q5SW86 | RAB1A, member RAS oncogene family | Rab1 | 24.74760437 | 24.83021164 | 25.05146408 | 24.91456223 | 25.08711624 | 24.99834633 |
|  | 0.692060222 | 0.09969902 | Q8QZY1;A0A2R8VH97 | Eukaryotic translation initiation factor 3 subunit L | Eif3l | 24.74509239 | 24.71088028 | 24.5775013 | 24.65818214 | 24.5543232 | 24.52187157 |
|  | 0.502822906 | 0.076044083 | Q9DBC7;A2AI69;D3Z0V6;P12849;D3Z068 | cAMP-dependent protein kinase type I-alpha regulatory subunit;cAMP-dependent protein kinase type I-alpha regulatory subunit, N-terminally processed | Prkar1a | 24.74391365 | 24.56720734 | 24.6776123 | 24.61840439 | 24.50476074 | 24.63743591 |
|  | 0.5422685 | 0.070714951 | Q91XH5;G3UXX3;Q64105;G3UZ79 | Sepiapterin reductase | Spr | 24.74232101 | 24.69301414 | 24.74960136 | 24.72171593 | 24.54857254 | 24.7025032 |
|  | 0.851642918 | 0.241500854 | E9Q7G1;D3YZZ5 | Transmembrane p24 trafficking protein 7 | Tmed7 | 24.7359314 | 24.55162621 | 24.3554821 | 24.43389893 | 24.30263901 | 24.18199921 |
|  | 1.238851124 | 0.200792313 | Q99LP6 | GrpE protein homolog 1, mitochondrial | Grpel1 | 24.73443413 | 24.7645607 | 24.6654644 | 24.54339027 | 24.38929367 | 24.62939835 |
|  | 0.351224575 | 0.031121572 | P23116;A0A494B9I0 | Eukaryotic translation initiation factor 3 subunit A | Eif3a | 24.73350334 | 24.75999641 | 24.83171082 | 24.76101303 | 24.77097321 | 24.69985962 |
|  | 0.753630052 | 0.180924733 | P70315 | Wiskott-Aldrich syndrome protein homolog | Was | 24.72369576 | 24.76633072 | 24.49564171 | 24.35528183 | 24.60280609 | 24.48480606 |
|  | 1.198112912 | -0.126061757 | Q11136;G3UXC5 | Xaa-Pro dipeptidase | Pepd | 24.72338295 | 24.69842911 | 24.56813431 | 24.79624939 | 24.80469322 | 24.76718903 |
|  | 0.223987549 | -0.033269246 | Q8BH40;O70439 | Syntaxin-7 | Stx7 | 24.71685791 | 24.60172844 | 24.7824173 | 24.77122498 | 24.68891144 | 24.74067497 |
|  | 0.163965242 | 0.047193527 | B2RPU8;D3Z5B1;Q9D1L0 | Coiled-coil-helix-coiled-coil-helix domain-containing protein 2 | Zbed5;Chchd2 | 24.71596718 | 24.67049789 | 24.46799469 | 24.49460411 | 24.49338341 | 24.72489166 |
|  | 1.759052429 | 0.156810125 | Q9Z204;A0A2I3BRM6;A0A2I3BQH3;A0A2I3BQW7 | Heterogeneous nuclear ribonucleoproteins C1/C2 | Hnrnpc | 24.71591568 | 24.8289547 | 24.72936058 | 24.58749199 | 24.57859612 | 24.63771248 |
|  | 1.075116903 | -0.167161306 | P46978;D3YZN5 | Dolichyl-diphosphooligosaccharide--protein glycosyltransferase subunit STT3A | Stt3a | 24.71450043 | 24.91364861 | 24.73778915 | 24.89772224 | 25.02533913 | 24.94436073 |
|  | 0.557157784 | 0.06980896 | P27870;E9PXI0;Q8VDU4 | Proto-oncogene vav | Vav1 | 24.70961952 | 24.74693871 | 24.74658012 | 24.66383743 | 24.57114792 | 24.75872612 |
|  | 0.683121891 | 0.031585058 | Q60864 | Stress-induced-phosphoprotein 1 | Stip1 | 24.70830345 | 24.67044449 | 24.6866703 | 24.62109184 | 24.67271233 | 24.6768589 |
|  | 0.228590811 | -0.052695592 | G5E8R2;E9Q453;E9Q456;E9Q450;E9Q448;Q8BP43 | Tropomyosin 1, alpha | Tpm1 | 24.70593452 | 24.57478905 | 24.82191849 | 24.8484745 | 24.65703773 | 24.7552166 |
|  | 0.285208452 | 0.082272847 | A0A1W2P6G5 | Myosin, light polypeptide 6, alkali, smooth muscle and non-muscle | Myl6 | 24.70461464 | 24.75031662 | 24.77565193 | 24.58198929 | 24.5148468 | 24.88692856 |
|  | 0.980504857 | -0.068042119 | A0A0R4J259;Q7TMK9;G3UZI2;G3V018;G3UZ48;G3UXJ6;G3UWM1;G3XA76 | Heterogeneous nuclear ribonucleoprotein Q | Syncrip | 24.70429802 | 24.61313057 | 24.69811058 | 24.72977448 | 24.76734161 | 24.72254944 |
|  | 1.200474345 | 0.087403615 | Q9WUM5;A0A0N4SVU4 | Succinyl-CoA ligase [ADP/GDP-forming] subunit alpha, mitochondrial | Suclg1 | 24.70260811 | 24.64498901 | 24.63350487 | 24.5354023 | 24.55882454 | 24.62466431 |
|  | 1.388607447 | 0.244147619 | Q922D8;A0A1W2P733;A0A1W2P7L5 | C-1-tetrahydrofolate synthase, cytoplasmic;Methylenetetrahydrofolate dehydrogenase;Methenyltetrahydrofolate cyclohydrolase;Formyltetrahydrofolate synthetase;C-1-tetrahydrofolate synthase, cytoplasmic, N-terminally processed | Mthfd1 | 24.69842911 | 24.55303383 | 24.52276993 | 24.4432888 | 24.23257065 | 24.36593056 |
|  | 0.804135395 | -0.104841232 | A0A1B0GT81;Q07813;A0A1B0GS13;A0A1B0GTA4;A0A1B0GT35 | Apoptosis regulator BAX | Bax | 24.69784546 | 24.62900925 | 24.81318474 | 24.76713943 | 24.86064911 | 24.8267746 |
|  | 1.701474704 | 0.224012375 | P50247;A2ALT5 | Adenosylhomocysteinase | Ahcy | 24.69731522 | 24.83504105 | 24.65408897 | 24.53005219 | 24.52790833 | 24.4564476 |
|  | 0.902668042 | 0.277788798 | A0A286YDB7;A0A286YCT4;A0A286YCG8;Q9CY50 | Translocon-associated protein subunit alpha | Ssr1 | 24.69545746 | 24.51773262 | 25.00423241 | 24.43319893 | 24.45004082 | 24.50081635 |
|  | 2.433405876 | -0.167047501 | Q60865;F6YLI0 | Caprin-1 | Caprin1 | 24.69333267 | 24.68024445 | 24.74668312 | 24.88362312 | 24.89970589 | 24.83807373 |
|  | 0.313726754 | -0.056325912 | P49722;Q8BKE0 | Proteasome subunit alpha type-2 | Psma2 | 24.68933868 | 24.64740753 | 24.83730507 | 24.71109009 | 24.76557159 | 24.86636734 |
|  | 0.565846666 | 0.24175326 | A0A2K6EDJ7;E9Q5L2;E9PVD2;A6X935 | Inter alpha-trypsin inhibitor, heavy chain 4 | Itih4 | 24.68907166 | 24.5118351 | 24.63577652 | 24.62928772 | 24.01843643 | 24.46369934 |
|  | 0.063822494 | 0.007762273 | P63085;A0A338P781;A0A338P736;E9PXX5;E9Q3I6;Q6P5G0;Q61532 | Mitogen-activated protein kinase 1 | Mapk1 | 24.68816566 | 24.80370712 | 24.77595329 | 24.7010231 | 24.77966499 | 24.76385117 |
|  | 0.134166928 | 0.02325503 | A0A571BG95;E9PUE7;Q5SSL4;H3BKV6;H3BKT3;H3BJY3;H3BL84 | Active breakpoint cluster region-related protein | Abr | 24.68565369 | 24.86994934 | 24.83566666 | 24.74545288 | 24.83306313 | 24.74298859 |
|  | 0.106942557 | -0.016874949 | P17225;Q8CB58;E9QMW9;F7DCW4;E9Q279;F7AXP1;E9Q0W3 | Polypyrimidine tract-binding protein 1 | Ptbp1 | 24.68404961 | 24.67352104 | 24.78481483 | 24.81147003 | 24.72390366 | 24.65763664 |
|  | 0.820067868 | 0.086468379 | Q7TMB8;A0A0R4J119;A0A0U1RQ05;G3UZI5 | Cytoplasmic FMR1-interacting protein 1 | Cyfip1 | 24.68292427 | 24.81004906 | 24.80410194 | 24.72977448 | 24.66351128 | 24.64438438 |
|  | 1.235153344 | 0.211389542 | O88531;B1B0P8;B1B0P9 | Palmitoyl-protein thioesterase 1 | Ppt1 | 24.67863274 | 24.74154854 | 24.53557968 | 24.42220306 | 24.36031342 | 24.53907585 |
|  | 1.63297391 | 0.199341456 | Q3U4W8;P56399;D3YYA5;D3Z4K7 | Ubiquitin carboxyl-terminal hydrolase;Ubiquitin carboxyl-terminal hydrolase 5 | Usp5 | 24.67556763 | 24.62617111 | 24.73551941 | 24.50093842 | 24.39172173 | 24.54657364 |
|  | 0.138096719 | -0.010128021 | Q8BGQ7 | Alanine--tRNA ligase, cytoplasmic | Aars | 24.67492104 | 24.61790085 | 24.67831039 | 24.65447235 | 24.64300919 | 24.70403481 |
|  | 1.439394147 | 0.080029806 | O08528;E9Q5B5;E9Q9M6 | Hexokinase-2;Hexokinase | Hk2 | 24.67271233 | 24.68442345 | 24.68115616 | 24.64993095 | 24.58141327 | 24.56685829 |
|  | 0.106145713 | 0.056319555 | E0CXB2;E9QKZ2;Q91YE6;E0CY46;F6UT58 | Importin-9 | Ipo9 | 24.6718483 | 24.54668999 | 25.00375938 | 24.91232491 | 24.69349289 | 24.44752121 |
|  | 0.120766452 | 0.044682185 | P62835;A0A0G2JE52;A0A0G2JED9 | Ras-related protein Rap-1A | Rap1a | 24.66833496 | 24.86679268 | 24.94842148 | 24.98580933 | 24.62605858 | 24.73763466 |
|  | 1.450530426 | 0.047382991 | Q6P5E4;G3UYG7;G3UY73;G3UZU8;G3UY35;G3UXP5;E9Q4X2 | UDP-glucose:glycoprotein glucosyltransferase 1 | Uggt1 | 24.65725517 | 24.69423676 | 24.67265892 | 24.63123131 | 24.60710144 | 24.64366913 |
|  | 1.466408167 | 0.231811523 | P04117;A0A0A6YW05;A0A0A6YXB9;A0A0A6YXI2;P24526;O08716 | Fatty acid-binding protein, adipocyte | Fabp4 | 24.65261269 | 24.51062775 | 24.66595268 | 24.28848648 | 24.47487831 | 24.37039375 |
|  | 1.246568263 | 0.115113576 | Q9WUA3;Q8C605;D3YUA3;F6YL81 | ATP-dependent 6-phosphofructokinase, platelet type;ATP-dependent 6-phosphofructokinase | Pfkp | 24.65097046 | 24.56917953 | 24.6320076 | 24.43103218 | 24.53812981 | 24.53765488 |
|  | 0.207881265 | -0.052552541 | O35593 | 26S proteasome non-ATPase regulatory subunit 14 | Psmd14 | 24.64107895 | 24.71837425 | 24.67959976 | 24.9216156 | 24.61974907 | 24.65534592 |
|  | 1.908354623 | 0.198458354 | P35550;A0A140LIR6 | rRNA 2-O-methyltransferase fibrillarin | Fbl | 24.63754654 | 24.71617699 | 24.71193123 | 24.43052101 | 24.56092453 | 24.47883415 |
|  | 1.425830445 | 0.193626404 | P55302;F6WMD1 | Alpha-2-macroglobulin receptor-associated protein | Lrpap1 | 24.63383865 | 24.77530098 | 24.74401474 | 24.47747612 | 24.47790718 | 24.61689186 |
|  | 0.879780618 | -0.225837708 | P63321;A0A1Y7VL93 | Ras-related protein Ral-A | Rala | 24.63317299 | 24.89813805 | 24.49606895 | 24.89421082 | 24.93786621 | 24.87281609 |
|  | 0.542188373 | -0.11147054 | Q7TMM9 | Tubulin beta-2A chain | Tubb2a | 24.63006401 | 24.67017365 | 24.44044113 | 24.58388329 | 24.71450043 | 24.7767067 |
|  | 1.057845506 | 0.186133067 | Q6NSP9;P52927 | High mobility group protein HMGI-C | Hmga2 | 24.62789536 | 24.68453026 | 24.76162148 | 24.48492813 | 24.64063835 | 24.39008141 |
|  | 0.309092814 | 0.047557195 | Q99KQ4 | Nicotinamide phosphoribosyltransferase | Nampt | 24.61924553 | 24.65479851 | 24.48548126 | 24.6089077 | 24.49069405 | 24.51725197 |
|  | 0.962066401 | 0.103404999 | Q9D0I9 | Arginine--tRNA ligase, cytoplasmic | Rars | 24.61874199 | 24.7206192 | 24.68045998 | 24.57796097 | 24.63594246 | 24.49570274 |
|  | 0.084223368 | 0.059427897 | Q8JZU2;F6VVY4 | Tricarboxylate transport protein, mitochondrial | Slc25a1 | 24.61593819 | 23.82395935 | 24.07798004 | 24.08107185 | 24.28107452 | 23.97744751 |
|  | 0.189561043 | 0.047732035 | P62192 | 26S protease regulatory subunit 4 | Psmc1 | 24.61374855 | 24.77384377 | 24.78331757 | 24.80163765 | 24.5298748 | 24.69620132 |
|  | 1.034038618 | 0.125259399 | Q99K48 | Non-POU domain-containing octamer-binding protein | Nono | 24.60783577 | 24.70682907 | 24.79793167 | 24.60879517 | 24.56500053 | 24.56302261 |
|  | 0.360799027 | -0.104756673 | O88653;A0A0G2JGQ3 | Ragulator complex protein LAMTOR3 | Lamtor3 | 24.60778046 | 24.60676384 | 24.50427628 | 24.87886047 | 24.6775589 | 24.47667122 |
|  | 0.581132246 | 0.035244624 | Q8BMD8;A0A0G2JFB9 | Calcium-binding mitochondrial carrier protein SCaMC-1 | Slc25a24 | 24.6063118 | 24.64405441 | 24.57421303 | 24.58514404 | 24.5961647 | 24.53753662 |
|  | 0.386633464 | -0.104492188 | Q61990;B2M1R7;A0A2R8VI25;A0A2R8W6L5;A0A2R8VI12;A0A2R8VHG2;A0A2R8VI71;A0A2R8VKN0;A0A2R8VI73;A0A2R8W6U6;A0A2R8W6H3 | Poly(rC)-binding protein 2 | Pcbp2 | 24.60523796 | 24.69948769 | 24.62310219 | 24.73024178 | 24.5654068 | 24.94565582 |
|  | 0.27341824 | 0.038373311 | Q3TXS7;J3QN38;D6RGR5 | 26S proteasome non-ATPase regulatory subunit 1 | Psmd1 | 24.60059547 | 24.6025219 | 24.60048103 | 24.46413422 | 24.6590538 | 24.56529045 |
|  | 0.125522124 | -0.050879161 | A0A0A6YXE3;Q9JKP5;G3X9Q0;A0A0A6YVV8;A0A0A6YWB0;A0A0A6YXP3;A0A2K6EDM5;Q8C181;Q3U581;A0A0A6YWG1;Q3U570;A0A0A6YXQ4;A0A0A6YXL7;S4R267;A0A2I3BRX8;A0A0A6YWJ5;Q8R003 | Muscleblind-like protein 1;Muscleblind-like protein 2 | Mbnl1;Mbnl2 | 24.59770012 | 24.4820385 | 24.95318222 | 24.6568737 | 24.80842972 | 24.7202549 |
|  | 0.340051847 | 0.052715937 | P62315;A0A3Q4L381;A0A3Q4L2W0 | Small nuclear ribonucleoprotein Sm D1 | Snrpd1 | 24.59166527 | 24.52396584 | 24.65283203 | 24.56482506 | 24.43568039 | 24.60980988 |
|  | 1.299251153 | 0.210390727 | Q9CQ22;A0A0A6YX02 | Ragulator complex protein LAMTOR1 | Lamtor1 | 24.5912075 | 24.65872574 | 24.64526367 | 24.43096733 | 24.54274178 | 24.29031563 |
|  | 1.277901807 | 0.107089996 | Q8CGC7;A0A0A6YWA4;A0A0A6YWH3 | Bifunctional glutamate/proline--tRNA ligase;Glutamate--tRNA ligase;Proline--tRNA ligase | Eprs | 24.59069443 | 24.51610947 | 24.60755348 | 24.40963364 | 24.48726273 | 24.49619102 |
|  | 1.032520903 | -0.13392067 | Q8BJY1;F7BA91 | 26S proteasome non-ATPase regulatory subunit 5 | Psmd5 | 24.58932304 | 24.51917267 | 24.38574409 | 24.65605545 | 24.61907768 | 24.62086868 |
|  | 1.086385716 | 0.182909012 | P54728 | UV excision repair protein RAD23 homolog B | Rad23b | 24.58273506 | 24.54209137 | 24.76187515 | 24.36339188 | 24.48511314 | 24.48946953 |
|  | 0.739864434 | 0.173032761 | P06797;A0A1Y7VP49;A0A1Y7VNM3;A0A1Y7VNQ4 | Cathepsin L1;Cathepsin L1 heavy chain;Cathepsin L1 light chain | Ctsl | 24.58043671 | 24.3755722 | 24.35124207 | 24.41660881 | 24.21593475 | 24.15560913 |
|  | 1.697725914 | 0.073059082 | P70441 | Na(+)/H(+) exchange regulatory cofactor NHE-RF1 | Slc9a3r1 | 24.57865334 | 24.59775734 | 24.60121918 | 24.54810333 | 24.48566628 | 24.524683 |
|  | 0.823266753 | -0.108600616 | P97369;A8XU21 | Neutrophil cytosol factor 4 | Ncf4 | 24.57847977 | 24.61363602 | 24.59997177 | 24.58594704 | 24.77540207 | 24.7565403 |
|  | 0.805082311 | 0.082502365 | Q9D1L9;G3UW70 | Ragulator complex protein LAMTOR5 | Lamtor5 | 24.57773209 | 24.71077538 | 24.56894684 | 24.53136253 | 24.55929184 | 24.51929283 |
|  | 1.028167493 | 0.146479925 | Q8BK64;A0A1Y7VM19 | Activator of 90 kDa heat shock protein ATPase homolog 1 | Ahsa1 | 24.57525253 | 24.47109985 | 24.67022896 | 24.46051407 | 24.45926476 | 24.35736275 |
|  | 0.050147834 | 0.014434179 | Q9ER00 | Syntaxin-12 | Stx12 | 24.57450104 | 24.56284714 | 24.71759033 | 24.70155144 | 24.67637634 | 24.43370819 |
|  | 1.02978702 | 0.054249446 | A0A286YDF5;Q69ZN7;A0A286YCZ3;A0A286YE65;A0A286YDV5 | Myoferlin | Myof | 24.57380867 | 24.53296852 | 24.5116539 | 24.50494385 | 24.49874878 | 24.45199013 |
|  | 0.017325734 | -0.002721786 | P46471;Q8BVQ9 | 26S protease regulatory subunit 7 | Psmc2 | 24.56801987 | 24.5114727 | 24.55754089 | 24.57554054 | 24.45293427 | 24.61672401 |
|  | 0.032263595 | -0.005023956 | E9Q855;Q3UXS0;O35609 | Secretory carrier-associated membrane protein 3 | Scamp3 | 24.5656395 | 24.47617722 | 24.49753189 | 24.57051086 | 24.55549431 | 24.4284153 |
|  | 0.341110507 | 0.076398849 | O70194;A0A2R8VK20 | Eukaryotic translation initiation factor 3 subunit D | Eif3d | 24.56284714 | 24.36359215 | 24.44890785 | 24.29312706 | 24.52563858 | 24.32738495 |
|  | 0.439849975 | 0.058643341 | Q8BGD9;B2RWE8 | Eukaryotic translation initiation factor 4B | Eif4b | 24.55987549 | 24.60501099 | 24.53747749 | 24.46376038 | 24.61565781 | 24.44701576 |
|  | 0.942395415 | 0.318962733 | Q9D0T1 | NHP2-like protein 1;NHP2-like protein 1, N-terminally processed | Nhp2l1 | 24.55771637 | 24.29319572 | 24.83190346 | 24.22552681 | 24.29949951 | 24.20090103 |
|  | 0.548816861 | -0.075698217 | G5E924;Q8R081;G3UY38;G3UY56 | Heterogeneous nuclear ribonucleoprotein L | Hnrnpl | 24.55642891 | 24.72499657 | 24.70044136 | 24.67513657 | 24.75842285 | 24.77540207 |
|  | 0.853936851 | -0.16460228 | Q91WK2 | Eukaryotic translation initiation factor 3 subunit H | Eif3h | 24.55602074 | 24.68939209 | 24.77791214 | 24.94908905 | 24.73391724 | 24.83412552 |
|  | 0.503948007 | 0.072774251 | P51859;E0CXA0;E0CYW7 | Hepatoma-derived growth factor | Hdgf | 24.54663086 | 24.60636902 | 24.46544266 | 24.39675903 | 24.55905914 | 24.44430161 |
|  | 0.569465645 | 0.08655866 | Q9DCL9;D3Z6P1;D6RCU8 | Multifunctional protein ADE2;Phosphoribosylaminoimidazole-succinocarboxamide synthase;Phosphoribosylaminoimidazole carboxylase | Paics | 24.54492378 | 24.32299232 | 24.38106346 | 24.33019257 | 24.30820656 | 24.35090446 |
|  | 0.082807732 | 0.017120361 | P60229 | Eukaryotic translation initiation factor 3 subunit E | Eif3e | 24.53670692 | 24.64834023 | 24.66400146 | 24.65299606 | 24.47716713 | 24.66752434 |
|  | 0.43445811 | -0.083308538 | Q9QZD9;A2AE03 | Eukaryotic translation initiation factor 3 subunit I | Eif3i | 24.53469086 | 24.46195221 | 24.6300087 | 24.51412392 | 24.74304008 | 24.61941338 |
|  | 1.3610971 | 0.236487707 | Q9D8W5;B1AT36;Q3TRH2 | 26S proteasome non-ATPase regulatory subunit 12 | Psmd12 | 24.53052902 | 24.7204113 | 24.47562027 | 24.37225533 | 24.27298546 | 24.37185669 |
|  | 0.174499038 | -0.018223445 | Q8BG32;G3UYH2;G3UYI4;G3UYL3;G3UYL8;G3UX15;G3UWW7;G3UXL5;G3UX67;G3UWV7;G3UZ28;G3UZ33 | 26S proteasome non-ATPase regulatory subunit 11 | Psmd11 | 24.5201931 | 24.47072792 | 24.38784981 | 24.48271561 | 24.48983574 | 24.46088982 |
|  | 0.158257762 | 0.016937256 | E9QL31;A0A0R4J104;P98078;E9PX84;Q3TRE6;F6TQN9;Q9DCE6;E0CXT5;E0CZ53;E0CYJ2 | Disabled homolog 2 | Dab2 | 24.51292038 | 24.61245537 | 24.51159286 | 24.54916 | 24.48431396 | 24.55268288 |
|  | 0.109052429 | 0.022028605 | Q6IRU5;F7BHJ0 | Clathrin light chain B | Cltb | 24.50669861 | 24.48228455 | 24.705513 | 24.53344345 | 24.57813454 | 24.51683235 |
|  | 0.561914861 | 0.069460551 | Q80WJ7;F6QHD1;E9PUX0;F6ZQL0;F6ZSG0;F6QFT1 | Protein LYRIC | Mtdh | 24.50439835 | 24.36452866 | 24.43726921 | 24.41325569 | 24.39185333 | 24.29270554 |
|  | 1.075773048 | 0.194199244 | O08547;E9Q6R3;A0A0G2JF08;D6RES2 | Vesicle-trafficking protein SEC22b | Sec22b | 24.50099945 | 24.54580688 | 24.49283409 | 24.48129845 | 24.2700634 | 24.20568085 |
|  | 0.119147852 | -0.03626442 | P28063;G3UZW8;A0A494BAB6 | Proteasome subunit beta type-8;Proteasome subunit beta type | Psmb8 | 24.49990463 | 24.28397369 | 24.30138397 | 24.28220749 | 24.34435081 | 24.56749725 |
|  | 0.242736907 | 0.080915451 | E9QN99;A0A087WPF8;A0A087WSR2;A0A087WRJ2;Q8VCR7;A0A1L1SUX1;A0A087WP24;A0A1L1SRH5 | Alpha/beta hydrolase domain-containing protein 14B | Abhd14b | 24.49576378 | 24.4484024 | 24.3433342 | 24.14273643 | 24.57004738 | 24.33197021 |
|  | 1.087634187 | -0.112745921 | O08583;G3X9I4;Q9JJW6 | THO complex subunit 4;Aly/REF export factor 2 | Alyref;Alyref2 | 24.49203873 | 24.52874184 | 24.56581306 | 24.65888977 | 24.70740891 | 24.55853271 |
|  | 0.113396531 | -0.067616145 | J3QNU6;Q8BWG8;E0CY53;E0CYB1 | Beta-arrestin-1 | Arrb1 | 24.49203873 | 25.02453613 | 24.328619 | 24.61694717 | 24.78401756 | 24.64707756 |
|  | 0.457385691 | -0.017904282 | O89079;D3Z315;F6YFR7;E9Q6I5;F6XIG5 | Coatomer subunit epsilon | Cope | 24.49185562 | 24.4571991 | 24.4453125 | 24.48148346 | 24.46687508 | 24.49972153 |
|  | 0.467123839 | -0.077517192 | Q924B0;O55023;Q80ZJ2;A0A0A6YW07;D3Z703 | Inositol monophosphatase 1 | Impa1 | 24.48916245 | 24.45594597 | 24.62125969 | 24.70028305 | 24.53433418 | 24.56430244 |
|  | 0.528615963 | -0.08033371 | O08992;Q3TMX0;H3BLG5;A2AKJ9;A2AKJ6;A2AKJ5 | Syntenin-1 | Sdcbp | 24.48609543 | 24.55771637 | 24.60382462 | 24.72166252 | 24.52396584 | 24.64300919 |
|  | 0.091535744 | -0.031359355 | Q9EPB4;A0A0U1RQ20 | Apoptosis-associated speck-like protein containing a CARD | Pycard | 24.48339272 | 24.31665421 | 24.46369934 | 24.2359333 | 24.59565353 | 24.52623749 |
|  | 0.462071702 | -0.095851898 | Q62418 | Drebrin-like protein | Dbnl | 24.48308372 | 24.52396584 | 24.58250618 | 24.68736458 | 24.45795059 | 24.73179626 |
|  | 0.646180577 | -0.113080343 | A2A6U3;Q80UG5;A2A6U5;A8Y5D3 | Septin-9 | Septin9 | 24.48043633 | 24.7112484 | 24.52282906 | 24.72260094 | 24.61487198 | 24.71628189 |
|  | 0.075595662 | 0.01367569 | O88325 | Alpha-N-acetylglucosaminidase | Naglu | 24.48000526 | 24.36879539 | 24.56546402 | 24.42278099 | 24.43663406 | 24.51382256 |
|  | 1.403327803 | 0.212617874 | Q3TPJ8;O88487;A2BFF8;A2BFF9;A2BFF5 | Cytoplasmic dynein 1 intermediate chain 2 | Dync1i2 | 24.47463226 | 24.3923111 | 24.370327 | 24.1898613 | 24.09587288 | 24.31368256 |
|  | 0.506081689 | 0.020141602 | Q99KK7;A0A494BBC1;A0A494BA16;A0A494BBB3;A0A494B918 | Dipeptidyl peptidase 3 | Dpp3 | 24.47438431 | 24.44985199 | 24.41583633 | 24.42387009 | 24.43421745 | 24.42156029 |
|  | 1.076435132 | 0.208005269 | Q9WTP6;F7BP55 | Adenylate kinase 2, mitochondrial;Adenylate kinase 2, mitochondrial, N-terminally processed | Ak2 | 24.47370338 | 24.56511688 | 24.50282097 | 24.13241768 | 24.39014816 | 24.39505959 |
|  | 0.404505011 | 0.070750554 | Q8R5A3;S4R2K5;F2Z4B7;G5E867;F2Z3U3 | Amyloid beta A4 precursor protein-binding family B member 1-interacting protein | Apbb1ip | 24.47233963 | 24.37311935 | 24.55783272 | 24.33544922 | 24.49923515 | 24.35635567 |
|  | 1.750194894 | 0.124872208 | Q91V61;Q3U4F0;A0A494BB84 | Sideroflexin-3 | Sfxn3 | 24.470541 | 24.53552055 | 24.55021858 | 24.40360069 | 24.42412758 | 24.35393524 |
|  | 0.181455458 | 0.034243902 | Q9D8L3;Q62186 | Translocon-associated protein subunit delta | Ssr4 | 24.47010612 | 24.25745773 | 24.47252655 | 24.37398148 | 24.37537384 | 24.34800339 |
|  | 1.743884948 | 0.139117559 | E9PV41;F6XC54;D3Z074;E9PXV7;O08808 | Protein diaphanous homolog 1 | Diap1;Diaph1 | 24.45557022 | 24.48339272 | 24.49856567 | 24.28312683 | 24.3997612 | 24.3372879 |
|  | 0.411444772 | 0.059520721 | Q9Z1Z0;A0A0J9YUG0 | General vesicular transport factor p115 | Uso1 | 24.45249367 | 24.44056702 | 24.56383705 | 24.48332977 | 24.33224297 | 24.46276283 |
|  | 0.095097395 | 0.017161051 | Q7M6Y3;A0A1L1SUR7;A0A140LHG9 | Phosphatidylinositol-binding clathrin assembly protein | Picalm | 24.44859314 | 24.53380013 | 24.57588577 | 24.50500298 | 24.59183502 | 24.40995789 |
|  | 1.570124503 | -0.200422923 | P62627;A2AVR9 | Dynein light chain roadblock-type 1 | Dynlrb1 | 24.44606972 | 24.41473961 | 24.37802315 | 24.51995277 | 24.7113533 | 24.60879517 |
|  | 0.234308184 | 0.031239192 | P29758 | Ornithine aminotransferase, mitochondrial | Oat | 24.44600677 | 24.40158463 | 24.38060188 | 24.30005836 | 24.36679649 | 24.46762085 |
|  | 2.038076543 | 0.194682439 | P97384;D3Z7U0 | Annexin A11;Annexin | Anxa11 | 24.44493294 | 24.37630272 | 24.34197807 | 24.18268204 | 24.24590302 | 24.15058136 |
|  | 0.333752122 | 0.045523326 | O55029 | Coatomer subunit beta | Copb2 | 24.44297409 | 24.40613365 | 24.30173302 | 24.37278557 | 24.2639904 | 24.37749481 |
|  | 2.246179565 | -0.161518733 | Q9JMA1;E9PYI8 | Ubiquitin carboxyl-terminal hydrolase 14;Ubiquitin carboxyl-terminal hydrolase | Usp14 | 24.43777657 | 24.40099907 | 24.38238335 | 24.59434319 | 24.5929184 | 24.5184536 |
|  | 0.056299643 | 0.016377131 | Q9D2G2 | Dihydrolipoyllysine-residue succinyltransferase component of 2-oxoglutarate dehydrogenase complex, mitochondrial | Dlst | 24.43421745 | 24.32258034 | 24.09964943 | 24.25190926 | 24.31001091 | 24.24539566 |
|  | 0.995248345 | -0.176340103 | O54962 | Barrier-to-autointegration factor;Barrier-to-autointegration factor, N-terminally processed | Banf1 | 24.43351746 | 24.20254517 | 24.26169586 | 24.38725662 | 24.49814034 | 24.54138184 |
|  | 1.330459218 | -0.16136233 | P05063 | Fructose-bisphosphate aldolase C | Aldoc | 24.42937279 | 24.45882607 | 24.47797012 | 24.60580254 | 24.71696281 | 24.52749062 |
|  | 0.284846073 | -0.022853216 | Q921M7;A0A2I3BRN5;A0A2I3BPH9;A0A2I3BQK1 | Protein FAM49B | Fam49b | 24.42579269 | 24.42104721 | 24.51364326 | 24.47401237 | 24.49795723 | 24.45707321 |
|  | 0.176216897 | 0.040982564 | P46664 | Adenylosuccinate synthetase isozyme 2 | Adss | 24.42290878 | 24.53445244 | 24.62967682 | 24.52372742 | 24.36205482 | 24.57830811 |
|  | 1.064338527 | -0.092023849 | Q8CIE6;F8WHL2;F6XJN3 | Coatomer subunit alpha;Xenin;Proxenin;Coatomer subunit alpha | Copa | 24.42207527 | 24.38817787 | 24.41138077 | 24.43083954 | 24.49960136 | 24.56726456 |
|  | 0.090518026 | 0.027575811 | P51807;A0A338P7B7 | Dynein light chain Tctex-type 1 | Dynlt1 | 24.41615868 | 24.47364044 | 24.51195526 | 24.54250526 | 24.54651451 | 24.23000717 |
|  | 0.202336775 | -0.049943288 | P61804 | Dolichyl-diphosphooligosaccharide--protein glycosyltransferase subunit DAD1 | Dad1 | 24.41602898 | 24.72624397 | 24.56709099 | 24.58020592 | 24.68367577 | 24.59531212 |
|  | 0.162034 | -0.031832377 | A0A2I3BRL8;Q91VM5;A0A2I3BQC0;S4R1F6;Q9DAE2 | RNA binding motif protein, X-linked-like-1 | Rbmxl1 | 24.41196251 | 24.44398499 | 24.36952782 | 24.53397751 | 24.30194283 | 24.48505211 |
|  | 1.147855706 | -0.099831263 | P97855 | Ras GTPase-activating protein-binding protein 1 | G3bp1 | 24.39564896 | 24.43536186 | 24.46101379 | 24.48879433 | 24.50008774 | 24.60263634 |
|  | 0.227573844 | 0.028458913 | Q6P9Q6;A0A6I8MWZ0;Q80YW9;Q80YW6;Q80YW7 | FK506-binding protein 15;Peptidyl-prolyl cis-trans isomerase | Fkbp15 | 24.39388275 | 24.44890785 | 24.47895622 | 24.49387169 | 24.38896561 | 24.35353279 |
|  | 0.359807241 | 0.087352753 | P70460 | Vasodilator-stimulated phosphoprotein | Vasp | 24.39250755 | 24.36325836 | 24.48597336 | 24.18978691 | 24.28277206 | 24.50712204 |
|  | 0.096727696 | 0.014364243 | Q91YS8;D3Z368 | Calcium/calmodulin-dependent protein kinase type 1 | Camk1 | 24.38409805 | 24.36466217 | 24.338377 | 24.28510284 | 24.30890083 | 24.45004082 |
|  | 0.386920939 | 0.069070816 | Q8CI94 | Glycogen phosphorylase, brain form | Pygb | 24.38225174 | 24.3730526 | 24.17721558 | 24.24945068 | 24.29712105 | 24.17873573 |
|  | 0.036362632 | 0.006548564 | P62196;Q8K1K2;A0A0E2WI80 | 26S protease regulatory subunit 8 | Psmc5 | 24.38198853 | 24.37464523 | 24.24059868 | 24.39001656 | 24.33551788 | 24.25205231 |
|  | 0.055814199 | -0.008857091 | A0A2I3BRW0;Q9D958;A0A2I3BQG3 | Signal peptidase complex subunit 1 | Spcs1 | 24.38007355 | 24.39492989 | 24.54757309 | 24.42828751 | 24.46836662 | 24.45249367 |
|  | 0.464411883 | 0.130758286 | Q9DCH4 | Eukaryotic translation initiation factor 3 subunit F | Eif3f | 24.37464523 | 24.4049015 | 24.14086533 | 24.12691689 | 24.34813881 | 24.05308151 |
|  | 0.592899097 | 0.015312195 | Q8BPU7;A0A1Y7VIX9 | Engulfment and cell motility protein 1 | Elmo1 | 24.37272072 | 24.37762642 | 24.40295029 | 24.35917473 | 24.36619759 | 24.38198853 |
|  | 0.723454123 | 0.16318957 | A0A087WRY3;Q80XU3 | Nuclear ubiquitous casein and cyclin-dependent kinase substrate 1 | Nucks1 | 24.37245369 | 24.58548927 | 24.56813431 | 24.39773941 | 24.2932663 | NaN |
|  | 0.395633699 | 0.119440079 | O54984;A0A1B0GRE1 | ATPase Asna1 | Asna1 | 24.36472893 | 24.17934418 | 24.5002079 | 24.07806015 | 24.38053513 | 24.22736549 |
|  | 0.456660658 | 0.168429057 | Q8R016;E9PZH4 | Bleomycin hydrolase | Blmh | 24.36332512 | 24.1139431 | 24.35743141 | 23.96322823 | NaN | 24.25638008 |
|  | 0.140500319 | 0.028025945 | P31938 | Dual specificity mitogen-activated protein kinase kinase 1 | Map2k1 | 24.35157967 | 24.30938721 | 24.44221497 | 24.32491684 | 24.23943329 | 24.45475388 |
|  | 0.575121625 | -0.101319631 | P08030;A0A1D5RLR6 | Adenine phosphoribosyltransferase | Aprt | 24.35144424 | 24.3767662 | 24.51213646 | 24.56633568 | 24.39414406 | 24.58382607 |
|  | 0.564869386 | 0.128740311 | Q9JKC8;H7BWY2;A0A286YDZ6;D3YXV9;D3YWU3;Q8R2R9;D6RI63 | AP-3 complex subunit mu-1 | Ap3m1 | 24.35056877 | 24.403862 | 24.44550133 | 24.09426117 | 24.43033028 | 24.28911972 |
|  | 0.39580149 | 0.08141009 | Q04899 | Cyclin-dependent kinase 18 | Cdk18 | 24.34563637 | 24.33960152 | 24.37477684 | 24.11743546 | 24.28277206 | 24.41557693 |
|  | 1.016193382 | 0.251084646 | Q9WU81 | Sugar phosphate exchanger 2 | Slc37a2 | 24.34516335 | 24.13116264 | 24.17371368 | 23.79143906 | 24.12170982 | 23.98363686 |
|  | 0.592333253 | -0.087587357 | A0A1C7CYV0;Q99K51;B1AX58 | Plastin-3 | Pls3 | 24.34387589 | 24.47500229 | 24.48136139 | 24.43224335 | 24.59957504 | 24.53118324 |
|  | 0.615753805 | 0.083562215 | O88685;A2AGN7;B7ZCF1 | 26S protease regulatory subunit 6A | Psmc3 | 24.3415699 | 24.34048462 | 24.4607029 | 24.21341515 | 24.3064003 | 24.37225533 |
|  | 0.574362355 | -0.058434804 | Q9DB05;A0A1B0GR35;P28663 | Alpha-soluble NSF attachment protein | Napa | 24.34082413 | 24.4434166 | 24.32765961 | 24.48086739 | 24.39204979 | 24.41428757 |
|  | 0.858173242 | 0.106463114 | Q8QZT1 | Acetyl-CoA acetyltransferase, mitochondrial | Acat1 | 24.34075546 | 24.32800293 | 24.27604294 | 24.18540573 | 24.12833214 | 24.31167412 |
|  | 1.811766287 | 0.201489766 | Q8K1X4;A0A2R8VHC4 | Nck-associated protein 1-like | Nckap1l | 24.33810616 | 24.46998215 | 24.43510818 | 24.2517643 | 24.153368 | 24.23359489 |
|  | 0.726493017 | 0.089246114 | Q3T9X3;P39054;F8WIV5;G3UZZ3 | Dynamin-2 | Dnm2 | 24.33681297 | 24.27120399 | 24.29621124 | 24.10868835 | 24.28291512 | 24.2448864 |
|  | 0.927378812 | 0.263479869 | F7CDT0;F6WMC0;P61082;G5E919 | NEDD8-conjugating enzyme Ubc12 | Ube2m | 24.33497238 | 24.56139183 | 24.63986588 | 24.08009529 | 24.25205231 | 24.41364288 |
|  | 0.252219932 | -0.05789121 | P63163;P27048;A0A0G2JGN4 | Small nuclear ribonucleoprotein-associated protein N;Small nuclear ribonucleoprotein-associated protein B | Snrpn;Snrpb | 24.33449554 | 24.38370323 | 24.30500793 | 24.23147202 | 24.43523598 | 24.53017235 |
|  | 0.089466909 | -0.04073143 | B7ZC46;B1AQY9;B1AQZ0;Q8CHH9;E0CYM4 | Septin-8 | Septin8 | 24.33197021 | 24.14211273 | 24.0350666 | 24.23469162 | 24.43421745 | 23.96243477 |
|  | 0.892936758 | -0.185934703 | Q4VAA2;A0A087WNP6;A0A087WRM0;A0A087WS49;F8WGL9 | Protein CDV3 | Cdv3 | 24.33101273 | 24.62750626 | 24.58880806 | 24.65003967 | 24.74463081 | 24.71046066 |
|  | 0.976624075 | -0.110764821 | Q9WVJ2;E9Q5I9;F6ZQQ3;E9Q0U1;F6PXS6 | 26S proteasome non-ATPase regulatory subunit 13 | Psmd13 | 24.31782722 | 24.38225174 | 24.38515282 | 24.37716293 | 24.5345726 | 24.50579071 |
|  | 1.220172476 | 0.097721736 | P45952;D3Z2A5 | Medium-chain specific acyl-CoA dehydrogenase, mitochondrial | Acadm | 24.31603241 | 24.33306313 | 24.23812294 | 24.15205383 | 24.21096611 | 24.23103333 |
|  | 0.692475595 | 0.335318883 | Q9WTL7 | Acyl-protein thioesterase 2 | Lypla2 | 24.31402779 | 24.44480705 | 24.62366104 | 24.42956352 | 23.74457932 | 24.20239639 |
|  | 0.800095669 | 0.120075226 | O55135;A6PWZ2;D6RG53;D6RJJ3;B1AZQ4 | Eukaryotic translation initiation factor 6 | Eif6 | 24.31084251 | 24.23578835 | 24.23746681 | 24.08350945 | 24.06972694 | 24.2706356 |
|  | 0.852791975 | 0.166015625 | O55234;Q8BTY5 | Proteasome subunit beta type-5 | Psmb5 | 24.30959511 | 24.16560936 | 24.08958244 | 23.96057892 | 24.14903259 | 23.95712852 |
|  | 0.381805339 | 0.061477025 | Q9Z2I9 | Succinyl-CoA ligase [ADP-forming] subunit beta, mitochondrial | Sucla2 | 24.30215073 | 24.33742523 | 24.34753036 | 24.15753746 | 24.38660049 | 24.25853729 |
|  | 1.030605875 | 0.166768392 | Q91YR1;D3Z2H0 | Twinfilin-1 | Twf1 | 24.29984856 | 24.44915962 | 24.22162437 | 24.15135574 | 24.09675789 | 24.22221375 |
|  | 0.100025821 | -0.053561529 | P61979;H3BLL4;A0A286YDM3;H3BK96;A0A286YCM2;A0A286YEC4;A0A286YDH1 | Heterogeneous nuclear ribonucleoprotein K | Hnrnpk | 24.29740143 | 24.38073349 | 24.8080368 | 24.470541 | 24.41170502 | 24.76461029 |
|  | 0.91059033 | 0.119410833 | Q9JHS3;D3YTS4 | Ragulator complex protein LAMTOR2 | Lamtor2 | 24.29263496 | 24.38751984 | 24.20239639 | 24.12273598 | 24.17546654 | 24.22611618 |
|  | 1.085200297 | -0.231494904 | O70252;D3YX62;D3YXN4;D3Z4A2 | Heme oxygenase 2 | Hmox2 | 24.29235458 | 23.97962952 | 24.07111931 | 24.27206039 | 24.36739731 | 24.39813042 |
|  | 1.01549674 | -0.12663269 | K3W4T3;Q9Z1G4;A2A599 | V-type proton ATPase subunit a;V-type proton ATPase 116 kDa subunit a isoform 1 | Atp6v0a1 | 24.28785324 | 24.42981911 | 24.38581085 | 24.47580719 | 24.57259369 | 24.43498039 |
|  | 0.076545579 | -0.014698664 | Q921F2;Q6VYI4;Q6VYI5;Q8BLD4;Q8R0B4;A0A087WRZ5;A0A087WR97;A0A087WQA5;A0A087WSH7;A0A087WSE4;H3BJV1;A0A087WRP4;A0A087WS74;A0A087WNY6;A0A087WQX8;A0A087WS17;A0A087WP57;A0A087WSC6 | TAR DNA-binding protein 43 | Tardbp | 24.28644371 | 24.21933556 | 24.44113731 | 24.31796646 | 24.31125832 | 24.3617878 |
|  | 1.838125209 | 0.178455989 | E9Q9E8;A0A0R4J0K5;Q18PI6;A0A0R4J1S4 | SLAM family member 5 | Cd84 | 24.28199577 | 24.19182014 | 24.14421654 | 23.99938011 | 24.05233574 | 24.03094864 |
|  | 0.764205718 | 0.092439651 | Q9WUA2;A0A087WS80;A0A087WPV4;A0A087WQ15 | Phenylalanine--tRNA ligase beta subunit | Farsb | 24.27710724 | 24.23731995 | 24.27134705 | 24.21200562 | 24.23469162 | 24.06175804 |
|  | 0.244098093 | -0.025310516 | Q9QUR6 | Prolyl endopeptidase | Prep | 24.27476311 | 24.16177177 | 24.22993279 | 24.21704483 | 24.29586029 | 24.22949409 |
|  | 1.348170812 | -0.253505707 | P63073;A0A0G2JGT5;A0A0G2JH04;A0A0G2JFB4;A0A0G2JG98 | Eukaryotic translation initiation factor 4E | Eif4e | 24.27134705 | 23.97945595 | 24.07316399 | 24.38692856 | 24.37099266 | 24.32656288 |
|  | 0.017665004 | -0.005486806 | Q9R062;K3W4S6;V9GX26 | Glycogenin-1 | Gyg1;Gyg | 24.26656532 | 24.25839424 | 24.32806969 | 24.18245506 | 24.19573021 | 24.4913044 |
|  | 0.561072608 | 0.149510066 | P28352;D3Z124;D3Z6R9 | DNA-(apurinic or apyrimidinic site) lyase;DNA-(apurinic or apyrimidinic site) lyase, mitochondrial | Apex1 | 24.26341629 | 24.01188469 | 23.98450661 | 23.78086662 | 24.01256752 | 24.01784325 |
|  | 1.368686467 | 0.149819056 | Q8R050;Q149F3;F7CE88 | Eukaryotic peptide chain release factor GTP-binding subunit ERF3A | Gspt1 | 24.25738716 | 24.33135414 | 24.3259449 | 24.24554062 | 24.10828972 | 24.1113987 |
|  | 0.932413665 | -0.13610967 | Q91ZJ5 | UTP--glucose-1-phosphate uridylyltransferase | Ugp2 | 24.25414658 | 24.38462448 | 24.30813599 | 24.37298584 | 24.42027664 | 24.56197357 |
|  | 0.405842964 | -0.191225688 | Q9D5V6 | Synapse-associated protein 1 | Syap1 | 24.2524147 | 23.62354851 | 24.00247383 | 24.31208992 | 24.06726837 | 24.07275581 |
|  | 0.812858386 | -0.207420985 | Q9CQX2 | Cytochrome b5 type B | Cyb5b | 24.24554062 | 24.03212547 | 24.38850594 | 24.34840775 | 24.53889847 | 24.40112877 |
|  | 0.927941423 | 0.163064957 | Q99L04 | Dehydrogenase/reductase SDR family member 1 | Dhrs1 | 24.24154472 | 24.12872696 | 24.22346687 | 24.17249298 | 23.91752434 | 24.01452637 |
|  | 1.182338782 | 0.196775119 | Q9D358;A0A1W2P7X3;Q561M1 | Low molecular weight phosphotyrosine protein phosphatase | Acp1 | 24.24045372 | 24.3321743 | 24.38903236 | NaN | 24.06874466 | 24.17881203 |
|  | 1.208085637 | 0.157905579 | Q9CQX8;Q9D6T9 | 28S ribosomal protein S36, mitochondrial | Mrps36 | 24.23257065 | 24.22971344 | 24.06447411 | 23.96780777 | 24.0287571 | 24.05647659 |
|  | 0.624940719 | 0.110692978 | P30681;A0A1B0GQX9 | High mobility group protein B2 | Hmgb2 | 24.22714424 | 24.09715843 | 23.96137428 | 23.94368935 | 24.01682281 | 23.99308586 |
|  | 1.682913713 | 0.077681859 | P24527 | Leukotriene A-4 hydrolase | Lta4h | 24.22405624 | 24.24648285 | 24.21859741 | 24.13414383 | 24.13163376 | 24.19031334 |
|  | 0.49327483 | -0.101031621 | Q9D031;Q01730;A2AUR7;B1AYQ0;E0CXG5;A0A0A6YWZ2 | Ras suppressor protein 1 | Rsu1 | 24.21985245 | 24.1378994 | 24.17767334 | 24.22457123 | 24.44833946 | 24.16560936 |
|  | 0.716157167 | -0.176692963 | P70372 | ELAV-like protein 1 | Elavl1 | 24.21852303 | 24.34705734 | 23.96419907 | 24.3692627 | 24.34631348 | 24.34428215 |
|  | 0.547655038 | -0.225695292 | Q8R307 | Vacuolar protein sorting-associated protein 18 homolog | Vps18 | 24.21512032 | 24.52324867 | 24.41783333 | 24.83903694 | 24.30688667 | 24.68736458 |
|  | 0.405266179 | -0.124140422 | Q8K1I7;F6QWW7;F6RQI2 | WAS/WASL-interacting protein family member 1 | Wipf1 | 24.21475029 | 24.44928551 | 24.56604576 | 24.66416359 | 24.54615974 | 24.39217949 |
|  | 1.564870929 | 0.18201383 | Q9DBH5 | Vesicular integral-membrane protein VIP36 | Lman2 | 24.21319199 | 24.37065887 | 24.23739243 | 24.07903862 | 24.13382912 | 24.06233406 |
|  | 0.248414067 | -0.067902883 | Q6ZWQ7 | Signal peptidase complex subunit 3 | Spcs3 | 24.21208 | 24.16038704 | 24.48400688 | 24.37967682 | 24.27398109 | 24.40652466 |
|  | 0.179751631 | -0.069330851 | Q9CQ65 | S-methyl-5-thioadenosine phosphorylase | Mtap | 24.21178246 | 23.99524498 | 24.07447243 | 24.03942299 | 24.42764664 | 24.02242279 |
|  | 0.385857153 | 0.082606634 | P56542;D3Z512;D3Z5U3 | Deoxyribonuclease-2-alpha | Dnase2;Dnase2a | 24.21081734 | 24.36599731 | 24.10133362 | 24.06825256 | 24.23052025 | 24.13155556 |
|  | 0.808618919 | 0.254301707 | Q08879 | Fibulin-1 | Fbln1 | 24.20732117 | 23.83595657 | 23.72806168 | 23.68474579 | 23.67238808 | 23.65130043 |
|  | 1.088965347 | 0.129740397 | P46460;G3UX86;G3UX98 | Vesicle-fusing ATPase | Nsf | 24.2047863 | 24.23797607 | 24.19887924 | 24.17850876 | 23.98910522 | 24.08480644 |
|  | 0.667964823 | -0.058123271 | Q9CZE3;A0A1Y7VIZ0;A0A140LHK2;E9QLQ7;Q91YQ1;Q8QZZ8 | Ras-related protein Rab-32 | Rab32 | 24.20291901 | 24.23659134 | 24.1649971 | 24.3091774 | 24.19550514 | 24.27419472 |
|  | 1.260166286 | 0.242812475 | P28650;J3QN31 | Adenylosuccinate synthetase isozyme 1 | Adssl1 | 24.19992828 | 24.16851997 | 24.33796883 | 23.84747124 | 24.04084587 | 24.08966255 |
|  | 0.145072254 | 0.030298869 | P62334 | 26S protease regulatory subunit 10B | Psmc6 | 24.19947815 | 24.27965927 | 24.44569016 | 24.31375122 | 24.22368813 | 24.29649162 |
|  | 0.888221923 | 0.260368983 | Q9Z1N5;G3UXI6 | Spliceosome RNA helicase Ddx39b | Ddx39b | 24.19422722 | 24.22839355 | 24.42943573 | 24.23731995 | 23.84201431 | 23.9916153 |
|  | 0.050249476 | -0.010890961 | Q922F4 | Tubulin beta-6 chain | Tubb6 | 24.19151878 | 24.3467865 | 24.19332504 | 24.2669239 | 24.15614891 | 24.34123039 |
|  | 0.075790539 | 0.018096288 | Q3UZ39;E9Q9T1;A0A087WPK3;A0A087WSF5;G5E8E1;A0A087WNU6;A0A087WPT0 | Leucine-rich repeat flightless-interacting protein 1 | Lrrfip1 | 24.1882782 | 24.17752075 | 24.41738129 | 24.18601227 | 24.25270271 | 24.29017639 |
|  | 0.946512092 | -0.045776367 | O54782;F6TMZ3;F6Z025 | Epididymis-specific alpha-mannosidase | Man2b2 | 24.18805122 | 24.20694733 | 24.16744804 | 24.19475365 | 24.24670029 | 24.25832176 |
|  | 1.006935131 | 0.12313652 | D3Z158;Q8BML9;A0A140LHZ5;A0A0A6YY08;A0A140LIS6;A0A140LIN2;A0A140LHJ3;A0A140LHB3;A0A140LJH2;A0A140LID3;A0A140LJK5;A0A140LJH1;A0A140LIZ4;A0A140LIR0;F6TDS3;A0A140LII8 | glutamine--tRNA ligase | Qars | 24.18585968 | 24.07520676 | 24.02825165 | 23.99920654 | 24.01350403 | 23.90719795 |
|  | 1.360376524 | -0.175400416 | Q8CIN4 | Serine/threonine-protein kinase PAK 2;PAK-2p27;PAK-2p34 | Pak2 | 24.18442345 | 24.2736969 | 24.14918709 | 24.44978905 | 24.28813553 | 24.39558411 |
|  | 0.043125702 | -0.00860405 | Q64737;D6RCG1;A0A338P6X4;A0A338P676;A0A338P6W0 | Trifunctional purine biosynthetic protein adenosine-3;Phosphoribosylamine--glycine ligase;Phosphoribosylformylglycinamidine cyclo-ligase;Phosphoribosylglycinamide formyltransferase | Gart | 24.18237877 | 24.04193115 | 24.21630478 | 24.09055138 | 24.14125633 | 24.23461914 |
|  | 0.188699739 | 0.061016719 | Q9DC51;A2AE32;P20612;Q3V3I2;P50149;P18872;A2AE31;Q8C040;D3Z2M7;Q8BHK8;F6QPU5 | Guanine nucleotide-binding protein G(k) subunit alpha | Gnai3 | 24.17904091 | 23.87530327 | 23.98207092 | 24.12257767 | 23.85595322 | 23.87483406 |
|  | 0.928083713 | 0.270151138 | Q5SSZ5;A0A5F8MP98;A0A5F8MPF2;A0A2R8VHQ0;Q8CGB6 | Tensin-3 | Tns3 | 24.17843246 | 23.98528862 | 23.9771862 | 23.89105988 | 23.53872108 | 23.90067291 |
|  | 1.300199273 | 0.237584432 | P61211;F8WIB1;E9Q006 | ADP-ribosylation factor-like protein 1 | Arl1 | 24.17835617 | 24.21289635 | 24.21652603 | 23.80823326 | 24.09924698 | 23.98754501 |
|  | 1.015087977 | -0.184842428 | Q8VC28;D6RGB1;G3X9Y6 | Aldo-keto reductase family 1 member C13 | Akr1c13 | 24.17417145 | 24.1180687 | 24.28312683 | 24.29249382 | 24.32092857 | 24.51647186 |
|  | 1.250275563 | 0.181362152 | Q91YH5;E9PYT3;A0A494BAX8;A0A494BAU1;A0A494BAW9;A0A494B9P1;Q8BH66 | Atlastin-3 | Atl3 | 24.16254044 | 24.11044312 | 24.22265625 | 24.04184914 | 23.86410141 | 24.0456028 |
|  | 0.890855302 | 0.347068151 | Q80XR5;P26369;Q3KQM4;A0A140LJK3;A0A140LJ08 | Splicing factor U2AF 65 kDa subunit | U2af2 | 24.16077232 | 23.7165966 | 23.61492729 | 23.59069443 | 23.3521862 | 23.50821114 |
|  | 0.694928601 | 0.101669947 | P24547;A0A0A6YY72;A0A0A6YXS4 | Inosine-5-monophosphate dehydrogenase 2 | Impdh2 | 24.15468216 | 24.30263901 | 24.28890991 | 24.18842888 | 24.19962883 | 24.05316353 |
|  | 0.101954233 | -0.024130503 | Q9ERN0;A0A1L1SUU4 | Secretory carrier-associated membrane protein 2 | Scamp2 | 24.14631462 | 24.04092979 | 24.10189438 | 24.16691208 | 24.22883415 | 23.96578407 |
|  | 0.716624604 | -0.116897583 | Q9R112;H3BLH2;F6ZKZ3 | Sulfide:quinone oxidoreductase, mitochondrial | Sqrdl | 24.14584923 | 24.00418854 | 24.23637199 | 24.27660942 | 24.27788734 | 24.18260574 |
|  | 0.672859656 | 0.159439087 | P32921 | Tryptophan--tRNA ligase, cytoplasmic;T1-TrpRS;T2-TrpRS | Wars | 24.14343834 | 23.85699844 | 24.19460297 | 23.94859886 | 23.8986454 | 23.86947823 |
|  | 0.414533321 | 0.04194959 | Q921T2;J3QQ40;Q3UE61;D3YXF8 | Torsin-1A-interacting protein 1 | Tor1aip1 | 24.14078712 | 24.28700829 | 24.21874619 | 24.19016266 | 24.16538048 | 24.16514969 |
|  | 0.366623741 | -0.048877716 | Q61768;E9QAK5;P28738;P33175;A2ARD4 | Kinesin-1 heavy chain;Kinesin-like protein | Kif5b | 24.13492584 | 24.11012459 | 24.13367271 | 24.28058052 | 24.0945034 | 24.15027237 |
|  | 1.406538355 | -0.097448349 | Q5SUA5;Q5SYD0 | Unconventional myosin-Ig | Myo1g | 24.13179016 | 24.08172226 | 24.10797119 | 24.148489 | 24.24437904 | 24.22096062 |
|  | 0.032040911 | -0.011305491 | Q9CS42 | Ribose-phosphate pyrophosphokinase 2 | Prps2 | 24.12864685 | 24.13367271 | 23.89198685 | 24.18465042 | 24.11251259 | 23.89105988 |
|  | 0.182196169 | -0.017719905 | P54775;A0A140LIZ5 | 26S protease regulatory subunit 6B | Psmc4 | 24.12439346 | 24.15475845 | 24.10781097 | 24.09756088 | 24.12959099 | 24.21297073 |
|  | 0.716256661 | -0.196813583 | P62307 | Small nuclear ribonucleoprotein F | Snrpf | 24.12423706 | 24.34177399 | 24.4396801 | 24.37132454 | 24.467062 | 24.65774536 |
|  | 0.016968408 | 0.002878189 | Q9QZ08;Q9D997;D3YXG2 | N-acetyl-D-glucosamine kinase | Nagk | 24.11981201 | 24.16522598 | 24.1914444 | 24.20657539 | 24.05142212 | 24.20985031 |
|  | 0.692638666 | 0.23899587 | Q3TAI4;A2AUD5;Q9CYZ2;V9GWU5;Q3TUJ9;Q8BKP1;F6VQ81 | Tumor protein D54 | Tpd52l2 | 24.11933708 | 23.74447823 | 23.87726974 | 23.86419678 | 23.68495941 | 23.47494125 |
|  | 0.279017842 | -0.075755437 | Q5SVG5;Q5SVG4;O35643 | AP-1 complex subunit beta-1 | Ap1b1 | 24.11711884 | 24.00478935 | 24.13633537 | 24.21881866 | 23.965168 | 24.30152321 |
|  | 0.489168106 | 0.104069392 | P70333 | Heterogeneous nuclear ribonucleoprotein H2 | Hnrnph2 | 24.11648369 | 24.18290901 | 24.34258842 | 24.11227417 | 24.21940994 | 23.99808884 |
|  | 0.843127542 | -0.047719955 | A0A087WP83;Q8VDJ3;A0A087WS92;A0A087WQY9;A0A087WPC5 | Vigilin | Hdlbp | 24.11616707 | 24.10157394 | 24.12399864 | 24.18631363 | 24.18782425 | 24.11076164 |
|  | 1.74405224 | 0.171892166 | Q68FL4;F8WGT1;F8WI65;H3BKT5;H3BL31;D3YYM7;A0A0N4SUY4;E9PX77;D3YX97;F7ATQ6;D3Z2Q0 | Putative adenosylhomocysteinase 3;Adenosylhomocysteinase | Ahcyl2 | 24.11433983 | 24.10789108 | 24.18343925 | 23.888834 | 24.00358772 | 23.99757195 |
|  | 0.112510353 | -0.033704122 | Q3UND0;A0A0N4SVW8 | Src kinase-associated phosphoprotein 2 | Skap2 | 24.11410141 | 23.95659828 | 23.8080368 | 23.91323853 | 23.94877815 | 24.11783218 |
|  | 1.099247031 | 0.204139074 | Q61206;A0A1L1SVK0;A0A1L1SRD0;A0A1L1SQ76;A0A1L1SQV7;A0A1L1SSQ5 | Platelet-activating factor acetylhydrolase IB subunit beta | Pafah1b2 | 24.10813141 | 24.04335022 | 24.31838036 | 23.9568634 | 23.90352631 | 23.99705505 |
|  | 1.101551533 | 0.173879623 | A0A5F8MPS7;P24638;B7ZCF5;B7ZCF4 | Lysosomal acid phosphatase | Acp2 | 24.10717392 | 23.99731255 | 23.89929008 | 23.78606224 | 23.914608 | 23.78146744 |
|  | 1.534649574 | -0.163155238 | Q9CZ44;A2AT02 | NSFL1 cofactor p47 | Nsfl1c | 24.1070137 | 23.98172188 | 24.0322094 | 24.13836861 | 24.22802544 | 24.24401665 |
|  | 0.03561535 | 0.01019605 | P61021 | Ras-related protein Rab-5B | Rab5b | 24.10653496 | 24.07079315 | 24.28722 | 24.09014702 | 24.28362083 | 24.06019211 |
|  | 0.572370743 | 0.136494954 | Q91YI0;E0CY49;F7D439;E0CYV3 | Argininosuccinate lyase | Asl | 24.10653496 | 23.99222183 | 24.09555054 | 23.95828056 | 23.7426281 | 24.0839138 |
|  | 1.276945684 | 0.110502879 | Q8R1B4;M0QWV3 | Eukaryotic translation initiation factor 3 subunit C | Eif3c | 24.10605431 | 24.14910889 | 24.06529617 | 23.94413757 | 23.98858643 | 24.05622673 |
|  | 0.182963395 | 0.038105647 | B7ZNL2;Q78ZA7;A0A140LJB5;A0A140LJ37;A0A140LI78 | Nucleosome assembly protein 1-like 4 | Nap1l4 | 24.10317612 | 24.00538826 | 23.97000504 | 24.04635239 | 23.85128975 | 24.06661034 |
|  | 1.616587513 | -0.208459218 | Q64133 | Amine oxidase [flavin-containing] A | Maoa | 24.09732056 | 24.12636566 | 24.00735855 | 24.26627922 | 24.37457848 | 24.21556473 |
|  | 0.044161094 | 0.015558243 | O35295 | Transcriptional activator protein Pur-beta | Purb | 24.09079361 | 23.9583683 | 24.01971054 | 24.10053062 | 23.78056717 | 24.14109993 |
|  | 0.445592874 | -0.215131442 | Q9D7S9 | Charged multivesicular body protein 5 | Chmp5 | 24.09022903 | 24.18495369 | NaN | 24.07005501 | 24.58880806 | 24.39930534 |
|  | 0.897409516 | 0.197587967 | Q7TPV4 | Myb-binding protein 1A | Mybbp1a | 24.08277702 | 24.18040657 | 24.28764153 | 23.9519825 | 23.86069679 | 24.14538193 |
|  | 1.629058406 | -0.105155945 | Q3U0V1;A0A3B2WCD8;A0A3B2W465 | Far upstream element-binding protein 2 | Khsrp | 24.08253479 | 24.06562424 | 24.12856865 | 24.15437317 | 24.23161888 | 24.20620346 |
|  | 0.299427581 | 0.063238144 | H7BX95;Q6PDM2;F7AI47;F6QXN3 | Serine/arginine-rich splicing factor 1 | Srsf1 | 24.08204651 | 24.18124199 | 24.20925522 | 24.05482101 | 24.24219894 | 23.98580933 |
|  | 0.858883817 | -0.116207123 | Q9QZE5;Q7TNQ1 | Coatomer subunit gamma-1 | Copg1 | 24.08025932 | 24.04526901 | 24.16560936 | 24.28841591 | 24.11402321 | 24.23731995 |
|  | 0.787482406 | 0.167867661 | Q6PGB6 | N-alpha-acetyltransferase 50 | Naa50 | 24.07960701 | 23.86580276 | 24.00727272 | 23.89458084 | 23.88957596 | 23.66492271 |
|  | 1.27122777 | 0.1489048 | Q3TVK3;Q9Z2W0;Q8BPW9;A0A087WS31;A0A087WSE6;A0A087WRC1;A0A087WSU0;A0A087WNX3;A0A087WSD3 | Aspartyl aminopeptidase | Dnpep | 24.06858063 | 24.14926529 | 24.13437843 | 24.01724815 | 24.01784325 | 23.87041855 |
|  | 0.422039652 | -0.123768489 | Q91VC3;A0A0N4SVP8;E9PV04;A2AFK7;A0A0N4SVM5;D6RJ11 | Eukaryotic initiation factor 4A-III;Eukaryotic initiation factor 4A-III, N-terminally processed | Eif4a3;Gm8994 | 24.06406403 | 23.76799774 | 23.84575081 | 24.17066193 | 24.01350403 | 23.86495209 |
|  | 0.437259245 | 0.077771505 | Q7TQI3;D3YWF6;D3Z7K0 | Ubiquitin thioesterase OTUB1 | Otub1 | 24.06406403 | 24.07651138 | 24.06940079 | 24.14444923 | 23.9205265 | 23.91168594 |
|  | 1.649777687 | 0.192272822 | Q9Z2Y8;A0A1B0GRP7;Q6P8V7;A0A1B0GRR5;Q80ZV3;A0A1B0GSP5;A0A1B0GSA1;A0A1B0GQY5 | Proline synthase co-transcribed bacterial homolog protein | Prosc | 24.0625 | 24.10461617 | 24.11004448 | 23.87098312 | 23.83006668 | 23.99929237 |
|  | 0.811905399 | -0.115092595 | Q8CIH5;E9PXZ8;Q9QZE2 | 1-phosphatidylinositol 4,5-bisphosphate phosphodiesterase gamma-2 | Plcg2 | 24.05912018 | 24.06192207 | 23.91542816 | 24.10053062 | 24.21378517 | 24.0674324 |
|  | 1.046865211 | 0.167660395 | A0A140LHL5;Q8VDQ8 | NAD-dependent protein deacetylase sirtuin-2 | Sirt2 | 24.05804634 | 24.06324005 | 23.97570038 | 23.73252106 | 23.89235878 | 23.96912575 |
|  | 0.500966475 | 0.085290273 | Q9CZN7;G3UZ26;G3UYY1;P50431 | Serine hydroxymethyltransferase | Shmt2 | 24.0578804 | 24.11894035 | 24.24372482 | 23.98433304 | 24.15259552 | 24.0277462 |
|  | 0.379136469 | 0.040987651 | Q8C3J5;Q5SRI3;D6RGU3;Q3TMS1 | Dedicator of cytokinesis protein 2 | Dock2 | 24.05771637 | 24.14273643 | 24.1682148 | 24.04934692 | 24.14336014 | 24.05299759 |
|  | 0.360254911 | -0.068570455 | Q8BH43;B1AUN0 | Wiskott-Aldrich syndrome protein family member 2 | Wasf2 | 24.04809952 | 24.31098175 | 24.18048286 | 24.22743797 | 24.22309875 | 24.29473877 |
|  | 0.560569805 | -0.167155584 | Q6R5N8 | Toll-like receptor 13 | Tlr13 | 24.04626846 | 24.06035614 | 23.89707565 | 23.99576187 | 24.40295029 | 24.10645485 |
|  | 0.392592053 | -0.125799815 | Q9D0S9 | Histidine triad nucleotide-binding protein 2, mitochondrial | Hint2 | 24.04017639 | 23.84536743 | 24.22206688 | 24.32113457 | 24.09972954 | 24.06414604 |
|  | 0.502532614 | 0.131697337 | E9Q586;E9Q3M3;O08788;D3YX34;D3Z2M9;D3YYG9 | Dynactin subunit 1 | Dctn1 | 24.03967476 | 23.83055115 | 23.93992996 | 23.8940258 | 23.91049576 | 23.6105423 |
|  | 0.375485334 | 0.110242208 | Q8VBT6;A0A0U1RNM0;A0A0U1RNE8;A0A0U1RPR7 | Apolipoprotein B receptor | Apobr | 24.03657532 | 23.97962952 | 23.9513588 | 23.8146019 | 23.70988274 | 24.11235237 |
|  | 0.796495491 | 0.159644445 | A0A498WGK6;Q9ET30 | Transmembrane 9 superfamily member 3 | Tm9sf3 | 24.03456306 | 23.86542511 | 23.95331383 | 23.94503021 | 23.68506622 | 23.74427223 |
|  | 0.139692223 | -0.020204544 | Q9EP69;A0A5F8MPK9;A0A1L1SR59 | Phosphatidylinositide phosphatase SAC1 | Sacm1l | 24.03330231 | 24.21793175 | 24.12526131 | 24.15530014 | 24.14180183 | 24.14000702 |
|  | 0.317666159 | 0.1149985 | P10922 | Histone H1.0;Histone H1.0, N-terminally processed | H1f0 | 24.02301407 | 24.04067802 | 24.18669128 | 23.69072533 | 24.1091671 | 24.10549545 |
|  | 0.154940683 | 0.038235346 | P51125;A0A338P6J9;Q8CE80;Q921U7;Q8C281;Q8CE04;A0A1Y7VJN8 | Calpastatin | Cast | 24.0202198 | 24.21955872 | 24.10924721 | 23.94931221 | 24.08642769 | 24.19857979 |
|  | 0.171775262 | -0.039918264 | P09055 | Integrin beta-1 | Itgb1 | 24.02005005 | 24.04401779 | 24.08642769 | 23.9745636 | 24.03808403 | 24.25760269 |
|  | 1.335065218 | 0.230721792 | P55264;A0A286YCD7;A0A286YCN3 | Adenosine kinase | Adk | 24.0140152 | 23.79471397 | 23.91734314 | 23.59000778 | 23.7635479 | 23.68035126 |
|  | 0.517058512 | -0.179322561 | Q91V77;P56565;D3YUT6 | Protein S100;Protein S100-A1 | S100a1 | 24.01188469 | 23.58726311 | 23.77293777 | 23.99498558 | 24.11155701 | 23.80351067 |
|  | 0.151339351 | -0.01335144 | Q8JZQ9;A0A0G2JG48 | Eukaryotic translation initiation factor 3 subunit B | Eif3b | 24.00932503 | 24.06808853 | 24.05531693 | 24.03523445 | 24.11259079 | 24.02495956 |
|  | 0.646815149 | 0.102228165 | P17918 | Proliferating cell nuclear antigen | Pcna | 24.00701523 | 24.00872612 | 23.90416908 | 23.8813839 | 23.75806618 | 23.97377586 |
|  | 0.484559369 | -0.079004924 | Q61081;A0A1L1STC0 | Hsp90 co-chaperone Cdc37;Hsp90 co-chaperone Cdc37, N-terminally processed | Cdc37 | 24.00633049 | 24.09595299 | 24.16138649 | 24.21475029 | 24.22846603 | 24.05746841 |
|  | 0.002844328 | 0.000829061 | A0A5F8MPP7;A2AAY5 | SH3 and PX domain-containing protein 2B | Sh3pxd2b | 24.00590324 | 23.9197979 | 23.84201431 | 24.07463455 | 23.89975166 | 23.79084206 |
|  | 0.084902214 | 0.027410507 | P38060;V9GXD2;V9GWZ9;D3YVU9;Q8JZS7 | Hydroxymethylglutaryl-CoA lyase, mitochondrial | Hmgcl | 23.99929237 | 23.96956635 | 23.65436172 | 23.90609741 | 23.80685806 | 23.82803345 |
|  | 0.127265701 | 0.026847204 | Q5SUH7;Q5SUH6;Q99KN9 | Clathrin interactor 1 | Clint1 | 23.99740028 | 24.06389809 | 23.94243813 | 23.89938354 | 24.11203384 | 23.9117775 |
|  | 0.028261384 | 0.009696325 | Q99KP6 | Pre-mRNA-processing factor 19 | Prpf19 | 23.99187469 | 23.79431725 | 24.02462196 | 24.06554222 | 23.75816727 | 23.95801544 |
|  | 0.503764714 | -0.370290756 | A0A1Y7VJ48;P48428 | Tubulin-specific chaperone A | Tbca | 23.98780441 | 24.82953453 | 24.82425117 | 25.22269249 | 24.69131088 | 24.83845901 |
|  | 0.117616522 | 0.035217921 | Q9CXW3;A0A0A6YY29 | Calcyclin-binding protein | Cacybp | 23.98320198 | 23.88613701 | 23.67648315 | 23.69200325 | 23.87370682 | 23.87445831 |
|  | 0.972968933 | 0.13478152 | Q64727 | Vinculin | Vcl | 23.98207092 | 23.8894825 | 23.96947861 | 23.91533661 | 23.80695534 | 23.71439552 |
|  | 0.475613539 | -0.085076014 | Q8BT60;V9GX86;B7ZCP8;V9GWY2;Q9D6C8;A0A0R4J0J1;Q3UYN2;Q1RLL3;Q9Z140;Q8BLR2;Q0VE82;Q9DC53;Q8JZW4 | Copine-3 | Cpne3 | 23.9791069 | 23.88994789 | 23.77424622 | 23.99593353 | 24.03405952 | 23.868536 |
|  | 0.364063895 | -0.091866811 | G5E8V9;E9QAY5 | Arfaptin-1 | Arfip1 | 23.97342491 | 24.33510971 | 24.16215515 | 24.2448864 | 24.2269249 | 24.27447891 |
|  | 0.277732089 | 0.049152374 | Q6ZQI3 | Malectin | Mlec | 23.97307396 | 24.01758766 | 23.90370941 | 23.96982956 | 23.79014587 | 23.98693848 |
|  | 1.01494379 | 0.264307658 | Q9D8Y7 | Tumor necrosis factor alpha-induced protein 8-like protein 2 | Tnfaip8l2 | 23.97105789 | 24.09643555 | 23.81284142 | 23.6365509 | 23.87370682 | 23.57715416 |
|  | 0.007977098 | -0.001005173 | D3YZ98;E9QLS6;Q6PF93;E9Q824 | Phosphatidylinositol 3-kinase;Phosphatidylinositol 3-kinase catalytic subunit type 3 | Pik3c3 | 23.9699173 | 24.02369118 | 23.99662399 | 23.94181061 | 23.98015404 | 24.07128334 |
|  | 0.379376169 | -0.112807592 | Q8R146;A0A0R4J107;A0A0A6YXV0 | Acylamino-acid-releasing enzyme | Apeh | 23.96947861 | 23.81811905 | 23.67400551 | 23.86740494 | 24.11362457 | 23.81899643 |
|  | 0.572734104 | 0.253972371 | G3UZY2;P97493;G3UX99;A2A439 | Thioredoxin, mitochondrial | Txn2 | 23.96446228 | 23.96569633 | 23.99946594 | 23.47568321 | 24.11187553 | 23.5801487 |
|  | 0.677525328 | -0.099688212 | P54923 | [Protein ADP-ribosylarginine] hydrolase | Adprh | 23.95650864 | 23.83961296 | 23.80587387 | 23.99567604 | 23.87182999 | 24.03355408 |
|  | 0.628360768 | 0.107351303 | Q3TXV4;Q921E2;Q3TM43;D6RFT6 | Ras-related protein Rab-31 | Rab31 | 23.9564209 | 23.92506218 | 23.73355484 | 23.82123756 | 23.7635479 | 23.70819855 |
|  | 0.937908624 | -0.130948385 | O88746;Q3UDC3;A0A1D5RM45;A0A1D5RL90;A0A1D5RM84 | Target of Myb protein 1 | Tom1 | 23.95464706 | 23.83151817 | 23.84306908 | 24.01758766 | 23.91232491 | 24.0921669 |
|  | 0.464605948 | 0.070127487 | P23780;A0A1L1SSJ7 | Beta-galactosidase | Glb1 | 23.9536705 | 23.97709846 | 23.8490963 | 23.78656197 | 23.95828056 | 23.82464027 |
|  | 0.007825558 | -0.002688726 | Q3TCU5;Q9R233;G3UZZ2;A0A3Q4EGL0 | Tapasin | Tapbp | 23.95100403 | 24.00761414 | 24.11513519 | 24.10485649 | 23.82483292 | 24.15213013 |
|  | 0.623016931 | 0.143802643 | P11404 | Fatty acid-binding protein, heart | Fabp3 | 23.93696785 | 23.93903351 | 24.10940742 | 23.79887199 | 24.02064323 | 23.73448563 |
|  | 1.128902252 | 0.104155223 | A2A9X5;Q9JM14 | 5(3)-deoxyribonucleotidase, cytosolic type | Nt5c | 23.93499184 | 23.99757195 | 23.88166428 | 23.84354782 | 23.78206825 | 23.87614632 |
|  | 1.384860098 | 0.152547836 | O70310;F7APP3;A2AJH3;O70311 | Glycylpeptide N-tetradecanoyltransferase 1 | Nmt1 | 23.93292046 | 24.03716278 | 23.9671917 | 23.86778259 | 23.86778259 | 23.74406624 |
|  | 1.010251654 | 0.285603205 | A0A0R4J1Z3;Q9CR67;Q9CZM3;E0CXY5;A0A0J9YV78 | Transmembrane protein 33 | Tmem33 | 23.92813873 | 24.02892685 | 23.81303787 | 23.81039238 | 23.68848419 | 23.41441727 |
|  | 0.082660394 | 0.023412069 | Q64324;F8WGM5;A0A140LJ60 | Syntaxin-binding protein 2 | Stxbp2 | 23.92043495 | 23.7875576 | 23.83209801 | 23.81411362 | 23.66828156 | 23.98745918 |
|  | 1.394077527 | 0.117483775 | P50396;B7FAU8;D6RI86 | Rab GDP dissociation inhibitor alpha | Gdi1 | 23.91561127 | 23.88054276 | 23.89799881 | 23.71795654 | 23.77464867 | 23.8490963 |
|  | 0.233243164 | -0.033571879 | Q8R317 | Ubiquilin-1 | Ubqln1 | 23.91469955 | 23.98328781 | 23.93409157 | 23.9419899 | 24.08131599 | 23.90948868 |
|  | 1.363555545 | 0.225669225 | O35343;A0A0B4J1E7;D3YTN1 | Importin subunit alpha-3 | Kpna4 | 23.91305542 | 23.68891144 | 23.69402313 | 23.52145195 | 23.58497238 | 23.51255798 |
|  | 0.255823386 | 0.032648087 | P54822;E9Q242;E9Q3T7;E9Q0A0;A0A140LHU1;A0A0G2JFI8;A0A0G2JFL8 | Adenylosuccinate lyase | Adsl | 23.91296387 | 24.00281715 | 24.00109863 | 23.9196167 | 23.88054276 | 24.01877594 |
|  | 0.104375437 | -0.010949453 | P14685;F7B7L8 | 26S proteasome non-ATPase regulatory subunit 3 | Psmd3 | 23.9045372 | 23.85148048 | 23.95313644 | 23.96093369 | 23.88380814 | 23.89726067 |
|  | 0.137613191 | -0.021944682 | E9Q137 | Testis-expressed protein 264 homolog | Tex264 | 23.9004879 | 23.92043495 | 24.02199936 | 23.92813873 | NaN | 24.0110321 |
|  | 1.488332416 | 0.235801697 | Q9WTX5;E9PUV4 | S-phase kinase-associated protein 1 | Skp1 | 23.89772224 | 23.83229065 | 23.96066856 | 23.74581146 | 23.53718185 | 23.70028305 |
|  | 0.604212542 | 0.1455911 | Q99L27;A0A2I3BQX8;A0A2I3BRT9 | GMP reductase 2 | Gmpr2 | 23.89522743 | 23.79837799 | 23.71565437 | 23.81967926 | 23.49179459 | 23.66101265 |
|  | 0.062622868 | -0.016658147 | P70444;D3Z463 | BH3-interacting domain death agonist;BH3-interacting domain death agonist p15;BH3-interacting domain death agonist p13;BH3-interacting domain death agonist p11 | Bid | 23.89300728 | 23.97543716 | 24.08367157 | 23.90857315 | 24.14732552 | 23.94619179 |
|  | 1.051596615 | -0.069261551 | Q9ES52;F7BZA7 | Phosphatidylinositol 3,4,5-trisphosphate 5-phosphatase 1 | Inpp5d | 23.89059639 | 23.84066963 | 23.86994934 | 23.88287735 | 23.95304871 | 23.97307396 |
|  | 0.563791003 | 0.072905858 | Q9D0F9;A2CEK3 | Phosphoglucomutase-1 | Pgm1;Pgm2 | 23.89022636 | 23.79263115 | 23.76435661 | 23.66025162 | 23.80449486 | 23.76375008 |
|  | 0.216724888 | 0.040255864 | Q64514;A0A087WRC0;Q05DJ9;A0A087WP19;A0A087WQR6 | Tripeptidyl-peptidase 2 | Tpp2 | 23.88753319 | 23.97298622 | 23.9380455 | 24.01988029 | 23.86976051 | 23.78815651 |
|  | 0.393154094 | 0.065449397 | Q8C0C7;E9PWY9;D6RIJ2 | Phenylalanine--tRNA ligase alpha subunit | Farsa | 23.88222313 | 23.72754288 | 23.86060143 | 23.78206825 | 23.6601429 | 23.83180809 |
|  | 0.69942437 | -0.206914902 | Q9D1J1;F6RCM5 | Adaptin ear-binding coat-associated protein 2 | Necap2 | 23.8818512 | 23.84115028 | 24.16438293 | 24.02690315 | 24.33203888 | 24.14918709 |
|  | 1.009178947 | 0.397224426 | Q9CR68 | Cytochrome b-c1 complex subunit Rieske, mitochondrial;Cytochrome b-c1 complex subunit 11 | Uqcrfs1 | 23.87867355 | 23.84995461 | 23.54733849 | NaN | 23.23265839 | 23.49020386 |
|  | 0.179002268 | -0.011250178 | Q3UPL0;S4R2A9;S4R192;S4R256;S4R1T5;S4R1Y8 | Protein transport protein Sec31A | Sec31a | 23.87811279 | 23.82016563 | 23.88054276 | 23.89578247 | 23.84957314 | 23.86721611 |
|  | 0.002302185 | 0.000588735 | P13597 | Intercellular adhesion molecule 1 | Icam1 | 23.87698936 | 23.63023186 | 23.87286186 | 23.76172256 | 23.8237648 | 23.79282951 |
|  | 0.573528861 | -0.115913391 | Q9JHJ0;A0A1L1SQ12;A0A1L1SR42 | Tropomodulin-3 | Tmod3 | 23.8747406 | 23.68474579 | 23.94360161 | 23.89393234 | 23.91588402 | 24.04101181 |
|  | 0.407941752 | 0.099761963 | Q80W54;I7HIP5 | CAAX prenyl protease 1 homolog | Zmpste24 | 23.8747406 | 23.8133316 | 23.83624458 | 23.53812981 | 23.82667732 | 23.86022377 |
|  | 1.133224681 | 0.129058202 | Q8CI51;D9J2Z9;D9J300;D9J301;D9J302;A0A0G2JGZ5;A0A0G2JEJ0;Q9CRA2;D9J303;F8WJI6;E9Q8P5 | PDZ and LIM domain protein 5 | Pdlim5 | 23.86778259 | 24.04276657 | 23.95978355 | 23.82832527 | 23.79669571 | 23.85813713 |
|  | 1.820976662 | 0.203577677 | Q8BH59;V9GXX9 | Calcium-binding mitochondrial carrier protein Aralar1 | Slc25a12 | 23.86684036 | 23.98320198 | 23.84871292 | 23.70334816 | 23.73820114 | 23.64647293 |
|  | 0.690434594 | -0.144744873 | B7ZCU2;B7ZCU5;B7ZCU4;B7ZCU3;B7ZCU0;J3QNK8;Q8CBW3;A0A087WNT3;A0A087WP64;A0A087WPE6;A0A087WNU9 | Abl interactor 1 | Abi1 | 23.86608505 | 24.11283112 | 24.09820366 | 24.26341629 | 24.1649971 | 24.08294106 |
|  | 1.198969908 | -0.103425344 | Q9Z1Q9;G3UY93;G3UZ22;G3UYW2;G3UZX1 | Valine--tRNA ligase | Vars | 23.86315727 | 23.76951218 | 23.8707943 | 23.88966942 | 23.95978355 | 23.9642868 |
|  | 0.344528194 | -0.103833516 | G3X9Q3;Q3TBD2 | Minor histocompatibility protein HA-1 | Hmha1 | 23.85889626 | 24.10709381 | 23.92687225 | 23.87220573 | 24.12707329 | 24.20508385 |
|  | 1.119418357 | 0.120079676 | Q99JR1 | Sideroflexin-1 | Sfxn1 | 23.8582325 | 23.85195732 | 23.92614746 | 23.77535057 | 23.82609558 | 23.6746521 |
|  | 1.065575768 | 0.131666819 | Q8BU30;E9Q866;F6Q6R1 | Isoleucine--tRNA ligase, cytoplasmic | Iars | 23.85595322 | 23.70609283 | 23.76162148 | 23.61963654 | 23.71753693 | 23.59149361 |
|  | 0.453332429 | 0.051015218 | P97823;J3QP56;D3YUG4;D3Z111;D3Z269;J3QQ63 | Acyl-protein thioesterase 1 | Lypla1 | 23.85309982 | 23.9998951 | 23.97114563 | 23.92542458 | 23.86391258 | 23.88175774 |
|  | 0.740353613 | -0.056325277 | Q9CZD3 | Glycine--tRNA ligase | Gars | 23.85271835 | 23.89661407 | 23.88278389 | 23.87041855 | 23.97744751 | 23.95322609 |
|  | 0.317114515 | -0.090384165 | Q60749;A0A087WSL3;A0A087WR32;A0A2R8VJR4;Q9R226;Q9WU01 | KH domain-containing, RNA-binding, signal transduction-associated protein 1 | Khdrbs1 | 23.85062218 | 23.9452095 | 23.90288162 | 23.87708282 | 24.21682358 | 23.8759594 |
|  | 0.915039935 | -0.124359131 | Q9D3L3;O09044;B0R030;B0R029;E9Q8A1;P60879 | Synaptosomal-associated protein;Synaptosomal-associated protein 23 | Snap23 | 23.8469944 | 23.90719795 | 23.96322823 | 24.06759644 | 24.09892654 | 23.92397499 |
|  | 0.632123137 | 0.059935252 | P14733 | Lamin-B1 | Lmnb1 | 23.84450531 | 23.89115334 | 23.96763229 | 23.87858009 | 23.79877281 | 23.84613228 |
|  | 1.107942419 | 0.175206502 | Q91VH6;A0A3B2W7C9;A0A3F2YNL8;A0A3B2W4I2;A0A3B2WCC1;A0A3B2W4G7 | Protein MEMO1 | Memo1 | 23.84210968 | 23.69019127 | 23.87427139 | 23.57149506 | 23.58680534 | 23.72265244 |
|  | 0.917388869 | 0.185907364 | Q9D6Z1;F7CHQ7;F6U250;F6V095;E0CXZ0;A2APD7;F7CHP9;F6USW7 | Nucleolar protein 56 | Nop56 | 23.83932495 | 23.77826309 | 23.98441887 | 23.54014206 | 23.77826309 | 23.72587967 |
|  | 0.125776154 | -0.022742589 | O55022 | Membrane-associated progesterone receptor component 1 | Pgrmc1 | 23.83855629 | 23.81733894 | 23.80538177 | 23.71397781 | 23.92660141 | 23.88892555 |
|  | 0.334944589 | 0.188003858 | Q3TYS2;A0A0A0MQK0;E9Q3W5;X1WI15 | Uncharacterized protein C17orf62 homolog | BC017643 | 23.83807373 | 23.87088966 | 23.63267326 | NaN | 23.86353493 | 23.32154846 |
|  | 1.364803328 | -0.132628759 | Q8BL66;A0A1W2P7A6 | Early endosome antigen 1 | Eea1 | 23.83797836 | 23.81626511 | 23.69412994 | 23.9004879 | 23.92170715 | 23.92406464 |
|  | 0.198299853 | -0.056071599 | Q3TC93;A0A1W2P6R8;A0A1W2P6H5 | HCLS1-binding protein 3 | Hs1bp3 | 23.83778572 | 23.83064842 | 23.98294067 | 23.74837303 | 24.06044006 | 24.01077652 |
|  | 0.533686056 | -0.056983312 | Q61035;A0A494BAV2;A0A494B9A1 | Histidine--tRNA ligase, cytoplasmic | Hars | 23.83325577 | 23.79143906 | 23.77122498 | 23.93885422 | 23.79282951 | 23.835186 |
|  | 1.512475278 | -0.212099075 | Q9DCC5;P23198;D3Z1A9;D3Z313 | Chromobox protein homolog 3 | Cbx3 | 23.83209801 | 23.83614922 | 23.87726974 | 23.95411301 | 24.05490303 | 24.17279816 |
|  | 0.000336345 | 5.09E-05 | Q11011;F6QYF8;E9Q039;E9Q6F4;F6V7K3;F2Z3V5;F7ANF4 | Puromycin-sensitive aminopeptidase | Npepps | 23.82871246 | 23.9474411 | 23.88576508 | 23.89735222 | 23.94288445 | 23.82152939 |
|  | 0.31065406 | -0.056868235 | G3X8Y3;A0A0A6YW80;Q80UM3;A0A0A6YX86;A0A0A6YXF4;E9PZ53;Q9DBB4 | N-alpha-acetyltransferase 15, NatA auxiliary subunit | Naa15 | 23.82842255 | 23.58623314 | 23.75185013 | 23.79808044 | 23.73551941 | 23.80351067 |
|  | 0.134485047 | 0.054819107 | Q9Z2N8;D3YVN1;A0A0A6YWG8;A0A0A6YWR1 | Actin-like protein 6A | Actl6a | 23.82667732 | 23.70461464 | 23.43948936 | 23.6537075 | 23.73923302 | 23.41338348 |
|  | 1.191958966 | 0.159054438 | Q8K2Q9 | Shootin-1 | Kiaa1598 | 23.82648277 | 23.89559746 | 23.93939209 | 23.68731117 | 23.83402824 | 23.66296959 |
|  | 0.747357505 | -0.169299444 | O35601;A0A2I3BRH1;A0A2I3BPY6 | FYN-binding protein | Fyb | 23.82211304 | 23.88297081 | 23.92225075 | 24.1165638 | 23.84785461 | 24.17081451 |
|  | 1.184234925 | 0.078605016 | Q99MN1;Q8R2P8 | Lysine--tRNA ligase | Kars | 23.82152939 | 23.88985443 | 23.88994789 | 23.79362297 | 23.74929428 | 23.82259941 |
|  | 0.232027927 | 0.047333399 | O35114 | Lysosome membrane protein 2 | Scarb2 | 23.82026291 | 23.77243423 | 23.74591446 | 23.59730148 | 23.86410141 | 23.73520851 |
|  | 2.072952113 | -0.152964274 | Q9Z1Z2 | Serine-threonine kinase receptor-associated protein | Strap | 23.81821632 | 23.8454628 | 23.84115028 | 24.04851532 | 23.95144844 | 23.96375847 |
|  | 0.019819209 | 0.004643122 | Q99J77 | Sialic acid synthase | Nans | 23.81215668 | 23.91387749 | 23.95978355 | 23.8047905 | 24.01733398 | 23.84976387 |
|  | 0.702421435 | -0.082253774 | Q60972 | Histone-binding protein RBBP4 | Rbbp4 | 23.81127357 | 23.76304054 | 23.67271233 | 23.89272881 | 23.82842255 | 23.77263641 |
|  | 0.069484538 | 0.009206772 | Q80X50;A0A0H2UH17;A0A0G2JDV6;A0A0G2JG47;A0A0G2JGD0;A0A0G2JDT1;A0A0G2JFN7;A0A0G2JE24;A0A0G2JEC6 | Ubiquitin-associated protein 2-like | Ubap2l | 23.80754471 | 23.79768562 | 23.85614395 | 23.88827515 | 23.80449486 | 23.74098396 |
|  | 0.473425149 | -0.116123835 | Q8K1T1 | Leucine-rich repeat-containing protein 25 | Lrrc25 | 23.80705452 | 23.6552372 | 23.55672264 | 23.94297409 | 23.69582939 | 23.72858238 |
|  | 1.274337895 | 0.250456492 | Q9CQ62 | 2,4-dienoyl-CoA reductase, mitochondrial | Decr1 | 23.80587387 | 23.88474083 | 23.74540138 | 23.40028191 | 23.67551422 | 23.60885048 |
|  | 0.676642481 | -0.203610102 | P49962;D3YZX8 | Signal recognition particle 9 kDa protein | Srp9 | 23.80459404 | 23.55566978 | 23.43631554 | 23.68120956 | 23.7645607 | 23.9616394 |
|  | 0.014167221 | 0.003911336 | P35585;A0A1D5RLW6;D3YZ71;A0A0R4IZX6;A0A0R4J1L4;Q9WVP1;A0A1D5RLL9;A0A1D5RMJ1;A0A1L1SUA0 | AP-1 complex subunit mu-1 | Ap1m1 | 23.80341148 | 23.87745667 | 24.01818275 | 23.98276711 | 23.93867493 | 23.76587486 |
|  | 1.917153697 | -0.121378581 | A0A087WPL5;E9QNN1;O70133;Q3UR42;A0A0R4J2C3;A0A087WRT3 | ATP-dependent RNA helicase A | Dhx9 | 23.79560661 | 23.86079216 | 23.82483292 | 23.94055748 | 23.98728561 | 23.91752434 |
|  | 0.002745635 | 0.000444412 | Q9JHI5 | Isovaleryl-CoA dehydrogenase, mitochondrial | Ivd | 23.79302788 | 23.76111412 | 23.87820625 | 23.7551136 | 23.88734627 | 23.78855515 |
|  | 0.60012407 | -0.054051717 | Q9JJ28 | Protein flightless-1 homolog | Flii | 23.79282951 | 23.85386086 | 23.79738808 | 23.86994934 | 23.80715179 | 23.92913246 |
|  | 0.134905663 | 0.057553609 | Q99LF4 | tRNA-splicing ligase RtcB homolog | Rtcb | 23.78286743 | 23.95109177 | 24.05763245 | 24.11171722 | 23.86448097 | 23.64273262 |
|  | 0.273751388 | 0.136425018 | A0A0G2JEC4;Q9JK48;A0A0G2JF57;A0A0G2JE45 | Endophilin-B1 | Sh3glb1 | 23.77454758 | 23.54686737 | 23.2844677 | 23.21706009 | 23.67669868 | 23.30284882 |
|  | 0.102720531 | -0.035529455 | A0A0R4J0U7;Q8R395 | COMM domain-containing protein 5 | Commd5 | 23.77263641 | 24.06027412 | 23.97936821 | 23.90637398 | 24.15151215 | 23.86098099 |
|  | 0.515805801 | -0.139968236 | Q8BYI6 | Lysophosphatidylcholine acyltransferase 2 | Lpcat2 | 23.76405334 | 23.58932304 | 23.97771072 | 23.94422722 | 23.83913231 | 23.96763229 |
|  | 1.081287116 | 0.087110519 | Q6A0A9 | Constitutive coactivator of PPAR-gamma-like protein 1 | FAM120A | 23.7634449 | 23.68324661 | 23.74570847 | 23.63876152 | 23.59605217 | 23.69625473 |
|  | 0.08070216 | 0.021760305 | P46467;Q8BPY9 | Vacuolar protein sorting-associated protein 4B | Vps4b | 23.76141739 | 23.60749817 | 23.59843826 | 23.7522583 | 23.4836998 | 23.66611481 |
|  | 0.529809591 | -0.191742579 | P61222 | ATP-binding cassette sub-family E member 1 | Abce1 | 23.75694656 | 23.77705956 | 23.71753693 | 23.85880089 | 23.71931458 | 24.24865532 |
|  | 1.179973725 | -0.304370244 | Q3U1Z5 | G-protein-signaling modulator 3 | Gpsm3 | 23.75684547 | 23.76557159 | 23.91104507 | 24.33599472 | 24.01809692 | 23.99248123 |
|  | 1.1619876 | -0.1052742 | Q9D662;A2ANA0;A2AN97;A2AN98;A2AN99 | Protein transport protein Sec23B | Sec23b | 23.75674438 | 23.66264343 | 23.73851204 | 23.76526833 | 23.83643723 | 23.87201691 |
|  | 0.295389817 | 0.089368184 | Q9CZ04;D3Z440;D3Z0S0;D3YVI6 | COP9 signalosome complex subunit 7a | Cops7a | 23.74837303 | 23.54144096 | 23.67874146 | 23.5861187 | 23.74088097 | 23.37345123 |
|  | 0.598799089 | -0.115390142 | Q9D8S5;O35326 | Serine/arginine-rich splicing factor 5 | Srsf5 | 23.74519539 | 23.75256348 | 23.85300446 | 23.78436661 | 24.05026054 | 23.86230659 |
|  | 0.30697899 | -0.096365611 | A0A0R4J079;Q8BMP6 | Golgi resident protein GCP60 | Acbd3 | 23.7449913 | 23.63854027 | 23.58394051 | 23.62053299 | 23.98945236 | 23.64658356 |
|  | 0.760299134 | -0.254434268 | Q6P542 | ATP-binding cassette sub-family F member 1 | Abcf1 | 23.74324417 | 23.91633987 | 24.05482101 | NaN | 24.27120399 | 24.04726791 |
|  | 0.925416743 | 0.110061646 | E9Q9H2;P54103;E9Q9H3;Q3TRX6 | DnaJ homolog subfamily C member 2;DnaJ homolog subfamily C member 2, N-terminally processed | Dnajc2 | 23.73902702 | 23.55414772 | 23.61874199 | 23.54674911 | 23.53076744 | 23.50421524 |
|  | 1.162855965 | 0.107645035 | P29452;A0A571BDI1;A0A571BF22 | Caspase-1;Caspase-1 subunit p20;Caspase-1 subunit p10 | Casp1 | 23.73458862 | 23.75827026 | 23.81303787 | 23.69657326 | 23.69901085 | 23.58737755 |
|  | 1.459840348 | -0.186056773 | Q6P1F6;Q9CWU3;Q925E7;F6RV17;A0A668KLA9;Q6ZWR4;A0A286YDJ9;A0A494B941;G3UXS9;A0A494B8Z0;Q8BG02 | Serine/threonine-protein phosphatase 2A 55 kDa regulatory subunit B alpha isoform;Serine/threonine-protein phosphatase 2A 55 kDa regulatory subunit B | Ppp2r2a | 23.73075867 | 23.89642906 | 23.8643856 | 24.03246307 | 23.95774841 | 24.05953217 |
|  | 1.434718653 | -0.183549245 | Q9ET01;Q3UEJ6 | Glycogen phosphorylase, liver form;Alpha-1,4 glucan phosphorylase | Pygl | 23.72795868 | 23.60636902 | 23.52540016 | 23.79659653 | 23.82065201 | 23.79312706 |
|  | 0.253448812 | 0.091094335 | Q3UIA2;E9QAJ9;A0A0U1RNM6;A0A0U1RPK6;Q5SSM3 | Rho GTPase-activating protein 17 | Arhgap17 | 23.72723198 | 23.99420929 | 23.95677376 | 24.02038765 | 23.62678337 | 23.757761 |
|  | 0.765572495 | 0.189325968 | Q9D819 | Inorganic pyrophosphatase | Ppa1 | 23.72442436 | 23.91697884 | 23.90948868 | 23.53575897 | 23.60025597 | 23.84689903 |
|  | 1.032921556 | -0.39451472 | A0A140LHP7;O54885 | TYRO protein tyrosine kinase-binding protein | Tyrobp | 23.72265244 | 23.62287903 | NaN | 24.02191353 | 24.29761124 | 23.88231659 |
|  | 0.157224737 | -0.031749725 | Q3U1J4;A0A494B987;A0A494BAF6;A0A494BBJ6 | DNA damage-binding protein 1 | Ddb1 | 23.72119331 | 23.46513176 | 23.55403137 | 23.6300087 | 23.60715866 | 23.59843826 |
|  | 0.646627155 | 0.184319814 | P97742;A0A494BAD3;A0A494BAC7 | Carnitine O-palmitoyltransferase 1, liver isoform | Cpt1a | 23.7205677 | 23.99550247 | 24.06956291 | 23.6028614 | 23.78396606 | 23.84584618 |
|  | 0.787275884 | -0.164173762 | E9PWZ6;A0A1D5RLW4;A0A8V5KX73;E9PZW8;Q9QY06 | Unconventional myosin-IXb | Myo9b | 23.71920967 | 23.83662987 | 23.56092453 | 23.90765762 | 23.76385117 | 23.93777657 |
|  | 1.527010517 | 0.134910583 | Q9CPY7;A0A0G2JEM7 | Cytosol aminopeptidase | Lap3 | 23.71900177 | 23.80104446 | 23.70767212 | 23.64405441 | 23.55203819 | 23.626894 |
|  | 0.240854709 | 0.043471654 | Q8R5C5;E0CZD4;E0CYB4 | Beta-centractin | Actr1b | 23.71774673 | 23.48296165 | 23.64152145 | 23.56720734 | 23.54333115 | 23.6012764 |
|  | 1.14583007 | -0.32891655 | P70404 | Isocitrate dehydrogenase [NAD] subunit gamma 1, mitochondrial | Idh3g | 23.7164917 | 23.75745583 | 24.05812836 | 24.06175804 | 24.33149147 | 24.12557602 |
|  | 0.705584541 | 0.104825974 | A2ADY9 | Protein DDI1 homolog 2 | Ddi2 | 23.71397781 | 23.6905117 | 23.5025177 | 23.51340103 | 23.528265 | 23.55086327 |
|  | 0.618495113 | 0.168592453 | P58389;A2AWF0;A2AWE9;B7ZDE0;D6RFC2;F6Z6I0 | Serine/threonine-protein phosphatase 2A activator | Ppp2r4 | 23.71376801 | 23.96939087 | 23.94503021 | 23.88966942 | 23.60195541 | 23.6307869 |
|  | 0.25021093 | -0.052535057 | Q62376;A0A1B0GRR8;A0A1B0GR69;A0A1B0GR44;A0A1B0GRV8 | U1 small nuclear ribonucleoprotein 70 kDa | Snrnp70 | 23.7136631 | 23.83634186 | NaN | 23.82036018 | 23.74190903 | 23.9203434 |
|  | 0.576893872 | -0.275192897 | Q61781;Q9D312 | Keratin, type I cytoskeletal 14 | Krt14 | 23.7135582 | 23.18360519 | 23.62734032 | 23.75235939 | 23.56685829 | 24.03086472 |
|  | 0.492257356 | 0.147947311 | H3BJU7;H3BJ45;H3BJ40;H3BKH9;H3BJX8;Q60875;H3BL15;H3BLF9;H3BJ59 | Rho guanine nucleotide exchange factor 2 | Arhgef2 | 23.71292877 | 23.37132454 | 23.51581001 | 23.29572105 | 23.303545 | 23.55695534 |
|  | 0.079627629 | 0.012458165 | Q9CQF9;D3Z275;F7CIP8;A0A0N4SWD3 | Prenylcysteine oxidase | Pcyox1 | 23.71240425 | 23.60195541 | 23.7565403 | 23.73520851 | 23.6673069 | 23.63101006 |
|  | 0.216970233 | 0.091121674 | Q9QUR7;Q3ULQ2 | Peptidyl-prolyl cis-trans isomerase NIMA-interacting 1 | Pin1 | 23.70756721 | 23.68035126 | 23.69529915 | 23.5480442 | 23.35231972 | 23.90948868 |
|  | 1.351792953 | 0.257024765 | P18181;F8WHM0 | CD48 antigen | Cd48 | 23.70619774 | 23.73768616 | 23.89504242 | 23.58428383 | 23.59479904 | 23.38876915 |
|  | 0.505566717 | -0.072574615 | Z4YKB8;Q3TEA8;Z4YKA3;A2AM70;A2AM65;A2AM62;A2AM63;A2AM69 | Heterochromatin protein 1-binding protein 3 | Hp1bp3 | 23.70493126 | 23.54049683 | 23.59069443 | 23.61481667 | 23.6866703 | 23.75235939 |
|  | 0.134193392 | -0.019866943 | Q61029 | Lamina-associated polypeptide 2, isoforms beta/delta/epsilon/gamma | Tmpo | 23.70366478 | 23.68196106 | 23.74273109 | 23.65053368 | 23.8263855 | 23.71103859 |
|  | 0.226055046 | -0.053572973 | Q9CPV4;E9Q197;F6ZTG3;E9Q055;E9Q2R6;F7BB55 | Glyoxalase domain-containing protein 4 | Glod4 | 23.70207977 | 23.47134781 | 23.6831398 | 23.60557747 | 23.78336716 | 23.62834167 |
|  | 1.146863579 | 0.226950963 | A0A087WQS2;Q9CQC6;A0A087WPF9;A0A087WS48 | Basic leucine zipper and W2 domain-containing protein 1 | Bzw1 | 23.70070648 | 23.95287132 | 23.80301857 | 23.60263634 | 23.68560028 | 23.48750687 |
|  | 0.011464051 | 0.004589081 | Q61655;Q9QY15 | ATP-dependent RNA helicase DDX19A | Ddx19a | 23.6996479 | 23.43974304 | 23.39270401 | 23.43338966 | 23.6875248 | 23.39741325 |
|  | 0.035972119 | -0.01301829 | Q91W86;A0A1L1SRH3;A0A1L1SQ62;A0A1L1SV27 | Vacuolar protein sorting-associated protein 11 homolog | Vps11 | 23.69487381 | 23.34502792 | 23.50057411 | 23.61874199 | 23.39126205 | 23.56952667 |
|  | 0.549291503 | -0.120241165 | O55222;D3YZA5;A0A1B0GRF6;A0A1B0GR42 | Integrin-linked protein kinase | Ilk | 23.69104385 | 23.65610886 | 23.54733849 | 23.74734879 | 23.60387993 | 23.90398598 |
|  | 0.270276273 | -0.094484965 | Q8VBT0;F6V084 | Thioredoxin-related transmembrane protein 1 | Tmx1 | 23.68955231 | 23.84210968 | 23.40132523 | 23.64107895 | 23.74878311 | 23.82658005 |
|  | 1.664609714 | -0.201295853 | Q5SUS9;Q61545;Q5SUT0;Q5SUS8 | RNA-binding protein EWS | Ewsr1 | 23.6894455 | 23.81938553 | 23.85186195 | 24.00710106 | 24.01665306 | 23.94082642 |
|  | 0.293230043 | -0.11473465 | A0A0J9YUI1;P61087;A0A0J9YU07;Q3V3R8;A0A0J9YUR9;D3Z4U3;F8WIC2 | Ubiquitin-conjugating enzyme E2 K | Ube2k | 23.6875248 | 24.04385185 | 24.15267181 | 24.15800095 | 24.14039803 | 23.92985344 |
|  | 1.525023981 | 0.231571198 | P50544;B1AR28 | Very long-chain specific acyl-CoA dehydrogenase, mitochondrial | Acadvl | 23.68645668 | 23.69795227 | 23.61380386 | 23.56150818 | 23.39479828 | 23.34719276 |
|  | 1.103535845 | 0.186603546 | Q00PI9;A0A494BAL6 | Heterogeneous nuclear ribonucleoprotein U-like protein 2 | Hnrnpul2 | 23.6848526 | 23.58897972 | 23.58083916 | 23.30159378 | 23.55086327 | 23.44240379 |
|  | 0.393449726 | 0.171132088 | Q9JKV1;A0A0A6YVU8;D3YUD8 | Proteasomal ubiquitin receptor ADRM1 | Adrm1;Gm9774 | 23.68453026 | 23.88641548 | 24.14755821 | 23.68720436 | 23.78286743 | NaN |
|  | 1.203659944 | -0.099850337 | P21279;A0A494BBL5;P30677;P21278;A0A0G2JG40;O70443;P27600 | Guanine nucleotide-binding protein G(q) subunit alpha | Gnaq | 23.68420982 | 23.77464867 | 23.73644829 | 23.87267494 | 23.84622955 | 23.77595329 |
|  | 1.436793552 | -0.189634959 | Q6PGH2 | Hematological and neurological expressed 1-like protein | Hn1l | 23.68099594 | 23.49399376 | 23.50166702 | 23.75674438 | 23.75154305 | 23.73727417 |
|  | 0.945689108 | -0.167445501 | Q9CX56;Q9CPS5;Q3TG45 | 26S proteasome non-ATPase regulatory subunit 8 | Psmd8 | 23.6806736 | 23.83113098 | 23.62934303 | 23.82045746 | 23.99412155 | 23.82890511 |
|  | 0.090099549 | 0.019215266 | Q8VEE0;B2KGF0;M0QWJ2;A0A087WQM3;M0QWQ0 | Ribulose-phosphate 3-epimerase | Rpe | 23.67874146 | 23.62243271 | 23.82725906 | 23.77996635 | 23.65283203 | 23.63798904 |
|  | 0.214815159 | 0.077355703 | Q9D024;F7C265;B1AR93 | Coiled-coil domain-containing protein 47 | Ccdc47 | 23.67691422 | 23.63942528 | 23.30924797 | 23.59809875 | 23.46413422 | 23.33128738 |
|  | 0.168181061 | 0.106215477 | Q8R550;B0R0Y8 | SH3 domain-containing kinase-binding protein 1 | Sh3kbp1 | 23.67486763 | NaN | 23.23790359 | 23.38679695 | NaN | 23.31354332 |
|  | 0.717201339 | -0.167578379 | P35821 | Tyrosine-protein phosphatase non-receptor type 1 | Ptpn1 | 23.67454529 | 23.49179459 | 23.8365345 | 23.81850815 | 23.778162 | 23.90893936 |
|  | 0.594532092 | 0.313550949 | Q9Z1F9;H3BLM2;H3BLR3;A2BH29 | SUMO-activating enzyme subunit 2 | Uba2 | 23.66871452 | 23.67033577 | 23.45487976 | 23.71920967 | 23.16538048 | 22.96868706 |
|  | 0.202560336 | -0.036739985 | Q9EPL9;Q3TAW3;E9Q296;Q8C178;D3Z2N6;A0A0J9YVH1 | Peroxisomal acyl-coenzyme A oxidase 3;Acyl-coenzyme A oxidase | Acox3 | 23.66503143 | 23.48246956 | 23.5793438 | 23.52073288 | 23.65140915 | 23.66492271 |
|  | 0.342676782 | 0.134863536 | Q3UGR5 | Haloacid dehalogenase-like hydrolase domain-containing protein 2 | Hdhd2 | 23.66470528 | 23.32388687 | 23.69540596 | 23.49996567 | 23.20794678 | 23.57149506 |
|  | 0.636589898 | -0.116611481 | P12382;P47857;A0A1W2P7T1;A0A0E2W9U1 | ATP-dependent 6-phosphofructokinase, liver type | Pfkl | 23.66459656 | 23.70756721 | 23.81421089 | 23.75979424 | 23.98328781 | 23.79312706 |
|  | 0.927714471 | -0.31531779 | O09005 | Sphingolipid delta(4)-desaturase DES1 | Degs1 | 23.66394615 | 23.52838516 | 23.73210526 | 23.93427086 | 23.71397781 | 24.22214127 |
|  | 0.876397086 | -0.05809466 | P32067;A2AR07;D6RI87;F6SXM5 | Lupus La protein homolog | Ssb | 23.66025162 | 23.60840034 | 23.56360435 | 23.67400551 | 23.64394379 | 23.688591 |
|  | 1.093022101 | -0.234871546 | Q501J6;Q3U741;Q3TU25 | Probable ATP-dependent RNA helicase DDX17 | Ddx17 | 23.6568737 | 23.84985924 | 23.90674019 | 24.08237267 | 24.12778282 | 23.90793228 |
|  | 1.012891579 | 0.166560491 | D3Z5U5;Q9Z257;Q9JJX6;D3YYR5;Q9Z256 | P2X purinoceptor;P2X purinoceptor 4 | P2rx4 | 23.65534592 | 23.75378799 | 23.77776146 | 23.4575119 | 23.68837929 | 23.54132271 |
|  | 0.522724033 | -0.135053635 | F6SUM2;O70496;E9PYL4;A0A3B2W4I8 | H(+)/Cl(-) exchange transporter 7;Chloride channel protein | Clcn7 | 23.65272331 | 23.80862617 | 23.55555153 | 23.96375847 | 23.79273033 | 23.66557312 |
|  | 0.417507321 | 0.072365443 | Q8R010;Q8R3V2 | Aminoacyl tRNA synthase complex-interacting multifunctional protein 2 | Aimp2 | 23.64746284 | 23.53943062 | 23.70155144 | 23.4702301 | 23.66275215 | 23.53836632 |
|  | 0.28777057 | -0.148801168 | Q91W90;E9PXX7;A0A0R4J1Y7 | Thioredoxin domain-containing protein 5 | Txndc5 | 23.64427376 | 24.21437836 | 23.88035583 | 24.31672287 | 23.94127464 | 23.92741394 |
|  | 0.940070928 | -0.259206772 | A0A494BB95;Q60872;Q3UTA4;Q3TQZ4;J3QP87;A0A1Y7VK80;A0A1Y7VJE9;J3QQ02;J3QPI8;A0A1Y7VLT7;Q8BX20;Q3UT53;J3QMW5;A0A1Y7VNG9;F6YNI8 | Eukaryotic translation initiation factor 1A | Eif1a;Gm8300;Gm2016;Gm2035;Gm2056;Gm6803;Gm5662 | 23.64118958 | 23.41931152 | 23.22592545 | 23.75000954 | 23.59514046 | 23.71889687 |
|  | 0.980820557 | -0.10582606 | Q9CQE5;A0A0U1RPU5;D3Z1B6 | Regulator of G-protein signaling 10 | Rgs10 | 23.63986588 | 23.74550438 | 23.58428383 | 23.75490952 | 23.7357254 | 23.79649734 |
|  | 0.088895838 | 0.022631327 | Q9WTI7 | Unconventional myosin-Ic | Myo1c | 23.63898277 | 23.65676498 | 23.66633224 | 23.64790154 | 23.77866364 | 23.46762085 |
|  | 0.90685959 | 0.335264842 | O88696 | ATP-dependent Clp protease proteolytic subunit, mitochondrial | Clpp | 23.63511276 | 23.85490799 | 23.55870819 | 23.20178413 | 23.49350548 | NaN |
|  | 0.517953764 | 0.14478302 | Q8BGS2 | BolA-like protein 2 | Bola2 | 23.63333893 | 24.00872612 | 23.81274414 | 23.78536415 | 23.59445763 | 23.64063835 |
|  | 1.870652492 | -0.156218847 | Q64674 | Spermidine synthase | Srm | 23.61582565 | 23.67562294 | 23.68773842 | 23.80252457 | 23.87258148 | 23.7727375 |
|  | 1.10543694 | 0.303284327 | Q9D883;G3UW94;A0A494BA95;A0A494B9X9;E9Q809;A0A494B947;F8WH71;E9Q5J3;E9PWX6;E9PWM2 | Splicing factor U2AF 35 kDa subunit | U2af1 | 23.61380386 | 23.99524498 | 23.62377167 | 23.38258171 | 23.48062134 | 23.45976448 |
|  | 0.431950159 | -0.057855606 | Q9DCR2;A0A286YD58;A0A286YDU3 | AP-3 complex subunit sigma-1 | Ap3s1 | 23.61313057 | 23.66633224 | 23.63765717 | NaN | 23.62811852 | 23.76567268 |
|  | 0.061874375 | 0.006795565 | O35942;F6TQF4;F6THK7;B1AUW8;B1AUW9;Q8BR10;B1AUW6;Q62406 | Serine/threonine-protein kinase Nek2;Interleukin-1 receptor-associated kinase 1 | Nek2;Irak1 | 23.60489845 | NaN | 23.51833344 | 23.58152962 | 23.55156898 | 23.53136253 |
|  | 0.605213108 | 0.129135768 | Q9CX34 | Suppressor of G2 allele of SKP1 homolog | Sugt1 | 23.60455894 | 23.72473526 | 23.89753723 | 23.69954109 | 23.5817585 | 23.55812454 |
|  | 0.772450212 | 0.183090846 | O88587;D3Z227 | Catechol O-methyltransferase | Comt | 23.60455894 | 23.55238914 | 23.63898277 | 23.37755966 | 23.25349617 | 23.61560249 |
|  | 1.100612503 | -0.149179777 | A0A0G2JGX4;Q8VCE0;Q6PIC6;Q9Z1W8 | Sodium/potassium-transporting ATPase subunit alpha-3 | Atp1a3 | 23.60229492 | 23.49728775 | 23.49497032 | 23.72650337 | NaN | 23.63489151 |
|  | 1.136677954 | 0.168927511 | Q9Z0Y1;E9Q919 | Dynactin subunit 3 | Dctn3 | 23.58451462 | 23.46088982 | 23.48750687 | 23.41944122 | 23.22635078 | 23.38033676 |
|  | 1.333253699 | 0.147896449 | Q8BYC6;Q3V3K3;A0A0R4J1T3;F6RXB5;Q6ZQ29 | Serine/threonine-protein kinase TAO3 | Taok3 | 23.5825634 | 23.58520126 | 23.7091465 | 23.53528404 | 23.46811867 | 23.42981911 |
|  | 0.48170319 | 0.048931122 | Q7TSV4;A0A0G2JG04;A0A0G2JF47 | Phosphoglucomutase-2 | Pgm2 | 23.57796097 | 23.62800789 | 23.49960136 | 23.55438232 | 23.47481728 | 23.52957726 |
|  | 0.095060928 | -0.04016908 | Q8BU14 | Translocation protein SEC62 | Sec62 | 23.57346153 | 23.66307831 | 23.71858215 | 23.50760651 | 23.87614632 | NaN |
|  | 1.100465 | 0.126968384 | O70172;F6RJE8;A2ASS3 | Phosphatidylinositol 5-phosphate 4-kinase type-2 alpha | Pip4k2a | 23.57045364 | 23.55835724 | 23.66264343 | 23.39636803 | 23.46849251 | 23.54568863 |
|  | 0.524437399 | -0.104879379 | Q6A028 | Switch-associated protein 70 | Swap70 | 23.56465149 | 23.66654968 | 23.67734337 | 23.90140915 | 23.66795731 | 23.65381622 |
|  | 0.407834622 | -0.089541753 | B1AV77;B1ATI0;P47740;A0A140LJF9;P47739 | Aldehyde dehydrogenase;Fatty aldehyde dehydrogenase | Aldh3a2 | 23.56045914 | 23.78915215 | 23.73169136 | 23.72244453 | 23.90921402 | 23.71826935 |
|  | 0.394596262 | 0.051153819 | Q8BMJ2;A0A494BA98;A0A494BBF9;A0A494B919;A0A494BA30 | Leucine--tRNA ligase, cytoplasmic | Lars | 23.5460434 | 23.42675209 | 23.47444534 | 23.49497032 | 23.35097313 | 23.44783592 |
|  | 0.318173036 | 0.045469284 | Q06138;Q9DB16 | Calcium-binding protein 39 | Cab39 | 23.54297638 | 23.35366631 | 23.4708519 | 23.37345123 | 23.44012451 | 23.41751099 |
|  | 0.250161047 | 0.072301229 | P36423;F6SG79;Q3TRY5 | Thromboxane-A synthase | Tbxas1 | 23.5421505 | 23.40262604 | 23.48628044 | 23.19635963 | 23.46575546 | 23.55203819 |
|  | 0.041990294 | -0.015036901 | Q8K2Q0 | COMM domain-containing protein 9 | Commd9 | 23.54085159 | 23.4777832 | 23.67022896 | 23.36919594 | 23.63422585 | 23.73055267 |
|  | 0.022153442 | -0.005971273 | Q921M3 | Splicing factor 3B subunit 3 | Sf3b3 | 23.53753662 | 23.4342804 | 23.69635963 | 23.58037949 | 23.47159576 | 23.63411522 |
|  | 0.96313934 | 0.157304128 | Q9QYA2;G3UY77 | Mitochondrial import receptor subunit TOM40 homolog | Tomm40 | 23.53528404 | 23.58221817 | 23.71450043 | 23.54863167 | 23.45161438 | 23.35984421 |
|  | 0.006455683 | 0.003539721 | Q8BVQ0;Q9ERF3;D6RDC7;D6RJ72 | WD repeat-containing protein 61;WD repeat-containing protein 61, N-terminally processed | Wdr61 | 23.53290939 | 23.1960144 | 23.23660469 | 23.46712303 | 23.16948318 | NaN |
|  | 1.2121621 | 0.299531301 | G3X9U9;Q9CQ92 | Mitochondrial fission 1 protein | Fis1 | 23.53195763 | 23.32567024 | 23.52324867 | 23.23350716 | 23.0880146 | NaN |
|  | 0.684755117 | -0.138843536 | Q9CQR6;A0A0N4SVL9;A0A0N4SVE2;A0A0N4SW66 | Serine/threonine-protein phosphatase 6 catalytic subunit;Serine/threonine-protein phosphatase 6 catalytic subunit, N-terminally processed | Ppp6c | 23.52933884 | 23.48148346 | 23.37729454 | 23.44720459 | 23.63865089 | 23.71879196 |
|  | 0.84006953 | 0.364411036 | F8VQ28;A0A1D5RMM8;Q8VI36;A0A0J9YV30 | Paxillin | Pxn | 23.52838516 | 23.39060593 | 23.67841911 | 23.38073349 | 22.80299759 | 23.32044601 |
|  | 1.657247007 | 0.169205983 | E0CZ22;D3Z624;E0CYC5 | Maestro heat-like repeat family member 1 | Mroh1 | 23.52611732 | 23.43338966 | 23.38099861 | 23.24907494 | 23.26999283 | 23.31381989 |
|  | 0.227956115 | -0.057047526 | A0A1D5RLS2;A0A1D5RM23;Q9CQF3;A0A1D5RLT7 | Cleavage and polyadenylation specificity factor subunit 5 | Nudt21 | 23.52563858 | 23.40781975 | 23.5378933 | 23.3997612 | 23.70661926 | 23.53611374 |
|  | 0.234453175 | -0.044647217 | Q3UE92;Q6P1B1;S4R1I3;A0A494BBG8;S4R228 | Xaa-Pro aminopeptidase 1 | Xpnpep1 | 23.52432442 | 23.71690941 | 23.626894 | 23.56964302 | 23.73489952 | 23.69752693 |
|  | 0.108578565 | -0.027869542 | G5E829;A0A1W2P7C7;F7AAP4;E9Q828;Q3UHH0;Q6Q477;F8WHB1;Q9R0K7;A0A1W2P867;A0A1W2P7R3 | Plasma membrane calcium-transporting ATPase 1 | Atp2b1 | 23.52432442 | 23.66459656 | 23.79798126 | 23.78735924 | 23.64196205 | 23.64118958 |
|  | 1.388465128 | -0.211149851 | A0A1W2P6X3;Q9JL26;A2AB60;G3UWI1 | Formin-like protein 1 | Fmnl1 | 23.52324867 | 23.58691978 | 23.54155922 | 23.64361382 | 23.76111412 | 23.8804493 |
|  | 2.469923387 | 0.167685191 | Q8C2E7 | WASH complex subunit strumpellin | Kiaa0196 | 23.51749229 | 23.44758415 | 23.5148468 | 23.32827568 | 23.29977798 | 23.34881401 |
|  | 0.795557247 | -0.187606812 | Q8BTZ7 | Mannose-1-phosphate guanyltransferase beta | Gmppb | 23.50930023 | 23.36652946 | 23.68271065 | 23.73138046 | 23.79540825 | 23.59457207 |
|  | 0.684703253 | -0.186368942 | Q810B6 | Rabankyrin-5 | Ankfy1 | 23.50809097 | 23.44985199 | 23.60602951 | 23.49069405 | 23.88278389 | 23.74960136 |
|  | 1.429465798 | -0.167075475 | E9Q2A6;Q9QVP9;Q3UDE9;F7CCX1 | Protein-tyrosine kinase 2-beta | Ptk2b | 23.50324631 | 23.44126511 | 23.57968903 | 23.63577652 | 23.64097023 | 23.74868011 |
|  | 0.038713565 | 0.005673091 | Q68FL6;E9QB02;F6W0G8 | Methionine--tRNA ligase, cytoplasmic | Mars | 23.49558067 | 23.55461693 | 23.46824265 | 23.44101143 | 23.47704315 | 23.58336639 |
|  | 0.613457004 | 0.10796229 | P27601;Q9D034 | Guanine nucleotide-binding protein subunit alpha-13 | Gna13 | 23.4942379 | 23.33824158 | 23.44733238 | 23.19171524 | 23.39741325 | 23.36679649 |
|  | 0.424675332 | 0.072746277 | Q91VR5;A0A1Y7VM48 | ATP-dependent RNA helicase DDX1 | Ddx1 | 23.49277306 | 23.55777359 | 23.66351128 | 23.6025219 | 23.42393494 | 23.46936226 |
|  | 0.967294198 | -0.189381282 | Q9Z1A1;F6QJV5;B8JJG7;B8JJG8;B8JJG9 | Trk-fused gene | Tfg | 23.49167252 | 23.36385918 | 23.61133003 | 23.56674194 | 23.71019745 | 23.75806618 |
|  | 0.114777515 | 0.031520844 | Q8K310;A0A494BAZ2;A0A494BAZ7;A0A494BAD4;A0A494B9X1;A0A494BAL7;A0A494B9B1;A0A494BAC5;A0A494BB50;A0A494B968;A0A494B9C9;A0A494B8Y5;A0A494B9Q4 | Matrin-3 | Matr3 | 23.49020386 | 23.70482635 | 23.59149361 | 23.59274864 | 23.41660881 | 23.68260384 |
|  | 0.146081446 | 0.037029266 | Q9QWR8;A0A2R8VHH9;Q3UZX5;A0A2R8VHJ3;A0A2R8W742 | Alpha-N-acetylgalactosaminidase | Naga | 23.48971367 | 23.39270401 | 23.68035126 | 23.49923515 | 23.40522575 | 23.54722023 |
|  | 0.025831729 | -0.005467097 | O54988;A0A5F8MPV2 | STE20-like serine/threonine-protein kinase | Slk | 23.47815514 | 23.34975815 | 23.57519341 | 23.52623749 | 23.46039009 | 23.4328804 |
|  | 0.268819577 | 0.053478241 | Q9EQH2;A0A1Y7VMT6;A0A1Y7VNY4 | Endoplasmic reticulum aminopeptidase 1 | Erap1 | 23.47617722 | 23.53112411 | 23.5793438 | 23.50409508 | 23.33565331 | 23.58646202 |
|  | 1.131035357 | -0.225992203 | Q62419;A0A3B2W7K0;A2ALV1;Q8BXU5;A2ALV3;Q62420 | Endophilin-A2 | Sh3gl1 | 23.47419739 | 23.49996567 | 23.6028614 | 23.66351128 | 23.9223423 | 23.66914749 |
|  | 0.084375817 | 0.02137057 | Q921J2 | GTP-binding protein Rheb | Rheb | 23.47221565 | 23.64427376 | 23.49289513 | 23.59889221 | 23.57346153 | 23.37291908 |
|  | 1.144065734 | 0.283365885 | Q9DB27;Q9CQ21 | Malignant T-cell-amplified sequence 1 | Mcts1 | 23.47221565 | 23.20197868 | 23.19207573 | 23.04119682 | 22.86717987 | 23.10779572 |
|  | 0.514640169 | -0.20202891 | Q3MIA8;G3UXW9;Q99LD4;A0A140LJB7;B1ATU4 | COP9 signalosome complex subunit 1 | Gps1 | 23.47184372 | 23.10173416 | 22.88966942 | 23.32950783 | 23.33019257 | 23.40963364 |
|  | 0.302909007 | -0.041733424 | Q3UKN6;P81117 | Nucleobindin-2;Nesfatin-1 | Nucb2 | 23.46849251 | 23.527668 | 23.36959457 | 23.4655056 | 23.56069183 | 23.46475792 |
|  | 0.11420495 | -0.024723689 | Q9QZ06;Q8C5G6;A9JEI5;F7AT44 | Toll-interacting protein | Tollip | 23.46039009 | 23.53694344 | 23.57010651 | 23.58520126 | 23.64768219 | 23.40872765 |
|  | 0.607516236 | -0.247959137 | P11680 | Properdin | Cfp | 23.45788765 | 23.91624832 | 23.70461464 | 23.90701485 | 23.97473717 | NaN |
|  | 0.166191369 | -0.046541214 | A0A0U1RNT6;A0A0U1RNK6;Q3THS6;A0A0U1RQB0;A0A0U1RQ95 | S-adenosylmethionine synthase isoform type-2 | Mat2a | 23.45726204 | 23.49081612 | 23.41454506 | 23.48173141 | 23.68827248 | 23.33224297 |
|  | 0.193701671 | -0.016429265 | Q91VC9;A0A286YDK2 | Growth hormone-inducible transmembrane protein | Ghitm | 23.45600891 | 23.42534447 | 23.42085457 | 23.5114727 | 23.41531944 | 23.4247036 |
|  | 0.023888831 | 0.007303238 | Q91XH6;O88384;F6UHS3;E0CYE5 | Vesicle transport through interaction with t-SNAREs homolog 1B | Vti1b | 23.45261955 | 23.29529953 | 23.63677216 | 23.4616394 | 23.40470695 | 23.49643517 |
|  | 0.155751465 | -0.02447319 | D3YZ62;D3Z4J3;Q99104;F6TDE5;B8JK05;F6Z2S4;A0A1L1SUF0;D3Z135;G5E8G6;G3X9Y9;P21271 | Unconventional myosin-Va | Myo5a | 23.45199013 | 23.50348854 | 23.50397301 | 23.42483139 | 23.61661148 | 23.49142838 |
|  | 0.991325552 | 0.281679153 | Q9WTP7;F6RP11 | GTP:AMP phosphotransferase AK3, mitochondrial | Ak3 | 23.4461956 | 23.37967682 | 23.54462814 | 22.96961784 | 23.39910889 | 23.15673637 |
|  | 0.059470401 | 0.016227086 | F6UND7;P08103 | Non-specific protein-tyrosine kinase;Tyrosine-protein kinase HCK | Hck | 23.44265747 | 23.18343925 | 23.41544914 | 23.36546326 | 23.23876381 | 23.38863754 |
|  | 0.56109147 | -0.231578827 | Q9JHU9 | Inositol-3-phosphate synthase 1 | Isyna1 | 23.44177055 | 23.33128738 | 23.35944366 | 23.25580406 | 23.72463226 | 23.84680176 |
|  | 1.174283969 | 0.114245733 | O08804;A0A1Y7VLD5;F7B9A0;I7HJI3 |  | Serpinb6b | 23.44088364 | 23.36372566 | 23.40885735 | 23.31326675 | 23.21257019 | 23.3448925 |
|  | 0.134765149 | 0.040318171 | Q8VE70;F8WI55;E0CY82 | Programmed cell death protein 10 | Pdcd10 | 23.43745995 | 23.52025414 | 23.69402313 | 23.59605217 | 23.58497238 | 23.34975815 |
|  | 0.428465832 | -0.120243073 | A0A0R4J034;Q99K01;D3YZA7 | Pyridoxal-dependent decarboxylase domain-containing protein 1 | Pdxdc1 | 23.43606186 | 23.42815781 | 23.46475792 | 23.37782478 | 23.78636169 | 23.52552032 |
|  | 0.347940765 | 0.289471944 | Q6P9R2 | Serine/threonine-protein kinase OSR1 | Oxsr1 | 23.43402672 | 23.47642517 | 22.96225739 | 23.28404427 | 23.32484818 | 22.395401 |
|  | 0.117729989 | 0.035421371 | P97379;A0A0J9YUY8;A0A0J9YTQ8 | Ras GTPase-activating protein-binding protein 2 | G3bp2 | 23.43275261 | 23.67314339 | 23.64394379 | 23.63931465 | 23.39034462 | 23.6139164 |
|  | 0.373296476 | 0.063238144 | Q9D8T2;A0A2R8VKQ7 | Gasdermin-D | Gsdmdc1 | 23.43198776 | 23.30270958 | 23.38679695 | 23.19983864 | 23.40613365 | 23.32580757 |
|  | 0.676632042 | 0.170249303 | Q8BYW1 | Rho GTPase-activating protein 25 | Arhgap25 | 23.43135071 | 23.42969131 | 23.27511787 | 23.01012802 | 23.34800339 | 23.26728058 |
|  | 1.279699354 | 0.134199778 | Q9WTX6;Q3TPM3;A0A0N4SUW1;D3Z2H3 | Cullin-1 | Cul1 | 23.43033028 | 23.53990555 | 23.38929367 | 23.35299301 | 23.28390312 | 23.32003403 |
|  | 1.118145066 | -0.187477112 | P01901;A0A494BA33;Q3TH01;A0A494BAT0;G3UXW2;P04223;P14428;P03991;P14426;A0A0B4J1G3;A0A494B9G8;A0A494B9G2 | H-2 class I histocompatibility antigen, K-B alpha chain;H-2 class I histocompatibility antigen, K-K alpha chain;H-2 class I histocompatibility antigen, K-Q alpha chain;H-2 class I histocompatibility antigen, K-W28 alpha chain | H2-K1 | 23.42585564 | 23.60195541 | 23.55484962 | 23.74180603 | 23.80064964 | 23.60263634 |
|  | 1.153449726 | 0.172972361 | A0A0A0MQN4;Q9D906;S4R2D5;S4R2T5;S4R2P7;S4R1T0;S4R1N8;S4R1U3;S4R1W9;S4R1K1 | Ubiquitin-like modifier-activating enzyme ATG7 | Atg7 | 23.42354965 | 23.33429146 | 23.43033028 | 23.28432655 | 23.0963707 | 23.28855705 |
|  | 0.924699023 | 0.201145808 | Q8JZQ2;Q920A7 | AFG3-like protein 2 | Afg3l2 | 23.42123985 | 23.26284409 | 23.3303299 | 22.99415588 | 23.30521584 | 23.11160469 |
|  | 1.390428723 | 0.197057724 | Q8VE47;F6VRI6;D6RH76;A0A1L1SSH6 | Ubiquitin-like modifier-activating enzyme 5 | Uba5 | 23.42111206 | 23.42815781 | 23.24972725 | 23.17634964 | 23.11224174 | 23.21923256 |
|  | 0.427309179 | -0.147251129 | Q9CQB4;Q9D855 | Cytochrome b-c1 complex subunit 7 | Uqcrb | 23.42034149 | 23.18157578 | 23.5853157 | 23.48283768 | 23.71837425 | 23.42777443 |
|  | 0.373591474 | -0.06183815 | P17427;A0A0J9YUA7 | AP-2 complex subunit alpha-2 | Ap2a2 | 23.41712379 | 23.31409836 | 23.51496696 | 23.53919411 | 23.40794945 | 23.48456001 |
|  | 0.663038322 | 0.13692983 | Q9CX86 | Heterogeneous nuclear ribonucleoprotein A0 | Hnrnpa0 | 23.41531944 | 23.27980042 | 23.3372879 | 23.20137978 | 23.35716248 | 23.06307602 |
|  | 0.869982844 | 0.168240865 | Q8K411 | Presequence protease, mitochondrial | Pitrm1 | 23.41519165 | 23.27326965 | 23.38324165 | 23.10126877 | 23.11892509 | 23.3467865 |
|  | 0.372638797 | 0.106521606 | D3Z7P0;Q8K3W0;E9Q0U3;A0A0J9YV56;E9Q8Q1 | BRCA1-A complex subunit BRE | Bre | 23.40794945 | 23.0722332 | 23.42316437 | 23.26327324 | 23.17197418 | 23.14853477 |
|  | 0.605190398 | 0.143477758 | Q8BH24 | Transmembrane 9 superfamily member 4 | Tm9sf4 | 23.40743256 | 23.74160004 | 23.524683 | 23.37848663 | 23.49679947 | 23.36799622 |
|  | 0.657495876 | -0.079580307 | A2AL85;Q8BSY0;Q8CBM2;A2AL83;A2AL79;A2AL78;Q3TU40;A2AL81;A2AL71;A2AL77;Q9CR06;A2AL74;A2AL76;A2AL75 | Aspartyl/asparaginyl beta-hydroxylase | Asph | 23.40730286 | 23.30396271 | 23.29263496 | 23.33374405 | 23.44189835 | 23.46699905 |
|  | 0.838607104 | 0.216178258 | A0A0R4J2B0;V9GX43;S4R2U7;S4R1S7;E9QA47;Q9Z0H4;S4R2L5;S4R2J2;A3KGT0;S4R2U1 | CUGBP Elav-like family member 2 | Celf2 | 23.40145493 | 23.2358017 | 23.54380226 | 23.07162666 | 23.12674332 | 23.33415413 |
|  | 0.329567871 | -0.020325979 | P63094;Q6R0H7;A0A571BEI3;Z4YKV1;A0A571BE25;A0A571BEM2;A0A571BEG2;A2A610;Q66L47;Q8CGK7;A0A571BGH5 | Guanine nucleotide-binding protein G(s) subunit alpha isoforms short;Guanine nucleotide-binding protein G(s) subunit alpha isoforms XLas | Gnas | 23.40080261 | 23.35353279 | 23.39466858 | 23.44404793 | 23.37755966 | 23.38837433 |
|  | 0.10359429 | -0.028938293 | Q3TCW6;P34960;Q8BJC0;D3Z705 | Macrophage metalloelastase | Mmp12 | 23.39126205 | 23.22751236 | 23.05212021 | 23.28700829 | 23.26126671 | 23.20943451 |
|  | 0.197094394 | 0.085854212 | Q8BJU0;A0A1W2P6P1;A0A1W2P7I5 | Small glutamine-rich tetratricopeptide repeat-containing protein alpha | Sgta | 23.38784981 | 23.47580719 | 23.26913643 | 23.32251167 | 23.54592514 | 23.00679398 |
|  | 1.012806827 | -0.202007929 | Q921Y0;Q3UDM0;Q8BPB0 | MOB kinase activator 1A;MOB kinase activator 1B | Mob1a;Mob1b | 23.38455963 | 23.37769318 | 23.52528 | 23.77464867 | 23.62198639 | 23.49692154 |
|  | 0.253038786 | -0.0356191 | H9H9R4;Q9JHJ3;H3BK59;H3BKY1 | Glycosylated lysosomal membrane protein | Glmp | 23.38192177 | 23.56616211 | 23.43771362 | 23.47790718 | 23.49521446 | 23.51953316 |
|  | 1.083400444 | 0.314200719 | P48758;A0A338P684 | Carbonyl reductase [NADPH] 1 | Cbr1 | 23.38152695 | 23.33388138 | 23.09434319 | 23.14890862 | 22.92304993 | 22.79519081 |
|  | 1.560155602 | 0.196694056 | Q8VDP3;E9PUI4;D3Z5P6 | Protein-methionine sulfoxide oxidase MICAL1 | Mical1 | 23.37517548 | 23.40002251 | 23.49460411 | 23.286726 | 23.25493813 | 23.1380558 |
|  | 0.693685887 | 0.138023376 | Q8R180;A0A2I3BPM1;A0A1Y7VJM4;A0A1Y7VNF4;Q8R2E9 | ERO1-like protein alpha | Ero1l | 23.37371635 | 23.29879951 | 23.29824066 | 23.02587128 | 23.20514488 | 23.32567024 |
|  | 0.184679303 | 0.045503616 | Q91Z50;P39749 | Flap endonuclease 1 | Fen1 | 23.36999512 | 23.3198967 | 23.55367851 | 23.30577278 | 23.30924797 | 23.49203873 |
|  | 0.044065716 | 0.021274567 | Q3UDS7;A0A1L1SSF2;Q8VDL4 | ADP-dependent glucokinase | Adpgk | 23.36932945 | 23.05763245 | 22.8854847 | 23.0824852 | 23.22909737 | 22.93704033 |
|  | 1.172352739 | 0.184165319 | Q7TQK5;E9QAD4 | Coiled-coil domain-containing protein 93 | Ccdc93 | 23.36932945 | 23.46188927 | 23.57172585 | 23.33388138 | 23.19350624 | 23.32306099 |
|  | 0.489096559 | -0.075431188 | P28271 | Cytoplasmic aconitate hydratase | Aco1 | 23.36666298 | 23.22271538 | 23.21483803 | 23.35407066 | 23.41660881 | 23.25983047 |
|  | 0.995140897 | -0.271086375 | P25911;P16277 | Tyrosine-protein kinase Lyn | Lyn | 23.36064911 | 23.69667816 | 23.67658997 | 23.71533966 | 23.91031265 | 23.92152405 |
|  | 1.380704577 | 0.231536229 | Q8CG76 | Aflatoxin B1 aldehyde reductase member 2 | Akr7a2 | 23.36024666 | 23.22430611 | 23.12899399 | 22.92589569 | 23.04224968 | 23.05079269 |
|  | 0.484058967 | -0.081330617 | P97300;H3BIX4;Z4YLB7;A0A0A0MQN8;H3BKA7 | Neuroplastin | Nptn | 23.35608673 | 23.31077385 | 23.41222191 | 23.37252045 | 23.57484818 | 23.37570572 |
|  | 0.014123713 | -0.00349172 | O35704 | Serine palmitoyltransferase 1 | Sptlc1 | 23.35595322 | 23.10615158 | 23.16249466 | 23.26899338 | 23.16183281 | 23.20424843 |
|  | 0.343284405 | 0.088502248 | Q9CRB9;F6QFL0;S4R238;Q9D9P1;D3Z0L4 | MICOS complex subunit Mic19 | Chchd3 | 23.34800339 | 23.53267097 | 23.56767082 | 23.34773254 | 23.28107452 | 23.55403137 |
|  | 0.177582227 | -0.013065974 | Q80WQ2;A0A1D5RLY2;A0A1D5RLN5 | Protein VAC14 homolog | Vac14 | 23.33864975 | 23.26828003 | 23.24688911 | 23.30382347 | 23.29165268 | 23.29754066 |
|  | 0.84340463 | 0.340741475 | Q8CHK3 | Lysophospholipid acyltransferase 7 | Mboat7 | 23.33756065 | 23.18709946 | 23.07957458 | 23.0756321 | 22.52063751 | 22.98574066 |
|  | 0.054698455 | 0.016390483 | Q9DAU1 | Protein canopy homolog 3 | Cnpy3 | 23.32676888 | 23.21571159 | 23.12533951 | 23.03667641 | 23.30827522 | 23.2736969 |
|  | 0.747321581 | 0.302722931 | A2ATI9;Q99JX3;A2ATI8;A2ATI6 | Golgi reassembly-stacking protein 2 | Gorasp2 | 23.32539749 | 23.43707848 | 23.70882988 | 23.00051498 | 23.08483887 | 23.4777832 |
|  | 0.277769582 | -0.087848981 | P54823 | Probable ATP-dependent RNA helicase DDX6 | Ddx6 | 23.32457352 | 23.22698212 | 23.34095955 | 23.53883934 | NaN | 23.23186874 |
|  | 0.400849758 | 0.191113154 | Q8BUU7;Q68FH4;B7ZCT5;B7ZCT4;B7ZCT3 | N-acetylgalactosamine kinase | Galk2 | 23.3212738 | 23.80970573 | 23.54285812 | 23.27753258 | 23.45613289 | NaN |
|  | 0.036083002 | -0.016250928 | D3Z6A4;D3Z1G3;Q8K5B2 | Multiple coagulation factor deficiency protein 2 homolog | Mcfd2 | 23.31630898 | 23.36919594 | 23.6848526 | NaN | 23.45085907 | 23.49521446 |
|  | 0.044718846 | -0.011035283 | Q3UJB0;A0A494B9S9 | Splicing factor 3b, subunit 2 | Sf3b2 | 23.31575584 | 23.16666794 | 23.40976334 | 23.35474396 | 23.35366631 | 23.21688271 |
|  | 0.608959758 | -0.329238256 | Q3V4D5;B1AUY9;B1AUZ1;B1AUY8;B1AUY7;Q3UX61;Q9QY36 | N-alpha-acetyltransferase 11;N-alpha-acetyltransferase 10 | Naa10;Naa11 | 23.31437492 | 23.0569725 | 22.57198143 | 23.12185097 | 23.317276 | 23.49191666 |
|  | 0.156884944 | 0.092719396 | Q8C0E2 | Vacuolar protein sorting-associated protein 26B | Vps26b | 23.31395912 | 23.15296555 | 23.09269905 | 22.80817413 | 22.96701622 | 23.50627518 |
|  | 0.132991936 | 0.045657476 | P67871;G3UXG7;G3UZJ5;G3UZA4;G3UXU2;G3UZX4;G3UWU5 | Casein kinase II subunit beta | Csnk2b | 23.31146622 | 23.38824272 | 23.11009216 | 23.12856865 | 23.41506195 | 23.12919807 |
|  | 0.150758181 | -0.030286153 | Q9CQE8 | UPF0568 protein C14orf166 homolog |  | 23.29614067 | 23.42624092 | 23.35420609 | 23.30396271 | 23.51629066 | 23.34719276 |
|  | 0.362185467 | 0.125044505 | Q9CR16 | Peptidyl-prolyl cis-trans isomerase D | Ppid | 23.28996468 | 23.24000168 | 23.42687988 | 23.12488365 | 23.00634766 | 23.45048141 |
|  | 0.181117726 | -0.056229909 | Q9JIA7;A0A1B0GS43 | Sphingosine kinase 2 | Sphk2 | 23.28911972 | 23.3755722 | 23.18295479 | 23.38376808 | 23.49252892 | 23.14003944 |
|  | 0.423729974 | 0.083091736 | Q8VCF0 | Mitochondrial antiviral-signaling protein | Mavs | 23.28898048 | 23.10913467 | 23.33319855 | 23.16969872 | 23.07332802 | 23.23901176 |
|  | 0.483025213 | 0.154457092 | E9Q0G1;Q9CQJ6 | Density-regulated protein | Denr | 23.28517342 | 23.31920624 | 22.93052292 | 23.11906624 | 23.04583549 | 22.90662956 |
|  | 0.620944215 | -0.136352539 | Q7TQE2;Q62523;A0A0N4SVD2;A0A0N4SUX7;A0A0N4SVP4 | Zyxin | Zyx | 23.28432655 | 23.23497009 | 23.46213913 | 23.37012672 | 23.41841125 | 23.60195541 |
|  | 1.71818246 | -0.199439367 | B1ARU4;Q9QXZ0;A0A571BGC6;A0A571BF93;A0A0A0MQA6;F7ACR9;F6Q750;F6SHS0;F6YKN8;A0A5F8MP99;F6RCJ3;F6RL59;A0A0A0MQH5;S4R2C6;S4R2A8;S4R1Y6 | Microtubule-actin cross-linking factor 1 | Macf1 | 23.28347969 | 23.23101807 | 23.23044586 | 23.50603294 | 23.34935379 | 23.48787498 |
|  | 0.54028916 | 0.208299637 | Q8BU33;A0A1W2P8E1;A0A1W2P727;A0A1W2P8D6;A0A1W2P6L8 | Acetolactate synthase-like protein | Ilvbl | 23.27810097 | 23.06849861 | 22.998106 | 22.65189171 | 22.90337944 | 23.16453552 |
|  | 0.605938246 | -0.077250799 | Q80UE5;O70318;Q80UE4;Q8C928;A0A1W2P6I5;A0A1W2P7I2;A0A1W2P7I4;A0A1W2P896;A0A1W2P7H7;A0A1W2P6H2;A0A1W2P8C0 | Band 4.1-like protein 2 | Epb4.1l2;Epb41l2 | 23.27724838 | 23.23410606 | 23.15019417 | 23.30827522 | 23.21809578 | 23.36693001 |
|  | 0.457174754 | -0.047299067 | Q64442 | Sorbitol dehydrogenase | Sord | 23.27696419 | 23.29963875 | 23.32196045 | 23.42956352 | 23.28714943 | 23.32374763 |
|  | 0.503320294 | 0.148338318 | Q9R1T2 | SUMO-activating enzyme subunit 1;SUMO-activating enzyme subunit 1, N-terminally processed | Sae1 | 23.27099037 | 23.66090393 | 23.33946419 | 23.29179192 | 23.18803596 | 23.34651566 |
|  | 0.009714562 | 0.004163106 | O35465 | Peptidyl-prolyl cis-trans isomerase FKBP8 | Fkbp8 | 23.27070618 | 23.69858742 | 23.31644821 | 23.49655724 | 23.36292458 | 23.41377068 |
|  | 0.909331534 | 0.154906591 | P27612;F7D1R5 | Phospholipase A-2-activating protein | Plaa | 23.26927948 | 23.21583176 | 23.30396271 | 22.95937729 | 23.20257568 | 23.1624012 |
|  | 0.300556567 | -0.082353592 | D3Z3W9;F8VQ94;P97484;E9Q6M9;A0A0B4J1F3;A0A0U1RP96;D3YYZ3;D3Z674;A0A087WSB3;D3Z678;F6PZL4 | Leukocyte immunoglobulin-like receptor subfamily B member 3 | Pira2;Lilrb3;Lilra6 | 23.26756668 | 23.24395752 | 23.40392685 | 23.33060265 | 23.58118439 | 23.25072479 |
|  | 0.950860516 | -0.178045909 | P12815 | Programmed cell death protein 6 | Pdcd6 | 23.26642227 | 23.29670143 | 23.38534927 | 23.44278336 | 23.38863754 | 23.6511898 |
|  | 0.225523728 | -0.03397433 | P42227;B7ZC18 | Signal transducer and activator of transcription 3;Signal transducer and activator of transcription | Stat3 | 23.26599312 | 23.40756226 | 23.35380173 | 23.38099861 | 23.30145454 | 23.44682693 |
|  | 1.881296677 | 0.173526128 | A0A286YCV9;E9Q4K7;A0A286YD28;A2AKH9 | Kinesin-like protein | Kif13b | 23.26356125 | 23.37358284 | 23.26456261 | 23.15279579 | 23.09176254 | 23.13656998 |
|  | 0.972617403 | 0.163448334 | Q9ERE7;D3YVR4;F6SWV4 | LDLR chaperone MESD | Mesdc2 | 23.26169586 | 23.14108467 | 23.32580757 | 23.05895424 | 23.0998745 | NaN |
|  | 0.039950757 | -0.023326238 | Q9DB25;D6RCG2 | Dolichyl-phosphate beta-glucosyltransferase | Alg5 | 23.26140976 | 23.55754089 | 23.25287628 | 23.25326538 | 23.71983719 | 23.16870308 |
|  | 1.373786574 | 0.176315943 | Q9ES28;A0A0R4J0X8;D3Z0V2;A0A1B0GRS3;A0A1B0GSX4 | Rho guanine nucleotide exchange factor 7 | Arhgef7 | 23.25954247 | 23.36238861 | 23.36332512 | 23.09179497 | 23.24923515 | 23.11527824 |
|  | 1.17509049 | -0.183523814 | Q8K2C9 | Very-long-chain (3R)-3-hydroxyacyl-CoA dehydratase 3 | Hacd3 | 23.25681114 | 23.40236664 | 23.30075645 | 23.45813751 | NaN | 23.54886627 |
|  | 1.481257485 | 0.195565542 | Q9D1E6 | Tubulin-folding cofactor B | Tbcb | 23.25508308 | 23.2606926 | 23.36652946 | 23.1929493 | 23.07589149 | 23.02676773 |
|  | 0.314535048 | 0.158561071 | Q3ULJ0;D3Z0L6;E0CXN5;P13707 | Glycerol-3-phosphate dehydrogenase 1-like protein | Gpd1l | 23.25421715 | 23.36972809 | 23.66947174 | 23.55238914 | 22.98158264 | 23.28376198 |
|  | 0.300839963 | -0.062783559 | O35344 | Importin subunit alpha-4 | Kpna3 | 23.25271797 | 22.97882843 | 23.16378403 | 23.23280525 | 23.14447975 | 23.2063961 |
|  | 0.121780027 | 0.085574468 | Q8BGH2 | Sorting and assembly machinery component 50 homolog | Samm50 | 23.25216866 | 23.23854446 | 23.3089695 | 23.00947762 | 23.68388939 | 22.84959221 |
|  | 0.034051651 | 0.021197637 | Q9WVQ5 | Methylthioribulose-1-phosphate dehydratase | Apip | 23.24914742 | 22.76405334 | 22.81487656 | 22.6513443 | 22.97317886 | 23.13996124 |
|  | 0.35723642 | -0.101102193 | Q9CR00;A0A0G2JGN6 | 26S proteasome non-ATPase regulatory subunit 9 | Psmd9 | 23.24796104 | 23.41002274 | 23.186903 | 23.49521446 | 23.18909264 | 23.46388626 |
|  | 1.343962207 | -0.107063293 | Q9Z2I8;A0A0N4SWD1 | Succinyl-CoA ligase [GDP-forming] subunit beta, mitochondrial | Suclg2 | 23.24729347 | 23.21998596 | 23.23420906 | 23.30730247 | 23.41364288 | 23.30173302 |
|  | 0.351537712 | 0.077298482 | Q91ZE0 | Trimethyllysine dioxygenase, mitochondrial | Tmlhe | 23.24682999 | 23.44569016 | 23.36799622 | 23.30047607 | 23.1434536 | 23.38469124 |
|  | 0.541317656 | -0.125555038 | A0A0R4J0D3;Q3TDQ1 | Dolichyl-diphosphooligosaccharide--protein glycosyltransferase subunit STT3B | Stt3b | 23.2467289 | 23.03074646 | 23.13092613 | 23.22489548 | 23.41712379 | 23.14304733 |
|  | 1.011767283 | 0.309879939 | Q8BMA6;A2AAN2 | Signal recognition particle subunit SRP68 | Srp68 | 23.24533653 | 23.58886528 | 23.20645714 | 23.04380226 | 23.16645241 | 22.90076447 |
|  | 1.255583736 | 0.215853373 | Q8BIJ7 | RUN and FYVE domain-containing protein 1 | Rufy1 | 23.24326134 | 22.98119926 | 23.08090973 | 22.93802834 | 22.85828972 | 22.86149216 |
|  | 0.274766098 | 0.132809957 | E9Q8N1;E9Q8K5;A0A5K1VVQ9;A2ASS6;F7CR78;A0A5K1VVQ1;F6RSJ3;A2AT70 | Titin | Ttn | 23.2401619 | NaN | 22.94374466 | 23.1915493 | 22.80752563 | 22.87835503 |
|  | 1.298133584 | 0.19051679 | Q91VH2 | Sorting nexin-9 | Snx9 | 23.23921585 | 23.18824768 | 23.27099037 | 23.09345627 | 22.91431618 | 23.11913109 |
|  | 0.665951806 | 0.214728991 | Q8JZN5;A0A0G2JDY4;A0A0G2JF25 | Acyl-CoA dehydrogenase family member 9, mitochondrial | Acad9 | 23.23597717 | 23.41015244 | 23.06536293 | NaN | 22.95751953 | 23.08735085 |
|  | 0.948201568 | -0.215422948 | Q9D2M8;A6X925;B2KF55 | Ubiquitin-conjugating enzyme E2 variant 2 | Ube2v2 | 23.23553848 | 23.24214172 | 23.13996124 | 23.5961647 | 23.42162514 | 23.24612045 |
|  | 0.388548601 | 0.190786997 | Q8K0C9 | GDP-mannose 4,6 dehydratase | Gmds | 23.23341942 | 23.10956573 | 23.05584717 | 23.05999374 | 22.55191994 | 23.21455765 |
|  | 0.241003567 | 0.055143992 | Q9D0B6 | Protein PBDC1 | Pbdc1 | 23.22776222 | 23.09600067 | 23.09998512 | 22.97100449 | 23.04948044 | 23.23783112 |
|  | 0.747730317 | -0.25228405 | Q9CXT8;D3Z1T9 | Mitochondrial-processing peptidase subunit beta | Pmpcb | 23.22374725 | 23.23151588 | 23.56197357 | 23.57680893 | NaN | 23.60591698 |
|  | 0.030381021 | 0.008014043 | P11688 | Integrin alpha-5;Integrin alpha-5 heavy chain;Integrin alpha-5 light chain | Itga5 | 23.21580124 | 23.49020386 | 23.36292458 | 23.27184677 | 23.40704346 | 23.36599731 |
|  | 0.727640862 | 0.25415802 | P13439 | Uridine 5-monophosphate synthase;Orotate phosphoribosyltransferase;Orotidine 5-phosphate decarboxylase | Umps | 23.21485329 | 22.95638466 | 22.73956299 | 22.67652702 | 22.59827995 | 22.8735199 |
|  | 0.758577342 | -0.109321594 | Q8C7X2;Z4YJW0;A0A1Y7VP81 | ER membrane protein complex subunit 1 | Emc1 | 23.21313286 | 23.19470787 | 23.30507851 | 23.26828003 | 23.31534195 | 23.45726204 |
|  | 0.109769389 | 0.042890549 | Q9D0W5 | Peptidyl-prolyl cis-trans isomerase-like 1 | Ppil1 | 23.212183 | 23.5308857 | 23.42367744 | 23.23435593 | 23.24606133 | 23.55765724 |
|  | 0.816990811 | 0.124010722 | Q80UM7 | Mannosyl-oligosaccharide glucosidase | Mogs | 23.21004486 | 23.0322094 | 23.12028503 | 23.08974457 | 22.92947578 | 22.97128677 |
|  | 0.171351107 | 0.068489075 | E9PYK0;Q8VCW4;A0A494BBK7;E9Q8P6 | Protein unc-93 homolog B1 | Unc93b1 | 23.20990944 | 23.45375061 | 23.31049538 | NaN | 23.10088348 | 23.41157532 |
|  | 0.477145462 | -0.137146632 | E9PUF7;Q61210;F6ZN61;A0A0U1RPP2;A0A0U1RPN7 | Rho guanine nucleotide exchange factor 1 | Arhgef1 | 23.20898819 | 23.22218513 | 23.05758286 | 23.48419189 | 23.09445572 | 23.32154846 |
|  | 0.250517751 | 0.197534561 | Q8VDM6 | Heterogeneous nuclear ribonucleoprotein U-like protein 1 | Hnrnpul1 | 23.20894432 | 23.00066948 | 22.66648483 | 23.14921761 | 22.89146805 | 22.2428093 |
|  | 1.034824618 | 0.236256282 | E9Q912;A0A0G2JGC8;A0A0G2JF70 | Rap1 GTPase-GDP dissociation stimulator 1 | Rap1gds1 | 23.20869064 | 23.15098572 | 23.22856903 | 23.034832 | 22.75307465 | 23.0915699 |
|  | 0.064028704 | -0.025362651 | Q9DBZ5;Q3TY56;A0A140LJ59 | Eukaryotic translation initiation factor 3 subunit K | Eif3k | 23.20799065 | 23.40106392 | 23.26713753 | 23.56302261 | 23.23867607 | 23.15058136 |
|  | 1.316603206 | 0.249962489 | Q9CWZ7;A0A494BAE3;A0A494BA20;D3Z4B2;A0A494BBF5;A0A494BA49;A0A494BAX2 | Gamma-soluble NSF attachment protein | Napg | 23.20760345 | 23.45362473 | 23.4777832 | 23.14622116 | 23.15545464 | 23.08744812 |
|  | 0.938182891 | 0.128931999 | Q9CX00 | IST1 homolog | Ist1 | 23.20709801 | 23.28489113 | 23.28093338 | 23.19404602 | 23.06337166 | NaN |
|  | 0.623692703 | -0.228676478 | Q80VQ0;J3QMK6;E9Q3E1;F6QPV9 | Aldehyde dehydrogenase family 3 member B1 | Aldh3b1 | 23.20423317 | 22.74392319 | 22.81870461 | 23.31644821 | 23.0616436 | 23.07479858 |
|  | 0.08937703 | 0.042036057 | A0A494B952;Q91V64 | Isochorismatase domain-containing protein 1 | Isoc1 | 23.19865417 | 23.27156067 | 23.01372528 | 22.96289253 | 23.41647911 | 22.97846031 |
|  | 0.010839592 | 0.005432129 | Q62189;D3Z0S6;D6RI83 | U1 small nuclear ribonucleoprotein A | Snrpa | 23.19784546 | 23.01930237 | 22.8937664 | 22.83551216 | 23.30201149 | 22.95709419 |
|  | 1.645205468 | -0.221251806 | A2AQ43;A2AQ44;A2AQ42;A2AQ45;A2AQ41;Q80TY0;A0A0A6YWT1;F6VVN1;A2AQ39;A2AQ47 | Formin-binding protein 1 | Fnbp1 | 23.19748497 | 23.24982834 | 23.27184677 | 23.43568039 | 23.5703373 | 23.37689781 |
|  | 0.42234488 | -0.101645788 | Q60710;F6TVP2;E9PYG9 | Deoxynucleoside triphosphate triphosphohydrolase SAMHD1 | Samhd1 | 23.19433212 | 23.24816322 | 23.18922806 | 23.12113953 | 23.351511 | 23.46401024 |
|  | 0.331934945 | 0.074253082 | Q9CYG7 | Mitochondrial import receptor subunit TOM34 | Tomm34 | 23.19102097 | 23.09165001 | 23.22368813 | 22.93686104 | 23.127388 | 23.21935081 |
|  | 0.095885249 | -0.023339589 | Q6P5F9;F6YA11;A2AKT6 | Exportin-1 | Xpo1 | 23.18721962 | 23.28121758 | 23.15120125 | 23.28460884 | 23.07608795 | 23.32896042 |
|  | 0.688374579 | -0.058734894 | Q8JZN2;Q91W50;A0A0G2JF72;A0A0G2JDJ7;A0A0G2JE62 | Cold shock domain-containing protein E1 | Csde1 | 23.18455887 | 23.28926086 | 23.18673706 | 23.31381989 | 23.26699448 | 23.25594711 |
|  | 0.831118201 | -0.148424149 | Q8JZR2;Q5ND50;Q64010;Q3TQV3;F7D232 | Adapter molecule crk | Crk | 23.18292427 | 23.39217949 | 23.1569519 | 23.46388626 | 23.34624481 | 23.36719704 |
|  | 0.055675051 | -0.019020081 | O35286;Q497W9;A0A0G2JG10;A0A0G2JGQ5 | Pre-mRNA-splicing factor ATP-dependent RNA helicase DHX15 | Dhx15 | 23.18213654 | 23.03557014 | 23.19260216 | 23.14864349 | 23.3436718 | 22.97505379 |
|  | 0.018713805 | 0.004046122 | Q9WU28;E9Q5Q8;E9Q093;E9PVG3;E9PZ62;H7BWX1 | Prefoldin subunit 5 | Pfdn5 | 23.18057442 | 23.01016045 | 23.12779808 | 23.11303711 | 23.00829887 | 23.18505859 |
|  | 0.333024066 | -0.115899404 | Q8K297 | Procollagen galactosyltransferase 1 | Colgalt1 | 23.17858505 | 23.03379059 | 23.17946625 | 23.06101608 | 23.50930023 | 23.16922379 |
|  | 0.367836862 | 0.144077937 | Q6XLQ8;O35887;G3V004;G3UXA8;G3UWV3;G3UWR0;G3UXA3;G3UY49 | Calumenin | Calu | 23.1784935 | 23.27454948 | 23.70281982 | 23.2983799 | 23.20269585 | 23.22255325 |
|  | 1.296533152 | -0.1431427 | Q6P8X1;A0A1W2P701;A0A494B972;Q80ZJ7;A0A1W2P6Y0 | Sorting nexin-6;Sorting nexin-6, N-terminally processed | Snx6 | 23.17736816 | 23.29473877 | 23.32416153 | 23.4596405 | 23.38968849 | 23.37636757 |
|  | 2.178392342 | 0.129663467 | A0A2I3BQL9;A0A0R4J0G0;Q8BH04;A0A2I3BS39;A0A2I3BRX1;A0A2I3BQ75;A0A2I3BQC6 | Phosphoenolpyruvate carboxykinase [GTP], mitochondrial | Pck2 | 23.17732239 | 23.10932732 | 23.11638832 | 23.0294323 | 22.99746895 | 22.98714638 |
|  | 0.580031009 | -0.155347824 | Q91V92;Q3V117;Q3TS02 | ATP-citrate synthase | Acly | 23.17531395 | 23.28883934 | 23.45136261 | 23.49679947 | 23.59240532 | 23.29235458 |
|  | 1.274212417 | -0.206673304 | P47811;A0A3B2WB60;B2KF34 | Mitogen-activated protein kinase 14 | Mapk14 | 23.17494774 | 23.41092873 | 23.26699448 | 23.55648804 | 23.45838928 | 23.45801353 |
|  | 0.934556025 | -0.160264015 | Q80UU9 | Membrane-associated progesterone receptor component 2 | Pgrmc2 | 23.17061615 | 23.21907043 | 23.16879654 | 23.44037819 | NaN | 23.25247192 |
|  | 0.956589479 | -0.267230988 | Q6PFB2;Q8VE37 | Regulator of chromosome condensation | Rcc1 | 23.16891861 | 23.30033684 | 23.29347801 | 23.70714569 | 23.56999016 | 23.28729057 |
|  | 0.07526445 | 0.035746257 | Q8CHP8 | Phosphoglycolate phosphatase | Pgp | 23.16606903 | 23.54203224 | 22.9982605 | 23.27084923 | 23.21144104 | 23.11683273 |
|  | 0.033334582 | 0.008390427 | D3YUK4;Q9DCS9 | NADH dehydrogenase [ubiquinone] 1 beta subcomplex subunit 10 | Ndufb10 | 23.16436768 | 22.94965172 | 23.14489937 | 23.17724609 | 23.0150547 | 23.04144669 |
|  | 0.810512206 | -0.259700775 | Q3TUQ7;Q5EG47;Q8BRK8 | 5-AMP-activated protein kinase catalytic subunit alpha-1 | Prkaa1 | 23.16134071 | 23.28149986 | 23.11219406 | 23.19109726 | 23.67206383 | 23.47097588 |
|  | 0.832377873 | 0.218208949 | G3UZ34;A2AH85;O08810;G3UXK8 | 116 kDa U5 small nuclear ribonucleoprotein component | Eftud2 | 23.16007996 | 22.95664978 | 23.09866905 | 22.70935631 | 22.79211426 | 23.05930138 |
|  | 0.346656176 | 0.115116755 | Q9JHS9;A0A1L1SVD8 | Spliceosome-associated protein CWC15 homolog | Cwc15 | 23.15946388 | 22.68795204 | 22.93012428 | 22.79084206 | 22.78939056 | 22.85195732 |
|  | 0.504035632 | -0.077672323 | Q8VBZ3 | Cleft lip and palate transmembrane protein 1 homolog | Clptm1 | 23.15943336 | 23.28714943 | 23.33388138 | 23.28517342 | 23.30591202 | 23.42239571 |
|  | 0.784508802 | -0.158666611 | Q60737;A2ANR6;Q6NSS6 | Casein kinase II subunit alpha | Csnk2a1 | 23.15705872 | 23.19022369 | 23.35353279 | 23.49692154 | 23.42265129 | 23.2572422 |
|  | 0.826980256 | -0.156320254 | Q9CQU0 | Thioredoxin domain-containing protein 12 | Txndc12 | 23.15658188 | NaN | 23.1828022 | 23.2026062 | 23.40171432 | 23.37371635 |
|  | 0.325287806 | -0.044197718 | G3XA17;F7CBP1;F6TW20 | Eukaryotic translation initiation factor 4, gamma 2 | Eif4g2 | 23.15407944 | 23.07111931 | 23.12952805 | 23.24298477 | 23.1738205 | 23.07051468 |
|  | 1.122690957 | 0.125707626 | P42669 | Transcriptional activator protein Pur-alpha | Pura | 23.15013313 | 23.25753021 | 23.2318821 | 23.0188961 | 23.16232491 | 23.08120155 |
|  | 0.272826308 | 0.066691717 | Q9EPE9 | Manganese-transporting ATPase 13A1 | Atp13a1 | 23.14965248 | 22.86625481 | 23.14254951 | 23.00225067 | 22.92803001 | 23.02810097 |
|  | 0.075721356 | 0.033290227 | Q8VEA8;D3YZQ6;D3Z4X4 | Ras-related protein Rab-7b | Rab7b;5430435G22Rik | 23.14958954 | 22.96944237 | 22.88513184 | 22.80640411 | 22.86508369 | 23.23280525 |
|  | 0.210050308 | -0.074506124 | Q8VHM5;F7B5B5;A2AW41 | Heterogeneous nuclear ribonucleoprotein R | Hnrnpr | 23.14614487 | 23.41866875 | 23.40626335 | 23.19573021 | 23.54674911 | 23.45211601 |
|  | 0.086919814 | -0.024445852 | S4R1B8;S4R270;A0A5F8MQ05;D3Z6Q9;S4R2J8;S4R171;S4R1Q1 | Bridging integrator 2 | Bin2 | 23.14192581 | 23.0772953 | 23.1600647 | 23.17759705 | 23.30326653 | 22.9717598 |
|  | 0.415943328 | 0.136620839 | A0A0G2JGI9;F8VPN4;F6XXE6;F7CSZ6;E0CYU6;E0CX86 | Glycogen debranching enzyme | Agl | 23.14134979 | 22.7745266 | 23.06863022 | 22.93214607 | 22.69161987 | 22.95087814 |
|  | 0.051861062 | 0.018607457 | O70274;Q63739 | Protein tyrosine phosphatase type IVA 2;Protein tyrosine phosphatase type IVA 1 | Ptp4a2;Ptp4a1 | 23.13136673 | 23.35810089 | 23.1288681 | 23.05223656 | 23.37610245 | 23.13417435 |
|  | 0.004219809 | -0.002250671 | Q505F5;E9PV22;F6Z4L9;F6YT33 | Leucine-rich repeat-containing protein 47 | Lrrc47 | 23.12603378 | 23.06088448 | 23.03682709 | 23.21409798 | 22.73405075 | 23.28234863 |
|  | 0.001648569 | 0.000768661 | P30416;F6S2D5;F7CAT1 | Peptidyl-prolyl cis-trans isomerase FKBP4;Peptidyl-prolyl cis-trans isomerase FKBP4, N-terminally processed | Fkbp4 | 23.12595558 | 23.02941513 | 22.80398369 | 22.87415886 | 23.22262764 | 22.86026192 |
|  | 0.802533702 | 0.455778122 | Q9D1M4 | Eukaryotic translation elongation factor 1 epsilon-1 | Eef1e1 | 23.12543488 | 23.24305725 | 23.24625015 | 23.18697929 | 22.28883934 | 22.77158928 |
|  | 0.16991936 | -0.051916122 | P47758;A0A1W2P830 | Signal recognition particle receptor subunit beta | Srprb | 23.12472534 | 23.07939529 | 22.9214344 | 23.08009529 | 22.93192863 | 23.26927948 |
|  | 0.256855312 | 0.056164424 | Q8C1E7;D3Z0U3 | Transmembrane protein 120A | Tmem120a | 23.12447357 | 23.07605553 | 23.13771248 | 23.16827583 | 22.88994789 | 23.11152458 |
|  | 2.125363709 | -0.175085386 | Q9CR26;F8WJC2;F6W5Q8 | Vacuolar protein sorting-associated protein VTA1 homolog | Vta1 | 23.12338448 | 23.13688278 | 23.11902046 | 23.33660698 | 23.26642227 | NaN |
|  | 1.233384476 | 0.117207845 | B2RXS4;Q3UH93 | Plexin-B2 | Plxnb2 | 23.12101364 | 23.20556259 | 23.1602478 | 23.11954308 | 23.0129261 | 23.00273132 |
|  | 0.730300249 | 0.037686666 | A0A1B0GRV0;Q9Z0S1;D3Z0E6;D3Z5X0 | 3(2),5-bisphosphate nucleotidase 1 | Bpnt1 | 23.11909866 | 23.08641052 | 23.16184807 | 23.07643127 | 23.07504272 | 23.10282326 |
|  | 1.098725516 | 0.157608668 | P61202;A2AQE4 | COP9 signalosome complex subunit 2 | Cops2 | 23.11791039 | 23.2407589 | 23.33878517 | 23.05511856 | 23.05147171 | 23.11803818 |
|  | 1.482708834 | 0.195052465 | H7BX01;P58281;E0CXD1;F6U775;P58682 | Dynamin-like 120 kDa protein, mitochondrial;Dynamin-like 120 kDa protein, form S1 | Opa1 | 23.11562729 | 23.11480141 | 23.11002731 | 23.02997208 | 22.82014656 | 22.90517998 |
|  | 0.620968301 | -1.032372157 | Q9Z1J3;F7CZD1;F6TXD3 | Cysteine desulfurase, mitochondrial | Nfs1 | 23.11503983 | 23.07612038 | 23.48456001 | 25.17630386 | NaN | 23.33892059 |
|  | 0.772945269 | 0.098300934 | Q91XU3 | Phosphatidylinositol 5-phosphate 4-kinase type-2 gamma | Pip4k2c | 23.11384773 | 23.10314369 | 23.02106667 | 22.99905205 | 22.88557816 | 23.05852509 |
|  | 0.316058847 | -0.070613543 | Q9QYS9;A0A3B2WCH2 | Protein quaking | Qki | 23.11343384 | 22.91426086 | 22.92163467 | 23.01728249 | 23.09016418 | NaN |
|  | 1.295084502 | 0.245178223 | Q8BTX9;A0A1D5RLG0;A0A1D5RM62 | Inactive hydroxysteroid dehydrogenase-like protein 1 | Hsdl1 | 23.10983658 | 22.95418549 | 23.26026154 | 22.86158752 | 22.87760735 | 22.84955406 |
|  | 0.358040191 | -0.135482788 | Q3UM45;F6TGJ2;A0A087WRA7 | Protein phosphatase 1 regulatory subunit 7 | Ppp1r7 | 23.10528755 | 23.09098816 | 22.99858856 | 23.10051537 | 23.50275993 | 22.99803734 |
|  | 0.291708521 | 0.076936086 | Q60766 | Immunity-related GTPase family M protein 1 | Irgm1 | 23.10283852 | 22.90525246 | 23.19249725 | 22.92565918 | 23.11938477 | 22.92473602 |
|  | 0.082350701 | -0.029724757 | P60670 | Nuclear protein localization protein 4 homolog | Nploc4 | 23.09762573 | 23.20097542 | 23.51304054 | 23.25364113 | 23.34516335 | 23.30201149 |
|  | 1.244578032 | -0.15958786 | A0A1W2P7G2;Q9D1J3;A0A1W2P6N2 | SAP domain-containing ribonucleoprotein | Sarnp | 23.09435844 | 23.27469254 | 23.13766479 | 23.30005836 | 23.38020515 | 23.30521584 |
|  | 1.412442593 | -0.152575175 | G3UYQ2;A0A1B0GX81;G3UZT6;Q3UF95;Q9Z1R2;G3UXT8;G3UYZ0;G3V013;S4R183;S4R224 | Large proline-rich protein BAG6 | Bag6 | 23.09187508 | 23.15268707 | 22.98764992 | 23.25479507 | 23.2046814 | 23.23046112 |
|  | 1.304161323 | 0.153255463 | Q3B7Z2;Q5QNQ6;Q5F209;Q8K0C7;Q5QNQ4 | Oxysterol-binding protein 1 | Osbp | 23.09074593 | 23.08713913 | 23.15421867 | 23.02446938 | 22.98952103 | 22.85834694 |
|  | 0.385993077 | 0.069864273 | Q9QYR9;O55137;Q32Q92;Q8BWN8;A0A1Y7VMZ4;Q91YQ6;Q6Q2Z6;Q9QYR7 | Acyl-coenzyme A thioesterase 2, mitochondrial;Acyl-coenzyme A thioesterase 1 | Acot2;Acot1 | 23.09026146 | 23.18369675 | 23.29852104 | 23.03942299 | 23.12227821 | 23.20118523 |
|  | 1.170242428 | -0.2315286 | P41241;A0A1L1SR46;A0A1L1STA1;A0A1L1SQQ5;D3YVQ8;D6RGA0;A0A0R4J1P8;D3Z4T5;A0A0R4J1N6;P41242 | Tyrosine-protein kinase CSK | Csk | 23.08916283 | 22.97249603 | 23.27113342 | 23.27895164 | 23.39113045 | 23.35729599 |
|  | 0.690364079 | 0.107730865 | Q9DCJ5 | NADH dehydrogenase [ubiquinone] 1 alpha subcomplex subunit 8 | Ndufa8 | 23.08903313 | 22.86398888 | 23.0342598 | 22.91078949 | 22.90795135 | 22.84534836 |
|  | 0.785335518 | -0.129202525 | Q6GQT9 | Nodal modulator 1 | Nomo1 | 23.0841732 | 22.83740044 | 22.95649147 | 23.04598618 | 23.08349228 | 23.13619423 |
|  | 0.197918185 | 0.04307429 | Q91WQ3;A2A7S7;F6VXZ2 | Tyrosine--tRNA ligase, cytoplasmic;Tyrosine--tRNA ligase, cytoplasmic, N-terminally processed;Tyrosine--tRNA ligase | Yars | 23.08180237 | 23.26184082 | 23.30813599 | 23.23737907 | 23.08151054 | 23.20366669 |
|  | 0.855290027 | -0.148164113 | E9PVA8 | Stalled ribosome sensor GCN1 | Gcn1l1 | 23.07962418 | 23.08383369 | 23.17430878 | 23.2347641 | 23.14695168 | 23.40054321 |
|  | 0.12218546 | -0.03071022 | Q6R891 | Neurabin-2 | Ppp1r9b | 23.07801247 | 23.02167702 | 22.99536514 | 23.07358932 | 23.20977592 | 22.90382004 |
|  | 0.322008538 | -0.157433192 | E9Q5H2;E9PZF5;P97822;E9Q0X5 | Acidic leucine-rich nuclear phosphoprotein 32 family member E | Anp32e | 23.07561493 | 22.99189186 | 23.36573029 | 23.05822754 | 23.22946358 | 23.61784554 |
|  | 0.483325759 | -0.06959788 | Q9QXX4 | Calcium-binding mitochondrial carrier protein Aralar2 | Slc25a13 | 23.07473373 | 23.12688446 | 23.02754402 | 23.15870857 | 23.23537827 | 23.04386902 |
|  | 0.195282451 | 0.069255193 | Q6P4T2 | U5 small nuclear ribonucleoprotein 200 kDa helicase | Snrnp200 | 23.07316399 | 22.85804176 | 22.9647274 | 22.86595345 | 22.70279884 | 23.11941528 |
|  | 0.358879577 | 0.092460632 | Q61187;A0A1B0GRX2;A0A1B0GS10;A0A1B0GS09;D3Z2V5;D3Z0S9 | Tumor susceptibility gene 101 protein | Tsg101 | 23.06817055 | 23.25479507 | 23.02337074 | 23.04971313 | 23.14735603 | 22.8718853 |
|  | 0.742114831 | 0.22624143 | P49586;D3Z3T5;Q811Q9 | Choline-phosphate cytidylyltransferase A | Pcyt1a | 23.06687355 | 22.96740341 | 22.84846497 | 22.84452438 | 22.48561668 | 22.87387657 |
|  | 0.709743227 | -0.50892512 | P54227;D3Z1Z8;D3Z5N2 | Stathmin | Stmn1 | 23.06674194 | 23.55133438 | 23.93741798 | 24.22442436 | 23.60918999 | 24.24865532 |
|  | 0.038663432 | 0.018939972 | Q9Z2Z6 | Mitochondrial carnitine/acylcarnitine carrier protein | Slc25a20 | 23.06376648 | 22.72486115 | 22.50295448 | 22.72916222 | 22.80785942 | 22.69774055 |
|  | 0.026450829 | -0.014816284 | P50427 | Steryl-sulfatase | Sts | 23.06289482 | 22.65092659 | 22.89074516 | 22.89975166 | 22.62401772 | 23.12524605 |
|  | 0.789524909 | 0.112613042 | A0A286YDI8;G3X972;A0A286YD08;A0A2I3BQS4 | Sec24 related gene family, member C (S. cerevisiae) | Sec24c | 23.05971336 | 22.93881798 | 22.87115288 | 22.9142971 | 22.82172394 | 22.79582405 |
|  | 0.291606073 | 0.099861145 | P27046;F6QMB7 | Alpha-mannosidase 2 | Man2a1 | 23.05966377 | 22.92469978 | 23.07033348 | 22.82650185 | 22.75293159 | 23.17568016 |
|  | 0.799189681 | 0.231756846 | A0A1W2P7C8;A0A0F6AIX5;A0A0F6AIX6;Q8BVA5;A0A1W2P7T7;A0A1W2P820;A0A1W2P6L7;A0A1W2P818;A0A1W2P6J8;A0A1W2P6H0 | UPF0554 protein C2orf43 homolog |  | 23.05905342 | 23.07133293 | 23.40210533 | 22.81554031 | 23.06815338 | 22.95352745 |
|  | 1.046187371 | -0.277531942 | P21995 | Embigin | Emb | 23.05205345 | 22.98375893 | 23.37610245 | 23.42675209 | 23.35836983 | 23.45938873 |
|  | 0.275413272 | 0.060738246 | Q9DCS3;A2A845 | Trans-2-enoyl-CoA reductase, mitochondrial | Mecr | 23.05045891 | 23.10707664 | 23.33524513 | 23.10078812 | 23.07695198 | 23.13282585 |
|  | 0.203275681 | -0.034641266 | Q9R1R8;Q9QYF1 | Retinol dehydrogenase 11 | Rdh11 | 23.04585266 | 22.97690582 | 23.03499985 | 22.95054054 | 23.04566956 | 23.16547203 |
|  | 0.265173368 | 0.058489482 | Q6PGL7;A0A0N4SUJ0;A0A0N4SV74 | WASH complex subunit FAM21 | Fam21 | 23.0437355 | 22.82390213 | 22.79296875 | 22.76486397 | 22.82063293 | 22.89964104 |
|  | 1.009368128 | -0.232756933 | Q8BTI8 | Serine/arginine repetitive matrix protein 2 | Srrm2 | 23.04218292 | 23.25333786 | 23.39597511 | 23.40600395 | 23.46051407 | 23.52324867 |
|  | 1.210531141 | -0.128588359 | Q9JJF9 | Signal peptide peptidase-like 2A | Sppl2a | 23.03866959 | 23.16490364 | 23.06340408 | 23.22152138 | 23.16094208 | 23.27027893 |
|  | 0.932156618 | 0.208897909 | O55126;Q7TMG8;A0A0G2JEV1 | Protein NipSnap homolog 2 | Gbas | 23.0350666 | 23.2172966 | 23.16949844 | NaN | 23.01855659 | 22.84488869 |
|  | 1.052688344 | 0.213010788 | E9PUD2;Q8K1M6;A0A2U3TZ67 | Dynamin-1-like protein | Dnm1l | 23.03246307 | 22.95103836 | 22.92469978 | 22.60458183 | 22.75058174 | 22.91400528 |
|  | 0.838722486 | 0.122894923 | Q9JHW2;A0A338P6G0;A0A338P7A1;A0A338P6Z4 | Omega-amidase NIT2 | Nit2 | 23.02561951 | 22.90297508 | 23.02529716 | 22.96569633 | 22.78214836 | 22.83736229 |
|  | 0.086438075 | 0.051468531 | Q9JII5;Q3UGB5;D3Z4J1 | DAZ-associated protein 1 | Dazap1 | 23.02458763 | 22.89254379 | 23.47159576 | 22.84898186 | 23.24222946 | 23.14311028 |
|  | 0.010518172 | -0.006664276 | A0A087WRN1;A0A087WQA0;F8WI22;Q8K019;A0A087WRF8 | Bcl-2-associated transcription factor 1 | Bclaf1 | 23.02196503 | 22.89443207 | 23.10346413 | 22.64246941 | 23.32759094 | 23.0697937 |
|  | 0.166403557 | 0.025375366 | Q91V76;A0A1L1SSF8;A0A1L1ST86 | Ester hydrolase C11orf54 homolog |  | 23.02113533 | 23.07098961 | 23.18925858 | 23.08024216 | 23.0140152 | 23.11100006 |
|  | 1.469819815 | 0.16773351 | Q8VD75;A0A0J9YUA3 | Huntingtin-interacting protein 1 | Hip1 | 23.01734924 | 23.07349205 | 23.13152313 | 22.94424438 | 22.82364845 | 22.95127106 |
|  | 0.360299096 | -0.198696772 | Q9ES46 | Beta-parvin | Parvb | 23.01638031 | 23.55367851 | 22.98111153 | 23.13340569 | 23.41144562 | 23.60240936 |
|  | 0.102506872 | -0.067614873 | P60605;G3UZX2 | Ubiquitin-conjugating enzyme E2 G2 | Ube2g2 | 23.0146122 | NaN | 23.40210533 | 23.54745483 | 23.07603836 | 23.20442772 |
|  | 0.488722949 | 0.118656794 | Q4FE56;P70398;F8VPU6;E9PWA9;G3UY52;G3UZS3 | Ubiquitin carboxyl-terminal hydrolase;Probable ubiquitin carboxyl-terminal hydrolase FAF-X | Usp9x | 23.01180077 | 22.79687309 | 23.03944016 | 22.92891502 | 22.87472153 | 22.68850708 |
|  | 0.190959775 | 0.035797119 | Q91YW3 | DnaJ homolog subfamily C member 3 | Dnajc3 | 23.00568008 | 23.223526 | 23.01190186 | 23.05207062 | 23.03441238 | 23.04723358 |
|  | 0.591118642 | -0.112286886 | O09106 | Histone deacetylase 1 | Hdac1 | 23.00135612 | 22.88773727 | 22.88228035 | 23.15052032 | 23.06409645 | 22.89361763 |
|  | 0.018019381 | -0.005303701 | Q9D7P6;D3Z7W0;D3Z0K9 | Iron-sulfur cluster assembly enzyme ISCU, mitochondrial | Iscu | 23.00008392 | 23.02553368 | 23.19436264 | 23.06529617 | 23.21728134 | 22.95331383 |
|  | 1.442293636 | 0.079919815 | Q99PV0;B7ZC27 | Pre-mRNA-processing-splicing factor 8 | Prpf8 | 22.9962616 | 22.92813873 | 22.94638824 | 22.86525536 | 22.90824318 | 22.85753059 |
|  | 0.453522475 | 0.247920354 | A0A0U1RPL0;Q7TQH0;E9Q5Q0;Q3TGG2;J3QP59 | Ataxin-2-like protein | Atxn2l | 22.99097633 | 23.31520462 | 23.0519371 | 22.89871788 | 22.48875809 | 23.22688103 |
|  | 0.055262671 | -0.012225469 | Q9EQ80;D3Z0G0;E9PWJ6 | NIF3-like protein 1 | Nif3l1 | 22.9907856 | 22.87844849 | 23.0024395 | 23.04276657 | NaN | 22.89613342 |
|  | 0.067478488 | 0.024028142 | Q5UE59;E9Q7C9;Q7TNF4;Q8CD76;A0A5F8MPZ2;O88447;D3YXZ3;Q91YS4;O88448;F6UYN4 | Kinesin light chain 1 | Klc1 | 22.98995399 | 22.97128677 | 23.13792992 | 22.82096481 | 23.21011925 | 22.9960022 |
|  | 1.298647789 | -0.112228394 | P70268;D6RH37 | Serine/threonine-protein kinase N1 | Pkn1 | 22.98936462 | 22.95682716 | 22.92822838 | 23.00734138 | 23.13359451 | 23.07016945 |
|  | 0.03107908 | 0.015221278 | Q9WTM5;A0A1B0GRW3;A0A1B0GSR4;A0A1B0GT54;A0A1B0GR89 | RuvB-like 2 | Ruvbl2 | 22.98744011 | 23.09424591 | 23.40483665 | 23.05941582 | 23.36078262 | 23.0206604 |
|  | 0.254685714 | 0.103695552 | Q8VDD8;A0A3B2WDA2 | WAS protein family homolog 1 | Wash1 | 22.97653961 | 23.46026421 | 23.37888336 | 23.18675232 | 23.05342865 | 23.26441956 |
|  | 0.939509325 | -0.051119486 | A0A0G2JG00;Q3TUE1;A0A0G2JGW9;A0A0G2JFY5;Q3UUU2;Q91WJ8;A0A0G2JFK2;A0A0G2JGV9 | Far upstream element-binding protein 1 | Fubp1 | 22.97515869 | 22.97893333 | 23.02245522 | 23.05158806 | 23.07380295 | 23.00451469 |
|  | 0.237214752 | -0.056993484 | Q63850;A0A140LJH5;A0A140LJ77 | Nuclear pore glycoprotein p62 | Nup62 | 22.97230339 | 22.79487228 | 23.06048965 | 22.90080261 | 23.01425362 | 23.08358955 |
|  | 0.857188761 | -0.275919596 | Q03958;G3UYF9;A0A3Q4EBT3 | Prefoldin subunit 6 | Pfdn6 | 22.97125053 | 22.86838531 | 23.2349987 | 23.12553024 | 23.47988129 | 23.29698181 |
|  | 0.34386272 | 0.162506739 | Q8BFY6 | Peflin | Pef1 | 22.96684074 | 22.9908371 | 22.9285183 | 22.45728683 | 23.13213539 | 22.80925369 |
|  | 0.657052348 | -0.77969869 | P56812;D3Z7Q5 | Programmed cell death protein 5 | Pdcd5 | 22.96610069 | 23.05751801 | 21.50392532 | 23.03451157 | 23.65501785 | 23.17711067 |
|  | 0.356865954 | -0.103192647 | Q9JI11;A0A2I3BQE0;Q9JI10;A2A5M9;Q8CDG4;F7BYZ4 | Serine/threonine-protein kinase 4;Serine/threonine-protein kinase 4 37kDa subunit;Serine/threonine-protein kinase 4 18kDa subunit | Stk4 | 22.96553802 | 23.29151154 | 23.17996597 | 23.13638306 | 23.22420311 | 23.38600731 |
|  | 0.129037748 | -0.035966873 | Q921W0 | Charged multivesicular body protein 1a | Chmp1a | 22.96354675 | 22.76368904 | 22.83271599 | 23.04969597 | 22.76654243 | 22.851614 |
|  | 0.464523726 | 0.17381986 | E9Q1J7;Q99MN9;D3YZC1;A0A087WQV1 | Propionyl-CoA carboxylase beta chain, mitochondrial | Pccb | 22.96167564 | 23.01081085 | 23.05996132 | 22.65193367 | 22.70497513 | 23.15407944 |
|  | 0.162615533 | 0.068963369 | P55096;A0A0G2JDI9 | ATP-binding cassette sub-family D member 3 | Abcd3 | 22.95746613 | 22.72161102 | 22.73469162 | 22.94753075 | 22.47147179 | 22.78787613 |
|  | 0.139873442 | 0.050278982 | Q91VJ4;A0A3B2WD34;A0A3B2WAZ4;A0A3B2W7T8;A0A3B2WD06;A0A3B2W7M3;A0A3B2W7L2 | Serine/threonine-protein kinase 38 | Stk38 | 22.95445061 | 23.25796127 | 23.03964043 | 22.87846756 | 23.21381569 | 23.00893211 |
|  | 0.423262315 | -0.23414739 | Q99K94;Q8C3V4;A0A087WSP5;P42225;A0A087WRI1;Q8CFQ1;A0A087WSQ5 | Signal transducer and activator of transcription;Signal transducer and activator of transcription 1 | Stat1 | 22.95336723 | 22.27960205 | 22.26530647 | 22.83452988 | 22.61315346 | 22.75303459 |
|  | 0.030936715 | 0.013771057 | Q6A0D4;D3Z731 | Raftlin | Rftn1 | 22.95253372 | 23.1635685 | 23.35487747 | 22.97964859 | 23.14201927 | 23.30799866 |
|  | 0.926196919 | -0.118672053 | Q8BX70;A0A1L1SUY8;A0A1L1SS63 | Vacuolar protein sorting-associated protein 13C | Vps13c | 22.94989967 | 23.01012802 | 22.9407177 | 23.08414078 | 22.98971176 | 23.18290901 |
|  | 1.541133608 | 0.130452474 | Q99JB2;A2AG39;F6WI02;A2AG41 | Stomatin-like protein 2, mitochondrial | Stoml2 | 22.94306374 | 23.03565407 | 23.02474022 | 22.82909966 | 22.86483955 | 22.91816139 |
|  | 0.030153981 | 0.018828074 | Q9CQC9 | GTP-binding protein SAR1b | Sar1b | 22.94129181 | 22.78849411 | 22.87639046 | 22.98337555 | 22.44659996 | 23.11971664 |
|  | 0.381972316 | -0.076678594 | Q9QXB9 | Developmentally-regulated GTP-binding protein 2 | Drg2 | 22.9412384 | 22.70882988 | 22.74079704 | 22.87843132 | 22.79718971 | 22.94528008 |
|  | 1.224234257 | -0.200160344 | Q7TNG5;D6RGM3;A0A0U1RQ42 | Echinoderm microtubule-associated protein-like 2 | Eml2 | 22.94012642 | 22.88242912 | 23.09221458 | 23.25652313 | 23.10562325 | 23.15310478 |
|  | 0.235231876 | 0.098061879 | P49282 | Natural resistance-associated macrophage protein 2 | Slc11a2 | 22.93081093 | 22.52013397 | 22.73539543 | NaN | 22.68987083 | 22.57156563 |
|  | 0.240368562 | 0.078400294 | O88545;D3Z0F5;F6QK86 | COP9 signalosome complex subunit 6 | Cops6 | 22.93046761 | 22.88395882 | 23.09218216 | 22.70777702 | 23.09413338 | 22.8694973 |
|  | 0.172416838 | 0.051267624 | Q8CEE7;A0A0R4J1N8;D3YVJ8 | Retinol dehydrogenase 13 | Rdh13 | 22.92988968 | 23.00727272 | 22.93355179 | 23.10179901 | 22.72254944 | 22.89256287 |
|  | 0.327822366 | 0.087206523 | Q9R1Q9;Q3TKX1;B7FAU3;F6ZE56;F6X9J0;B7FAU7 | V-type proton ATPase subunit S1 | Atp6ap1 | 22.9289875 | 23.13875771 | 22.86183357 | 22.76241112 | 23.00728989 | 22.89825821 |
|  | 0.070742443 | 0.027907054 | F6SLP4;A0A494BAW5;A0A494BAK4;Q9DC16;A0A494BAX4 | Endoplasmic reticulum-Golgi intermediate compartment protein 1 | Ergic1 | 22.92477226 | 22.96292877 | 23.34692192 | 23.04430199 | 23.00188828 | 23.10471153 |
|  | 0.631881315 | -0.295825322 | P10810 | Monocyte differentiation antigen CD14 | Cd14 | 22.92352104 | 23.27994347 | 23.33510971 | 23.74847603 | 23.17263031 | 23.50494385 |
|  | 0.329040334 | 0.11120669 | Q3UEB3 | Poly(U)-binding-splicing factor PUF60 | Puf60 | 22.92257881 | 22.56558037 | 22.84924889 | 22.54919624 | 22.61813545 | 22.8364563 |
|  | 0.146070334 | -0.04996109 | P97363 | Serine palmitoyltransferase 2 | Sptlc2 | 22.92159843 | 23.03779984 | 23.16884232 | 22.8854847 | 23.22767258 | 23.16496658 |
|  | 0.831531161 | -0.192380269 | Q8BFY9;Q3TKD0;J3QMX2 | Transportin-1 | Tnpo1 | 22.91522789 | 22.80937195 | 22.90032196 | 23.26770973 | 22.93346024 | 23.00089264 |
|  | 0.516860913 | -0.14976565 | Q9JKX6;A2ATT5;A0A0A6YVU1 | ADP-sugar pyrophosphatase | Nudt5 | 22.90510559 | 22.88492775 | 22.87190437 | 22.87904739 | 23.28785324 | 22.94433403 |
|  | 0.063717561 | 0.031799316 | A2AN08;F6SSP6;Z4YMA7;Z4YLP1 | E3 ubiquitin-protein ligase UBR4 | Ubr4 | 22.90481186 | 22.94023514 | 22.62827492 | 22.91799736 | 22.50767899 | 22.95224762 |
|  | 0.092411559 | -0.024819692 | Z4YJY0;Q3TCJ1;D3Z4D8;D6RJ39 | BRISC complex subunit Abro1 | Fam175b | 22.90394783 | 22.71458435 | 22.78100777 | 22.68226051 | 22.95139503 | 22.84034348 |
|  | 0.717435697 | -0.263188044 | Q7TNC4;E9Q715;Q05CX5 | Putative RNA-binding protein Luc7-like 2 | Luc7l2 | 22.90343475 | 22.70438385 | 23.12401581 | 23.18598175 | NaN | 23.16161728 |
|  | 0.560520523 | 0.190793037 | P58021;E9PZ69 | Transmembrane 9 superfamily member 2 | Tm9sf2 | 22.90021324 | 23.07594109 | 23.24141502 | NaN | 22.79322624 | 22.97023392 |
|  | 0.130732666 | 0.027702967 | Q99KH8;A2AD84;Q99JT2;D3Z359;Q9Z2W1 | Serine/threonine-protein kinase 24;Serine/threonine-protein kinase 24 35 kDa subunit;Serine/threonine-protein kinase 24 12 kDa subunit | Stk24 | 22.89989853 | 22.72039986 | 22.73392677 | 22.76995659 | 22.84049606 | 22.6606636 |
|  | 0.048725686 | -0.007123947 | A2AL50;Q8C0I1;H3BKN2;A2AL49;H3BIY5 | Alkyldihydroxyacetonephosphate synthase, peroxisomal | Agps | 22.89840508 | 22.80677795 | 22.88786697 | 22.86519814 | 22.80392456 | 22.94529915 |
|  | 0.09674699 | 0.040196101 | P60122;D3YW60 | RuvB-like 1 | Ruvbl1 | 22.89668846 | 22.79655647 | 23.1294651 | 23.04415131 | 22.68155289 | 22.97641754 |
|  | 0.293575325 | 0.091224035 | A0A0G2JFT8;Q9D394 | Protein RUFY3 | Rufy3 | 22.89500618 | 22.75936699 | 23.15533066 | 22.88054276 | 22.74970245 | 22.90578651 |
|  | 0.164798831 | 0.093445142 | Q8BJ71;A0A1D5RLQ0;A0A1D5RM86 | Nuclear pore complex protein Nup93 | Nup93 | 22.89458084 | 22.72822952 | 22.66114235 | NaN | 22.92027092 | 22.41547394 |
|  | 0.209581501 | -0.059254328 | Q9ESW8;A0A1B0GSY1;A0A1B0GSC4 | Pyroglutamyl-peptidase 1 | Pgpep1 | 22.89359856 | 22.79845619 | 22.73223114 | 22.74949837 | NaN | 22.98520088 |
|  | 0.022305599 | -0.006307602 | Q7TNV0;E9Q8Y1;D3YVJ6 | Protein DEK | Dek | 22.88716125 | 22.77187157 | 22.7801857 | 22.80717087 | 22.67551422 | 22.97545624 |
|  | 0.000334134 | 9.06E-05 | Q91YP3;A0A0N4SV34 | Deoxyribose-phosphate aldolase | Dera | 22.87742043 | 22.6858139 | 22.80620766 | 22.853899 | NaN | 22.72554779 |
|  | 0.470894152 | -0.116365433 | Q9CPX6 | Ubiquitin-like-conjugating enzyme ATG3 | Atg3 | 22.87186623 | 22.88334274 | 23.01864052 | 22.85180473 | 23.16514969 | 23.10599136 |
|  | 1.423019813 | 0.224127452 | Q8C052;A0A1D5RLY6;A0A1D5RMG7 | Microtubule-associated protein 1S;MAP1S heavy chain;MAP1S light chain | Map1s | 22.87182999 | 22.87824249 | 22.81950378 | 22.76322365 | 22.61438942 | 22.51958084 |
|  | 0.632918994 | 0.163092931 | G3X922;D4AFX7;A0A087WRC9;A0A1L1STR9;A0A087WS25 | DnaJ heat shock protein family (Hsp40) member C13 | Dnajc13 | 22.87141609 | 22.7359314 | 23.12008095 | 22.79459572 | 22.69487381 | 22.74868011 |
|  | 0.25712247 | 0.033068975 | O55091;A0A3Q4EC12;A0A3Q4L314 | Protein IMPACT | Impact | 22.87130356 | 22.82170486 | 22.91716003 | 22.80067062 | 22.92283249 | 22.78745842 |
|  | 0.718710465 | 0.095667521 | Q3U816;Q9Z2G9 | Oxidoreductase HTATIP2 | Htatip2 | 22.86680222 | 22.87730789 | 22.94728088 | 22.76878548 | 22.72615051 | 22.90945244 |
|  | 0.433617453 | -0.135335922 | A2AJI1;A8Y5P4;A2AJI0;F6WF36 | MAP7 domain-containing protein 1 | Map7d1 | 22.8657074 | 23.2130146 | 23.16855049 | 23.35070229 | 23.08349228 | 23.21908569 |
|  | 0.181776075 | -0.059530258 | Q62446;A0A1Y7VLK0;A0A1Y7VMJ9;A0A1Y7VP01;A0A1Y7VJ86 | Peptidyl-prolyl cis-trans isomerase FKBP3 | Fkbp3 | 22.8639698 | 23.00816154 | 23.27980042 | 23.09034157 | 23.0779953 | 23.16218567 |
|  | 0.377371504 | -0.102269491 | O70475;D3YXP9 | UDP-glucose 6-dehydrogenase | Ugdh | 22.86058235 | 22.74392319 | 22.5703373 | 22.85555458 | 22.6831398 | 22.94295692 |
|  | 0.874680407 | 0.19586436 | P10711;E9PYD5;B7ZCS4;F6XMY4;Q9QVN7 | Transcription elongation factor A protein 1 | Tcea1 | 22.85405159 | 22.93003464 | 22.86878204 | 22.83732414 | 22.73376083 | 22.49419022 |
|  | 0.055034864 | 0.028253555 | J3QMM7;K3W4M4;Q9CZ42;J3QN06;J3QPU6 | ATP-dependent (S)-NAD(P)H-hydrate dehydratase | Carkd | 22.85300446 | 22.53865051 | 22.79320717 | 22.79463387 | 22.89668846 | 22.40877914 |
|  | 0.340790561 | 0.138621012 | Q8R3D1;D6RI34;D6RJI8 | TBC1 domain family member 13 | Tbc1d13 | 22.84687996 | 22.6975708 | 22.61892128 | 22.66162109 | 22.2844677 | 22.80142021 |
|  | 0.307411614 | 0.120210012 | Q8C5R8 | ribose-phosphate diphosphokinase | Prps1l1 | 22.83982468 | 22.98198318 | 22.60927963 | 22.88220406 | 22.7089138 | 22.4793396 |
|  | 0.721992421 | 0.18995285 | P47856;D3YYD9;D3YYE0 | Glutamine--fructose-6-phosphate aminotransferase [isomerizing] 1 | Gfpt1 | 22.83449173 | 22.71561241 | 22.86640549 | 22.65999031 | 22.40447235 | 22.78218842 |
|  | 0.147627096 | -0.089241982 | A0A0R4J1G9;E9QN92;Q8CI59;D3YTP0 | Metalloreductase STEAP3 | Steap3 | 22.8312664 | NaN | 22.42290878 | 22.76407433 | NaN | 22.66858482 |
|  | 0.49966623 | 0.067082723 | A2AKI5;P43406;F6RWM8;F6W6Q6 | Integrin alpha-V;Integrin alpha-V heavy chain;Integrin alpha-V light chain | Itgav | 22.83016396 | 22.90460968 | 22.91905403 | 22.72494507 | 22.90398598 | 22.82364845 |
|  | 0.304636705 | 0.080429713 | Q80VP0 | Tectonin beta-propeller repeat-containing protein 1 | Tecpr1 | 22.83010674 | 22.88777351 | 23.12721634 | 22.76437759 | 22.9620285 | 22.87740135 |
|  | 0.912487326 | 0.11746788 | P48410;A2ALN0 | ATP-binding cassette sub-family D member 1 | Abcd1 | 22.82977676 | 22.88199997 | 22.87663269 | 22.70015526 | 22.86001396 | 22.67583656 |
|  | 0.083300173 | -0.040545146 | Q91XD6 | Vacuolar protein-sorting-associated protein 36 | Vps36 | 22.8292923 | 22.57110214 | 22.68865585 | 22.57276726 | 23.04720116 | 22.59071732 |
|  | 0.048644555 | 0.015528361 | Q3TDN2;D3Z2H4;D3YUP4;D6RE51;D3Z4G9 | FAS-associated factor 2 | Faf2 | 22.82679367 | 22.90483093 | 23.05573082 | 22.98820496 | 22.74131203 | 23.01125336 |
|  | 1.242466876 | -0.264883359 | Q8BUM3 | Tyrosine-protein phosphatase non-receptor type 7 | Ptpn7 | 22.82665825 | 22.69376945 | 22.6259594 | 23.03965759 | NaN | 22.92103386 |
|  | 0.527855575 | -0.443232218 | A0A087WNM1;Q91Z67;A0A087WRV4;A0A087WSQ1;A0A087WS59;D3YZW1;Q91Z69;A0A087WNR5 | SLIT-ROBO Rho GTPase-activating protein 2 | Srgap2 | 22.82564926 | 22.68367577 | 22.10881615 | 22.70252419 | 23.26270103 | NaN |
|  | 1.294403652 | -0.255210241 | Q9D892 | Inosine triphosphate pyrophosphatase | Itpa | 22.8235321 | 22.90587807 | 23.01916695 | 23.18387794 | 23.03923988 | 23.29109001 |
|  | 0.292658109 | -0.071847916 | Q3TIR6;P61759 | Prefoldin subunit 3 | Vbp1 | 22.82279396 | 23.07880974 | 22.85207176 | 23.03469658 | 23.05935097 | 22.87517166 |
|  | 0.15138641 | 0.046885808 | Q921K2;P11103;A0A0A6YY63 | Poly [ADP-ribose] polymerase 1 | Parp1 | 22.82016563 | 22.72052574 | 22.65691757 | 22.61894226 | 22.54594803 | 22.89206123 |
|  | 0.667007787 | -0.171129862 | O88543;D3Z036;F6YCA7 | COP9 signalosome complex subunit 3 | Cops3 | 22.81952286 | 23.01670456 | 22.94968605 | NaN | 22.99282646 | 23.20670891 |
|  | 0.056509005 | -0.025115331 | Q99KE1 | NAD-dependent malic enzyme, mitochondrial | Me2 | 22.81739616 | 22.58118439 | 22.44118881 | 22.80045319 | 22.43542671 | 22.67923546 |
|  | 0.16412889 | 0.050961812 | Q61205;D3Z2X5;Q8CA83;D3Z7E6 | Platelet-activating factor acetylhydrolase IB subunit gamma | Pafah1b3 | 22.81692886 | 22.87781334 | 22.93405533 | 22.61281586 | 22.9921875 | 22.87090874 |
|  | 0.093124094 | 0.032382329 | Q61191;B1AUX2;F6SJS2 | Host cell factor 1;HCF N-terminal chain 1;HCF N-terminal chain 2;HCF N-terminal chain 3;HCF N-terminal chain 4;HCF N-terminal chain 5;HCF N-terminal chain 6;HCF C-terminal chain 1;HCF C-terminal chain 2;HCF C-terminal chain 3;HCF C-terminal chain 4;HCF C-terminal chain 5;HCF C-terminal chain 6 | Hcfc1 | 22.81436729 | 22.45269394 | 22.56639481 | 22.61703873 | 22.66399002 | 22.4552803 |
|  | 0.706287876 | -0.275720596 | A0A0G2JEA9;A0A0G2JFX7;Q9CWZ3;A0A0N4SUH6 | RNA-binding protein 8A | Rbm8a | 22.81352615 | 22.493433 | 22.70672417 | 23.05802917 | 22.64763832 | 23.13517761 |
|  | 0.541741517 | -0.14801089 | Q8VH51;E9Q8F0;F7AA45;B7ZD61;B7ZD63 | RNA-binding protein 39 | Rbm39 | 22.81313515 | 22.92018127 | 22.67432976 | 22.8533287 | 23.14546013 | 22.85289001 |
|  | 0.308031669 | 0.141820908 | P47941;A0A338P6Q0;A0A338P675 | Crk-like protein | Crkl | 22.80689621 | 22.58205795 | 22.27383804 | 22.57010651 | 22.45793724 | 22.20928574 |
|  | 0.331848073 | -0.039990107 | E9Q0U7;Q61699;D3Z3I9;A0A0J9YTZ7;D3Z027 | Heat shock protein 105 kDa | Hsph1 | 22.80400276 | 22.73886108 | 22.8382473 | 22.88851738 | 22.85720634 | 22.75535774 |
|  | 0.736269878 | -0.202147166 | Q9QUJ7 | Long-chain-fatty-acid--CoA ligase 4 | Acsl4 | 22.80290031 | 23.09187508 | 22.81393814 | 23.00805855 | 23.03667641 | 23.27042007 |
|  | 0.537818942 | -0.096438726 | Q61334 | B-cell receptor-associated protein 29 | Bcap29 | 22.80104446 | 22.6979084 | 22.79847717 | 22.9492588 | 22.91694069 | 22.72054672 |
|  | 0.209476521 | 0.093748728 | Q9D8N2;Q3TH34;D6RIQ6;D3YUG1 | Protein FAM45A | Fam45a | 22.7981987 | 22.91774368 | 23.1877346 | 22.76708794 | NaN | 22.98119926 |
|  | 0.274950838 | -0.08867836 | O88983;Q9D0J1;Q8BS59 | Syntaxin-8 | Stx8 | 22.79764557 | 22.76235199 | 22.74896622 | 23.11456299 | 22.75013351 | 22.71030235 |
|  | 1.175586664 | 0.307516734 | P35831;F6Z0X5;D6RGT2 | Tyrosine-protein phosphatase non-receptor type 12 | Ptpn12 | 22.79507065 | 22.95790863 | 22.9711113 | 22.8178463 | 22.47335625 | 22.51033783 |
|  | 1.261135653 | -0.226434708 | Q5KU39 | Vacuolar protein sorting-associated protein 41 homolog | Vps41 | 22.78984833 | 22.70607185 | 22.8864727 | 23.01087761 | 22.91104507 | 23.13977432 |
|  | 0.088959608 | 0.046440125 | P31750;D3Z783;D3YXX3;D3YYP9;Q9WUA6 | RAC-alpha serine/threonine-protein kinase | Akt1 | 22.7891922 | 22.64409828 | 22.77715874 | 22.3767128 | 22.99922371 | 22.69519234 |
|  | 0.057505295 | 0.033217112 | O88842;Q3TNB8;A0A0R4J1D9 | FYVE, RhoGEF and PH domain-containing protein 3 | Fgd3 | 22.77289772 | 23.03345299 | 23.03664398 | 23.05581474 | 23.12973213 | 22.55779648 |
|  | 0.198496961 | 0.068916957 | A0A0R3P9C8;Q9DC69 | NADH dehydrogenase [ubiquinone] 1 alpha subcomplex subunit 9, mitochondrial | Ndufa9 | 22.76870537 | 23.08872604 | 23.19386673 | 22.88278389 | 23.01685715 | 22.94490623 |
|  | 0.330091709 | 0.1167895 | F6ZDS4;Q7M739;F6RX08 | Nucleoprotein TPR | Tpr | 22.76621819 | 22.72598457 | 22.36396599 | 22.55723572 | 22.36343193 | 22.5851326 |
|  | 0.182806375 | 0.079258601 | P70677;A0A1B0GRX1 | Caspase-3;Caspase-3 subunit p17;Caspase-3 subunit p12 | Casp3 | 22.76603699 | 22.72768784 | 22.77046013 | 23.00463486 | 22.51828575 | 22.50348854 |
|  | 0.202114132 | -0.04192543 | E9QA74;E9Q405;A0A1C7ZN10;K3W4L0;B2RRE2;E9QAX2;Q9JMH9 | Unconventional myosin-XVIIIa | Myo18a | 22.76559258 | 22.57082367 | 22.59650612 | 22.58318329 | 22.74359512 | 22.73192024 |
|  | 0.599195558 | -0.266340574 | P97287 | Induced myeloid leukemia cell differentiation protein Mcl-1 homolog | Mcl1 | 22.76176262 | 22.44242859 | 22.60295296 | 23.06406403 | 22.6733799 | NaN |
|  | 0.771892636 | -0.193463008 | Q9DCC4 | Pyrroline-5-carboxylate reductase 3 | Pycrl | 22.7556839 | 22.47652435 | 22.74414825 | 22.81197929 | 22.75482941 | 22.98993683 |
|  | 0.663983093 | -0.190605799 | Q3UHX2 | 28 kDa heat- and acid-stable phosphoprotein | Pdap1 | 22.7535038 | 22.9501667 | 23.11410141 | 23.27951813 | 23.01779175 | 23.09227943 |
|  | 0.650739016 | -0.190775553 | E9QLA5;Q0GNC1;A0A1Y7VM80 | Inverted formin-2 | Inf2 | 22.7521553 | 22.98292351 | 23.09976196 | 23.29991722 | 23.08830452 | 23.01894569 |
|  | 0.009874219 | 0.008649826 | Q8BGU5 | Cyclin-Y | Ccny | 22.74281311 | 22.55702591 | NaN | 23.06307602 | 22.38695526 | 22.47377777 |
|  | 0.124461542 | -0.039361954 | H7BX99;P19221 | Prothrombin;Prothrombin;Activation peptide fragment 1;Activation peptide fragment 2;Thrombin light chain;Thrombin heavy chain | F2 | 22.74166107 | 22.86953354 | 23.12272072 | 22.94041443 | 22.90606117 | 23.00552559 |
|  | 0.656287439 | -0.145814896 | Q91ZR1 | Ras-related protein Rab-4B | Rab4b | 22.73620033 | 22.79685402 | 23.05143929 | 22.95143127 | 23.03116608 | 23.03934097 |
|  | 0.106070253 | 0.026988347 | P18654;B1AXN9 | Ribosomal protein S6 kinase alpha-3 | Rps6ka3 | 22.73599434 | 22.7283535 | 22.93790245 | 22.7932663 | 22.7542572 | NaN |
|  | 0.688820008 | -0.069457054 | Q99KU0;Q5SXR2 | Vacuole membrane protein 1 | Vmp1 | 22.73591042 | 22.69684792 | 22.62283516 | 22.74906921 | 22.76024055 | NaN |
|  | 0.501639894 | 0.183869998 | P46737;E9Q0P6;A3KGA8 | Lys-63-specific deubiquitinase BRCC36 | Brcc3 | 22.73402977 | 22.35172844 | 22.54992485 | 22.28754425 | NaN | 22.43517113 |
|  | 0.063927568 | -0.033612569 | Q5MJS3;E0CY01;Q8CID3 | Extracellular serine/threonine protein kinase FAM20C | Fam20c | 22.72957802 | NaN | 22.627985 | 22.53385925 | 22.6248436 | 22.97847939 |
|  | 0.144912386 | 0.064748128 | Q8R2Y8 | Peptidyl-tRNA hydrolase 2, mitochondrial | Ptrh2 | 22.72750092 | 22.40043831 | 22.56010818 | 22.30966377 | 22.76318169 | 22.42095757 |
|  | 0.351510288 | -0.3744723 | E9PW20;Q9CY57;D3YZA1;D3Z7T7 | Chromatin target of PRMT1 protein | Chtop | 22.72246552 | 23.69646645 | 23.93102837 | 24.29073906 | 23.70893478 | 23.47370338 |
|  | 1.190522653 | 0.245126088 | Q9DCT2 | NADH dehydrogenase [ubiquinone] iron-sulfur protein 3, mitochondrial | Ndufs3 | 22.72012901 | 22.71749496 | 22.83638 | 22.36709023 | 22.49884605 | 22.67268944 |
|  | 0.846265243 | -0.1967055 | Q62261;A0A0A0MQG2;E9Q397;Q3UGX2;P15508 | Spectrin beta chain, non-erythrocytic 1 | Sptbn1 | 22.71634483 | 22.81470108 | 22.85321426 | 22.80862617 | 23.01289177 | 23.15285873 |
|  | 0.524130873 | 0.02031517 | Q91ZA3;D3YWM4;H3BL62 | Propionyl-CoA carboxylase alpha chain, mitochondrial | Pcca | 22.71634483 | 22.7033062 | 22.75855446 | 22.71253014 | 22.70066261 | 22.70406723 |
|  | 0.873621001 | -0.544423421 | P29595;A0A2I3BRG0 | NEDD8 | Nedd8 | 22.71061897 | 22.59882545 | 22.84525299 | 23.75011253 | 22.77601433 | 23.26184082 |
|  | 1.253424409 | 0.109860738 | E9Q9C3;Q9QZQ1;E9Q852;E9PYX7;F7C3I9;D3Z7L2;D3YUD2 | Afadin | Mllt4 | 22.70284081 | 22.66242599 | 22.70748329 | 22.65811729 | 22.54571342 | 22.53933716 |
|  | 1.535168449 | 0.1901172 | D3YZ86;A0A571BDP7;Q8VDV3;D3Z585 | Guanine nucleotide exchange factor for Rab-3A | Rab3il1 | 22.702631 | 22.80901909 | 22.7602005 | 22.60401726 | NaN | 22.5303154 |
|  | 0.553757279 | 0.220087687 | Q78IK4;B1AV14 | MICOS complex subunit Mic27 | Apool | 22.70207977 | 22.66983986 | 22.84151459 | 22.8426857 | 22.28133011 | 22.42915535 |
|  | 0.315203333 | 0.04442215 | Q61024 | Asparagine synthetase [glutamine-hydrolyzing] | Asns | 22.70140457 | 22.80254555 | 22.76013947 | 22.67831039 | 22.64486885 | 22.80764389 |
|  | 0.119288205 | -0.030409495 | Q8BZA9 | Fructose-2,6-bisphosphatase TIGAR | Tigar | 22.70100212 | 22.57602501 | 22.78508568 | 22.85549736 | 22.62444115 | 22.67340279 |
|  | 0.670475363 | 0.16906325 | Q9DCM0 | Persulfide dioxygenase ETHE1, mitochondrial | Ethe1 | 22.68903923 | 22.8979435 | 22.96511459 | 22.67538452 | NaN | 22.68788719 |
|  | 0.181971253 | 0.066990852 | G3XA66;Q8R5C0;A0A6I8MWZ8;P47802;G3XA75;D3YVW0;D3Z3F4;G3UYJ5;G3UXX9;G3UXB5 | Metaxin-1 | Mtx1 | 22.68741798 | 22.33628082 | 22.49399376 | 22.38687515 | 22.49093819 | NaN |
|  | 1.142777121 | 0.17240715 | Q80X90 | Filamin-B | Flnb | 22.6848526 | 22.81878281 | 22.76995659 | 22.6816597 | 22.59707451 | 22.47763634 |
|  | 0.182983788 | 0.092539469 | O08915;D3YW40 | AH receptor-interacting protein | Aip | 22.68198204 | 22.68284035 | 22.8824482 | 22.74882317 | 22.30760956 | 22.91321945 |
|  | 0.414924862 | -0.109759649 | Q91YS7;Q63932;M0QWN2;A0A1W2P7V9 | Dual specificity mitogen-activated protein kinase kinase 2 | Map2k2 | 22.67962265 | 22.56704521 | 22.58740044 | 22.88877678 | 22.75268745 | 22.52188301 |
|  | 1.551162925 | 0.20877711 | Q7TSI3;A0A0U1RQ86;A0A0U1RNI2 | Serine/threonine-protein phosphatase 6 regulatory subunit 1 | Ppp6r1 | 22.67904282 | 22.56360435 | 22.56685829 | 22.48937035 | 22.32580757 | 22.36799622 |
|  | 0.311376942 | -0.108381271 | Q5ND34;K4DI77;F6XD87 | WD repeat-containing protein 81 | Wdr81 | 22.67826843 | 22.3329525 | 22.63358307 | 22.7966156 | 22.48350334 | 22.68982887 |
|  | 0.251634933 | -0.11100324 | Q9ES74;Q3TN15 | Serine/threonine-protein kinase Nek7 | Nek7 | 22.67616081 | 22.34740829 | 22.64790154 | 22.58157539 | 22.48123741 | 22.94166756 |
|  | 0.336344129 | 0.096827825 | Q9ERU9 | E3 SUMO-protein ligase RanBP2 | Ranbp2 | 22.67613792 | 22.6254673 | 22.77289772 | 22.78134727 | 22.39824867 | 22.60442352 |
|  | 0.749672071 | -0.241444906 | Q6W4W7;O70566;E9Q4U7 | Protein diaphanous homolog 2 | Diap2;Diaph2 | 22.67182732 | 22.59459496 | 22.59030533 | 22.67329407 | 23.14699936 | 22.76076889 |
|  | 0.191842827 | -0.053063711 | P61290;A2A4J1;A2A4J3 | Proteasome activator complex subunit 3 | Psme3 | 22.67154694 | 22.86022377 | 22.97655678 | 22.84804535 | 22.81624413 | 23.00322914 |
|  | 0.449042819 | 0.067830086 | Q9CWW6 | Peptidyl-prolyl cis-trans isomerase NIMA-interacting 4 | Pin4 | 22.67001152 | NaN | 22.64037323 | 22.53240967 | 22.64231491 | NaN |
|  | 0.923191112 | 0.146867116 | Q9WV85 | Nucleoside diphosphate kinase 3 | Nme3 | 22.66882324 | 22.67495346 | 22.72060966 | 22.45462799 | 22.62789536 | NaN |
|  | 0.811408783 | 0.092215856 | Q9WUP7;A0A087WP81;A0A087WRL3 | Ubiquitin carboxyl-terminal hydrolase isozyme L5 | Uchl5 | 22.66754532 | 22.79016685 | 22.69848251 | 22.56527901 | 22.61948013 | 22.69478798 |
|  | 0.617187626 | 0.372081121 | Q99KI3 | ER membrane protein complex subunit 3 | Emc3 | 22.66438103 | 22.79534912 | 22.67833328 | NaN | 22.00535393 | 22.67585945 |
|  | 0.450493105 | 0.080716451 | O09110;A0A0R4J1Q6;A2AGS2;P70236 | Dual specificity mitogen-activated protein kinase kinase 3 | Map2k3 | 22.65942383 | 22.80825233 | 22.56271935 | 22.65024757 | 22.5503006 | 22.58769798 |
|  | 0.895490945 | 0.254751841 | D3YUM1;Q91YT0;D3Z1U9;D6RG60;A0A494BAS8;D3Z0K1;D3YXX5 | NADH dehydrogenase [ubiquinone] flavoprotein 1, mitochondrial | Ndufv1 | 22.65894508 | 22.40051651 | 22.57056999 | 22.26730919 | 22.48684502 | 22.11162186 |
|  | 0.619866009 | -0.149609248 | Q8BXC6;G3X955 | COMM domain-containing protein 2 | Commd2 | 22.65778923 | 22.51989365 | 22.38635063 | 22.65013885 | NaN | 22.69176865 |
|  | 0.795512599 | -0.137245814 | O35841 | Apoptosis inhibitor 5 | Api5 | 22.65693855 | 22.74112701 | 22.4757576 | 22.73326492 | 22.76906776 | 22.78322792 |
|  | 2.077973 | 0.149968465 | Q8K4Z5 | Splicing factor 3A subunit 1 | Sf3a1 | 22.6541214 | 22.69835472 | 22.73208618 | 22.55931473 | 22.57212067 | 22.50322151 |
|  | 1.055823459 | 0.293974559 | Q8BZN6;A0A0R4J2B7;A0A087WRP5;A0A087WS26;Q8BLX9;A0A087WQA1 | Dedicator of cytokinesis protein 10 | Dock10 | 22.65125656 | 22.7455864 | 22.76753235 | 22.40387344 | 22.222229 | 22.65634918 |
|  | 0.831170379 | 0.207758904 | Q91VN4;E9Q4M4 | MICOS complex subunit Mic25 | Chchd6 | 22.65068626 | 22.57454681 | 22.83580208 | 22.42864418 | 22.52986145 | NaN |
|  | 0.142756058 | -0.068958282 | A0A0U1RPM2;P16879;A0A0U1RNL3;A0A0U1RPB9;A0A0U1RQ80 | Tyrosine-protein kinase Fes/Fps | Fes | 22.649086 | 22.08540726 | 22.14491653 | 22.34201813 | 22.37891006 | 22.36535645 |
|  | 0.974562506 | -0.209718704 | P61967;D3Z268;D3Z0D6;D3YXN0;B1B0F6;Q8BW87 | AP-1 complex subunit sigma-1A | Ap1s1 | 22.64548492 | 22.46525574 | 22.6374588 | 22.63593102 | 22.91265488 | 22.82876968 |
|  | 1.800626409 | 0.204387665 | Q9CZW5 | Mitochondrial import receptor subunit TOM70 | Tomm70a | 22.63692665 | 22.55992317 | 22.61717224 | 22.399786 | 22.47891998 | 22.32215309 |
|  | 0.904996549 | 0.447386106 | Q8QZS1;E0CX19 | 3-hydroxyisobutyryl-CoA hydrolase, mitochondrial | Hibch | 22.63358307 | 22.88803482 | 22.71404076 | 22.04600143 | 22.54966545 | NaN |
|  | 0.250253133 | -0.067671458 | E9PV14;A2AUK7;A2AUK8;A2AUK5;Q9Z2H5;A0A2R8VHB6 | Band 4.1-like protein 1 | Epb4.1l1;Epb41l1 | 22.62896347 | 22.6238842 | 22.72949409 | 22.92806625 | 22.59671211 | 22.66057777 |
|  | 0.03759165 | -0.004847844 | E0CYJ0;E0CXA9;Q6PEB6;G8JL35 | MOB-like protein phocein | Mob4 | 22.60914421 | 22.58735466 | 22.60733986 | 22.57800865 | 22.54947853 | 22.69089508 |
|  | 0.981002447 | 0.297679901 | Q3UDR8;F6YIY0 | Protein YIPF3;Protein YIPF3, N-terminally processed;Protein YIPF | Yipf3 | 22.60394859 | 22.44009781 | 22.26610756 | 21.94799423 | 22.16551781 | 22.30360222 |
|  | 0.073607155 | -0.032466888 | Q3ULW8;A0A5H1ZRP0;A0A5H1ZRL8;A0A5H1ZRL5 | Poly (ADP-ribose) polymerase family, member 3 | Parp3 | 22.60148048 | 22.81593132 | NaN | 22.64288712 | 22.83945847 | NaN |
|  | 0.264985009 | -0.055464427 | Q9D2N9;A0A0G2JEL2 | Vacuolar protein sorting-associated protein 33A | Vps33a | 22.59830284 | 22.69735718 | 22.60331535 | 22.68365288 | 22.8243866 | 22.55732918 |
|  | 0.307973841 | 0.072608312 | E9QP49;A0A494BBL8;A0A494BA91;Q99MS7;A0A494B9G7;G5E8Y6 | EH domain-binding protein 1-like protein 1 | Ehbp1l1 | 22.59714317 | 22.45861435 | 22.35089111 | 22.37777138 | NaN | 22.41477776 |
|  | 0.743631831 | 0.098271688 | D6RE33;A0A0R4J1Q0;G5E896;Q3UJB9;F6ZJ27;F6V5I7 | Enhancer of mRNA-decapping protein 4 | Edc4 | 22.59343147 | 22.56092453 | 22.42398643 | 22.48583794 | 22.37824821 | 22.41944122 |
|  | 0.007332209 | 0.003102303 | Z4YKT6;Q99J47 | Dehydrogenase/reductase SDR family member 7B | Dhrs7b | 22.59005356 | 22.49684906 | 22.46749687 | 22.68619919 | NaN | 22.34386253 |
|  | 0.039338456 | -0.00914828 | D3Z656;Q8CHC4;E9Q7S0;F7BQW7;D3Z1M7;F6VSS8;A0A338P6C7;A0A338P6V7;F7CD11 | Synaptojanin-1 | Synj1 | 22.58675957 | 22.6950016 | 22.64105797 | 22.63865089 | 22.78110695 | 22.53050613 |
|  | 0.216907354 | -0.049679438 | Q99NH8 | Triggering receptor expressed on myeloid cells 2 | Trem2 | 22.58609581 | 22.49592209 | 22.30869293 | 22.46879005 | 22.5835495 | 22.48740959 |
|  | 0.330340735 | 0.065880458 | A0A3B2WCL5;Q9CT10 | Ran-binding protein 3 | Ranbp3 | 22.58327484 | 22.67678452 | 22.5365181 | 22.4904232 | 22.43690109 | 22.67161179 |
|  | 0.539282665 | 0.225581487 | Q8JZR0;A0A286YCG4;A0A286YD68 | Long-chain-fatty-acid--CoA ligase 5 | Acsl5 | 22.57925224 | 23.00219917 | 22.9127636 | 22.52078056 | 22.43117142 | 22.86551857 |
|  | 0.499834718 | 0.379362106 | E9QKA4;A2A8V8;A2A8V9;E9PUK6;Q52KI8;F6T4M4;A2A983;F6UK16 | Serine/arginine repetitive matrix protein 1 | Srrm1 | 22.57745552 | 23.01597214 | NaN | 22.60154724 | 22.2331562 | NaN |
|  | 0.476840055 | 0.086797714 | Q8BWQ6;D3YW20;D3YW19;I1E4X5;F6RR19;H3BJE8 | UPF0505 protein C16orf62 homolog | 9030624J02Rik | 22.57251358 | 22.57175064 | 22.5882473 | 22.57047653 | 22.33308983 | 22.56855202 |
|  | 0.324317805 | -0.104668299 | Q8BRF7;A0A1W2P6R7 | Sec1 family domain-containing protein 1 | Scfd1 | 22.56901741 | 22.75276947 | 22.88721657 | 22.67109299 | 22.8515377 | 23.00037766 |
|  | 0.414821527 | 0.177106857 | Q8CC88 | von Willebrand factor A domain-containing protein 8 | Vwa8 | 22.5643959 | 22.3208046 | 22.30234718 | 21.96589088 | 22.51732445 | 22.17301178 |
|  | 0.302213849 | -0.111538887 | Q8R574 | Phosphoribosyl pyrophosphate synthase-associated protein 2 | Prpsap2 | 22.56206703 | 22.17858505 | 22.38861084 | NaN | 22.45425224 | 22.52166748 |
|  | 0.275750237 | 0.038824399 | Q3TL72;Q8C878 | NEDD8-activating enzyme E1 catalytic subunit | Uba3 | 22.55810165 | NaN | 22.49633598 | 22.47748756 | 22.55994606 | 22.42774963 |
|  | 1.195839705 | -0.238208135 | O55106;F8WH41;F6Z700 | Striatin | Strn | 22.55510712 | 22.6674366 | 22.72392464 | 23.0234375 | 22.88926125 | 22.74839401 |
|  | 0.149713121 | -0.050961177 | Q3UHJ0;A0A571BDM4 | AP2-associated protein kinase 1 | Aak1 | 22.54759598 | 22.18716049 | 22.28537178 | 22.48770332 | 22.2617836 | 22.42352486 |
|  | 0.088988098 | -0.03592364 | A0A0R4J052;G5E8T9;Q99KB8;E9PYA3;E9Q2H8;D3YUX8;D3YWI0 | Hydroxyacylglutathione hydrolase, mitochondrial | Hagh | 22.53464317 | 22.59053421 | 22.83616829 | 22.77376366 | 22.82316208 | 22.47219086 |
|  | 0.613709339 | -0.596234639 | Q80Y14;A0A1Y7VN70 | Glutaredoxin-related protein 5, mitochondrial | Glrx5 | 22.53100586 | 22.84465981 | 21.91173935 | 22.7430191 | 23.30772018 | NaN |
|  | 0.633659514 | 0.301480293 | Q8BIW1 | Protein prune homolog | Prune | 22.52260208 | 22.17157745 | NaN | 22.01576805 | NaN | 22.0754509 |
|  | 0.194276255 | 0.106757482 | D3Z6S1;Q8BM55 | Transmembrane protein 214 | Tmem214 | 22.52245712 | 22.76038361 | 22.45941544 | 22.19709587 | 22.83766937 | 22.38721848 |
|  | 0.275016311 | -0.104862849 | A0A494BAQ2;A0A494B9Y5;Q9WTK5 | Nuclear factor NF-kappa-B p100 subunit;Nuclear factor NF-kappa-B p52 subunit | Nfkb2 | 22.51234055 | 22.70824051 | 22.62602615 | 22.44101143 | 22.81487656 | 22.90530777 |
|  | 0.17282625 | 0.085851034 | Q78IS1 | Transmembrane emp24 domain-containing protein 3 | Tmed3 | 22.50971031 | 22.75117493 | 22.63911629 | 22.57427025 | 22.83566666 | 22.23251152 |
|  | 1.23242834 | 0.143053691 | A6PWC3;A2A9Q2;Q8BHG1;Q3V3G9 | Nardilysin | Nrd1 | 22.50743675 | 22.40215683 | 22.39142036 | 22.35863876 | 22.29316902 | 22.22004509 |
|  | 0.31788061 | 0.130618095 | Q8K4M5;F7BCN0;F7BZY0;Q8VI86;G8JL54 | COMM domain-containing protein 1 | Commd1;Gm28048 | 22.50579071 | 22.58856773 | 22.89624405 | 22.60614204 | NaN | 22.45969009 |
|  | 0.811214613 | 0.319643974 | Q9ERG0 | LIM domain and actin-binding protein 1 | Lima1 | 22.4994545 | 22.48721313 | 22.34497261 | NaN | 21.91480827 | 22.33366394 |
|  | 1.072106237 | 0.319194158 | G3X956;Q920B9 | FACT complex subunit SPT16 | Supt16;Supt16h | 22.49211311 | 22.82586288 | 22.6638813 | 22.1507225 | 22.37660599 | 22.49694633 |
|  | 0.26394721 | -0.063197454 | P0DN34 | NADH dehydrogenase [ubiquinone] 1 beta subcomplex subunit 1 | Ndufb1 | 22.4908638 | 22.3507843 | 22.32190514 | 22.35555077 | 22.38748169 | 22.61011314 |
|  | 0.833829215 | -0.227695465 | O35551;J3QJV7;Q3U983 | Rab GTPase-binding effector protein 1 | Rabep1 | 22.48291206 | 22.44242859 | 22.18310547 | 22.4904232 | 22.53604317 | 22.76506615 |
|  | 0.586367233 | 0.218397776 | Q6PAR5;F7ADT6;F7ADQ2;E9Q0D1;F7ADS7;F6X819 | GTPase-activating protein and VPS9 domain-containing protein 1 | Gapvd1 | 22.47486687 | 22.09152031 | 22.30669212 | 22.14640808 | 22.24042511 | 21.83105278 |
|  | 0.624202719 | 0.270513535 | P14576;E9PXC0;A0A1W2P809;A0A1Y7VJJ0 | Signal recognition particle 54 kDa protein | Srp54;Srp54c | 22.47008133 | 22.26859474 | 22.3449192 | 22.31539726 | NaN | 21.86597252 |
|  | 0.589316963 | 0.181614558 | Q8CI11;A0A2I3BRV9;A0A2I3BPZ6;A0A2I3BR82;A0A2I3BR32 | Guanine nucleotide-binding protein-like 3 | Gnl3 | 22.46839142 | 22.29440308 | 22.13467598 | 22.16916275 | NaN | 22.06592178 |
|  | 0.300710058 | -0.069859187 | Q3THK7 | GMP synthase [glutamine-hydrolyzing] | Gmps | 22.46578026 | 22.62598038 | 22.77307892 | 22.73554039 | 22.62874222 | 22.71013451 |
|  | 0.027264055 | 0.014676094 | P25206 | DNA replication licensing factor MCM3 | Mcm3 | 22.46263885 | 22.36238861 | 22.79766464 | 22.59295273 | 22.45948982 | NaN |
|  | 0.222747881 | 0.076250712 | Q922H4;D3Z3L0;D3Z3M0;D3Z2L8;D3YVK2;D3Z5Z5;D3Z0T7 | Mannose-1-phosphate guanyltransferase alpha | Gmppa | 22.45660973 | 22.45798874 | 22.8131752 | 22.46376038 | 22.41575813 | 22.61950302 |
|  | 0.389701161 | 0.0552152 | Q8VBV7;A0A087WPM5 | COP9 signalosome complex subunit 8 | Cops8 | 22.45314789 | 22.41964722 | 22.46018982 | 22.27235794 | 22.45116043 | 22.44382095 |
|  | 0.075819623 | 0.023123423 | Q99MR6;A0A0G2JG65;A0A0G2JDF8;A0A0G2JDN3;A0A1Y7VNN4;A0A0G2JDF1 | Serrate RNA effector molecule homolog | Srrt | 22.45216751 | 22.60070801 | 22.68942451 | 22.56485939 | 22.69574547 | 22.41232491 |
|  | 0.249243293 | -0.053504308 | Q6ZQ58;Z4YJT3;Q9D423 | La-related protein 1 | Larp1 | 22.45153809 | 22.48777771 | 22.41645432 | 22.63579941 | 22.35267067 | 22.52781296 |
|  | 0.661224195 | -0.098890305 | S4R2J9;A0A0A0MQ79;Q3TLH4;S4R294;S4R2L9;S4R209;S4R2E2 | Protein PRRC2C | Prrc2c | 22.44151688 | 22.54080391 | 22.40937614 | 22.52864647 | 22.67042351 | 22.48929787 |
|  | 0.529559076 | -0.144967397 | Q99K85;Q3U6K9;E9Q6P1 | Phosphoserine aminotransferase | Psat1 | 22.43789101 | 22.24522018 | 22.04956245 | 22.38463783 | 22.31484413 | 22.46809387 |
|  | 1.181700818 | 0.16762352 | H3BKN0;Q1HFZ0 | tRNA (cytosine(34)-C(5))-methyltransferase | Nsun2 | 22.43489265 | 22.55342102 | 22.40488815 | 22.33027458 | 22.35960388 | 22.2004528 |
|  | 0.160573391 | -0.050409317 | Q9ERB0 | Synaptosomal-associated protein 29 | Snap29 | 22.43163109 | 22.47850037 | 22.71592522 | 22.61683655 | 22.56801987 | NaN |
|  | 0.259198814 | 0.045130412 | P56380 | Bis(5-nucleosyl)-tetraphosphatase [asymmetrical] | Nudt2 | 22.43081474 | 22.39675903 | 22.5359726 | 22.32808495 | 22.38527107 | 22.51479912 |
|  | 0.232326758 | 0.119593302 | A0A0R4IZY0;Q8C1A5 | Thimet oligopeptidase | Thop1 | 22.42963982 | 22.61941338 | 22.94927597 | 22.56479073 | NaN | 22.52824211 |
|  | 0.028517832 | -0.011159897 | Q5FWX7;E9Q6C1;F6UHR6;E9PXZ2;A0A0A6YXH3;A2A8Z1 | Oxysterol-binding protein;Oxysterol-binding protein-related protein 9 | Osbpl9 | 22.42321587 | NaN | 22.17943573 | 22.29018974 | NaN | 22.33478165 |
|  | 0.667388603 | -0.326759974 | A0A1D5RMC1;Q6PDL0;A0A1D5RM94 | Cytoplasmic dynein 1 light intermediate chain 2 | Dync1li2 | 22.41573334 | 23.00099564 | 22.55069923 | 22.87906647 | 23.24867058 | 22.81997108 |
|  | 0.629132632 | 0.217188517 | Q9Z0H8 | CAP-Gly domain-containing linker protein 2 | Clip2 | 22.41408157 | 22.47062874 | 22.39018631 | 22.48202515 | 21.950737 | 22.19056892 |
|  | 0.258955086 | -0.156709035 | A0A0R4J0B4;Q99KK2;A0A0N4SW65;A0A0N4SWC3 | N-acylneuraminate cytidylyltransferase | Cmas | 22.41384888 | 22.98551369 | 22.73353386 | 22.52303314 | 23.09440613 | 22.98558426 |
|  | 0.556025689 | -0.313735962 | Q8BYA0;F6UI15;B1ATU0;Q9D2H8 | Tubulin-specific chaperone D | Tbcd | 22.41142082 | 22.43692589 | 22.19171524 | 22.63085365 | 22.26436234 | 23.08605385 |
|  | 0.780894423 | 0.338335991 | Q9D8S9 | BolA-like protein 1 | Bola1 | 22.40947914 | 22.62949753 | 22.58847618 | 22.42375565 | NaN | 21.98454094 |
|  | 0.702911225 | -0.303860982 | Q8CE50 | Sorting nexin-30 | Snx30 | 22.40660095 | 22.28028297 | 22.4122467 | 22.31843376 | 22.71097565 | 22.98130417 |
|  | 1.076895695 | -0.192025503 | E9QKE4;A0A0A6YWM5;Q8BMG7 | Rab3 GTPase-activating protein non-catalytic subunit | Rab3gap2 | 22.40111542 | 22.52781296 | 22.39217949 | 22.67538452 | 22.49284554 | 22.72895432 |
|  | 0.024036098 | 0.004677137 | Q9Z0G0;A0A1D5RML2 | PDZ domain-containing protein GIPC1 | Gipc1 | 22.39636803 | 22.48706627 | 22.31608772 | 22.39291382 | 22.39741325 | NaN |
|  | 2.085119876 | 0.147066434 | Q8BYK6 | YTH domain-containing family protein 3 | Ythdf3 | 22.38758659 | 22.37875175 | 22.33030128 | NaN | 22.22493935 | 22.21202087 |
|  | 0.3047464 | -0.124915759 | P04202 | Transforming growth factor beta-1;Latency-associated peptide | Tgfb1 | 22.38171005 | 22.02201462 | 22.13614845 | 22.18999672 | NaN | 22.41975021 |
|  | 0.289354525 | -0.204201698 | P61965;F6Q3W0 | WD repeat-containing protein 5 | Wdr5 | 22.38023186 | 21.83945847 | 22.40867615 | 22.22746849 | 22.59984589 | NaN |
|  | 0.389367237 | -0.126168569 | I1E4X0;F6YMR0;E9PUF2;B7ZNS2;B1AZP2;H3BJW9;H3BKJ0;H3BL90;H3BJD4 | Disks large-associated protein 4 | Dlgap4 | 22.37642097 | 22.26582146 | 22.53957367 | 22.30195618 | 22.66561699 | 22.59274864 |
|  | 0.953283712 | 0.218686422 | Q91WC9 | Sn1-specific diacylglycerol lipase beta | Daglb | 22.37501526 | 22.62578011 | 22.30860901 | 22.28627396 | 22.23908424 | 22.12798691 |
|  | 0.70693479 | -0.110056241 | Q9CR98;A0A0N4SWI4 | Protein FAM136A | Fam136a | 22.36908913 | 22.50263786 | 22.36337852 | 22.48000526 | NaN | 22.56351089 |
|  | 0.562829088 | 0.064702988 | E9Q7G0;F6ZQA3 | Nuclear mitotic apparatus protein 1 | Numa1 | 22.36396599 | 22.36581039 | 22.25237083 | 22.29260635 | 22.19367027 | 22.30176163 |
|  | 0.732548931 | -0.264523824 | Q9EQQ9 | Protein O-GlcNAcase | Mgea5 | 22.3613987 | 22.0790062 | 22.48113823 | 22.5878582 | 22.55555153 | NaN |
|  | 0.114048448 | -0.070696195 | Q9DBE8;F6RBY3 | Alpha-1,3/1,6-mannosyltransferase ALG2 | Alg2 | 22.35519981 | 22.83580208 | 22.32824898 | NaN | 22.50244522 | 22.65178108 |
|  | 0.581679953 | -0.123167038 | Q9QXK3 | Coatomer subunit gamma-2 | Copg2 | 22.35064888 | 22.3345356 | 22.43786621 | 22.32303429 | 22.61328697 | 22.55623055 |
|  | 1.466894077 | -0.183451653 | Q3UE37 | Ubiquitin-conjugating enzyme E2 Z | Ube2z | 22.35016251 | 22.37687111 | NaN | 22.5148468 | 22.57909012 | NaN |
|  | 0.896761139 | 0.322683016 | P46061;A0A2R8W753;A0A2R8VHK9 | Ran GTPase-activating protein 1 | Rangap1 | 22.34640694 | 22.05705452 | 22.19979286 | 21.7312355 | NaN | 22.02556801 |
|  | 0.73727098 | 0.177062988 | Q3UID0;Q6PDG5;A0A1W2P6N7;Q3UNN4;P97496 | SWI/SNF complex subunit SMARCC2 | Smarcc2 | 22.33570862 | 22.60424232 | 22.45068359 | 22.31525993 | NaN | 22.25770378 |
|  | 1.333897973 | 0.229136785 | A0A1B0GRT6;A0A1B0GRH2;P49446 | Receptor-type tyrosine-protein phosphatase epsilon | Ptpre | 22.32600021 | 22.17809868 | 22.43435669 | 22.13677406 | 22.08394623 | 22.03032494 |
|  | 0.716342877 | 0.163696925 | O35127 | Protein C10 | Grcc10 | 22.32421494 | 22.21682358 | 22.11981201 | 22.18721962 | 21.8938961 | 22.08864403 |
|  | 0.526634827 | -0.185222626 | A0A140LHA2;Q9WVA3;A0A140LJ21;A0A140LI47;A0A140LIM5 | Mitotic checkpoint protein BUB3 | Bub3 | 22.31603241 | 22.66674423 | 22.57835388 | 22.60569 | 22.93185616 | 22.57925224 |
|  | 0.752558136 | -0.173591614 | B1AVH7 | TBC1 domain family member 2A | Tbc1d2 | 22.31467819 | 22.37917328 | 22.39923859 | 22.33448219 | 22.61333275 | 22.66604996 |
|  | 1.073240386 | -0.256244024 | P30999;E9Q986;E9Q8Z6;E9Q8Z5;G3X9V2;E9Q904;E9Q901;E9Q903;D3Z2H2;E9Q8Z9;E9Q8Z4;E9Q905;E9Q907;E9Q906;D3Z7H6;E9Q8Z8;D3Z2H7 | Catenin delta-1 | Ctnnd1 | 22.31465149 | 22.47459412 | 22.64132309 | 22.64882278 | 22.69924545 | 22.85123253 |
|  | 0.269088567 | 0.099594752 | A0A1D5RLS1;Q60902 | Epidermal growth factor receptor substrate 15-like 1 | Eps15l1 | 22.31310081 | 22.50518608 | 22.48155785 | 22.23765564 | 22.16278648 | 22.60061836 |
|  | 0.723001567 | 0.321481705 | Q64521;A2AQR0 | Glycerol-3-phosphate dehydrogenase, mitochondrial;Glycerol-3-phosphate dehydrogenase | Gpd2 | 22.31196594 | 22.47422218 | 22.41982651 | 21.68624306 | 22.30541229 | 22.24991417 |
|  | 0.885407091 | 0.177956899 | Q91YP2;A0A286YD12;A0A286YD77 | Neurolysin, mitochondrial | Nln | 22.30563354 | 22.22864342 | 22.39652443 | 21.97379303 | 22.23160362 | 22.19153404 |
|  | 1.253929814 | -0.221439362 | Q9EQG9 | Collagen type IV alpha-3-binding protein | Col4a3bp | 22.30005836 | 22.11546707 | 22.35307503 | 22.48628044 | 22.40259933 | 22.54403877 |
|  | 0.498198362 | -0.113835017 | Q5JC28;H3BK65;P42567;F6W2Q5;H3BJB8;A0A0R4J0A0;H3BLE4 | Epidermal growth factor receptor substrate 15 | Eps15 | 22.2998333 | 22.30259705 | 22.27866745 | 22.524086 | 22.20961189 | 22.48890495 |
|  | 1.123909969 | -0.145827611 | Q9DC23 | DnaJ homolog subfamily C member 10 | Dnajc10 | 22.28881073 | 22.21317863 | 22.34459305 | 22.34662437 | 22.42570305 | 22.51173782 |
|  | 0.072153372 | -0.040236791 | O35382;Q8C391;Q9CXE1 | Exocyst complex component 4 | Exoc4 | 22.28700829 | 22.31121635 | 22.50132751 | 22.1754818 | 22.27605629 | 22.76872444 |
|  | 0.424483285 | -0.231031418 | Q99LE6 | ATP-binding cassette sub-family F member 2 | Abcf2 | 22.27395248 | 22.69046783 | 22.98567009 | 23.0342598 | 22.93272209 | 22.67620277 |
|  | 1.066651721 | 0.330108643 | O35857 | Mitochondrial import inner membrane translocase subunit TIM44 | Timm44 | 22.27381134 | 22.50792122 | 22.31160545 | 22.27900696 | 21.96469116 | 21.85931396 |
|  | 0.014838004 | 0.004588445 | P49615;A0A0G2JDL3 | Cyclin-dependent-like kinase 5 | Cdk5 | 22.27287102 | 22.29642105 | 22.39876938 | 22.34948921 | 22.14039803 | 22.46440887 |
|  | 0.409928901 | 0.075700442 | H3BJ30;H3BJW3;Q6NVF9;H3BKW0 | Cleavage and polyadenylation specificity factor subunit 6 | Cpsf6 | 22.27116203 | 22.47109985 | 22.35259056 | 22.27863884 | 22.29986191 | NaN |
|  | 0.374145421 | -0.22959741 | Q6PGF7 | Exocyst complex component 8 | Exoc8 | 22.25919914 | 21.97228622 | 22.49313927 | 22.67725754 | NaN | 22.26502037 |
|  | 0.168391442 | -0.060295105 | E9QMK9;Q8BH86 | UPF0317 protein C14orf159 homolog, mitochondrial | 9030617O03Rik | 22.25577354 | 22.23470688 | 22.54924393 | 22.38721848 | NaN | 22.4265213 |
|  | 0.50373533 | -0.140354156 | Q6PDI5;A2ALV7;A2ALV6;A2ALV8;A2ALV9;A2ALW1 | Proteasome-associated protein ECM29 homolog | Ecm29;AI314180 | 22.25413132 | 22.12933922 | 21.91353035 | 22.14777565 | 22.19264793 | 22.37763977 |
|  | 0.193507229 | -0.078312556 | Z4YJU8;A0A6I8MWY5;E9PUQ5;A0A6I8MX07;Q921M4;A2AN45;A2AN48;A2AN46 | Golgin subfamily A member 2 | Golga2 | 22.24895859 | 22.2320137 | 22.07551765 | 22.27315521 | 22.51031494 | 22.00795746 |
|  | 0.125935585 | -0.039235433 | Q14CH1 | Molybdenum cofactor sulfurase | Mocos | 22.24565506 | 22.28816223 | 22.51905441 | 22.5402832 | 22.29227066 | 22.33802414 |
|  | 0.873955855 | -0.322875659 | Q80XI4 | Phosphatidylinositol 5-phosphate 4-kinase type-2 beta | Pip4k2b | 22.2418499 | 21.9915123 | 22.20176888 | NaN | 22.63187599 | 22.30396271 |
|  | 0.344077146 | 0.050894419 | E9Q1S3;Q01405 | Protein transport protein Sec23A | Sec23a | 22.24001694 | 22.44500923 | 22.36575699 | 22.27227402 | 22.30810928 | 22.3177166 |
|  | 0.700989765 | -0.135350227 | O35864;A0A087WQA8;A0A087WQ60;A0A087WRH6 | COP9 signalosome complex subunit 5 | Cops5 | 22.23818016 | 22.31746864 | NaN | 22.3536129 | NaN | 22.47273636 |
|  | 0.294964494 | -0.116404851 | Q8BLN5;F7BJL0 | Lanosterol synthase | Lss | 22.23385811 | 21.95386505 | 21.95404243 | 22.03291702 | NaN | 22.29440308 |
|  | 0.925437231 | -0.223924637 | A0A087WQM0;A0A087WRU0;A0A087WQ94;A0A6I8MWZ2;A0A087WQS0;A0A1D5RM59;E9Q0S6;Q9DBT6 | Tensin 1 | Tns1 | 22.23198509 | 22.24950981 | 22.3345089 | 22.71053314 | 22.36032677 | 22.4169178 |
|  | 0.26165822 | -0.103781382 | Q8BJF9;A0A338P7L8 | Charged multivesicular body protein 2b | Chmp2b | 22.23116493 | 22.34475708 | 22.49313927 | 22.7361393 | 22.34694862 | 22.2973175 |
|  | 0.731149604 | -0.085092545 | Q80YW0;A0A2R8VI37;A0A2R8VKC6;A0A2R8VHE0;A0A2R8W712 | Cytohesin-4 | Cyth4 | 22.22423172 | 22.22831917 | 22.11352921 | 22.19856453 | 22.31791115 | 22.30488205 |
|  | 0.427910783 | -0.264158885 | Q99P31;A0A0U1RPF2;A0A0U1RPE7;A0A0U1RQ49 | Hsp70-binding protein 1 | Hspbp1 | 22.2083931 | 22.55615997 | 22.65471268 | 22.49162292 | 22.98287201 | NaN |
|  | 0.68435954 | -0.435538292 | Q8CCH2 | NHL repeat-containing protein 3 | Nhlrc3 | 22.20102119 | 21.93854904 | NaN | 22.30860901 | NaN | 22.70203781 |
|  | 0.111444761 | 0.02952226 | Q8VDK1;D3YY53;D3Z2Y2;D3Z3I3 | Nitrilase homolog 1 | Nit1 | 22.19940376 | 21.98974609 | 22.02695274 | NaN | 22.10027504 | 21.98474884 |
|  | 0.137571099 | -0.040876389 | A0A1L1STE4;Q9Z1X4;Q45VK5;A0A1L1SU19;A0A1L1SQR7;A0A1L1SR62;A0A1L1SQ69;A0A1L1SSU8 | Interleukin enhancer-binding factor 3 | Ilf3 | 22.19625473 | 22.25652313 | 22.19117165 | 22.08660507 | 22.45585823 | 22.22411537 |
|  | 0.931530524 | 0.198214531 | A2AJ88;F6UDU9;B0R009;B0R010;A0A0A6YWJ6 | Patatin-like phospholipase domain-containing protein 7 | Pnpla7 | 22.19011688 | 22.27937508 | NaN | 22.09622574 | NaN | 21.97683716 |
|  | 0.044354014 | -0.056043943 | Q6PE01 | U5 small nuclear ribonucleoprotein 40 kDa protein | Snrnp40 | 22.18452835 | 22.46774673 | 22.27358246 | NaN | 22.91296387 | 21.81636238 |
|  | 0.871311338 | -0.227392832 | A6H8H2;E9Q449 | DENN domain-containing protein 4C | Dennd4c | 22.17462921 | 21.80982399 | 21.91455269 | 22.24626541 | 22.25055122 | 22.08436775 |
|  | 0.881663516 | -0.362167994 | P47199;D3YUG9;V9GXY8;A0A0A6YXR4;D3YWU6;D3Z4Q4;D3Z2X0 | Quinone oxidoreductase | Cryz | 22.16683578 | 21.99638176 | 22.1653347 | 22.25729942 | 22.6860714 | NaN |
|  | 0.106851199 | 0.041595459 | Q69ZK0;I7HPV9 | Phosphatidylinositol 3,4,5-trisphosphate-dependent Rac exchanger 1 protein | Prex1 | 22.15838623 | 21.75139999 | 21.95123482 | 22.02035522 | 21.7630806 | 21.95279884 |
|  | 0.558397091 | -0.124902089 | Q6PB44;A0A0G2JEW5 | Tyrosine-protein phosphatase non-receptor type 23 | Ptpn23 | 22.15322876 | 22.14954376 | 21.88654709 | 22.26350212 | 22.10712433 | 22.19339943 |
|  | 0.287002222 | 0.118222555 | Q80ZX0;F6VJC5;F6YIN5;A2AA71;Q3U2P1 | Sec24 related gene family, member B (S. cerevisiae) | Sec24b | 22.14457321 | 22.07567978 | 22.31024742 | 21.83572578 | 21.99348259 | 22.34662437 |
|  | 0.071620666 | 0.023103396 | E9QNG1;B2RR82;Q9Z0R6;A0A1W2P775;A0A1W2P7G8 | Intersectin-2 | Itsn2 | 22.13964844 | 21.89936447 | 22.00956345 | NaN | 22.08008003 | 21.90609741 |
|  | 0.156347544 | -0.059915543 | E9QAI5;B2RQC6;G3UWN2;E9QAT6 | CAD protein;Glutamine-dependent carbamoyl-phosphate synthase;Aspartate carbamoyltransferase;Dihydroorotase | Cad | 22.13842964 | 22.56176376 | 22.19880486 | 22.355093 | 22.45828819 | 22.26536369 |
|  | 0.023215732 | 0.022212029 | Q61249;A0A0B4J1F7;Q9QZ29 | Immunoglobulin-binding protein 1 | Igbp1 | 22.13157082 | 22.18833733 | 22.6489315 | 22.61092567 | 21.99054337 | NaN |
|  | 0.231791905 | 0.080528895 | Q6PD26 | GPI transamidase component PIG-S | Pigs | 22.12641144 | 21.75037766 | 22.17901039 | 21.96940804 | 21.94113159 | 21.90367317 |
|  | 0.202595556 | -0.034950574 | A0A0G2JG95;Q8BX10 | Serine/threonine-protein phosphatase PGAM5, mitochondrial | Pgam5 | 22.11441994 | 22.19195557 | 22.28768349 | 22.23847198 | 22.22746849 | NaN |
|  | 1.04120417 | -0.349112829 | Q01965;E9PV93;E9PX73 | T-lymphocyte surface antigen Ly-9 | Ly9 | 22.11270332 | 22.0952282 | 22.55695534 | 22.51636314 | 22.63500214 | 22.66086006 |
|  | 0.032453633 | -0.009203911 | D3YUV1;Q99J45;A0A0J9YUZ6;A0A0J9YTX8;A0A0J9YUQ4 | Nuclear receptor-binding protein | Nrbp1 | 22.10306358 | 22.10671043 | 21.91783333 | 21.98724937 | NaN | 22.11623001 |
|  | 0.579341414 | 0.271251361 | Q9EPK2 | Protein XRP2 | Rp2 | 22.1022625 | NaN | 21.91016769 | 21.45483017 | 21.92183304 | 21.828228 |
|  | 0.134534429 | 0.042329152 | A2AI52;Q80U87 | Ubiquitin carboxyl-terminal hydrolase;Ubiquitin carboxyl-terminal hydrolase 8 | Usp8 | 22.09423065 | 22.22172737 | 22.39089584 | 22.16210938 | NaN | 22.22446823 |
|  | 0.160505696 | 0.06421725 | P58044;G3XA48;H3BLF8;H3BLP1 | Isopentenyl-diphosphate Delta-isomerase 1 | Idi1 | 22.08514786 | 22.12316322 | 21.76283836 | 22.00600624 | 21.73223114 | 22.04026031 |
|  | 0.396721156 | -0.125944773 | Q8VDQ1;Q3TXN1;D6RGL6 | Prostaglandin reductase 2 | Ptgr2 | 22.08238792 | 22.27602768 | 22.2299633 | 22.46261215 | NaN | 22.18153 |
|  | 0.099931086 | -0.029078166 | Q9D832 | DnaJ homolog subfamily B member 4 | Dnajb4 | 22.07868004 | 22.11359406 | 21.78851509 | 21.9913044 | 22.02718925 | 22.04953003 |
|  | 0.735568578 | -0.195328395 | Q9R1J0 | Sterol-4-alpha-carboxylate 3-dehydrogenase, decarboxylating | Nsdhl | 22.07391739 | 22.09429359 | 22.14164543 | 22.09100342 | 22.29793358 | 22.5069046 |
|  | 0.575846517 | -0.164257685 | Q9EPQ7;D3YU00 | StAR-related lipid transfer protein 5 | Stard5 | 22.06736565 | 21.78783798 | 21.98370552 | 22.04913139 | 21.98321915 | 22.29933167 |
|  | 0.292216971 | -0.264178276 | Q6NXL1 | Sec24 related gene family, member D (S. cerevisiae) | Sec24d | 22.0639801 | 21.61589432 | NaN | 21.85836411 | 22.34986687 | NaN |
|  | 0.702766567 | 0.104890823 | P59108;A0A0R4J1D0;A0A1D5RLP0 | Copine-2 | Cpne2 | 22.06259918 | 21.98259163 | 22.10683823 | 21.86139679 | 22.05579758 | 21.9201622 |
|  | 0.317760188 | 0.125705719 | Q6PF96;Q921G7 | Electron transfer flavoprotein-ubiquinone oxidoreductase, mitochondrial | Etfdh | 22.04776764 | 21.72895432 | 21.97582245 | 21.87421417 | 21.53687286 | 21.96434021 |
|  | 0.503597353 | -0.192071279 | P70206;Q80UG2 | Plexin-A1 | Plxna1 | 22.04650116 | 22.26896477 | 21.76011848 | 22.36546326 | 22.1867981 | 22.0995369 |
|  | 0.959283714 | 0.242323558 | Q571E4;Q8CC47 | N-acetylgalactosamine-6-sulfatase | Galns | 22.04026031 | 22.03560257 | 22.38065529 | 21.96105766 | 21.85528755 | 21.91320229 |
|  | 0.349093699 | -0.172048251 | F6WMJ3;Q8K4I3;A2AFJ8;H3BKQ0;H3BJ53 | Rho guanine nucleotide exchange factor 6 | Arhgef6 | 22.03691101 | 22.55363274 | 22.39286232 | 22.48478127 | 22.51491928 | NaN |
|  | 0.384165341 | -0.043737094 | Q8CAA7;E0CX81 | Glucose 1,6-bisphosphate synthase | Pgm2l1 | 22.03143692 | 22.01576805 | 21.97788429 | 21.99669266 | NaN | 22.10750771 |
|  | 0.176206295 | -0.128844579 | Q8K2C7 | Protein OS-9 | Os9 | 22.03140259 | 21.98165321 | 22.31005287 | 21.91958046 | 22.55351448 | NaN |
|  | 0.474762597 | -0.321648916 | Q9Z2L7;J3QMV5 | Cytokine receptor-like factor 3 | Crlf3 | 22.02988625 | 22.3999176 | 22.68459511 | 22.97042656 | 22.24858284 | 22.8603363 |
|  | 1.17034165 | 0.279860179 | H3BKD4;H3BKE6;E9QMI7;E9QMJ1;H3BL41;H3BJY2;E9QN63;Q9QWY8 | Arf-GAP with SH3 domain, ANK repeat and PH domain-containing protein 1 | Asap1 | 22.02988625 | 21.99855232 | 22.22323036 | 21.85976791 | 21.74829102 | NaN |
|  | 0.689459666 | 0.240439097 | Q91WC0;F2Z420;F2Z438;D6RCY6 | Histone-lysine N-methyltransferase setd3 | Setd3 | 22.0276947 | 22.05569839 | 22.27514648 | 21.99092293 | 21.60440063 | 22.04189873 |
|  | 0.202445275 | -0.102643967 | Q9CQK7 | RWD domain-containing protein 1 | Rwdd1 | 22.02350616 | 22.29030228 | 22.09042168 | 21.91823387 | 22.25888252 | 22.53504562 |
|  | 0.202463526 | 0.032747587 | Q925I1;V9GWS5;H3BKI6;H3BK90 | ATPase family AAA domain-containing protein 3 | Atad3 | 22.02279472 | 21.95712852 | 21.86071587 | 21.91517258 | NaN | 21.91309166 |
|  | 0.240848442 | -0.1616141 | Q8BVF2;A0A0A6YXV1 | Phosducin-like protein 3 | Pdcl3 | 22.00751114 | 22.59231377 | 22.05125618 | NaN | 22.49240494 | 22.26487732 |
|  | 0.445286738 | -0.261315664 | A0A6I8MX27;P16125;D3Z7F0;A0A0N4SVV8 | L-lactate dehydrogenase B chain;L-lactate dehydrogenase | Ldhb | 21.99855232 | 21.63418198 | 22.45731163 | 22.14510155 | 22.29611206 | 22.43277931 |
|  | 0.28669528 | -0.099285444 | Q99LD9;D3Z487;D3Z7J6 | Translation initiation factor eIF-2B subunit beta | Eif2b2 | 21.99081993 | 22.0146122 | 22.28070641 | NaN | 22.27920532 | 22.11012459 |
|  | 0.073892072 | -0.030659358 | E9Q2M9;E9PV60 | WD repeat and FYVE domain containing 4 | Wdfy4 | 21.98516655 | 21.70070648 | 22.15613365 | 22.04640198 | 21.8590107 | 22.02857208 |
|  | 0.062877366 | 0.026564916 | Q9QZB9;H3BJ75 | Dynactin subunit 5 | Dctn5 | 21.97865295 | 22.21608353 | 22.10098076 | 22.08316803 | 21.84176445 | 22.29109001 |
|  | 0.150283691 | -0.151073456 | F8WJB9;E9PVP4;A0A1Y7VJA2;P70429 | Ena/VASP-like protein | Evl | 21.96912575 | 22.24901772 | 21.83337212 | 21.72533989 | 22.61115074 | NaN |
|  | 0.334537681 | 0.218889236 | Q9JK23;A0A3B2W3V4;A0A3B2WB82 | Proteasome assembly chaperone 1 | Psmg1 | 21.9542923 | 21.84835052 | NaN | 21.44521141 | 21.91965294 | NaN |
|  | 0.211658254 | 0.085448583 | P52825;A2A8E7;A2A8E8;A2A8E9 | Carnitine O-palmitoyltransferase 2, mitochondrial | Cpt2 | 21.94674492 | 21.79261017 | 21.9084816 | 21.50071907 | 21.9799099 | 21.91086197 |
|  | 0.029911156 | -0.009869258 | Q6P9Q4 | FH1/FH2 domain-containing protein 1 | Fhod1 | 21.9221611 | 21.97952652 | 22.24991417 | 22.00734138 | 22.02184677 | 22.15202141 |
|  | 0.502117574 | -0.179162979 | H7BX88;P47934;B7ZDD7;A2AWJ5;F7BF80 | Carnitine O-acetyltransferase | Crat | 21.91805267 | 21.83198166 | NaN | 21.92661858 | 22.18174171 | NaN |
|  | 0.653377587 | -0.360629082 | A2AF47;A2AF67;A2AF65;A0A1D5RLE0;A0A5F8MPL9;E9QMR2;A0A1D5RMM1;F8VPN7;Q8BIK4 | Dedicator of cytokinesis protein 11 | Dock11 | 21.88487053 | 21.863554 | 22.41560364 | 22.4529705 | 22.37763977 | NaN |
|  | 0.578865403 | -0.195232391 | P35235 | Tyrosine-protein phosphatase non-receptor type 11 | Ptpn11 | 21.85391808 | 21.84724236 | 21.58916283 | 22.0755825 | NaN | 21.84176445 |
|  | 0.788813654 | 0.371271769 | Q61102 | ATP-binding cassette sub-family B member 7, mitochondrial | Abcb7 | 21.83174896 | 21.72225761 | 22.00336647 | 21.71141624 | 21.25095558 | NaN |
|  | 0.061097943 | -0.0413812 | Q8R5H1 | Ubiquitin carboxyl-terminal hydrolase 15 | Usp15 | 21.81260681 | 22.06713676 | 21.73508453 | 22.28616142 | 21.55027771 | 21.90253258 |
|  | 0.293776503 | 0.200442314 | Q64735;A0A0A6YVT1;A0A0A6YXN9;A0A0A6YY75;A0A0A6YXR6 | Complement component receptor 1-like protein | Cr1l | 21.80487061 | 21.85722542 | NaN | 21.3808918 | 21.8803196 | NaN |
|  | 0.236556877 | 0.058457057 | Q09200;A0A1W2P6F0 | Beta-1,4 N-acetylgalactosaminyltransferase 1 | B4galnt1 | 21.76615906 | 21.87361336 | 21.96194077 | 21.88918686 | 21.72837448 | NaN |
|  | 0.110115815 | -0.040492376 | P25799 | Nuclear factor NF-kappa-B p105 subunit;Nuclear factor NF-kappa-B p50 subunit | Nfkb1 | 21.74906921 | 21.61728477 | 21.41426277 | 21.67912865 | NaN | 21.58893394 |
|  | 0.471500977 | 0.168113073 | Q9D338 | 39S ribosomal protein L19, mitochondrial | Mrpl19 | 21.73657227 | 21.72425652 | 21.76320267 | 21.54321289 | 21.3226757 | 21.85380363 |
|  | 0.303097971 | 0.171902339 | Q8BJS4;E0CY39 | SUN domain-containing protein 2 | Sun2 | 21.62929726 | 21.64662743 | 22.14749527 | 21.57925224 | 21.69255638 | NaN |
|  | 0.702359625 | -0.165898005 | Q9DBR7;A0A1W2P750 | Protein phosphatase 1 regulatory subunit 12A | Ppp1r12a | 21.6003685 | 21.56285858 | 21.72579765 | 21.8087635 | 21.95454025 | 21.62341499 |
|  | 0.891969909 | -0.150472641 | Q3TFQ1;Q3UBW1 | SPRY domain-containing protein 7 | Spryd7 | 21.55529594 | 21.62435341 | 21.74480629 | 21.77908516 | NaN | 21.80483055 |
|  | 0.098730626 | -0.04454422 | Q6PAM1;A8Y5J8;A2ADZ3;A2ADZ4;A2ADZ2 | Alpha-taxilin | Txlna | 21.53164864 | 21.44879341 | 21.77072144 | 21.70277786 | 21.37565231 | 21.80636597 |
|  | 0.590410876 | 0.132754008 | E9PVG7;P97820;B7ZNR9;B2RUE8;A0A0A6YWR8;F8VPL5;A0A0A6YWM8;A0A0A6YW53;P83510;B9EKN8;E9PUL9;E0CY98;E0CXD6;E0CZF8;E0CZD7;B2RQ80;A0A0A6YWJ2;A0A0A6YVZ8;A0A0A6YXE5;A0A0A6YVR7 | Mitogen-activated protein kinase kinase kinase kinase 4 | Map4k4 | 21.28714943 | 21.52831268 | 21.3494339 | 21.27963066 | 21.23145866 | NaN |
|  | 0.071864431 | 0.020213445 | Q7TMY8;A2AFQ0;F6XP90 | E3 ubiquitin-protein ligase HUWE1 | Huwe1 | 21.21945381 | 21.13062859 | 21.29170799 | 21.13946152 | 21.07796288 | 21.36372566 |
|  | 0.967407765 | -0.273171743 | D3Z0M9 | RNA helicase | Ddx23 | 21.1958046 | 21.11426163 | 21.31080055 | 21.35250854 | 21.60774612 | NaN |
|  | 0.516223964 | 0.276262283 | Q3TET1;Q3UI47;B1AQZ2;P28741;B1AQZ5;Q61771 | Kinesin-like protein;Kinesin-like protein KIF3A | Kif3a | 21.14513397 | 21.25764656 | NaN | 20.7312355 | 21.11902046 | NaN |
|  | 0.345358335 | 0.130945206 | Q4VBE8 | WD repeat-containing protein 18 | Wdr18 | 21.13018799 | 21.26304436 | 21.32493019 | 20.93091965 | 21.28596497 | NaN |
|  | 0.354253223 | 0.093296051 | Q3TYL7;Q9QYJ3 | DnaJ homolog subfamily B member 1 | Dnajb1 | 20.99258423 | 21.08485603 | 21.22299576 | 21.08841896 | 20.92527962 | NaN |
|  | 1.115051936 | -0.34428215 | A0A1D5RLG3;Q80UJ7 | Rab3 GTPase-activating protein catalytic subunit | Rab3gap1 | 20.95610046 | 21.09847641 | 20.95581818 | 21.20329285 | 21.62203026 | 21.2179184 |
|  | 0.093272633 | -0.036753337 | G5E8A0;A0A338P6F2;Q8CI95 | Oxysterol-binding protein;Oxysterol-binding protein-related protein 11 | Osbpl11 | 20.94785118 | 21.24580193 | 21.27486229 | 21.00470352 | 21.2873745 | 21.28669739 |
|  | 0.327387824 | 0.103764852 | A0A1B0GQY8;D6RHM6;D3Z2F3;A0A1B0GSM3;A0A1B0GS63;Q8K221 | Arfaptin-2 | Arfip2 | 20.80207062 | 20.5945034 | 20.56001472 | 20.44151688 | 20.65534592 | NaN |
