# Supplementary material for: Intracellular iron accumulation facilitates mycobacterial infection in old mouse macrophages
Source: GeroScience. 2023 Dec 30;46(2):2739–54. doi: 10.1007/s11357-023-01048-1 (PMC10828278; doi:10.1007/s11357-023-01048-1)
Supplement: Supplementary file 9 — Supplementary file9 (DOCX 36 KB) [file 11357_2023_1048_MOESM9_ESM.docx]

| **Significant** | **-LOG(P-value)** | **Fold Change (Log2)** | **Protein IDs** | **Protein names** | **Gene names** | **id** | **Uninfected Old BMMs** | | | **Uninfected Young BMMs** | | |
| --- | --- | --- | --- | --- | --- | --- | --- | --- | --- | --- | --- | --- |
|  |  |  |  |  |  |  | **LFQ intensity 1967_br1_tr1** | **LFQ intensity 1967_br1_tr2** | **LFQ intensity 1967_br1_tr3** | **LFQ intensity 1967_br3_tr1** | **LFQ intensity 1967_br3_tr2** | **LFQ intensity 1967_br3_tr3** |
| + | 3.197253231 | 0.905281703 | A0A1B0GR60;Q9CPX4;P29391;P49945;A0A1Y7VNT9;A0A1B0GRH4 | Ferritin light chain 1 | Ftl1 | 244 | 26.43906212 | 26.71292877 | 26.57773209 | 25.72340775 | 25.71871376 | 25.57175636 |
| + | 4.24653539 | 0.765289307 | P09528;A0A494BA92;A0A494B9D4;A0A494BAP3 | Ferritin heavy chain | Fth1 | 1230 | 26.74650383 | 26.6875248 | 26.64333916 | 25.93429375 | 25.9759407 | 25.87126541 |
|  | 0.940695364 | 0.296627045 | Q91V61;Q3U4F0;A0A494BB84 | Sideroflexin-3 | Sfxn3 | 2627 | 24.75450325 | 24.53552055 | 24.52796745 | 24.06882668 | 24.35682678 | 24.50245667 |
|  | 0.800077725 | 0.216241837 | Q8C872;Q62351 | Transferrin receptor protein 1 | Tfrc | 2071 | 22.86894989 | 22.59848404 | 22.78805542 | 22.69031906 | 22.55609131 | 22.36035347 |
| + | 1.468601747 | 0.200106939 | Q99JR1 | Sideroflexin-1 | Sfxn1 | 2740 | 23.96013832 | 24.07855034 | 23.97254944 | 23.90380096 | 23.76860428 | 23.73851204 |
| + | 1.442855624 | 0.18570137 | P28271 | Cytoplasmic aconitate hydratase | Aco1 | 1368 | 23.51280022 | 23.47877121 | 23.39689064 | 23.3474617 | 23.18300056 | 23.30089569 |
| + | 2.017220882 | 0.131970088 | Q99KI0;A0A2R8W744;A0A2R8VHM8;A0A2R8VJW0 | Aconitate hydratase, mitochondrial | Aco2 | 2757 | 25.53204727 | 25.56964302 | 25.51749229 | 25.42943573 | 25.43342209 | 25.36041451 |
|  | 0.155806522 | 0.029919942 | Q6P069 | Sorcin | Sri | 2128 | 24.88460159 | 24.74021149 | 24.73003387 | 24.82298851 | 24.65354347 | 24.78855515 |
|  | 0.07062382 | -0.013228734 | O70252;D3YX62;D3YXN4;D3Z4A2 | Heme oxygenase 2 | Hmox2 | 1129 | 24.24670029 | 24.27632523 | 24.12691689 | 24.24670029 | 24.30159378 | 24.14133453 |
|  | 1.143046086 | -0.074849447 | A0A0R4J0I9;Q91ZX7;Q3U5J2;D3Z5M3 | Low-density lipoprotein receptor-related protein 1 | Lrp1 | 159 | 25.10673332 | 25.09655571 | 25.06780052 | 25.21537971 | 25.16357613 | 25.11668205 |
| + | 3.838160344 | -0.595802307 | A0A3Q4EBK4;Q6P1B9;O08539;A0A3Q4EBR8 | Myc box-dependent-interacting protein 1 | Bin1 | 387 | 23.64339447 | 23.72004509 | 23.67163277 | 24.22036934 | 24.34170532 | 24.26040459 |
| + | 4.568384001 | -1.435453415 | F7CJN9;Q921I1;F7BAE9 | Serotransferrin | Trf;Tf | 909 | 26.05895424 | 25.87420082 | 25.88313293 | 27.3698616 | 27.42478371 | 27.32800293 |

**Supplementary Table 3A: Uninfected Old BMMs *vs* Uninfected Young BMMs**

**Supplementary Table 3B: *M.avium*-infected Old BMMs vs *M.avium*-infected Young BMMs**

| **Significant** | **-LOG(P-value)** | **Fold Change (Log2)** | **Protein IDs** | **Protein names** | **Gene names** | **id** | ***M.avium*-infected Old BMMs** | | | ***M.avium*-infected Young BMMs** | | |
| --- | --- | --- | --- | --- | --- | --- | --- | --- | --- | --- | --- | --- |
|  |  |  |  |  |  |  | **LFQ intensity 1967_br2_tr1** | **LFQ intensity 1967_br2_tr2** | **LFQ intensity 1967_br2_tr3** | **LFQ intensity 1967_br4_tr1** | **LFQ intensity 1967_br4_tr2** | **LFQ intensity 1967_br4_tr3** |
| + | 3.143907111 | 0.559800466 | P09528;A0A494BA92;A0A494B9D4;A0A494BAP3 | Ferritin heavy chain | Fth1 | 1230 | 26.01039124 | 26.1908226 | 26.02290916 | 25.4913044 | 25.5368557 | 25.51656151 |
| + | 2.868953931 | 0.541412354 | A0A1B0GR60;Q9CPX4;P29391;P49945;A0A1Y7VNT9;A0A1B0GRH4 | Ferritin light chain 1 | Ftl1 | 244 | 25.92892838 | 25.93012428 | 25.90150261 | 25.37706375 | 25.49637413 | 25.26288033 |
| + | 1.373317672 | 0.387318929 | Q8C872;Q62351 | Transferrin receptor protein 1 | Tfrc | 2071 | 22.82596016 | 22.73640633 | 22.81113625 | 22.34892082 | 22.21347427 | 22.64915085 |
| + | 2.444926008 | 0.268542608 | Q99KI0;A0A2R8W744;A0A2R8VHM8;A0A2R8VJW0 | Aconitate hydratase, mitochondrial | Aco2 | 2757 | 25.66979599 | 25.58832169 | 25.70065308 | 25.38732338 | 25.33408546 | 25.43173409 |
| + | 1.613278713 | 0.21810468 | Q6P069 | Sorcin | Sri | 2128 | 25.12900162 | 25.05668259 | 25.08160019 | 24.77052116 | 24.97193527 | 24.87051392 |
| + | 1.750194894 | 0.124872208 | Q91V61;Q3U4F0;A0A494BB84 | Sideroflexin-3 | Sfxn3 | 2627 | 24.470541 | 24.53552055 | 24.55021858 | 24.40360069 | 24.42412758 | 24.35393524 |
|  | 1.119418357 | 0.120079676 | Q99JR1 | Sideroflexin-1 | Sfxn1 | 2740 | 23.8582325 | 23.85195732 | 23.92614746 | 23.77535057 | 23.82609558 | 23.6746521 |
|  | 0.879590173 | 0.063374837 | A0A0R4J0I9;Q91ZX7;Q3U5J2;D3Z5M3 | Low-density lipoprotein receptor-related protein 1 | Lrp1 | 159 | 24.98002243 | 24.99912071 | 25.02529716 | 24.94761848 | 24.88049698 | 24.98620033 |
|  | 0.489096559 | -0.075431188 | P28271 | Cytoplasmic aconitate hydratase | Aco1 | 1368 | 23.36666298 | 23.22271538 | 23.21483803 | 23.35407066 | 23.41660881 | 23.25983047 |
|  | 1.085200297 | -0.231494904 | O70252;D3YX62;D3YXN4;D3Z4A2 | Heme oxygenase 2 | Hmox2 | 1129 | 24.29235458 | 23.97962952 | 24.07111931 | 24.27206039 | 24.36739731 | 24.39813042 |
| + | 3.305986781 | -0.505087535 | A0A3Q4EBK4;Q6P1B9;O08539;A0A3Q4EBR8 | Myc box-dependent-interacting protein 1 | Bin1 | 387 | 23.88362312 | 23.89235878 | 23.85728264 | 24.28911972 | 24.41415787 | 24.44524956 |
|  | 0 | NaN | F7CJN9;Q921I1;F7BAE9 | Serotransferrin | Trf;Tf | 909 | NaN | NaN | NaN | 27.32225418 | 27.13819313 | 27.45904541 |

**Supplementary Table 3C: *M.avium*-infected Old BMMs vs Uninfected Old BMMs**

| **Significant** | **-LOG(P-value)** | **Fold Change (Log2)** | **Protein IDs** | **Protein names** | **Gene names** | **id** | **Uninfected Old BMMs** | | | ***M.avium*-infected Old BMMs** | | |
| --- | --- | --- | --- | --- | --- | --- | --- | --- | --- | --- | --- | --- |
|  |  |  |  |  |  |  | **LFQ intensity 1967_br1_tr1** | **LFQ intensity 1967_br1_tr2** | **LFQ intensity 1967_br1_tr3** | **LFQ intensity 1967_br2_tr1** | **LFQ intensity 1967_br2_tr2** | **LFQ intensity 1967_br2_tr3** |
| + | 2.304007654 | 0.304145813 | Q6P069 | Sorcin | Sri | 2128 | 24.88460159 | 24.74021149 | 24.73003387 | 25.12900162 | 25.05668259 | 25.08160019 |
| + | 2.890455378 | 0.199397405 | A0A3Q4EBK4;Q6P1B9;O08539;A0A3Q4EBR8 | Myc box-dependent-interacting protein 1 | Bin1 | 387 | 23.64339447 | 23.72004509 | 23.67163277 | 23.88362312 | 23.89235878 | 23.85728264 |
| + | 1.426195914 | 0.113196055 | Q99KI0;A0A2R8W744;A0A2R8VHM8;A0A2R8VJW0 | Aconitate hydratase, mitochondrial | Aco2 | 2757 | 25.53204727 | 25.56964302 | 25.51749229 | 25.66979599 | 25.58832169 | 25.70065308 |
|  | 0.175959031 | 0.039337794 | Q8C872;Q62351 | Transferrin receptor protein 1 | Tfrc | 2071 | 22.86894989 | 22.59848404 | 22.78805542 | 22.82596016 | 22.73640633 | 22.81113625 |
|  | 0.485207177 | -0.08723704 | Q91V61;Q3U4F0;A0A494BB84 | Sideroflexin-3 | Sfxn3 | 2627 | 24.75450325 | 24.53552055 | 24.52796745 | 24.470541 | 24.53552055 | 24.55021858 |
|  | 2.144965586 | -0.088883082 | A0A0R4J0I9;Q91ZX7;Q3U5J2;D3Z5M3 | Low-density lipoprotein receptor-related protein 1 | Lrp1 | 159 | 25.10673332 | 25.09655571 | 25.06780052 | 24.98002243 | 24.99912071 | 25.02529716 |
|  | 0.421572583 | -0.102279663 | O70252;D3YX62;D3YXN4;D3Z4A2 | Heme oxygenase 2 | Hmox2 | 1129 | 24.24670029 | 24.27632523 | 24.12691689 | 24.29235458 | 23.97962952 | 24.07111931 |
|  | 1.316465769 | -0.124966939 | Q99JR1 | Sideroflexin-1 | Sfxn1 | 2740 | 23.96013832 | 24.07855034 | 23.97254944 | 23.8582325 | 23.85195732 | 23.92614746 |
| + | 1.498288266 | -0.194748561 | P28271 | Cytoplasmic aconitate hydratase | Aco1 | 1368 | 23.51280022 | 23.47877121 | 23.39689064 | 23.36666298 | 23.22271538 | 23.21483803 |
| + | 3.154643673 | -0.61774826 | P09528;A0A494BA92;A0A494B9D4;A0A494BAP3 | Ferritin heavy chain | Fth1 | 1230 | 26.74650383 | 26.6875248 | 26.64333916 | 26.01039124 | 26.1908226 | 26.02290916 |
| + | 2.928030386 | -0.656389236 | A0A1B0GR60;Q9CPX4;P29391;P49945;A0A1Y7VNT9;A0A1B0GRH4 | Ferritin light chain 1 | Ftl1 | 244 | 26.43906212 | 26.71292877 | 26.57773209 | 25.92892838 | 25.93012428 | 25.90150261 |
|  | 0 | NaN | F7CJN9;Q921I1;F7BAE9 | Serotransferrin | Trf;Tf | 909 | 26.05895424 | 25.87420082 | 25.88313293 | NaN | NaN | NaN |

**Supplementary Table 3D: *M.avium*-infected Young BMMs** ***vs*** **Uninfected Young BMMs**

| **Significant** | **-LOG(P-value)** | **Fold Change (Log2)** | **Protein IDs** | **Protein names** | **Gene names** | **id** | **Uninfected Young BMMs** | | | ***M.avium*-infected Young BMMs** | | |
| --- | --- | --- | --- | --- | --- | --- | --- | --- | --- | --- | --- | --- |
|  |  |  |  |  |  |  | **LFQ intensity 1967_br3_tr1** | **LFQ intensity 1967_br3_tr2** | **LFQ intensity 1967_br3_tr3** | **LFQ intensity 1967_br4_tr1** | **LFQ intensity 1967_br4_tr2** | **LFQ intensity 1967_br4_tr3** |
|  | 0.894993 | 0.115987 | O70252;D3YX62;D3YXN4;D3Z4A2 | Heme oxygenase 2 | Hmox2 | 1129 | 24.24670029 | 24.30159378 | 24.14133453 | 24.27206039 | 24.36739731 | 24.39813042 |
|  | 0.676969 | 0.115961 | Q6P069 | Sorcin | Sri | 2128 | 24.82298851 | 24.65354347 | 24.78855515 | 24.77052116 | 24.97193527 | 24.87051392 |
|  | 0.847008 | 0.108683 | A0A3Q4EBK4;Q6P1B9;O08539;A0A3Q4EBR8 | Myc box-dependent-interacting protein 1 | Bin1 | 387 | 24.22036934 | 24.34170532 | 24.26040459 | 24.28911972 | 24.41415787 | 24.44524956 |
|  | 0.260891 | 0.084518 | Q91V61;Q3U4F0;A0A494BB84 | Sideroflexin-3 | Sfxn3 | 2627 | 24.06882668 | 24.35682678 | 24.50245667 | 24.40360069 | 24.42412758 | 24.35393524 |
|  | 0.423627 | 0.066384 | P28271 | Cytoplasmic aconitate hydratase | Aco1 | 1368 | 23.3474617 | 23.18300056 | 23.30089569 | 23.35407066 | 23.41660881 | 23.25983047 |
|  | 0.251518 | -0.02338 | Q99KI0;A0A2R8W744;A0A2R8VHM8;A0A2R8VJW0 | Aconitate hydratase, mitochondrial | Aco2 | 2757 | 25.42943573 | 25.43342209 | 25.36041451 | 25.38732338 | 25.33408546 | 25.43173409 |
|  | 0.265747 | -0.04494 | Q99JR1 | Sideroflexin-1 | Sfxn1 | 2740 | 23.90380096 | 23.76860428 | 23.73851204 | 23.77535057 | 23.82609558 | 23.6746521 |
|  | 0.280743 | -0.06772 | F7CJN9;Q921I1;F7BAE9 | Serotransferrin | Trf;Tf | 909 | 27.3698616 | 27.42478371 | 27.32800293 | 27.32225418 | 27.13819313 | 27.45904541 |
|  | 0.339347 | -0.13174 | Q8C872;Q62351 | Transferrin receptor protein 1 | Tfrc | 2071 | 22.69031906 | 22.55609131 | 22.36035347 | 22.34892082 | 22.21347427 | 22.64915085 |
| + | 2.245877 | -0.22711 | A0A0R4J0I9;Q91ZX7;Q3U5J2;D3Z5M3 | Low-density lipoprotein receptor-related protein 1 | Lrp1 | 159 | 25.21537971 | 25.16357613 | 25.11668205 | 24.94761848 | 24.88049698 | 24.98620033 |
| + | 1.600177 | -0.29252 | A0A1B0GR60;Q9CPX4;P29391;P49945;A0A1Y7VNT9;A0A1B0GRH4 | Ferritin light chain 1 | Ftl1 | 244 | 25.72340775 | 25.71871376 | 25.57175636 | 25.37706375 | 25.49637413 | 25.26288033 |
| + | 3.618743 | -0.41226 | P09528;A0A494BA92;A0A494B9D4;A0A494BAP3 | Ferritin heavy chain | Fth1 | 1230 | 25.93429375 | 25.9759407 | 25.87126541 | 25.4913044 | 25.5368557 | 25.51656151 |
